# Synthetic Studies Towards the Core Structure of Nakadomarin A by a Thioamide-Based Strategy

**DOI:** 10.1002/ejoc.201301063

**Published:** 2013-11-14

**Authors:** Jai K Chavda, Panayiotis A Procopiou, Peter N Horton, Simon J Coles, Michael J Porter

**Affiliations:** [a] Department of Chemistry, University College LondonChristopher Ingold Building, 20 Gordon Street, London, WC1H 0AJ, UK; [b] GlaxoSmithKline Research & Development Limited, Medicines Research CentreGunnels Wood Road, Stevenage, Hertfordshire, SG1 2NY, UK; [c] UK National Crystallography Service, School of Chemistry, University of SouthamptonSouthampton, SO17 1BJ, UK

**Keywords:** Natural products, Alkaloids, Sigmatropic rearrangement, Multicomponent reactions

## Abstract

The tricyclic BCD substructure of the marine natural product nakadomarin A has been synthesised. The strategy utilised a key carbon–carbon bond-forming reaction between a furan and an *N*-acyliminium ion derived from a secondary thiolactam. In addition, a novel three-component coupling reaction between a thioamide, an allylic bromide and an isocyanate, leading to the establishment of two new stereogenic centres, is reported.

Two key steps in a projected total synthesis of nakadomarin A have been realised by using the unique chemistry of thioamides. Formation of the carbocyclic B ring can be effected by nucleophilic attack of a furan on a thiolactam-derived iminium ion, and the key quaternary centre can be established by a novel three-component coupling reaction.

## Introduction

Nakadomarin A (**1**), a hexacyclic alkaloid of the manzamine family, was isolated in 1997 by Kobayashi et al. from the marine sponge *Amphimedon* sp., collected off the Kerama Islands in Japan.1a It displays cytotoxicity against L1210 murine lymphoma cells, is an inhibitor of cyclin-dependent kinase 4 and shows antifungal and antibacterial activity.

The structure of nakadomarin A incorporates a tetracyclic furan-containing core (the ABCD ring system) fused to an eight-membered E ring and bridged by a fifteen-membered F ring. The combination of intriguing structural features and promising biological activity has made the compound a popular target for synthesis. Several total syntheses have been published,2a as have numerous reports on the synthesis of various substructures.^3^–^9^

**Scheme 1:**
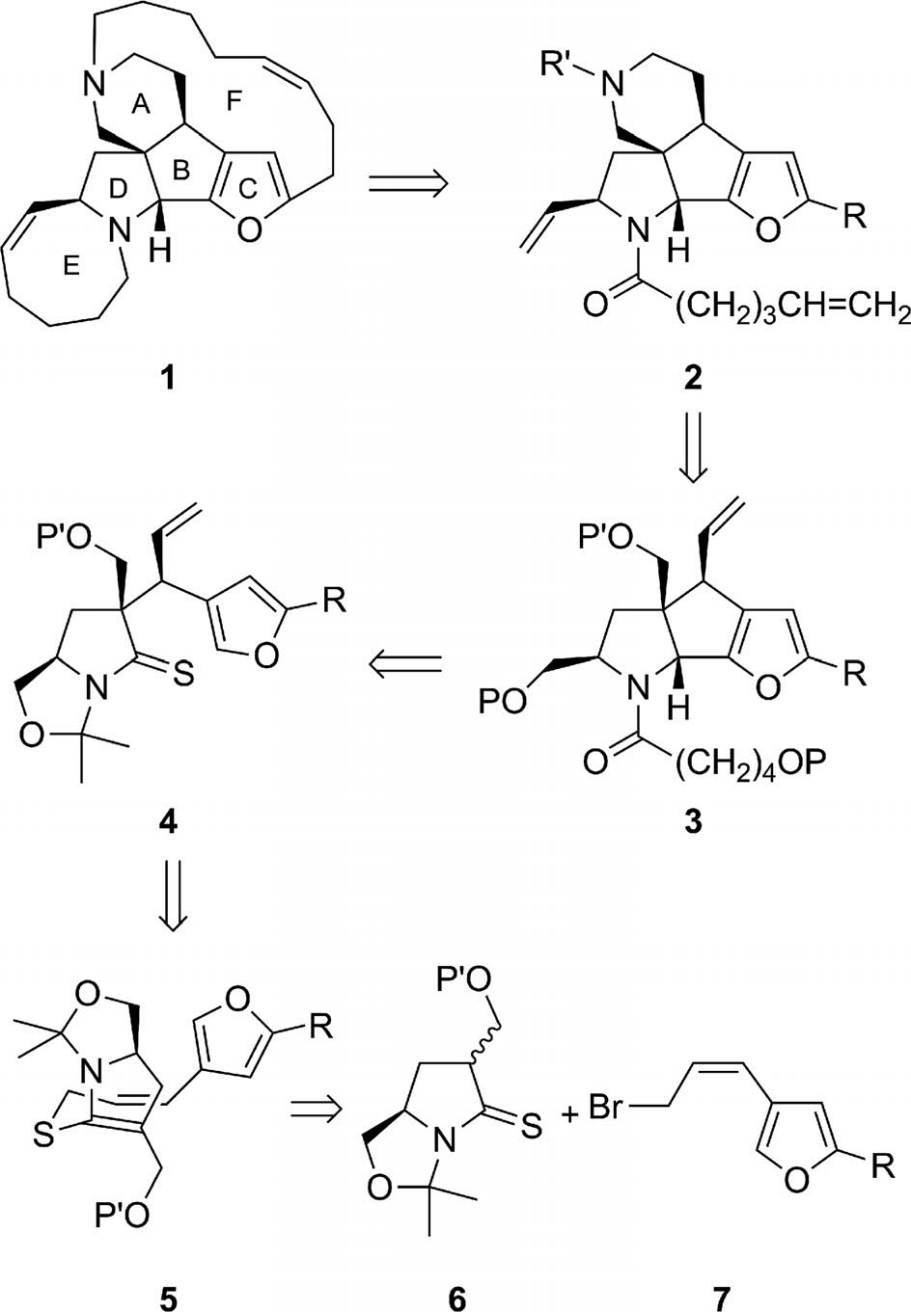
Retrosynthesesis of nakadomarin A (1).

Our initial retrosynthetic analysis of nakadomarin A is depicted in Scheme 1. Late-stage construction of the E and F rings by ring-closing metathesis would lead back to the tetracyclic substructure **2**. We planned to install the piperidine A ring through hydroboration of an alkene such as **3** and subsequent functionalisation. It was anticipated that formation of the cyclopentanoid B ring would be possible through cyclisation of the furan in **4** onto an electrophile derived from the thiolactam portion of the molecule with subsequent reductive removal of the sulfur. Padwa and co-workers have shown that mono-*N*-substituted thioamides, on treatment with α-bromoacyl chlorides, are transformed through *N*-acylation and *S*-alkylation into potent *N*-acyliminium ion electrophiles, which react intramolecularly with nucleophilic alkenes, indoles and benzenes.10a

We planned to establish the vicinal stereocentres of compound **4** through thia-Claisen rearrangement of a diene such as **5**; reaction on the *exo* face of the bicyclic system, through the chair conformation shown, should lead to the desired stereoisomer **4**. We expected that **5** would be accessible from the pyroglutamate-derived thiolactam **6** and (*Z*)-allylic bromide **7**.^11^

In this paper, we describe our model studies in two areas of this retrosynthesis: the cyclisation of a thiolactam-derived iminium ion to generate the BCD ring system of nakadomarin, and the development of a thia-Claisen rearrangement strategy to establish the stereogenic centres in a compound related to **4**.

## Results and Discussion

**Scheme 2:**
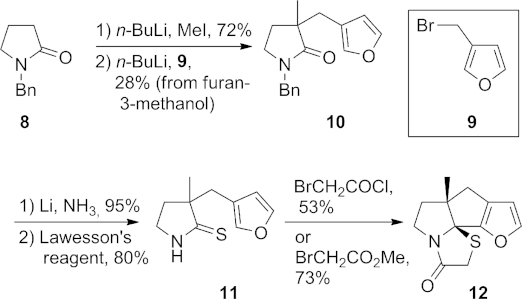
Synthesis of the BCD ring system (12).

We first focused on generating the carbocyclic B ring of nakadomarin A in a simple model of the BCD ring system (Scheme 2). *N*-Benzylpyrrolidin-2-one (**8**) was alkylated sequentially with iodomethane and 3-(bromomethyl)furan (**9**), prepared as a solution in diethyl ether,^12^ to afford lactam **10**. Removal of the benzyl group was effected with lithium in liquid ammonia, and thionation with Lawesson's reagent13a then gave thiolactam **11**. Treatment of this compound with bromoacetyl chloride10b in toluene at 100 °C led to the desired tetracycle **12** in 53 % yield. The same transformation could be effected in higher yield (73 %) by treatment of thiolactam **11** with methyl bromoacetate in toluene at reflux.

**Scheme 3:**
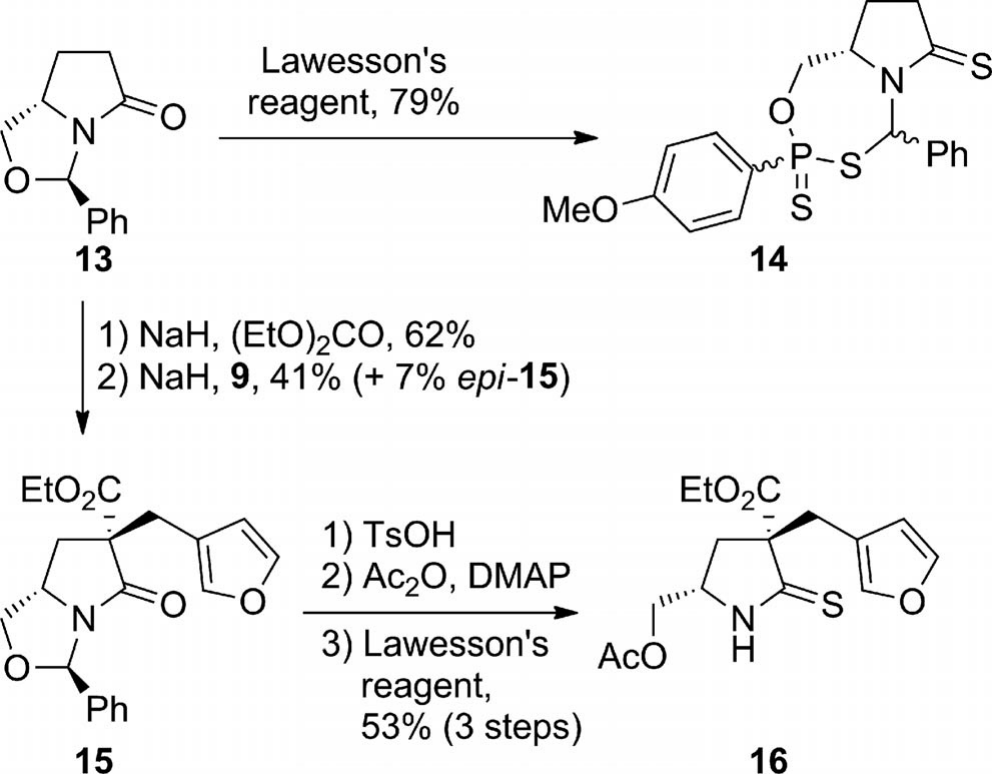
Synthesis of thiolactam 16.

Our next aim was to extend this study through the preparation of a compound with functionalities that would allow the construction of the eight-membered E ring of nakadomarin A. To this end, L-pyroglutaminol^14^ was protected as its benzylidene *N*,*O*-acetal **13** (Scheme 3).^15^ Attempts to convert **13** into its thiolactam analogue using Lawesson's reagent were unsuccessful, with the only product isolated being the 1,3,5,2-oxathiazaphosphepane **14**, in which a P–S unit has been inserted into the benzylic C–O bond. Clivio and co-workers^16^ previously reported the insertion of Lawesson's reagent into the C–O bond of a dihydrouracil nucleoside and found that the unwanted ArPS_2_ unit was expelled upon extended heating. In our case, however, further heating of **14** in pyridine led only to recovery of the starting material and so a revised strategy was adopted in which thionation took place at a later stage.

*C*-Ethoxycarbonylation of **13** was carried out according to a literature procedure.17a Subsequent alkylation with 3-(bromomethyl)furan (**9**) afforded **15** as a 5:1 diastereomeric mixture; the major diastereomer depicted was isolated in 41 % yield. Hydrolysis of the benzylidene group of **15** with *p*-toluenesulfonic acid was followed by acetylation of the resulting alcohol and thionation to give secondary thiolactam **16**.

**Scheme 4:**
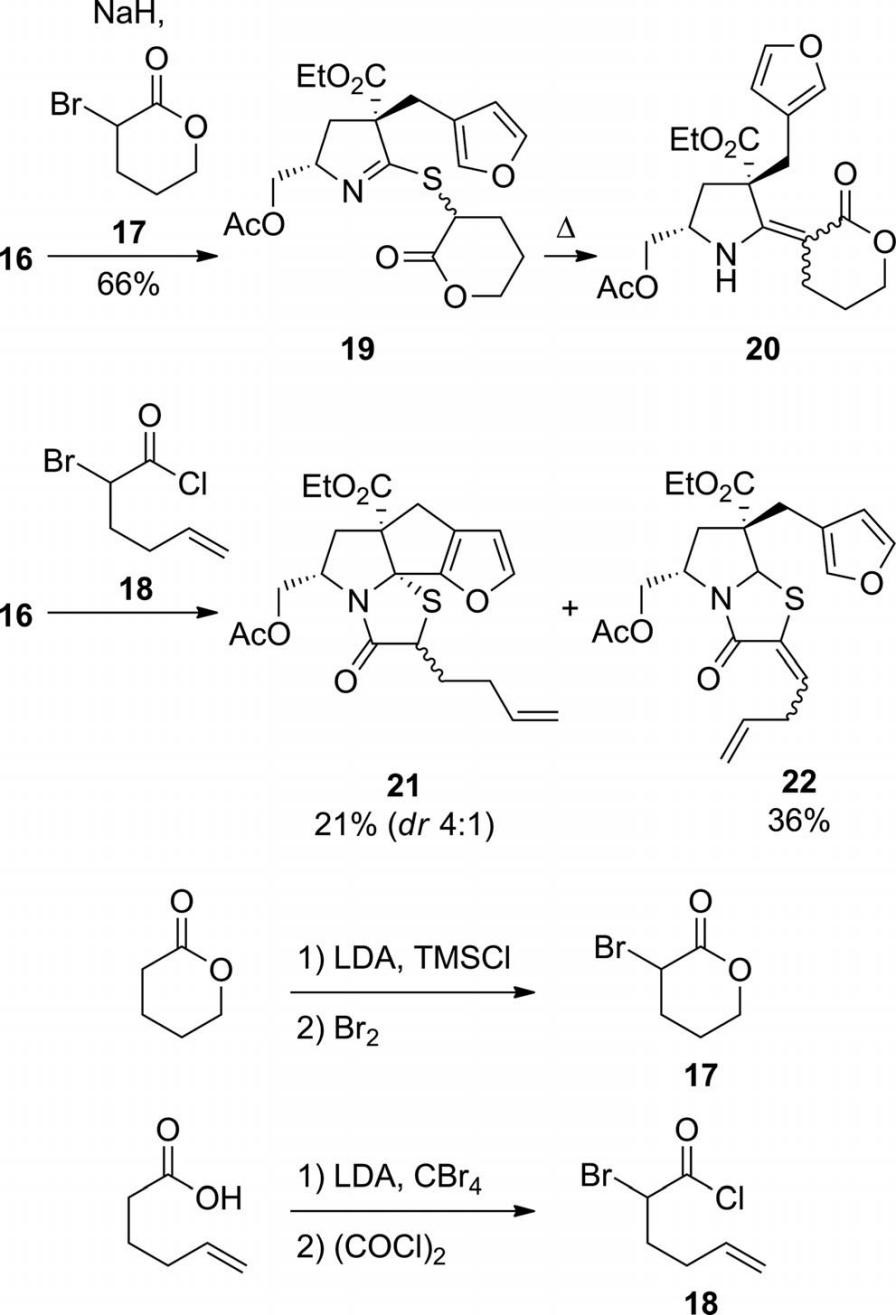
Reactions of thiolactam 16.

Two α-bromocarbonyl electrophiles **17** and **18**, each containing a further carbon chain for elaboration of the E ring, were prepared for reaction with **16** (Scheme 4). Lactone **17** was synthesised by conversion of δ-valerolactone into the corresponding TMS ketene acetal and treatment with bromine, and acyl chloride **18** was synthesised by double deprotonation of hex-5-enoic acid with LDA, bromination with carbon tetrabromide and then conversion of the resulting α-bromo acid into an acid chloride using oxalyl chloride.

Initial attempts to effect direct reaction of thiolactam **16** with lactone **17** resulted in recovery of the starting materials, and so the nucleophilicity of **16** was increased through deprotonation. Treatment of **16** with sodium hydride followed by reaction with α-bromo-δ-valerolactone (**17**) afforded thioimidate **19** as a mixture of diastereoisomers (Scheme 4). On heating in toluene in a sealed tube, no acylation of the nitrogen atom was observed; rather the substrate underwent an Eschenmoser sulfide contraction to give vinylogous carbamate **20** as a single unassigned geometric isomer. Given the lack of base or thiophile in the reaction medium, the observation of this product is somewhat surprising.

**Scheme 5:**
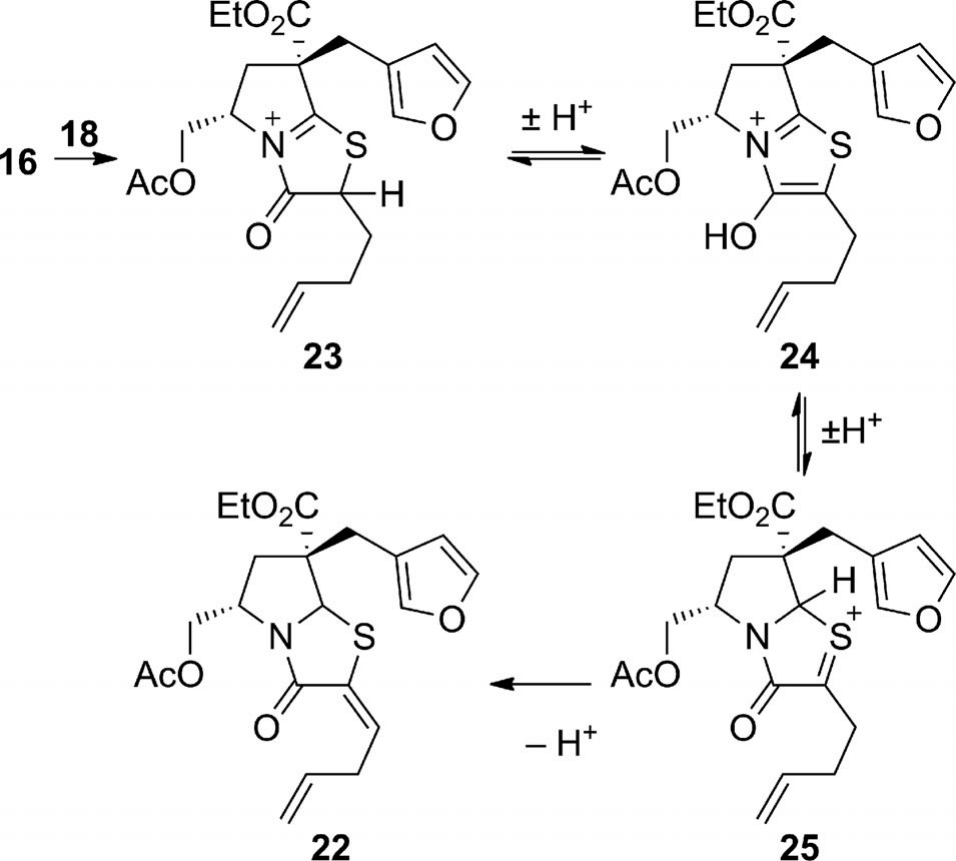
Formation of bicycle 22.

Reaction of thiolactam **16** with the unsaturated α-bromoacyl chloride **18** in toluene gave two major products. The desired product **21** was obtained in 21 % yield as an inconsequential 4:1 mixture of diastereomers. The major product, however, was the diene **22**, which was isolated as a single, but unidentified diastereoisomer. This unexpected product may be formed as shown in Scheme 5. Reaction of thiolactam **16** with acid chloride **18** through *S*-alkylation and *N*-acylation gives the expected *N*-acyliminium ion **23**, which can either react with the nucleophilic furan to produce the desired product **21**, or can undergo enolisation to give the aromatic thiazolium ion **24**. If ketonisation then occurs through addition of a proton at the carbon between the nitrogen and sulfur atoms, cation **25** results; this can lose a proton to give the diene **22**. All attempts to improve the yield of tetracycle **21** at the expense of the diene product **22**, through modification of the reaction conditions, were unsuccessful.

**Scheme 6:**
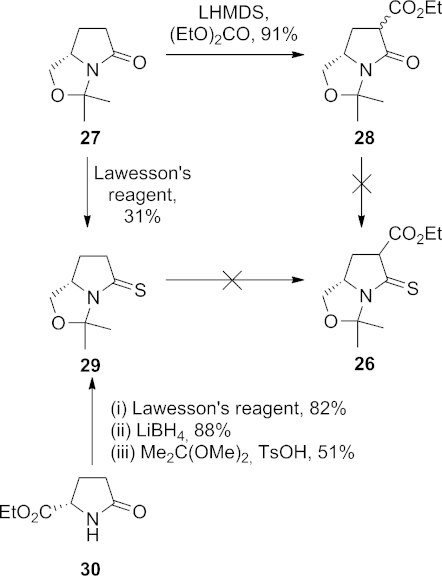
Attempted synthesis of thiolactam 26.

We next investigated the thia-Claisen step of Scheme 1, and initially targeted tertiary thiolactam **26** (Scheme 6). L-Pyroglutaminol was protected as its acetonide **27** by using 2,2-dimethoxypropane and 4-toluenesulfonic acid,^18^ and this was acylated with diethyl carbonate2g to give **28** as a mixture of diastereomers. Attempts to thionate this compound to give **26** by using either Lawesson's reagent or phosphorus pentasulfide were unsuccessful.

A reversal of the step order was attempted. Acetonide **27** could be thionated, albeit in low yield, with Lawesson's reagent, whereas the use of other thionating reagents failed; decomposition was observed when 2,4-bis(phenylthio)-1,3-dithia-2,4-diphosphetane-2,4-disulfide (Yokoyama's reagent)^19^ was employed, and the use of Bergman's P_4_S_10_–pyridine complex^20^ led to recovery of the starting material. A slightly more efficient synthesis of **29** could be achieved by starting from ethyl L-pyroglutamate (**30**)^21^ by thionation with Lawesson's reagent in 82 % yield,^22^ followed by the selective reduction of the ester with lithium borohydride and protection using 2,2-dimethoxypropane. However, we were unable to conduct a successful α-acylation of thiolactam **29**.

**Scheme 7:**
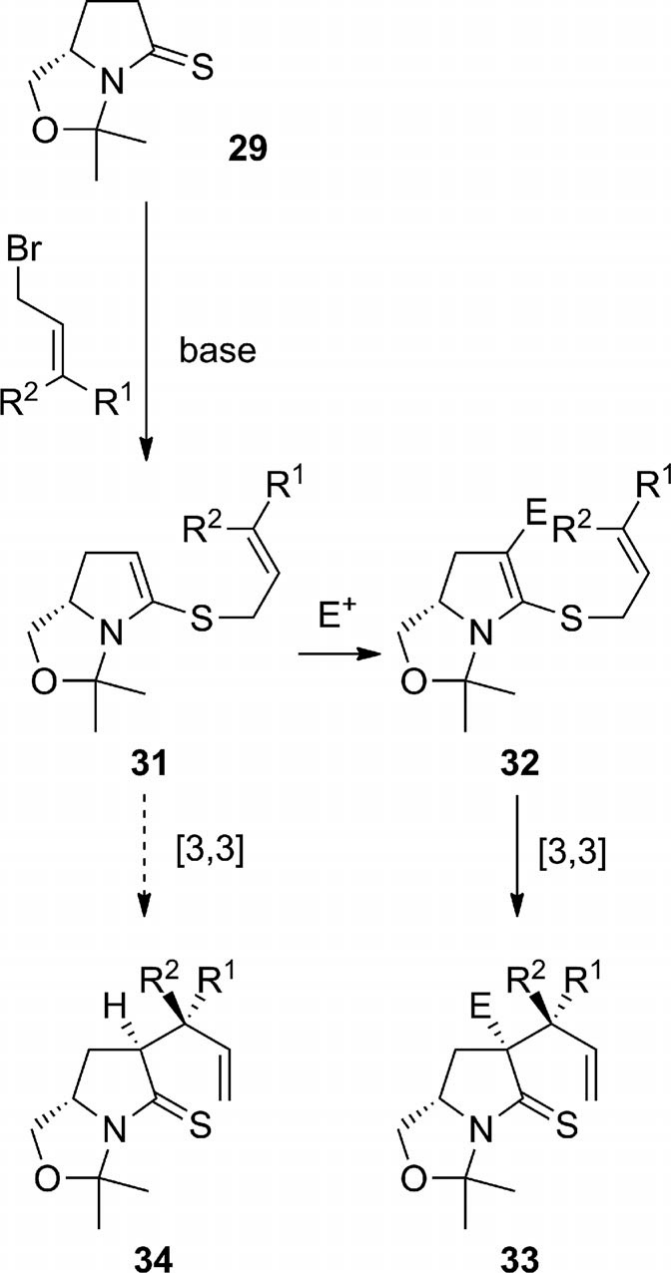
Proposed three-component coupling to give 33.

Having failed in attempts to prepare the desired thia-Claisen substrate **26**, we instead conceived a three-component coupling approach that would allow the stereospecific formation of two new C–C bonds at the α position of thiolactam **29** in a single laboratory operation. This strategy is illustrated in Scheme 7.

The reaction of thiolactam **29** with an allylic bromide should give *N*,*S*-ketene acetal **31**. Such compounds are known to readily undergo substitution reactions with electrophiles such as acid chlorides^23^ or isocyanates^24^ at the nucleophilic carbon atom; our hope was that we could intercept the *N*,*S*-ketene acetal **31** with such an electrophile to generate **32**, which would then undergo the desired [3,3]-sigmatropic rearrangement to give the product **33**. An obvious potential pitfall of this chemistry is that the 1,5-diene moiety of **31** may undergo the sigmatropic rearrangement more rapidly than it reacts with the electrophile; if this were the case, the simple thia-Claisen product **34** would be obtained.

**Scheme 8:**
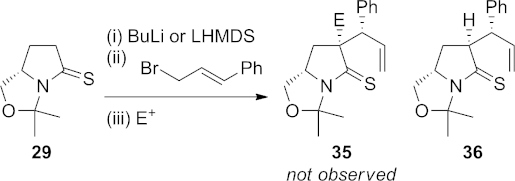
Attempted three-component coupling reaction using (*E*)-cinnamyl bromide.

Initial studies on the three-component coupling reaction were carried out by using (*E*)-cinnamyl bromide as the allylic bromide component. Deprotonation of **29** with either *n*-butyllithium or lithium hexamethyldisilazide (LHMDS) at low temperature was followed by treatment with (*E*)-cinnamyl bromide. Once alkylation of the sulfur was complete, as judged by TLC, the second electrophile was added and the reaction mixture warmed to room temperature (Scheme 8).

A wide range of electrophiles were tested: ethyl chloroformate, acetic formic anhydride, phenyl isocyanate, chlorosulfonyl isocyanate, trichloroacetyl isocyanate and phosgene. However, under none of the conditions tried could any of the putative three-component coupling products **35** be obtained; rather, the only compound isolated was the product **36** from rearrangement of the first-formed *N*,*S*-ketene acetal. This clearly indicated that the rate of acylation of the *N*,*S*-ketene acetal was not competitive with the rate of sigmatropic rearrangement.

**Figure 1:**
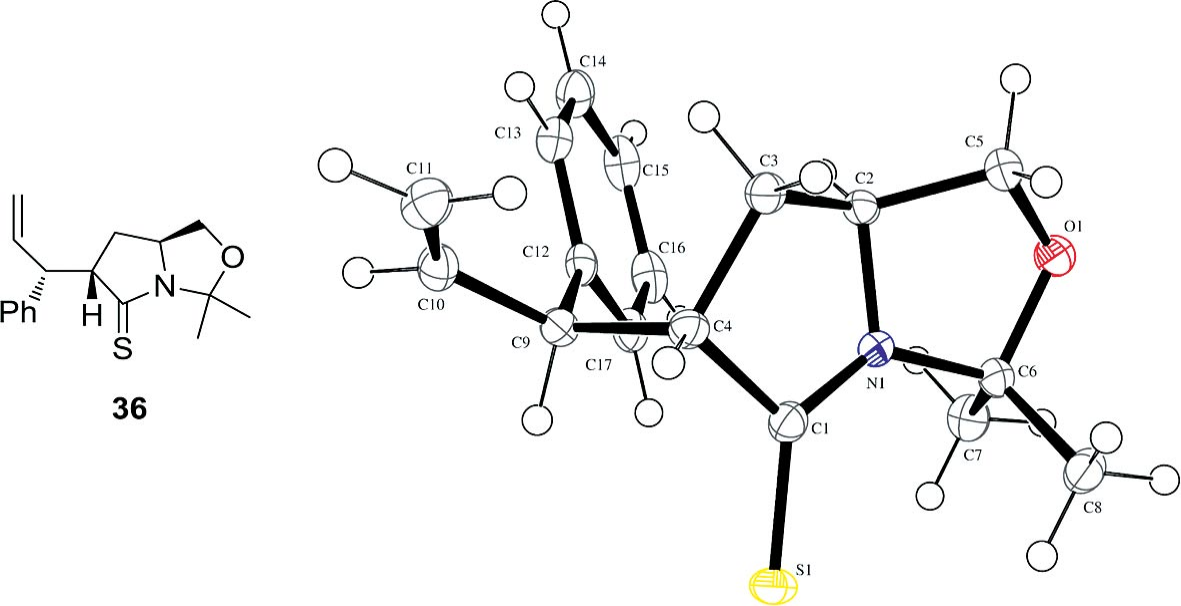
X-ray crystal structure of 36.

A control experiment carried out in the absence of the second electrophile showed that the [3,3]-sigmatropic rearrangement to form **36** was indeed rapid, proceeding in less than 5 min at room temperature. Under these conditions, **36** could be isolated in 73 % yield as a 25:1 mixture of diastereoisomers. The stereochemistry of the major isomer was confirmed by X-ray crystallography (Figure 1).^25^,^26^

Rawal and co-workers previously reported that *N*,*S*-ketene acetals derived from (*Z*)-allylic bromides rearrange much more slowly than those derived from the corresponding *E* isomers (3 h at reflux in THF vs. warming from –78 °C to room temperature).^27^ In light of this observation, and given the requirement for a (*Z*)-allylic bromide in the projected total synthesis (Scheme 1), we next investigated the use of (*Z*)-cinnamyl bromide.

**Scheme 9:**
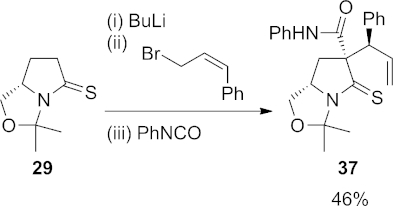
Three-component coupling reaction using (*Z*)-cinnamyl bromide.

Fortunately, deprotonation of thiolactam **29** at 0 °C followed by reaction with (*Z*)-cinnamyl bromide and then phenyl isocyanate afforded the desired three-component coupling product **37** in 46 % yield (Scheme 9).

Further investigations into this three-component coupling reaction and its application to the total synthesis of nakadomarin A are ongoing.

## Conclusions

We have demonstrated the feasibility of two key steps in a projected total synthesis of nakadomarin A. First, we have shown that the carbocyclic B ring in a tricyclic ABC substructure can be constructed by electrophilic substitution of a furan by using a thiolactam-derived acyliminium electrophile. Secondly, we have developed a novel three-component coupling reaction involving a thioamide, allylic bromide and isocyanate to stereoselectively install two vicinal stereogenic centres, one of them quaternary.

## Experimental Section

**General:** (Bromomethyl)furan (**9**),^12^ lactam **13**,^15^ Lawesson's reagent [2,4-bis(4-methoxyphenyl)-1,3,2,4-dithiadiphosphetane 2,4-disulfide]^28^ and hex-5-enoic acid^29^ were prepared by literature procedures. All other reagents were used as obtained from commercial sources. Solvents (THF, toluene, dichloromethane) were dried by passage through a column of activated alumina under nitrogen. All reactions in anhydrous solvents were carried out in flame-dried glassware under argon.

**1-Benzyl-3-(furan-3-ylmethyl)-3-methylpyrrolidin-2-one (10):** A stirred solution of 1-benzylpyrrolidin-2-one (**8**; 1.80 mL, 11.4 mmol) in THF (33 mL) at –78 °C was treated dropwise with *n*-butyllithium (2.5 M in hexanes, 5.50 mL, 13.7 mmol) and stirred for 30 min. The reaction mixture was treated dropwise with MeI (1.40 mL, 22.8 mmol) and stirred for 30 min. The reaction mixture was warmed to room temp. and stirred for 1.5 h and then quenched with saturated aq. NH_4_Cl (30 mL). The organic material was extracted with EtOAc (3 × 30 mL) and the combined organic extracts were washed with H_2_O (30 mL) and brine (30 mL), dried (MgSO_4_) and then concentrated in vacuo. Purification by flash chromatography (SiO_2_, 50 % EtOAc in petroleum ether) gave 1-benzyl-3-methylpyrrolidin-2-one (**8a**; 1.56 g, 72 %) as a yellow oil. IR (film): 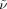
_max_ = 2965, 2930, 2872, 1677 cm^–1^. ^1^H NMR (600 MHz, CDCl_3_): *δ* = 1.24 (d, ^3^*J*_H,H_ = 7.2 Hz, 3 H, CH_3_), 1.59 (dq, ^2^*J*_H,H_ = 12.7, ^3^*J*_H,H_ = 8.4 Hz, 1 H, 4-H), 2.21 (dddd, ^2^*J*_H,H_ = 12.6, ^3^*J*_H,H_ = 8.6, 6.7, 4.1 Hz, 1 H, 4-H), 2.52 (ddt, ^3^*J*_H,H_ = 15.8, 8.7, 7.2 Hz, 1 H, 3-H), 3.15–3.19 (m, 2 H, 5-H), 4.43 (d, ^2^*J*_H,H_ = 14.7 Hz, 1 H, PhCH_2_), 4.47 (d, ^2^*J*_H,H_ = 14.7 Hz, 1 H, PhCH_2_), 7.20–7.36 (m, 5 H, Ar-H) ppm. ^13^C NMR (150 MHz, CDCl_3_): *δ* = 16.6 (CH_3_), 27.2 (C-4), 36.9 (C-3), 44.8 (C-5), 46.9 (PhCH_2_), 127.6 (CH), 128.2 (CH), 128.8 (CH), 136.8 (C), 177.5 (C-2) ppm. MS (EI): *m*/*z* (%) = 189 (63) [M]^+^, 92. HRMS (EI): calcd. for C_12_H_15_NO [M]^+^ 189.1148; found 189.1152.

A stirred solution of lactam **8a** (1.56 g, 8.23 mmol) in THF (28 mL) at –78 °C was treated dropwise with *n*-butyllithium (2.5 M in hexanes, 3.5 mL, 8.6 mmol) and stirred for 40 min. The reaction mixture was treated with a solution of bromide **9** (prepared from 6.43 mmol of furan-3-methanol and PBr_3_),^12^ stirred for 30 min, and then warmed to room temp. The reaction mixture was quenched with saturated aq. NH_4_Cl (20 mL) and the organic material extracted with EtOAc (3 × 20 mL). The combined organic extracts were washed with H_2_O (20 mL) and brine (20 mL), dried (MgSO_4_) and then concentrated in vacuo. Purification by flash chromatography (SiO_2_, 25 % EtOAc in petroleum ether) gave lactam **10** (480 mg, 28 % over two steps from furan-3-methanol) as a colourless oil. IR (CH_2_Cl_2_ cast): 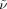
_max_ = 2961, 2930, 2871, 1682 cm^–1^. ^1^H NMR (600 MHz, CDCl_3_): *δ* = 1.21 (s, 3 H, CH_3_), 1.68 (ddd, ^2^*J*_H,H_ = 12.8, ^3^*J*_H,H_ = 8.3, 4.5 Hz, 1 H, 4-H), 1.99 (ddd, ^2^*J*_H,H_ = 12.8, ^3^*J*_H,H_ = 9.0, 6.4 Hz, 1 H, 4-H), 2.50 (d, ^2^*J*_H,H_ = 14.1 Hz, 1 H, FurCH_2_), 2.80 (d, ^2^*J*_H,H_ = 14.1 Hz, 1 H, FurCH_2_), 2.86 (ddd, ^2^*J*_H,H_ = 9.6, ^3^*J*_H,H_ = 9.0, 4.5 Hz, 1 H, 5-H), 3.06 (ddd, ^2^*J*_H,H_ = 9.7, ^3^*J*_H,H_ = 8.4, 6.4 Hz, 1 H, 5-H), 4.36 (d, ^2^*J*_H,H_ = 14.7 Hz, 1 H, PhCH_2_), 4.43 (d, ^2^*J*_H,H_ = 14.7 Hz, 1 H, PhCH_2_), 6.26 (d, ^4^*J*_H,H_ = 1.3 Hz, 1 H, Fur-H), 7.11–7.33 (m, 7 H, Ar-H) ppm. ^13^C NMR (150 MHz, CDCl_3_): *δ* = 24.0 (CH_3_), 30.2 (C-4), 33.2 (Fur-CH_2_), 43.4 (C-2), 45.1 (C-5), 46.9 (PhCH_2_), 112.3 (Fur-C-4), 120.7 (Fur-C-3), 127.6 (Ph-C-1), 128.1 (CH), 128.7 (CH), 136.6 (CH), 140.8 (Fur-C-5), 142.7 (Fur-C-2), 178.5 (C-2) ppm. MS (EI): *m*/*z* (%) = 269 (31) [M]^+^, 254 (13) [M – CH_3_]^+^, 187 (100) [M – CH_2_Fur]^+^, 158 (20), 91 (69) [PhCH_2_]^+^. HRMS (EI): calcd. for C_17_H_19_NO_2_ [M]^+^ 269.1410; found 269.1407.

**3-(Furan-3-ylmethyl)-3-methylpyrrolidine-2-thione (11):** A solution of lactam **10** (373 mg, 1.38 mmol) in THF (15 mL) was added to liquid NH_3_ (ca. 15 mL) at –78 °C. The stirred solution was treated with Na (65 mg, 2.8 mmol) and stirred for 10 min. The reaction mixture was quenched with satd. aq. NH_4_Cl (15 mL) and then warmed to room temp. overnight. The solvent was removed in vacuo and the organic material extracted with EtOAc (3 × 30 mL). The combined organic extracts were washed with H_2_O (30 mL) and brine (30 mL), dried (MgSO_4_) and then concentrated in vacuo to give 3-(furan-3-ylmethyl)-3-methylpyrrolidin-2-one (**10a**; 234 mg, 95 %) as a pale-yellow oil. IR (CDCl_3_ cast): 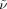
_max_ = 3230, 2964, 2927, 2872, 1692 cm^–1^. ^1^H NMR (600 MHz, CDCl_3_): *δ* = 1.17 (s, 3 H, CH_3_), 1.78 (ddd, ^2^*J*_H,H_ = 12.7, ^3^*J*_H,H_ = 8.0, 4.5 Hz, 1 H, 4-H), 2.09 (ddd, ^2^*J*_H,H_ = 12.7, ^3^*J*_H,H_ = 8.6, 6.6 Hz, 1 H, 4-H), 2.48 (d, ^2^*J*_H,H_ = 14.2 Hz, 1 H, FurCH_2_), 2.71 (d, ^2^*J*_H,H_ = 14.2 Hz, 1 H, FurCH_2_), 3.04 (td, ^2^*J*_H,H_ = 9.0, ^3^*J*_H,H_ = 9.0, 4.5 Hz, 1 H, 5-H), 3.22 (br. q, ^2^*J*_H,H_ = ^3^*J*_H,H_ = 8.0 Hz, 1 H, 5-H), 6.28 (s, 1 H, Fur-H), 6.79 (br. s, 1 H, NH), 7.24 (s, 1 H, Fur-H), 7.32 (s, 1 H, Fur-H) ppm. ^13^C NMR (150 MHz, CDCl_3_): *δ* = 23.5 (CH_3_), 32.7 (C-4), 32.8 (Fur-CH_2_), 39.0 (C-3), 44.0 (C-5), 112.2 (Fur-C-4), 120.7 (Fur-C-3), 140.7 (Fur-C-5), 142.8 (Fur-C-2), 182.8 (C-2) ppm. MS (EI): *m*/*z* (%) = 179 (41) [M]^+^, 164 (35) [M – CH_3_]^+^, 98 (35), 82 (100) [FurCH_2_]^+^. HRMS (EI): calcd. for C_10_H_13_NO_2_ [M]^+^ 179.0941; found 179.0944.

A stirred solution of lactam **10a** (234 mg, 1.31 mmol) in THF (5 mL) was treated with Lawesson's reagent (292 mg, 0.721 mmol). The reaction mixture was heated at 60 °C for 2 h and then concentrated in vacuo. Purification by flash chromatography (SiO_2_, 25 % EtOAc in petroleum ether) gave thiolactam **11** (204 mg, 80 %) as an orange oil. IR (CH_2_Cl_2_ cast): 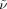
_max_ = 3166 (br), 2967, 2926, 2888, 1524 cm^–1^. ^1^H NMR (600 MHz, CDCl_3_): *δ* = 1.30 (s, 3 H, CH_3_), 1.95 (ddd, ^2^*J*_H,H_ = 12.8, ^3^*J*_H,H_ = 8.6, 5.7 Hz, 1 H, 4-H), 2.23 (ddd, ^2^*J*_H,H_ = 12.8, ^3^*J*_H,H_ = 8.7, 5.8 Hz, 1 H, 4-H), 2.67 (d, ^2^*J*_H,H_ = 14.3 Hz, 1 H, Fur-CH_2_), 2.88 (d, ^2^*J*_H,H_ = 14.3 Hz, 1 H, Fur-CH_2_), 3.16 (dddd, ^2^*J*_H,H_ = 11.0, ^3^*J*_H,H_ = 8.7, 5.7, 1.1 Hz, 1 H, 5-H), 3.40 (dddd, ^2^*J*_H,H_ = 11.0, ^3^*J*_H,H_ = 8.6, 5.9, 1.1 Hz, 1 H, 5-H), 6.37 (br. s, 1 H, Fur-H), 7.31 (m, 1 H, Fur-H), 7.33 (t, ^3^*J*_H,H_ = ^4^*J*_H,H_ = 1.7 Hz, 1 H, Fur-H), 7.67 (br. s, 1 H, NH) ppm. ^13^C NMR (150 MHz, CDCl_3_): *δ* = 26.5 (CH_3_), 33.5 (Fur-CH_2_), 35.3 (C-4), 45.6 (C-5), 54.1 (C-3), 112.1 (Fur-C-4), 120.4 (Fur-C-3), 140.7 (Fur-C-5), 142.6 (Fur-C-2), 213.0 (C-2) ppm. MS (EI): *m*/*z* (%) = 195 (100) [M]^+^, 180 (45) [M – CH_3_]^+^, 162 (16), 114 (22), 81 (60) [FurCH_2_]^+^. HRMS (EI): calcd. for C_10_H_13_NOS [M]^+^ 195.0712; found 195.0718.

**(8*RS*,14*RS*)-8-Methyl-3-oxa-14-thia-11-azatetracyclo[6.6.0.0^1,11^.0^2,6^]tetradeca-2(6),4-dien-12-one (12):** A stirred solution of thiolactam **11** (21 mg, 0.11 mmol) in toluene (2 mL) was treated with methyl bromoacetate (12 μL, 0.13 mmol). The reaction mixture was heated at 150 °C for 18 h and then concentrated in vacuo. Purification by flash chromatography (SiO_2_, 20 % EtOAc in petroleum ether) gave tetracycle **12** (18 mg, 73 %) as a yellow viscous oil. IR (CH_2_Cl_2_ thin film): 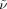
_max_ = 2960, 2922, 2864, 1683 cm^–1^. ^1^H NMR (600 MHz, CDCl_3_): *δ* = 1.34 (s, 3 H, CH_3_), 1.97 (dt, ^2^*J*_H,H_ = 13.0, ^3^*J*_H,H_ = 8.4 Hz, 1 H, 9-H), 2.08 (ddd, ^2^*J*_H,H_ = 12.8, ^3^*J*_H,H_ = 7.9, 4.1 Hz, 1 H, 9-H), 2.49 (d, ^2^*J*_H,H_ = 15.2 Hz, 1 H, 7-H), 2.65 (d, ^2^*J*_H,H_ = 15.2 Hz, 1 H, 7-H), 2.98 (dtd, ^2^*J*_H,H_ = 12.0, ^3^*J*_H,H_ = 8.1, 1.1 Hz, 1 H, 10-H), 3.55 (d, ^2^*J*_H,H_ = 15.2 Hz, 1 H, 13-H), 3.90 (ddd, ^2^*J*_H,H_ = 12.0, ^3^*J*_H,H_ = 8.3, 4.1 Hz, 1 H, 10-H), 4.17 (d, ^2^*J*_H,H_ = 15.2 Hz, 1 H, 13-H), 6.20 (d, ^3^*J*_H,H_ = 1.9 Hz, 1 H, 5-H), 7.44 (d, ^3^*J*_H,H_ = 1.9 Hz, 1 H, 4-H) ppm. ^13^C NMR (150 MHz, CDCl_3_): *δ* = 25.0 (CH_3_), 36.5 (C-13), 37.0 (C-7), 41.8 (C-9), 42.7 (C-10), 58.2 (C-8), 80.3 (C-1), 108.4 (C-5), 126.2 (C-6), 148.7 (C-4), 155.6 (C-2), 171.0 (C-12) ppm. MS (EI): *m*/*z* (%) = 235 (75) [M]^+^, 220 (43) [M – CH_3_]^+^, 175 (48), 162 (72) [M – SCH_2_CO]^+^, 146 (100). HRMS (EI): calcd. for C_12_H_13_NO_2_S [M]^+^ 235.0662; found 235.0664.

**(1*S*)-4-(4-Methoxyphenyl)-6-phenyl-4-thioxo-3-oxa-5-thia-7-aza-4-phosphabicyclo[5.3.0]decane-8-thione (14):** A stirred solution of lactam **13**^15^ (207 mg, 1.02 mmol) in THF (5 mL) was treated with Lawesson's reagent (247 mg, 0.611 mmol). The mixture was heated at reflux for 6 h and then concentrated in vacuo. Purification by flash chromatography (SiO_2_, 50 % EtOAc in petroleum ether) gave thiolactam **14** (340 mg, 79 %) as a colourless solid, m.p. 138–139 °C; [*α*]_D_^20^ = –74 (*c* = 1.0, CHCl_3_). IR (solid): 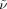
_max_ = 2924, 2838, 1591 cm^–1^. ^1^H NMR (600 MHz, CDCl_3_): *δ* = 2.02 (m, 1 H, 10-H), 2.40 (m, 1 H, 10-H), 3.12 (ddd, ^2^*J*_H,H_ = 18.1, ^3^*J*_H,H_ = 8.7, 2.6 Hz, 1 H, 9-H), 3.21 (ddd, ^2^*J*_H,H_ = 17.3, ^3^*J*_H,H_ = 10.5, 7.9 Hz, 1 H, 9-H), 3.89 (s, 3 H, OCH_3_), 4.04 (ddd, ^3^*J*_H,P_ = 28.2, ^2^*J*_H,H_ = 12.4, ^3^*J*_H,H_ = 5.7 Hz, 1 H, 2-H), 4.94 (m, 1 H, 1-H), 5.09 (ddd, ^3^*J*_H,P_ = 15.2, ^2^*J*_H,H_ = 12.1, ^3^*J*_H,H_ = 6.2 Hz, 1 H, 2-H), 6.99–7.07 (m, 3 H, 6-H, Ar-H), 7.28–7.39 (m, 5 H, Ar-H), 7.93 (dd, ^3^*J*_H,P_ = 14.4, ^3^*J*_H,H_ = 8.8 Hz, 2 H, Ar-H) ppm. ^13^C NMR (150 MHz, CDCl_3_): *δ* = 28.0 (C-10), 43.7 (C-9), 55.7 (OCH_3_), 62.5 (C-1), 64.8 (d, ^3^*J*_C,P_ = 4.2 Hz, C-6), 65.6 (d, ^3^*J*_C,P_ = 6.6 Hz, C-2), 114.5 (d, ^2^*J*_C,P_ = 16.1 Hz, *C*HCP), 124.7 (d, ^1^*J*_C,P_ = 129.9 Hz, CP), 127.3 (CH), 129.11 (CH), 129.15 (CH), 133.5 (d, ^3^*J*_C,P_ = 14.3 Hz, *C*HCHCP), 135.4 (d, ^3^*J*_C,P_ = 6.6 Hz, Ph-C-1), 163.6 (d, ^4^*J*_C,P_ = 3.6 Hz, MeO*C*), 203.3 (C-8) ppm. MS (CI): *m*/*z* (%) = 422 (44) [MH]^+^, 300 (100) [MH – SCHPh]^+^. HRMS (CI): calcd. for C_20_H_22_NO_2_PS_3_ [MH]^+^ 422.0472; found 422.0474.

**Ethyl (2*R*,5*S*,7*R*)-7-(Furan-3-ylmethyl)-8-oxo-2-phenyl-3-oxa-1-azabicyclo[3.3.0]octane-7-carboxylate (*epi*-15) and Ethyl (2*R*,5*S*,7*S*)-7-(Furan-3-ylmethyl)-8-oxo-2-phenyl-3-oxa-1-azabicyclo[3.3.0]octane-7-carboxylate (15):** Compound **13** was acylated following the procedure of Moloney and co-workers.17b A flask containing a stirred solution of lactam **13** (8.7 g, 43 mmol) and diethyl carbonate (21 mL, 170 mmol) in toluene (70 mL) was fitted with a Dean–Stark trap. The solution was heated at reflux for 4.5 h, cooled to 0 °C and then added to a pre-washed NaH (60 % dispersion in mineral oil, 4.12 g, 100 mmol). The reaction mixture was heated at reflux for 14 h and then cooled to 0 °C. It was then treated with AcOH (4.40 g, 73.3 mmol) and stirred for 1 h at room temp. The mixture was filtered and the filtrate concentrated in vacuo. Purification by flash chromatography (SiO_2_, 30–50 % EtOAc in petroleum ether, gradient elution) gave ethyl (2*R*,5*S*,7*RS*)-8-oxo-2-phenyl-3-oxa-1-azabicyclo[3.3.0]octane-7-carboxylate (**13a**) as a 13:1 mixture of diastereoisomers (7.3 g, 62 %) as a yellow solid, m.p. 66–67 °C (ref.17b 85–87 °C for a 1:1 ratio of diastereoisomers). IR (solid): 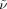
_max_ = 2985, 2917, 2875, 1736, 1687 cm^–1^. ^1^H NMR (600 MHz, CDCl_3_, data for major isomer): *δ* = 1.33 (t, ^3^*J*_H,H_ = 7.2 Hz, 3 H, CH_3_), 2.42 (ddd, ^2^*J*_H,H_ = 13.3, ^3^*J*_H,H_ = 9.8, 6.2 Hz, 1 H, 6-H), 2.57 (ddd, ^2^*J*_H,H_ = 13.3, ^3^*J*_H,H_ = 9.4, 7.5 Hz, 1 H, 6-H), 3.69 (t, ^2^*J*_H,H_ = ^3^*J*_H,H_ = 8.2 Hz, 1 H, 4-H), 3.87 (t, ^3^*J*_H,H_ = 9.6 Hz, 1 H, 7-H), 4.13 (tt, ^3^*J*_H,H_ = 7.5, 6.5 Hz, 1 H, 5-H), 4.20–4.34 (m, 3 H, 4-H, C*H*_2_CH_3_), 6.32 (s, 1 H, 2-H), 7.30–7.48 (m, 5 H, Ar-H) ppm. ^13^C NMR (150 MHz, CDCl_3_, data for major isomer): *δ* = 14.3 (CH_3_), 27.6 (C-6), 51.7 (C-7), 57.0 (C-5), 62.1 (*C*H_2_CH_3_), 72.0 (C-4), 87.2 (C-2), 126.0 (CH), 128.6 (CH), 128.8 (CH), 138.4 (C), 169.3 (C-8), 172.3 (*C*O_2_Et) ppm.

NaH (60 % dispersion in oil, 620 mg, 15.3 mmol) was washed with hexanes and then suspended in THF (80 mL) and cooled to 0 °C. The suspension was treated with lactam **13a** (13:1 mixture of diastereoisomers, 3.52 g, 12.8 mmol) in THF (20 mL), then warmed to room temp. and stirred for 30 min. The reaction mixture was treated with a solution of bromide **9** (prepared from 16.8 mmol of furan-3-methanol and PBr_3_)^12^ and stirred for 21 h. The reaction mixture was quenched with saturated aq. NH_4_Cl (120 mL) and the organic material extracted with EtOAc (3 × 75 mL). The combined organic extracts were washed with water (50 mL) and brine (50 mL), dried (MgSO_4_) and then concentrated in vacuo. Purification by flash chromatography (SiO_2_, 20 % Et_2_O in petroleum ether) gave lactam *epi*-**15** (312 mg, 7 %) as a colourless solid, m.p. 50–52 °C; [*α*]_D_^20^ = +165 (*c* 0.99, CHCl_3_). IR (CHCl_3_): 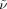
_max_ = 2981, 2938, 2876, 1736, 1708 cm^–1^. ^1^H NMR (600 MHz, CDCl_3_): *δ* = 1.27 (t, ^3^*J*_H,H_ = 7.2 Hz, 3 H, CH_3_), 1.87 (dd, ^2^*J*_H,H_ = 13.2, ^3^*J*_H,H_ = 6.8 Hz, 1 H, 6-H), 2.78 (dd, ^2^*J*_H,H_ = 13.2, ^3^*J*_H,H_ = 7.2 Hz, 1 H, 6-H), 3.09 (d, ^2^*J*_H,H_ = 14.8 Hz, 1 H, Fur-CH_2_), 3.14 (d, ^2^*J*_H,H_ = 14.8 Hz, 1 H, Fur-CH_2_), 3.18 (t, ^2^*J*_H,H_ = ^3^*J*_H,H_ = 7.9 Hz, 1 H, 4-H), 4.14 (dd, ^2^*J*_H,H_ = 8.0, ^3^*J*_H,H_ = 6.3 Hz, 1 H, 4-H), 4.17–4.28 (m, 3 H, 4-H, C*H*_2_CH_3_), 6.25–6.28 (m, 2 H, 2-H, Fur-H), 7.27–7.43 (m, 7 H, Ar-H) ppm. ^13^C NMR (150 MHz, CDCl_3_): *δ* = 14.2 (CH_3_), 29.2 (Fur-CH_2_), 33.8 (C-6), 56.3 (C-5), 61.6 (C-7), 62.2 (*C*H_2_CH_3_), 72.1 (C-4), 87.0 (C-2), 111.9 (Fur-C-4), 119.6 (Fur-C-3), 126.1 (CH), 128.6 (CH), 128.8 (CH), 138.1 (C), 141.2 (Fur-C-5), 143.3 (Fur-C-2), 170.7 (C-10), 173.0 (*C*O_2_Et) ppm. MS (CI): *m*/*z* (%) = 356 (100) [MH]^+^. HRMS (CI): calcd. for C_20_H_22_NO_5_ [MH]^+^ 356.1498; found 356.1504.

Further elution (20 % Et_2_O in petroleum ether) afforded lactam **15** (1.86 g, 41 %, major isomer) as a colourless oil. [*α*]_D_^20^ = +59 (*c* 0.81, CHCl_3_). IR (CHCl_3_): 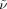
_max_ = 2981, 2939, 2878, 1740, 1704 cm^–1^. ^1^H NMR (600 MHz, CDCl_3_): *δ* = 1.32 (t, ^3^*J*_H,H_ = 7.2 Hz, 3 H, CH_3_), 2.32 (dd, ^2^*J*_H,H_ = 13.9, ^3^*J*_H,H_ = 7.9 Hz, 1 H, C6-H), 2.50 (dd, ^2^*J*_H,H_ = 13.9, ^3^*J*_H,H_ = 4.9 Hz, 1 H, 6-H), 3.07 (d, ^2^*J*_H,H_ = 14.6 Hz, 1 H, Fur-CH_2_), 3.12 (d, ^2^*J*_H,H_ = 14.6 Hz, 1 H, Fur-CH_2_), 3.62 (dd, ^3^*J*_H,H_ = 8.7, ^2^*J*_H,H_ = 7.9 Hz, 1 H, 4-H), 3.74 (tdd, ^3^*J*_H,H_ = 8.5, 6.1, 4.6 Hz, 1 H, 5-H), 4.19 (dd, ^2^*J*_H,H_ = 7.7, ^3^*J*_H,H_ = 6.2 Hz, 1 H, 4-H), 4.22–4.32 (m, 2 H, C*H*_2_CH_3_), 6.23 (t, ^3^*J*_H,H_ = ^4^*J*_H,H_ = 1.3 Hz, 1 H, Fur-H), 6.29 (s, 1 H, 2-H), 7.27–7.38 (m, 7 H, Ar-H) ppm. ^13^C NMR (150 MHz, CDCl_3_): *δ* = 14.2 (CH_3_), 29.8 (Fur-CH_2_), 31.1 (C-6), 56.3 (C-5), 60.7 (C-7), 62.2 (*C*H_2_CH_3_), 71.7 (C-4), 87.3 (C-2), 112.0 (Fur-C-4), 119.2 (Fur-C-3), 126.0 (CH), 128.5 (CH), 128.8 (CH), 138.4 (C), 141.2 (Fur-C-5), 143.1 (Fur-C-2), 171.2 (C-10), 174.9 (*C*O_2_Et) ppm. MS (CI): *m*/*z* (%) = 356 (100) [MH]^+^. HRMS (CI): calcd. for C_20_H_22_NO_5_ [MH]^+^ 356.1498; found 356.1507.

**Ethyl (3*R*,5*S*)-5-Acetoxymethyl-3-(furan-3-ylmethyl)-2-thioxopyrrolidine-3-carboxylate (16):** A stirred solution of lactam **15** (557 mg, 1.57 mmol) in CH_2_Cl_2_ (42 mL) was treated with *p*-toluenesulfonic acid monohydrate (298 mg, 1.57 mmol) and stirred for 105 min. The reaction mixture was passed through a short pad of silica gel, eluting with EtOAc to give ethyl (3*R*,5*S*)-3-(furan-3-ylmethyl)-5-hydroxymethyl-2-oxopyrrolidine-3-carboxylate (**15a**, 371 mg, 87 %) as a colourless solid, m.p. 82–83 °C; [*α*]_D_^20^ = +6.5 (*c* = 0.31, CHCl_3_). IR (solid): 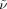
_max_ = 3227 (br), 2930, 1721, 1676 cm^–1^. ^1^H NMR (600 MHz, CDCl_3_): *δ* = 1.29 (t, ^3^*J*_H,H_ = 7.2 Hz, 3 H, CH_3_), 2.24 (dd, ^2^*J*_H,H_ = 13.8, ^3^*J*_H,H_ = 8.7 Hz, 1 H, 4-H), 2.29 (dd, ^2^*J*_H,H_ = 13.8, ^3^*J*_H,H_ = 5.2 Hz, 1 H, 4-H), 2.43 (br. t, ^3^*J*_H,H_ = 5.5 Hz, 1 H, OH), 3.02 (d, ^2^*J*_H,H_ = 14.4 Hz, 1 H, Fur-CH_2_), 3.08 (d, ^2^*J*_H,H_ = 14.4 Hz, 1 H, Fur-CH_2_), 3.40 (br. m, 1 H, C-5), 3.52 (ddd, ^2^*J*_H,H_ = 11.1, ^3^*J*_H,H_ = 7.0, 6.0 Hz, 1 H, C*H*_2_OH), 3.64 (ddd, ^2^*J*_H,H_ = 10.9, ^3^*J*_H,H_ = 5.3, 3.8 Hz, 1 H, C*H*_2_OH), 4.19–4.27 (m, 2 H, C*H*_2_CH_3_), 6.30 (d, ^3^*J*_H,H_ = 1.3 Hz, 1 H, Fur-H), 6.49 (br. s, 1 H, NH), 7.30 (s, 1 H, Fur-H), 7.35 (t, ^3^*J*_H,H_ = ^4^*J*_H,H_ = 1.6 Hz, 1 H, Fur-H) ppm. ^13^C NMR (150 MHz, CDCl_3_): *δ* = 14.2 (CH_3_), 29.6 (Fur-CH_2_), 31.3 (C-4), 53.1 (C-5), 55.9 (C-3), 62.2 (*C*H_2_CH_3_), 66.0 (*C*H_2_OH), 111.9 (Fur-C-4), 119.2 (Fur-C-3), 141.2 (Fur-C-5), 143.3 (Fur-C-2), 172.1 (C-2), 175.2 (*C*O_2_Et) ppm. MS (ES^+^): *m*/*z* (%) = 290 (100) [MNa]^+^, 222 (50) [MNa – C_4_H_3_O]^+^. HRMS (ES^+^): calcd. for C_13_H_17_NO_5_Na [MNa]^+^ 290.1004; found 290.1012.

A stirred solution of lactam **15a** (269 mg, 1.01 mmol) in CH_2_Cl_2_ (10 mL) was treated with Ac_2_O (105 μL, 1.1 mmol) and DMAP (24 mg, 0.2 mmol). The reaction mixture was stirred for 80 min and then concentrated in vacuo. Purification by flash chromatography (SiO_2_, 30–50 % EtOAc in petroleum ether) gave ethyl (3*R*,5*S*)-5-acetoxymethyl-3-(furan-3-ylmethyl)-2-oxopyrrolidine-3-carboxylate (**15b**, 298 mg, 96 %) as a colourless oil. [*α*]_D_^20^ = –3.0 (*c* = 1.0, CHCl_3_). IR (film): 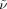
_max_ = 3114 (br), 2940, 2912, 2907, 1734, 1697 cm^–1^. ^1^H NMR (600 MHz, CDCl_3_): *δ* = 1.30 (t, ^3^*J*_H,H_ = 7.2 Hz, 3 H, CH_2_C*H*_3_), 2.06 (s, 3 H, CH_3_CO), 2.28 (dd, ^2^*J*_H,H_ = 13.9, ^3^*J*_H,H_ = 8.4 Hz, 1 H, 4-H), 2.32 (dd, ^2^*J*_H,H_ = 13.9, ^3^*J*_H,H_ = 5.5 Hz, 1 H, 4-H), 3.02 (d, ^2^*J*_H,H_ = 14.5 Hz, 1 H, Fur-CH_2_), 3.09 (d, ^2^*J*_H,H_ = 14.5 Hz, 1 H, Fur-CH_2_), 3.47 (m, 1 H, 5-H), 3.93 (dd, ^2^*J*_H,H_ = 11.2, ^3^*J*_H,H_ = 8.5 Hz, 1 H, AcOCH_2_), 4.16 (dd, ^2^*J*_H,H_ = 11.2, ^3^*J*_H,H_ = 4.0 Hz, 1 H, AcOCH_2_), 4.24 (q, ^3^*J*_H,H_ = 7.2 Hz, 2 H, C*H*_2_CH_3_), 6.06 (br. s, 1 H, NH), 6.30 (br. s, 1 H, Fur-H), 7.30 (s, 1 H, Fur-H), 7.35 (t, ^3^*J*_H,H_ = ^4^*J*_H,H_ = 1.7 Hz, 1 H, Fur-H) ppm. ^13^C NMR (150 MHz, CDCl_3_): *δ* = 14.2 (CH_2_*C*H_3_), 20.9 (*C*H_3_CO), 29.5 (Fur-CH_2_), 31.5 (C-4), 50.0 (C-5), 55.4 (C-3), 62.2 (*C*H_2_CH_3_), 67.3 (AcOCH_2_), 111.9 (Fur-C-4), 119.1 (Fur-C-3), 141.3 (Fur-C-5), 143.3 (Fur-C-2), 170.7 (CH_3_*C*=O), 171.3 (*C*O_2_Et), 174.6 (C-2) ppm. MS (CI): *m*/*z* (%) = 310 (42) [MH]^+^, 236 (90) [MH – CO_2_Et]^+^, 222 (19), 203 (47), 190 (43), 81 (100) [FurCH_2_]^+^. HRMS (CI): calcd. for C_15_H_20_NO_6_ [MH]^+^ 310.1291; found 310.1289.

A stirred solution of lactam **15b** (285 mg, 0.92 mmol) in THF (5 mL) was treated with Lawesson's reagent (206 mg, 0.51 mmol) and heated at reflux for 5.5 h. The reaction mixture was treated with further Lawesson's reagent (21 mg, 0.051 mmol) and heated at reflux for 2 h and then concentrated in vacuo. Purification by flash chromatography (SiO_2_, 25–50 % Et_2_O in petroleum ether) gave thiolactam **16** (197 mg, 65 %) as a colourless oil. [*α*]_D_^20^ = –0.8 (*c* = 1.25, CHCl_3_). IR (film): 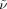
_max_ = 3142 (br), 2982, 2932, 2906, 1733, 1515 cm^–1^. ^1^H NMR (600 MHz, CDCl_3_): *δ* = 1.30 (t, ^3^*J*_H,H_ = 7.2 Hz, 3 H, CH_2_C*H*_3_), 2.08 (s, 3 H, CH_3_CO), 2.35–2.42 (m, 2 H, 4-H), 3.11 (d, ^2^*J*_H,H_ = 14.5 Hz, 1 H, Fur-CH_2_), 3.26 (d, ^2^*J*_H,H_ = 14.5 Hz, 1 H, Fur-CH_2_), 3.64 (m, 1 H, 5-H), 3.98 (dd, ^2^*J*_H,H_ = 11.2, ^3^*J*_H,H_ = 8.9 Hz, 1 H, AcOCH_2_), 4.18–4.28 (m, 3 H, C*H*_2_CH_3_, AcOCH_2_), 6.38 (br. s, 1 H, Fur-H), 7.26 (s, 1 H, Fur-H), 7.34 (s, 1 H, Fur-H), 8.04 (br. s, 1 H, NH) ppm. ^13^C NMR (150 MHz, CDCl_3_): *δ* = 14.1 (CH_2_*C*H_3_), 20.9 (*C*H_3_CO), 32.1 (Fur-CH_2_), 33.2 (C-4), 58.4 (C-5), 62.3 (*C*H_2_CH_3_), 65.2 (C-3), 66.3 (AcOCH_2_), 112.0 (Fur-C-4), 119.1 (Fur-C-3), 141.4 (Fur-C-5), 143.2 (Fur-C-2), 170.6 (CH_3_*C*O), 171.0 (*C*O_2_Et), 205.1 (CS) ppm. MS (EI): *m*/*z* (%) = 325 (48) [M]^+^, 252 (100) [M – CO_2_Et]^+^, 210 (33) [M – CH_3_CO]^+^. HRMS (EI): calcd. for C_15_H_19_NO_5_S [M]^+^ 325.0979; found 325.0973.

**3-Bromotetrahydro-2*H*-pyran-2-one (17):** A stirred solution of diisopropylamine (1.7 mL, 12 mmol) in THF (10 mL) was cooled to 0 °C and treated dropwise with *n*-butyllithium (2.5 M in hexanes, 4.4 mL, 11 mmol), stirred for 15 min and then cooled to –78 °C. The solution was treated with *δ*-valerolactone (0.93 mL, 10 mmol) in THF (1 mL) and stirred for 15 min. The reaction mixture was treated with chlorotrimethylsilane (1.7 mL, 13 mmol), stirred for 1 h and then warmed to room temp. The mixture was concentrated in vacuo and diluted with pentane (10 mL), filtered and then concentrated in vacuo once more. Purification by distillation (31–32 °C, 4 Torr) gave 6-trimethylsilyloxy-3,4-dihydro-2*H*-pyran^30^ (**17a**; 1.32 g, 77 %) as a colourless oil. IR (CDCl_3_ cast): 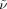
_max_ = 2956, 2899, 2878 cm^–1^. ^1^H NMR (600 MHz, CDCl_3_): *δ* = 0.21 [s, 9 H, Si(CH_3_)_3_], 1.74 (m, 2 H, 3-H), 2.03 (td, ^3^*J*_H,H_ = 6.4, 3.6 Hz, 2 H, 4-H), 3.81 (t, ^3^*J*_H,H_ = 3.6 Hz, 1 H, 5-H), 4.04 (br. dd, ^3^*J*_H,H_ = 6.0, 4.3 Hz, 2 H, 2-H) ppm. ^13^C NMR (150 MHz, CDCl_3_): *δ* = 0.1 [Si(CH_3_)_3_], 20.0 (C-4), 22.5 (C-3), 67.3 (C-2), 74.1 (C-5), 154.6 (C-6) ppm.

A stirred solution of silyl enol ether **17a** (44 mg, 0.26 mmol) in CH_2_Cl_2_ (1 mL) was cooled to –15 °C and treated dropwise with Br_2_ (13 μL, 0.26 mmol). The solution was stirred for 30 min and then concentrated in vacuo. Purification by flash chromatography (SiO_2_, 30 % Et_2_O in petroleum ether) gave lactone **17**^31^ (45 mg, 98 %) as a colourless oil. IR (CDCl_3_ cast): 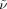
_max_ = 2944, 2876, 2861, 1733 cm^–1^. ^1^H NMR (600 MHz, CDCl_3_): *δ* = 1.90 (ddt, ^2^*J*_H,H_ = 14.6, ^3^*J*_H,H_ = 10.5, 5.2 Hz, 1 H, 5-H), 2.25 (tdd, ^2^*J*_H,H_ = 14.2, ^3^*J*_H,H_ = 9.4, 5.1 Hz, 1 H, 5-H), 2.35 (m, 1 H, 4-H), 2.46 (ddt, ^2^*J*_H,H_ = 14.8, ^3^*J*_H,H_ = 9.5, 5.2 Hz, 1 H, 4-H), 4.40 (ddd, ^3^*J*_H,H_ = 11.4, 8.9, 4.5 Hz, 1 H, 3-H), 4.56–4.63 (m, 2 H, 6-H) ppm. ^13^C NMR (150 MHz, CDCl_3_): *δ* = 20.0 (C-5), 30.4 (C-4), 40.8 (C-3), 70.0 (C-6), 166.9 (C-2) ppm. MS (CI): *m*/*z* (%) = 181/179 (78/81) [MH]^+^, 101 (100), 99 (100) [MH – HBr]^+^. HRMS (CI): calcd. for C_5_H_8_^79^BrO_2_ [MH]^+^ 178.9708; found 178.9702.

**2-Bromohex-5-enoyl Chloride (18):** A stirred solution of diisopropylamine (1.80 mL, 12.8 mmol) in THF (22 mL) was cooled to 0 °C and then treated dropwise with *n*-butyllithium (2.5 M in hexanes, 5.10 mL, 12.8 mmol). The reaction mixture was stirred for 45 min, then cooled to –10 °C and treated dropwise with hex-5-enoic acid^30^ (666 mg, 5.83 mmol) in THF (11 mL) and stirred for 2 h. The reaction mixture was cooled to –78 °C and treated with CBr_4_ (3.90 g, 11.7 mmol) in THF (1 mL) and then warmed to room temp. over 1.5 h. The reaction mixture was diluted with brine (30 mL) and 2 M HCl (30 mL) and then extracted with Et_2_O (3 × 40 mL). The combined organic extracts were dried (MgSO_4_) and then concentrated in vacuo. Purification by flash chromatography (SiO_2_, 30 % Et_2_O in petroleum ether) gave an oil which was subsequently distilled under reduced pressure (1.3 Torr, 120 °C) to give 2-bromohex-5-enoic acid (**18a**, 517 mg, 46 %) as a yellow oil. IR (CDCl_3_ cast): 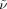
_max_ = 3081 (br), 2934, 2881, 2850, 1715 cm^–1^. ^1^H NMR (600 MHz, CDCl_3_): *δ* = 2.03–2.33 (m, 4 H, 3-H, 4-H), 4.27 (dd, ^3^*J*_H,H_ = 8.1, 6.2 Hz, 1 H, 2-H), 5.06 (d, ^3^*J*_H,H_ = 10.5 Hz, 1 H, 6-H), 5.11 (dd, ^3^*J*_H,H_ = 16.9, ^2^*J*_H,H_ = 1.5 Hz, 1 H, 6-H), 5.76 (ddt, ^3^*J*_H,H_ = 17.1, 10.4, 6.6 Hz, 1 H, 5-H) ppm. ^13^C NMR (150 MHz, CDCl_3_): *δ* = 31.2 (C-4), 33.7 (C-3), 44.6 (C-2), 116.8 (C-6), 135.9 (C-5), 174.2 (C-1) ppm. MS (CI): *m*/*z* (%) = 195/193 (100) [MH]^+^, 177/175 (100) [MH – H_2_O]^+^, 113 (97) [MH – HBr]^+^. HRMS (CI): calcd. for C_6_H_10_^79^BrO_2_ [MH]^+^ 192.9864; found 192.9869.

A stirred solution of acid **18a** (155 mg, 0.80 mmol) in CH_2_Cl_2_ (3 mL) was treated with oxalyl chloride (0.202 mL, 2.41 mmol) and DMF (1 drop) and then stirred for 1.5 h. The reaction mixture was concentrated in vacuo to give acyl chloride **18** (contaminated with DMF, 115 mg, <68 %) as an oil. IR (film): 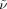
_max_ = 2933, 2885, 2863, 1718, 1642 cm^–1^. ^1^H NMR (600 MHz, CDCl_3_): *δ* = 2.14 (m, 1 H, 3-H), 2.20–2.33 (m, 3 H, 3-H, 4-H), 4.52 (dd, ^3^*J*_H,H_ = 8.1, 5.5 Hz, 1 H, 2-H), 5.10 (d, ^3^*J*_H,H_ = 10.4 Hz, 1 H, 6-H), 5.13 (dd, ^3^*J*_H,H_ = 16.9, ^2^*J*_H,H_ = 1.1 Hz, 1 H, 6-H), 5.75 (ddt, ^3^*J*_H,H_ = 16.9, 10.4, 6.4 Hz, 1 H, 5-H) ppm. ^13^C NMR (150 MHz, CDCl_3_): *δ* = 30.9 (C-4), 33.8 (C-3), 53.6 (C-2), 117.5 (C-6), 135.3 (C-5), 170.3 (C-1) ppm. MS (CI): *m*/*z* (%) = 209/207 (82), 177/175 (15) [MH – HCl]^+^, 163 (48), 127 (100).

**Ethyl (2*S*,4*R*)-2-Acetoxymethyl-4-(furan-3-ylmethyl)-5-(2-oxooxan-3-ylsulfanyl)-3,4-dihydro-2*H*-pyrrole-4-carboxylate (19):** NaH (60 % dispersion in oil, 58 mg, 1.45 mmol) was washed with hexanes and then suspended in THF (7 mL), cooled to 0 °C and treated dropwise with a solution of thiolactam **16** (394 mg, 1.21 mmol) in THF (2 mL) and then stirred for 30 min. The reaction mixture was treated dropwise with a solution of lactone **17** (234 mg, 1.31 mmol) in THF (1 mL) and then warmed to room temp. The reaction mixture was stirred for 1.5 h and then diluted with H_2_O (12 mL). The organic material was extracted with EtOAc (4 × 15 mL) and the organic extracts were combined, washed with brine (2 × 15 mL), dried (MgSO_4_) and then concentrated in vacuo to give thioimidate **19** (6:4 mixture of diastereomers, 340 mg, 66 %) as a brown oil which was used in the next step without further purification. IR (CDCl_3_ cast): 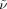
_max_ = 2924, 2872, 2854, 1729 cm^–1^. ^1^H NMR (600 MHz, CDCl_3_): *δ* = 1.30 (t, ^3^*J*_H,H_ = 7.1 Hz, 3 × 0.6 H, CH_2_C*H*_3_^maj^), 1.31 (t, ^3^*J*_H,H_ = 7.2 Hz, 3 × 0.4 H, CH_2_C*H*_3_^min^), 1.88–2.00 (m, 2 H, C*H*_2_CH_2_O), 2.04 (s, 3 × 0.6 H, CH_3_CO^maj^), 2.05 (s, 3 × 0.4 H, CH_3_CO^min^), 2.16 (dd, ^2^*J*_H,H_ = 13.3, ^3^*J*_H,H_ = 8.4 Hz, 0.6 H, 3-H^maj^), 2.20 (dd, ^2^*J*_H,H_ = 13.4, ^3^*J*_H,H_ = 8.0 Hz, 0.4 H, 3-H^min^), 2.24–2.35 (m, 3 H, 3-H, C*H*_2_CHS), 2.91 (d, ^2^*J*_H,H_ = 14.8 Hz, 0.6 H, Fur-CH_2_^maj^), 2.92 (d, ^2^*J*_H,H_ = 14.8 Hz, 0.4 H, Fur-CH_2_^min^), 3.07 (d, ^2^*J*_H,H_ = 14.8 Hz, 0.6 H, Fur-CH_2_^maj^), 3.08 (d, ^2^*J*_H,H_ = 14.8 Hz, 0.4 H, Fur-CH_2_^min^), 3.87–3.93 (m, 1.4 H, 2-H, CHS^min^), 4.07 (dd, ^2^*J*_H,H_ = 11.2, ^3^*J*_H,H_ = 6.2 Hz, 0.6 H, AcOCH_2_^maj^), 4.13 (dd, ^2^*J*_H,H_ = 11.0, ^3^*J*_H,H_ = 6.2 Hz, 0.4 H, AcOCH_2_^min^), 4.18–4.28 (m, 3 H, AcOCH_2_, C*H*_2_CH_3_), 4.37–4.47 (m, 2 H, CHS^maj^, CH_2_O), 4.53 (dt, ^2^*J*_H,H_ = 10.8, ^3^*J*_H,H_ = 4.3 Hz, 0.6 H, CH_2_O), 6.34 (d, ^3^*J*_H,H_ = 1.8 Hz, 0.4 H, Fur-H^min^), 6.35 (d, ^3^*J*_H,H_ = 1.8 Hz, 0.6 H, Fur-H^maj^), 7.30 (s, 0.6 H, Fur-H^maj^), 7.31 (s, 0.4 H, Fur-H^min^), 7.33 (s, 0.6 H, Fur-H^maj^), 7.34 (s, 0.4 H, Fur-H^min^) ppm. ^13^C NMR (150 MHz, CDCl_3_): *δ* = 14.1 (CH_2_*C*H_3_^min^), 14.2 (CH_2_*C*H_3_^maj^), 21.03 (*C*H_3_CO^maj^), 21.04 (*C*H_3_CO^min^), 23.0 (*C*H_2_CH_2_O^min^), 24.0 (*C*H_2_CH_2_O^maj^), 27.2 (*C*H_2_CHS^min^), 27.8 (*C*H_2_CHS^maj^), 30.5 (Fur-CH_2_^maj^), 30.7 (Fur-CH_2_^min^), 35.7 (C-3^maj^), 35.9 (C-3^min^), 43.9 (CHS^maj^), 44.1 (CHS^min^), 62.05 (*C*H_2_CH_3_^maj^), 62.13 (*C*H_2_CH_3_^min^), 66.3 (C-4^maj^), 66.58 (C-4^min^), 66.60 (AcOCH_2_^maj^), 66.7 (AcOCH_2_^min^), 69.1 (CH_2_O^min^), 69.5 (CH_2_O^maj^), 69.8 (C-2^min^), 70.0 (C-2^maj^), 111.7 (Fur-C-4^maj^), 111.8 (Fur-C-4^min^), 118.9 (Fur-C-3^maj^), 119.0 (Fur-C-3^min^), 141.2 (Fur-C-2^min^), 141.4 (Fur-C-2^maj^), 143.1 (Fur-C-5^min^), 143.3 (Fur-C-5^maj^), 168.9 (SCH*C*O^maj^), 169.3 (SCH*C*O^min^), 170.2 (*C*O_2_Et^maj^), 170.6 (*C*O_2_Et^min^), 171.0 (CH_3_*C*O^maj^), 171.1 (CH_3_*C*O^min^), 171.3 (C-5^maj^), 171.4 (C-5^min^) ppm. MS (CI): *m*/*z* (%) = 424 (100) [MH]^+^. HRMS (CI): calcd. for C_20_H_26_NO_7_S [MH]^+^ 424.1430; found 424.1433.

**Ethyl (2*E*,3*S*,5*S*)-5-Acetoxymethyl-3-(furan-3-ylmethyl)-2-(2-oxooxan-3-ylidene)pyrrolidine-3-carboxylate (20):** A stirred solution of thioimidate **19** (31 mg, 0.073 mmol) in toluene (2 mL) was heated at 160 °C in a sealed tube for 18 h. The reaction mixture was concentrated in vacuo. Purification by flash chromatography (SiO_2_, 70 % Et_2_O in petroleum ether) gave vinylogous carbamate **20** (23 mg, 81 %) as a yellow viscous oil. [*α*]_D_^20^ = +269 (*c* = 0.7, CHCl_3_). IR (film): 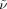
_max_ = 3364 (br), 2923, 2854, 1728 cm^–1^. ^1^H NMR (600 MHz, CDCl_3_): *δ* = 1.29 (t, ^3^*J*_H,H_ = 7.2 Hz, 3 H, CH_2_C*H*_3_), 1.77–1.89 (m, 2 H, C*H*_2_CH_2_O), 2.00 (dd, ^2^*J*_H,H_ = 13.2, ^3^*J*_H,H_ = 7.9 Hz, 1 H, 4-H), 2.06 (s, 3 H, CH_3_CO), 2.13–2.21 (m, 2 H, 4-H, C=C-CH_2_), 2.39 (m, 1 H, C=C-CH_2_), 3.03 (d, ^2^*J*_H,H_ = 14.9 Hz, 1 H, Fur-CH_2_), 3.07 (d, ^2^*J*_H,H_ = 14.9 Hz, 1 H, Fur-CH_2_), 3.46 (qd, ^3^*J*_H,H_ = 7.8, 3.8 Hz, 1 H, 5-H), 3.82 (dd, ^2^*J*_H,H_ = 11.1, ^3^*J*_H,H_ = 7.9 Hz, 1 H, AcOCH_2_), 4.09 (dd, ^2^*J*_H,H_ = 11.1, ^3^*J*_H,H_ = 4.1 Hz, 1 H, AcOCH_2_), 4.20–4.30 (m, 4 H, C*H*_2_CH_3_, CH_2_C*H*_2_O), 6.21 (br. s, 1 H, Fur-H), 7.28 (s, 1 H, Fur-H), 7.33 (t, ^2^*J*_H,H_ = ^3^*J*_H,H_ = 1.5 Hz, 1 H, Fur-H), 9.43 (s, 1 H, NH) ppm. ^13^C NMR (150 MHz, CDCl_3_): *δ* = 14.3 (CH_2_*C*H_3_), 20.9 (*C*H_3_CO), 23.0 (C=C*C*H_2_), 23.2 (*C*H_2_CH_2_O), 29.4 (Fur-CH_2_), 37.1 (C-4), 56.5 (C-5), 57.7 (C-3), 62.0 (*C*H_2_CH_3_), 67.0 (AcOCH_2_), 68.4 (CH_2_*C*H_2_O), 83.9 (NC=*C*), 112.0 (Fur-C-4), 118.7 (Fur-C-3), 141.3 (Fur-C-5), 143.5 (Fur-C-2), 164.9 (N*C*=C), 169.7 (C=C*C*O), 170.9 (CH_3_*C*O), 173.4 (*C*O_2_Et) ppm. MS (CI): *m*/*z* (%) = 392 (100) [MH]^+^. HRMS (CI): calcd. for C_20_H_26_NO_7_ [MH]^+^ 392.1909; found 392.1706.

**Ethyl (1*R*,8*R*,10*S*)-10-Acetoxymethyl-13-(but-3-enyl)-12-oxo-3-oxa-14-thia-11-azatetracyclo[6.6.0.0^1,11^.0^2,6^]tetradeca-2(6),4-diene-8-carboxylate (21) and Ethyl (3*E*,6*R*,8*S*)-8-Acetoxymethyl-3-(but-3-enylidene)-6-(furan-3-ylmethyl)-2-oxo-4-thia-1-azabicyclo[3.3.0]octane-6-carboxylate (22):** A stirred solution of thiolactam **16** (136 mg, 0.418 mmol) in toluene (2 mL) was treated with acyl chloride **18** (129 mg, 0.610 mmol) in toluene (2 mL). The reaction mixture was heated at reflux for 7 h and then concentrated in vacuo. Purification by flash chromatography (SiO_2_, 10–20 % EtOAc in hexane) gave bicycle **22** (64 mg, 36 %) as a yellow oil. [*α*]_D_^20^ = –19 (*c* = 1.0, CHCl_3_). IR (film): 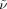
_max_ = 2937, 2907, 2882, 1738, 1683, 1634 cm^–1^. ^1^H NMR (600 MHz, CDCl_3_): *δ* = 1.30 (t, ^3^*J*_H,H_ = 7.2 Hz, 3 H, CH_2_C*H*_3_), 2.08 (s, 3 H, C*H*_3_CO), 2.26 (ddd, ^2^*J*_H,H_ = 13.9, ^3^*J*_H,H_ = 8.7, 1.2 Hz, 1 H, 7-H), 2.35 (dd, ^2^*J*_H,H_ = 13.9, ^3^*J*_H,H_ = 7.7 Hz, 1 H, 7-H), 2.90 (br. t, ^3^*J*_H,H_ = 6.9 Hz, 2 H, CH_2_=CHC*H*_2_), 3.20 (d, ^2^*J*_H,H_ = 14.9 Hz, 1 H, Fur-CH_2_), 4.11–4.33 (m, 5 H, AcOCH_2_, 8-H, C*H*_2_CH_3_), 5.08 (br. d, ^3^*J*_H,H_ = 10.1 Hz, 1 H, CH=C*H*_2_), 5.14 (br. d, ^3^*J*_H,H_ = 17.0 Hz, 1 H, CH=C*H*_2_), 5.20 (s, 1 H, 5-H), 5.82 (ddt, ^3^*J*_H,H_ = 16.9, 10.1, 6.3 Hz, 1 H, C*H*=CH_2_), 6.13 (s, 1 H, Fur-H), 6.78 (t, ^3^*J*_H,H_ = 7.4 Hz, 1 H, C=CH), 7.20 (s, 1 H, Fur-H), 7.35 (br. s, 1 H, Fur-H) ppm. ^13^C NMR (150 MHz, CDCl_3_): *δ* = 14.3 (CH_2_*C*H_3_), 21.0 (*C*H_3_CO), 25.5 (Fur-CH_2_), 33.3 (C-7), 34.7 (CH_2_=CH*C*H_2_), 53.4 (C-8), 55.1 (C-6), 61.9 (*C*H_2_CH_3_), 64.9 (AcOCH_2_), 67.3 (C-5), 111.5 (Fur-C-4), 116.9 (*C*H_2_=CH), 118.6 (Fur-C-3), 126.4 (C=*C*H), 130.9 (*C*=CH), 133.2 (CH_2_=*C*H), 140.9 (Fur-C-2), 143.3 (Fur-C-5), 166.4 (C-2), 170.8 (CH_3_*C*O), 172.1 (*C*O_2_Et) ppm. MS (CI): *m*/*z* (%) = 420 (100) [MH]^+^. HRMS (CI): calcd. for C_21_H_26_NO_6_S [MH]^+^ 420.1481; found 420.1479.

Further elution (20 % EtOAc in hexane) gave tetracycle **21** (37 mg, 21 %, 4:1 ratio of diastereoisomers) as a yellow oil. IR (film): 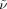
_max_ = 2937, 2907, 2878, 1734, 1685, 1640 cm^–1^. ^1^H NMR (600 MHz, CDCl_3_): *δ* = 1.30 (t, ^3^*J*_H,H_ = 6.8 Hz, 2.4 H, CH_2_C*H*_3_^maj^), 1.32 (t, ^3^*J*_H,H_ = 7.2 Hz, 0.6 H, CH_2_C*H*_3_^min^), 1.75 (m, 0.8 H, SCHC*H*_2_^maj^), 2.06 (s, 2.4 H, CH_3_CO^maj^), 2.07 (s, 0.6 H, CH_3_CO^min^), 2.09–2.15 (m, 1.8 H, CH_2_=CHC*H*_2_^maj^, SCHC*H*_2_^min^), 2.15–2.20 (m, 1 H, 9-H), 2.21–2.37 (m, 1.4 H, SCHC*H*_2_, CH_2_=CHC*H*_2_^min^), 2.60 (d, ^2^*J*_H,H_ = 15.1 Hz, 0.2 H, 7-H^min^), 2.61 (d, ^2^*J*_H,H_ = 15.4 Hz, 0.8 H, 7-H^maj^), 2.89 (dd, ^2^*J*_H,H_ = 13.4, ^3^*J*_H,H_ = 8.7 Hz, 0.2 H, 9-H^min^), 2.99 (dd, ^2^*J*_H,H_ = 13.6, ^3^*J*_H,H_ = 8.7 Hz, 0.8 H, 9-H^maj^), 3.36 (d, ^2^*J*_H,H_ = 15.4 Hz, 0.8 H, C7-H^maj^), 3.40 (d, ^2^*J*_H,H_ = 15.1 Hz, 0.2 H, 7-H^min^), 3.73 (m, 0.8 H, 10-H^maj^), 3.77 (dd, ^3^*J*_H,H_ = 10.0, 3.8 Hz, 0.2 H, 13-H^min^), 4.14 (dd, ^2^*J*_H,H_ = 11.3, ^3^*J*_H,H_ = 5.2 Hz, 0.2 H, AcOCH_2_^min^), 4.18–4.31 (m, 1.4 H, C*H*_2_CH_3_, 10-H^min^, AcOCH_2_^min^), 4.38 (dd, ^3^*J*_H,H_ = 9.3, 3.7 Hz, 0.8 H, 13-H^maj^), 4.55 (dd, ^2^*J*_H,H_ = 11.3, ^3^*J*_H,H_ = 6.7 Hz, 0.8 H, AcOCH_2_^maj^), 4.92 (dd, ^2^*J*_H,H_ = 11.3, ^3^*J*_H,H_ = 4.4 Hz, 0.8 H, AcOCH_2_^maj^), 4.99–5.08 (m, 2 H, C*H*_2_=CH), 5.74–5.84 (m, 1 H, CH_2_=C*H*), 6.23 (d, ^3^*J*_H,H_ = 2.0 Hz, 0.8 H, 5-H^maj^), 6.24 (d, ^3^*J*_H,H_ = 2.0 Hz, 0.2 H, 5-H^min^), 7.43 (d, ^3^*J*_H,H_ = 2.0 Hz, 0.8 H, 4-H^maj^), 7.47 (d, ^3^*J*_H,H_ = 2.0 Hz, 0.2 H, 4-H^min^) ppm. ^13^C NMR (150 MHz, CDCl_3_): *δ* = 14.32 (CH_2_*C*H_3_^min^), 14.36 (CH_2_*C*H_3_^maj^), 21.01 (CO*C*H_3_^maj^), 21.04 (CO*C*H_3_^min^), 31.3 (CH_2_=CH*C*H_2_), 31.9 (SCH*C*H_2_^min^), 32.0 (SCH*C*H_2_^maj^), 33.4 (C-7^min^), 34.4 (C-7^maj^), 42.1 (C-9^maj^), 43.1 (C-9^min^), 51.1 (C-13^maj^), 51.9 (C-13^min^), 55.4 (C-10^maj^), 56.0 (C-10^min^), 61.8 (AcOCH_2_^maj^), 61.95 (AcOCH_2_^min^), 62.0 (*C*H_2_CH_3_^min^), 62.1 (*C*H_2_CH_3_^maj^), 66.3 (C-8^maj^), 67.0 (C-8^min^), 78.0 (C-1^maj^), 79.4 (C-1^min^), 108.3 (C-5^maj^), 108.5 (C-5^min^), 116.08 (*C*H_2_=CH^maj^), 116.13 (*C*H_2_=CH^min^), 125.8 (C-6^maj^), 126.6 (C-6^min^), 136.9 (CH_2_=*C*H^maj^), 137.1 (CH_2_=*C*H^min^), 149.3 (C-4^maj^), 149.7 (C-4^min^), 153.4 (C-2^min^), 154.5 (C-2^maj^), 170.7 (C-12^maj^), 170.8 (CH_3_*C*O^maj^), 170.9 (CH_3_*C*O^min^), 171.8 (C-12^min^), 171.9 (*C*O_2_Et^maj^), 172.0 (*C*O_2_Et^min^) ppm. MS (CI): *m*/*z* (%) = 420 (100) [MH]^+^, 378 (16), 360 (52) [MH – HOAc]^+^, 292 (22). HRMS (CI): calcd. for C_21_H_26_NO_6_S [MH]^+^ 420.1481; found 420.1475.

**(5*S*)-2,2-Dimethyl-3-oxa-1-azabicyclo[3.3.0]octan-8-one (27):**^18^ A stirred suspension of L-pyroglutaminol (10.9 g, 94.7 mmol) in toluene (100 mL) was treated with 2,2-dimethoxypropane (15.1 mL, 123 mmol) and *p*-toluenesulfonic acid (360 mg, 1.89 mmol) and then heated at reflux for 2 h. MeOH was removed from the reaction mixture by distillation and further 2,2-dimethoxypropane (15.1 mL, 123 mmol) was added. The reaction mixture was heated at reflux for 30 min and then concentrated in vacuo. Purification by flash chromatography (SiO_2_, 50–75 % Et_2_O in hexane) gave acetonide **27** (10.6 g, 72 %) as a colourless solid, m.p. 24–25 °C (ref.17b = 36–37 °C). IR (solid): 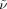
_max_ = 2980, 2933, 2888, 1670 cm^–1^. ^1^H NMR (600 MHz, CDCl_3_): *δ* = 1.45 (s, 3 H, CH_3_), 1.65 (s, 3 H, CH_3_), 1.75 (tt, ^2^*J*_H,H_ = 12.2, ^3^*J*_H,H_ = 12.2, 9.0 Hz, 1 H, 6-H), 2.16 (m, 1 H, 6-H), 2.53 (dd, ^2^*J*_H,H_ = 16.6, ^3^*J*_H,H_ = 9.1 Hz, 1 H, 7-H), 2.79 (ddd, ^2^*J*_H,H_ = 16.6, ^3^*J*_H,H_ = 12.1, 8.5 Hz, 1 H, 7-H), 3.44 (t, ^2^*J*_H,H_ = ^3^*J*_H,H_ = 8.8 Hz, 1 H, 4-H), 4.07 (dd, ^2^*J*_H,H_ = 8.2, ^3^*J*_H,H_ = 5.7 Hz, 1 H, 4-H), 4.25 (tt, ^3^*J*_H,H_ = 8.8, 6.0 Hz, 1 H, 5-H) ppm. ^13^C NMR (150 MHz, CDCl_3_): *δ* = 23.9 (CH_3_), 24.4 (C-6), 26.9 (CH_3_), 37.3 (C-7), 61.7 (C-5), 70.0 (C-4), 91.4 (C-2), 171.6 (C-8) ppm.

**Ethyl (5*S*)-2,2-Dimethyl-8-oxo-3-oxa-1-azabicyclo[3.3.0]octane-7-carboxylate (28):** A flask charged with LHMDS (1 M in toluene, 0.64 mL, 0.64 mmol) at –78 °C was treated dropwise with a solution of lactam **27** (500 mg, 0.322 mmol) and diethyl carbonate (0.507 mL, 4.19 mmol) in THF (12 mL) over 30 min. The reaction mixture was stirred for 1 h and then warmed to 0 °C and quenched with AcOH (0.92 mL). Et_2_O (12 mL) was added and the mixture was concentrated in vacuo. Purification by flash chromatography (SiO_2_, 70 % Et_2_O in hexane) gave ester **28** (663 mg, 91 %, 6:4 mixture of diastereoisomers) as a colourless oil. IR (film): 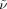
_max_ = 2984, 2938, 2873, 1735, 1694 cm^–1^. ^1^H NMR (600 MHz, CDCl_3_): *δ* = 1.30 (t, ^3^*J*_H,H_ = 7.2 Hz, 1.2 H, CH_2_C*H*_3_^min^), 1.31 (t, ^3^*J*_H,H_ = 7.2 Hz, 1.8 H, CH_2_C*H*_3_^maj^), 1.46 (s, 1.8 H, CH_3_^maj^), 1.46 (s, 1.2 H, CH_3_^min^), 1.65 (s, 1.8 H, CH_3_^maj^), 1.66 (s, 1.2 H, CH_3_^min^), 1.96 (dt, ^2^*J*_H,H_ = 12.9, ^3^*J*_H,H_ = 8.8 Hz, 0.4 H, 6-H^min^), 2.24 (td, ^2^*J*_H,H_ = 12.1, ^3^*J*_H,H_ = 8.8 Hz, 0.6 H, 6-H^maj^), 2.36 (ddd, ^2^*J*_H,H_ = 12.6, ^3^*J*_H,H_ = 7.7, 6.2 Hz, 0.6 H, 6-H^maj^), 2.49 (dd, ^3^*J*_H,H_ = 12.7, ^3^*J*_H,H_ = 6.2 Hz, 0.4 H, 6-H^min^), 3.45 (t, ^2^*J*_H,H_ = ^3^*J*_H,H_ = 8.9 Hz, 0.4 H, 4-H^min^), 3.55 (t, ^2^*J*_H,H_ = ^3^*J*_H,H_ = 8.8 Hz, 0.6 H, 4-H^maj^), 3.60 (d, ^3^*J*_H,H_ = 9.2 Hz, 0.4 H, 7-H^min^), 3.81 (dd, ^3^*J*_H,H_ = 11.7, 8.0 Hz, 0.6 H, 7-H^maj^), 4.07–4.14 (m, 1 H, 4-H), 4.14–4.30 (m, 2.6 H, C*H*_2_CH_3_, 5-H^maj^), 4.47 (tt, ^3^*J*_H,H_ = 8.9, 6.1 Hz, 0.4 H, 5-H^min^) ppm. ^13^C NMR (150 MHz, CDCl_3_): *δ* = 14.2 (CH_2_*C*H_3_^min^), 14.3 (CH_2_*C*H_3_^maj^), 23.7 (CH_3_^min^), 23.8 (CH_3_^maj^), 26.7 (CH_3_^min^), 26.8 (CH_3_^maj^), 28.0 (C-6^maj^), 28.2 (C-6^min^), 54.2 (C-7^maj^), 55.3 (C-7^min^), 59.2 (C-5^maj^), 60.8 (C-5^min^), 61.8 (*C*H_2_CH_3_^maj^), 61.9 (*C*H_2_CH_3_^min^), 69.8 (C-4^maj^), 69.9 (C-4^min^), 91.8 (C-2^min^), 91.9 (C-2^maj^), 166.0 (C-8^min^), 166.4 (C-8^maj^), 169.5 (*C*O_2_Et^maj^), 169.7 (*C*O_2_Et^min^) ppm. MS (ES^+^): *m*/*z* (%) = 250 (50) [MNa]^+^. HRMS (ES^+^): calcd. for C_11_H_17_NO_4_Na [MNa]^+^ 250.1055; found 250.1055.

**(5*S*)-2,2-Dimethyl-3-oxa-1-azabicyclo[3.3.0]octane-8-thione (29):** A stirred solution of ethyl L-pyroglutamate (**30**;^21^ 2.00 g, 12.7 mmol) in THF (30 mL) was treated with Lawesson's reagent (2.60 g, 6.33 mmol) and then stirred for 2 h. The reaction mixture was concentrated in vacuo. Purification by repeated flash chromatography (SiO_2_, 40 % EtOAc in hexane and then SiO_2_, 65 % Et_2_O in hexane) gave ethyl (*S*)-5-thioxopyrrolidine-2-carboxylate (**30a**; 1.80 g, 82 %) as a colourless solid, m.p. 37–38 °C (ref.^22^ = 35–36 °C). IR (solid): 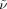
_max_ = 3300 (br), 2982, 2941, 2875, 1743, 1716, 1524 cm^–1^. ^1^H NMR (600 MHz, CDCl_3_): *δ* = 1.30 (t, ^3^*J*_H,H_ = 7.2 Hz, 3 H, CH_3_), 2.35 (ddt, ^2^*J*_H,H_ = 13.2, ^3^*J*_H,H_ = 9.3, 6.7 Hz, 1 H, 3-H), 2.56 (dtd, ^2^*J*_H,H_ = 13.2, ^3^*J*_H,H_ = 8.9, 5.9 Hz, 1 H, 3-H), 2.92 (ddd, ^2^*J*_H,H_ = 18.2, ^3^*J*_H,H_ = 9.0, 7.3 Hz, 1 H, 4-H), 2.99 (ddd, ^2^*J*_H,H_ = 18.2, ^3^*J*_H,H_ = 9.4 6.0 Hz, 1 H, 4-H), 4.20–4.29 (m, 2 H, C*H*_2_CH_3_), 4.51 (dd, ^3^*J*_H,H_ = 8.7, 6.4 Hz, 1 H, 2-H), 8.10 (br. s, 1 H, NH) ppm. ^13^C NMR (150 MHz, CDCl_3_): *δ* = 14.2 (CH_3_), 27.2 (C-3), 42.7 (C-4), 62.3 (*C*H_2_CH_3_), 62.6 (C-2), 170.1 (*C*O_2_Et), 206.8 (C-5) ppm.

A stirred solution of ester **30a** (11.1 g, 64.1 mmol) in THF (100 mL) was treated with LiBH_4_ (1.50 g, 70.5 mmol) and then stirred for 1 h. The reaction mixture was cooled to 0 °C and treated with 20 % aqueous acetic acid (40 mL) and then adsorbed onto silica gel. Purification by flash chromatography (SiO_2_, 6 % MeOH in CHCl_3_) gave (*S*)-5-hydroxymethylpyrrolidine-2-thione (**30b**; 7.43 g, 88 %) as a colourless solid, m.p. 125–126 °C (ref.^22^ = 124–125 °C). IR (solid): 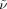
_max_ = 3178 (br), 3046 (br), 2973, 2932, 2877, 1532 cm^–1^. ^1^H NMR (600 MHz, [D_6_]DMSO): *δ* = 1.85 (dddd, ^2^*J*_H,H_ = 12.8, ^3^*J*_H,H_ = 9.4, 6.0, 5.3 Hz, 1 H, 4-H), 2.09 (dddd, ^3^*J*_H,H_ = 12.7, ^3^*J*_H,H_ = 9.6, 8.4, 6.6 Hz, 1 H, 4-H), 2.65 (ddd, ^2^*J*_H,H_ = 17.8, ^3^*J*_H,H_ = 9.8, 6.4 Hz, 1 H, 3-H), 2.73 (ddd, ^2^*J*_H,H_ = 18.1, ^3^*J*_H,H_ = 9.4, 6.4 Hz, 1 H, 3-H), 3.37–3.44 (m, 2 H, C*H*_2_OH), 3.84 (dt, ^3^*J*_H,H_ = 13.2, 4.5 Hz, 1 H, 5-H), 4.91 (t, ^3^*J*_H,H_ = 5.5 Hz, 1 H, OH), 10.12 (br. s, 1 H, NH) ppm. ^13^C NMR (150 MHz, [D_6_]DMSO): *δ* = 24.7 (C-4), 43.2 (C-3), 62.7 (CH_2_OH), 63.6 (C-5), 203.7 (C-2) ppm.

A stirred suspension of thiolactam **30b** (0.40 g, 3.1 mmol) in toluene (4 mL) was treated with 2,2-dimethoxypropane (0.487 mL, 3.96 mmol) and *p*-toluenesulfonic acid (12 mg, 0.061 mmol). The mixture was heated at reflux for 3 h and then concentrated in vacuo. Purification by flash chromatography (SiO_2_, 30–50 % Et_2_O in petroleum ether) gave bicycle **29** (268 mg, 51 %) as a colourless solid, m.p. 74–75 °C; [*α*]_D_^20^ = +241 (*c* = 1.0, CHCl_3_). IR (CHCl_3_ cast): 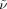
_max_ = 2987, 2940, 2871, 1476 cm^–1^. ^1^H NMR (600 MHz, CDCl_3_): *δ* = 1.67 (s, 3 H, CH_3_), 1.80 (tdd, ^2^*J*_H,H_ = 12.2, ^3^*J*_H,H_ = 12.2, 10.3, 8.5 Hz, 1 H, 6-H), 1.83 (s, 3 H, CH_3_), 2.17 (dt, ^2^*J*_H,H_ = 12.2, ^3^*J*_H,H_ = 6.3 Hz, 1 H, 6-H), 3.25 (dd, ^2^*J*_H,H_ = 17.3, ^3^*J*_H,H_ = 8.4 Hz, 1 H, 7-H), 3.34 (ddd, ^2^*J*_H,H_ = 17.3, ^3^*J*_H,H_ = 12.5, 7.3 Hz, 1 H, 7-H), 3.53 (dd, ^3^*J*_H,H_ = 10.0, ^2^*J*_H,H_ = 8.6 Hz, 1 H, 4-H), 4.10 (dd, ^2^*J*_H,H_ = 8.6, ^3^*J*_H,H_ = 5.5 Hz, 1 H, 4-H), 4.49 (tt, ^3^*J*_H,H_ = 10.5, 5.5 Hz, 1 H, 5-H) ppm. ^13^C NMR (150 MHz, CDCl_3_): *δ* = 22.2 (CH_3_), 24.9 (CH_3_), 26.1 (C-6), 52.5 (C-7), 68.0 (C-4), 69.6 (C-5), 93.4 (C-2), 195.8 (C-8) ppm. MS (CI): *m*/*z* (%) = 172 (100) [MH]^+^. HRMS (CI): calcd. for C_8_H_14_NOS [MH]^+^ 172.0796; found 172.0791. C_8_H_14_NOS (171.26): C 56.10, H 7.65, N 8.18, found C 56.22, H 7.71, N 8.16.

**(5*S*,7*S*,1′*R*)-2,2-Dimethyl-7-(1-phenylprop-2-enyl)-3-oxa-1-azabicyclo[3.3.0]octane-8-thione (36):** A stirred solution of thioamide **29** (205 mg, 1.20 mmol) in THF (5 mL) at 0 °C was treated with *n*-butyllithium (2.5 M in hexane, 0.55 mL, 1.3 mmol) and then stirred for 15 min. The reaction mixture was treated with cinnamyl bromide (260 mg, 1.32 mmol) in THF (1 mL) and stirred at 0 °C for 30 min and then at room temp. for 1 h. A saturated aq. NaHCO_3_ solution (6 mL) was added and the organic material extracted with Et_2_O (3 × 6 mL). The combined organic extracts were washed with brine (6 mL), dried (MgSO_4_) and then concentrated in vacuo. Purification by flash chromatography (SiO_2_, 20 % Et_2_O in hexane) gave thioamide **36** (252 mg, 73 %, 92 % *de*) as a colourless solid, m.p. 119–120 °C; [*α*]_D_^20^ = –94 (*c* = 0.35, CHCl_3_). IR (CH_2_Cl_2_ cast): 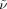
_max_ = 2984, 2928, 2869, 1484 cm^–1^. ^1^H NMR (600 MHz, CDCl_3_, data for major diastereoisomer): *δ* = 1.40 (s, 3 H, CH_3_), 1.80 (s, 3 H, CH_3_), 1.90 (dt, ^2^*J*_H,H_ = 12.7, ^3^*J*_H,H_ = 9.7 Hz, 1 H, C6-H), 2.09 (dd, ^2^*J*_H,H_ = 12.7, ^3^*J*_H,H_ = 6.5 Hz, 1 H, 6-H), 2.82 (tt, ^3^*J*_H,H_ = 10.0, 6.0 Hz, 1 H, 5-H), 3.25 (dd, ^3^*J*_H,H_ = 10.2, ^2^*J*_H,H_ = 8.4 Hz, 1 H, 4-H), 3.73 (dd, ^2^*J*_H,H_ = 8.4, ^3^*J*_H,H_ = 5.6 Hz, 1 H, 4-H), 3.78 (dd, ^3^*J*_H,H_ = 9.4, 3.1 Hz, 1 H, 1′-H), 4.34 (m, 1 H, 7-H), 5.15 (ddd, ^3^*J*_H,H_ = 17.4, ^2^*J*_H,H_ = 1.9, ^4^*J*_H,H_ = 1.1 Hz, 1 H, 3′-H), 5.28 (ddd, ^3^*J*_H,H_ = 10.6, ^2^*J*_H,H_ = 1.8, ^4^*J*_H,H_ = 0.9 Hz, 1 H, 3′-H), 6.20 (ddd, ^3^*J*_H,H_ = 17.5, 10.6, 5.4 Hz, 1 H, 2′-H), 7.26–7.38 (m, 5 H, Ar-H) ppm. ^13^C NMR (150 MHz, CDCl_3_, data for major diastereoisomer): *δ* = 22.3 (CH_3_), 24.3 (CH_3_), 26.4 (C-6), 50.1 (C-7), 66.8 (C-1′), 68.3 (C-4), 68.4 (C-5), 93.2 (C-2), 115.6 (C-3′), 127.7 (CH), 128.4 (CH), 128.9 (CH), 138.8 (C-2′), 139.5 (C), 197.6 (C-8) ppm. MS (EI): *m*/*z* (%) = 287 (23) [M]^+^, 117 (17) [PhCHCH=CH_2_]^+^, 86 (100). HRMS (EI): calcd. for C_17_H_21_NOS [M]^+^ 287.1339; found 287.1341.

**(5*S*,7*S*,1′*R*)-2,2-Dimethyl-*N*-phenyl-7-(1-phenylprop-2-enyl)-8-thioxo-3-oxa-1-azabicyclo[3.3.0]octane-7-carboxamide (37):** A stirred solution of thioamide **29** (141 mg, 0.824 mmol) in THF (2 mL) at 0 °C was treated with *n*-butyllithium (2.02 M in hexanes, 0.40 mL, 0.81 mmol) and then stirred for 25 min. The reaction mixture was cooled to –78 °C and then treated with (*Z*)-cinnamyl bromide (195 mg, 0.99 mmol) in THF (2 mL). The reaction mixture was stirred for 15 min and then warmed to 0 °C and treated with AcOH (3 μL, 50 μmol) followed immediately by PhNCO (120 μL, 1.1 mmol). The reaction mixture was warmed to room temp. over 18 h and then quenched with brine (4 mL). The organic material was extracted with CH_2_Cl_2_ (3 × 5 mL) and the combined extracts were dried (hydrophobic frit) and then concentrated in vacuo. Purification by flash chromatography (SiO_2_, 13 % EtOAc in hexane) gave thioamide **37** (155 mg, 46 %, 96 % *de*) as a colourless solid. M.p 116–117 °C; [*α*]_D_^25^ = +56.8 (*c* = 1.02, CHCl_3_). IR (solid): 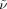
_max_ = 3184, 2986, 2926, 2863, 1670, 1549 cm^–1^. ^1^H NMR (600 MHz, CDCl_3_): *δ* = 1.56 (s, 3 H, CH_3_), 1.77 (s, 3 H, CH_3_), 2.39 (dd, ^2^*J*_H,H_ = 13.6, ^3^*J*_H,H_ = 9.9 Hz, 1 H, 6-H), 2.59 (dd, ^2^*J*_H,H_ = 13.6, ^3^*J*_H,H_ = 6.6 Hz, 1 H, 6-H), 2.95 (tt, ^3^*J*_H,H_ = 10.1, 6.0 Hz, 1 H, 5-H), 3.26 (dd, ^3^*J*_H,H_ = 10.3, ^2^*J*_H,H_ = 8.5 Hz, 1 H, 4-H), 3.86 (dd, ^2^*J*_H,H_ = 8.5, ^3^*J*_H,H_ = 5.5 Hz, 1 H, 4-H), 4.01 (d, ^3^*J*_H,H_ = 9.6 Hz, 1 H, 1′-H), 5.08 (dd, ^3^*J*_H,H_ = 10.1, ^2^*J*_H,H_ = 1.2 Hz, 1 H, 3′-H), 5.12 (d, ^3^*J*_H,H_ = 16.8 Hz, 1 H, 3′-H), 6.69 (dt, ^3^*J*_H,H_ = 16.8, 9.8 Hz, 1 H, 2′-H), 7.11 (tt, ^3^*J*_H,H_ = 7.3, ^4^*J*_H,H_ = 1.1 Hz, 1 H, Ar-H), 7.28–7.36 (m, 5 H, Ar-H), 7.43 (m, 2 H, Ar-H), 7.55 (m, 2 H, Ar-H), 11.31 (s, 1 H, NH) ppm. ^13^C NMR (150 MHz, CDCl_3_): *δ* = 22.0 (CH_3_), 24.0 (CH_3_), 33.5 (C-6), 59.9 (C-1′), 64.4 (C-5), 68.6 (C-4), 77.1 (C-7), 94.5 (C-2), 118.1 (C-3′), 120.1 (CH), 124.5 (CH), 128.2 (CH), 128.9 (CH), 129.0 (CH), 129.1 (CH), 136.1 (C-2′), 137.7 (C), 138.7 (C), 168.7 (CONH), 192.9 (C-8) ppm. MS (CI): *m*/*z* (%) = 407 (8) [MH]^+^, 349 (23) [MH – OC(CH_3_)_2_]^+^, 291 (100), 233 (73), 119 (28), 82 (69), 56 (28). HRMS (CI): calcd. for C_24_H_27_N_2_O_2_S [MH]^+^ 407.1793; found 407.1796.

**Supporting Information** (see footnote on the first page of this article): 1D ^1^H and ^13^C NMR spectra, and selected 2D NMR spectra of all compounds synthesised.

## Supporting Information

(see footnote on the first page of this article): 1D ^1^H and ^13^C NMR spectra, and selected 2D NMR spectra of all compounds synthesised.
