# Supplementary material for: Synthetic Studies Towards the Core Structure of Nakadomarin A by a Thioamide-Based Strategy
Source: European J Org Chem. 2013 Nov 14;2014(1):129–39. doi: 10.1002/ejoc.201301063 (PMC4015372; doi:10.1002/ejoc.201301063)
Supplement: Supplementary file 1 [file ejoc2014-0129-sd1.pdf]

*Eur. J. Org. Chem.* **2013** · © WILEY-VCH Verlag GmbH & Co. KGaA, 69451 Weinheim, 2013 · ISSN 1099–0690

# ***SUPPORTING INFORMATION***

***DOI:*** 10.1002/ejoc.201301063
***Title:*** Synthetic Studies Towards the Core Structure of Nakadomarin A by a Thioamide-Based Strategy
***Author(s):*** Jai K. Chavda, Panayiotis A. Procopiou, Peter N. Horton, Simon J. Coles, Michael J. Porter*

## NMR Spectra


**1-Benzyl-3-methylpyrrolidin-2-one (8a)**

$^{1}$H 3-4
$^{13}$C 5
COSY 6
DEPT-135 7
HSQC 8
HMBC 9

**1-Benzyl-3-(furan-3-ylmethyl)-3-methylpyrrolidin-2-one (10)**

$^{1}$H 10
$^{13}$C 11

**3-(Furan-3-ylmethyl)-3-methylpyrrolidin-2-one (10a)**

$^{1}$H 12
$^{13}$C 13
HSQC 14

**3-(Furan-3-ylmethyl)-3-methylpyrrolidine-2-thione (11)**

$^{1}$H 15
$^{13}$C 16
DEPT-135 17

**(6a*RS*,10b*RS*)-6a-Methyl-5,6,6a,7-tetrahydrofuro[3″,2″:4′,5′]-cyclopenta[1′,2′:2,3]pyrrolo[2,1-*b*]thiazol-3(2*H*)-one (12)**

$^{1}$H 18
$^{13}$C 19
COSY 20
HSQC 21
HMBC 22

**(1*S*)-4-(4-Methoxyphenyl)-6-phenyl-4-sulfanylidene-3-oxa-5-thia-7-aza-4-phosphabicyclo[5.3.0]decane-8-thione (14)**

$^{1}$H 23-25
$^{13}$C 26
COSY 27
DEPT-135 28
HSQC 29
HMBC 30

**Ethyl (2*R*,5*S*,7*RS*)-8-oxo-2-phenyl-3-oxa-1-azabicyclo[3.3.0]octane-7-carboxylate (13a)**

$^{1}$H 31
$^{13}$C 32
COSY 33
HSQC 34
HMBC 35

**Ethyl (2*R*,5*S*,7*R*)-7-(furan-3-ylmethyl)-8-oxo-2-phenyl-3-oxa-1-azabicyclo[3.3.0]octane-7-carboxylate (*epi*-15)**

$^{1}$H 36
$^{13}$C 37
COSY 38
HSQC 39
HMBC 40

**Ethyl (2*R*,5*S*,7*S*)-7-(furan-3-ylmethyl)-8-oxo-2-phenyl-3-oxa-1-azabicyclo[3.3.0]octane-7-carboxylate (15)**

$^{1}$H 41
$^{13}$C 42
COSY 43
HSQC 44
HMBC 45

**Ethyl (3*R*,5*S*)-3-(furan-3-ylmethyl)-5-hydroxymethyl-2-oxopyrrolidine-3-carboxylate (15a)**

$^{1}$H 46-48
$^{13}$C 49
COSY 50
DEPT-135 51
HSQC 52
HMBC 53

**Ethyl (3*R*,5*S*)-5-acetoxymethyl-3-(furan-3-ylmethyl)-2-oxopyrrolidine-3-carboxylate (15b)**

$^{1}$H 54
$^{13}$C 55
COSY 56
NOESY 57
DEPT-135 58
HSQC 59
HMBC 60

**Ethyl (3*R*,5*S*)-5-acetoxymethyl-3-(furan-3-ylmethyl)-2-sulfanylidenepyrrolidine-3-carboxylate (16)**

$^{1}$H 61
$^{13}$C 62
COSY 63
DEPT-135 64
HSQC 65
HMBC 66

**6-Trimethylsilanyloxy-3,4-dihydro-2*H*-pyran (17a)**

$^{1}$H 67
$^{13}$C 68

**3-Bromotetrahydro-2*H*-pyran-2-one (17)**
$^{1}$H 69-71
$^{13}$C 72
COSY 73
HSQC 74
HMBC 75

**2-Bromohex-5-enoic acid (18a)**
$^{1}$H 76-78
$^{13}$C 79
COSY 80
HSQC 81
HMBC 82

**2-Bromohex-5-enoyl chloride (18)**
$^{1}$H 83-85
$^{13}$C 86
COSY 87
DEPT-135 88
HSQC 89
HMBC 90

**Ethyl (2*S*,4*R*)-2-acetoxymethyl-4-(furan-3-ylmethyl)-5-(2-oxooxan-3-ylsulfanyl)-3,4-dihydro-2*H*-pyrrole-4-carboxylate (19)**
$^{1}$H 91
$^{13}$C 92
COSY 93
HSQC 94

**Ethyl (2*E*,3*S*,5*S*)-5-Acetoxymethyl-3-(furan-3-ylmethyl)-2-(2-oxooxan-3-ylidene)pyrrolidine-3-carboxylate (20)**
$^{1}$H 95-96
$^{13}$C 97
COSY 98
DEPT-135 99
HSQC 100
HMBC 101

**Ethyl (1*R*,8*R*,10*S*)-10-acetoxymethyl-13-but-3-enyl-12-oxo-3-oxa-14-thia-11-azatetracyclo[6.6.0.0$^{1,11}$.0$^{2,6}$]tetradeca-2(6),4-diene-8-carboxylate (21)**
$^{1}$H 102-103
$^{13}$C 104
COSY 105
DEPT-135 106
HSQC 107
HMBC 108

**Ethyl (3*E*,6*R*,8*S*)-8-acetoxymethyl-3-(but-3-enylidene)-6-(furan-3-ylmethyl)-2-oxo-4-thia-1-azabicyclo[3.3.0]octane-6-carboxylate (22)**
$^{1}$H 109-110
$^{13}$C 111
COSY 112
DEPT-135 113
HSQC 114
HMBC 115

**(5*S*)-2,2-Dimethyl-3-oxa-1-azabicyclo[3.3.0]octan-8-one (27)**
$^{1}$H 116
$^{13}$C 117
COSY 118
DEPT-135 119
HSQC 120
HMBC 121

**Ethyl (5*S*)-2,2-dimethyl-8-oxo-3-oxa-1-azabicyclo[3.3.0]octane-7-carboxylate (28)**
$^{1}$H 122
$^{13}$C 123
COSY 124
DEPT-135 125
HSQC 126
HMBC 127

**Ethyl (*S*)-5-Sulfanylidenepyrrolidine-2-carboxylate (30a)**
$^{1}$H 128
$^{13}$C 129
COSY 130
HSQC 131
HMBC 132

**(*S*)-5-Hydroxymethylpyrrolidine-2-thione (30b)**
$^{1}$H 133
$^{13}$C 134
COSY 135
HSQC 136
HMBC 137

**(5*S*)-2,2-Dimethyl-3-oxa-1-azabicyclo[3.3.0]octane-8-thione (29)**
$^{1}$H 138
$^{13}$C 139
COSY 140
HSQC 141
HMBC 142

**(5*S*,7*S*,1′*R*)-2,2-Dimethyl-7-(1-phenylprop-2-en-1-yl)-3-oxa-1-azabicyclo[3.3.0]octane-8-thione (36)**
$^{1}$H 143
$^{13}$C 144
COSY 145
NOESY 146
HSQC 147
HMBC 148

**(5*S*,7*S*,1′*R*)-2,2-Dimethyl-*N*-phenyl-7-(1-phenylprop-2-en-1-yl)-8-sulfanylidene-3-oxa-1-azabicyclo[3.3.0]octane-8-carboxamide (37)**
$^{1}$H 149-152
$^{13}$C 153
COSY 154
DEPT-135 155
HSQC 156
HMBC 157

JC–253–2

PROTON.ucl CDCl3 {V:\Bruker\TOPSPIN} mjp 40

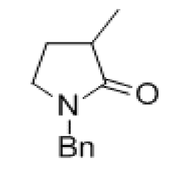


8a

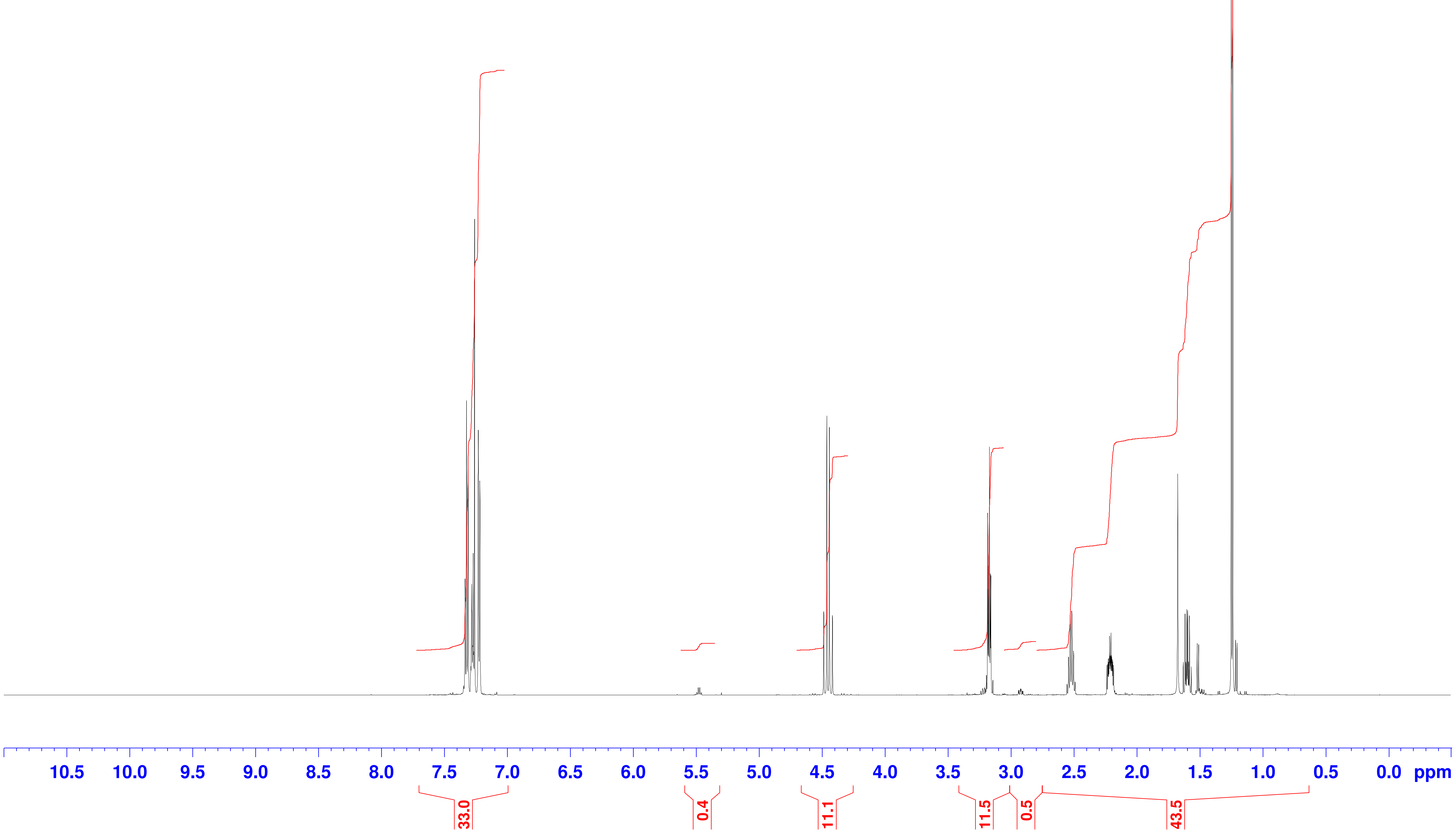


UCL

| | |
|---|---|
| NAME | JC-253-2 |
| EXPNO | 10 |
| PROCNO | 1 |
| Date_ | 20120330 |
| Time | 17.21 |
| INSTRUM | AV600 |
| PROBHD | 5 mm CPDCH 13C |
| PULPROG | zg30 |
| TD | 98682 |
| SOLVENT | CDCl3 |
| NS | 8 |
| DS | 0 |
| SWH | 12335.526 Hz |
| FIDRES | 0.125003 Hz |
| AQ | 3.9999604 sec |
| RG | 36 |
| DW | 40.533 use |
| DE | 10.48 use |
| TE | 298.0 K |
| D1 | 1.00000000 sec |
| TD0 | 1 |

======== CHANNEL f1 ========

| | |
|---|---|
| NUC1 | 1H |
| P1 | 11.40 use |
| PL1 | 1.00 dB |
| PL1W | 13.76731014 W |
| SFO1 | 600.1337061 MHz |
| SI | 32768 |
| SF | 600.1300116 MHz |
| WDW | EM |
| SSB | 0 |
| LB | 0.30 Hz |
| GB | 0 |
| PC | 1.40 |

V:BrukerTOPSPIN/mjp/JC−253−2/10/1
Integral regions #6 to #6
60 Hz/cm

JC−253−2
PROTON.ucl CDCl3 {V:\Bruker\TOPSPIN} mjp 40

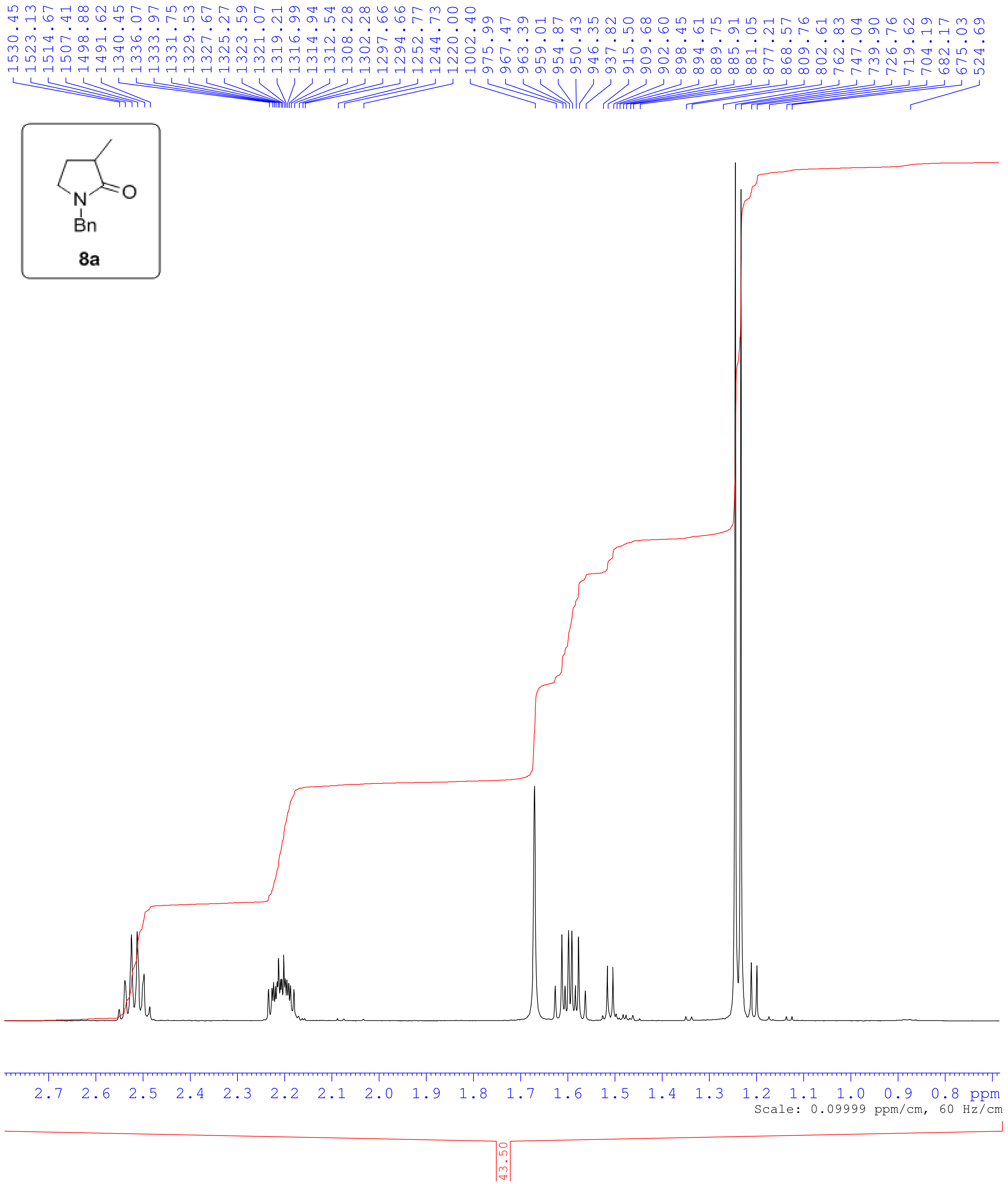

JC–253–2
C13CPD.ucl CDCl3 {V:\Bruker\TOPSPIN} mjp 40

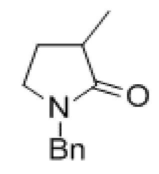


8a

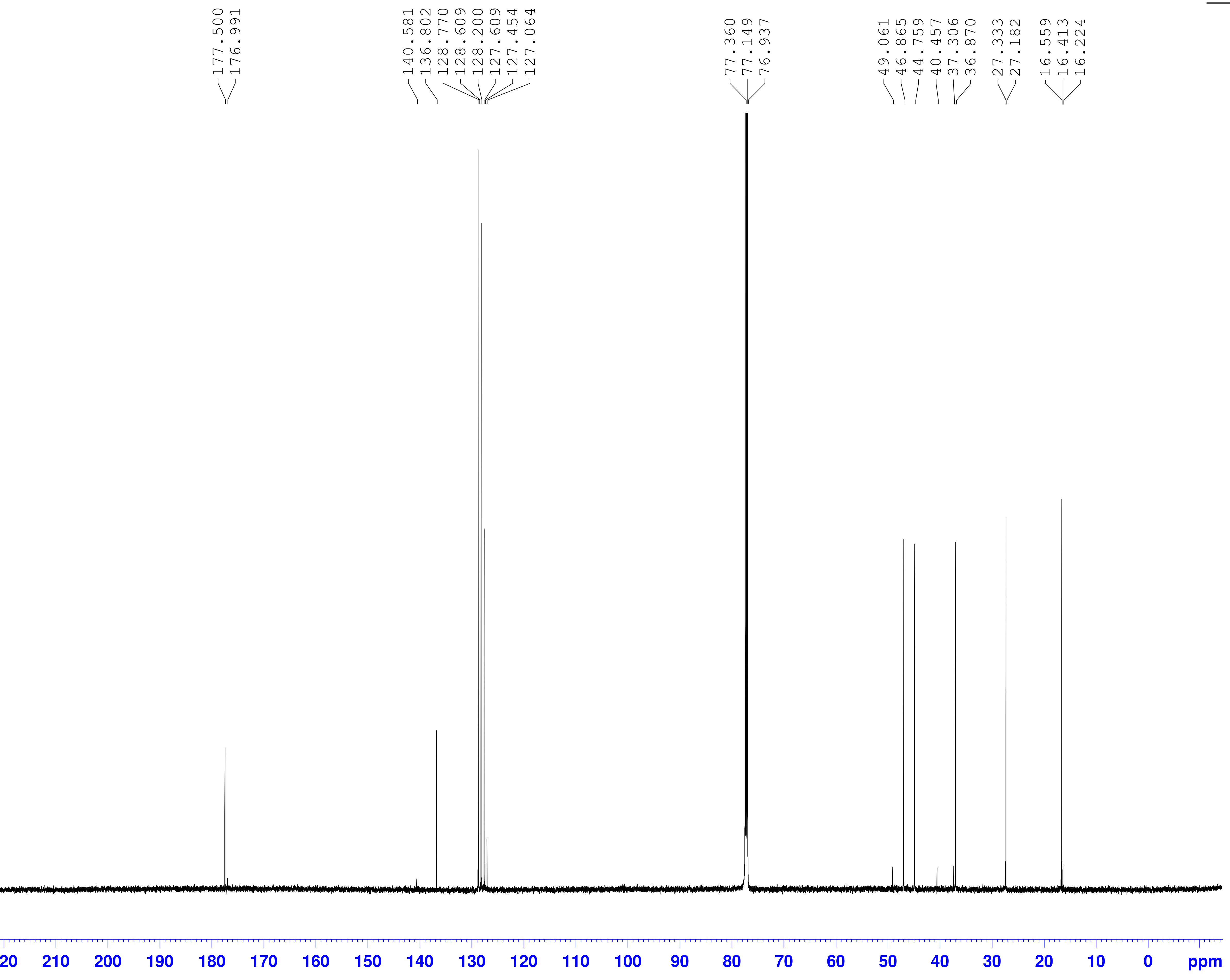


| | |
|---|---|
| NAME | JC-253-2 |
| EXPNO | 12 |
| PROCNO | 1 |
| Date_ | 20120330 |
| Time | 17.33 |
| INSTRUM | AV600 |
| PROBHD | 5 mm CPDCH 13C |
| PULPROG | zgpg30 |
| TD | 70308 |
| SOLVENT | CDCl3 |
| NS | 128 |
| DS | 0 |
| SWH | 39062.500 H |
| FIDRES | 0.555591 H |
| AQ | 0.8999924 s |
| RG | 1030 |
| DW | 12.800 u |
| DE | 21.12 u |
| TE | 298.0 K |
| D1 | 2.00000000 s |
| D11 | 0.03000000 s |
| TD0 | 1 |
| ======== CHANNEL f1 ===== | |
| NUC1 | 13C |
| P1 | 9.80 u |
| PL1 | 5.00 d |
| PL1W | 26.76886177 W |
| SFO1 | 150.9201628 M |
| ======== CHANNEL f2 ===== | |
| CPDPRG2 | waltz16 |
| NUC2 | 1H |
| PCPD2 | 70.00 u |
| PL2 | 1.00 d |
| PL12 | 17.23 d |
| PL13 | 20.00 d |
| PL2W | 13.76731014 W |
| PL12W | 0.32798135 W |
| PL13W | 0.17332016 W |
| SFO2 | 600.1324005 M |
| SI | 65536 |
| SF | 150.9027930 M |
| WDW | EM |
| SSB | 0 |
| LB | 1.00 H |
| GB | 0 |
| PC | 1.40 |

JC–253–2
COSY.ucl CDCl3 {V:\Bruker\TOPSPIN} mjp 40

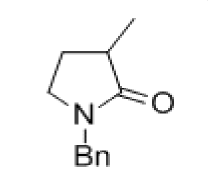

8a

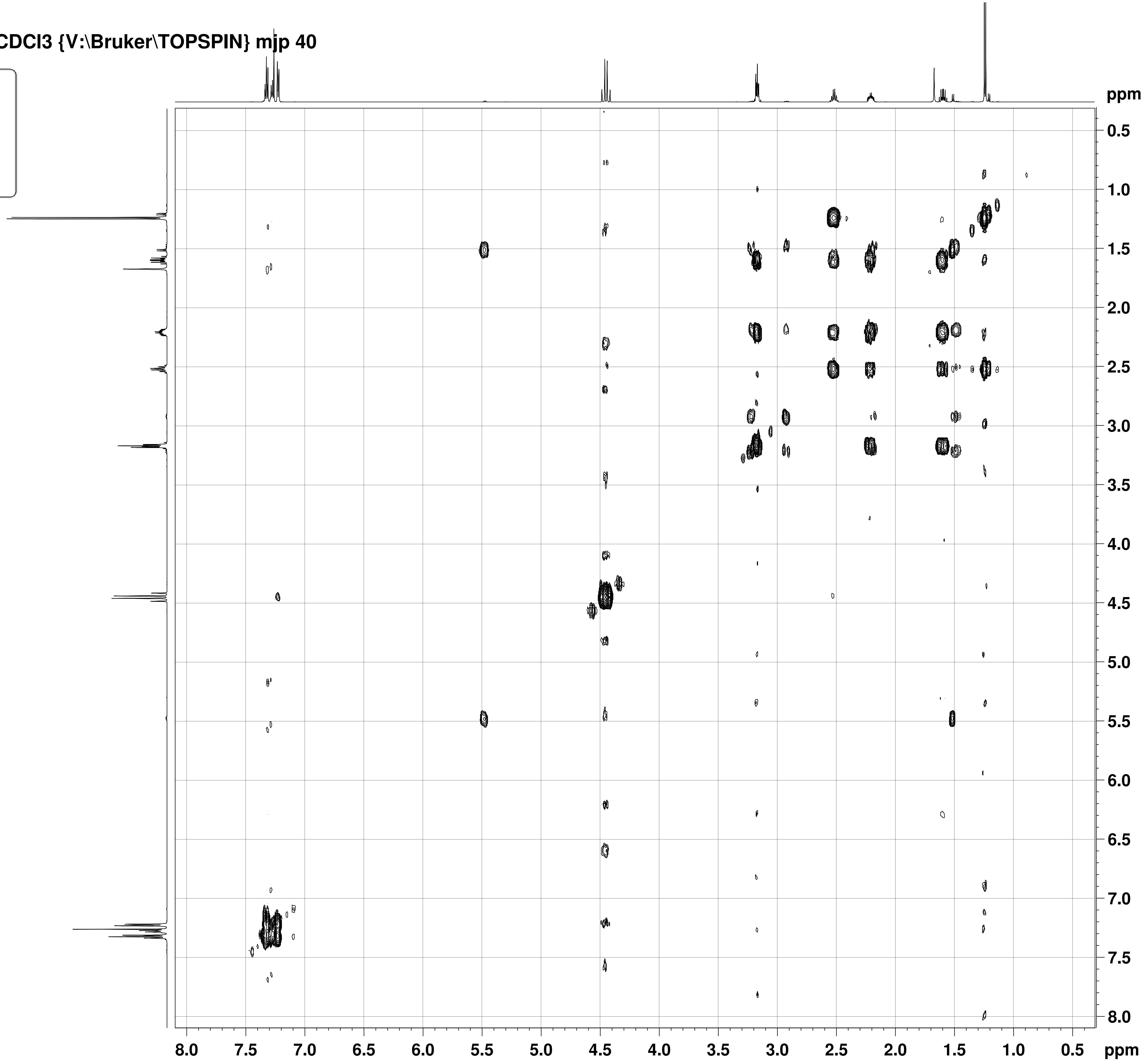


UCL

| | |
|---|---|
| NAME | JC-253-2 |
| EXPNO | 11 |
| PROCNO | 1 |
| Date_ | 20120330 |
| Time | 17.22 |
| INSTRUM | AV600 |
| PROBHD | 5 mm CPDCH 13C |
| PULPROG | cosygpmfqf |
| TD | 2048 |
| SOLVENT | CDCl3 |
| NS | 1 |
| DS | 8 |
| SWH | 4672.897 Hz |
| FIDRES | 2.281688 Hz |
| AQ | 0.2191860 sec |
| RG | 2050 |
| DW | 107.000 usec |
| DE | 6.50 usec |
| TE | 298.0 K |
| D0 | 0.00000300 sec |
| D1 | 1.68092096 sec |
| D13 | 0.00000400 sec |
| D16 | 0.00020000 sec |
| IN0 | 0.00021400 sec |
| ======== CHANNEL f1 ======== | |
| NUC1 | 1H |
| P1 | 11.40 usec |
| PL1 | 1.00 dB |
| PL1W | 13.76731014 W |
| SFO1 | 600.1325341 MHz |
| ====== GRADIENT CHANNEL ==== | |
| GPNAM1 | SINE.100 |
| GPNAM2 | SINE.100 |
| GPNAM3 | SINE.100 |
| GPZ1 | 16.00 % |
| GPZ2 | 12.00 % |
| GPZ3 | 40.00 % |
| P16 | 1000.00 usec |
| ND0 | 1 |
| TD | 128 |
| SFO1 | 600.1325 MHz |
| FIDRES | 36.507008 Hz |
| SW | 7.786 ppm |
| FnMODE | QF |
| SI | 1024 |
| SF | 600.1300095 MHz |
| WDW | QSINE |
| SSB | 0 |
| LB | 0.00 Hz |
| GB | 0 |
| PC | 1.40 |
| SI | 1024 |
| MC2 | QF |
| SF | 600.1300095 MHz |
| WDW | QSINE |
| SSB | 0 |
| LB | 0.00 Hz |
| GB | 0 |

# JC−253−2
## C13DEPT135.ucl CDCl3 {V:\Bruker\TOPSPIN} mjp 40

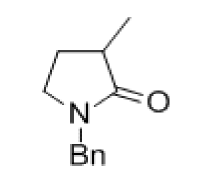

**8a**

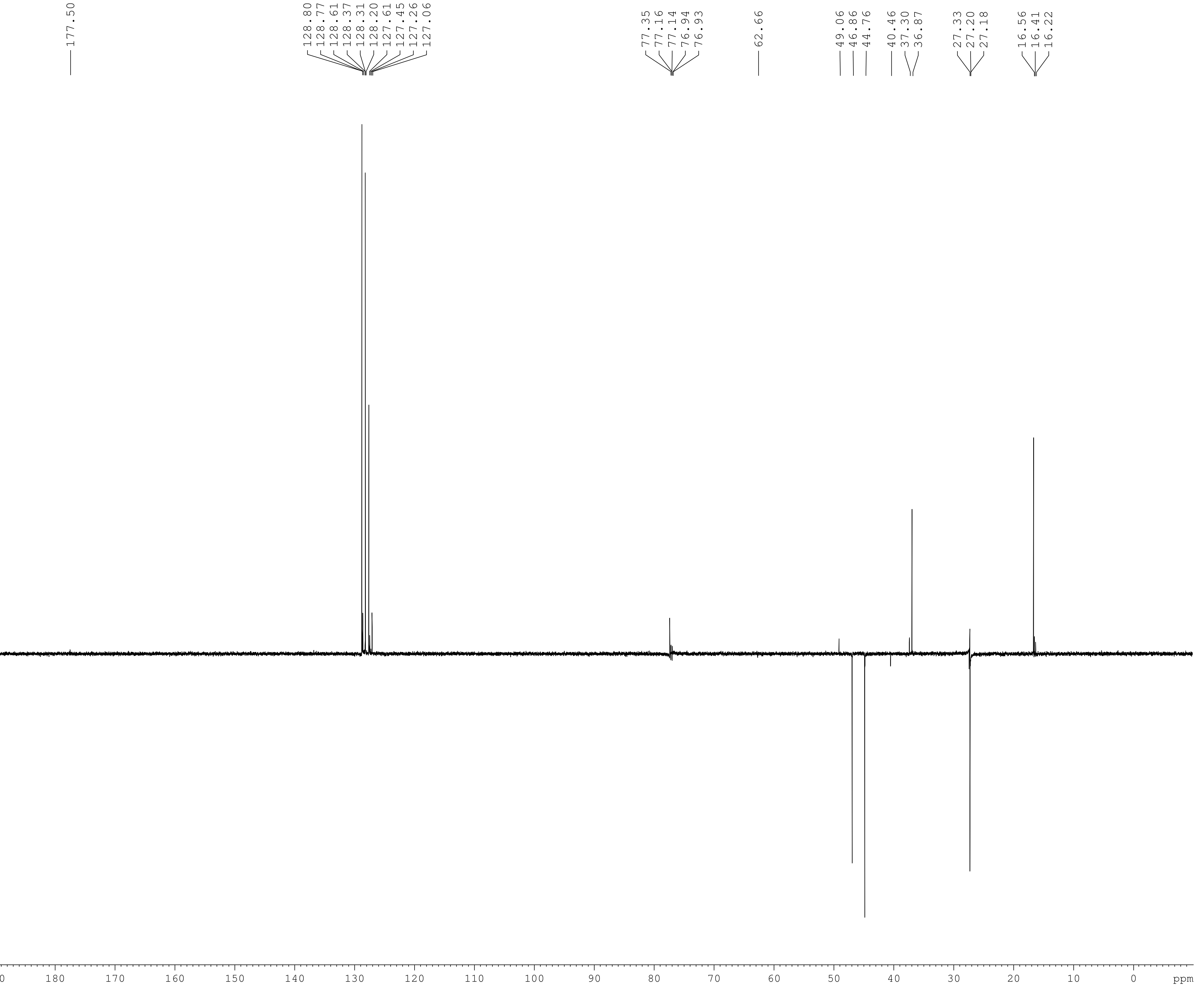


```
NAME               JC-253-2
EXPNO                    15
PROCNO                    1
Date_              20120330
Time                  17.56
INSTRUM               AV600
PROBHD    5 mm CPDCH 13C
PULPROG            dept135
TD                    70308
SOLVENT               CDCl3
NS                       64
DS                        4
SWH             39062.500 H
FIDRES           0.555591 H
AQ              0.8999924 s
RG                      256
DW                 12.800 u
DE                   6.50 u
TE                  298.0 K
CNST2          145.0000000
D1              2.00000000 s
D2              0.00344828 s
D12             0.00002000 s
TD0                       1

======== CHANNEL f1 =====
NUC1                    13C
P1                     9.80 u
P2                    19.60 u
PL1                    5.00 d
PL1W            26.76886177 W
SFO1            150.9201628 M

======== CHANNEL f2 =====
CPDPRG2             waltz16
NUC2                     1H
P3                    10.80 u
P4                    21.60 u
PCPD2                 70.00 u
PL2                    1.00 d
PL12                  17.23 d
PL2W            13.76731014 W
PL12W            0.32798135 W
SFO2            600.1324005 M
SI                    65536
SF              150.9027930 M
WDW                      EM
SSB                       0
LB                     1.00 H
GB                        0
PC                     1.40
```

JC−253−2
HSQC.ucl CDCl3 {V:\Bruker\TOPSPIN} mjp 40

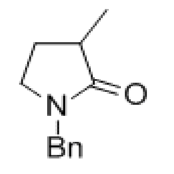


8a

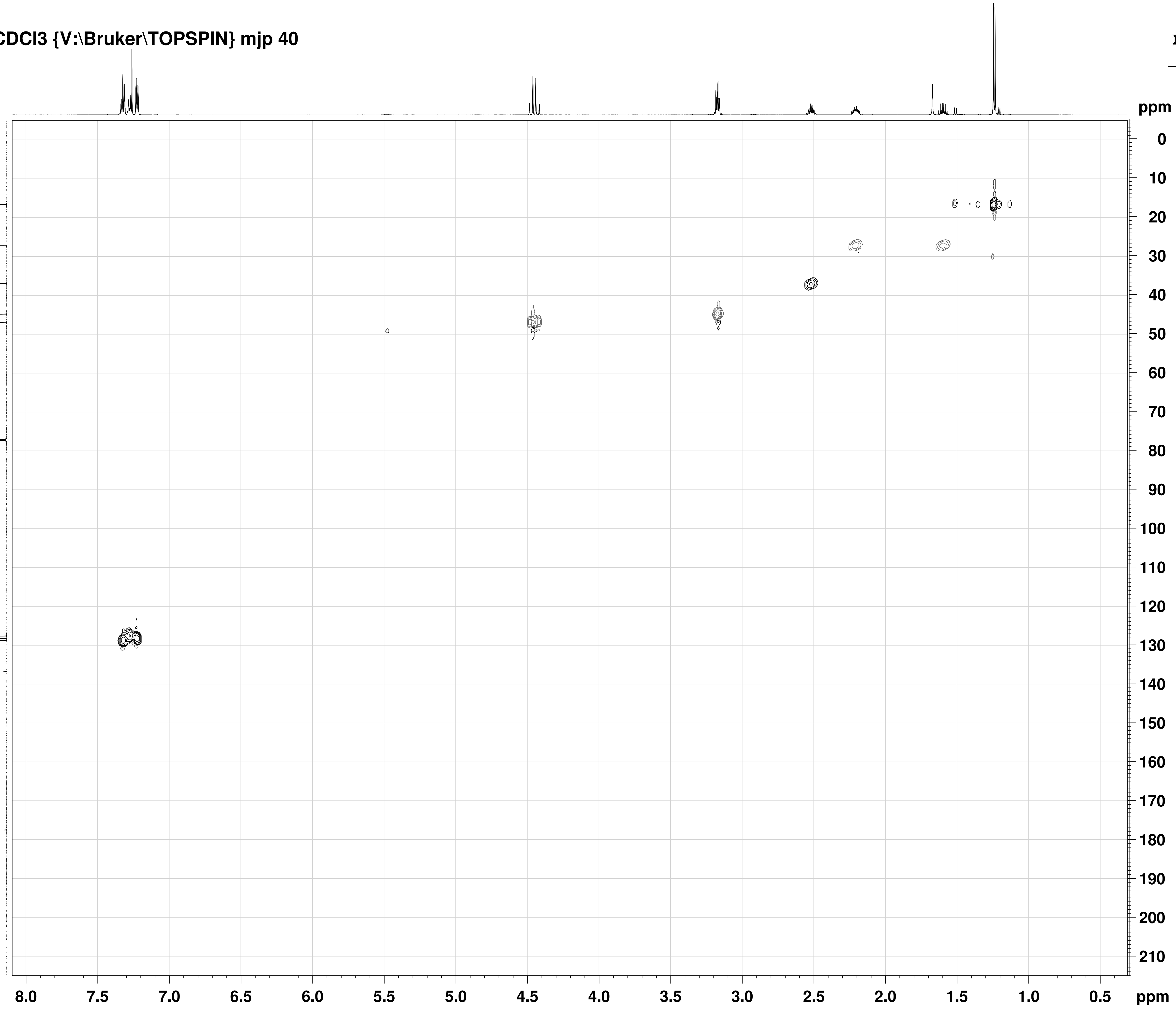


```
NAME             JC-253-2
EXPNO                  13
PROCNO                  1
Date_            20120330
Time                17.34
INSTRUM             AV600
PROBHD   5 mm CPDCH 13C
PULPROG  hsqcedetgpsisp2.4
TD                   1024
SOLVENT             CDCl3
NS                      2
DS                     32
SWH              4672.897 Hz
FIDRES           4.563376 Hz
AQ              0.1096180 sec
RG                   2050
DW                107.000 usec
DE                   6.50 usec
TE                  298.0 K
CNST2         145.0000000
CNST17         -0.5000000
D0             0.00000300 sec
D1             1.47562897 sec
D2             0.00344828 sec
D4             0.00172414 sec
D11            0.03000000 sec
D16            0.00020000 sec
D21            0.00344828 sec
D24            0.00086207 sec
IN0            0.00001505 sec
L0                      0
L31                     1
TD0                     2

======== CHANNEL f1 ========
NUC1                   1H
P1                  11.40 usec
P2                  22.80 usec
P28                  0.00 usec
PL1                  1.00 dB
PL1W          13.76731014 W
SFO1          600.1325341 MHz

======== CHANNEL f2 ========
CPDPRG2  bi_p5m4sp_4sp.2
NUC2                  13C
P3                   9.80 usec
P14                500.00 usec
P24               2000.00 usec
P31               1730.00 usec
P63               1500.00 usec
PL0                120.00 dB
PL2                  5.00 dB
PL12                20.74 dB
PL0W           0.00000000 W
PL2W          26.76886177 W
PL12W          0.71388775 W
SFO2          150.9186538 MHz
SP3                 13.33 dB
SP7                 13.33 dB
SP14                14.82 dB
SP18                18.73 dB
SP31                20.84 dB
SPNAM3   Crp60,0.5,20.1
SPNAM7      Crp60comp.4
SPNAM14  Crp32,1.5,20.2
SPNAM18   Crp60_xfilt.2
SPNAM31  Crp32,1.5,20.2
SPOAL3              0.500
SPOAL7              0.500
SPOAL14             0.500
SPOAL18             0.500
SPOAL31             0.500
SPOFFS3              0.00 Hz
SPOFFS7              0.00 Hz
SPOFFS14             0.00 Hz
SPOFFS18             0.00 Hz
SPOFFS31             0.00 Hz

====== GRADIENT CHANNEL =====
GPNAM1           SINE.100
GPNAM2           SINE.100
GPNAM3           SINE.100
GPNAM4           SINE.100
GPZ1                80.00 %
GPZ2                20.10 %
GPZ3                11.00 %
GPZ4                -5.00 %
P16               1000.00 usec
P19                600.00 usec
ND0                     2
TD                    128
SFO1             150.9187 MHz
FIDRES         259.391449 Hz
SW                220.000 ppm
FnMODE       Echo-Antiecho
SI                   1024
SF            600.1300095 MHz
WDW                 QSINE
SSB                     2
LB                   0.00 Hz
GB                      0
PC                   1.40
SI                   1024
MC2          echo-antiecho
SF            150.9027771 MHz
WDW                 QSINE
SSB                     2
LB                   0.00 Hz
GB                      0
```

JC–253–2
HMBC.ucl CDCl3 {V:\Bruker\TOPSPIN} mjp 40

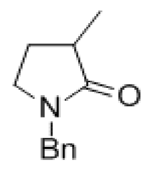


8a

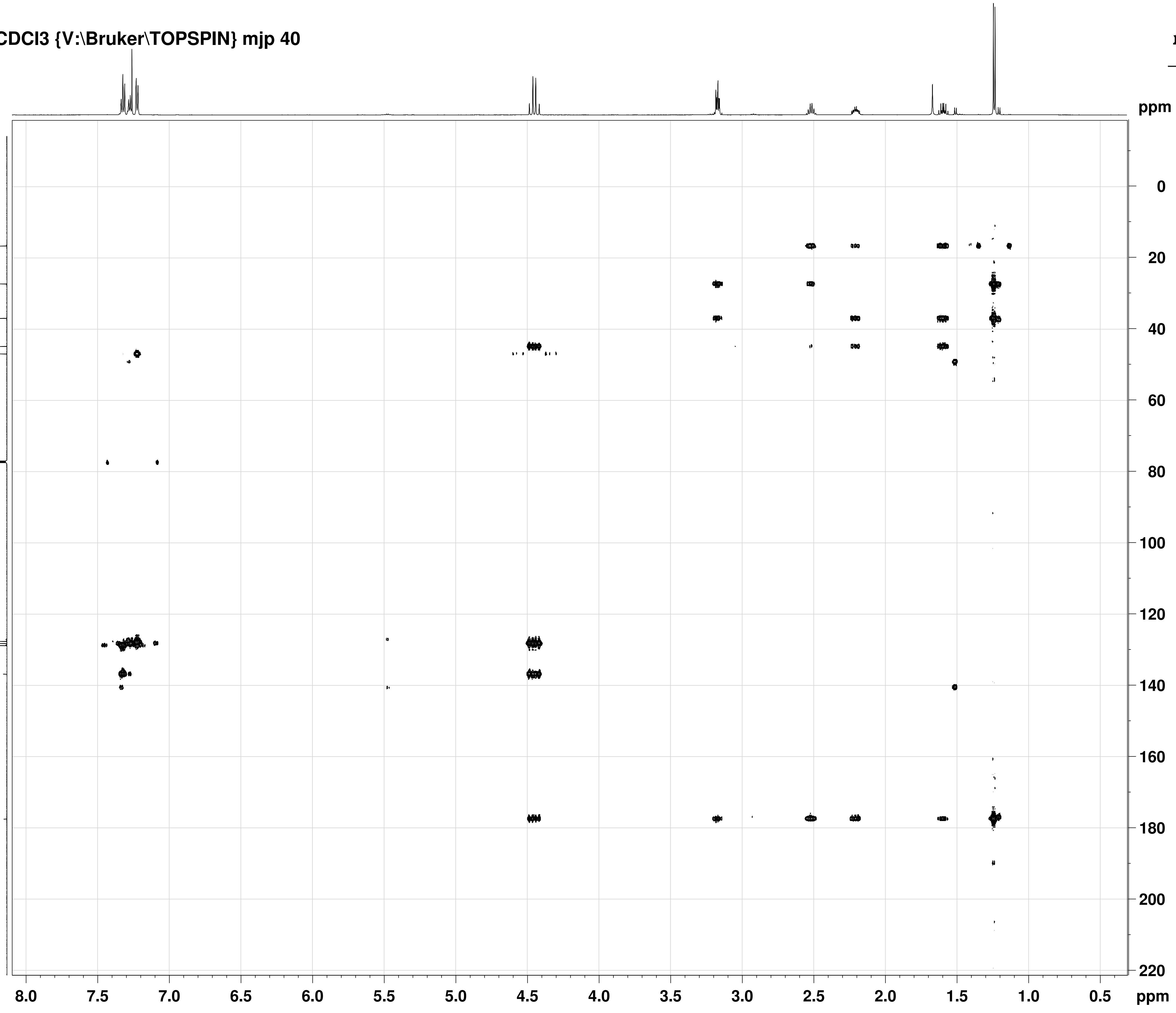


```
NAME              JC-253-2
EXPNO                   14
PROCNO                   1
Date_             20120330
Time                 17.42
INSTRUM              AV600
PROBHD   5 mm CPDCH 13C
PULPROG      hmbcetgpl3nd
TD                    4096
SOLVENT              CDCl3
NS                       2
DS                      16
SWH               4672.897 Hz
FIDRES            1.140844 Hz
AQ               0.4383220 sec
RG                    2050
DW                 107.000 usec
DE                    6.50 usec
TE                   298.0 K
CNST6          120.0000000
CNST7          160.0000000
CNST13          10.0000000
CNST30           0.5981151
D0              0.00000300 sec
D1              0.73952258 sec
D6              0.05000000 sec
D16             0.00020000 sec
IN0             0.00001380 sec

======== CHANNEL f1 ========
NUC1                    1H
P1                   11.40 usec
P2                   22.80 usec
PL1                   1.00 dB
PL1W           13.76731014 W
SFO1           600.1325341 MHz

======== CHANNEL f2 ========
NUC2                   13C
P3                    9.80 usec
P24                2000.00 usec
PL2                   5.00 dB
PL2W           26.76886177 W
SFO2           150.9178993 MHz
SP7                  13.33 dB
SPNAM7         Crp60comp.4
SPOAL7               0.500
SPOFFS7               0.00 Hz

====== GRADIENT CHANNEL =====
GPNAM1            SINE.100
GPNAM3            SINE.100
GPNAM4            SINE.100
GPNAM5            SINE.100
GPNAM6            SINE.100
GPZ1                 80.00 %
GPZ3                 14.00 %
GPZ4                 -8.00 %
GPZ5                 -4.00 %
GPZ6                 -2.00 %
P16                1000.00 usec
ND0                      2
TD                     256
SFO1              150.9179 MHz
FIDRES          141.485535 Hz
SW                 240.000 ppm
FnMODE        Echo-Antiecho
SI                    2048
SF             600.1300106 MHz
WDW                   SINE
SSB                      2
LB                    0.00 Hz
GB                       0
PC                    1.40
SI                    1024
MC2          echo-antiecho
SF             150.9027756 MHz
WDW                   SINE
SSB                      2
LB                    0.00 Hz
GB                       0
```

jc/256/2
PROTON.ucl CDCl3 {C:\Bruker\TOPSPIN} mjp 36

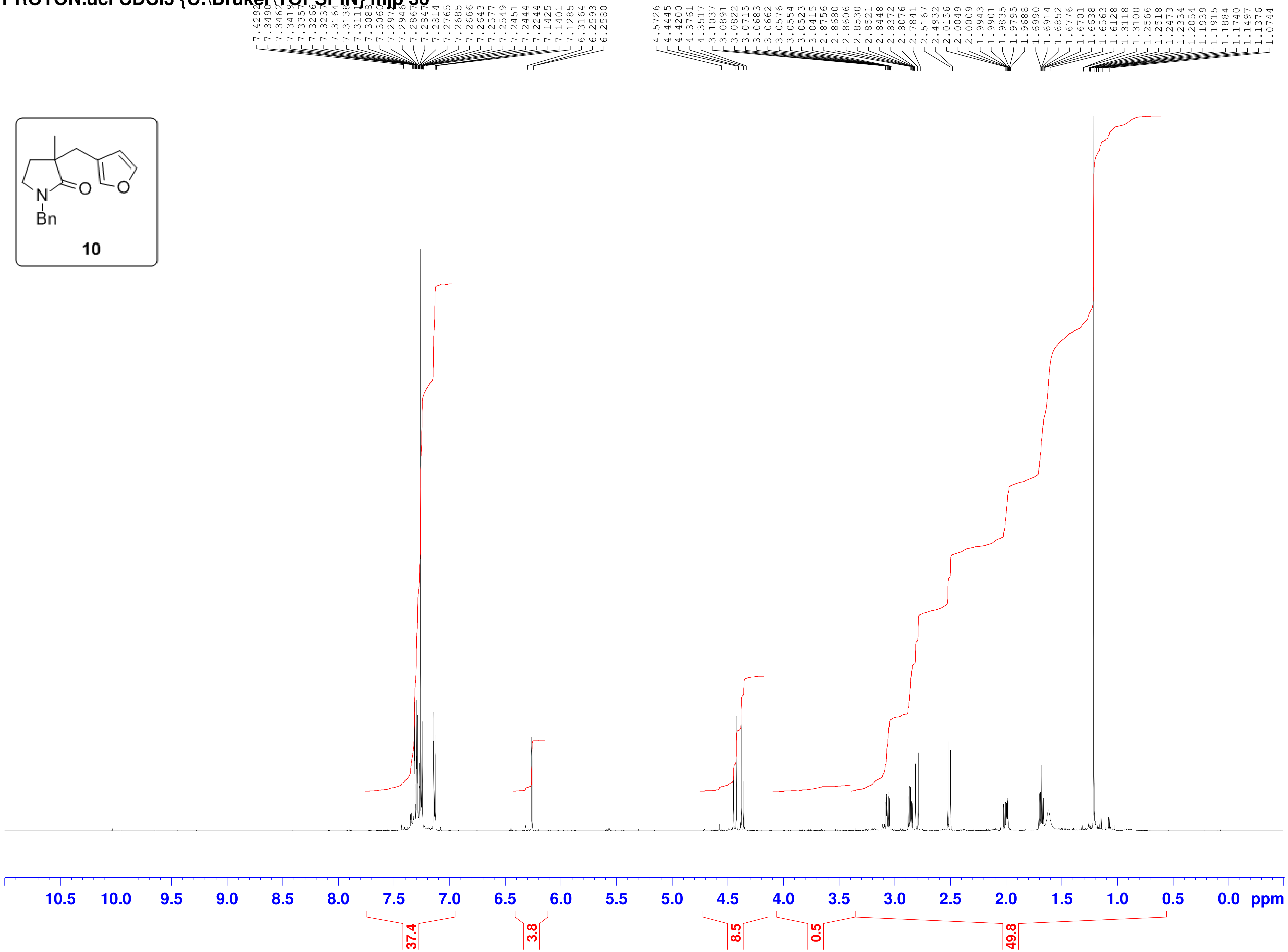

| | |
|---|---|
| NAME | Mar08-2010 |
| EXPNO | 40 |
| PROCNO | 1 |
| Date_ | 20100308 |
| Time | 16.09 |
| INSTRUM | AV600 |
| PROBHD | 5 mm CPDCH 13C |
| PULPROG | zg30 |
| TD | 65536 |
| SOLVENT | CDCl3 |
| NS | 8 |
| DS | 0 |
| SWH | 12335.526 Hz |
| FIDRES | 0.188225 Hz |
| AQ | 2.6564426 sec |
| RG | 32 |
| DW | 40.533 use |
| DE | 10.48 use |
| TE | 298.0 K |
| D1 | 1.00000000 sec |
| TD0 | 1 |

======== CHANNEL f1 ========

| | |
|---|---|
| NUC1 | 1H |
| P1 | 11.40 use |
| PL1 | 1.00 dB |
| PL1W | 13.76731014 W |
| SFO1 | 600.1337061 MHz |
| SI | 32768 |
| SF | 600.1300116 MHz |
| WDW | EM |
| SSB | 0 |
| LB | 0.30 Hz |
| GB | 0 |
| PC | 1.40 |

jc/256/2
C13CPD.ucl CDCl3 {C:\Bruker\TOPSPIN} mjp 36

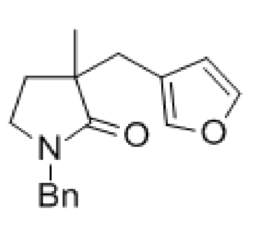

10

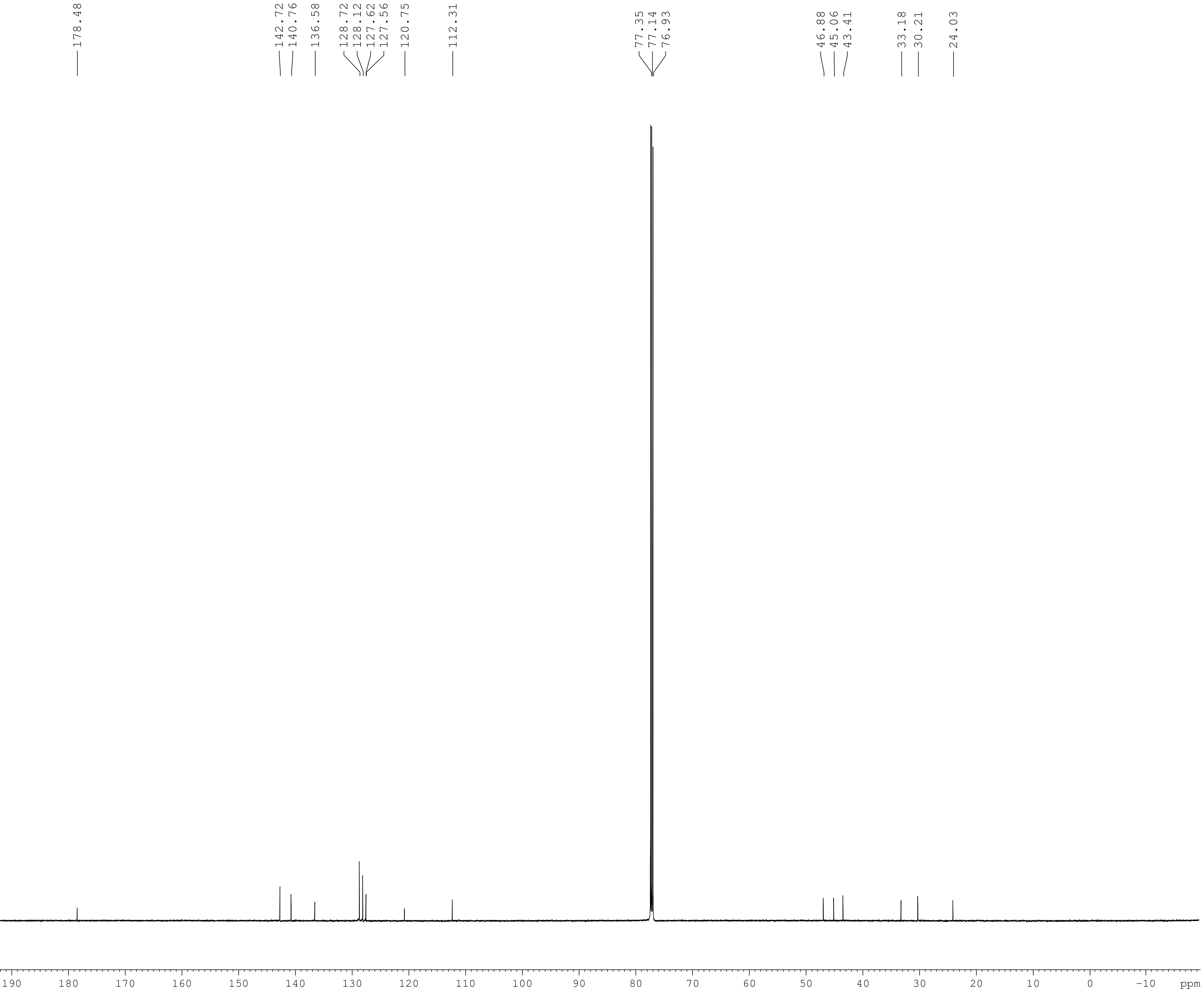


| | |
|---|---|
| NAME | Mar08-2010 |
| EXPNO | 41 |
| PROCNO | 1 |
| Date_ | 20100309 |
| Time | 8.57 |
| INSTRUM | AV600 |
| PROBHD | 5 mm CPDCH 13C |
| PULPROG | zgpg30 |
| TD | 65536 |
| SOLVENT | CDCl3 |
| NS | 128 |
| DS | 0 |
| SWH | 36057.691 Hz |
| FIDRES | 0.550197 Hz |
| AQ | 0.9088159 sec |
| RG | 1030 |
| DW | 13.867 usec |
| DE | 21.55 usec |
| TE | 298.0 K |
| D1 | 2.00000000 sec |
| D11 | 0.03000000 sec |
| TD0 | 1 |
| ======== CHANNEL f1 ===== | |
| NUC1 | 13C |
| P1 | 9.80 usec |
| PL1 | 5.00 dB |
| PL1W | 26.76886177 W |
| SFO1 | 150.9178981 MHz |
| ======== CHANNEL f2 ===== | |
| CPDPRG2 | waltz16 |
| NUC2 | 1H |
| PCPD2 | 70.00 usec |
| PL2 | 1.00 dB |
| PL12 | 16.76 dB |
| PL13 | 120.00 dB |
| PL2W | 13.76731014 W |
| PL12W | 0.36546776 W |
| PL13W | 0.00000000 W |
| SFO2 | 600.1324005 MHz |
| SI | 32768 |
| SF | 150.9027930 MHz |
| WDW | EM |
| SSB | 0 |
| LB | 1.00 Hz |
| GB | 0 |
| PC | 1.40 |

jc/256/1
PROTON.ucl CDCl3 {C:\Bruker\TOPSPIN} mjp 20

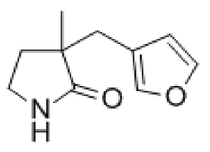

10a

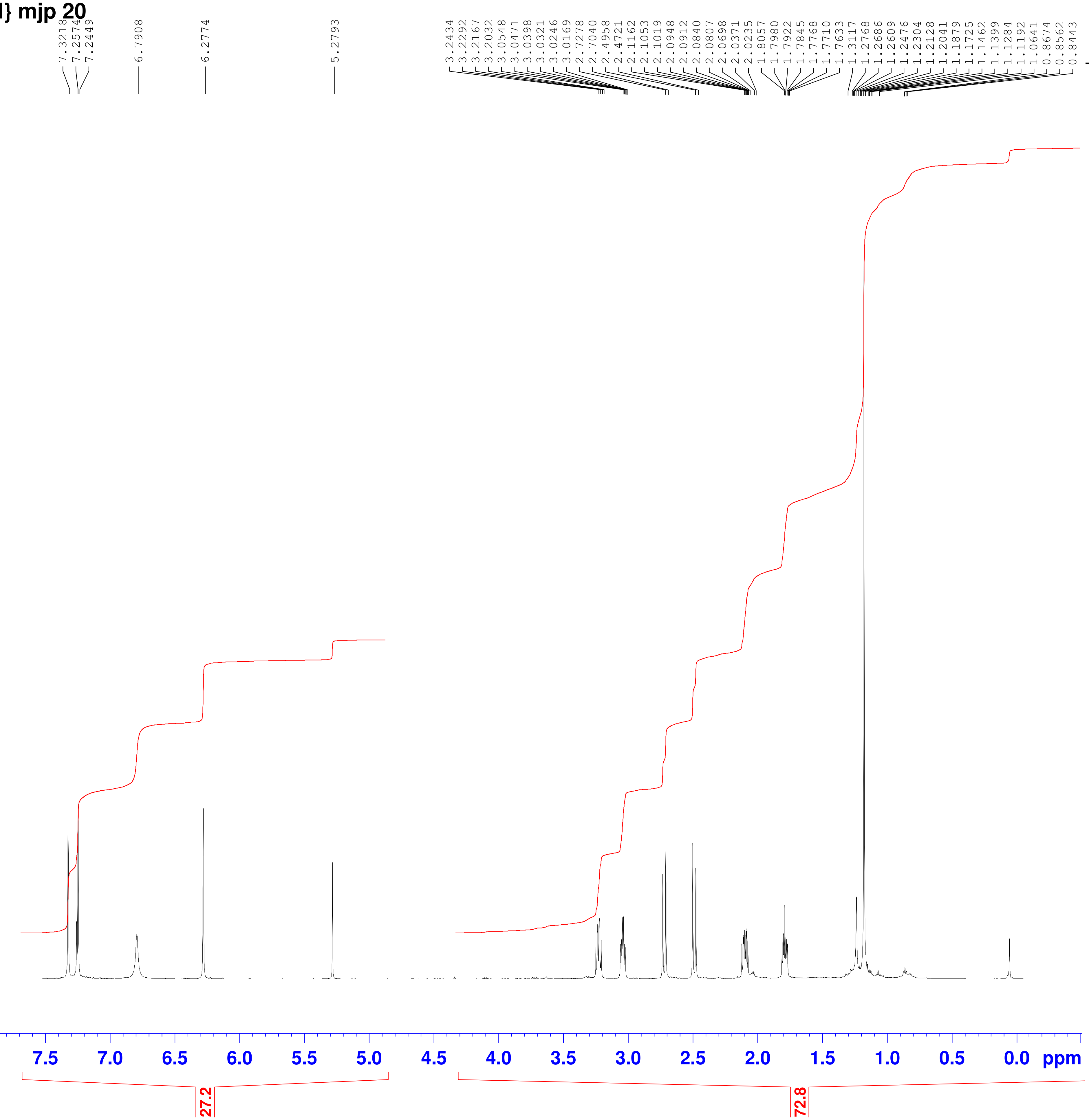


| | |
|---|---|
| NAME | Jan26-2010 |
| EXPNO | 10 |
| PROCNO | 1 |
| Date_ | 20100126 |
| Time | 11.06 |
| INSTRUM | AV600 |
| PROBHD | 5 mm CPDCH 13C |
| PULPROG | zg30 |
| TD | 65536 |
| SOLVENT | CDCl3 |
| NS | 16 |
| DS | 0 |
| SWH | 12335.526 Hz |
| FIDRES | 0.188225 Hz |
| AQ | 2.6564426 sec |
| RG | 18 |
| DW | 40.533 use |
| DE | 10.48 use |
| TE | 298.0 K |
| D1 | 1.00000000 sec |
| TD0 | 1 |

======== CHANNEL f1 ========

| | |
|---|---|
| NUC1 | 1H |
| P1 | 11.40 use |
| PL1 | 1.00 dB |
| PL1W | 13.76731014 W |
| SFO1 | 600.1337061 MHz |
| SI | 32768 |
| SF | 600.1300116 MHz |
| WDW | EM |
| SSB | 0 |
| LB | 0.30 Hz |
| GB | 0 |
| PC | 1.40 |

**jc/256/1**
**C13CPD.ucl CDCl3 {C:\Bruker\TOPSPIN} mjp 20**

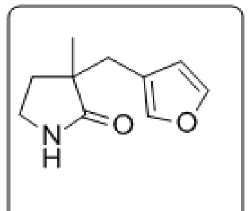

**10a**

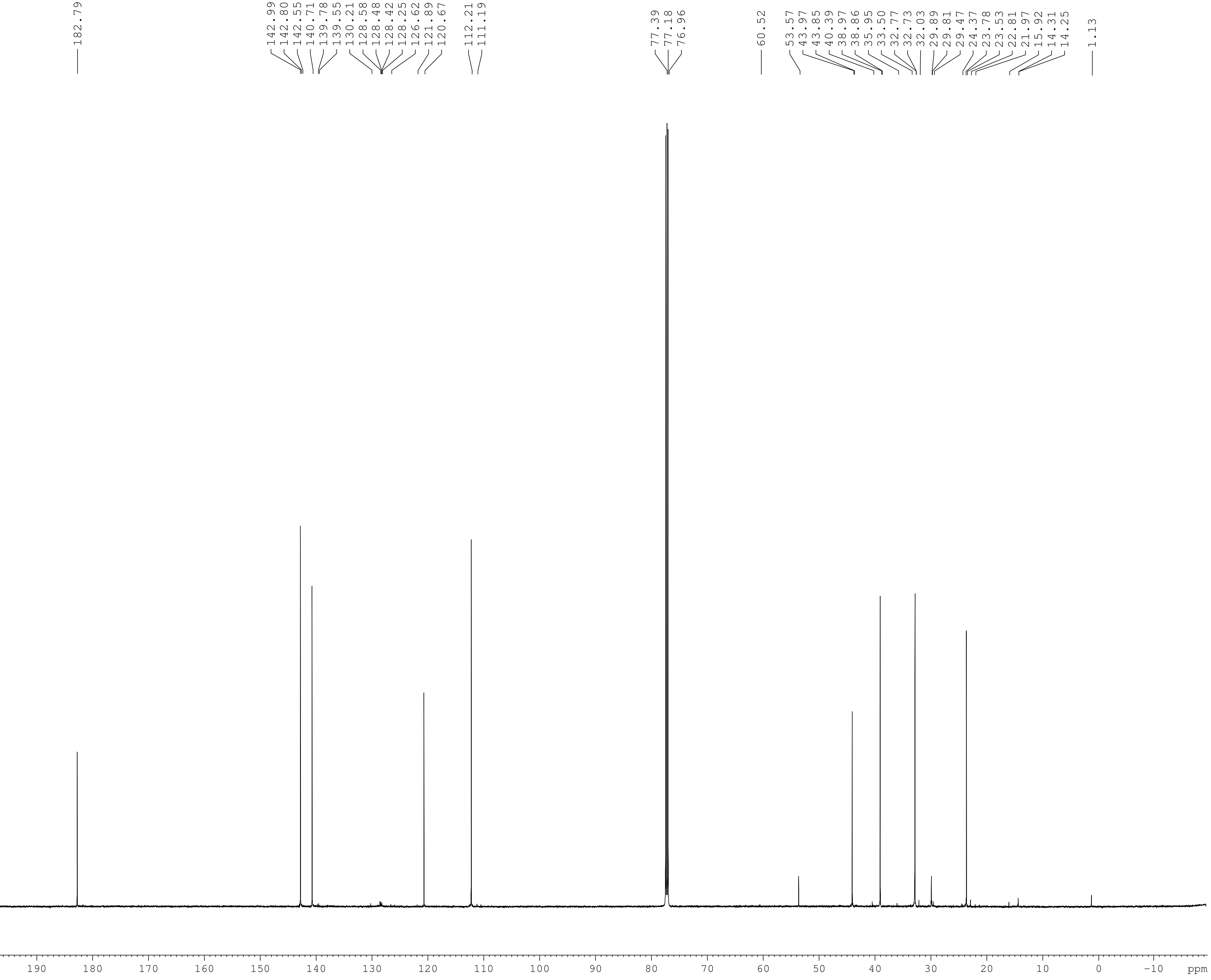


UCL

| | |
|---|---|
| NAME | Jan26-2010 |
| EXPNO | 11 |
| PROCNO | 1 |
| Date_ | 20100126 |
| Time | 11.13 |
| INSTRUM | AV600 |
| PROBHD | 5 mm CPDCH 13C |
| PULPROG | zgpg30 |
| TD | 65536 |
| SOLVENT | CDCl3 |
| NS | 128 |
| DS | 0 |
| SWH | 36057.691 H |
| FIDRES | 0.550197 H |
| AQ | 0.9088159 s |
| RG | 1030 |
| DW | 13.867 u |
| DE | 21.55 u |
| TE | 298.0 K |
| D1 | 2.00000000 s |
| D11 | 0.03000000 s |
| TD0 | 1 |
| ======== CHANNEL f1 ===== | |
| NUC1 | 13C |
| P1 | 9.80 u |
| PL1 | 5.00 d |
| PL1W | 26.76886177 W |
| SFO1 | 150.9178981 M |
| ======== CHANNEL f2 ===== | |
| CPDPRG2 | waltz16 |
| NUC2 | 1H |
| PCPD2 | 70.00 u |
| PL2 | 1.00 d |
| PL12 | 16.76 d |
| PL13 | 120.00 d |
| PL2W | 13.76731014 W |
| PL12W | 0.36546776 W |
| PL13W | 0.00000000 W |
| SFO2 | 600.1324005 M |
| SI | 32768 |
| SF | 150.9027930 M |
| WDW | EM |
| SSB | 0 |
| LB | 1.00 H |
| GB | 0 |
| PC | 1.40 |

jc/256/1
HSQC.ucl CDCl3 {C:\Bruker\TOPSPIN} mjp 20

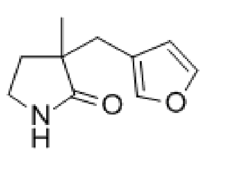

10a

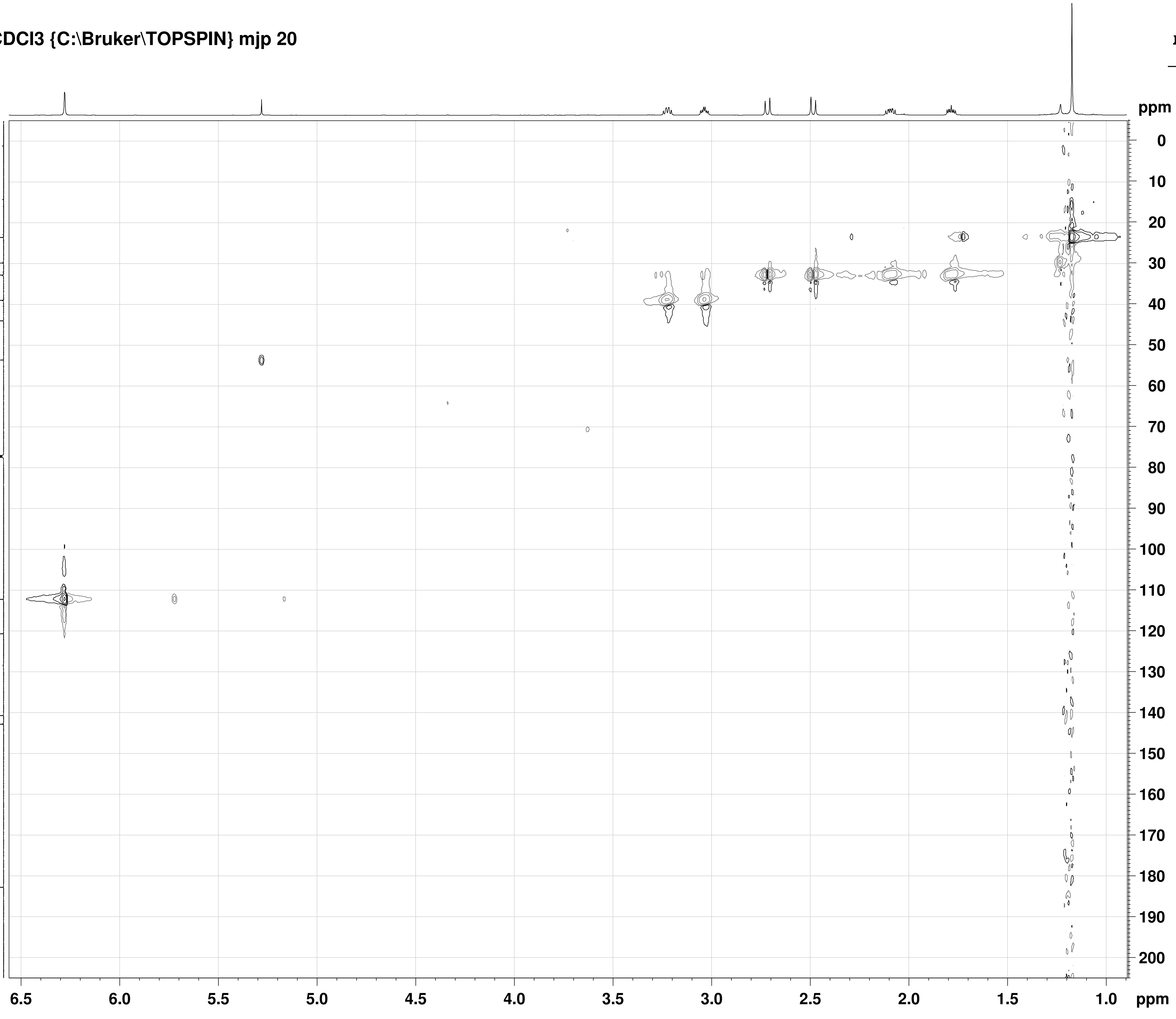


UCL



| | |
|---|---|
| NAME | Jan26-2010 |
| EXPNO | 12 |
| PROCNO | 1 |
| Date_ | 20100126 |
| Time | 11.24 |
| INSTRUM | AV600 |
| PROBHD | 5 mm CPDCH 13C |
| PULPROG | hsqcedetgpsisp2.4 |
| TD | 1024 |
| SOLVENT | CDCl3 |
| NS | 2 |
| DS | 32 |
| SWH | 3401.361 Hz |
| FIDRES | 3.321641 Hz |
| AQ | 0.1505780 sec |
| RG | 2050 |
| DW | 147.000 usec |
| DE | 6.50 usec |
| TE | 298.0 K |
| CNST2 | 145.0000000 |
| CNST17 | -0.5000000 |
| D0 | 0.00000300 sec |
| D1 | 1.43466902 sec |
| D2 | 0.00344828 sec |
| D4 | 0.00172414 sec |
| D11 | 0.03000000 sec |
| D16 | 0.00020000 sec |
| D21 | 0.00344828 sec |
| D24 | 0.00086207 sec |
| IN0 | 0.00001580 sec |
| L0 | 0 |
| L31 | 1 |
| TD0 | 2 |
| ======== CHANNEL f1 ======== | |
| NUC1 | 1H |
| P1 | 11.40 usec |
| P2 | 22.80 usec |
| P28 | 0.00 usec |
| PL1 | 1.00 dB |
| PL1W | 13.76731014 W |
| SFO1 | 600.1322482 MHz |
| ======== CHANNEL f2 ======== | |
| CPDPRG2 | bi_p5m4sp_4sp.2 |
| NUC2 | 13C |
| P3 | 9.80 usec |
| P14 | 500.00 usec |
| P24 | 2000.00 usec |
| P31 | 1730.00 usec |
| P63 | 1500.00 usec |
| PL0 | 120.00 dB |
| PL2 | 5.00 dB |
| PL12 | 20.74 dB |
| PL0W | 0.00000000 W |
| PL2W | 26.76886177 W |
| PL12W | 0.71388775 W |
| SFO2 | 150.9178993 MHz |
| SP3 | 13.33 dB |
| SP7 | 13.33 dB |
| SP14 | 14.82 dB |
| SP18 | 18.73 dB |
| SP31 | 20.84 dB |
| SPNAM3 | Crp60,0.5,20.1 |
| SPNAM7 | Crp60comp.4 |
| SPNAM14 | Crp32,1.5,20.2 |
| SPNAM18 | Crp60_xfilt.2 |
| SPNAM31 | Crp32,1.5,20.2 |
| SPOAL3 | 0.500 |
| SPOAL7 | 0.500 |
| SPOAL14 | 0.500 |
| SPOAL18 | 0.500 |
| SPOAL31 | 0.500 |
| SPOFFS3 | 0.00 Hz |
| SPOFFS7 | 0.00 Hz |
| SPOFFS14 | 0.00 Hz |
| SPOFFS18 | 0.00 Hz |
| SPOFFS31 | 0.00 Hz |
| ====== GRADIENT CHANNEL ===== | |
| GPNAM1 | SINE.100 |
| GPNAM2 | SINE.100 |
| GPNAM3 | SINE.100 |
| GPNAM4 | SINE.100 |
| GPZ1 | 80.00 % |
| GPZ2 | 20.10 % |
| GPZ3 | 11.00 % |
| GPZ4 | -5.00 % |
| P16 | 1000.00 usec |
| P19 | 600.00 usec |
| ND0 | 2 |
| TD | 128 |
| SFO1 | 150.9179 MHz |
| FIDRES | 247.599686 Hz |
| SW | 210.000 ppm |
| FnMODE | Echo-Antiecho |
| SI | 1024 |
| SF | 600.1300101 MHz |
| WDW | QSINE |
| SSB | 2 |
| LB | 0.00 Hz |
| GB | 0 |
| PC | 1.40 |
| SI | 1024 |
| MC2 | echo-antiecho |
| SF | 150.9027781 MHz |
| WDW | QSINE |
| SSB | 2 |
| LB | 0.00 Hz |
| GB | 0 |

Short File Name: jc155g.010.esp

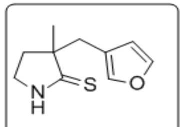

**11**

$^1$H NMR

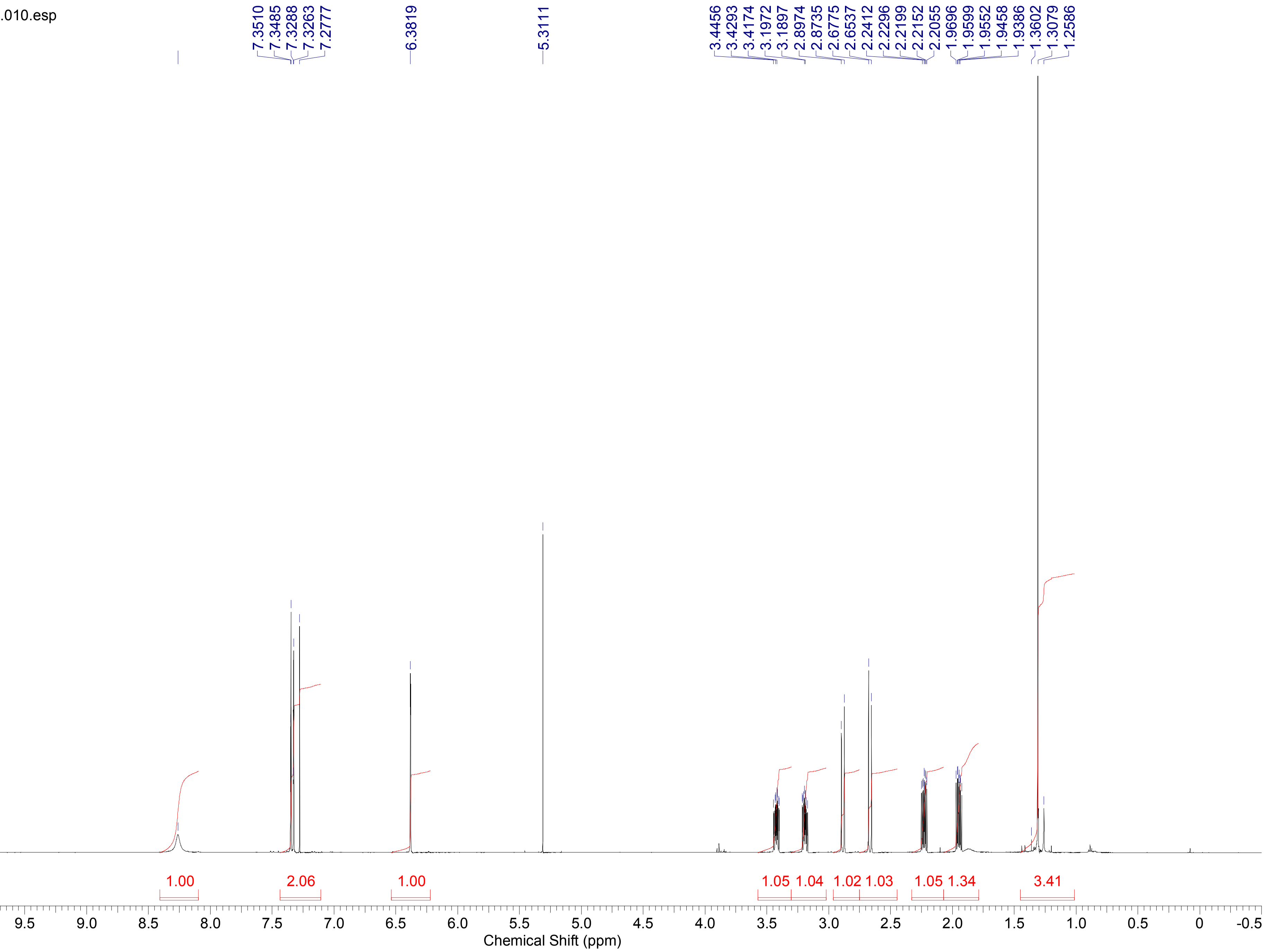

Short File Name: jc155g.011.001.1r.esp

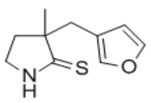

**11**

$^{13}$C NMR

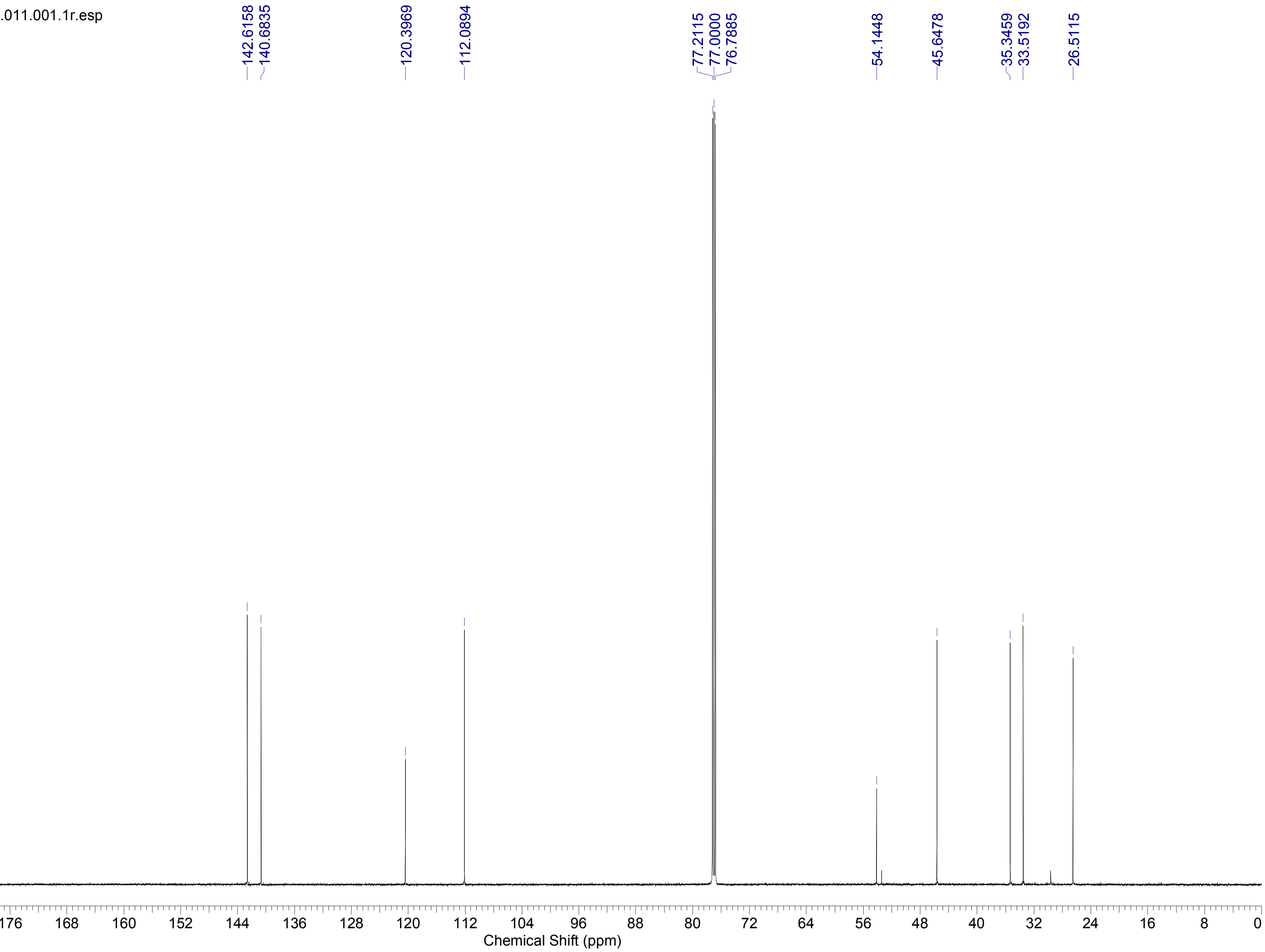

Short File Name: jc155g.013.001.1r.esp

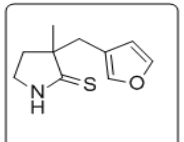

11

DEPT-135

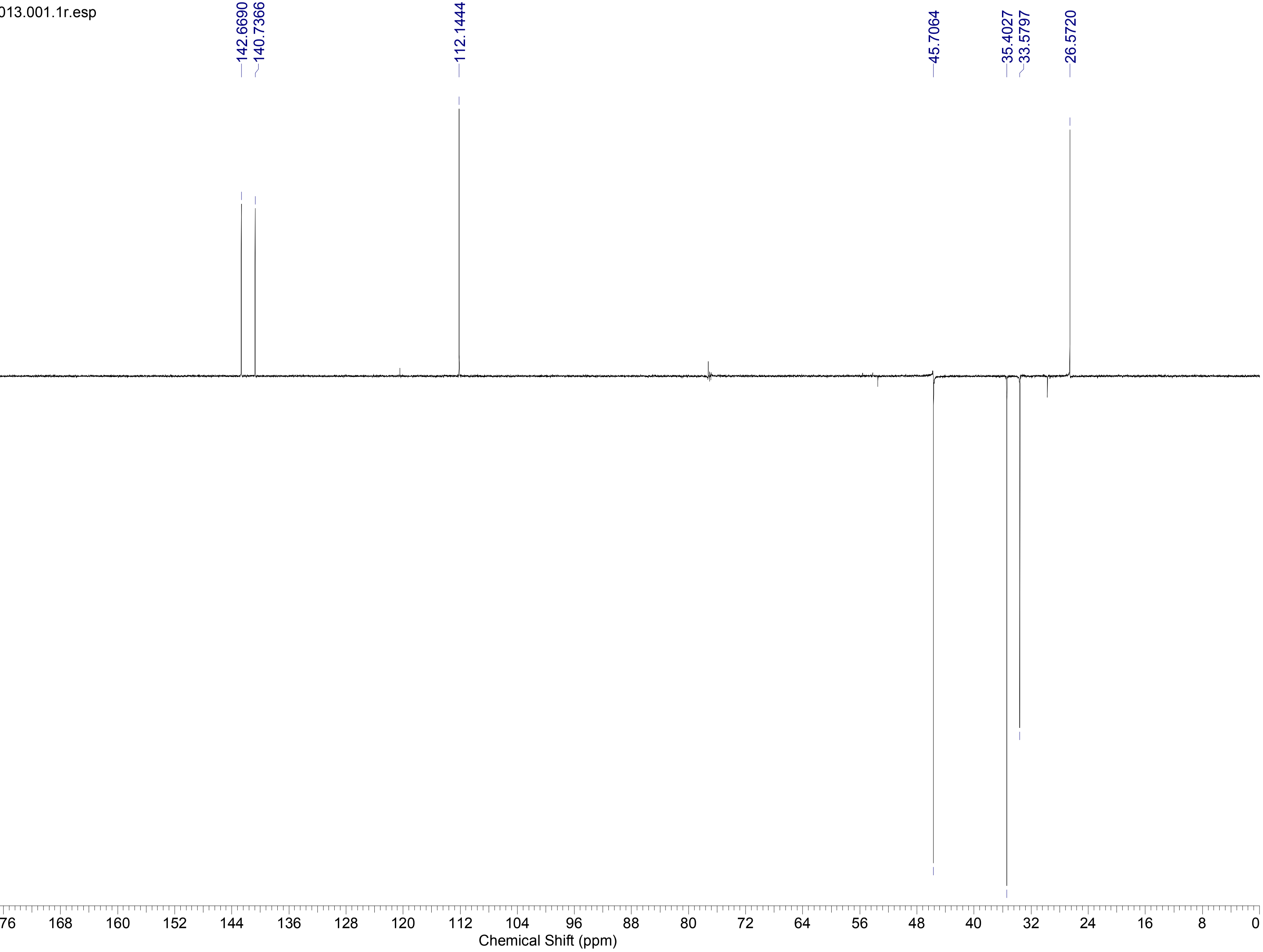

jc/157/2
PROTON.ucl CDCl3 {C:\Bruker\TOPSPIN} mjp 52

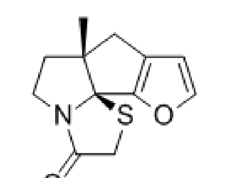


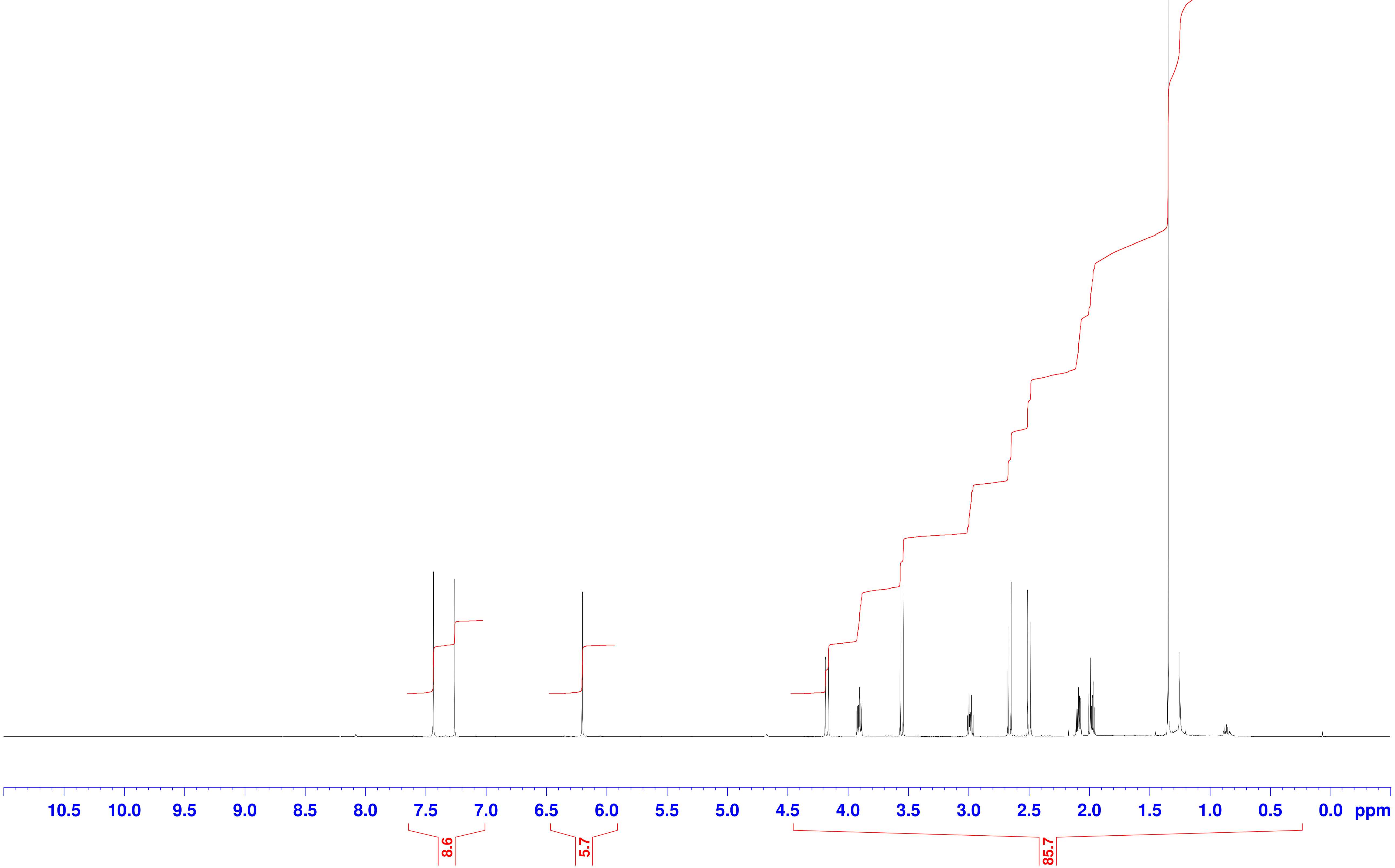


| | | |
|---|---|---|
| NAME | Mar08-2010 | |
| EXPNO | 50 | |
| PROCNO | 1 | |
| Date_ | 20100309 | |
| Time | 11.38 | |
| INSTRUM | AV600 | |
| PROBHD | 5 mm CPDCH 13C | |
| PULPROG | zg30 | |
| TD | 65536 | |
| SOLVENT | CDCl3 | |
| NS | 8 | |
| DS | 0 | |
| SWH | 12335.526 | Hz |
| FIDRES | 0.188225 | Hz |
| AQ | 2.6564426 | sec |
| RG | 28.5 | |
| DW | 40.533 | use |
| DE | 10.48 | use |
| TE | 298.0 | K |
| D1 | 1.00000000 | sec |
| TD0 | 1 | |

======== CHANNEL f1 =======

| | | |
|---|---|---|
| NUC1 | 1H | |
| P1 | 11.40 | use |
| PL1 | 1.00 | dB |
| PL1W | 13.76731014 | W |
| SFO1 | 600.1337061 | MHz |
| SI | 32768 | |
| SF | 600.1300116 | MHz |
| WDW | EM | |
| SSB | 0 | |
| LB | 0.30 | Hz |
| GB | 0 | |
| PC | 1.40 | |

# jc/157/2
# C13CPD.ucl CDCl3 {C:\Bruker\TOPSPIN} mjp 52

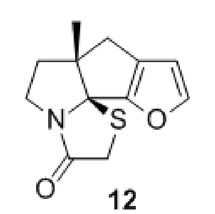

12

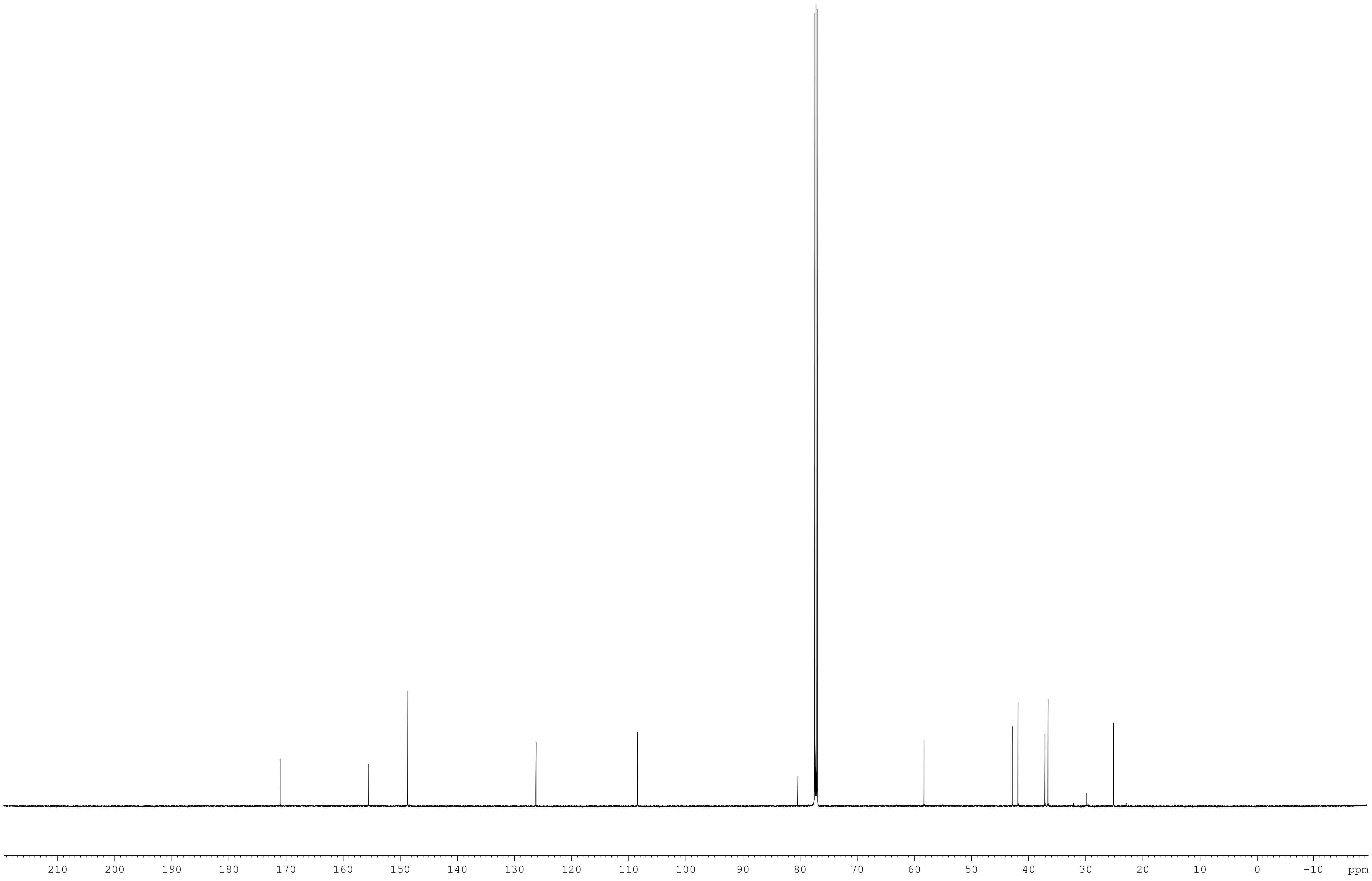


```
NAME          Mar08-2010
EXPNO                 51
PROCNO                 1
Date_           20100309
Time               11.45
INSTRUM            AV600
PROBHD   5 mm CPDCH 13C
PULPROG          zgpg30
TD                 65536
SOLVENT            CDCl3
NS                   128
DS                     0
SWH            36057.691 Hz
FIDRES          0.550197 Hz
AQ             0.9088159 sec
RG                  1030
DW                13.867 usec
DE                 21.55 usec
TE                 298.0 K
D1           2.00000000 sec
D11          0.03000000 sec
TD0                    1

======== CHANNEL f1 ========
NUC1                 13C
P1                  9.80 usec
PL1                 5.00 dB
PL1W         26.76886177 W
SFO1         150.9178981 MHz

======== CHANNEL f2 ========
CPDPRG2          waltz16
NUC2                  1H
PCPD2              70.00 usec
PL2                 1.00 dB
PL12               16.76 dB
PL13              120.00 dB
PL2W         13.76731014 W
PL12W         0.36546776 W
PL13W         0.00000000 W
SFO2         600.1324005 MHz
SI                 32768
SF           150.9027930 MHz
WDW                   EM
SSB                    0
LB                  1.00 Hz
GB                     0
PC                  1.40
```

jc/157/2
COSY.ucl CDCl3 {C:\Bruker\TOPSPIN} mjp 52

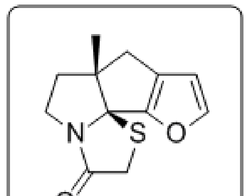
12

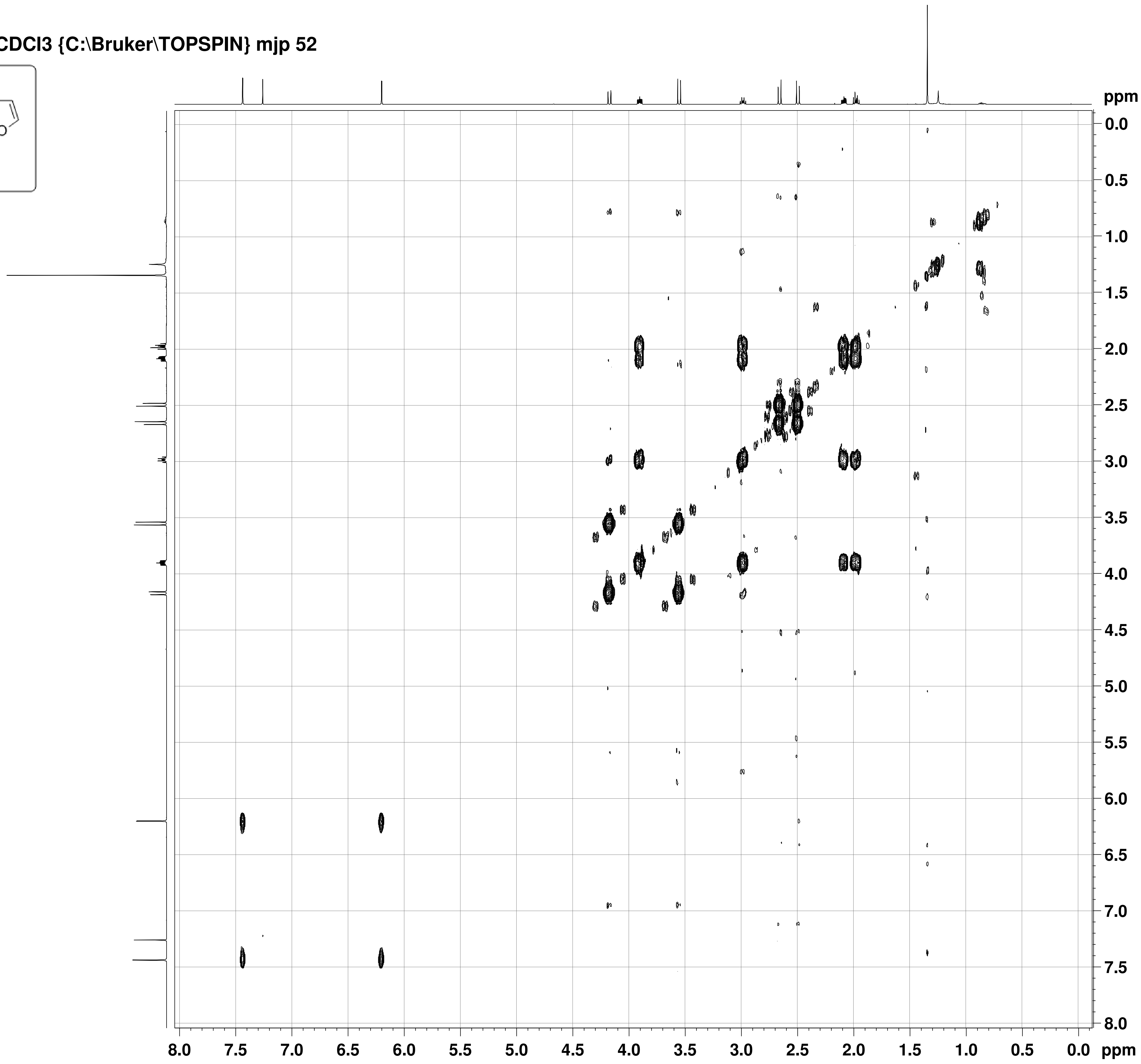


UCL

```
NAME            Mar08-2010
EXPNO                   54
PROCNO                   1
Date_             20100309
Time                 12.27
INSTRUM              AV600
PROBHD   5 mm CPDCH 13C
PULPROG         cosygpmfqf
TD                    2048
SOLVENT              CDCl3
NS                       1
DS                       8
SWH               4901.961 Hz
FIDRES            2.393536 Hz
AQ               0.2089460 sec
RG                    2050
DW                 102.000 usec
DE                    6.50 usec
TE                   298.0 K
D0              0.00000300 sec
D1              1.69116104 sec
D13             0.00000400 sec
D16             0.00020000 sec
IN0             0.00020400 sec

======== CHANNEL f1 ========
NUC1                    1H
P1                   11.40 usec
PL1                   1.00 dB
PL1W           13.76731014 W
SFO1           600.1323875 MHz

====== GRADIENT CHANNEL ====
GPNAM1            SINE.100
GPNAM2            SINE.100
GPNAM3            SINE.100
GPZ1                 16.00 %
GPZ2                 12.00 %
GPZ3                 40.00 %
P16                1000.00 usec
ND0                      1
TD                     128
SFO1              600.1324 MHz
FIDRES           38.296570 Hz
SW                   8.168 ppm
FnMODE                  QF
SI                    1024
SF             600.1300094 MHz
WDW                  QSINE
SSB                      0
LB                    0.00 Hz
GB                       0
PC                    1.40
SI                    1024
MC2                     QF
SF             600.1300094 MHz
WDW                  QSINE
SSB                      0
LB                    0.00 Hz
GB                       0
```

**jc/157/2**
**HSQC.ucl CDCl3 {C:\Bruker\TOPSPIN} mjp 52**

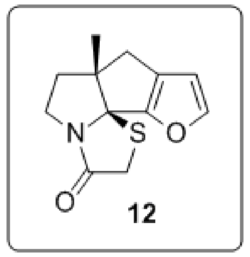
12

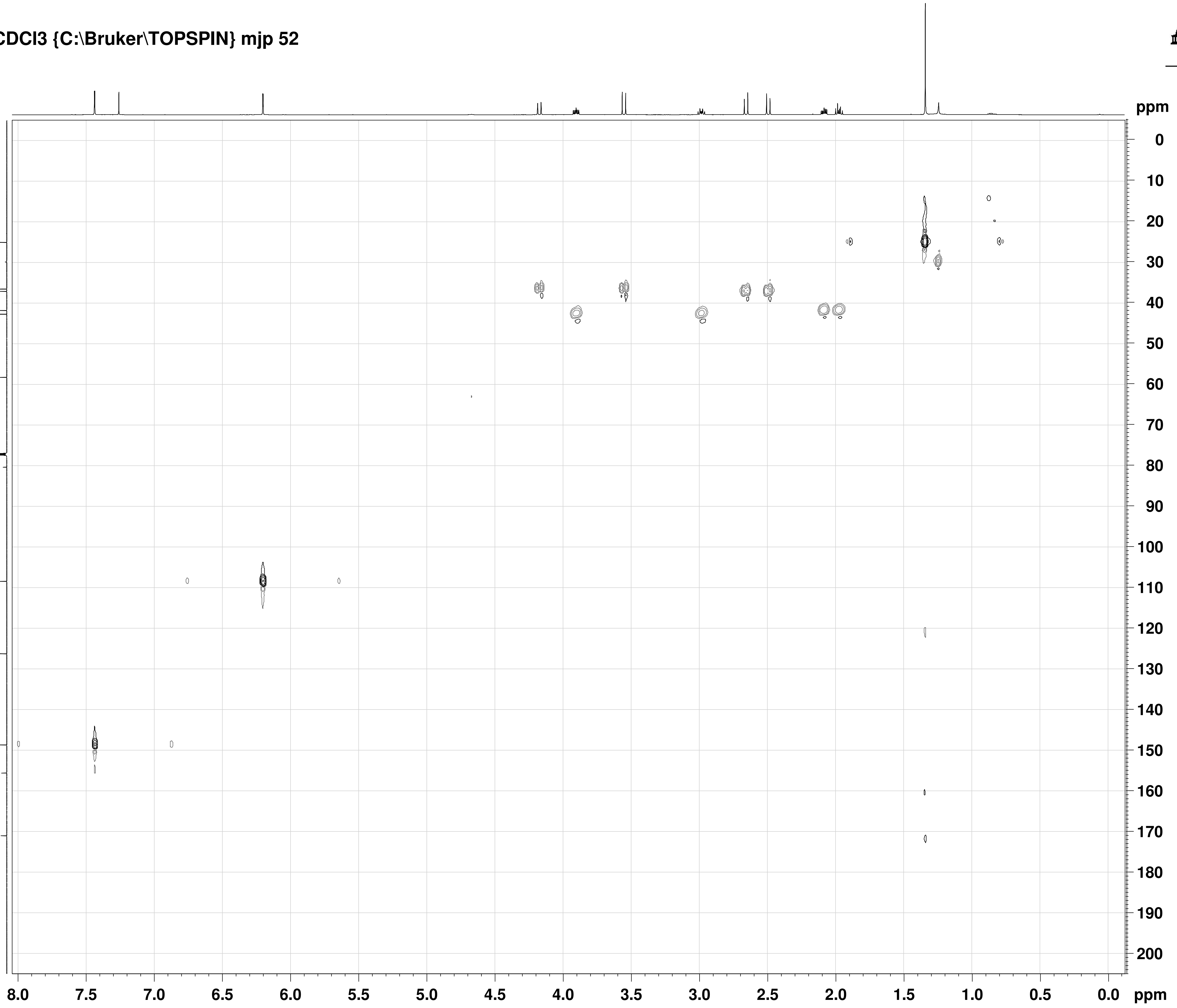


```
NAME           Mar08-2010
EXPNO                  52
PROCNO                  1
Date_            20100309
Time                11.46
INSTRUM             AV600
PROBHD   5 mm CPDCH 13C
PULPROG  hsqcedetgpsisp2.4
TD                   1024
SOLVENT             CDCl3
NS                      2
DS                     32
SWH              4901.961 Hz
FIDRES           4.787071 Hz
AQ              0.1044980 sec
RG                   2050
DW                102.000 usec
DE                   6.50 usec
TE                  298.0 K
CNST2         145.0000000
CNST17         -0.5000000
D0             0.00000300 sec
D1             1.48074901 sec
D2             0.00344828 sec
D4             0.00172414 sec
D11            0.03000000 sec
D16            0.00020000 sec
D21            0.00344828 sec
D24            0.00086207 sec
IN0            0.00001580 sec
L0                      0
L31                     1
TD0                     2

======== CHANNEL f1 ========
NUC1                   1H
P1                  11.40 usec
P2                  22.80 usec
P28                  0.00 usec
PL1                  1.00 dB
PL1W          13.76731014 W
SFO1          600.1323875 MHz

======== CHANNEL f2 ========
CPDPRG2    bi_p5m4sp_4sp.2
NUC2                  13C
P3                   9.80 usec
P14                500.00 usec
P24               2000.00 usec
P31               1730.00 usec
P63               1500.00 usec
PL0                120.00 dB
PL2                  5.00 dB
PL12                20.74 dB
PL0W           0.00000000 W
PL2W          26.76886177 W
PL12W          0.71388775 W
SFO2          150.9178993 MHz
SP3                 13.33 dB
SP7                 13.33 dB
SP14                14.82 dB
SP18                18.73 dB
SP31                20.84 dB
SPNAM3    Crp60,0.5,20.1
SPNAM7       Crp60comp.4
SPNAM14   Crp32,1.5,20.2
SPNAM18    Crp60_xfilt.2
SPNAM31   Crp32,1.5,20.2
SPOAL3              0.500
SPOAL7              0.500
SPOAL14             0.500
SPOAL18             0.500
SPOAL31             0.500
SPOFFS3              0.00 Hz
SPOFFS7              0.00 Hz
SPOFFS14             0.00 Hz
SPOFFS18             0.00 Hz
SPOFFS31             0.00 Hz

====== GRADIENT CHANNEL =====
GPNAM1           SINE.100
GPNAM2           SINE.100
GPNAM3           SINE.100
GPNAM4           SINE.100
GPZ1                80.00 %
GPZ2                20.10 %
GPZ3                11.00 %
GPZ4                -5.00 %
P16               1000.00 usec
P19                600.00 usec
ND0                     2
TD                    128
SFO1             150.9179 MHz
FIDRES         247.599686 Hz
SW                210.000 ppm
FnMODE       Echo-Antiecho
SI                   1024
SF            600.1300094 MHz
WDW                 QSINE
SSB                     2
LB                   0.00 Hz
GB                      0
PC                   1.40
SI                   1024
MC2          echo-antiecho
SF            150.9027781 MHz
WDW                 QSINE
SSB                     2
LB                   0.00 Hz
GB                      0
```

**jc/157/2**
**HMBC.ucl CDCl3 {C:\Bruker\TOPSPIN} mjp 52**

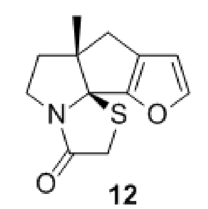

12

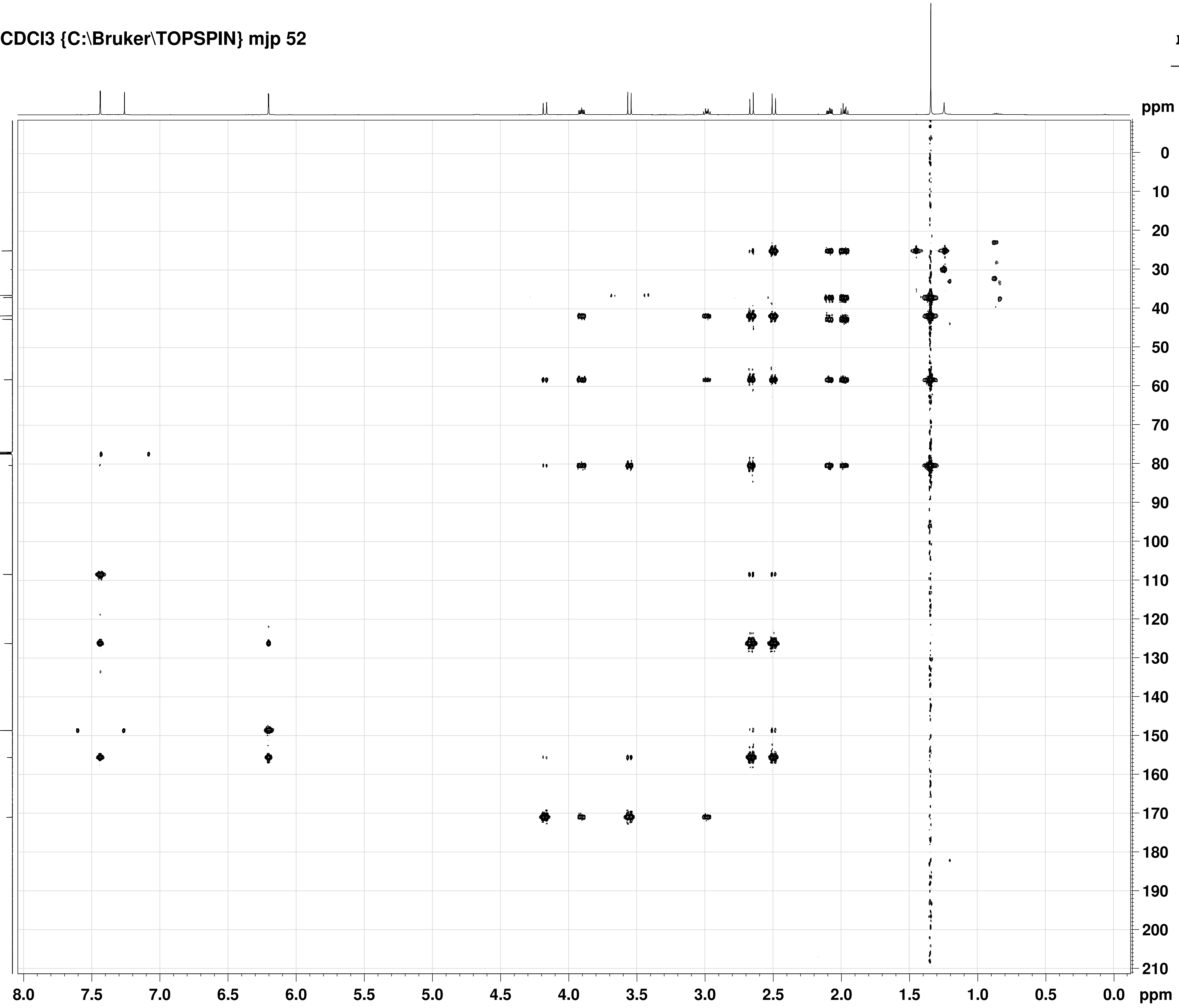


```
NAME             Mar08-2010
EXPNO                    53
PROCNO                    1
Date_              20100309
Time                  11.54
INSTRUM               AV600
PROBHD   5 mm CPDCH 13C
PULPROG      hmbcetgpl3nd
TD                     4096
SOLVENT               CDCl3
NS                        2
DS                       16
SWH              4901.961 Hz
FIDRES           1.196768 Hz
AQ              0.4178420 sec
RG                     2050
DW                  102.000 usec
DE                     6.50 usec
TE                    298.0 K
CNST6           120.0000000
CNST7           160.0000000
CNST13           10.0000000
CNST30            0.5981151
D0               0.00000300 sec
D1               0.76000261 sec
D6               0.05000000 sec
D16              0.00020000 sec
IN0              0.00001505 sec

======== CHANNEL f1 ========
NUC1                     1H
P1                    11.40 usec
P2                    22.80 usec
PL1                    1.00 dB
PL1W            13.76731014 W
SFO1            600.1323875 MHz

======== CHANNEL f2 ========
NUC2                    13C
P3                     9.80 usec
P24                 2000.00 usec
PL2                    5.00 dB
PL2W            26.76886177 W
SFO2            150.9178993 MHz
SP7                   13.33 dB
SPNAM7          Crp60comp.4
SPOAL7                0.500
SPOFFS7                0.00 Hz

====== GRADIENT CHANNEL =====
GPNAM1             SINE.100
GPNAM3             SINE.100
GPNAM4             SINE.100
GPNAM5             SINE.100
GPNAM6             SINE.100
GPZ1                  80.00 %
GPZ3                  14.00 %
GPZ4                  -8.00 %
GPZ5                  -4.00 %
GPZ6                  -2.00 %
P16                 1000.00 usec
ND0                       2
TD                      256
SFO1               150.9179 MHz
FIDRES           129.695068 Hz
SW                  220.000 ppm
FnMODE         Echo-Antiecho
SI                     2048
SF              600.1300106 MHz
WDW                    SINE
SSB                       2
LB                     0.00 Hz
GB                        0
PC                     1.40
SI                     1024
MC2           echo-antiecho
SF              150.9027773 MHz
WDW                    SINE
SSB                       2
LB                     0.00 Hz
GB                        0
```

**JC–160–2**
**PROTON.ucl CDCl3 {V:\Bruker\TOPSPIN} mjp 52**

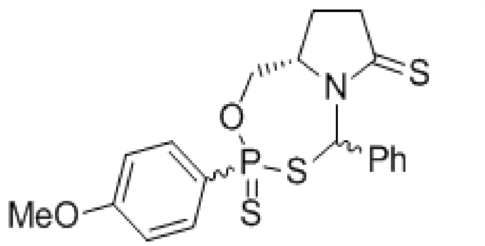


**14**

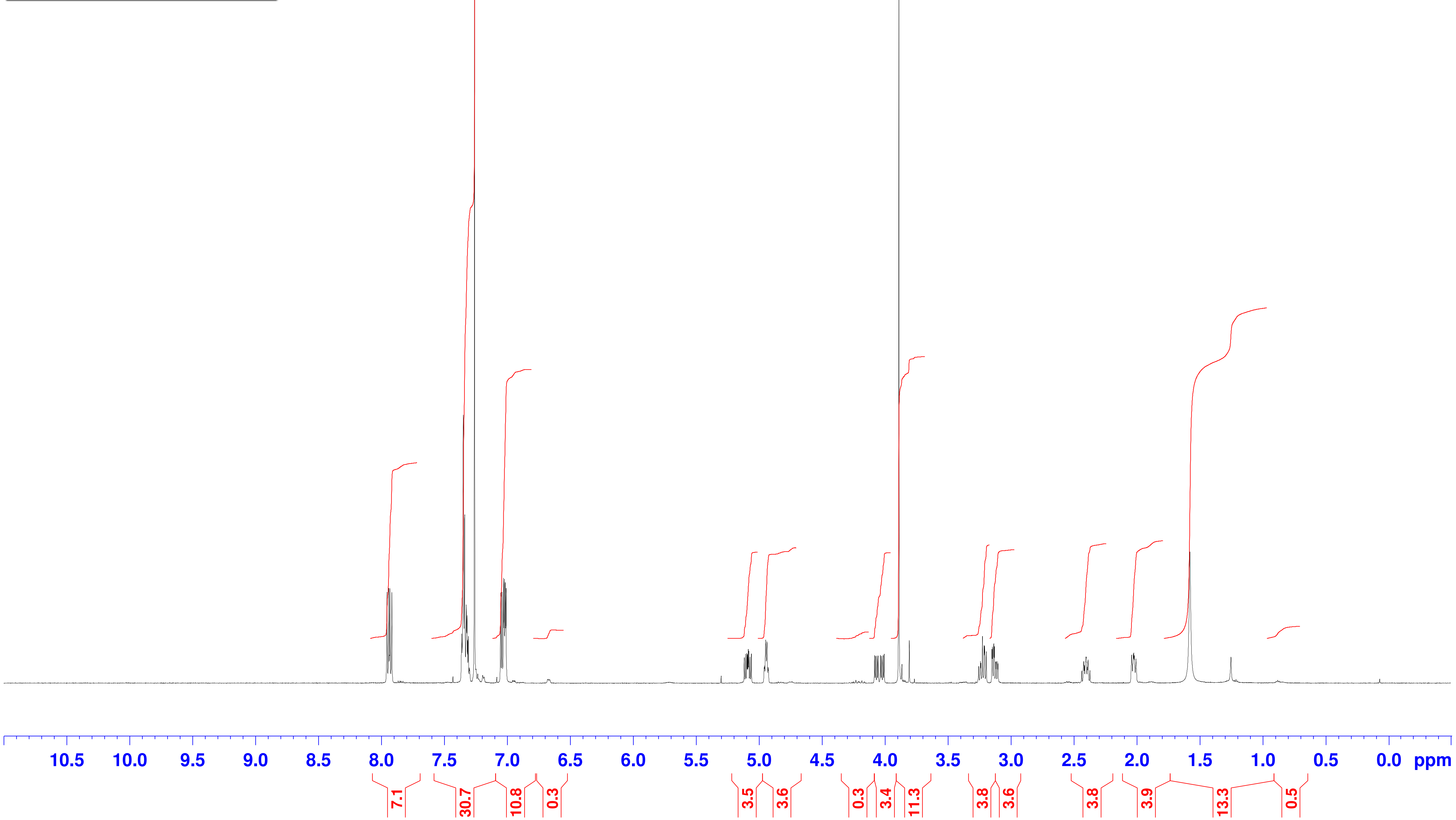


UCL

| | |
|---|---|
| NAME | JC-160-2 |
| EXPNO | 10 |
| PROCNO | 1 |
| Date_ | 20120330 |
| Time | 16.24 |
| INSTRUM | AV600 |
| PROBHD | 5 mm CPDCH 13C |
| PULPROG | zg30 |
| TD | 98682 |
| SOLVENT | CDCl3 |
| NS | 8 |
| DS | 0 |
| SWH | 12335.526 Hz |
| FIDRES | 0.125003 Hz |
| AQ | 3.9999604 sec |
| RG | 32 |
| DW | 40.533 use |
| DE | 10.48 use |
| TE | 298.0 K |
| D1 | 1.00000000 sec |
| TD0 | 1 |

======== CHANNEL f1 ========

| | |
|---|---|
| NUC1 | 1H |
| P1 | 11.40 use |
| PL1 | 1.00 dB |
| PL1W | 13.76731014 W |
| SFO1 | 600.1337061 MHz |
| SI | 32768 |
| SF | 600.1300116 MHz |
| WDW | EM |
| SSB | 0 |
| LB | 0.30 Hz |
| GB | 0 |
| PC | 1.40 |

V:BrukerTOPSPIN/mjp/JC-160-2/10/1
Integral regions #5 to #9

JC-160-2
PROTON.ucl CDCl3 {V:\Bruker\TOPSPIN} mjp 52

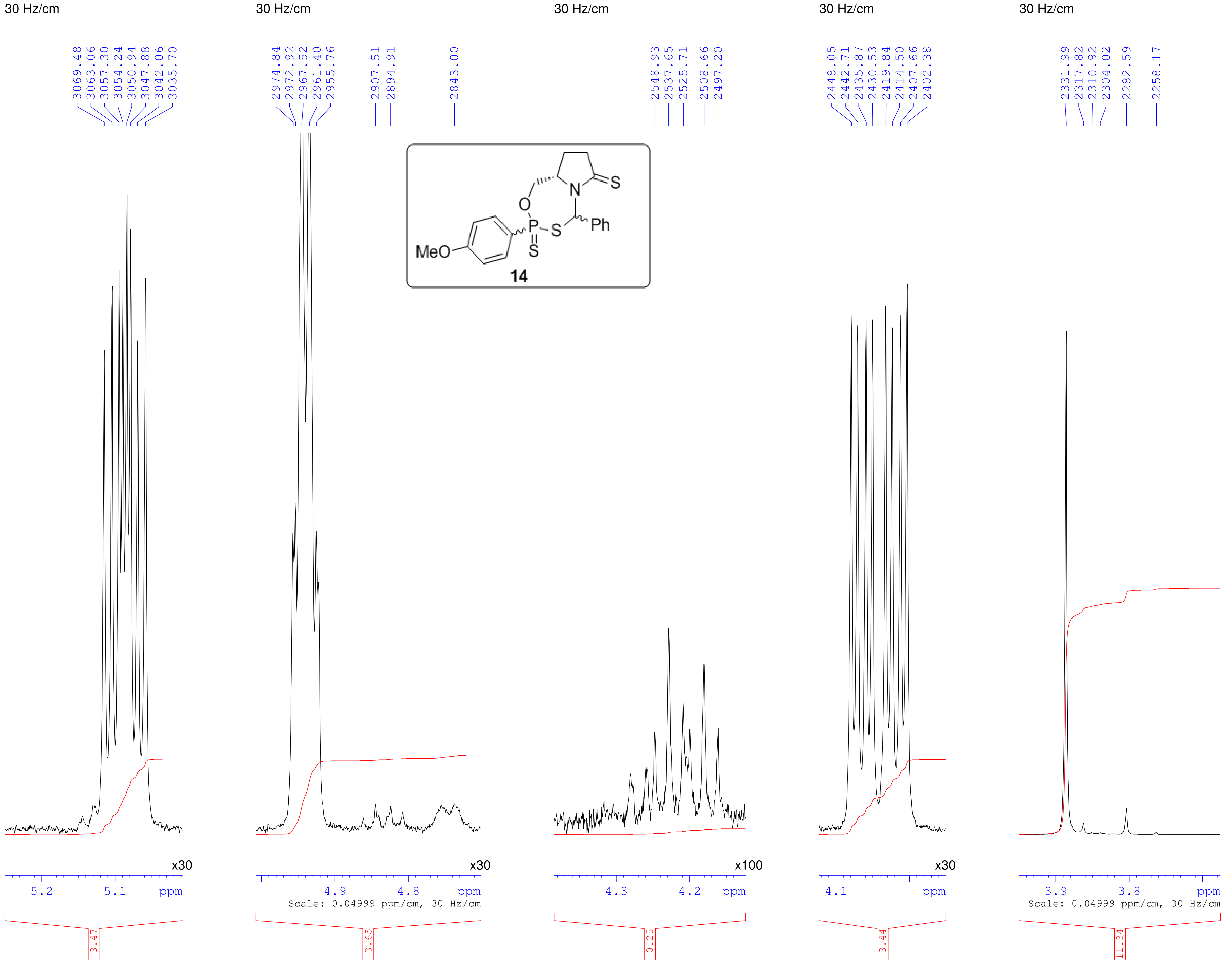

V:BrukerTOPSPIN/mjp/JC−160−2/10/1
Integral regions #10 to #13

JC−160−2
PROTON.ucl CDCl3 {V:\Bruker\TOPSPIN} mjp 52

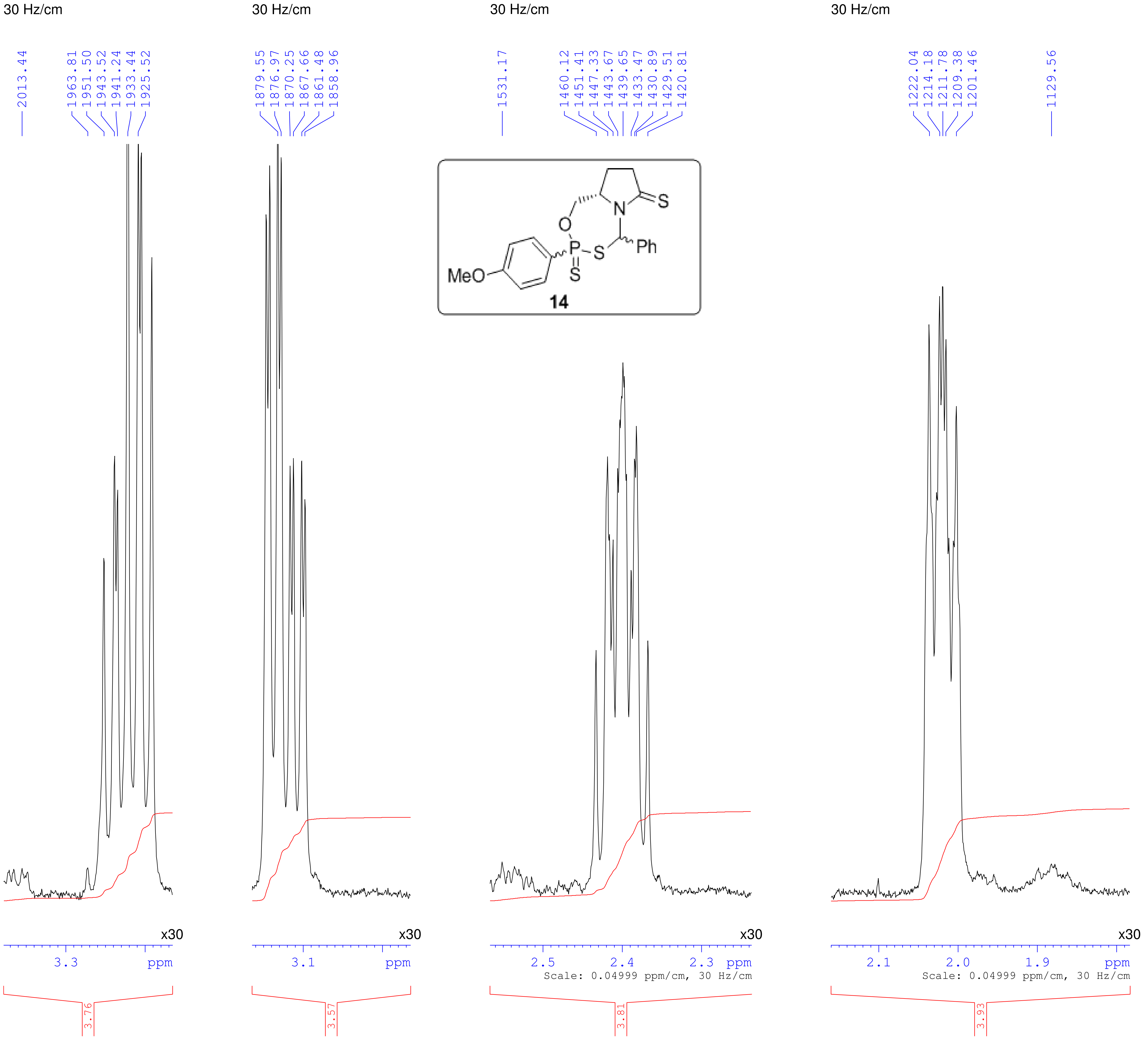

**JC−160−2**
**C13CPD.ucl CDCl3 {V:\Bruker\TOPSPIN} mjp 52**

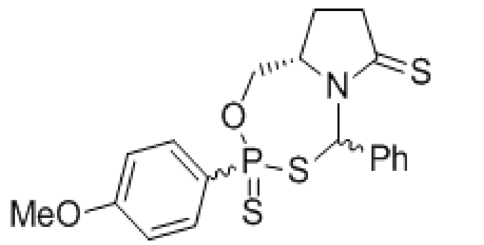


**14**

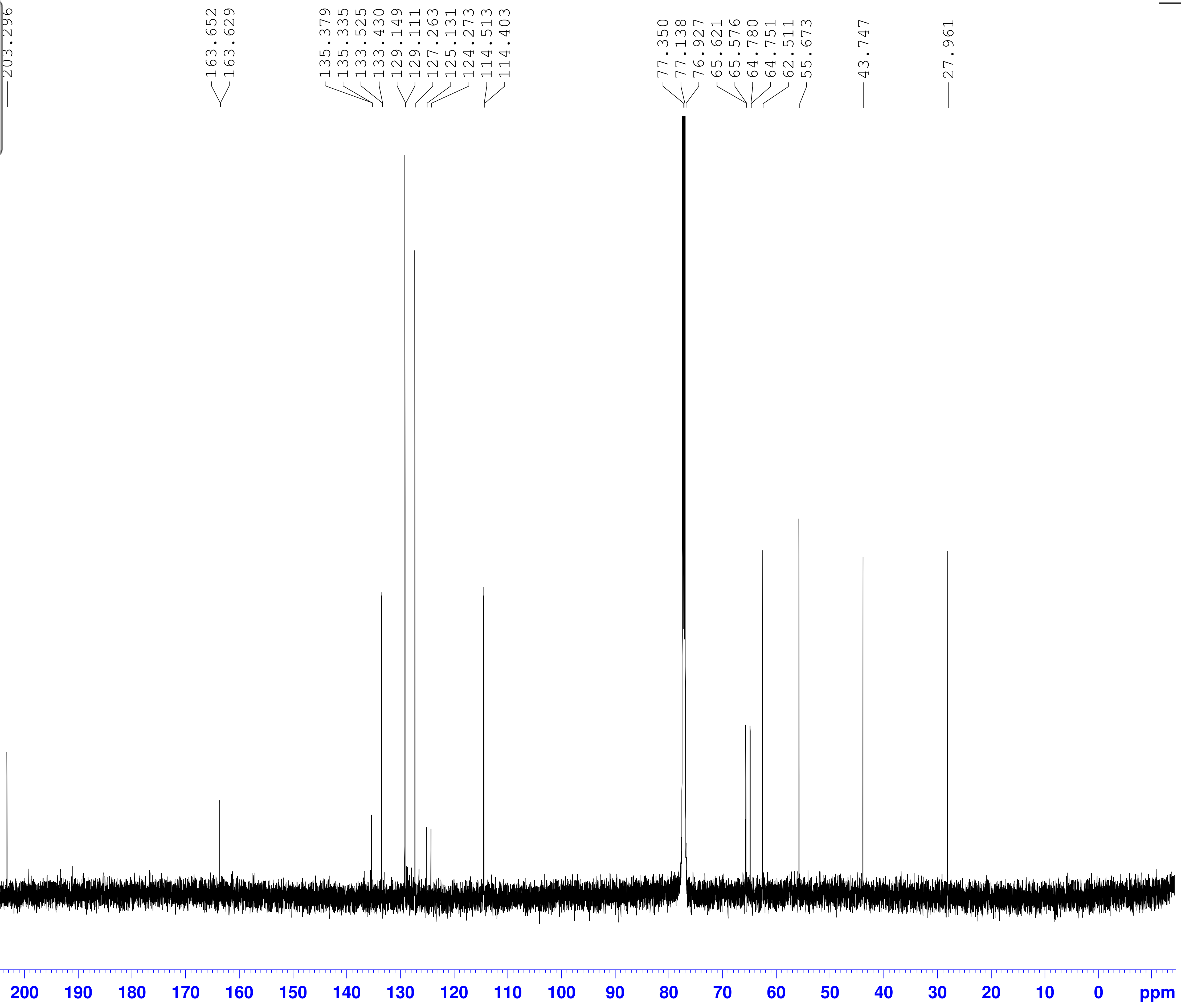


```
NAME               JC-160-2
EXPNO                    12
PROCNO                    1
Date_              20120330
Time                  18.06
INSTRUM               AV600
PROBHD   5 mm CPDCH 13C
PULPROG             zgpg30
TD                    70308
SOLVENT               CDCl3
NS                      128
DS                        0
SWH              39062.500 Hz
FIDRES            0.555591 Hz
AQ               0.8999924 sec
RG                     1030
DW                   12.800 usec
DE                    21.12 usec
TE                    300.0 K
D1               2.00000000 sec
D11              0.03000000 sec
TD0                       1

======== CHANNEL f1 =====
NUC1                    13C
P1                     9.80 usec
PL1                    5.00 dB
PL1W            26.76886177 W
SFO1            150.9201628 MHz

======== CHANNEL f2 =====
CPDPRG2             waltz16
NUC2                     1H
PCPD2                 70.00 usec
PL2                    1.00 dB
PL12                  17.23 dB
PL13                  20.00 dB
PL2W            13.76731014 W
PL12W            0.32798135 W
PL13W            0.17332016 W
SFO2            600.1324005 MHz
SI                    65536
SF              150.9027930 MHz
WDW                      EM
SSB                       0
LB                     1.00 Hz
GB                        0
PC                     1.40
```

JC–160–2
COSY.ucl CDCl3 {V:\Bruker\TOPSPIN} mjp 52

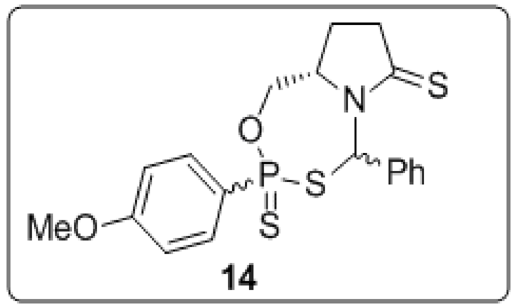

14

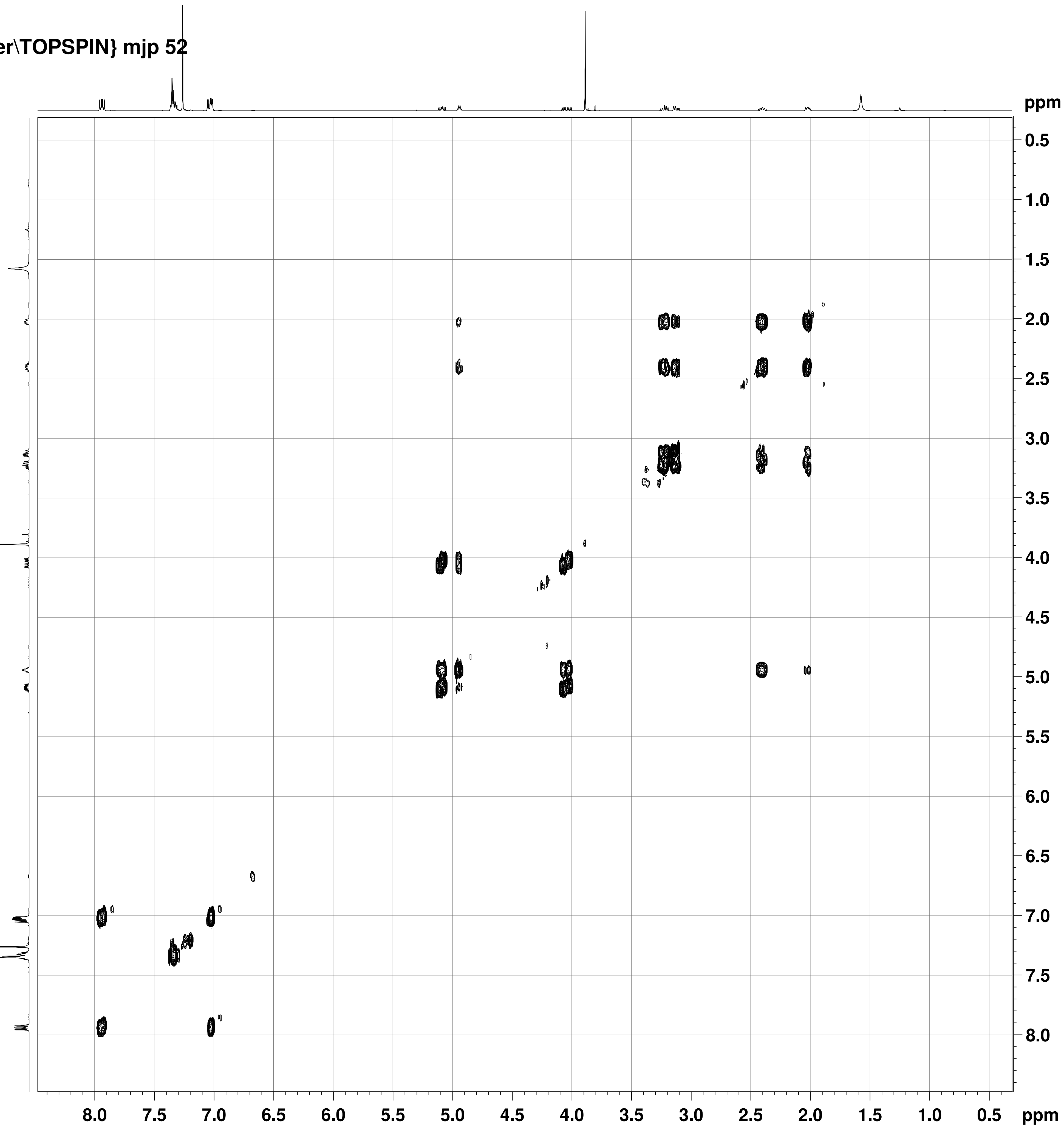


```
NAME            JC-160-2
EXPNO                 11
PROCNO                 1
Date_           20120330
Time               16.33
INSTRUM            AV600
PROBHD   5 mm CPDCH 13C
PULPROG       cosygpmfqf
TD                  2048
SOLVENT            CDCl3
NS                     1
DS                     8
SWH             4901.961 Hz
FIDRES          2.393536 Hz
AQ             0.2089460 sec
RG                  2050
DW               102.000 usec
DE                  6.50 usec
TE                 298.0 K
D0            0.00000300 sec
D1            1.69116104 sec
D13           0.00000400 sec
D16           0.00020000 sec
IN0           0.00020400 sec

======== CHANNEL f1 ========
NUC1                  1H
P1                 11.40 usec
PL1                 1.00 dB
PL1W         13.76731014 W
SFO1         600.1326476 MHz

====== GRADIENT CHANNEL ====
GPNAM1          SINE.100
GPNAM2          SINE.100
GPNAM3          SINE.100
GPZ1               16.00 %
GPZ2               12.00 %
GPZ3               40.00 %
P16              1000.00 usec
ND0                    1
TD                   128
SFO1            600.1326 MHz
FIDRES         38.296570 Hz
SW                 8.168 ppm
FnMODE                QF
SI                  1024
SF           600.1300094 MHz
WDW                QSINE
SSB                    0
LB                  0.00 Hz
GB                     0
PC                  1.40
SI                  1024
MC2                   QF
SF           600.1300094 MHz
WDW                QSINE
SSB                    0
LB                  0.00 Hz
GB                     0
```

# JC–160–2
# C13DEPT135.ucl CDCl3 {V:\Bruker\TOPSPIN} mjp 52

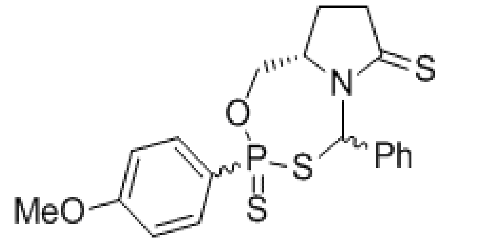


**14**

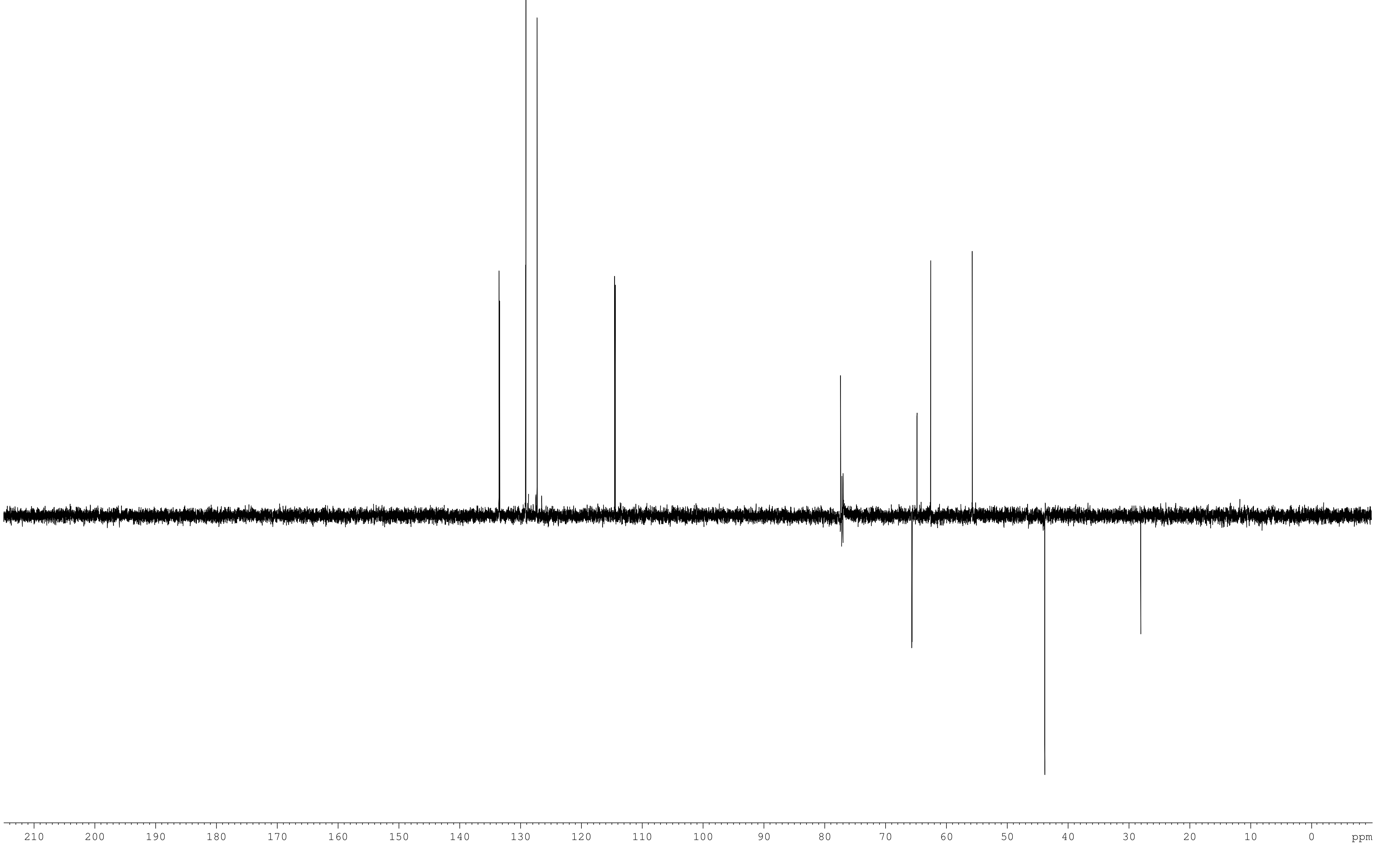


| | |
|---|---|
| NAME | JC-160-2 |
| EXPNO | 15 |
| PROCNO | 1 |
| Date_ | 20120330 |
| Time | 18.43 |
| INSTRUM | AV600 |
| PROBHD | 5 mm CPDCH 13C |
| PULPROG | dept135 |
| TD | 70308 |
| SOLVENT | CDCl3 |
| NS | 64 |
| DS | 4 |
| SWH | 39062.500 H |
| FIDRES | 0.555591 H |
| AQ | 0.8999924 s |
| RG | 256 |
| DW | 12.800 u |
| DE | 6.50 u |
| TE | 298.0 K |
| CNST2 | 145.0000000 |
| D1 | 2.00000000 s |
| D2 | 0.00344828 s |
| D12 | 0.00002000 s |
| TD0 | 1 |
| ======== CHANNEL f1 ===== | |
| NUC1 | 13C |
| P1 | 9.80 u |
| P2 | 19.60 u |
| PL1 | 5.00 d |
| PL1W | 26.76886177 W |
| SFO1 | 150.9201628 M |
| ======== CHANNEL f2 ===== | |
| CPDPRG2 | waltz16 |
| NUC2 | 1H |
| P3 | 10.80 u |
| P4 | 21.60 u |
| PCPD2 | 70.00 u |
| PL2 | 1.00 d |
| PL12 | 17.23 d |
| PL2W | 13.76731014 W |
| PL12W | 0.32798135 W |
| SFO2 | 600.1324005 M |
| SI | 65536 |
| SF | 150.9027930 M |
| WDW | EM |
| SSB | 0 |
| LB | 1.00 H |
| GB | 0 |
| PC | 1.40 |

JC−160−2
HSQC.ucl CDCl3 {V:\Bruker\TOPSPIN} mjp 52

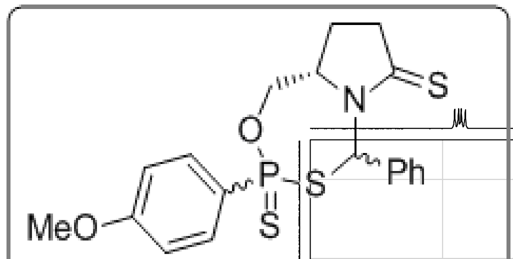

14

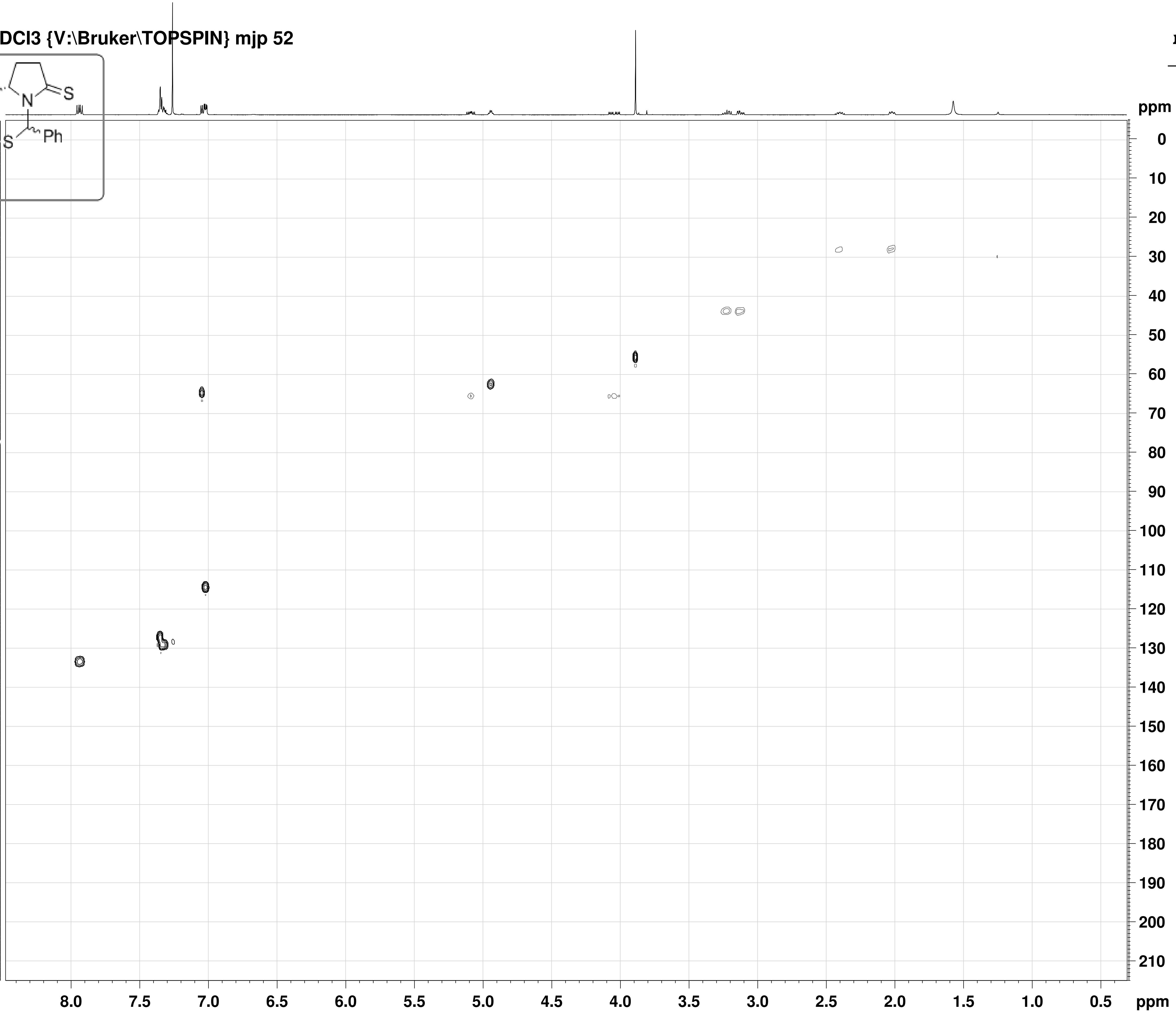


UCL

| | |
|---|---|
| NAME | JC-160-2 |
| EXPNO | 13 |
| PROCNO | 1 |
| Date_ | 20120330 |
| Time | 18.07 |
| INSTRUM | AV600 |
| PROBHD | 5 mm CPDCH 13C |
| PULPROG | hsqcedetgpsisp2.4 |
| TD | 1024 |
| SOLVENT | CDCl3 |
| NS | 2 |
| DS | 32 |
| SWH | 4901.961 Hz |
| FIDRES | 4.787071 Hz |
| AQ | 0.1044980 sec |
| RG | 2050 |
| DW | 102.000 usec |
| DE | 6.50 usec |
| TE | 298.0 K |
| CNST2 | 145.0000000 |
| CNST17 | -0.5000000 |
| D0 | 0.00000300 sec |
| D1 | 1.48074901 sec |
| D2 | 0.00344828 sec |
| D4 | 0.00172414 sec |
| D11 | 0.03000000 sec |
| D16 | 0.00020000 sec |
| D21 | 0.00344828 sec |
| D24 | 0.00086207 sec |
| IN0 | 0.00001505 sec |
| L0 | 0 |
| L31 | 1 |
| TD0 | 2 |
| ======== CHANNEL f1 ======== | |
| NUC1 | 1H |
| P1 | 11.40 usec |
| P2 | 22.80 usec |
| P28 | 0.00 usec |
| PL1 | 1.00 dB |
| PL1W | 13.76731014 W |
| SFO1 | 600.1326476 MHz |
| ======== CHANNEL f2 ======== | |
| CPDPRG2 | bi_p5m4sp_4sp.2 |
| NUC2 | 13C |
| P3 | 9.80 usec |
| P14 | 500.00 usec |
| P24 | 2000.00 usec |
| P31 | 1730.00 usec |
| P63 | 1500.00 usec |
| PL0 | 120.00 dB |
| PL2 | 5.00 dB |
| PL12 | 20.74 dB |
| PL0W | 0.00000000 W |
| PL2W | 26.76886177 W |
| PL12W | 0.71388775 W |
| SFO2 | 150.9186538 MHz |
| SP3 | 13.33 dB |
| SP7 | 13.33 dB |
| SP14 | 14.82 dB |
| SP18 | 18.73 dB |
| SP31 | 20.84 dB |
| SPNAM3 | Crp60,0.5,20.1 |
| SPNAM7 | Crp60comp.4 |
| SPNAM14 | Crp32,1.5,20.2 |
| SPNAM18 | Crp60_xfilt.2 |
| SPNAM31 | Crp32,1.5,20.2 |
| SPOAL3 | 0.500 |
| SPOAL7 | 0.500 |
| SPOAL14 | 0.500 |
| SPOAL18 | 0.500 |
| SPOAL31 | 0.500 |
| SPOFFS3 | 0.00 Hz |
| SPOFFS7 | 0.00 Hz |
| SPOFFS14 | 0.00 Hz |
| SPOFFS18 | 0.00 Hz |
| SPOFFS31 | 0.00 Hz |
| ====== GRADIENT CHANNEL ===== | |
| GPNAM1 | SINE.100 |
| GPNAM2 | SINE.100 |
| GPNAM3 | SINE.100 |
| GPNAM4 | SINE.100 |
| GPZ1 | 80.00 % |
| GPZ2 | 20.10 % |
| GPZ3 | 11.00 % |
| GPZ4 | -5.00 % |
| P16 | 1000.00 usec |
| P19 | 600.00 usec |
| ND0 | 2 |
| TD | 128 |
| SFO1 | 150.9187 MHz |
| FIDRES | 259.391449 Hz |
| SW | 220.000 ppm |
| FnMODE | Echo-Antiecho |
| SI | 1024 |
| SF | 600.1300094 MHz |
| WDW | QSINE |
| SSB | 2 |
| LB | 0.00 Hz |
| GB | 0 |
| PC | 1.40 |
| SI | 1024 |
| MC2 | echo-antiecho |
| SF | 150.9027771 MHz |
| WDW | QSINE |
| SSB | 2 |
| LB | 0.00 Hz |
| GB | 0 |

JC–160–2
HMBC.ucl CDCl3 {V:\Bruker\TOPSPIN} mjp 52

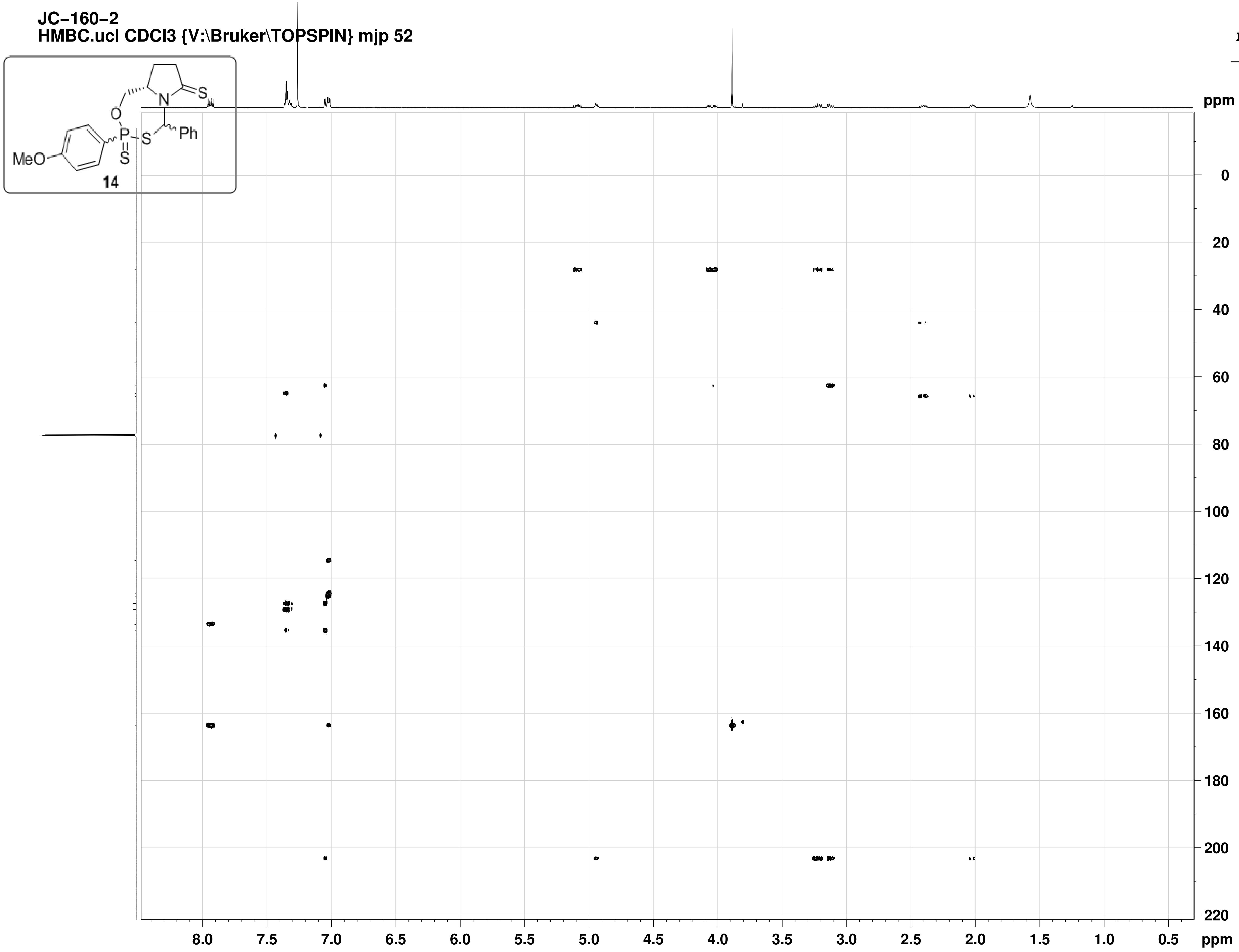


UCL

| | | |
|---|---|---|
| NAME | JC-160-2 | |
| EXPNO | 14 | |
| PROCNO | 1 | |
| Date_ | 20120330 | |
| Time | 18.29 | |
| INSTRUM | AV600 | |
| PROBHD | 5 mm CPDCH 13C | |
| PULPROG | hmbcetgpl3nd | |
| TD | 4096 | |
| SOLVENT | CDCl3 | |
| NS | 2 | |
| DS | 16 | |
| SWH | 4901.961 | Hz |
| FIDRES | 1.196768 | Hz |
| AQ | 0.4178420 | sec |
| RG | 2050 | |
| DW | 102.000 | usec |
| DE | 6.50 | usec |
| TE | 298.0 | K |
| CNST6 | 120.0000000 | |
| CNST7 | 160.0000000 | |
| CNST13 | 10.0000000 | |
| CNST30 | 0.5981152 | |
| D0 | 0.00000300 | sec |
| D1 | 0.76000261 | sec |
| D6 | 0.05000000 | sec |
| D16 | 0.00020000 | sec |
| IN0 | 0.00001380 | sec |
| ======== CHANNEL f1 ======== | | |
| NUC1 | 1H | |
| P1 | 11.40 | usec |
| P2 | 22.80 | usec |
| PL1 | 1.00 | dB |
| PL1W | 13.76731014 | W |
| SFO1 | 600.1326476 | MHz |
| ======== CHANNEL f2 ======== | | |
| NUC2 | 13C | |
| P3 | 9.80 | usec |
| P24 | 2000.00 | usec |
| PL2 | 5.00 | dB |
| PL2W | 26.76886177 | W |
| SFO2 | 150.9178993 | MHz |
| SP7 | 13.33 | dB |
| SPNAM7 | Crp60comp.4 | |
| SPOAL7 | 0.500 | |
| SPOFFS7 | 0.00 | Hz |
| ====== GRADIENT CHANNEL ===== | | |
| GPNAM1 | SINE.100 | |
| GPNAM3 | SINE.100 | |
| GPNAM4 | SINE.100 | |
| GPNAM5 | SINE.100 | |
| GPNAM6 | SINE.100 | |
| GPZ1 | 80.00 | % |
| GPZ3 | 14.00 | % |
| GPZ4 | -8.00 | % |
| GPZ5 | -4.00 | % |
| GPZ6 | -2.00 | % |
| P16 | 1000.00 | usec |
| ND0 | 2 | |
| TD | 256 | |
| SFO1 | 150.9179 | MHz |
| FIDRES | 141.485535 | Hz |
| SW | 240.000 | ppm |
| FnMODE | Echo-Antiecho | |
| SI | 2048 | |
| SF | 600.1300106 | MHz |
| WDW | SINE | |
| SSB | 2 | |
| LB | 0.00 | Hz |
| GB | 0 | |
| PC | 1.40 | |
| SI | 1024 | |
| MC2 | echo-antiecho | |
| SF | 150.9027756 | MHz |
| WDW | SINE | |
| SSB | 2 | |
| LB | 0.00 | Hz |
| GB | 0 | |

**JC–166–2**
**PROTON.ucl CDCl3 {V:\Bruker\TOPSPIN} mjp 54**

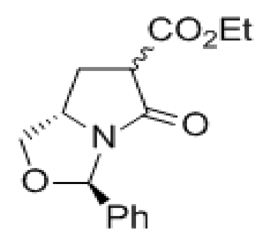


**13a**

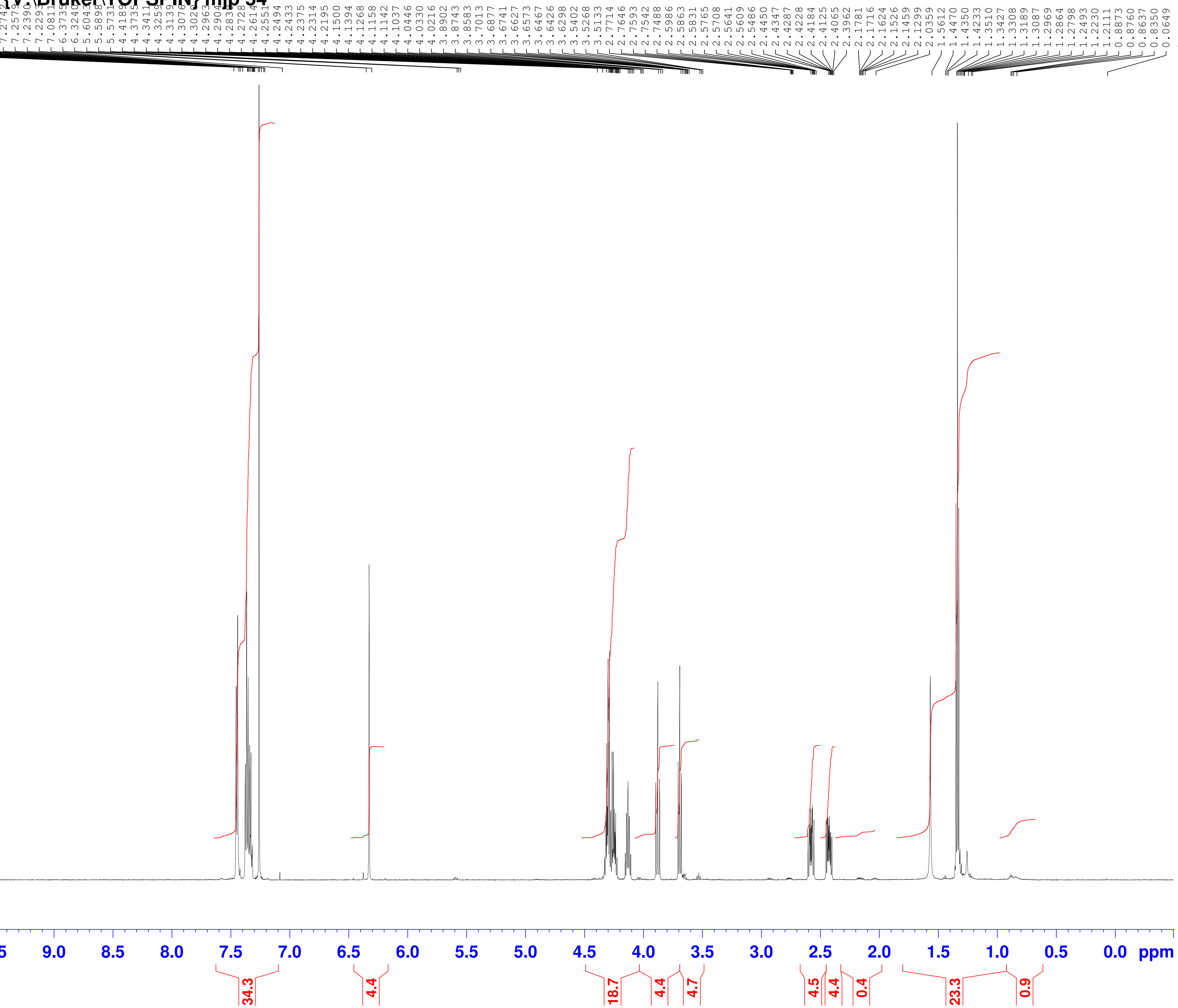


| | |
|---|---|
| NAME | JC-166-2 |
| EXPNO | 10 |
| PROCNO | 1 |
| Date_ | 20120206 |
| Time | 16.55 |
| INSTRUM | AV600 |
| PROBHD | 5 mm CPDCH 13C |
| PULPROG | zg30 |
| TD | 98682 |
| SOLVENT | CDCl3 |
| NS | 8 |
| DS | 0 |
| SWH | 12335.526 Hz |
| FIDRES | 0.125003 Hz |
| AQ | 3.9999604 sec |
| RG | 32 |
| DW | 40.533 use |
| DE | 10.48 use |
| TE | 298.0 K |
| D1 | 1.00000000 sec |
| TD0 | 1 |

======== CHANNEL f1 ========

| | |
|---|---|
| NUC1 | 1H |
| P1 | 11.40 use |
| PL1 | 1.00 dB |
| PL1W | 13.76731014 W |
| SFO1 | 600.1337061 MHz |
| SI | 32768 |
| SF | 600.1300116 MHz |
| WDW | EM |
| SSB | 0 |
| LB | 0.30 Hz |
| GB | 0 |
| PC | 1.40 |

**JC–166–2**
**C13CPD.ucl CDCl3 {V:\Bruker\TOPSPIN} mjp 54**

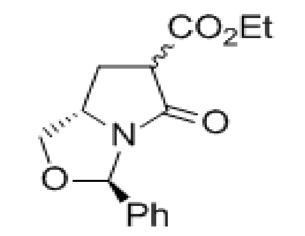


**13a**

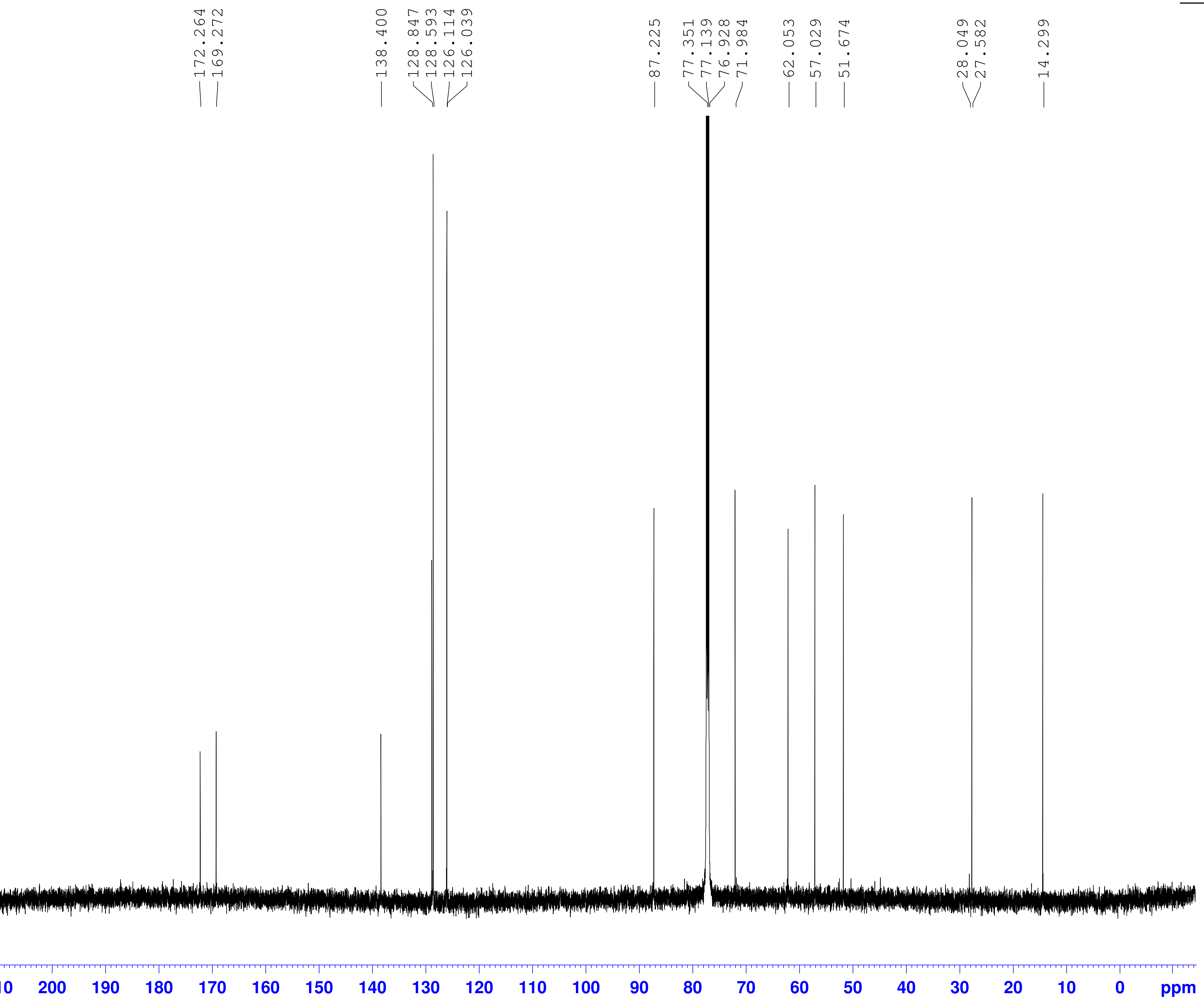


| | |
|---|---|
| NAME | JC-166-2 |
| EXPNO | 12 |
| PROCNO | 1 |
| Date_ | 20120207 |
| Time | 10.22 |
| INSTRUM | AV600 |
| PROBHD | 5 mm CPDCH 13C |
| PULPROG | zgpg30 |
| TD | 70308 |
| SOLVENT | CDCl3 |
| NS | 128 |
| DS | 0 |
| SWH | 39062.500 H |
| FIDRES | 0.555591 H |
| AQ | 0.8999924 s |
| RG | 1030 |
| DW | 12.800 u |
| DE | 21.12 u |
| TE | 298.0 K |
| D1 | 2.00000000 s |
| D11 | 0.03000000 s |
| TD0 | 1 |
| ======== CHANNEL f1 ===== | |
| NUC1 | 13C |
| P1 | 9.80 u |
| PL1 | 5.00 d |
| PL1W | 26.76886177 W |
| SFO1 | 150.9201628 M |
| ======== CHANNEL f2 ===== | |
| CPDPRG2 | waltz16 |
| NUC2 | 1H |
| PCPD2 | 70.00 u |
| PL2 | 1.00 d |
| PL12 | 17.23 d |
| PL13 | 20.00 d |
| PL2W | 13.76731014 W |
| PL12W | 0.32798135 W |
| PL13W | 0.17332016 W |
| SFO2 | 600.1324005 M |
| SI | 65536 |
| SF | 150.9027930 M |
| WDW | EM |
| SSB | 0 |
| LB | 1.00 H |
| GB | 0 |
| PC | 1.40 |

**JC–166–2**
**COSY.ucl CDCl3 {V:\Bruker\TOPSPIN} mjp 54**

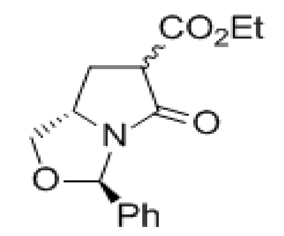


**13a**

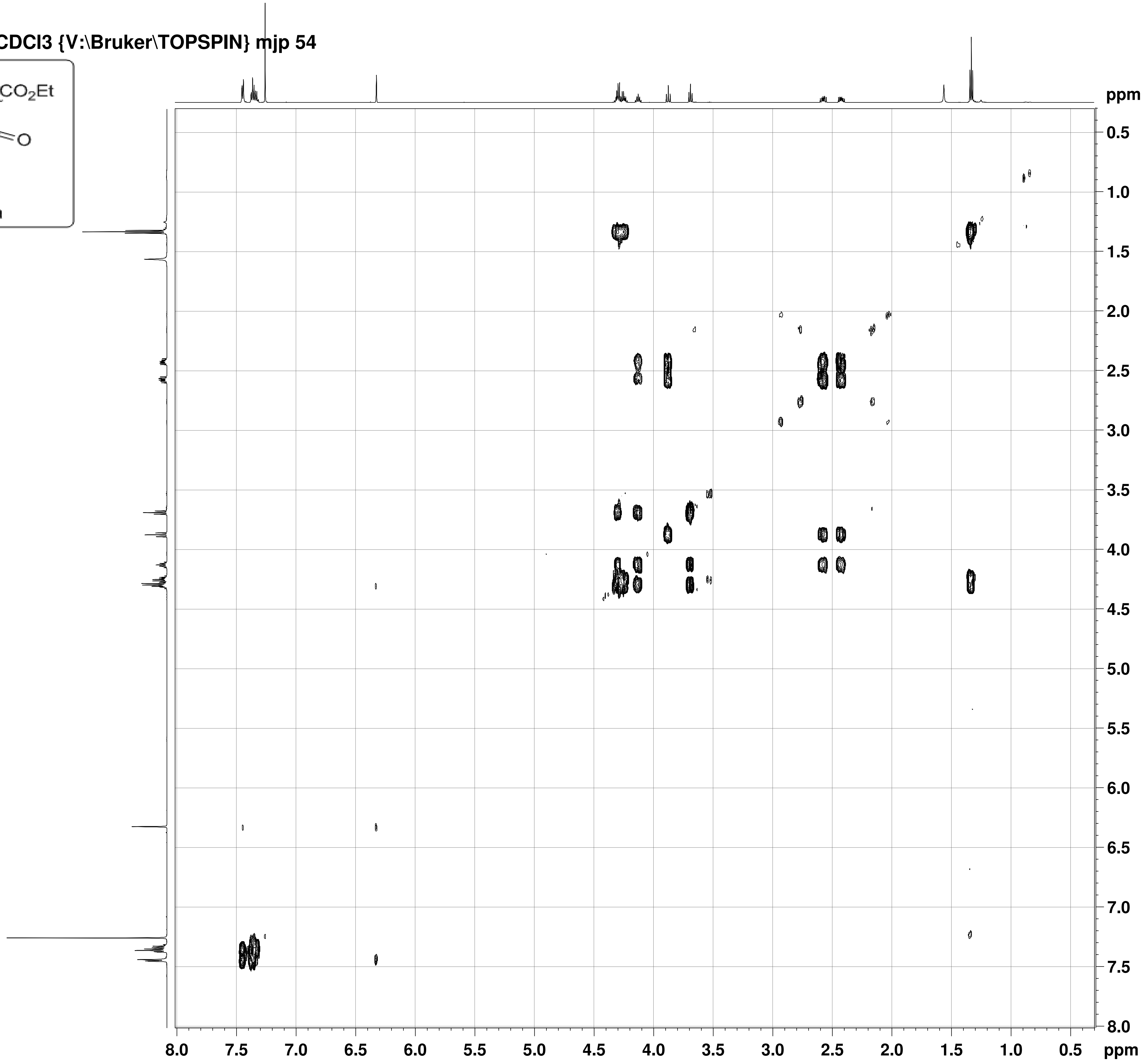


```
NAME              JC-166-2
EXPNO                   11
PROCNO                   1
Date_             20120207
Time                 10.11
INSTRUM              AV600
PROBHD   5 mm CPDCH 13C
PULPROG         cosygpmfqf
TD                    2048
SOLVENT              CDCl3
NS                       1
DS                       8
SWH               4629.629 Hz
FIDRES            2.260561 Hz
AQ               0.2212340 sec
RG                    2050
DW                 108.000 usec
DE                    6.50 usec
TE                   298.0 K
D0              0.00000300 sec
D1              1.67887294 sec
D13             0.00000400 sec
D16             0.00020000 sec
IN0             0.00021600 sec

======== CHANNEL f1 ========
NUC1                    1H
P1                   11.40 usec
PL1                   1.00 dB
PL1W           13.76731014 W
SFO1           600.1325063 MHz

====== GRADIENT CHANNEL ====
GPNAM1            SINE.100
GPNAM2            SINE.100
GPNAM3            SINE.100
GPZ1                 16.00 %
GPZ2                 12.00 %
GPZ3                 40.00 %
P16                1000.00 usec
ND0                      1
TD                     128
SFO1              600.1325 MHz
FIDRES           36.168980 Hz
SW                   7.714 ppm
FnMODE                  QF
SI                    1024
SF             600.1300095 MHz
WDW                  QSINE
SSB                      0
LB                    0.00 Hz
GB                       0
PC                    1.40
SI                    1024
MC2                     QF
SF             600.1300095 MHz
WDW                  QSINE
SSB                      0
LB                    0.00 Hz
GB                       0
```

JC–166–2
HSQC.ucl CDCl3 {V:\Bruker\TOPSPIN} mjp 54

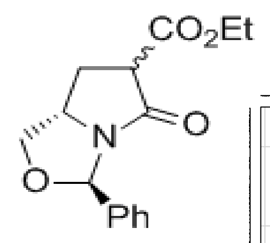


13a

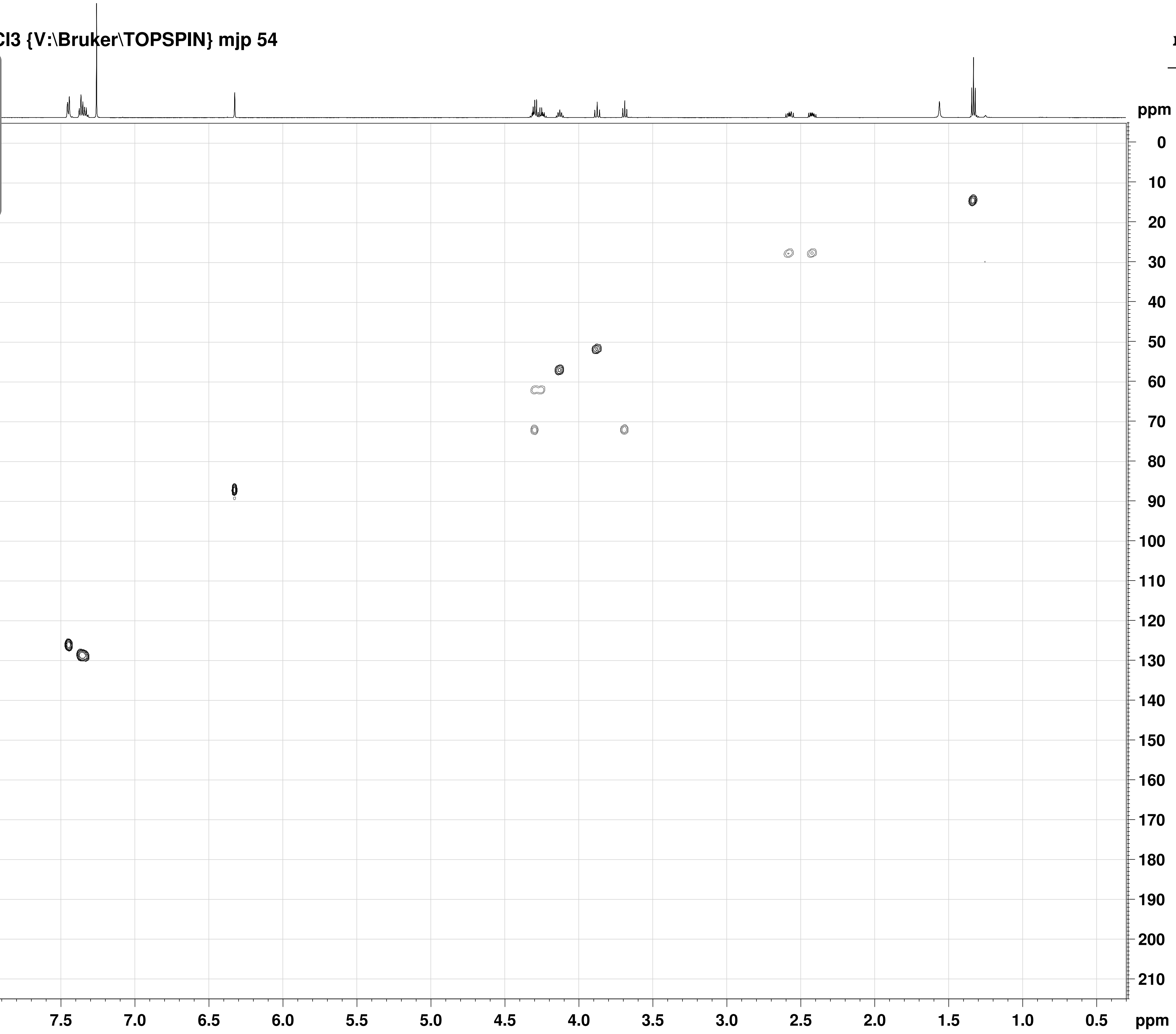


```
NAME              JC-166-2
EXPNO                   13
PROCNO                   1
Date_             20120207
Time                 10.23
INSTRUM              AV600
PROBHD   5 mm CPDCH 13C
PULPROG  hsqcedetgpsisp2.4
TD                    1024
SOLVENT              CDCl3
NS                       2
DS                      32
SWH               4629.629 Hz
FIDRES            4.521122 Hz
AQ               0.1106420 sec
RG                    2050
DW                 108.000 usec
DE                    6.50 usec
TE                   298.0 K
CNST2          145.0000000
CNST17          -0.5000000
D0              0.00000300 sec
D1              1.47460496 sec
D2              0.00344828 sec
D4              0.00172414 sec
D11             0.03000000 sec
D16             0.00020000 sec
D21             0.00344828 sec
D24             0.00086207 sec
IN0             0.00001505 sec
L0                       0
L31                      1
TD0                      2

======== CHANNEL f1 ========
NUC1                    1H
P1                   11.40 usec
P2                   22.80 usec
P28                   0.00 usec
PL1                   1.00 dB
PL1W           13.76731014 W
SFO1          600.1325063 MHz

======== CHANNEL f2 ========
CPDPRG2   bi_p5m4sp_4sp.2
NUC2                   13C
P3                    9.80 usec
P14                 500.00 usec
P24                2000.00 usec
P31                1730.00 usec
P63                1500.00 usec
PL0                 120.00 dB
PL2                   5.00 dB
PL12                 20.74 dB
PL0W            0.00000000 W
PL2W           26.76886177 W
PL12W           0.71388775 W
SFO2          150.9186538 MHz
SP3                  13.33 dB
SP7                  13.33 dB
SP14                 14.82 dB
SP18                 18.73 dB
SP31                 20.84 dB
SPNAM3    Crp60,0.5,20.1
SPNAM7       Crp60comp.4
SPNAM14   Crp32,1.5,20.2
SPNAM18    Crp60_xfilt.2
SPNAM31   Crp32,1.5,20.2
SPOAL3               0.500
SPOAL7               0.500
SPOAL14              0.500
SPOAL18              0.500
SPOAL31              0.500
SPOFFS3               0.00 Hz
SPOFFS7               0.00 Hz
SPOFFS14              0.00 Hz
SPOFFS18              0.00 Hz
SPOFFS31              0.00 Hz

====== GRADIENT CHANNEL =====
GPNAM1           SINE.100
GPNAM2           SINE.100
GPNAM3           SINE.100
GPNAM4           SINE.100
GPZ1                80.00 %
GPZ2                20.10 %
GPZ3                11.00 %
GPZ4                -5.00 %
P16               1000.00 usec
P19                600.00 usec
ND0                      2
TD                     128
SFO1              150.9187 MHz
FIDRES          259.391449 Hz
SW                 220.000 ppm
FnMODE       Echo-Antiecho
SI                    1024
SF             600.1300095 MHz
WDW                  QSINE
SSB                      2
LB                    0.00 Hz
GB                       0
PC                    1.40
SI                    1024
MC2            echo-antiecho
SF             150.9027771 MHz
WDW                  QSINE
SSB                      2
LB                    0.00 Hz
GB                       0
```

JC–166–2
HMBC.ucl CDCl3 {V:\Bruker\TOPSPIN} mjp 54

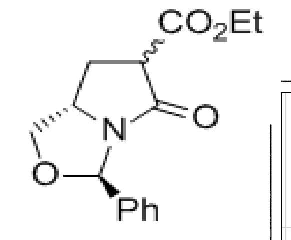

**13a**

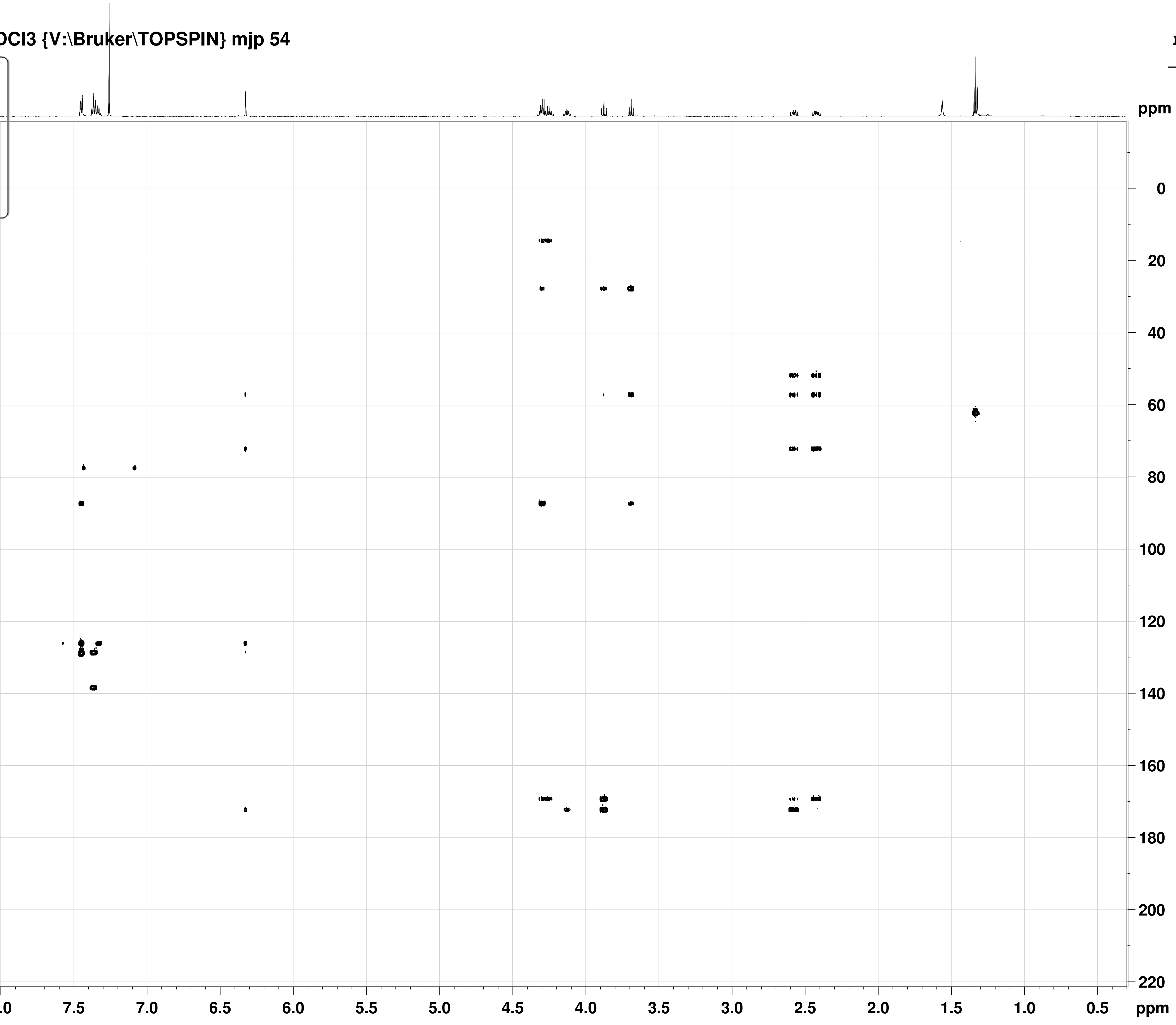


| | |
|---|---|
| NAME | JC-166-2 |
| EXPNO | 14 |
| PROCNO | 1 |
| Date_ | 20120207 |
| Time | 10.31 |
| INSTRUM | AV600 |
| PROBHD | 5 mm CPDCH 13C |
| PULPROG | hmbcetgpl3nd |
| TD | 4096 |
| SOLVENT | CDCl3 |
| NS | 2 |
| DS | 16 |
| SWH | 4629.629 Hz |
| FIDRES | 1.130281 Hz |
| AQ | 0.4424180 sec |
| RG | 2050 |
| DW | 108.000 usec |
| DE | 6.50 usec |
| TE | 298.0 K |
| CNST6 | 120.0000000 |
| CNST7 | 160.0000000 |
| CNST13 | 10.0000000 |
| CNST30 | 0.5981151 |
| D0 | 0.00000300 sec |
| D1 | 0.73542649 sec |
| D6 | 0.05000000 sec |
| D16 | 0.00020000 sec |
| IN0 | 0.00001380 sec |
| ======== CHANNEL f1 ======== | |
| NUC1 | 1H |
| P1 | 11.40 usec |
| P2 | 22.80 usec |
| PL1 | 1.00 dB |
| PL1W | 13.76731014 W |
| SFO1 | 600.1325063 MHz |
| ======== CHANNEL f2 ======== | |
| NUC2 | 13C |
| P3 | 9.80 usec |
| P24 | 2000.00 usec |
| PL2 | 5.00 dB |
| PL2W | 26.76886177 W |
| SFO2 | 150.9178993 MHz |
| SP7 | 13.33 dB |
| SPNAM7 | Crp60comp.4 |
| SPOAL7 | 0.500 |
| SPOFFS7 | 0.00 Hz |
| ====== GRADIENT CHANNEL ===== | |
| GPNAM1 | SINE.100 |
| GPNAM3 | SINE.100 |
| GPNAM4 | SINE.100 |
| GPNAM5 | SINE.100 |
| GPNAM6 | SINE.100 |
| GPZ1 | 80.00 % |
| GPZ3 | 14.00 % |
| GPZ4 | -8.00 % |
| GPZ5 | -4.00 % |
| GPZ6 | -2.00 % |
| P16 | 1000.00 usec |
| ND0 | 2 |
| TD | 256 |
| SFO1 | 150.9179 MHz |
| FIDRES | 141.485535 Hz |
| SW | 240.000 ppm |
| FnMODE | Echo-Antiecho |
| SI | 2048 |
| SF | 600.1300107 MHz |
| WDW | SINE |
| SSB | 2 |
| LB | 0.00 Hz |
| GB | 0 |
| PC | 1.40 |
| SI | 1024 |
| MC2 | echo-antiecho |
| SF | 150.9027756 MHz |
| WDW | SINE |
| SSB | 2 |
| LB | 0.00 Hz |
| GB | 0 |

JC/266/3
PROTON.ucl CDCl3 {C:\Bruker\TOPSPIN} mjp 52

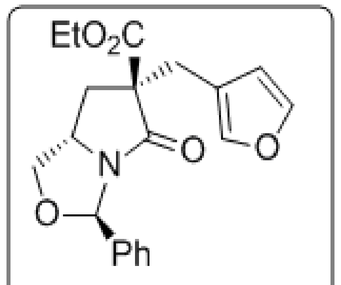


*epi*-**15**

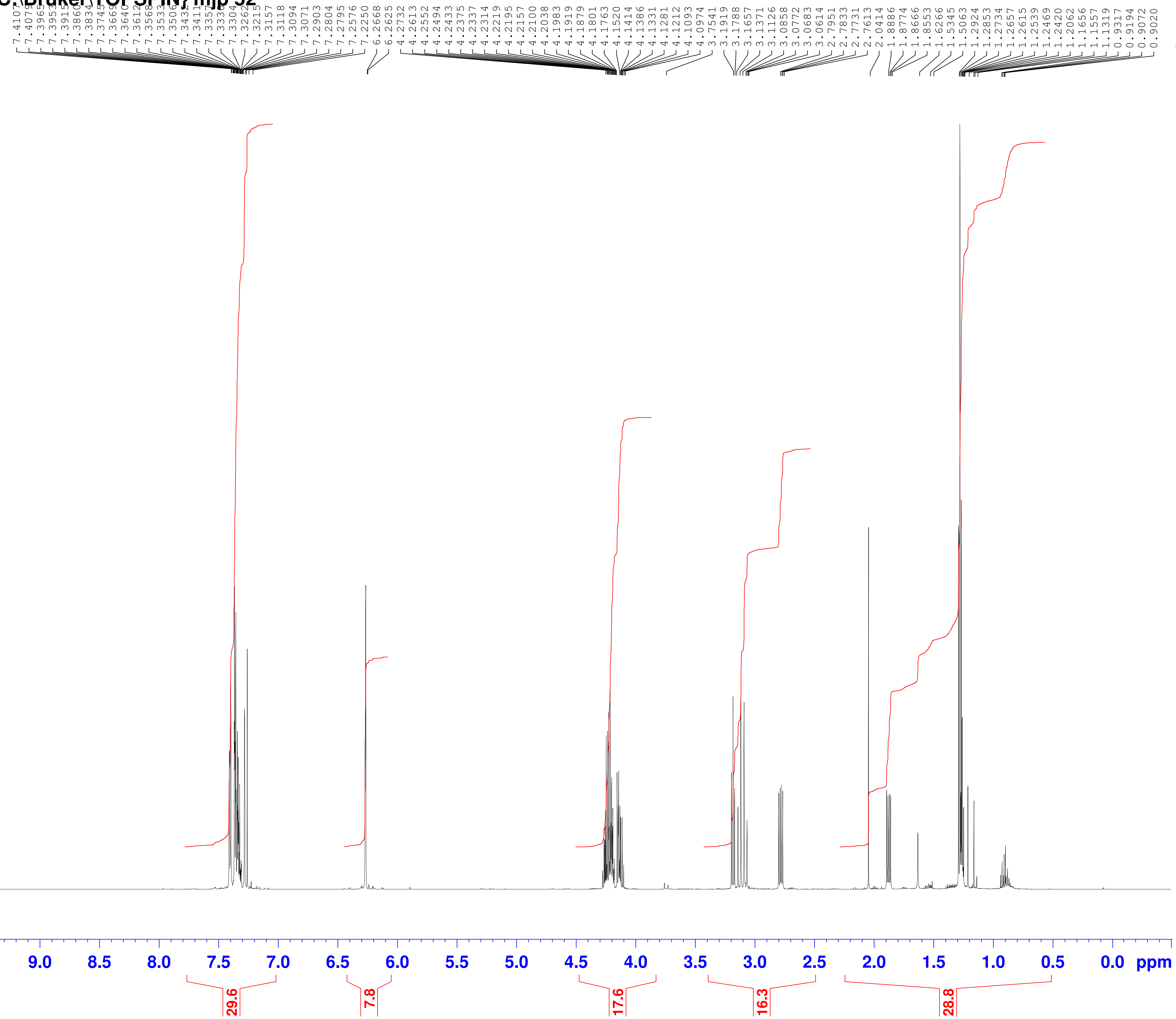


| | |
|---|---|
| NAME | Feb22-2010 |
| EXPNO | 20 |
| PROCNO | 1 |
| Date_ | 20100222 |
| Time | 20.38 |
| INSTRUM | AV600 |
| PROBHD | 5 mm CPDCH 13C |
| PULPROG | zg30 |
| TD | 65536 |
| SOLVENT | CDCl3 |
| NS | 8 |
| DS | 0 |
| SWH | 12335.526 Hz |
| FIDRES | 0.188225 Hz |
| AQ | 2.6564426 sec |
| RG | 32 |
| DW | 40.533 use |
| DE | 10.48 use |
| TE | 298.0 K |
| D1 | 1.00000000 sec |
| TD0 | 1 |

======== CHANNEL f1 ========

| | |
|---|---|
| NUC1 | 1H |
| P1 | 11.40 use |
| PL1 | 1.00 dB |
| PL1W | 13.76731014 W |
| SFO1 | 600.1337061 MHz |
| SI | 32768 |
| SF | 600.1300116 MHz |
| WDW | EM |
| SSB | 0 |
| LB | 0.30 Hz |
| GB | 0 |
| PC | 1.40 |

JC/266/3
C13CPD.ucl CDCl3 {C:\Bruker\TOPSPIN} mjp 52

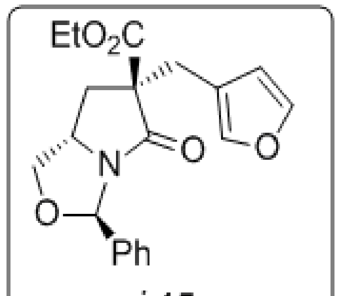


*epi*-**15**

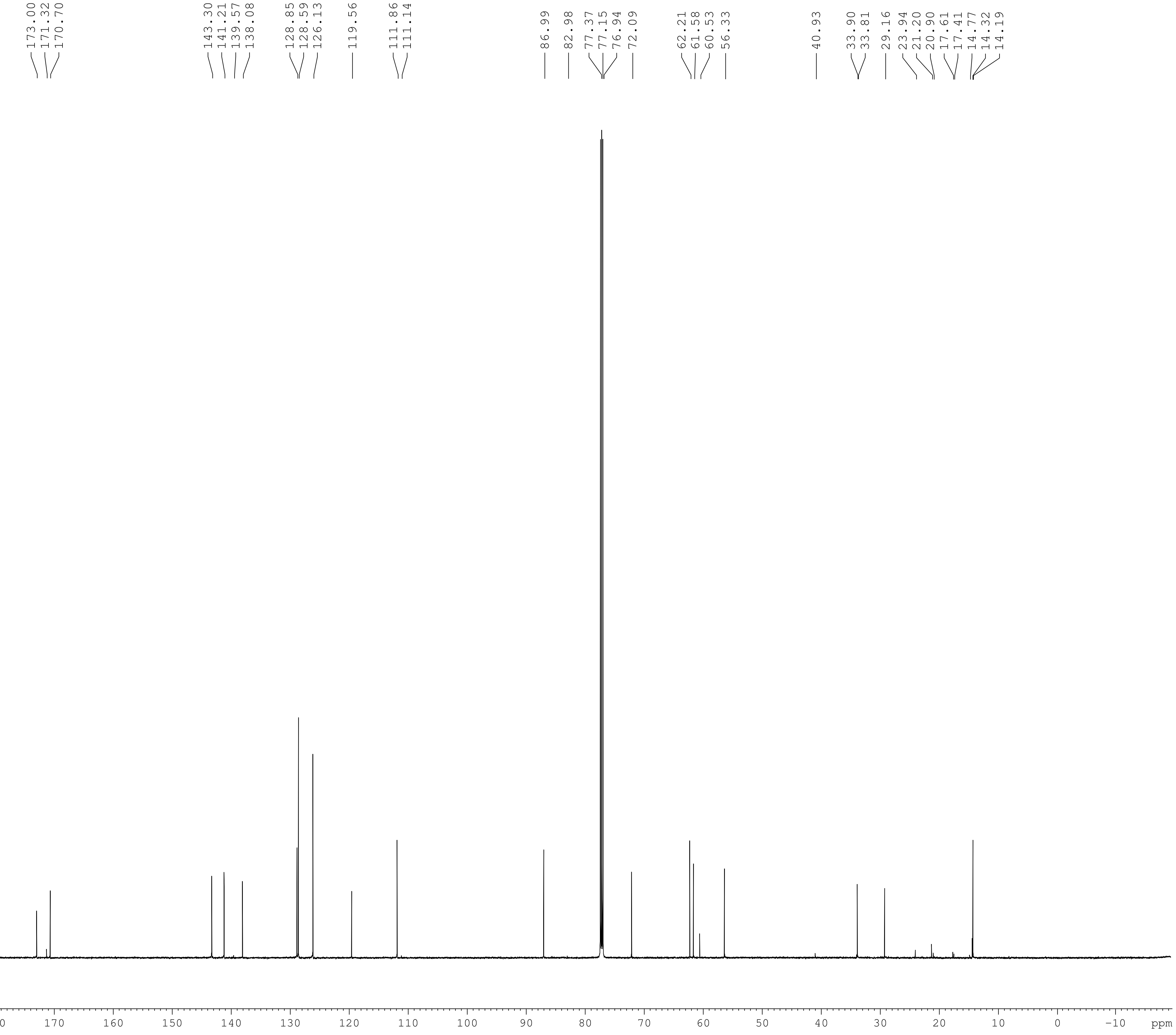


UCL

| | | |
|---|---|---|
| NAME | Feb22-2010 | |
| EXPNO | 21 | |
| PROCNO | 1 | |
| Date_ | 20100222 | |
| Time | 20.45 | |
| INSTRUM | AV600 | |
| PROBHD | 5 mm CPDCH 13C | |
| PULPROG | zgpg30 | |
| TD | 65536 | |
| SOLVENT | CDCl3 | |
| NS | 128 | |
| DS | 0 | |
| SWH | 36057.691 | H |
| FIDRES | 0.550197 | H |
| AQ | 0.9088159 | s |
| RG | 1030 | |
| DW | 13.867 | u |
| DE | 21.55 | u |
| TE | 298.0 | K |
| D1 | 2.00000000 | s |
| D11 | 0.03000000 | s |
| TD0 | 1 | |
| ======== CHANNEL f1 ===== | | |
| NUC1 | 13C | |
| P1 | 9.80 | u |
| PL1 | 5.00 | d |
| PL1W | 26.76886177 | W |
| SFO1 | 150.9178981 | M |
| ======== CHANNEL f2 ===== | | |
| CPDPRG2 | waltz16 | |
| NUC2 | 1H | |
| PCPD2 | 70.00 | u |
| PL2 | 1.00 | d |
| PL12 | 16.76 | d |
| PL13 | 120.00 | d |
| PL2W | 13.76731014 | W |
| PL12W | 0.36546776 | W |
| PL13W | 0.00000000 | W |
| SFO2 | 600.1324005 | M |
| SI | 32768 | |
| SF | 150.9027930 | M |
| WDW | EM | |
| SSB | 0 | |
| LB | 1.00 | H |
| GB | 0 | |
| PC | 1.40 | |

**JC/266/3**
**COSY.ucl CDCl3 {C:\Bruker\TOPSPIN} mjp 52**

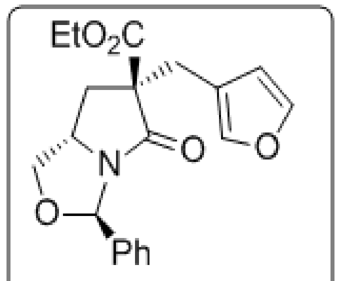

*epi*-**15**

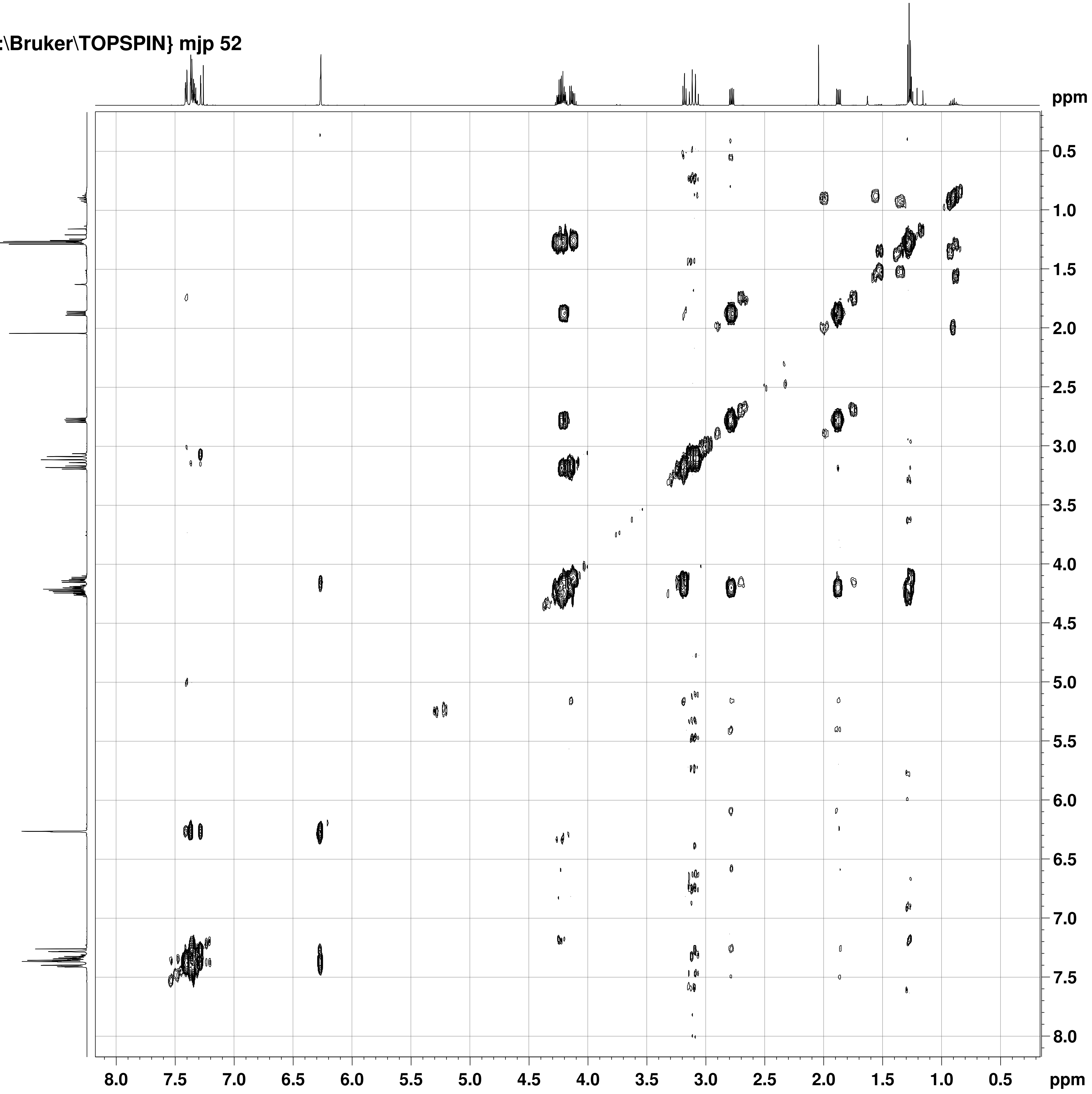


UCL

```
NAME              Feb22-2010
EXPNO                     24
PROCNO                     1
Date_               20100222
Time                   21.05
INSTRUM                AV600
PROBHD   5 mm CPDCH 13C
PULPROG          cosygpmfqf
TD                      2048
SOLVENT                CDCl3
NS                         1
DS                         8
SWH                 4807.692 Hz
FIDRES              2.347506 Hz
AQ                 0.2130420 sec
RG                      1820
DW                   104.000 usec
DE                      6.50 usec
TE                     298.0 K
D0                0.00000300 sec
D1                1.68706501 sec
D13               0.00000400 sec
D16               0.00020000 sec
IN0               0.00020800 sec

======== CHANNEL f1 ========
NUC1                      1H
P1                     11.40 usec
PL1                     1.00 dB
PL1W             13.76731014 W
SFO1             600.1325142 MHz

====== GRADIENT CHANNEL ====
GPNAM1             SINE.100
GPNAM2             SINE.100
GPNAM3             SINE.100
GPZ1                   16.00 %
GPZ2                   12.00 %
GPZ3                   40.00 %
P16                  1000.00 usec
ND0                        1
TD                       128
SFO1                600.1325 MHz
FIDRES             37.560097 Hz
SW                     8.011 ppm
FnMODE                    QF
SI                      1024
SF               600.1300094 MHz
WDW                    QSINE
SSB                        0
LB                      0.00 Hz
GB                         0
PC                      1.40
SI                      1024
MC2                       QF
SF               600.1300094 MHz
WDW                    QSINE
SSB                        0
LB                      0.00 Hz
GB                         0
```

JC/266/3
HSQC.ucl CDCl3 {C:\Bruker\TOPSPIN} mjp 52

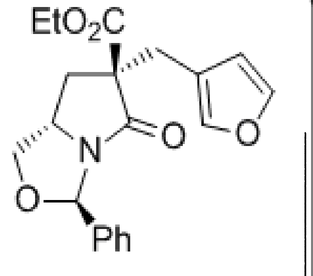

*epi*-**15**

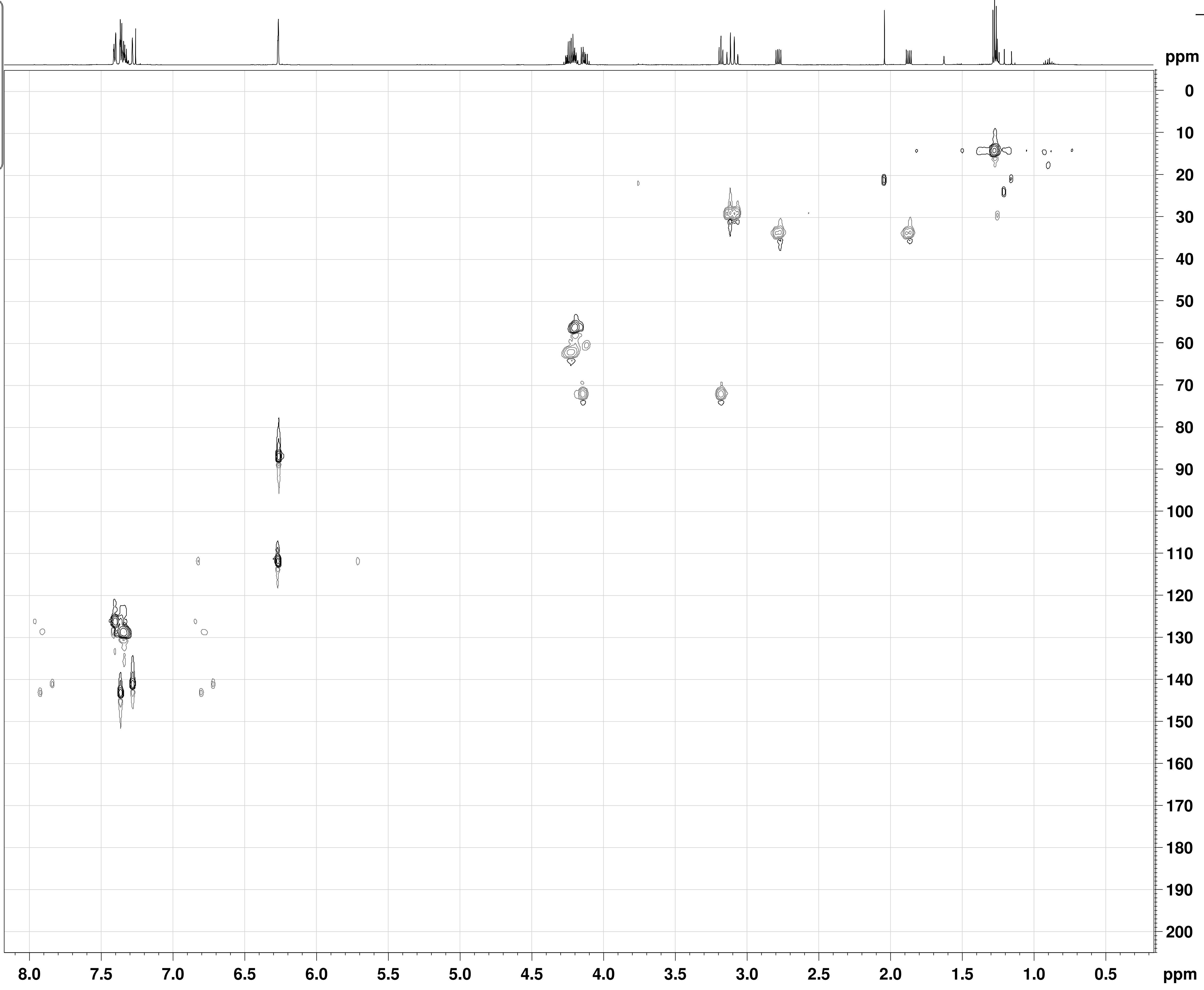

UCL

```
NAME          Feb22-2010
EXPNO                 22
PROCNO                 1
Date_           20100222
Time               20.46
INSTRUM            AV600
PROBHD   5 mm CPDCH 13C
PULPROG  hsqcedetgpsisp2.4
TD                  1024
SOLVENT            CDCl3
NS                     2
DS                    32
SWH             4807.692 Hz
FIDRES          4.695012 Hz
AQ             0.1065460 sec
RG                  2050
DW               104.000 usec
DE                  6.50 usec
TE                 298.0 K
CNST2        145.0000000
CNST17        -0.5000000
D0            0.00000300 sec
D1            1.47870100 sec
D2            0.00344828 sec
D4            0.00172414 sec
D11           0.03000000 sec
D16           0.00020000 sec
D21           0.00344828 sec
D24           0.00086207 sec
IN0           0.00001580 sec
L0                     0
L31                    1
TD0                    2

======== CHANNEL f1 ========
NUC1                  1H
P1                 11.40 usec
P2                 22.80 usec
P28                 0.00 usec
PL1                 1.00 dB
PL1W         13.76731014 W
SFO1         600.1325142 MHz

======== CHANNEL f2 ========
CPDPRG2  bi_p5m4sp_4sp.2
NUC2                 13C
P3                  9.80 usec
P14               500.00 usec
P24              2000.00 usec
P31              1730.00 usec
P63              1500.00 usec
PL0               120.00 dB
PL2                 5.00 dB
PL12               20.74 dB
PL0W          0.00000000 W
PL2W         26.76886177 W
PL12W         0.71388775 W
SFO2         150.9178993 MHz
SP3                13.33 dB
SP7                13.33 dB
SP14               14.82 dB
SP18               18.73 dB
SP31               20.84 dB
SPNAM3    Crp60,0.5,20.1
SPNAM7       Crp60comp.4
SPNAM14   Crp32,1.5,20.2
SPNAM18    Crp60_xfilt.2
SPNAM31   Crp32,1.5,20.2
SPOAL3             0.500
SPOAL7             0.500
SPOAL14            0.500
SPOAL18            0.500
SPOAL31            0.500
SPOFFS3             0.00 Hz
SPOFFS7             0.00 Hz
SPOFFS14            0.00 Hz
SPOFFS18            0.00 Hz
SPOFFS31            0.00 Hz

====== GRADIENT CHANNEL =====
GPNAM1          SINE.100
GPNAM2          SINE.100
GPNAM3          SINE.100
GPNAM4          SINE.100
GPZ1               80.00 %
GPZ2               20.10 %
GPZ3               11.00 %
GPZ4               -5.00 %
P16              1000.00 usec
P19               600.00 usec
ND0                    2
TD                   128
SFO1            150.9179 MHz
FIDRES        247.599686 Hz
SW               210.000 ppm
FnMODE      Echo-Antiecho
SI                  1024
SF           600.1300094 MHz
WDW                QSINE
SSB                    2
LB                  0.00 Hz
GB                     0
PC                  1.40
SI                  1024
MC2         echo-antiecho
SF           150.9027781 MHz
WDW                QSINE
SSB                    2
LB                  0.00 Hz
GB                     0
```

JC/266/3
HMBC.ucl CDCl3 {C:\Bruker\TOPSPIN} mjp 52

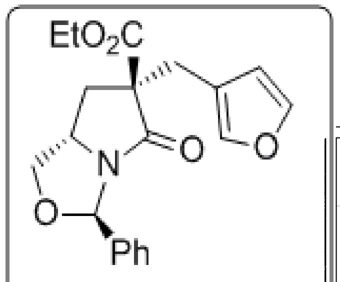


*epi*-**15**

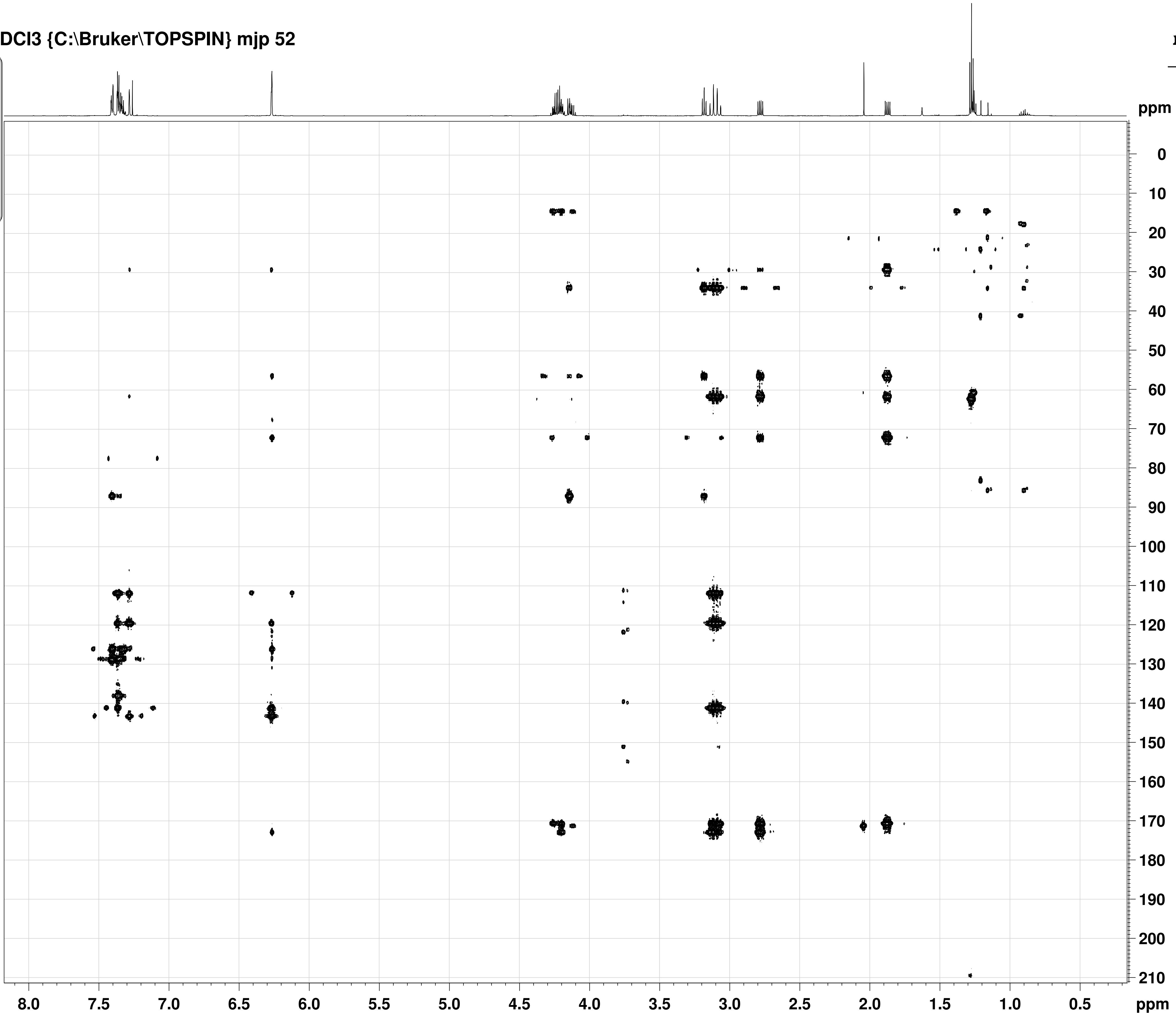


```
NAME             Feb22-2010
EXPNO                    23
PROCNO                    1
Date_              20100222
Time                  20.54
INSTRUM               AV600
PROBHD    5 mm CPDCH 13C
PULPROG       hmbcetgpl3nd
TD                     4096
SOLVENT               CDCl3
NS                        2
DS                       16
SWH               4807.692 Hz
FIDRES            1.173753 Hz
AQ               0.4260340 sec
RG                     2050
DW                  104.000 usec
DE                     6.50 usec
TE                    298.0 K
CNST6           120.0000000
CNST7           160.0000000
CNST13           10.0000000
CNST30            0.5981151
D0               0.00000300 sec
D1               0.75181061 sec
D6               0.05000000 sec
D16              0.00020000 sec
IN0              0.00001505 sec

======== CHANNEL f1 ========
NUC1                     1H
P1                    11.40 usec
P2                    22.80 usec
PL1                    1.00 dB
PL1W            13.76731014 W
SFO1            600.1325142 MHz

======== CHANNEL f2 ========
NUC2                    13C
P3                     9.80 usec
P24                 2000.00 usec
PL2                    5.00 dB
PL2W            26.76886177 W
SFO2            150.9178993 MHz
SP7                   13.33 dB
SPNAM7          Crp60comp.4
SPOAL7                0.500
SPOFFS7                0.00 Hz

====== GRADIENT CHANNEL =====
GPNAM1             SINE.100
GPNAM3             SINE.100
GPNAM4             SINE.100
GPNAM5             SINE.100
GPNAM6             SINE.100
GPZ1                  80.00 %
GPZ3                  14.00 %
GPZ4                  -8.00 %
GPZ5                  -4.00 %
GPZ6                  -2.00 %
P16                 1000.00 usec
ND0                       2
TD                      256
SFO1               150.9179 MHz
FIDRES           129.695068 Hz
SW                  220.000 ppm
FnMODE         Echo-Antiecho
SI                     2048
SF              600.1300106 MHz
WDW                    SINE
SSB                       2
LB                     0.00 Hz
GB                        0
PC                     1.40
SI                     1024
MC2             echo-antiecho
SF              150.9027773 MHz
WDW                    SINE
SSB                       2
LB                     0.00 Hz
GB                        0
```

JC/266/3
PROTON.ucl CDCl3 {C:\Bruker\TOPSPIN} mjp 53

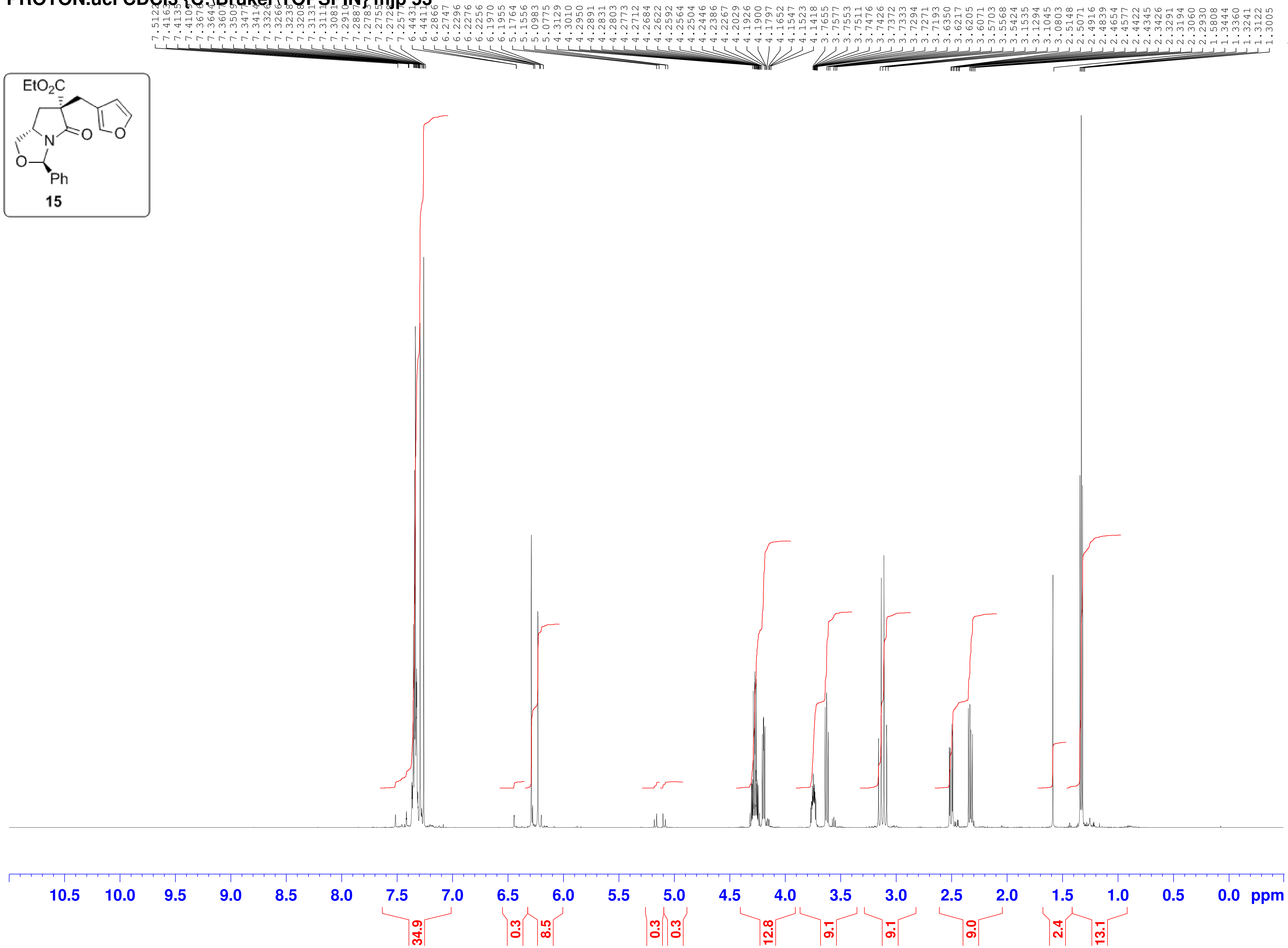


| | |
|---|---|
| NAME | Feb22-2010 |
| EXPNO | 10 |
| PROCNO | 1 |
| Date_ | 20100222 |
| Time | 21.12 |
| INSTRUM | AV600 |
| PROBHD | 5 mm CPDCH 13C |
| PULPROG | zg30 |
| TD | 65536 |
| SOLVENT | CDCl3 |
| NS | 8 |
| DS | 0 |
| SWH | 12335.526 Hz |
| FIDRES | 0.188225 Hz |
| AQ | 2.6564426 sec |
| RG | 36 |
| DW | 40.533 use |
| DE | 10.48 use |
| TE | 298.0 K |
| D1 | 1.00000000 sec |
| TD0 | 1 |
| ======== CHANNEL f1 ======== | |
| NUC1 | 1H |
| P1 | 11.40 use |
| PL1 | 1.00 dB |
| PL1W | 13.76731014 W |
| SFO1 | 600.1337061 MHz |
| SI | 32768 |
| SF | 600.1300116 MHz |
| WDW | EM |
| SSB | 0 |
| LB | 0.30 Hz |
| GB | 0 |
| PC | 1.40 |

**JC/266/3**
**C13CPD.ucl CDCl3 {C:\Bruker\TOPSPIN} mjp 53**

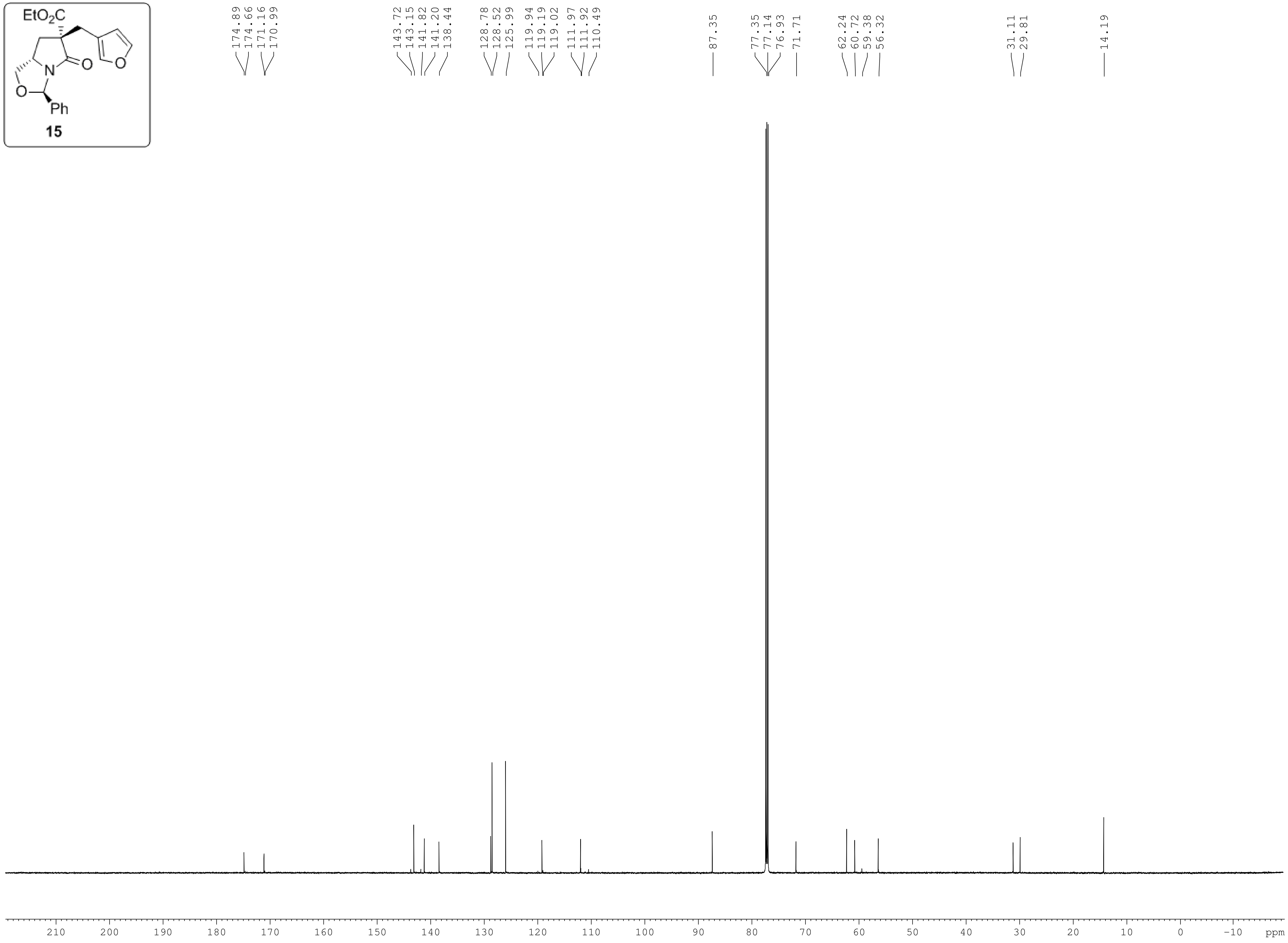


| | |
|---|---|
| NAME | Feb22-2010 |
| EXPNO | 11 |
| PROCNO | 1 |
| Date_ | 20100222 |
| Time | 21.19 |
| INSTRUM | AV600 |
| PROBHD | 5 mm CPDCH 13C |
| PULPROG | zgpg30 |
| TD | 65536 |
| SOLVENT | CDCl3 |
| NS | 128 |
| DS | 0 |
| SWH | 36057.691 H |
| FIDRES | 0.550197 H |
| AQ | 0.9088159 s |
| RG | 1030 |
| DW | 13.867 u |
| DE | 21.55 u |
| TE | 298.0 K |
| D1 | 2.00000000 s |
| D11 | 0.03000000 s |
| TD0 | 1 |
| ======== CHANNEL f1 ===== | |
| NUC1 | 13C |
| P1 | 9.80 u |
| PL1 | 5.00 d |
| PL1W | 26.76886177 W |
| SFO1 | 150.9178981 M |
| ======== CHANNEL f2 ===== | |
| CPDPRG2 | waltz16 |
| NUC2 | 1H |
| PCPD2 | 70.00 u |
| PL2 | 1.00 d |
| PL12 | 16.76 d |
| PL13 | 120.00 d |
| PL2W | 13.76731014 W |
| PL12W | 0.36546776 W |
| PL13W | 0.00000000 W |
| SFO2 | 600.1324005 M |
| SI | 32768 |
| SF | 150.9027930 M |
| WDW | EM |
| SSB | 0 |
| LB | 1.00 H |
| GB | 0 |
| PC | 1.40 |

**JC/266/3**
**COSY.ucl CDCl3 {C:\Bruker\TOPSPIN} mjp 53**

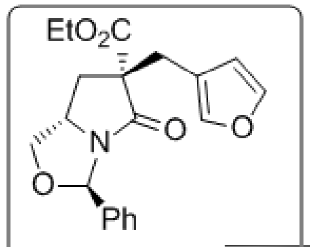

**15**

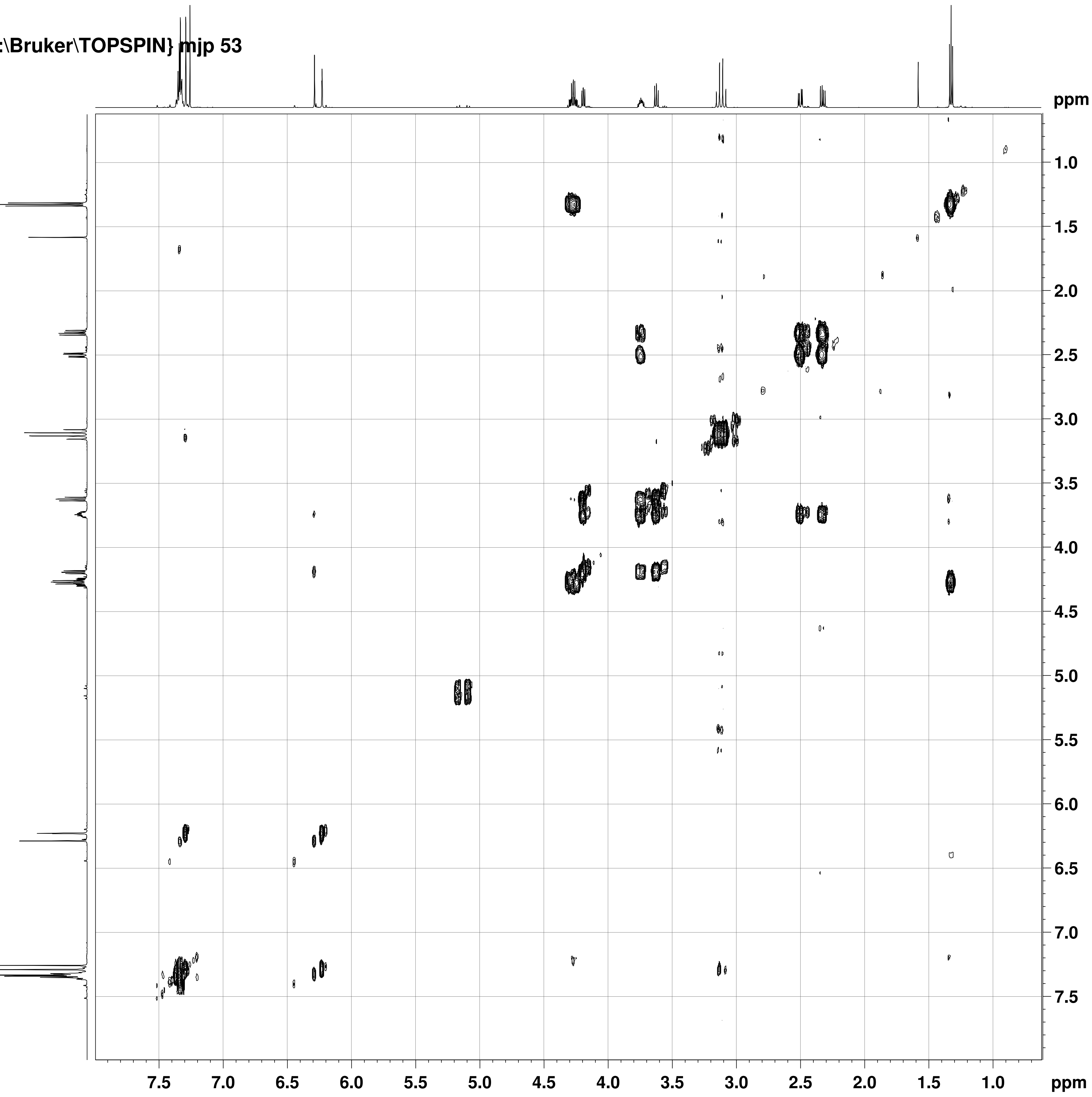


UCL

```
NAME           Feb22-2010
EXPNO                  14
PROCNO                  1
Date_            20100222
Time                21.39
INSTRUM             AV600
PROBHD   5 mm CPDCH 13C
PULPROG       cosygpmfqf
TD                   2048
SOLVENT             CDCl3
NS                      1
DS                      8
SWH              4424.779 Hz
FIDRES           2.160537 Hz
AQ              0.2314740 sec
RG                   2050
DW                113.000 usec
DE                   6.50 usec
TE                  298.0 K
D0             0.00000300 sec
D1             1.66863298 sec
D13            0.00000400 sec
D16            0.00020000 sec
IN0            0.00022600 sec

======== CHANNEL f1 ========
NUC1                   1H
P1                  11.40 usec
PL1                  1.00 dB
PL1W          13.76731014 W
SFO1          600.1325966 MHz

====== GRADIENT CHANNEL ====
GPNAM1           SINE.100
GPNAM2           SINE.100
GPNAM3           SINE.100
GPZ1                16.00 %
GPZ2                12.00 %
GPZ3                40.00 %
P16               1000.00 usec
ND0                     1
TD                    128
SFO1             600.1326 MHz
FIDRES          34.568584 Hz
SW                  7.373 ppm
FnMODE                 QF
SI                   1024
SF            600.1300096 MHz
WDW                 QSINE
SSB                     0
LB                   0.00 Hz
GB                      0
PC                   1.40
SI                   1024
MC2                    QF
SF            600.1300096 MHz
WDW                 QSINE
SSB                     0
LB                   0.00 Hz
GB                      0
```

JC/266/3
HSQC.ucl CDCl3 {C:\Bruker\TOPSPIN} mjp 53

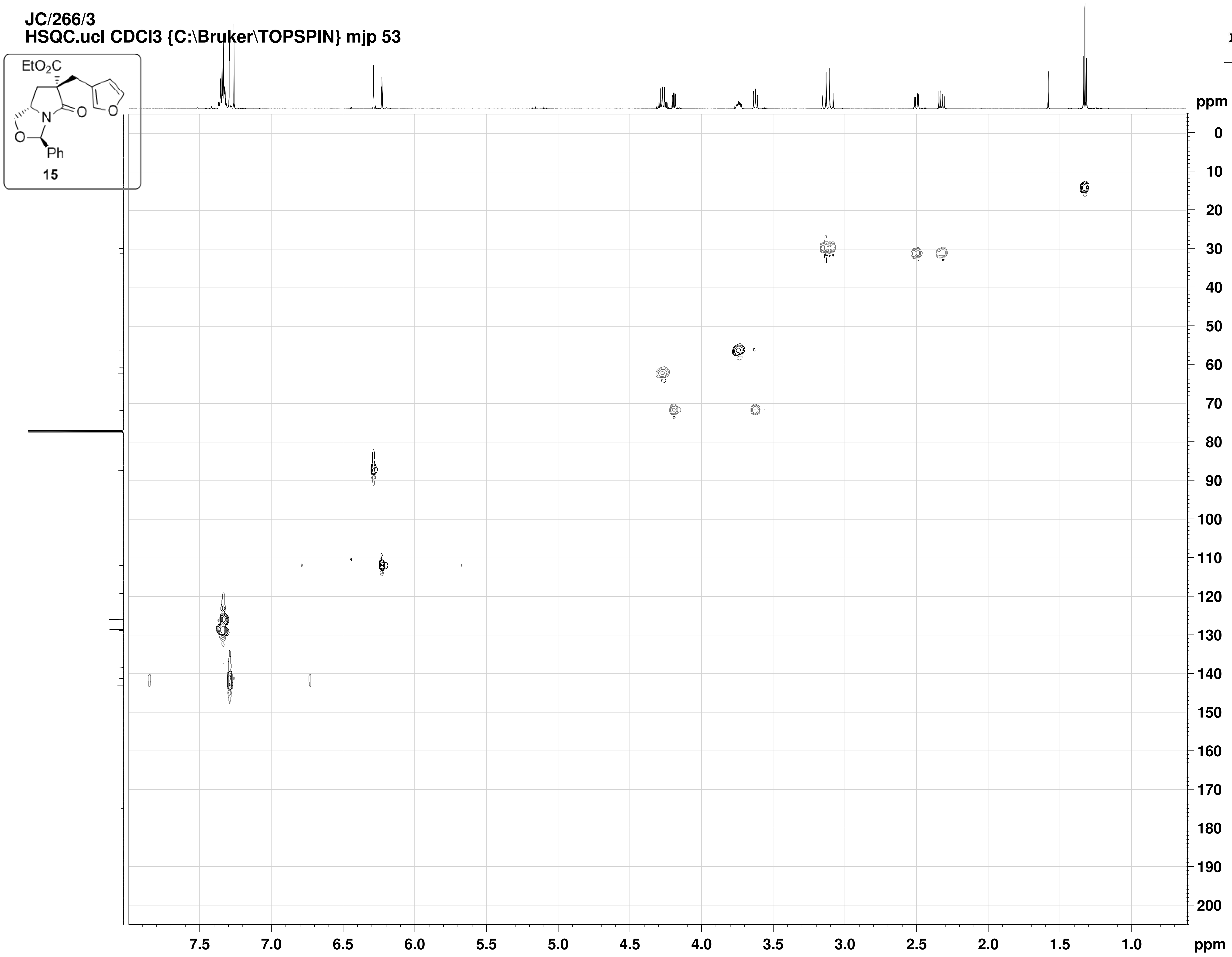


```
NAME          Feb22-2010
EXPNO                 13
PROCNO                 1
Date_           20100222
Time               21.31
INSTRUM            AV600
PROBHD   5 mm CPDCH 13C
PULPROG  hsqcedetgpsisp2.4
TD                  1024
SOLVENT            CDCl3
NS                     2
DS                    32
SWH             4424.779 Hz
FIDRES          4.321073 Hz
AQ             0.1157620 sec
RG                  2050
DW               113.000 usec
DE                  6.50 usec
TE                 298.0 K
CNST2        145.0000000
CNST17        -0.5000000
D0            0.00000300 sec
D1            1.46948504 sec
D2            0.00344828 sec
D4            0.00172414 sec
D11           0.03000000 sec
D16           0.00020000 sec
D21           0.00344828 sec
D24           0.00086207 sec
IN0           0.00001580 sec
L0                     0
L31                    1
TD0                    2

======== CHANNEL f1 ========
NUC1                  1H
P1                 11.40 usec
P2                 22.80 usec
P28                 0.00 usec
PL1                 1.00 dB
PL1W         13.76731014 W
SFO1         600.1325966 MHz

======== CHANNEL f2 ========
CPDPRG2  bi_p5m4sp_4sp.2
NUC2                 13C
P3                  9.80 usec
P14               500.00 usec
P24              2000.00 usec
P31              1730.00 usec
P63              1500.00 usec
PL0               120.00 dB
PL2                 5.00 dB
PL12               20.74 dB
PL0W          0.00000000 W
PL2W         26.76886177 W
PL12W         0.71388775 W
SFO2         150.9178993 MHz
SP3                13.33 dB
SP7                13.33 dB
SP14               14.82 dB
SP18               18.73 dB
SP31               20.84 dB
SPNAM3    Crp60,0.5,20.1
SPNAM7      Crp60comp.4
SPNAM14   Crp32,1.5,20.2
SPNAM18    Crp60_xfilt.2
SPNAM31   Crp32,1.5,20.2
SPOAL3             0.500
SPOAL7             0.500
SPOAL14            0.500
SPOAL18            0.500
SPOAL31            0.500
SPOFFS3             0.00 Hz
SPOFFS7             0.00 Hz
SPOFFS14            0.00 Hz
SPOFFS18            0.00 Hz
SPOFFS31            0.00 Hz

====== GRADIENT CHANNEL =====
GPNAM1          SINE.100
GPNAM2          SINE.100
GPNAM3          SINE.100
GPNAM4          SINE.100
GPZ1               80.00 %
GPZ2               20.10 %
GPZ3               11.00 %
GPZ4               -5.00 %
P16              1000.00 usec
P19               600.00 usec
ND0                    2
TD                   128
SFO1            150.9179 MHz
FIDRES        247.599686 Hz
SW               210.000 ppm
FnMODE      Echo-Antiecho
SI                  1024
SF           600.1300096 MHz
WDW                QSINE
SSB                    2
LB                  0.00 Hz
GB                     0
PC                  1.40
SI                  1024
MC2         echo-antiecho
SF           150.9027781 MHz
WDW                QSINE
SSB                    2
LB                  0.00 Hz
GB                     0
```

JC/266/3
HMBC.ucl CDCl3 {C:\Bruker\TOPSPIN} mjp 53

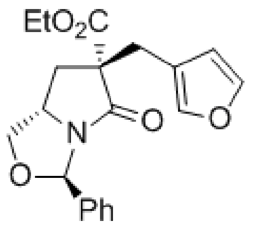


15

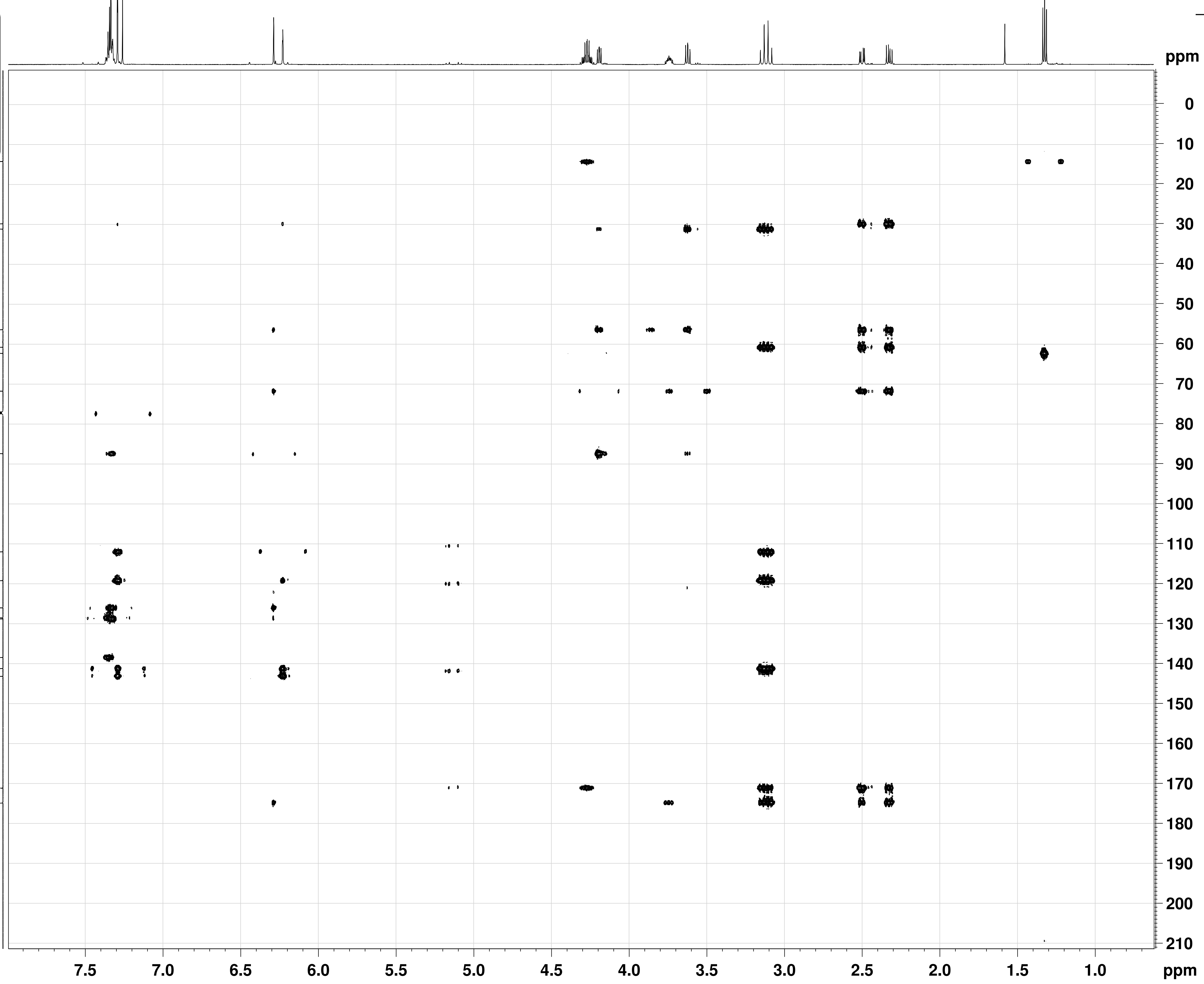


UCL

```
NAME            Feb22-2010
EXPNO                   12
PROCNO                   1
Date_             20100222
Time                 21.19
INSTRUM              AV600
PROBHD   5 mm CPDCH 13C
PULPROG      hmbcetgpl3nd
TD                    4096
SOLVENT              CDCl3
NS                       2
DS                      16
SWH               4424.779 Hz
FIDRES            1.080268 Hz
AQ               0.4628980 sec
RG                    2050
DW                 113.000 usec
DE                    6.50 usec
TE                   298.0 K
CNST6          120.0000000
CNST7          160.0000000
CNST13          10.0000000
CNST30           0.5981151
D0              0.00000300 sec
D1              0.71494663 sec
D6              0.05000000 sec
D16             0.00020000 sec
IN0             0.00001505 sec

======== CHANNEL f1 ========
NUC1                    1H
P1                   11.40 usec
P2                   22.80 usec
PL1                   1.00 dB
PL1W           13.76731014 W
SFO1           600.1325966 MHz

======== CHANNEL f2 ========
NUC2                   13C
P3                    9.80 usec
P24                2000.00 usec
PL2                   5.00 dB
PL2W           26.76886177 W
SFO2           150.9178993 MHz
SP7                  13.33 dB
SPNAM7          Crp60comp.4
SPOAL7               0.500
SPOFFS7               0.00 Hz

====== GRADIENT CHANNEL =====
GPNAM1            SINE.100
GPNAM3            SINE.100
GPNAM4            SINE.100
GPNAM5            SINE.100
GPNAM6            SINE.100
GPZ1                 80.00 %
GPZ3                 14.00 %
GPZ4                 -8.00 %
GPZ5                 -4.00 %
GPZ6                 -2.00 %
P16                1000.00 usec
ND0                      2
TD                     256
SFO1              150.9179 MHz
FIDRES          129.695068 Hz
SW                 220.000 ppm
FnMODE        Echo-Antiecho
SI                    2048
SF             600.1300107 MHz
WDW                   SINE
SSB                      2
LB                    0.00 Hz
GB                       0
PC                    1.40
SI                    1024
MC2           echo-antiecho
SF             150.9027773 MHz
WDW                   SINE
SSB                      2
LB                    0.00 Hz
GB                       0
```

**JC−324−1**
**PROTON.ucl CDCl3 {V:\Bruker\TOPSPIN} mjp 43**

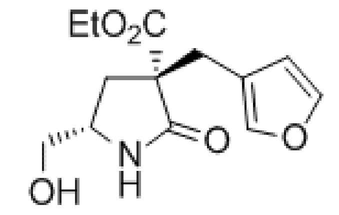


**15a**

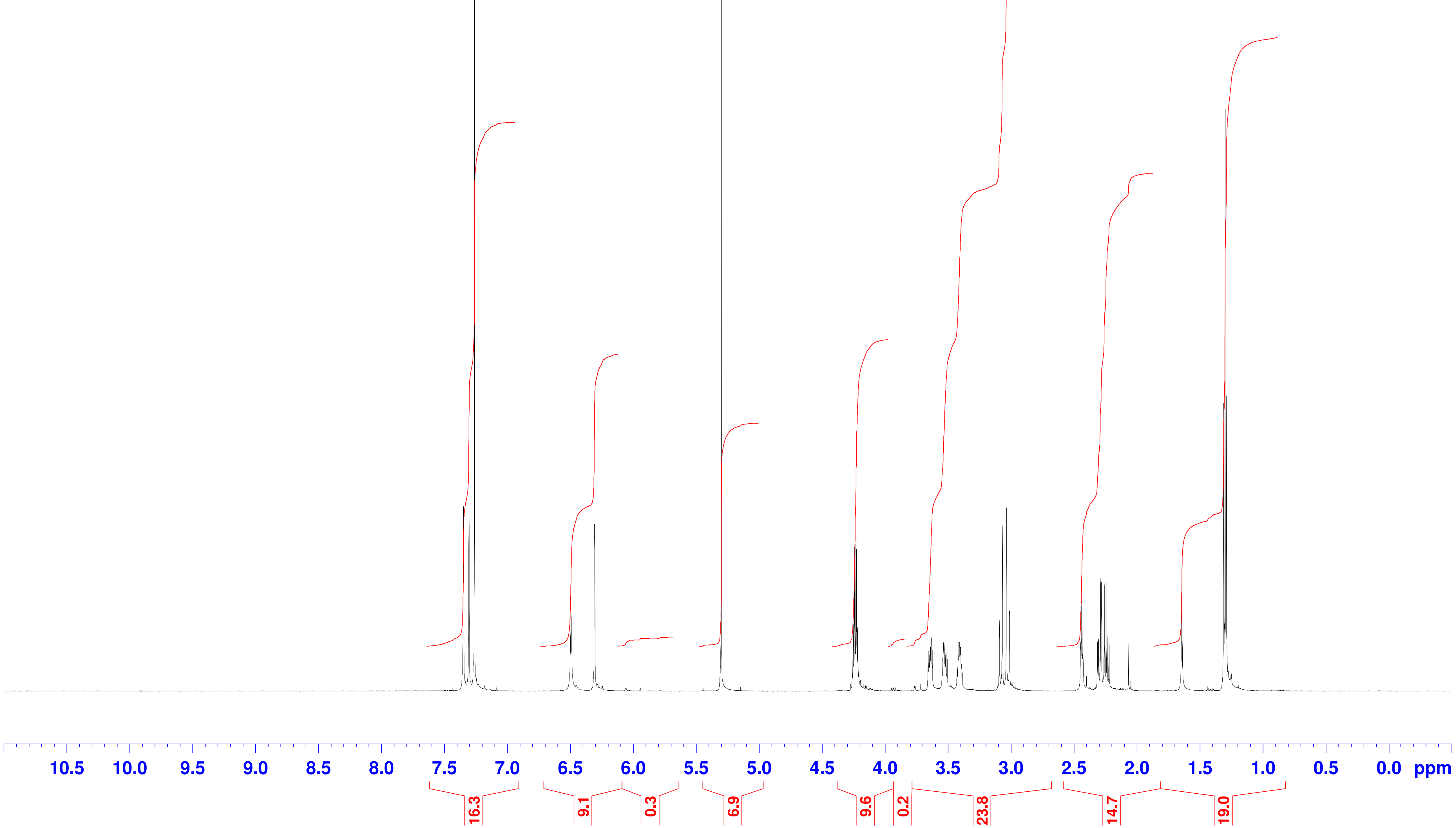


| | |
|---|---|
| NAME | JC-324-1 |
| EXPNO | 10 |
| PROCNO | 1 |
| Date_ | 20120317 |
| Time | 14.15 |
| INSTRUM | AV600 |
| PROBHD | 5 mm CPDCH 13C |
| PULPROG | zg30 |
| TD | 98682 |
| SOLVENT | CDCl3 |
| NS | 8 |
| DS | 0 |
| SWH | 12335.526 Hz |
| FIDRES | 0.125003 Hz |
| AQ | 3.9999604 sec |
| RG | 40.3 |
| DW | 40.533 use |
| DE | 10.48 use |
| TE | 298.0 K |
| D1 | 1.00000000 sec |
| TD0 | 1 |
| ======== CHANNEL f1 ======= | |
| NUC1 | 1H |
| P1 | 11.40 use |
| PL1 | 1.00 dB |
| PL1W | 13.76731014 W |
| SFO1 | 600.1337061 MHz |
| SI | 32768 |
| SF | 600.1300116 MHz |
| WDW | EM |
| SSB | 0 |
| LB | 0.30 Hz |
| GB | 0 |
| PC | 1.40 |

V:BrukerTOPSPIN/mjp/JC-324-1/10/1
Integral regions #6 to #7

JC-324-1
PROTON.ucl CDCl3 {V:\Bruker\TOPSPIN} mjp 43

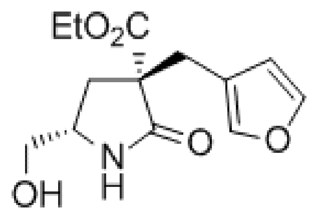


15a

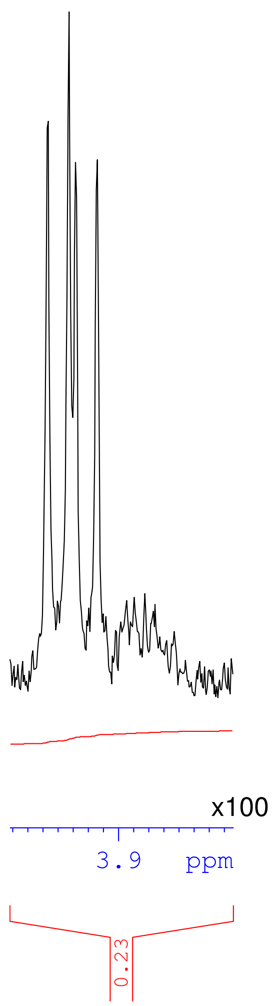


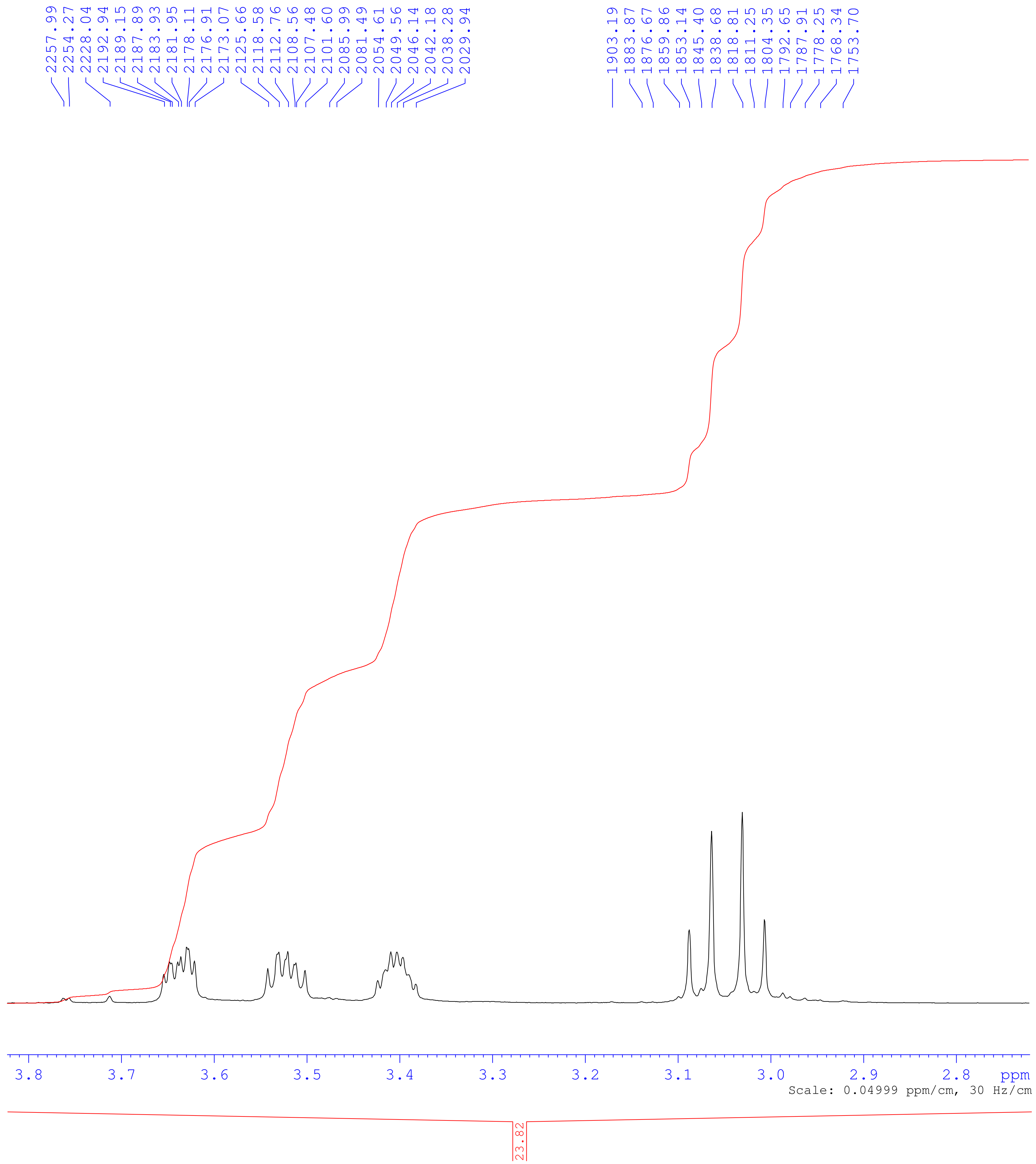

V:BrukerTOPSPIN/mjp/JC–324–1/10/1
Integral regions #8 to #8
30 Hz/cm

JC–324–1
PROTON.ucl CDCl3 {V:\Bruker\TOPSPIN} mjp 43

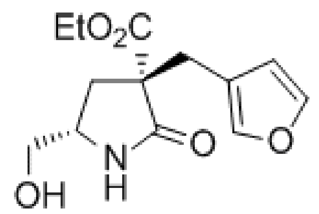


**15a**

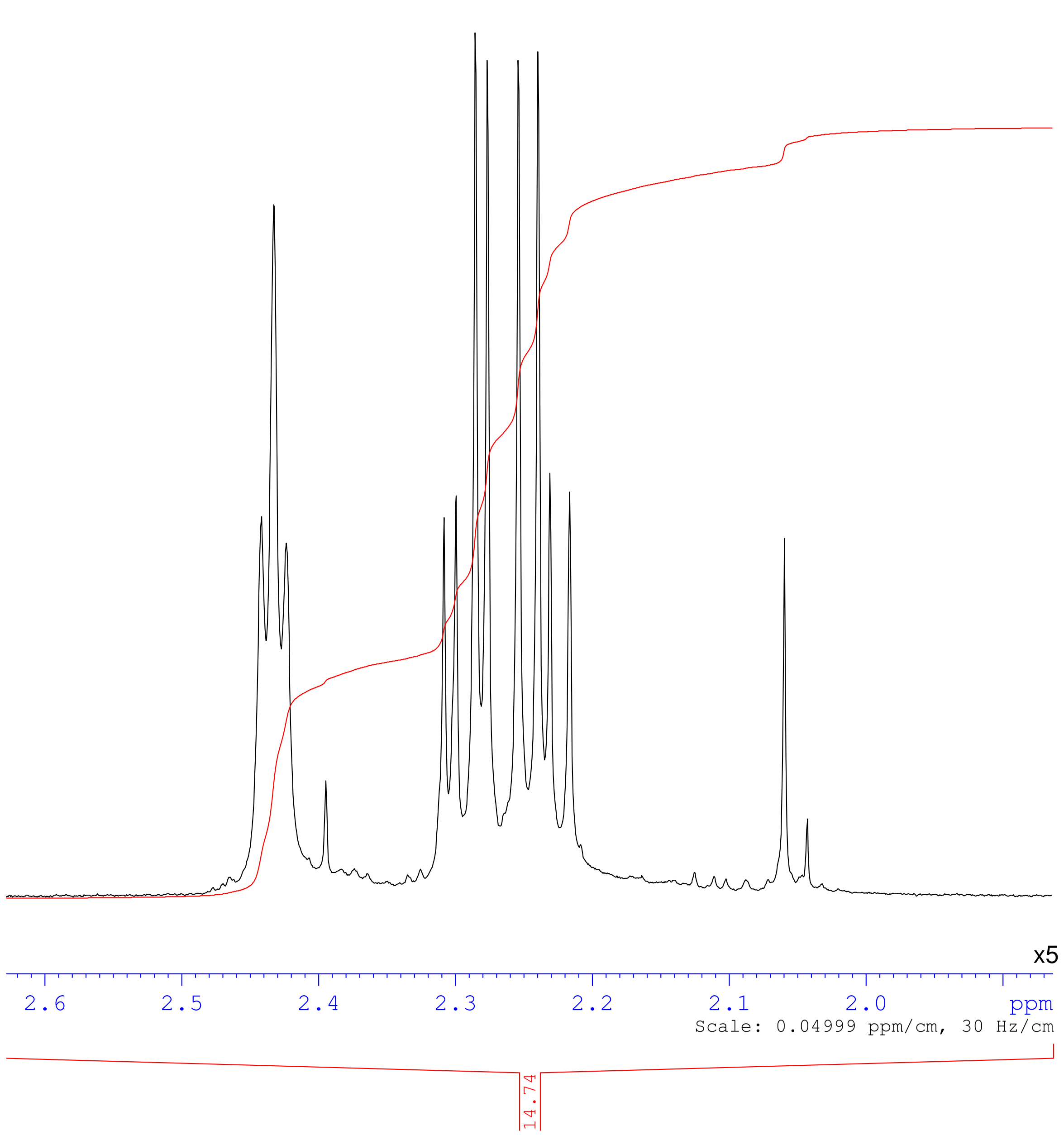

**JC−324−1**
**C13CPD.ucl CDCl3 {V:\Bruker\TOPSPIN} mjp 43**

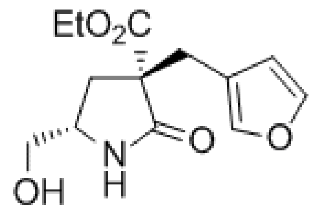

**15a**

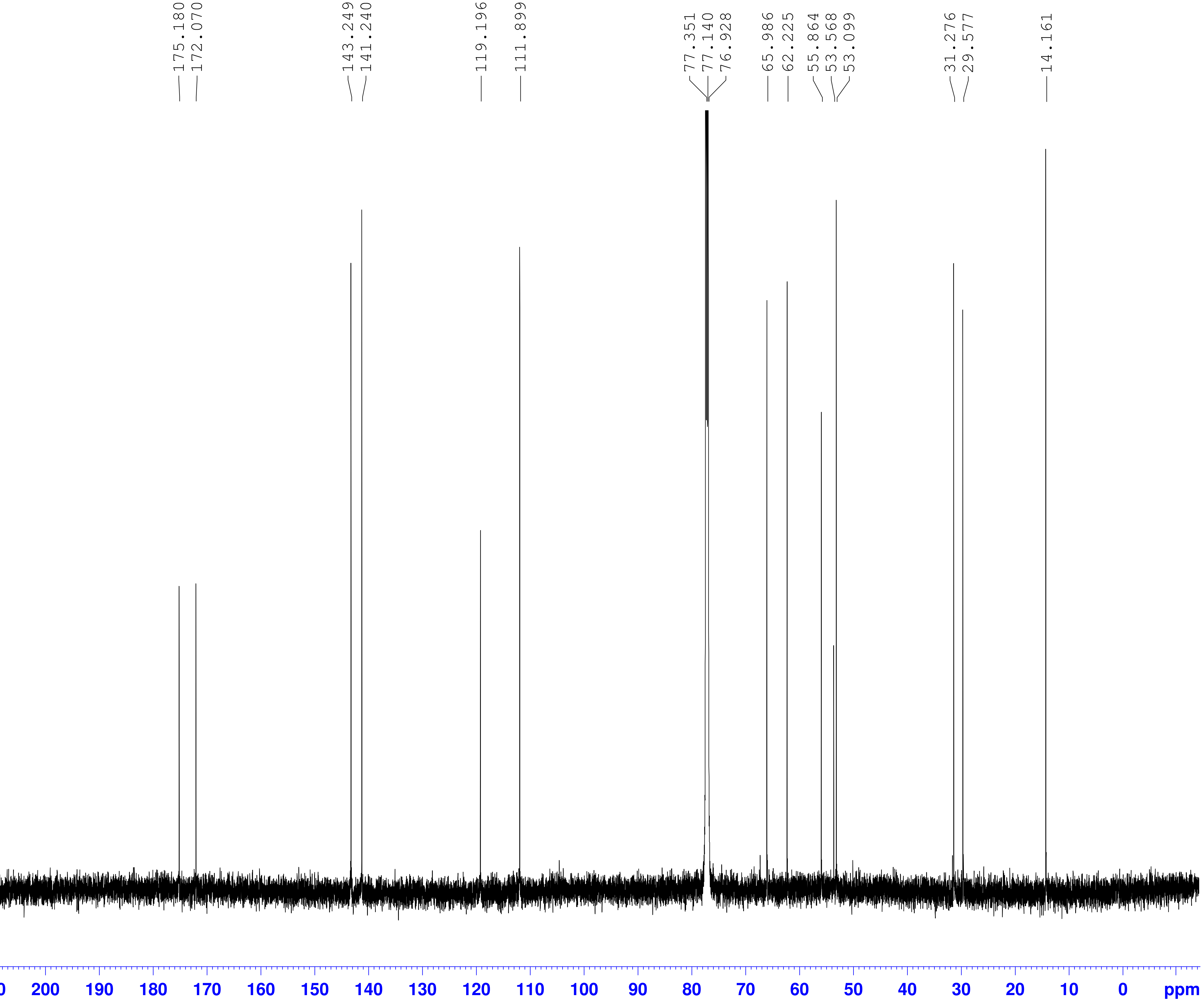


| | |
|---|---|
| NAME | JC-324-1 |
| EXPNO | 12 |
| PROCNO | 1 |
| Date_ | 20120317 |
| Time | 14.26 |
| INSTRUM | AV600 |
| PROBHD | 5 mm CPDCH 13C |
| PULPROG | zgpg30 |
| TD | 70308 |
| SOLVENT | CDCl3 |
| NS | 128 |
| DS | 0 |
| SWH | 39062.500 H |
| FIDRES | 0.555591 H |
| AQ | 0.8999924 s |
| RG | 1030 |
| DW | 12.800 u |
| DE | 21.12 u |
| TE | 298.0 K |
| D1 | 2.00000000 s |
| D11 | 0.03000000 s |
| TD0 | 1 |
| ======== CHANNEL f1 ===== | |
| NUC1 | 13C |
| P1 | 9.80 u |
| PL1 | 5.00 d |
| PL1W | 26.76886177 W |
| SFO1 | 150.9201628 M |
| ======== CHANNEL f2 ===== | |
| CPDPRG2 | waltz16 |
| NUC2 | 1H |
| PCPD2 | 70.00 u |
| PL2 | 1.00 d |
| PL12 | 17.23 d |
| PL13 | 20.00 d |
| PL2W | 13.76731014 W |
| PL12W | 0.32798135 W |
| PL13W | 0.17332016 W |
| SFO2 | 600.1324005 M |
| SI | 65536 |
| SF | 150.9027930 M |
| WDW | EM |
| SSB | 0 |
| LB | 1.00 H |
| GB | 0 |
| PC | 1.40 |

**JC–324–1**
**COSY.ucl CDCl3 {V:\Bruker\TOPSPIN} mjp 43**

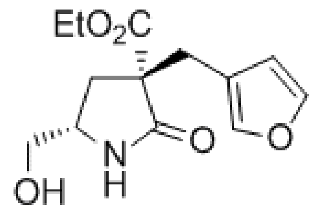


**15a**

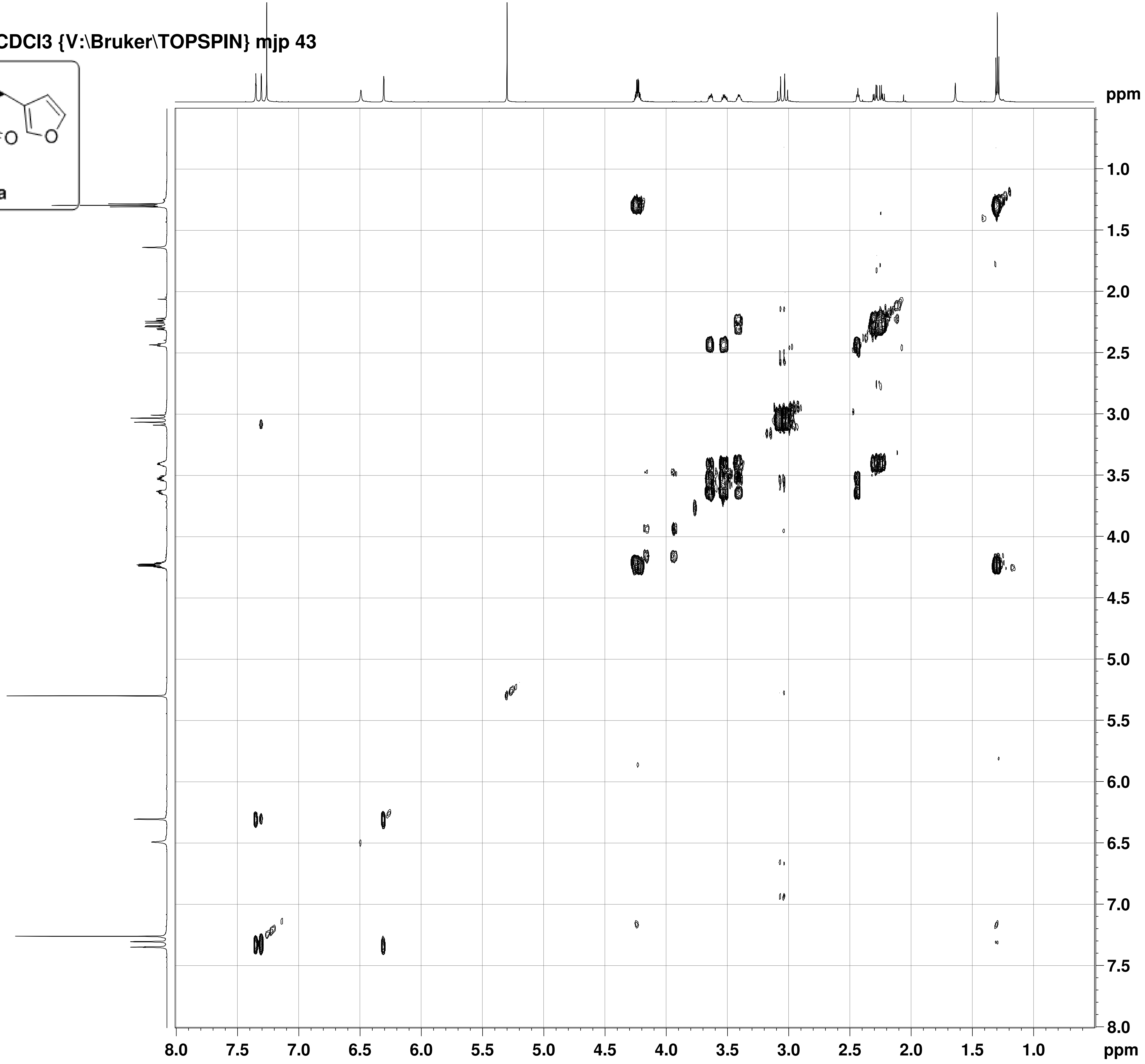


| | |
|---|---|
| NAME | JC-324-1 |
| EXPNO | 11 |
| PROCNO | 1 |
| Date_ | 20120317 |
| Time | 14.16 |
| INSTRUM | AV600 |
| PROBHD | 5 mm CPDCH 13C |
| PULPROG | cosygpmfqf |
| TD | 2048 |
| SOLVENT | CDCl3 |
| NS | 1 |
| DS | 8 |
| SWH | 4504.504 Hz |
| FIDRES | 2.199465 Hz |
| AQ | 0.2273780 sec |
| RG | 2050 |
| DW | 111.000 usec |
| DE | 6.50 usec |
| TE | 298.0 K |
| D0 | 0.00000300 sec |
| D1 | 1.67272902 sec |
| D13 | 0.00000400 sec |
| D16 | 0.00020000 sec |
| IN0 | 0.00022200 sec |
| ======== CHANNEL f1 ======== | |
| NUC1 | 1H |
| P1 | 11.40 usec |
| PL1 | 1.00 dB |
| PL1W | 13.76731014 W |
| SFO1 | 600.1325655 MHz |
| ====== GRADIENT CHANNEL ==== | |
| GPNAM1 | SINE.100 |
| GPNAM2 | SINE.100 |
| GPNAM3 | SINE.100 |
| GPZ1 | 16.00 % |
| GPZ2 | 12.00 % |
| GPZ3 | 40.00 % |
| P16 | 1000.00 usec |
| ND0 | 1 |
| TD | 128 |
| SFO1 | 600.1326 MHz |
| FIDRES | 35.191441 Hz |
| SW | 7.506 ppm |
| FnMODE | QF |
| SI | 1024 |
| SF | 600.1300096 MHz |
| WDW | QSINE |
| SSB | 0 |
| LB | 0.00 Hz |
| GB | 0 |
| PC | 1.40 |
| SI | 1024 |
| MC2 | QF |
| SF | 600.1300096 MHz |
| WDW | QSINE |
| SSB | 0 |
| LB | 0.00 Hz |
| GB | 0 |

**JC–324–1**
**C13DEPT135.ucl CDCl3 {V:\Bruker\TOPSPIN} mjp 43**

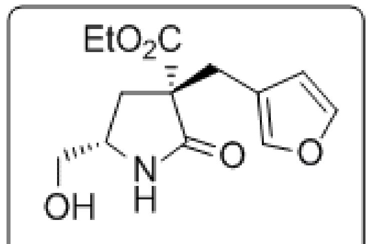


15a

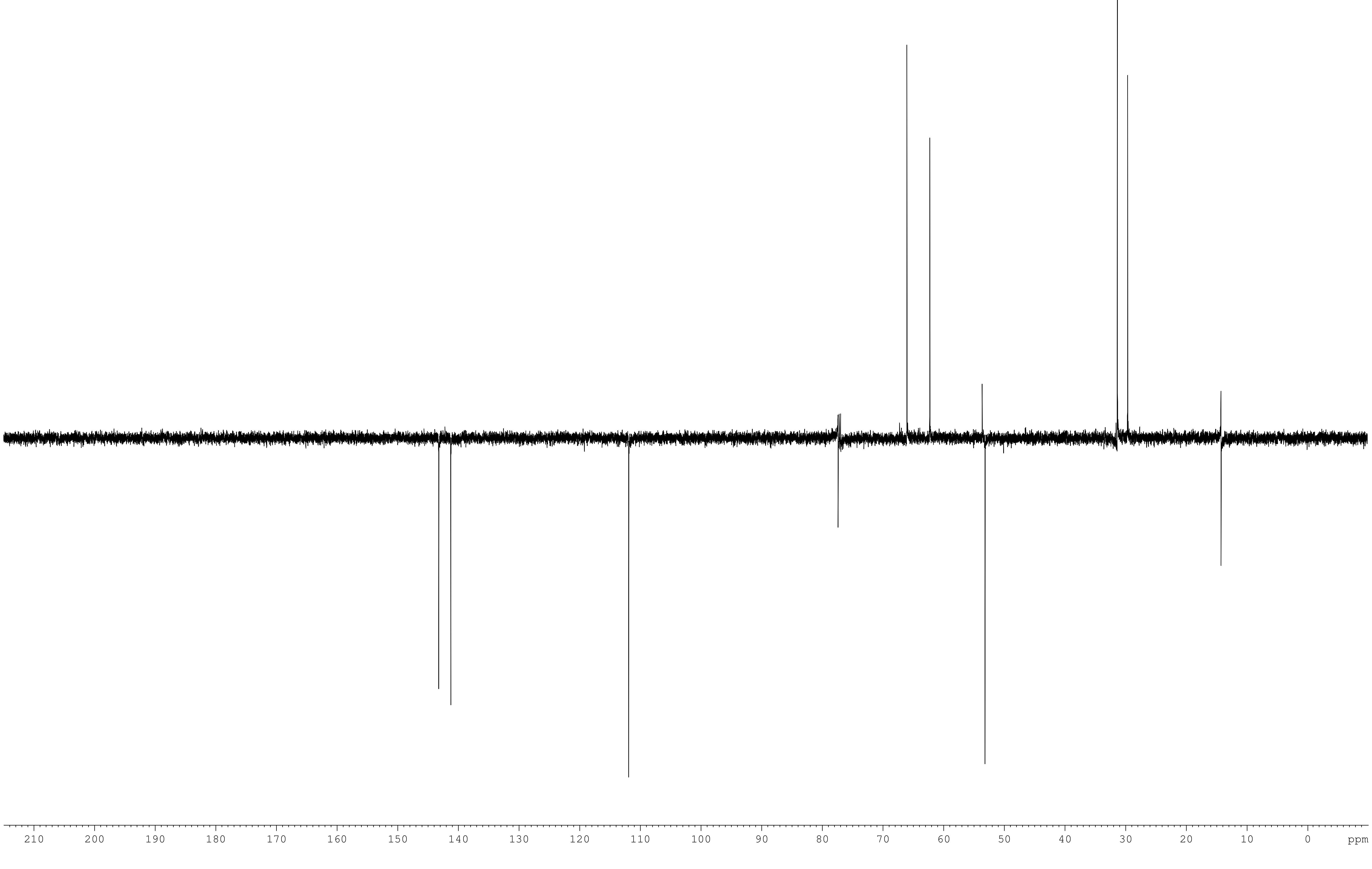


| | |
|---|---|
| NAME | JC-324-1 |
| EXPNO | 15 |
| PROCNO | 1 |
| Date_ | 20120317 |
| Time | 14.49 |
| INSTRUM | AV600 |
| PROBHD | 5 mm CPDCH 13C |
| PULPROG | dept135 |
| TD | 70308 |
| SOLVENT | CDCl3 |
| NS | 64 |
| DS | 4 |
| SWH | 39062.500 H |
| FIDRES | 0.555591 H |
| AQ | 0.8999924 s |
| RG | 256 |
| DW | 12.800 u |
| DE | 6.50 u |
| TE | 298.0 K |
| CNST2 | 145.0000000 |
| D1 | 2.00000000 s |
| D2 | 0.00344828 s |
| D12 | 0.00002000 s |
| TD0 | 1 |

======== CHANNEL f1 =====

| | |
|---|---|
| NUC1 | 13C |
| P1 | 9.80 u |
| P2 | 19.60 u |
| PL1 | 5.00 d |
| PL1W | 26.76886177 W |
| SFO1 | 150.9201628 M |

======== CHANNEL f2 =====

| | |
|---|---|
| CPDPRG2 | waltz16 |
| NUC2 | 1H |
| P3 | 10.80 u |
| P4 | 21.60 u |
| PCPD2 | 70.00 u |
| PL2 | 1.00 d |
| PL12 | 17.23 d |
| PL2W | 13.76731014 W |
| PL12W | 0.32798135 W |
| SFO2 | 600.1324005 M |
| SI | 65536 |
| SF | 150.9027930 M |
| WDW | EM |
| SSB | 0 |
| LB | 1.00 H |
| GB | 0 |
| PC | 1.40 |

JC–324–1
HSQC.ucl CDCl3 {V:\Bruker\TOPSPIN} mjp 43

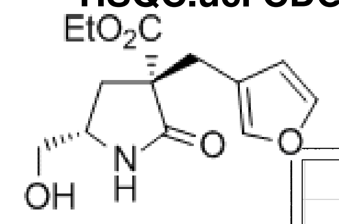


**15a**

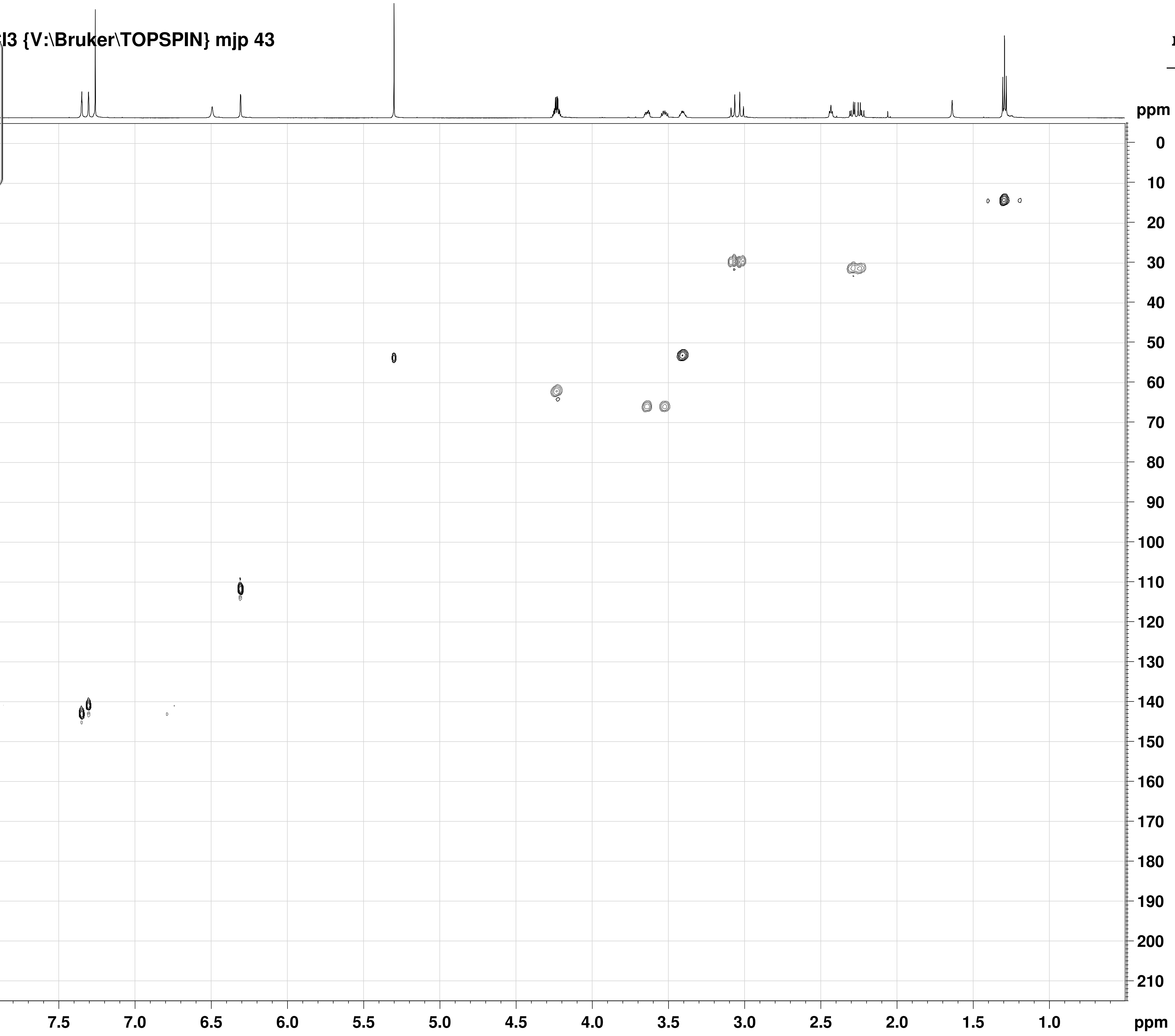


```
NAME              JC-324-1
EXPNO                   13
PROCNO                   1
Date_             20120317
Time                 14.28
INSTRUM              AV600
PROBHD   5 mm CPDCH 13C
PULPROG  hsqcedetgpsisp2.4
TD                    1024
SOLVENT              CDCl3
NS                       2
DS                      32
SWH           4504.504 Hz
FIDRES        4.398930 Hz
AQ           0.1137140 sec
RG                    2050
DW             111.000 usec
DE               6.50 usec
TE               298.0 K
CNST2      145.0000000
CNST17      -0.5000000
D0          0.00000300 sec
D1          1.47153294 sec
D2          0.00344828 sec
D4          0.00172414 sec
D11         0.03000000 sec
D16         0.00020000 sec
D21         0.00344828 sec
D24         0.00086207 sec
IN0         0.00001505 sec
L0                       0
L31                      1
TD0                      2

======== CHANNEL f1 ========
NUC1                    1H
P1                11.40 usec
P2                22.80 usec
P28                0.00 usec
PL1                1.00 dB
PL1W        13.76731014 W
SFO1        600.1325655 MHz

======== CHANNEL f2 ========
CPDPRG2   bi_p5m4sp_4sp.2
NUC2                   13C
P3                 9.80 usec
P14              500.00 usec
P24             2000.00 usec
P31             1730.00 usec
P63             1500.00 usec
PL0              120.00 dB
PL2                5.00 dB
PL12              20.74 dB
PL0W         0.00000000 W
PL2W        26.76886177 W
PL12W        0.71388775 W
SFO2        150.9186538 MHz
SP3               13.33 dB
SP7               13.33 dB
SP14              14.82 dB
SP18              18.73 dB
SP31              20.84 dB
SPNAM3    Crp60,0.5,20.1
SPNAM7       Crp60comp.4
SPNAM14   Crp32,1.5,20.2
SPNAM18    Crp60_xfilt.2
SPNAM31   Crp32,1.5,20.2
SPOAL3               0.500
SPOAL7               0.500
SPOAL14              0.500
SPOAL18              0.500
SPOAL31              0.500
SPOFFS3            0.00 Hz
SPOFFS7            0.00 Hz
SPOFFS14           0.00 Hz
SPOFFS18           0.00 Hz
SPOFFS31           0.00 Hz

====== GRADIENT CHANNEL =====
GPNAM1            SINE.100
GPNAM2            SINE.100
GPNAM3            SINE.100
GPNAM4            SINE.100
GPZ1               80.00 %
GPZ2               20.10 %
GPZ3               11.00 %
GPZ4               -5.00 %
P16              1000.00 usec
P19               600.00 usec
ND0                      2
TD                     128
SFO1          150.9187 MHz
FIDRES      259.391449 Hz
SW             220.000 ppm
FnMODE      Echo-Antiecho
SI                    1024
SF          600.1300096 MHz
WDW                  QSINE
SSB                      2
LB                 0.00 Hz
GB                       0
PC                    1.40
SI                    1024
MC2           echo-antiecho
SF          150.9027771 MHz
WDW                  QSINE
SSB                      2
LB                 0.00 Hz
GB                       0
```

**JC–324–1**
**HMBC.ucl CDCl3 {V:\Bruker\TOPSPIN} mjp 43**

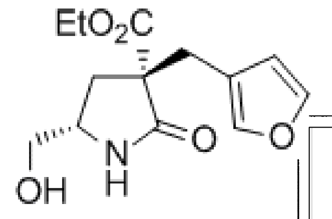

**15a**

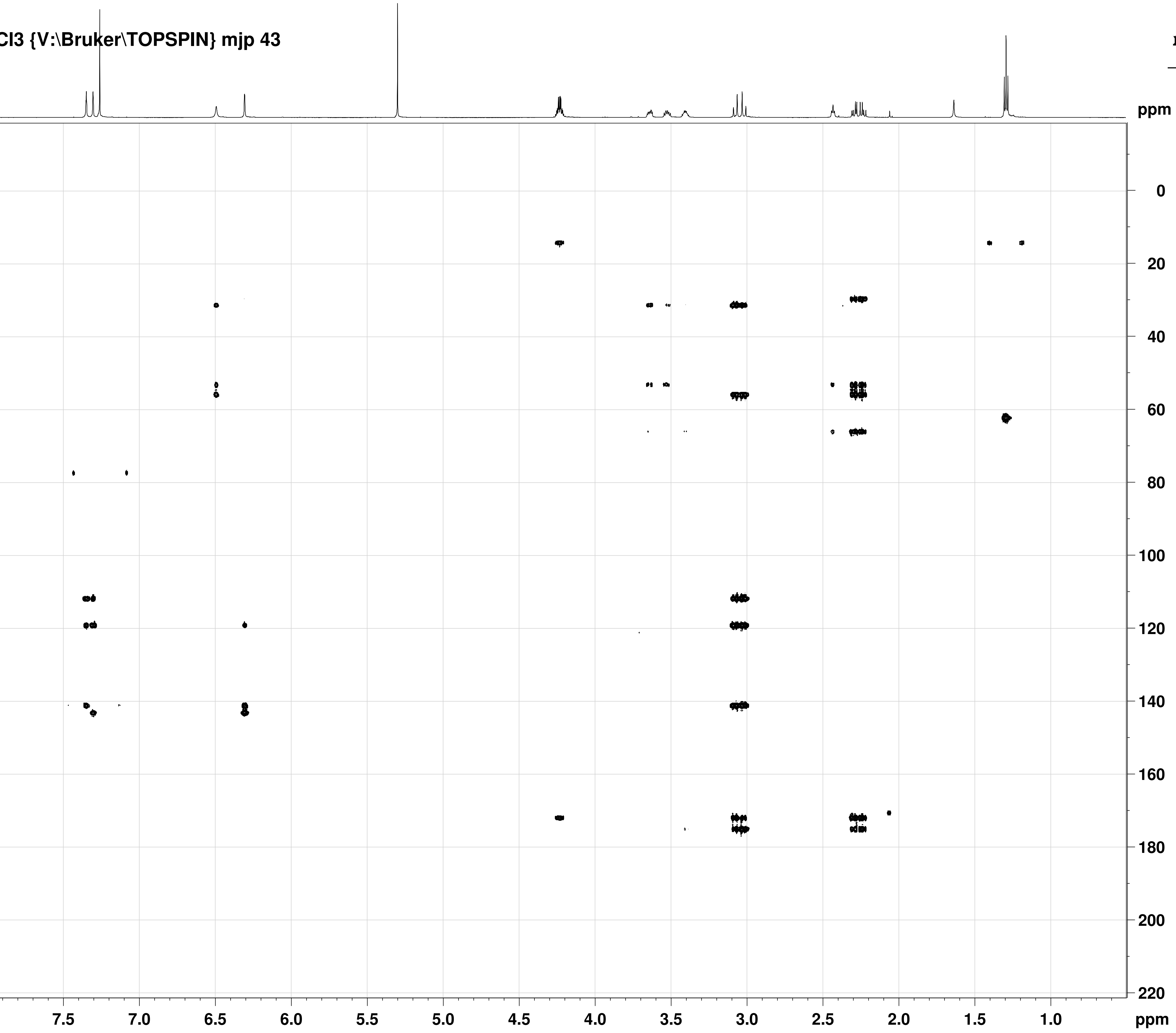


| | |
|---|---|
| NAME | JC-324-1 |
| EXPNO | 14 |
| PROCNO | 1 |
| Date_ | 20120317 |
| Time | 14.35 |
| INSTRUM | AV600 |
| PROBHD | 5 mm CPDCH 13C |
| PULPROG | hmbcetgpl3nd |
| TD | 4096 |
| SOLVENT | CDCl3 |
| NS | 2 |
| DS | 16 |
| SWH | 4504.504 Hz |
| FIDRES | 1.099733 Hz |
| AQ | 0.4547060 sec |
| RG | 2050 |
| DW | 111.000 usec |
| DE | 6.50 usec |
| TE | 298.0 K |
| CNST6 | 120.0000000 |
| CNST7 | 160.0000000 |
| CNST13 | 10.0000000 |
| CNST30 | 0.5981151 |
| D0 | 0.00000300 sec |
| D1 | 0.72313857 sec |
| D6 | 0.05000000 sec |
| D16 | 0.00020000 sec |
| IN0 | 0.00001380 sec |
| ======== CHANNEL f1 ======== | |
| NUC1 | 1H |
| P1 | 11.40 usec |
| P2 | 22.80 usec |
| PL1 | 1.00 dB |
| PL1W | 13.76731014 W |
| SFO1 | 600.1325655 MHz |
| ======== CHANNEL f2 ======== | |
| NUC2 | 13C |
| P3 | 9.80 usec |
| P24 | 2000.00 usec |
| PL2 | 5.00 dB |
| PL2W | 26.76886177 W |
| SFO2 | 150.9178993 MHz |
| SP7 | 13.33 dB |
| SPNAM7 | Crp60comp.4 |
| SPOAL7 | 0.500 |
| SPOFFS7 | 0.00 Hz |
| ====== GRADIENT CHANNEL ===== | |
| GPNAM1 | SINE.100 |
| GPNAM3 | SINE.100 |
| GPNAM4 | SINE.100 |
| GPNAM5 | SINE.100 |
| GPNAM6 | SINE.100 |
| GPZ1 | 80.00 % |
| GPZ3 | 14.00 % |
| GPZ4 | -8.00 % |
| GPZ5 | -4.00 % |
| GPZ6 | -2.00 % |
| P16 | 1000.00 usec |
| ND0 | 2 |
| TD | 256 |
| SFO1 | 150.9179 MHz |
| FIDRES | 141.485535 Hz |
| SW | 240.000 ppm |
| FnMODE | Echo-Antiecho |
| SI | 2048 |
| SF | 600.1300107 MHz |
| WDW | SINE |
| SSB | 2 |
| LB | 0.00 Hz |
| GB | 0 |
| PC | 1.40 |
| SI | 1024 |
| MC2 | echo-antiecho |
| SF | 150.9027756 MHz |
| WDW | SINE |
| SSB | 2 |
| LB | 0.00 Hz |
| GB | 0 |

JC–326–2
PROTON.ucl CDCl3 {V:\Bruker\TOPSPIN} mjp 59

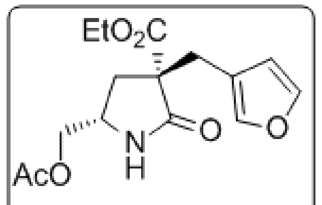


15b

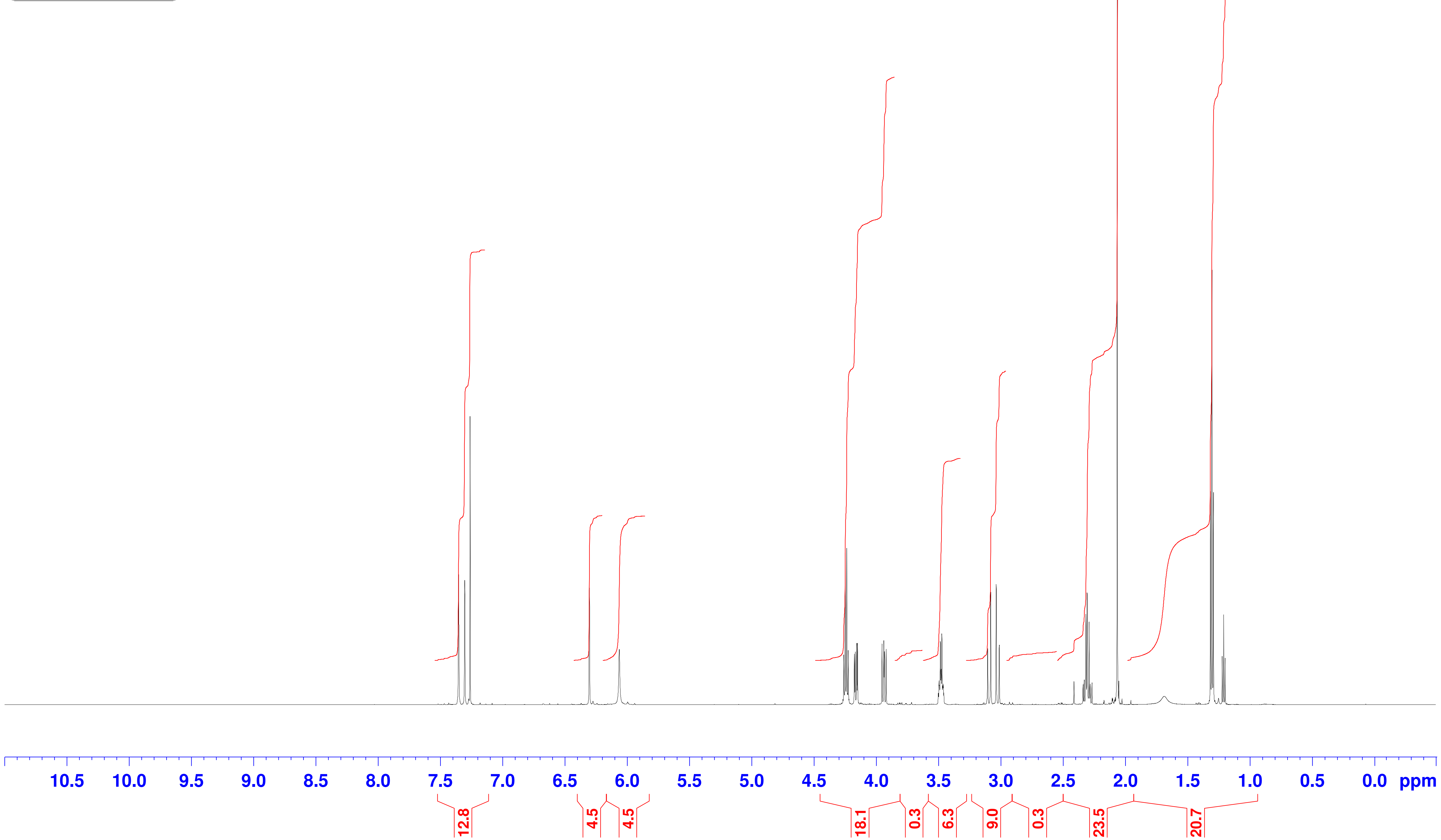


| | |
|---|---|
| NAME | JC-326-2 |
| EXPNO | 10 |
| PROCNO | 1 |
| Date_ | 20120130 |
| Time | 17.02 |
| INSTRUM | AV600 |
| PROBHD | 5 mm CPDCH 13C |
| PULPROG | zg30 |
| TD | 98682 |
| SOLVENT | CDCl3 |
| NS | 8 |
| DS | 0 |
| SWH | 12335.526 Hz |
| FIDRES | 0.125003 Hz |
| AQ | 3.9999604 sec |
| RG | 36 |
| DW | 40.533 use |
| DE | 10.48 use |
| TE | 298.0 K |
| D1 | 1.00000000 sec |
| TD0 | 1 |

======== CHANNEL f1 ========

| | |
|---|---|
| NUC1 | 1H |
| P1 | 11.40 use |
| PL1 | 1.00 dB |
| PL1W | 13.76731014 W |
| SFO1 | 600.1337061 MHz |
| SI | 32768 |
| SF | 600.1300116 MHz |
| WDW | EM |
| SSB | 0 |
| LB | 0.30 Hz |
| GB | 0 |
| PC | 1.40 |

**JC–326–2**
**C13CPD.ucl CDCl3 {V:\Bruker\TOPSPIN} mjp 59**

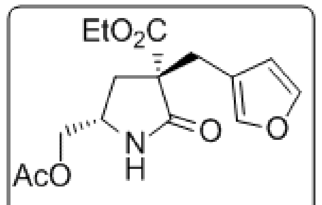


**15b**

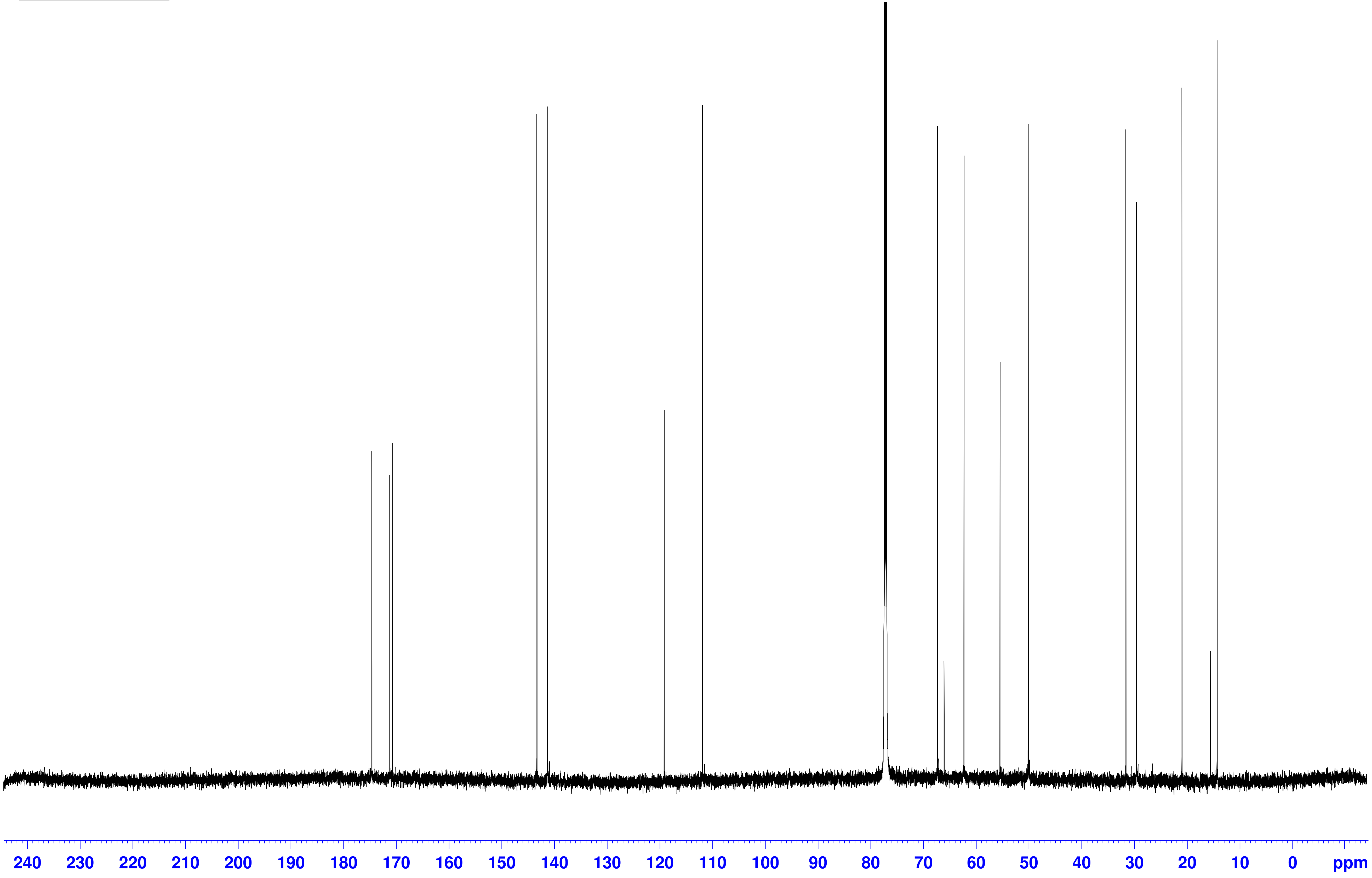


UCL

| | |
|---|---|
| NAME | JC-326-2 |
| EXPNO | 12 |
| PROCNO | 1 |
| Date_ | 20120130 |
| Time | 17.20 |
| INSTRUM | AV600 |
| PROBHD | 5 mm CPDCH 13C |
| PULPROG | zgpg30 |
| TD | 70308 |
| SOLVENT | CDCl3 |
| NS | 256 |
| DS | 0 |
| SWH | 39062.500 Hz |
| FIDRES | 0.555591 Hz |
| AQ | 0.8999924 sec |
| RG | 1030 |
| DW | 12.800 usec |
| DE | 21.12 usec |
| TE | 298.0 K |
| D1 | 2.00000000 sec |
| D11 | 0.03000000 sec |
| TD0 | 1 |
| ======== CHANNEL f1 ===== | |
| NUC1 | 13C |
| P1 | 9.80 usec |
| PL1 | 5.00 dB |
| PL1W | 26.76886177 W |
| SFO1 | 150.9201628 MHz |
| ======== CHANNEL f2 ===== | |
| CPDPRG2 | waltz16 |
| NUC2 | 1H |
| PCPD2 | 70.00 usec |
| PL2 | 1.00 dB |
| PL12 | 17.23 dB |
| PL13 | 20.00 dB |
| PL2W | 13.76731014 W |
| PL12W | 0.32798135 W |
| PL13W | 0.17332016 W |
| SFO2 | 600.1324005 MHz |
| SI | 65536 |
| SF | 150.9027930 MHz |
| WDW | EM |
| SSB | 0 |
| LB | 1.00 Hz |
| GB | 0 |
| PC | 1.40 |

JC–326–2
COSY.ucl CDCl3 {V:\Bruker\TOPSPIN} mjp 59

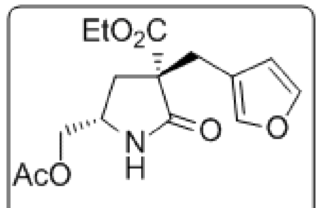


15b

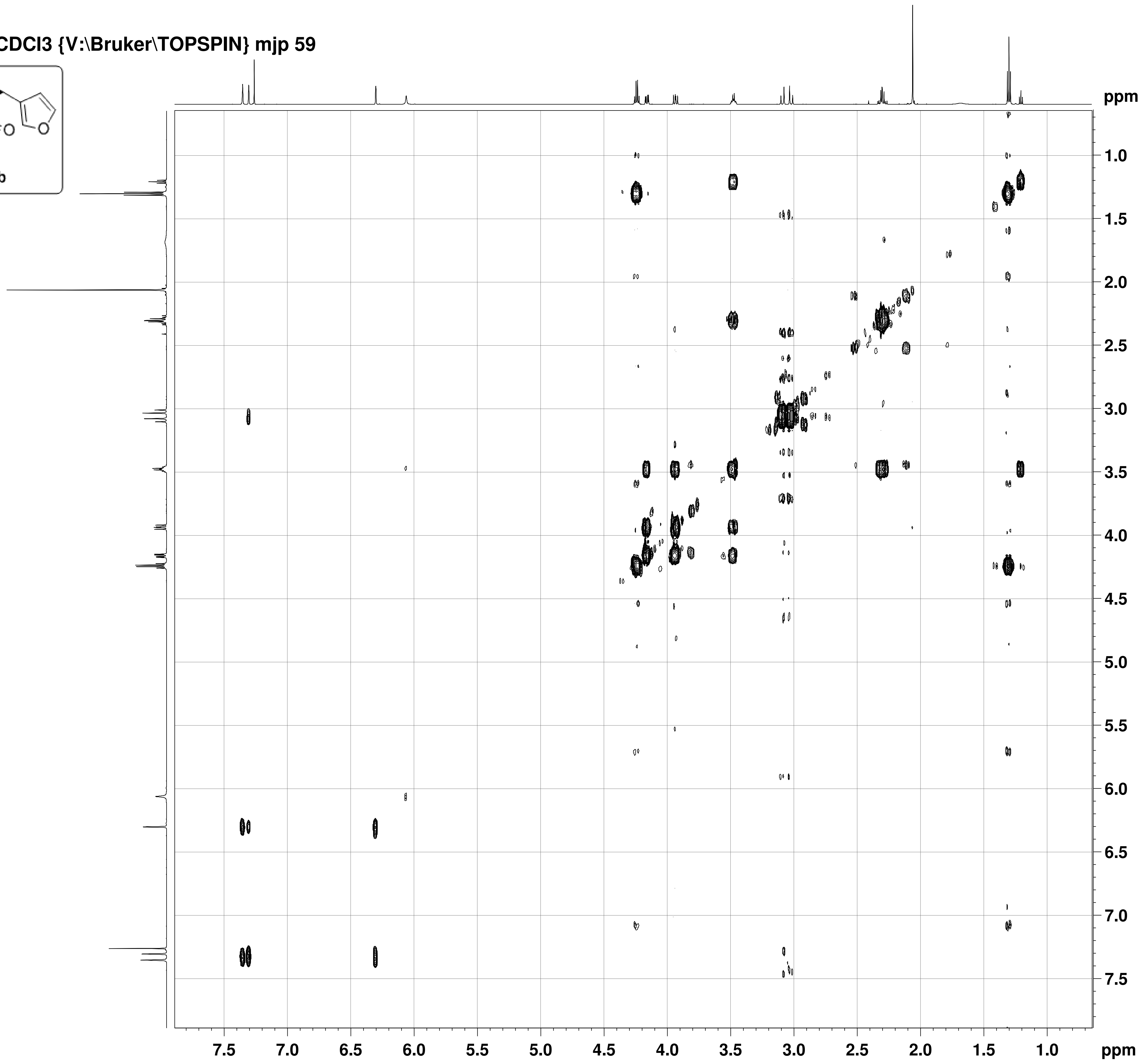


UCL

| | |
|---|---|
| NAME | JC-326-2 |
| EXPNO | 11 |
| PROCNO | 1 |
| Date_ | 20120130 |
| Time | 17.02 |
| INSTRUM | AV600 |
| PROBHD | 5 mm CPDCH 13C |
| PULPROG | cosygpmfqf |
| TD | 2048 |
| SOLVENT | CDCl3 |
| NS | 1 |
| DS | 8 |
| SWH | 4347.826 Hz |
| FIDRES | 2.122962 Hz |
| AQ | 0.2355700 sec |
| RG | 2050 |
| DW | 115.000 usec |
| DE | 6.50 usec |
| TE | 298.0 K |
| D0 | 0.00000300 sec |
| D1 | 1.66453695 sec |
| D13 | 0.00000400 sec |
| D16 | 0.00020000 sec |
| IN0 | 0.00023000 sec |
| ======== CHANNEL f1 ======== | |
| NUC1 | 1H |
| P1 | 11.40 usec |
| PL1 | 1.00 dB |
| PL1W | 13.76731014 W |
| SFO1 | 600.1325723 MHz |
| ====== GRADIENT CHANNEL ==== | |
| GPNAM1 | SINE.100 |
| GPNAM2 | SINE.100 |
| GPNAM3 | SINE.100 |
| GPZ1 | 16.00 % |
| GPZ2 | 12.00 % |
| GPZ3 | 40.00 % |
| P16 | 1000.00 usec |
| ND0 | 1 |
| TD | 128 |
| SFO1 | 600.1326 MHz |
| FIDRES | 33.967392 Hz |
| SW | 7.245 ppm |
| FnMODE | QF |
| SI | 1024 |
| SF | 600.1300097 MHz |
| WDW | QSINE |
| SSB | 0 |
| LB | 0.00 Hz |
| GB | 0 |
| PC | 1.40 |
| SI | 1024 |
| MC2 | QF |
| SF | 600.1300097 MHz |
| WDW | QSINE |
| SSB | 0 |
| LB | 0.00 Hz |
| GB | 0 |

# JC–326–2
## NOESY.ucl CDCl3 {V:\Bruker\TOPSPIN} mjp 59

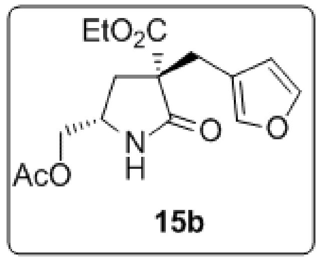

**15b**

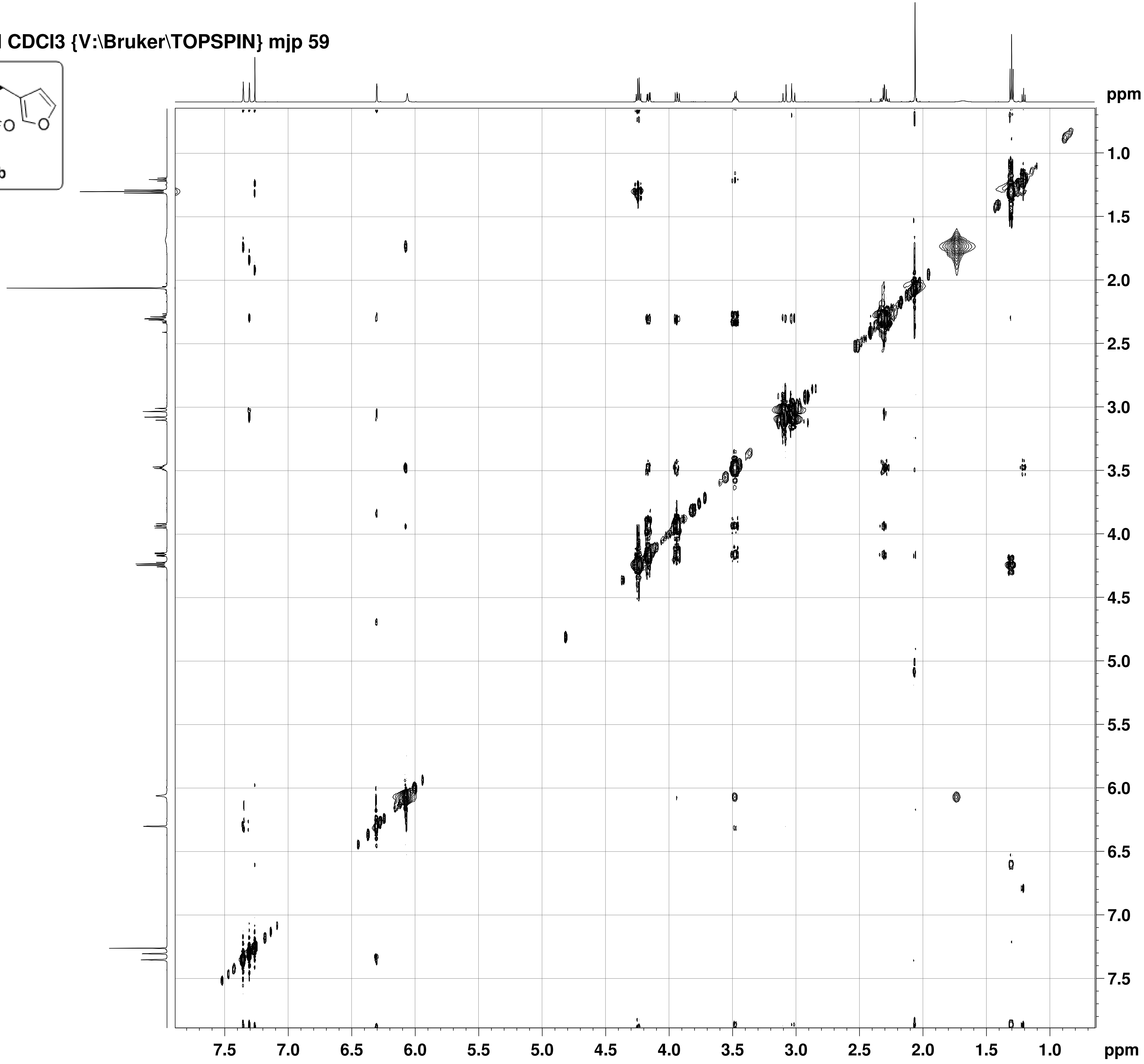


| | |
|---|---|
| NAME | JC-326-2 |
| EXPNO | 16 |
| PROCNO | 1 |
| Date_ | 20120130 |
| Time | 22.53 |
| INSTRUM | AV600 |
| PROBHD | 5 mm CPDCH 13C |
| PULPROG | noesygpph |
| TD | 2048 |
| SOLVENT | CDCl3 |
| NS | 2 |
| DS | 16 |
| SWH | 4347.826 Hz |
| FIDRES | 2.122962 Hz |
| AQ | 0.2355700 sec |
| RG | 45.2 |
| DW | 115.000 usec |
| DE | 6.50 usec |
| TE | 298.0 K |
| D0 | 0.00010125 sec |
| D1 | 1.95371497 sec |
| D8 | 0.60000002 sec |
| D16 | 0.00020000 sec |
| IN0 | 0.00023000 sec |
| ======== CHANNEL f1 ======== | |
| NUC1 | 1H |
| P1 | 10.80 usec |
| P2 | 21.60 usec |
| PL1 | 1.00 dB |
| PL1W | 13.76731014 W |
| SFO1 | 600.1325723 MHz |
| ====== GRADIENT CHANNEL ==== | |
| GPNAM1 | SINE.100 |
| GPNAM2 | SINE.100 |
| GPZ1 | 40.00 % |
| GPZ2 | -40.00 % |
| P16 | 1000.00 usec |
| ND0 | 1 |
| TD | 256 |
| SFO1 | 600.1326 MHz |
| FIDRES | 16.983696 Hz |
| SW | 7.245 ppm |
| FnMODE | States-TPPI |
| SI | 1024 |
| SF | 600.1300097 MHz |
| WDW | QSINE |
| SSB | 2 |
| LB | 0.00 Hz |
| GB | 0 |
| PC | 1.00 |
| SI | 1024 |
| MC2 | States-TPPI |
| SF | 600.1300097 MHz |
| WDW | QSINE |
| SSB | 2 |
| LB | 0.00 Hz |
| GB | 0 |

**JC–326–2**
**C13DEPT135.ucl CDCl3 {V:\Bruker\TOPSPIN} mjp 59**

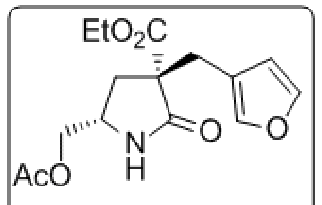

15b

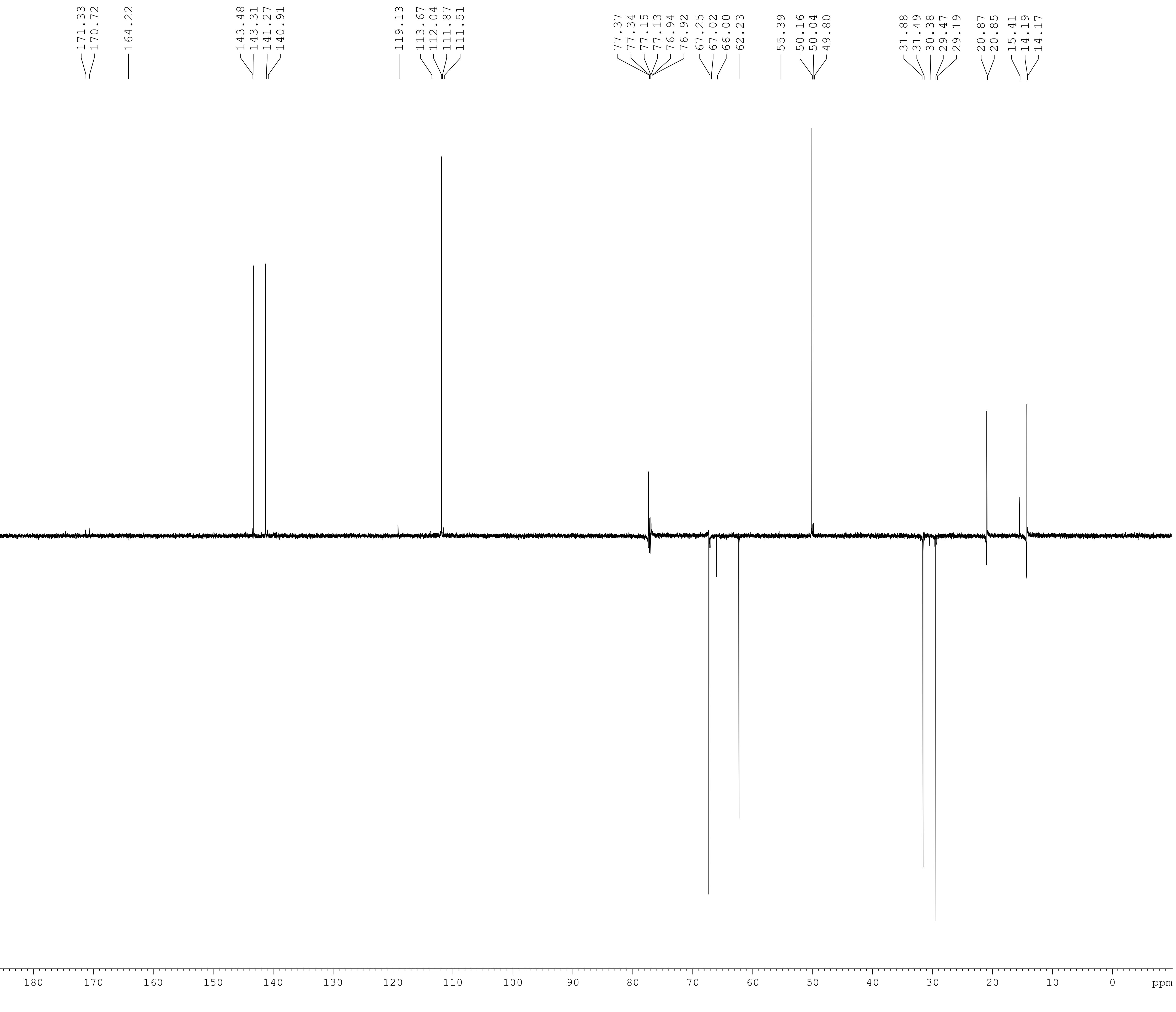


UCL

| | |
|---|---|
| NAME | JC-326-2 |
| EXPNO | 15 |
| PROCNO | 1 |
| Date_ | 20120130 |
| Time | 22.52 |
| INSTRUM | AV600 |
| PROBHD | 5 mm CPDCH 13C |
| PULPROG | dept135 |
| TD | 70308 |
| SOLVENT | CDCl3 |
| NS | 256 |
| DS | 4 |
| SWH | 39062.500 H |
| FIDRES | 0.555591 H |
| AQ | 0.8999924 s |
| RG | 256 |
| DW | 12.800 u |
| DE | 6.50 u |
| TE | 298.0 K |
| CNST2 | 145.0000000 |
| D1 | 2.00000000 s |
| D2 | 0.00344828 s |
| D12 | 0.00002000 s |
| TD0 | 1 |
| ======== CHANNEL f1 ===== | |
| NUC1 | 13C |
| P1 | 9.80 u |
| P2 | 19.60 u |
| PL1 | 5.00 d |
| PL1W | 26.76886177 W |
| SFO1 | 150.9201628 M |
| ======== CHANNEL f2 ===== | |
| CPDPRG2 | waltz16 |
| NUC2 | 1H |
| P3 | 10.80 u |
| P4 | 21.60 u |
| PCPD2 | 70.00 u |
| PL2 | 1.00 d |
| PL12 | 17.23 d |
| PL2W | 13.76731014 W |
| PL12W | 0.32798135 W |
| SFO2 | 600.1324005 M |
| SI | 65536 |
| SF | 150.9027930 M |
| WDW | EM |
| SSB | 0 |
| LB | 1.00 H |
| GB | 0 |
| PC | 1.40 |

JC–326–2
HSQC.ucl CDCl3 {V:\Bruker\TOPSPIN} mjp 59

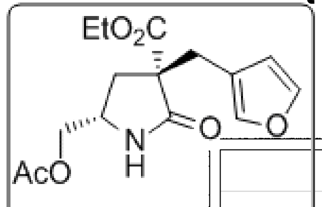


15b

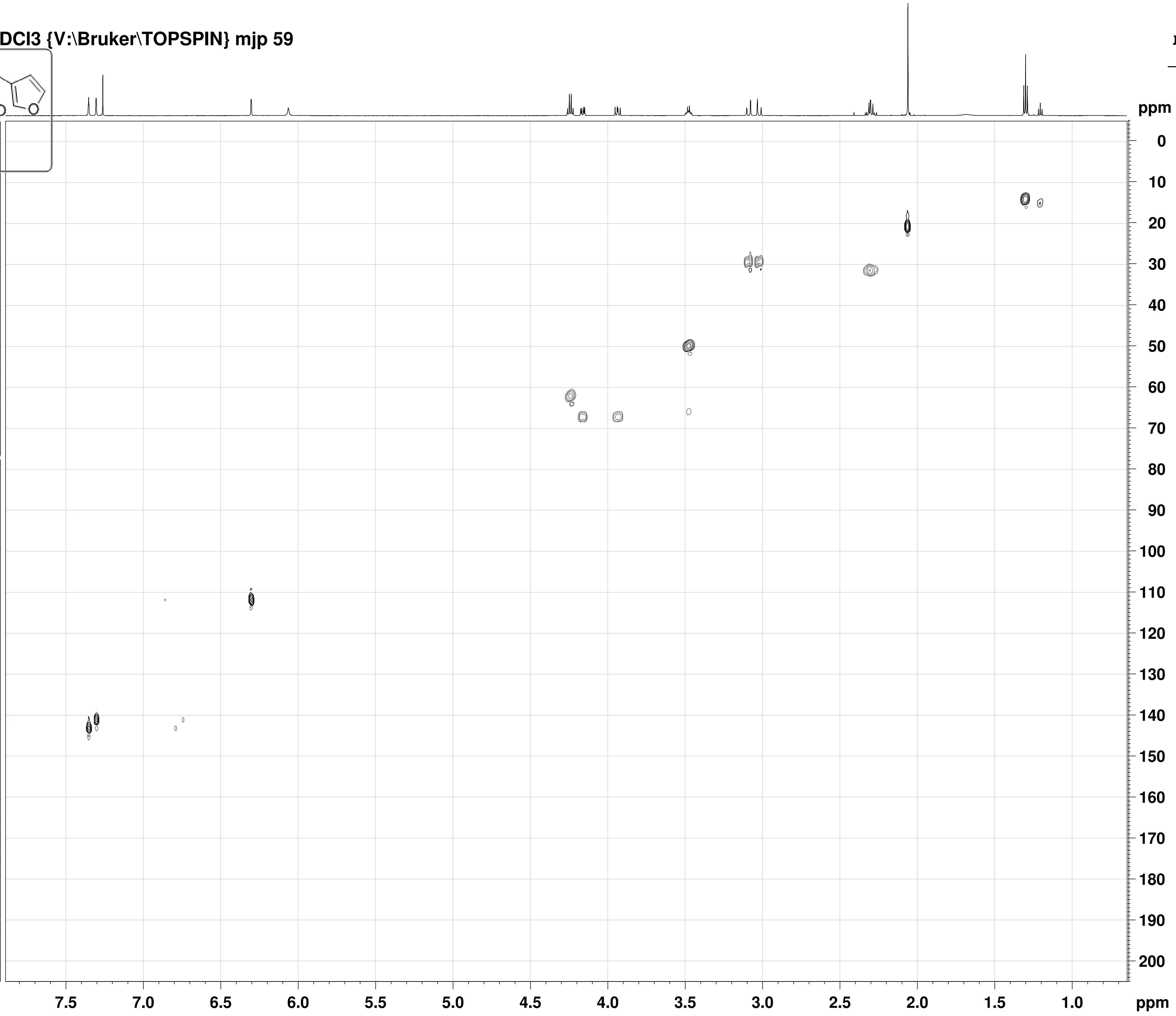


| | |
|---|---|
| NAME | JC-326-2 |
| EXPNO | 13 |
| PROCNO | 1 |
| Date_ | 20120130 |
| Time | 17.43 |
| INSTRUM | AV600 |
| PROBHD | 5 mm CPDCH 13C |
| PULPROG | hsqcedetgpsisp2.4 |
| TD | 1024 |
| SOLVENT | CDCl3 |
| NS | 2 |
| DS | 32 |
| SWH | 4347.826 Hz |
| FIDRES | 4.245924 Hz |
| AQ | 0.1178100 sec |
| RG | 2050 |
| DW | 115.000 usec |
| DE | 6.50 usec |
| TE | 298.0 K |
| CNST2 | 145.0000000 |
| CNST17 | -0.5000000 |
| D0 | 0.00000300 sec |
| D1 | 1.46743703 sec |
| D2 | 0.00344828 sec |
| D4 | 0.00172414 sec |
| D11 | 0.03000000 sec |
| D16 | 0.00020000 sec |
| D21 | 0.00344828 sec |
| D24 | 0.00086207 sec |
| IN0 | 0.00001580 sec |
| L0 | 0 |
| L31 | 1 |
| TD0 | 2 |
| ======== CHANNEL f1 ======== | |
| NUC1 | 1H |
| P1 | 11.40 usec |
| P2 | 22.80 usec |
| P28 | 0.00 usec |
| PL1 | 1.00 dB |
| PL1W | 13.76731014 W |
| SFO1 | 600.1325723 MHz |
| ======== CHANNEL f2 ======== | |
| CPDPRG2 | bi_p5m4sp_4sp.2 |
| NUC2 | 13C |
| P3 | 9.80 usec |
| P14 | 500.00 usec |
| P24 | 2000.00 usec |
| P31 | 1730.00 usec |
| P63 | 1500.00 usec |
| PL0 | 120.00 dB |
| PL2 | 5.00 dB |
| PL12 | 20.74 dB |
| PL0W | 0.00000000 W |
| PL2W | 26.76886177 W |
| PL12W | 0.71388775 W |
| SFO2 | 150.9178993 MHz |
| SP3 | 13.33 dB |
| SP7 | 13.33 dB |
| SP14 | 14.82 dB |
| SP18 | 18.73 dB |
| SP31 | 20.84 dB |
| SPNAM3 | Crp60,0.5,20.1 |
| SPNAM7 | Crp60comp.4 |
| SPNAM14 | Crp32,1.5,20.2 |
| SPNAM18 | Crp60_xfilt.2 |
| SPNAM31 | Crp32,1.5,20.2 |
| SPOAL3 | 0.500 |
| SPOAL7 | 0.500 |
| SPOAL14 | 0.500 |
| SPOAL18 | 0.500 |
| SPOAL31 | 0.500 |
| SPOFFS3 | 0.00 Hz |
| SPOFFS7 | 0.00 Hz |
| SPOFFS14 | 0.00 Hz |
| SPOFFS18 | 0.00 Hz |
| SPOFFS31 | 0.00 Hz |
| ====== GRADIENT CHANNEL ===== | |
| GPNAM1 | SINE.100 |
| GPNAM2 | SINE.100 |
| GPNAM3 | SINE.100 |
| GPNAM4 | SINE.100 |
| GPZ1 | 80.00 % |
| GPZ2 | 20.10 % |
| GPZ3 | 11.00 % |
| GPZ4 | -5.00 % |
| P16 | 1000.00 usec |
| P19 | 600.00 usec |
| ND0 | 2 |
| TD | 128 |
| SFO1 | 150.9179 MHz |
| FIDRES | 247.599686 Hz |
| SW | 210.000 ppm |
| FnMODE | Echo-Antiecho |
| SI | 1024 |
| SF | 600.1300097 MHz |
| WDW | QSINE |
| SSB | 2 |
| LB | 0.00 Hz |
| GB | 0 |
| PC | 1.40 |
| SI | 1024 |
| MC2 | echo-antiecho |
| SF | 150.9027778 MHz |
| WDW | QSINE |
| SSB | 2 |
| LB | 0.00 Hz |
| GB | 0 |

JC–326–2
HMBC.ucl CDCl3 {V:\Bruker\TOPSPIN} mjp 59

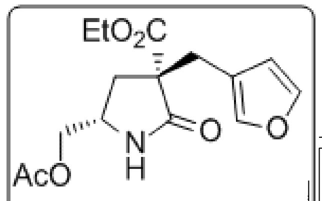


15b

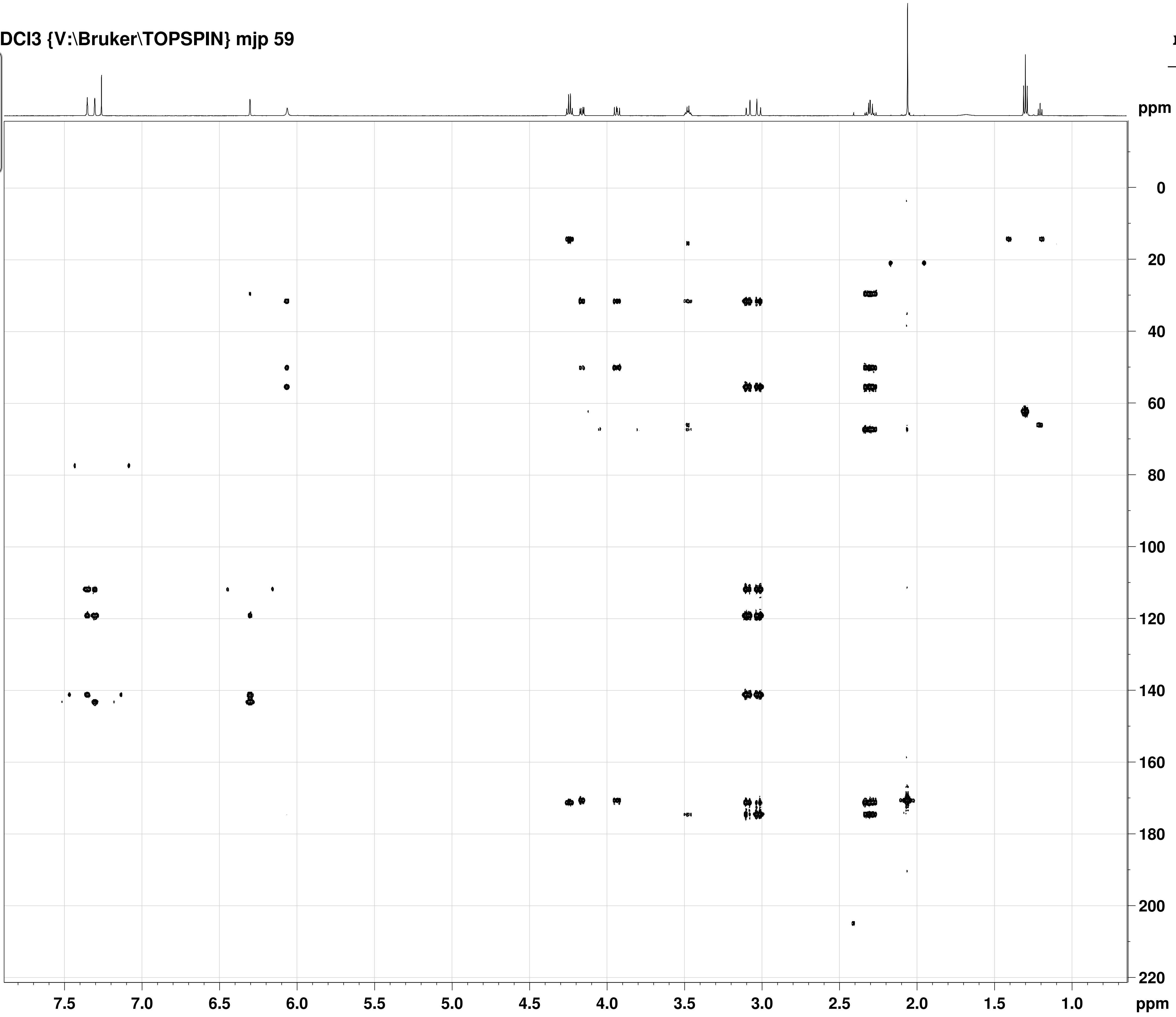


UCL

| | |
|---|---|
| NAME | JC-326-2 |
| EXPNO | 14 |
| PROCNO | 1 |
| Date_ | 20120130 |
| Time | 17.50 |
| INSTRUM | AV600 |
| PROBHD | 5 mm CPDCH 13C |
| PULPROG | hmbcetgpl3nd |
| TD | 4096 |
| SOLVENT | CDCl3 |
| NS | 2 |
| DS | 16 |
| SWH | 4347.826 Hz |
| FIDRES | 1.061481 Hz |
| AQ | 0.4710900 sec |
| RG | 2050 |
| DW | 115.000 usec |
| DE | 6.50 usec |
| TE | 298.0 K |
| CNST6 | 120.0000000 |
| CNST7 | 160.0000000 |
| CNST13 | 10.0000000 |
| CNST30 | 0.5981151 |
| D0 | 0.00000300 sec |
| D1 | 0.70675462 sec |
| D6 | 0.05000000 sec |
| D16 | 0.00020000 sec |
| IN0 | 0.00001380 sec |
| ======== CHANNEL f1 ======== | |
| NUC1 | 1H |
| P1 | 11.40 usec |
| P2 | 22.80 usec |
| PL1 | 1.00 dB |
| PL1W | 13.76731014 W |
| SFO1 | 600.1325723 MHz |
| ======== CHANNEL f2 ======== | |
| NUC2 | 13C |
| P3 | 9.80 usec |
| P24 | 2000.00 usec |
| PL2 | 5.00 dB |
| PL2W | 26.76886177 W |
| SFO2 | 150.9178993 MHz |
| SP7 | 13.33 dB |
| SPNAM7 | Crp60comp.4 |
| SPOAL7 | 0.500 |
| SPOFFS7 | 0.00 Hz |
| ====== GRADIENT CHANNEL ===== | |
| GPNAM1 | SINE.100 |
| GPNAM3 | SINE.100 |
| GPNAM4 | SINE.100 |
| GPNAM5 | SINE.100 |
| GPNAM6 | SINE.100 |
| GPZ1 | 80.00 % |
| GPZ3 | 14.00 % |
| GPZ4 | -8.00 % |
| GPZ5 | -4.00 % |
| GPZ6 | -2.00 % |
| P16 | 1000.00 usec |
| ND0 | 2 |
| TD | 256 |
| SFO1 | 150.9179 MHz |
| FIDRES | 141.485535 Hz |
| SW | 240.000 ppm |
| FnMODE | Echo-Antiecho |
| SI | 2048 |
| SF | 600.1300107 MHz |
| WDW | SINE |
| SSB | 2 |
| LB | 0.00 Hz |
| GB | 0 |
| PC | 1.40 |
| SI | 1024 |
| MC2 | echo-antiecho |
| SF | 150.9027756 MHz |
| WDW | SINE |
| SSB | 2 |
| LB | 0.00 Hz |
| GB | 0 |

**JC–327–2**
**PROTON.ucl CDCl3 {V:\Bruker\TOPSPIN} mjp 18**

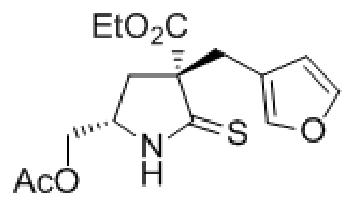


**16**

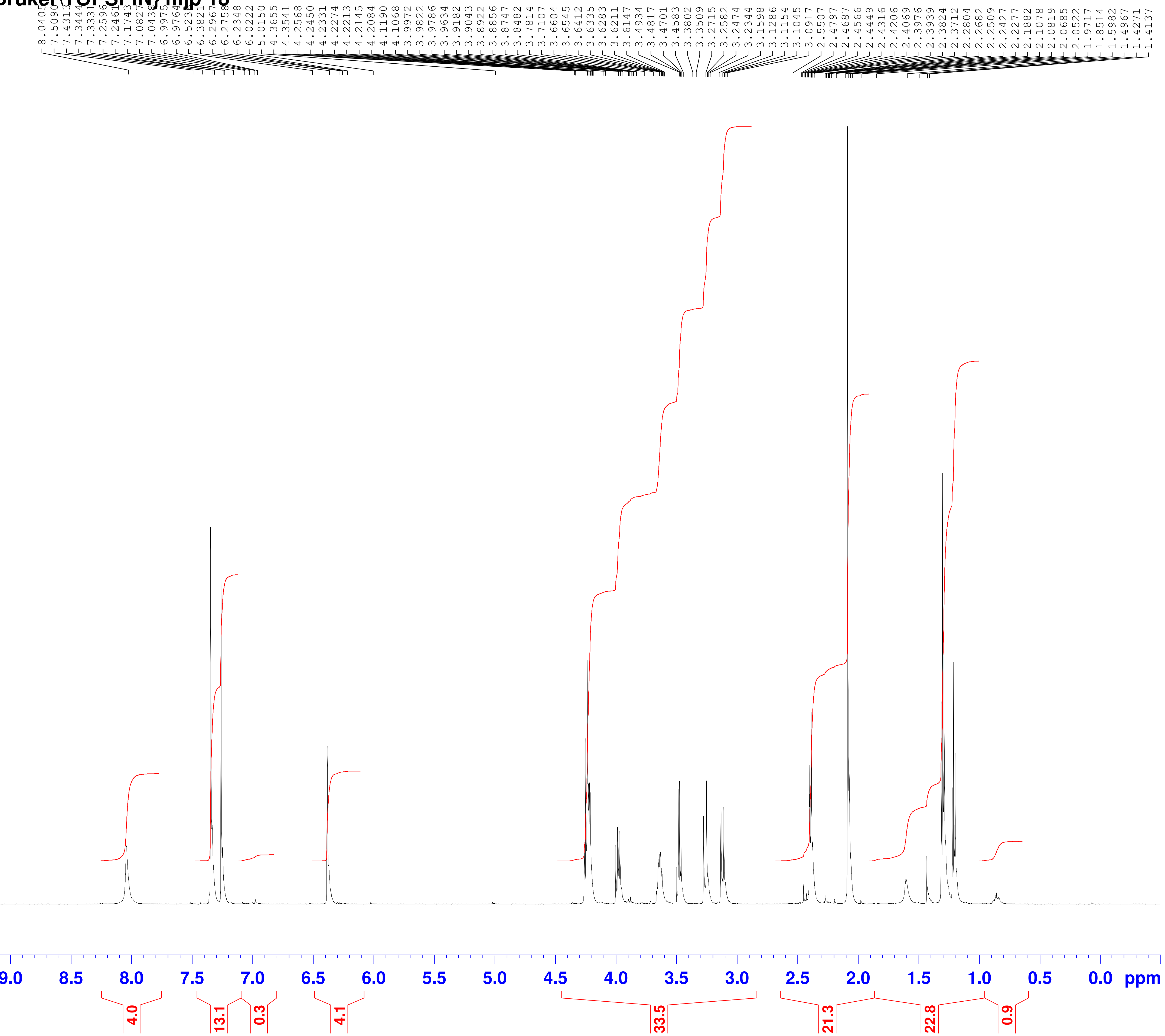


| | |
|---|---|
| NAME | JC-327-2 |
| EXPNO | 10 |
| PROCNO | 1 |
| Date_ | 20120204 |
| Time | 14.37 |
| INSTRUM | AV600 |
| PROBHD | 5 mm CPDCH 13C |
| PULPROG | zg30 |
| TD | 98682 |
| SOLVENT | CDCl3 |
| NS | 8 |
| DS | 0 |
| SWH | 12335.526 Hz |
| FIDRES | 0.125003 Hz |
| AQ | 3.9999604 sec |
| RG | 32 |
| DW | 40.533 use |
| DE | 10.48 use |
| TE | 298.0 K |
| D1 | 1.00000000 sec |
| TD0 | 1 |

======== CHANNEL f1 ========

| | |
|---|---|
| NUC1 | 1H |
| P1 | 11.40 use |
| PL1 | 1.00 dB |
| PL1W | 13.76731014 W |
| SFO1 | 600.1337061 MHz |
| SI | 32768 |
| SF | 600.1300116 MHz |
| WDW | EM |
| SSB | 0 |
| LB | 0.30 Hz |
| GB | 0 |
| PC | 1.40 |

# JC–327–2
# C13CPD.ucl CDCl3 {V:\Bruker\TOPSPIN} mjp 18

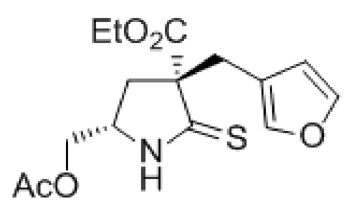

**16**

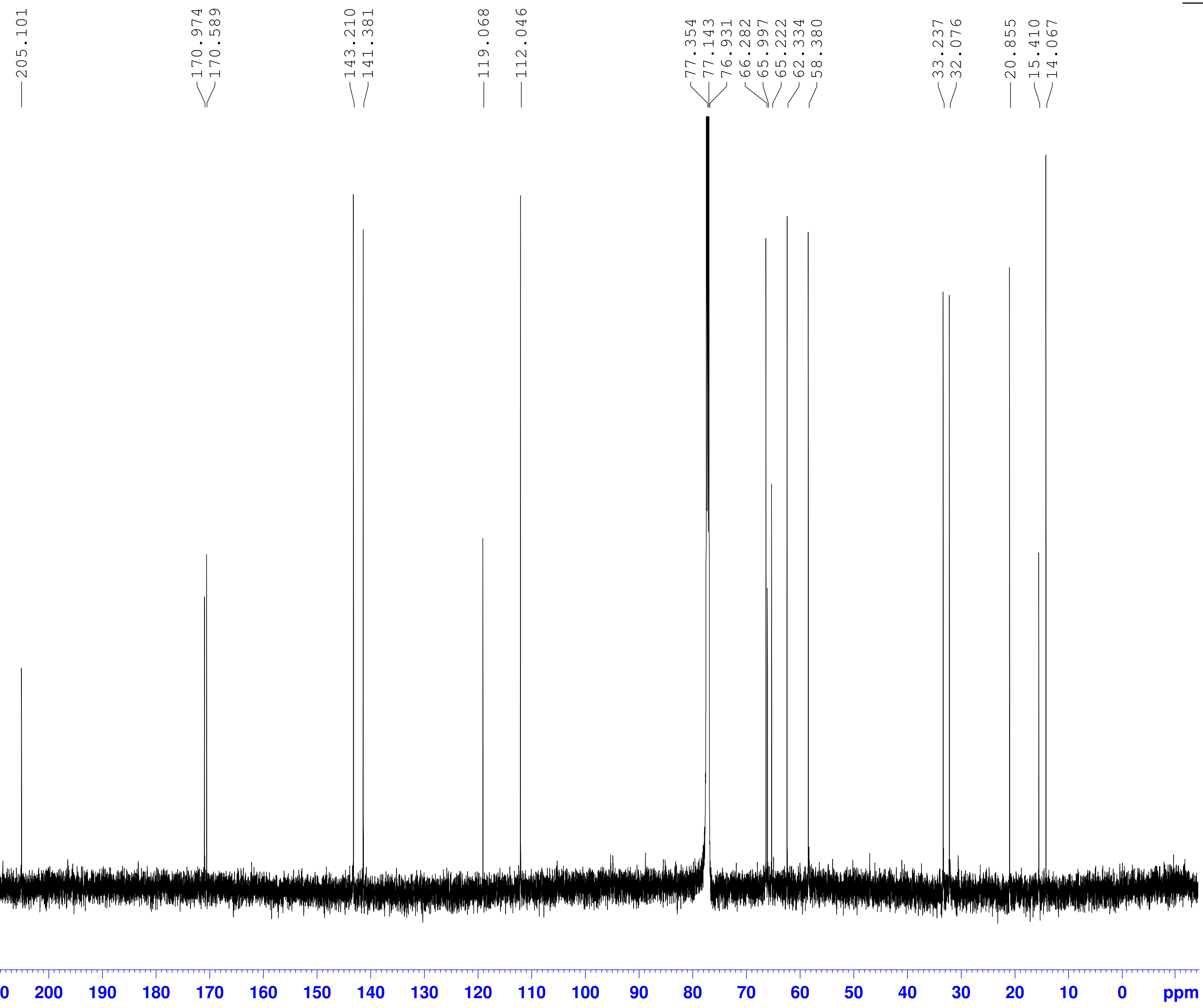


| | |
|---|---|
| NAME | JC-327-2 |
| EXPNO | 12 |
| PROCNO | 1 |
| Date_ | 20120204 |
| Time | 14.49 |
| INSTRUM | AV600 |
| PROBHD | 5 mm CPDCH 13C |
| PULPROG | zgpg30 |
| TD | 70308 |
| SOLVENT | CDCl3 |
| NS | 128 |
| DS | 0 |
| SWH | 39062.500 Hz |
| FIDRES | 0.555591 Hz |
| AQ | 0.8999924 sec |
| RG | 1030 |
| DW | 12.800 usec |
| DE | 21.12 usec |
| TE | 298.0 K |
| D1 | 2.00000000 sec |
| D11 | 0.03000000 sec |
| TD0 | 1 |

======== CHANNEL f1 ========

| | |
|---|---|
| NUC1 | 13C |
| P1 | 9.80 usec |
| PL1 | 5.00 dB |
| PL1W | 26.76886177 W |
| SFO1 | 150.9201628 MHz |

======== CHANNEL f2 ========

| | |
|---|---|
| CPDPRG2 | waltz16 |
| NUC2 | 1H |
| PCPD2 | 70.00 usec |
| PL2 | 1.00 dB |
| PL12 | 17.23 dB |
| PL13 | 20.00 dB |
| PL2W | 13.76731014 W |
| PL12W | 0.32798135 W |
| PL13W | 0.17332016 W |
| SFO2 | 600.1324005 MHz |
| SI | 65536 |
| SF | 150.9027930 MHz |
| WDW | EM |
| SSB | 0 |
| LB | 1.00 Hz |
| GB | 0 |
| PC | 1.40 |

JC–327–2
COSY.ucl CDCl3 {V:\Bruker\TOPSPIN} mjp 18

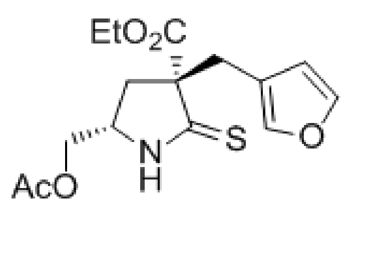


16

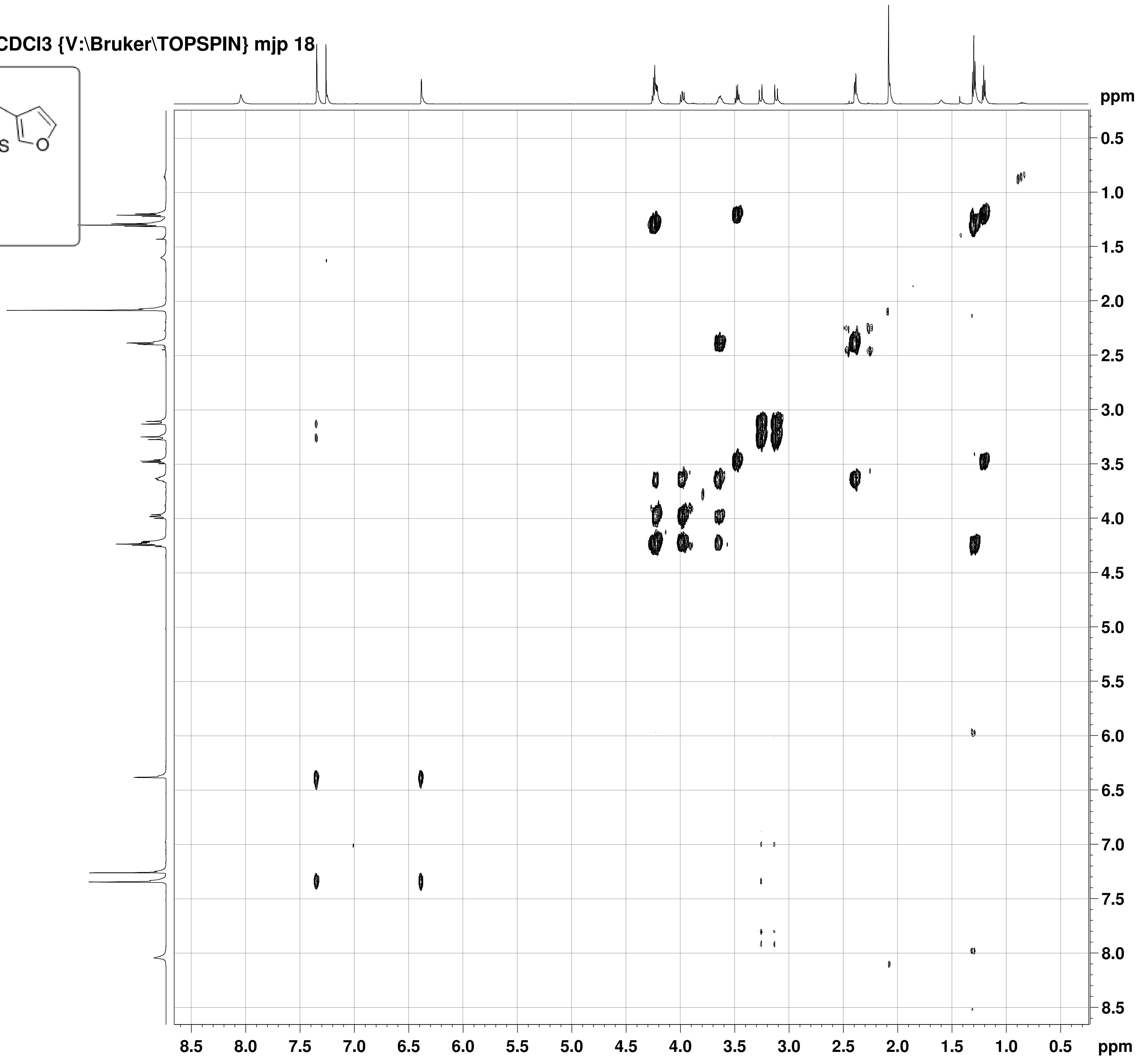


| | |
|---|---|
| NAME | JC-327-2 |
| EXPNO | 11 |
| PROCNO | 1 |
| Date_ | 20120204 |
| Time | 14.38 |
| INSTRUM | AV600 |
| PROBHD | 5 mm CPDCH 13C |
| PULPROG | cosygpmfqf |
| TD | 2048 |
| SOLVENT | CDCl3 |
| NS | 1 |
| DS | 8 |
| SWH | 5050.505 Hz |
| FIDRES | 2.466067 Hz |
| AQ | 0.2028020 sec |
| RG | 2050 |
| DW | 99.000 usec |
| DE | 6.50 usec |
| TE | 298.0 K |
| D0 | 0.00000300 sec |
| D1 | 1.69730496 sec |
| D13 | 0.00000400 sec |
| D16 | 0.00020000 sec |
| IN0 | 0.00019800 sec |
| ======== CHANNEL f1 ======== | |
| NUC1 | 1H |
| P1 | 11.40 usec |
| PL1 | 1.00 dB |
| PL1W | 13.76731014 W |
| SFO1 | 600.1326815 MHz |
| ====== GRADIENT CHANNEL ==== | |
| GPNAM1 | SINE.100 |
| GPNAM2 | SINE.100 |
| GPNAM3 | SINE.100 |
| GPZ1 | 16.00 % |
| GPZ2 | 12.00 % |
| GPZ3 | 40.00 % |
| P16 | 1000.00 usec |
| ND0 | 1 |
| TD | 128 |
| SFO1 | 600.1327 MHz |
| FIDRES | 39.457069 Hz |
| SW | 8.416 ppm |
| FnMODE | QF |
| SI | 1024 |
| SF | 600.1300093 MHz |
| WDW | QSINE |
| SSB | 0 |
| LB | 0.00 Hz |
| GB | 0 |
| PC | 1.40 |
| SI | 1024 |
| MC2 | QF |
| SF | 600.1300093 MHz |
| WDW | QSINE |
| SSB | 0 |
| LB | 0.00 Hz |
| GB | 0 |

**JC–327–2**
**C13DEPT135.ucl CDCl3 {V:\Bruker\TOPSPIN} mjp 18**

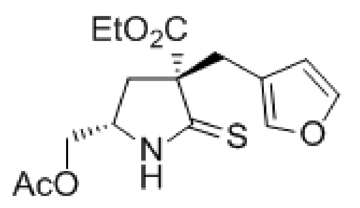


**16**

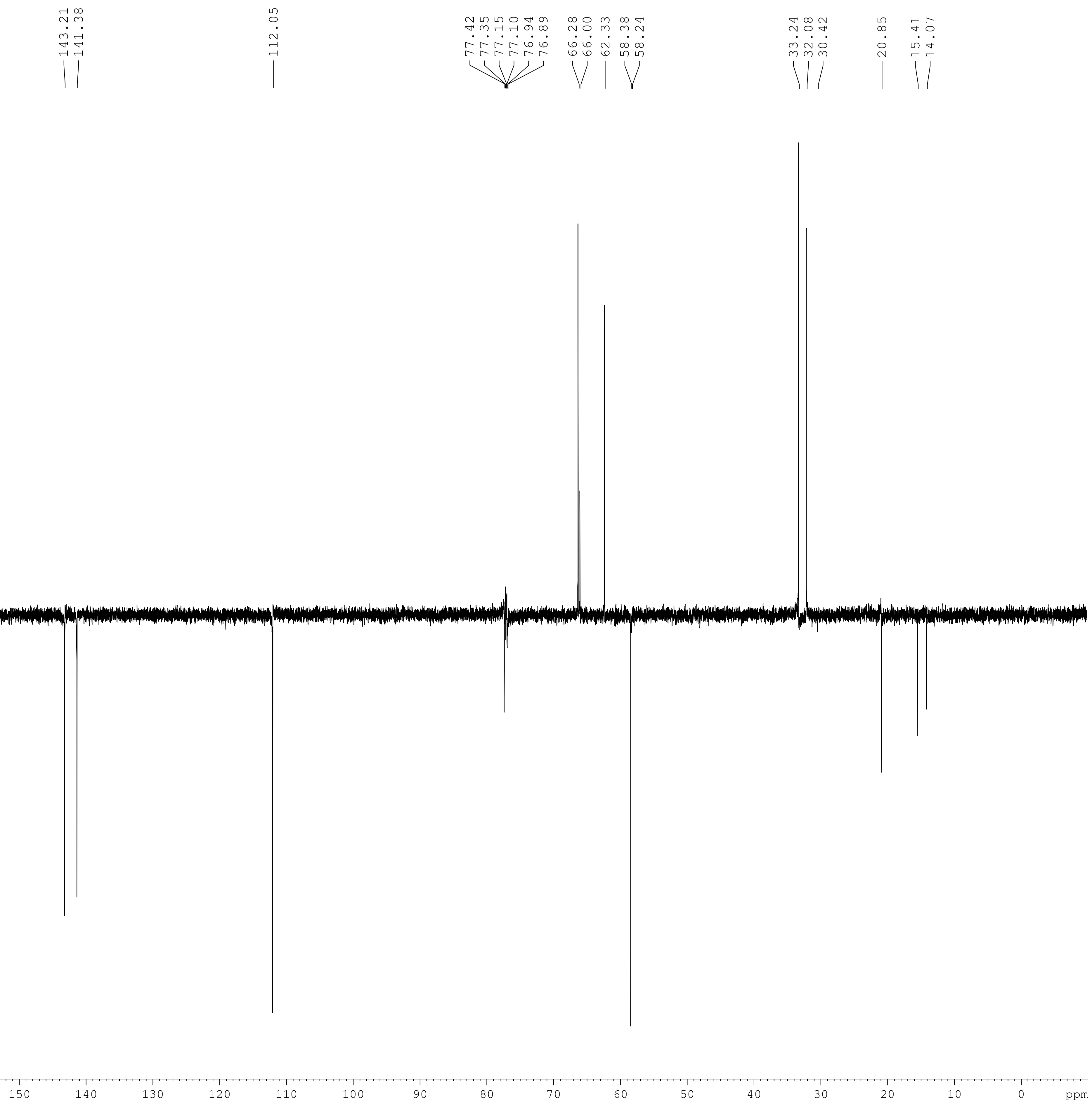


```
NAME             JC-327-2
EXPNO                  15
PROCNO                  1
Date_            20120204
Time                15.12
INSTRUM             AV600
PROBHD   5 mm CPDCH 13C
PULPROG           dept135
TD                  70308
SOLVENT             CDCl3
NS                     64
DS                      4
SWH             39062.500 H
FIDRES           0.555591 H
AQ              0.8999924 s
RG                    256
DW                 12.800 u
DE                   6.50 u
TE                  298.0 K
CNST2         145.0000000
D1             2.00000000 s
D2             0.00344828 s
D12            0.00002000 s
TD0                     1

======== CHANNEL f1 =====
NUC1                  13C
P1                   9.80 u
P2                  19.60 u
PL1                  5.00 d
PL1W          26.76886177 W
SFO1          150.9201628 M

======== CHANNEL f2 =====
CPDPRG2           waltz16
NUC2                   1H
P3                  10.80 u
P4                  21.60 u
PCPD2               70.00 u
PL2                  1.00 d
PL12                17.23 d
PL2W          13.76731014 W
PL12W          0.32798135 W
SFO2          600.1324005 M
SI                  65536
SF            150.9027930 M
WDW                    EM
SSB                     0
LB                   1.00 H
GB                      0
PC                   1.40
```

JC–327–2
HSQC.ucl CDCl3 {V:\Bruker\TOPSPIN} mjp 18

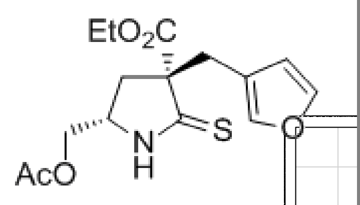

**16**

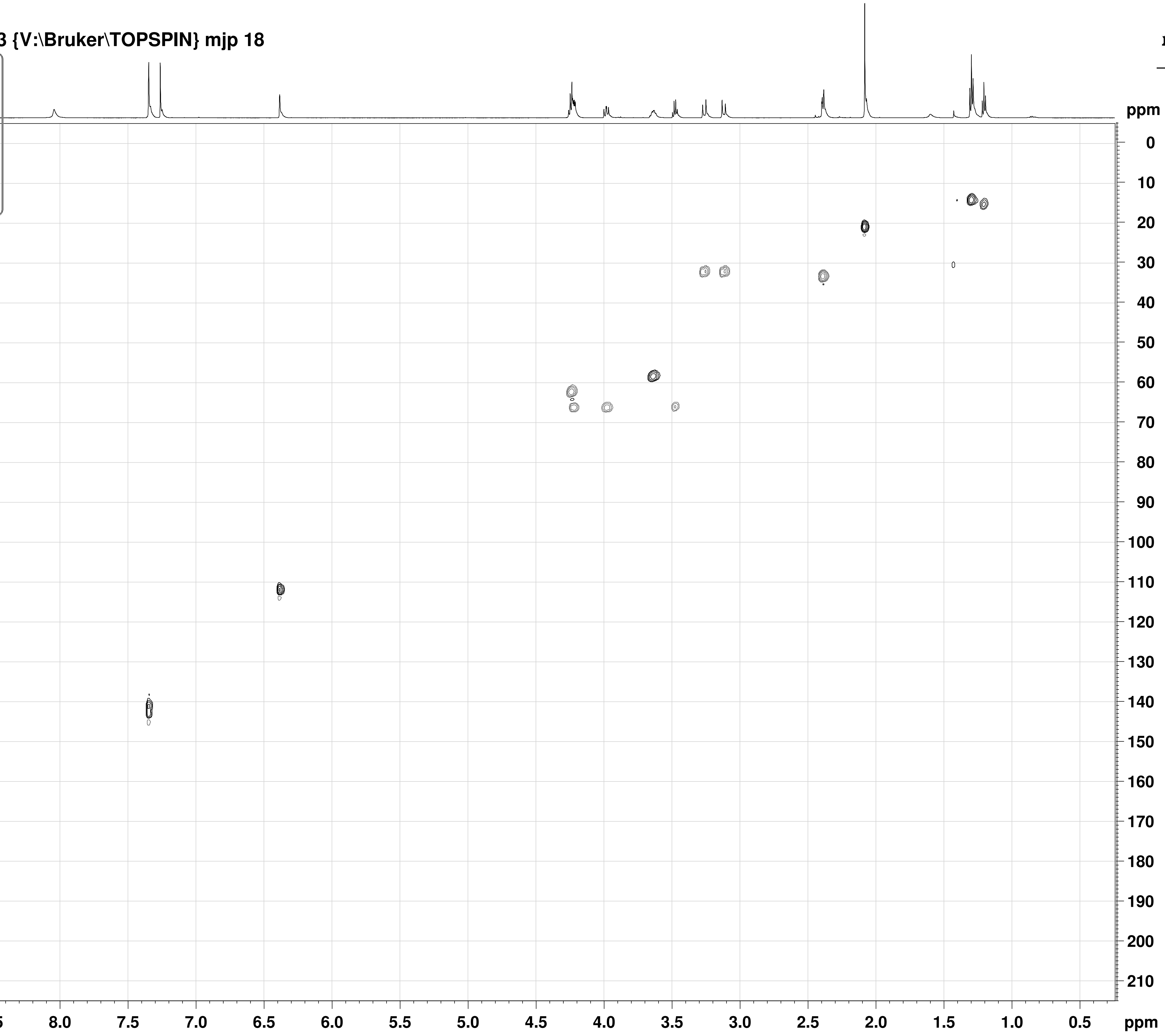


```
NAME          JC-327-2
EXPNO               13
PROCNO               1
Date_         20120204
Time             14.50
INSTRUM          AV600
PROBHD   5 mm CPDCH 13C
PULPROG  hsqcedetgpsisp2.4
TD                1024
SOLVENT          CDCl3
NS                   2
DS                  32
SWH           5050.505 Hz
FIDRES        4.932134 Hz
AQ           0.1014260 sec
RG                2050
DW              99.000 usec
DE               6.50 usec
TE              298.0 K
CNST2      145.0000000
CNST17      -0.5000000
D0          0.00000300 sec
D1          1.48382103 sec
D2          0.00344828 sec
D4          0.00172414 sec
D11         0.03000000 sec
D16         0.00020000 sec
D21         0.00344828 sec
D24         0.00086207 sec
IN0         0.00001505 sec
L0                   0
L31                  1
TD0                  2

======== CHANNEL f1 ========
NUC1                1H
P1               11.40 usec
P2               22.80 usec
P28               0.00 usec
PL1               1.00 dB
PL1W       13.76731014 W
SFO1       600.1326815 MHz

======== CHANNEL f2 ========
CPDPRG2  bi_p5m4sp_4sp.2
NUC2               13C
P3                9.80 usec
P14             500.00 usec
P24            2000.00 usec
P31            1730.00 usec
P63            1500.00 usec
PL0             120.00 dB
PL2               5.00 dB
PL12             20.74 dB
PL0W        0.00000000 W
PL2W       26.76886177 W
PL12W       0.71388775 W
SFO2       150.9186538 MHz
SP3              13.33 dB
SP7              13.33 dB
SP14             14.82 dB
SP18             18.73 dB
SP31             20.84 dB
SPNAM3   Crp60,0.5,20.1
SPNAM7     Crp60comp.4
SPNAM14  Crp32,1.5,20.2
SPNAM18   Crp60_xfilt.2
SPNAM31  Crp32,1.5,20.2
SPOAL3           0.500
SPOAL7           0.500
SPOAL14          0.500
SPOAL18          0.500
SPOAL31          0.500
SPOFFS3           0.00 Hz
SPOFFS7           0.00 Hz
SPOFFS14          0.00 Hz
SPOFFS18          0.00 Hz
SPOFFS31          0.00 Hz

====== GRADIENT CHANNEL =====
GPNAM1        SINE.100
GPNAM2        SINE.100
GPNAM3        SINE.100
GPNAM4        SINE.100
GPZ1             80.00 %
GPZ2             20.10 %
GPZ3             11.00 %
GPZ4             -5.00 %
P16            1000.00 usec
P19             600.00 usec
ND0                  2
TD                 128
SFO1          150.9187 MHz
FIDRES      259.391449 Hz
SW             220.000 ppm
FnMODE    Echo-Antiecho
SI                1024
SF         600.1300093 MHz
WDW              QSINE
SSB                  2
LB                0.00 Hz
GB                   0
PC                1.40
SI                1024
MC2       echo-antiecho
SF         150.9027771 MHz
WDW              QSINE
SSB                  2
LB                0.00 Hz
GB                   0
```

**JC–327–2**
**HMBC.ucl CDCl3 {V:\Bruker\TOPSPIN} mjp 18**

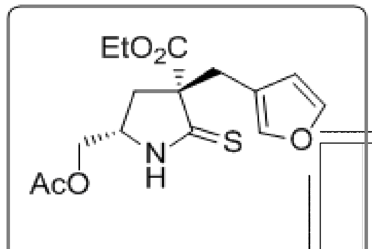


16

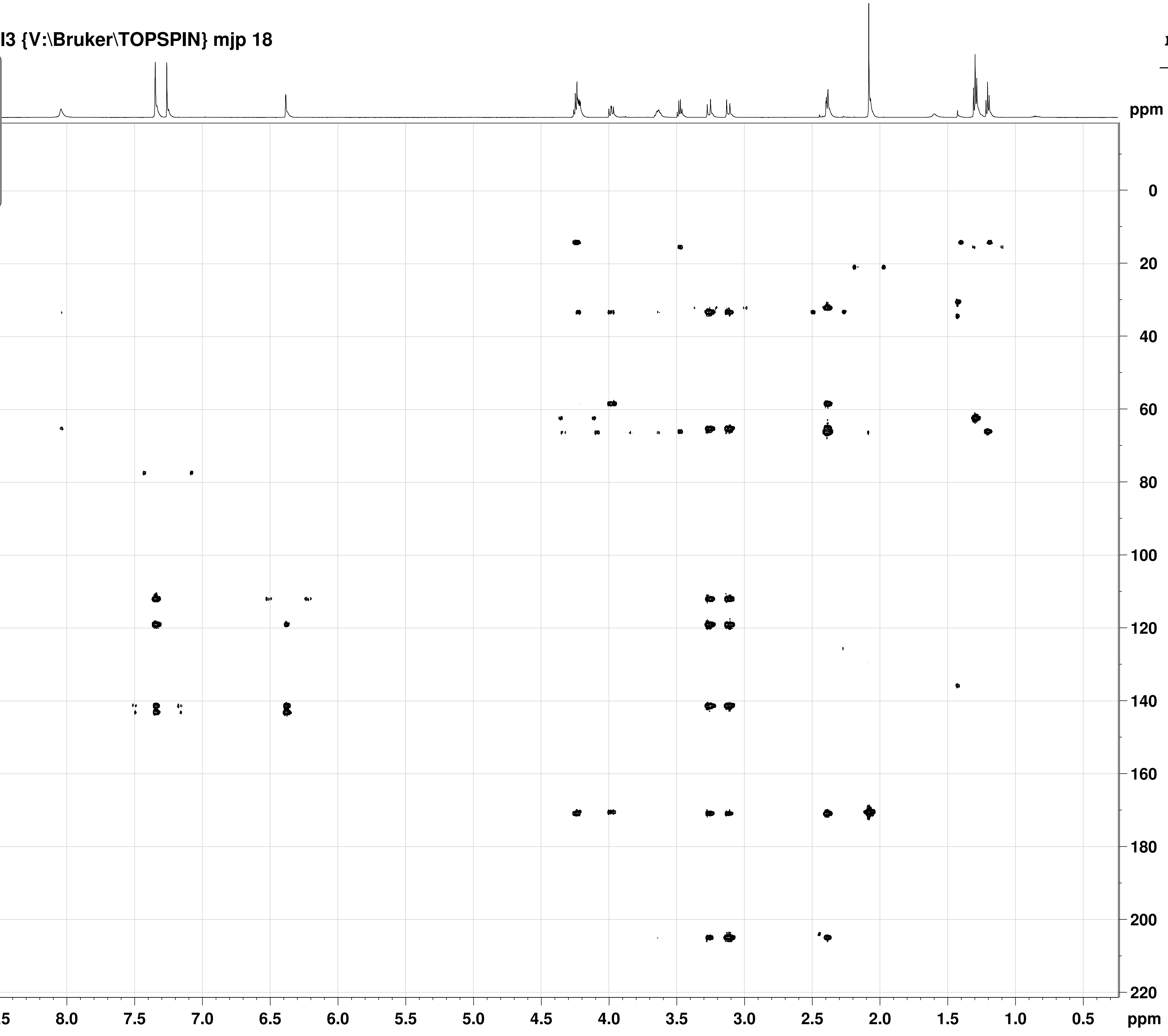


```
NAME              JC-327-2
EXPNO                   14
PROCNO                   1
Date_             20120204
Time                 14.58
INSTRUM              AV600
PROBHD   5 mm CPDCH 13C
PULPROG     hmbcetgpl3nd
TD                    4096
SOLVENT              CDCl3
NS                       2
DS                      16
SWH               5050.505 Hz
FIDRES            1.233033 Hz
AQ               0.4055540 sec
RG                    2050
DW                  99.000 usec
DE                    6.50 usec
TE                   298.0 K
CNST6          120.0000000
CNST7          160.0000000
CNST13          10.0000000
CNST30           0.5981152
D0              0.00000300 sec
D1              0.77229059 sec
D6              0.05000000 sec
D16             0.00020000 sec
IN0             0.00001380 sec

======== CHANNEL f1 ========
NUC1                    1H
P1                   11.40 usec
P2                   22.80 usec
PL1                   1.00 dB
PL1W           13.76731014 W
SFO1           600.1326815 MHz

======== CHANNEL f2 ========
NUC2                   13C
P3                    9.80 usec
P24                2000.00 usec
PL2                   5.00 dB
PL2W           26.76886177 W
SFO2           150.9178993 MHz
SP7                  13.33 dB
SPNAM7          Crp60comp.4
SPOAL7               0.500
SPOFFS7               0.00 Hz

====== GRADIENT CHANNEL =====
GPNAM1            SINE.100
GPNAM3            SINE.100
GPNAM4            SINE.100
GPNAM5            SINE.100
GPNAM6            SINE.100
GPZ1                 80.00 %
GPZ3                 14.00 %
GPZ4                 -8.00 %
GPZ5                 -4.00 %
GPZ6                 -2.00 %
P16                1000.00 usec
ND0                      2
TD                     256
SFO1              150.9179 MHz
FIDRES          141.485535 Hz
SW                 240.000 ppm
FnMODE        Echo-Antiecho
SI                    2048
SF             600.1300106 MHz
WDW                   SINE
SSB                      2
LB                    0.00 Hz
GB                       0
PC                    1.40
SI                    1024
MC2           echo-antiecho
SF             150.9027756 MHz
WDW                   SINE
SSB                      2
LB                    0.00 Hz
GB                       0
```

JC/2792
PROTON.ucl CDCl3 {C:\Bruker\TOPSPIN} mjp 31

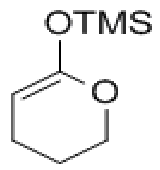


**17a**

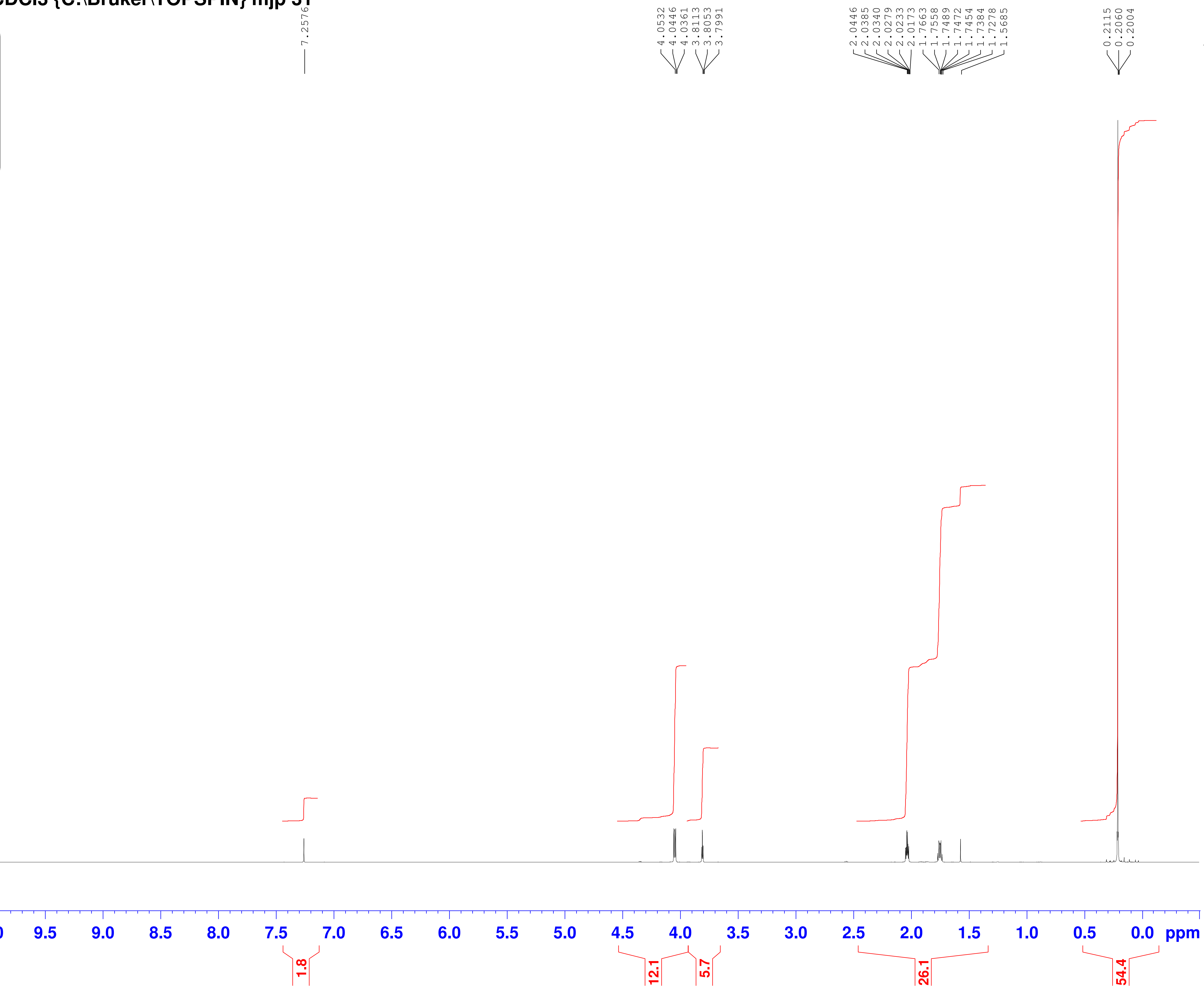


| | |
|---|---|
| NAME | Mar16-2010 |
| EXPNO | 20 |
| PROCNO | 1 |
| Date_ | 20100316 |
| Time | 15.18 |
| INSTRUM | AV600 |
| PROBHD | 5 mm CPDCH 13C |
| PULPROG | zg30 |
| TD | 65536 |
| SOLVENT | CDCl3 |
| NS | 8 |
| DS | 0 |
| SWH | 12335.526 Hz |
| FIDRES | 0.188225 Hz |
| AQ | 2.6564426 sec |
| RG | 18 |
| DW | 40.533 use |
| DE | 10.48 use |
| TE | 298.0 K |
| D1 | 1.00000000 sec |
| TD0 | 1 |

======== CHANNEL f1 =======

| | |
|---|---|
| NUC1 | 1H |
| P1 | 11.40 use |
| PL1 | 1.00 dB |
| PL1W | 13.76731014 W |
| SFO1 | 600.1337061 MHz |
| SI | 32768 |
| SF | 600.1300116 MHz |
| WDW | EM |
| SSB | 0 |
| LB | 0.30 Hz |
| GB | 0 |
| PC | 1.40 |

**JC/2792**
**C13CPD.ucl CDCl3 {C:\Bruker\TOPSPIN} mjp 31**

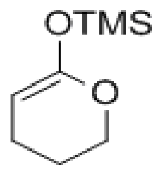


17a

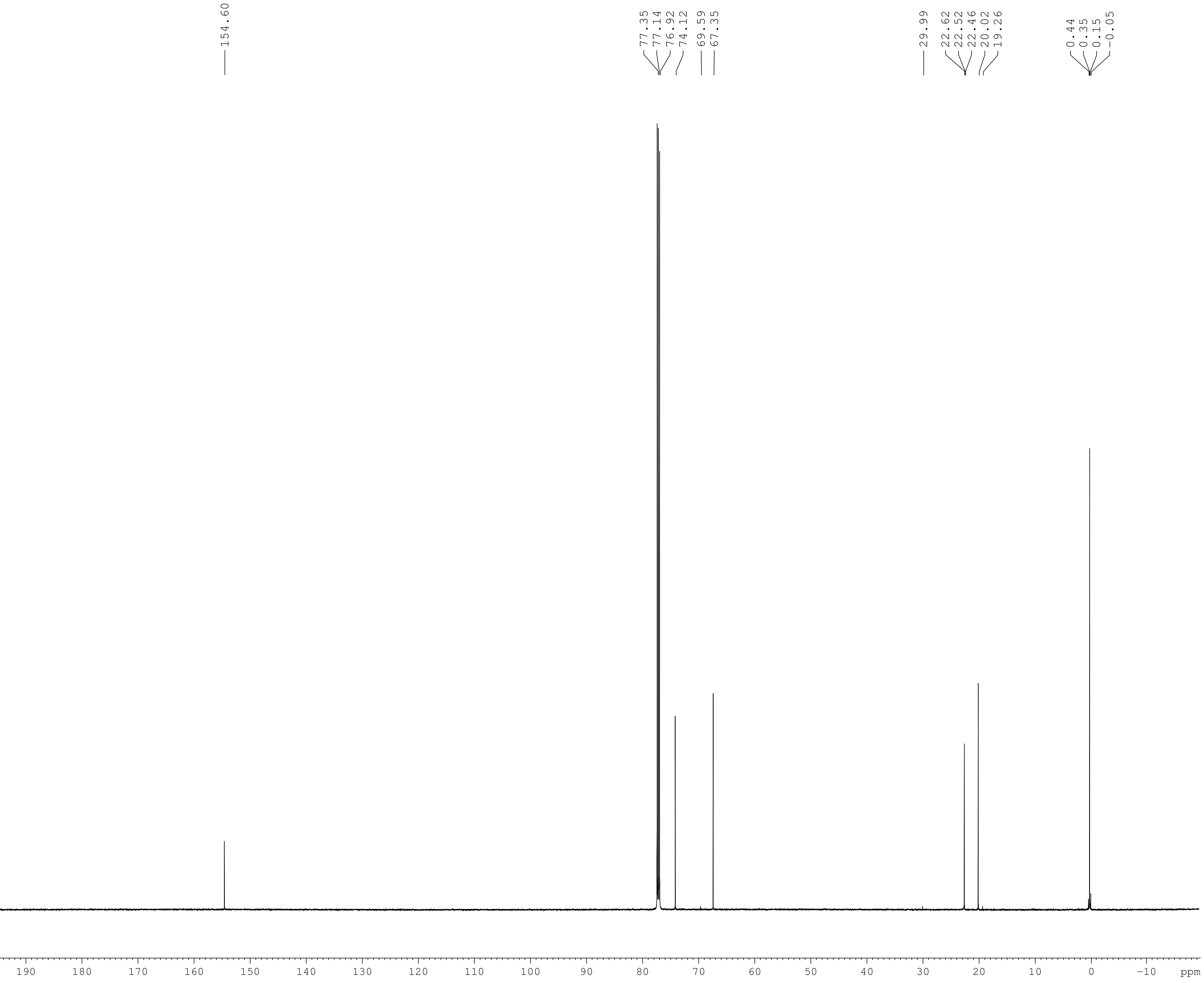


UCL

| | |
|---|---|
| NAME | Mar16-2010 |
| EXPNO | 21 |
| PROCNO | 1 |
| Date_ | 20100316 |
| Time | 15.31 |
| INSTRUM | AV600 |
| PROBHD | 5 mm CPDCH 13C |
| PULPROG | zgpg30 |
| TD | 65536 |
| SOLVENT | CDCl3 |
| NS | 128 |
| DS | 0 |
| SWH | 36057.691 H |
| FIDRES | 0.550197 H |
| AQ | 0.9088159 s |
| RG | 1030 |
| DW | 13.867 u |
| DE | 21.55 u |
| TE | 298.0 K |
| D1 | 2.00000000 s |
| D11 | 0.03000000 s |
| TD0 | 1 |
| ======== CHANNEL f1 ===== | |
| NUC1 | 13C |
| P1 | 9.80 u |
| PL1 | 5.00 d |
| PL1W | 26.76886177 W |
| SFO1 | 150.9178981 M |
| ======== CHANNEL f2 ===== | |
| CPDPRG2 | waltz16 |
| NUC2 | 1H |
| PCPD2 | 70.00 u |
| PL2 | 1.00 d |
| PL12 | 16.76 d |
| PL13 | 120.00 d |
| PL2W | 13.76731014 W |
| PL12W | 0.36546776 W |
| PL13W | 0.00000000 W |
| SFO2 | 600.1324005 M |
| SI | 32768 |
| SF | 150.9027930 M |
| WDW | EM |
| SSB | 0 |
| LB | 1.00 H |
| GB | 0 |
| PC | 1.40 |

**JC–358–2**
**PROTON.ucl CDCl3 {V:\Bruker\TOPSPIN} mjp 13**

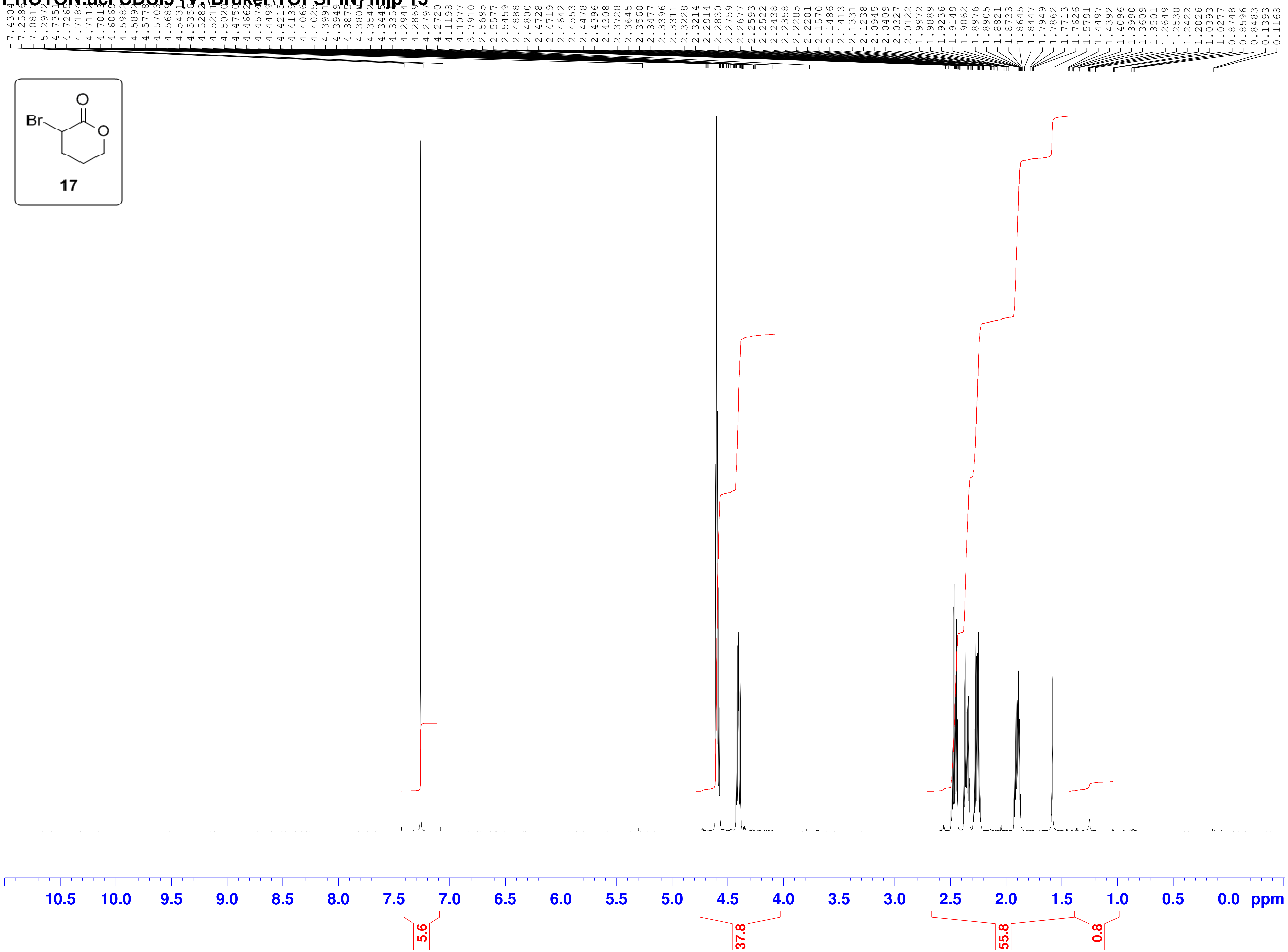


| | |
|---|---|
| NAME | JC-358-2 |
| EXPNO | 10 |
| PROCNO | 1 |
| Date_ | 20120323 |
| Time | 18.13 |
| INSTRUM | AV600 |
| PROBHD | 5 mm CPDCH 13C |
| PULPROG | zg30 |
| TD | 98682 |
| SOLVENT | CDCl3 |
| NS | 8 |
| DS | 0 |
| SWH | 12335.526 Hz |
| FIDRES | 0.125003 Hz |
| AQ | 3.9999604 sec |
| RG | 40.3 |
| DW | 40.533 use |
| DE | 10.48 use |
| TE | 298.0 K |
| D1 | 1.00000000 sec |
| TD0 | 1 |

======== CHANNEL f1 ========

| | |
|---|---|
| NUC1 | 1H |
| P1 | 11.40 use |
| PL1 | 1.00 dB |
| PL1W | 13.76731014 W |
| SFO1 | 600.1337061 MHz |
| SI | 32768 |
| SF | 600.1300116 MHz |
| WDW | EM |
| SSB | 0 |
| LB | 0.30 Hz |
| GB | 0 |
| PC | 1.40 |

V:BrukerTOPSPIN/mjp/JC–358–2/10/1
Integral regions #1 to #2

JC–358–2
PROTON.ucl CDCl3 {V:\Bruker\TOPSPIN} mjp 13

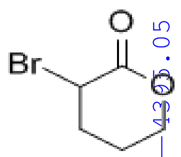


17

30 Hz/cm

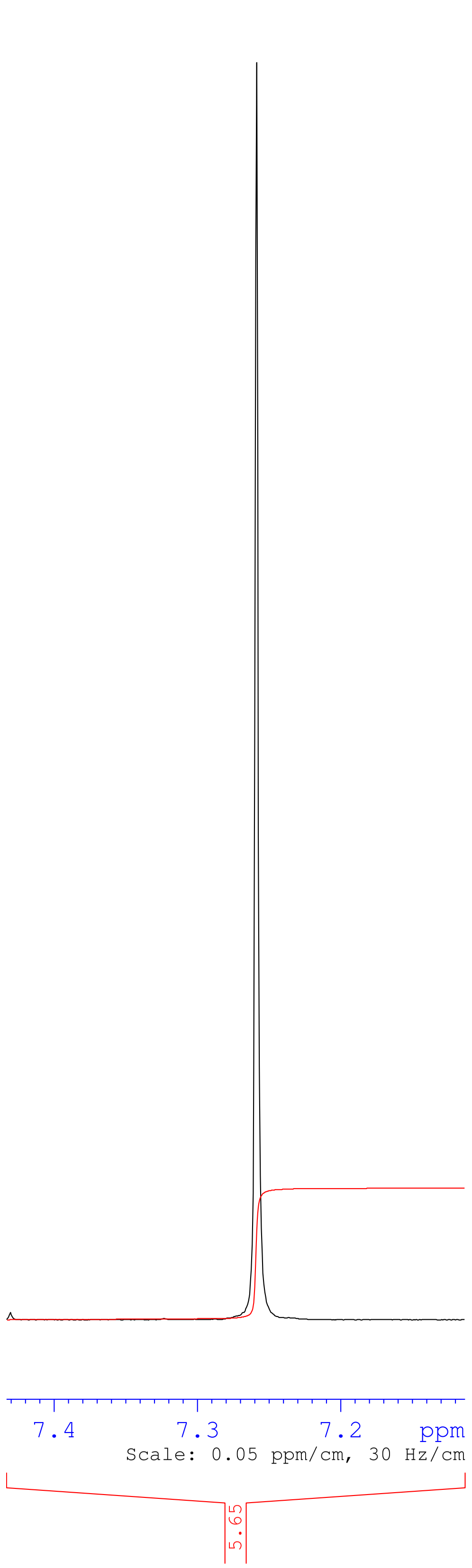


30 Hz/cm

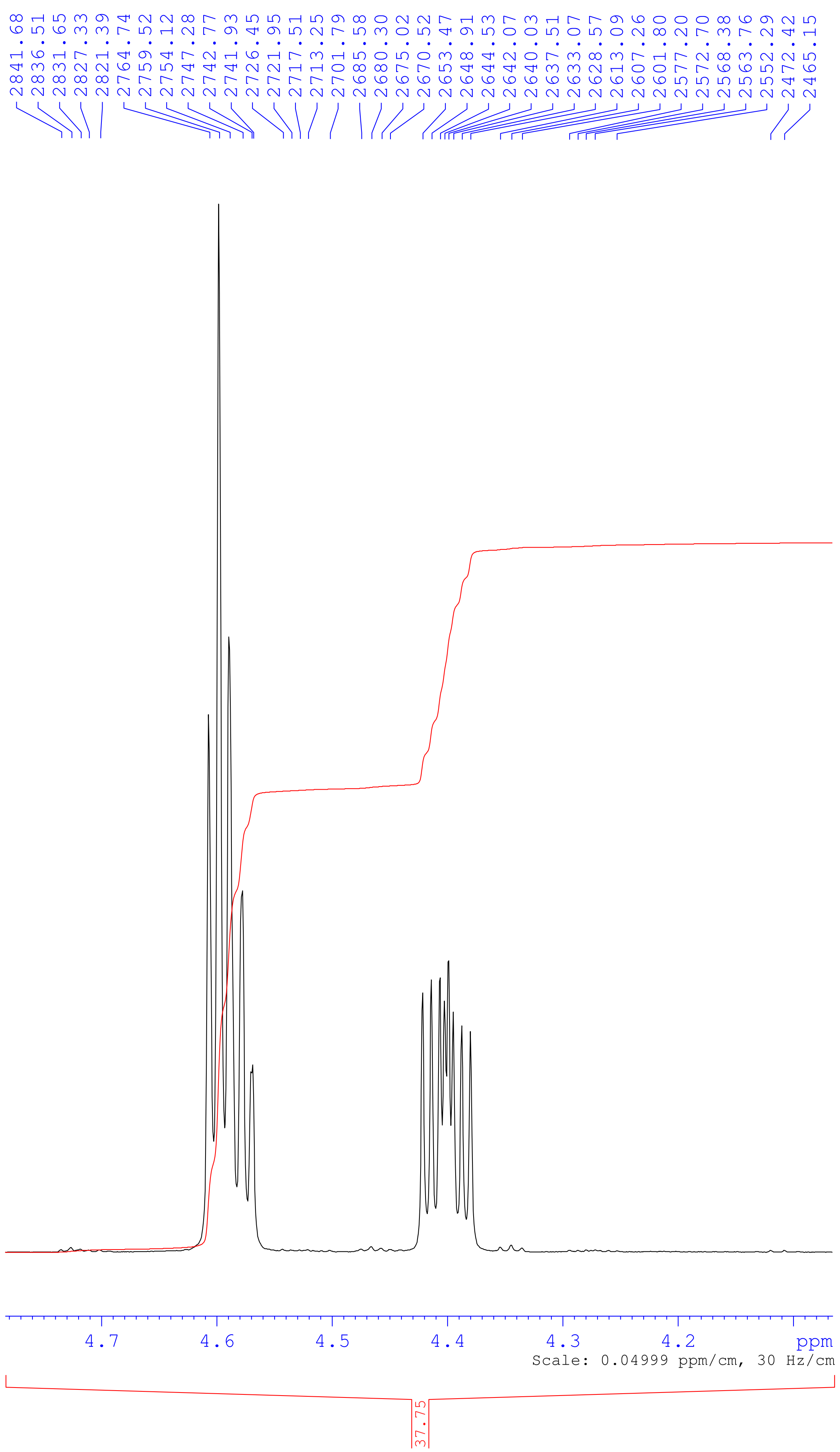

V:BrukerTOPSPIN/mjp/JC−358−2/10/1
Integral regions #3 to #3

30 Hz/cm

JC−358−2
PROTON.ucl CDCl3 {V:\Bruker\TOPSPIN} mjp 13

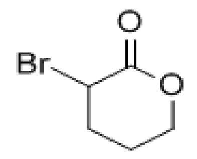


**17**

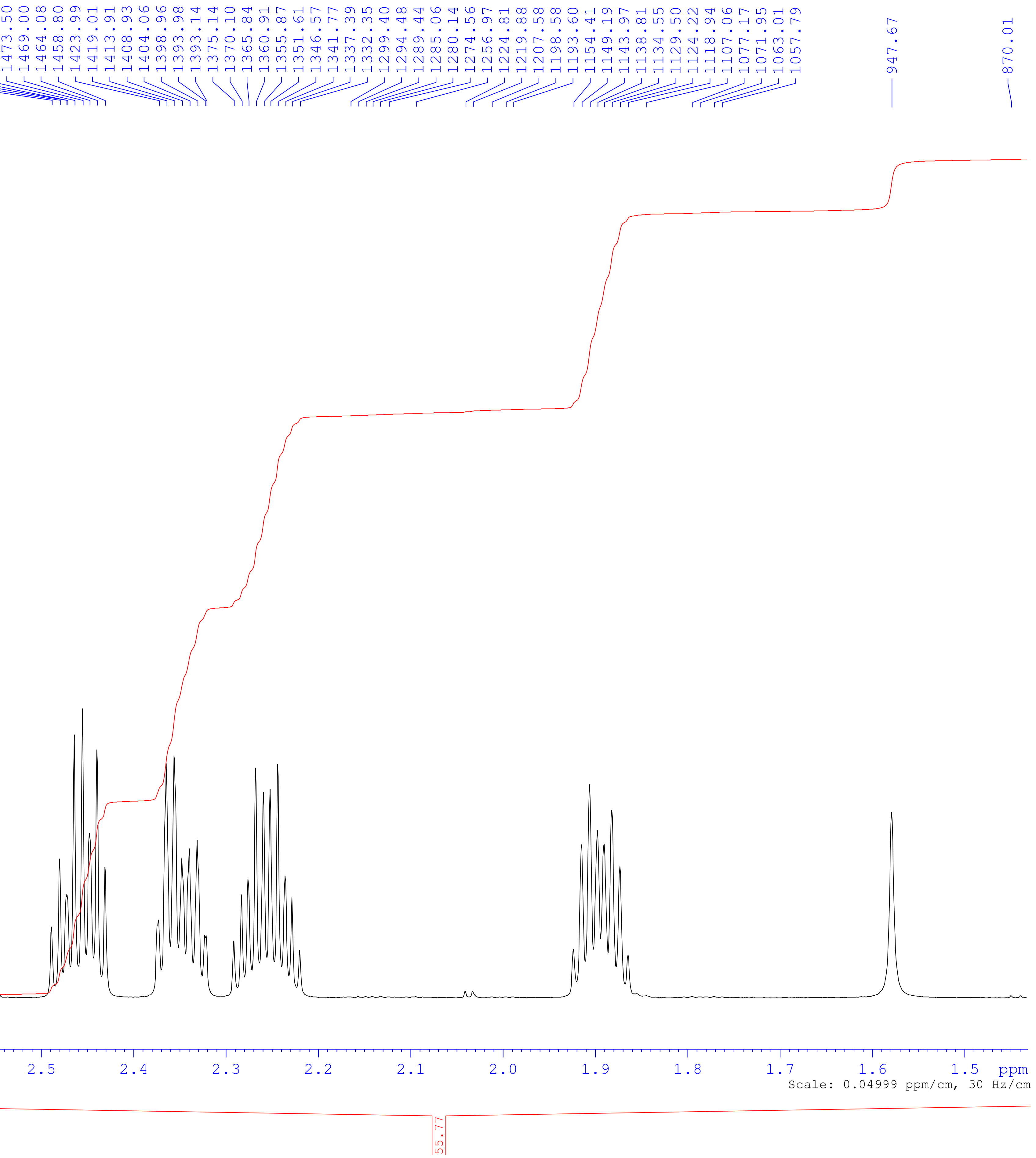

**JC−358−2**
**C13CPD.ucl CDCl3 {V:\Bruker\TOPSPIN} mjp 13**

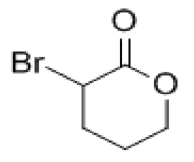


**17**

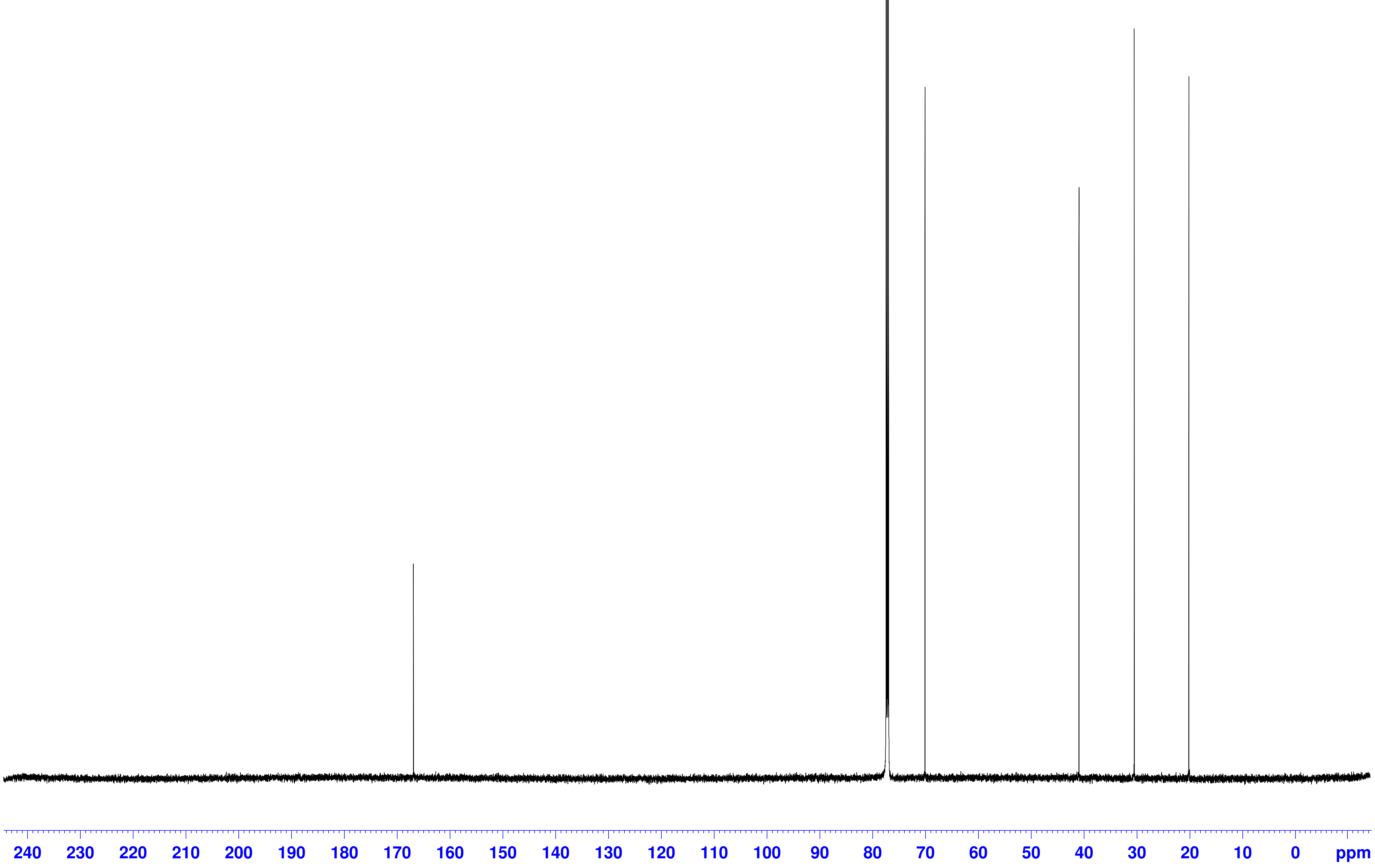


UCL

| | |
|---|---|
| NAME | JC-358-2 |
| EXPNO | 12 |
| PROCNO | 1 |
| Date_ | 20120323 |
| Time | 18.25 |
| INSTRUM | AV600 |
| PROBHD | 5 mm CPDCH 13C |
| PULPROG | zgpg30 |
| TD | 70308 |
| SOLVENT | CDCl3 |
| NS | 128 |
| DS | 0 |
| SWH | 39062.500 H |
| FIDRES | 0.555591 H |
| AQ | 0.8999924 s |
| RG | 1030 |
| DW | 12.800 u |
| DE | 21.12 u |
| TE | 298.0 K |
| D1 | 2.00000000 s |
| D11 | 0.03000000 s |
| TD0 | 1 |
| ======== CHANNEL f1 ===== | |
| NUC1 | 13C |
| P1 | 9.80 u |
| PL1 | 5.00 d |
| PL1W | 26.76886177 W |
| SFO1 | 150.9201628 M |
| ======== CHANNEL f2 ===== | |
| CPDPRG2 | waltz16 |
| NUC2 | 1H |
| PCPD2 | 70.00 u |
| PL2 | 1.00 d |
| PL12 | 17.23 d |
| PL13 | 20.00 d |
| PL2W | 13.76731014 W |
| PL12W | 0.32798135 W |
| PL13W | 0.17332016 W |
| SFO2 | 600.1324005 M |
| SI | 65536 |
| SF | 150.9027930 M |
| WDW | EM |
| SSB | 0 |
| LB | 1.00 H |
| GB | 0 |
| PC | 1.40 |

JC–358–2
COSY.ucl CDCl3 {V:\Bruker\TOPSPIN} mjp 13

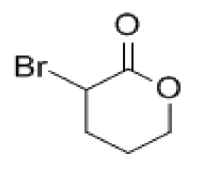


17

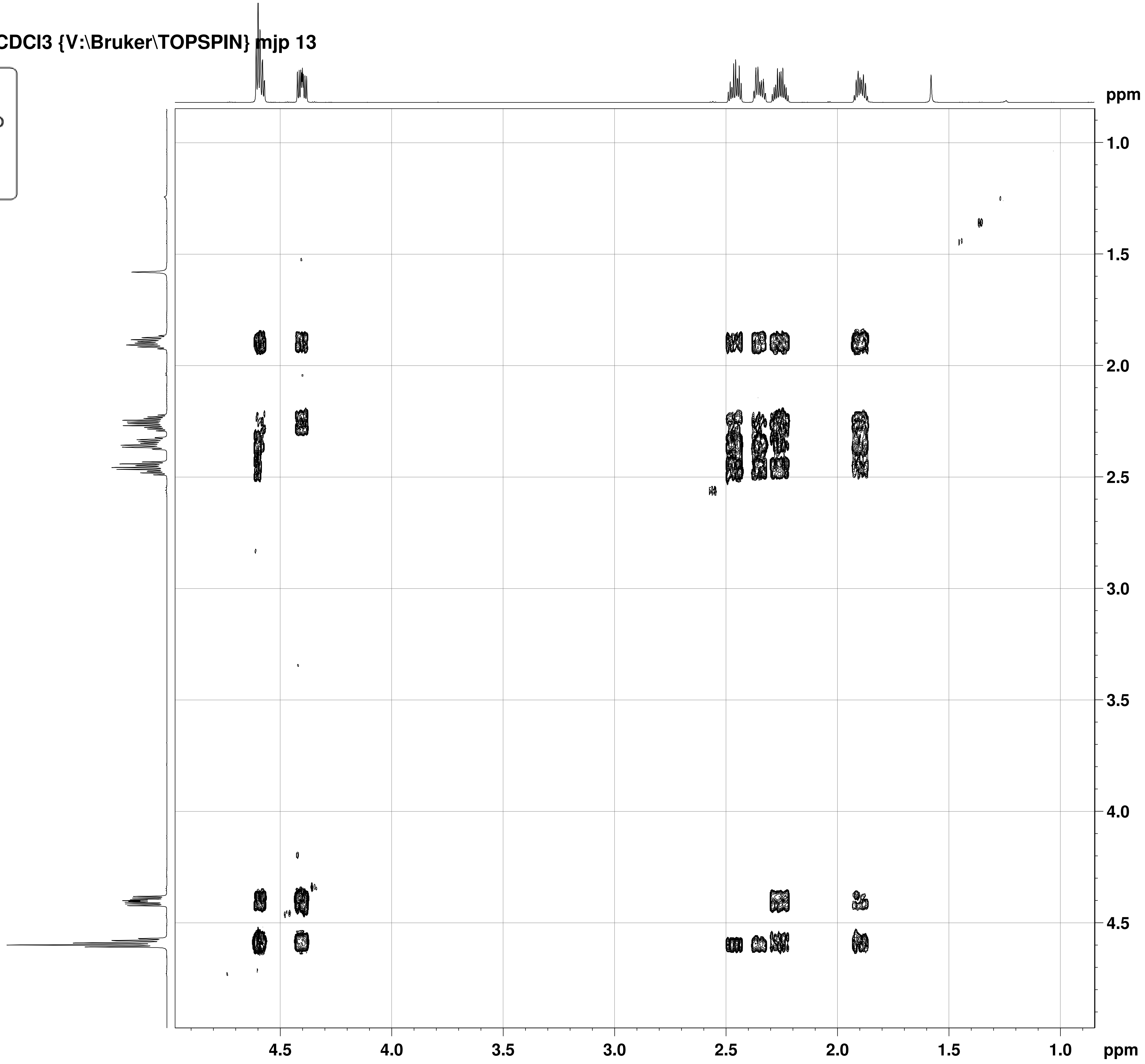


UCL

```
NAME            JC-358-2
EXPNO                 11
PROCNO                 1
Date_           20120323
Time               18.14
INSTRUM            AV600
PROBHD   5 mm CPDCH 13C
PULPROG       cosygpmfqf
TD                  2048
SOLVENT            CDCl3
NS                     1
DS                     8
SWH             2475.248 Hz
FIDRES          1.208617 Hz
AQ             0.4137460 sec
RG                  2050
DW               202.000 usec
DE                  6.50 usec
TE                 298.0 K
D0            0.00000300 sec
D1            1.48636103 sec
D13           0.00000400 sec
D16           0.00020000 sec
IN0           0.00040400 sec

======== CHANNEL f1 ========
NUC1                  1H
P1                 11.40 usec
PL1                 1.00 dB
PL1W         13.76731014 W
SFO1         600.1317575 MHz

====== GRADIENT CHANNEL ====
GPNAM1          SINE.100
GPNAM2          SINE.100
GPNAM3          SINE.100
GPZ1               16.00 %
GPZ2               12.00 %
GPZ3               40.00 %
P16              1000.00 usec
ND0                    1
TD                   128
SFO1            600.1318 MHz
FIDRES         19.337872 Hz
SW                 4.125 ppm
FnMODE                QF
SI                  1024
SF           600.1300106 MHz
WDW                QSINE
SSB                    0
LB                  0.00 Hz
GB                     0
PC                  1.40
SI                  1024
MC2                   QF
SF           600.1300106 MHz
WDW                QSINE
SSB                    0
LB                  0.00 Hz
GB                     0
```

**JC–358–2**
**HSQC.ucl CDCl3 {V:\Bruker\TOPSPIN} mjp 13**

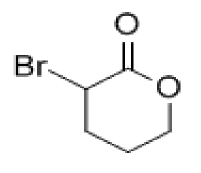

17

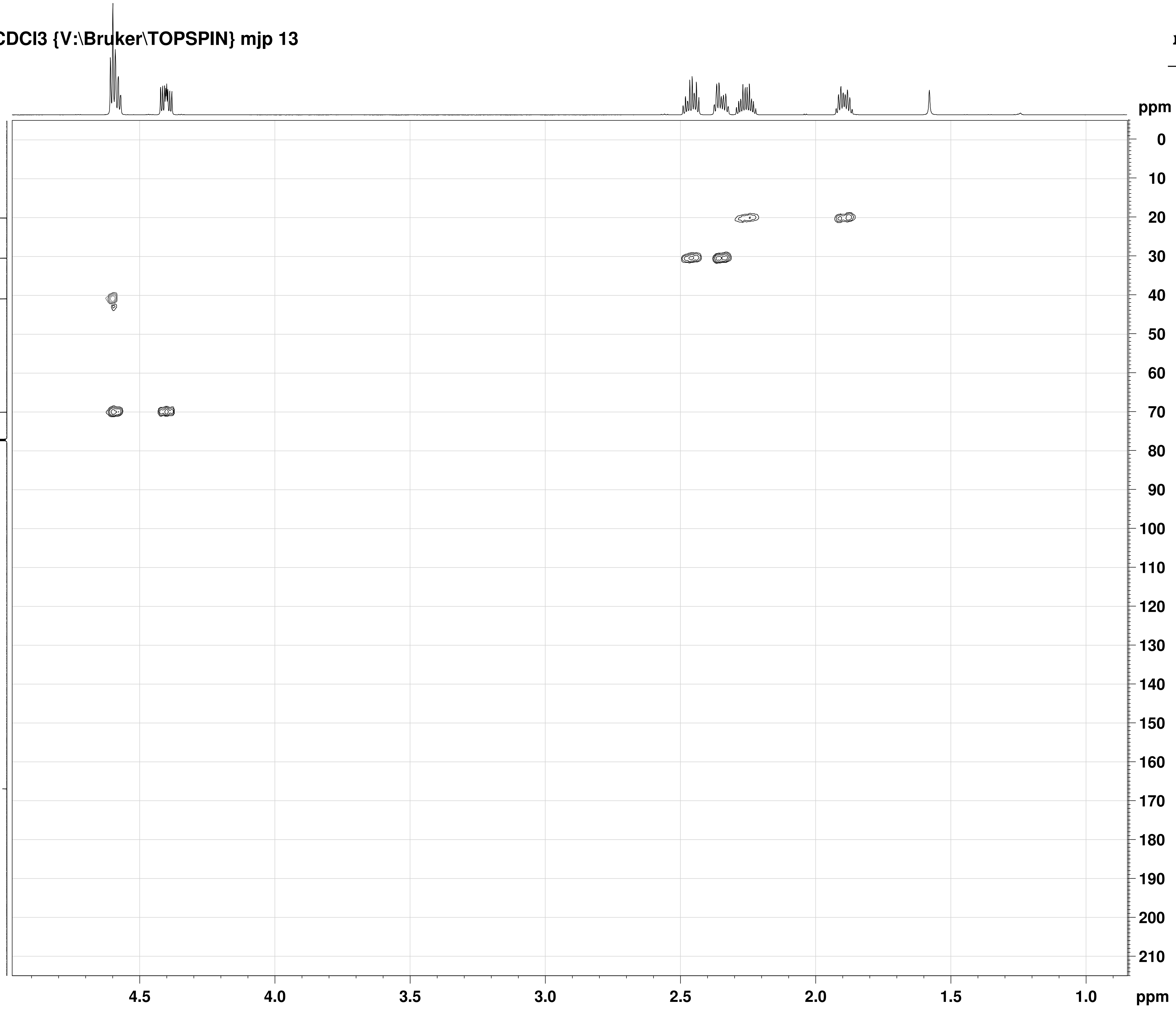


UCL

```
NAME          JC-358-2
EXPNO               13
PROCNO               1
Date_         20120323
Time             18.26
INSTRUM          AV600
PROBHD   5 mm CPDCH 13C
PULPROG  hsqcedetgpsisp2.4
TD                1024
SOLVENT          CDCl3
NS                   2
DS                  32
SWH           2475.248 Hz
FIDRES        2.417234 Hz
AQ           0.2068980 sec
RG                2050
DW             202.000 usec
DE                6.50 usec
TE               298.0 K
CNST2      145.0000000
CNST17      -0.5000000
D0          0.00000300 sec
D1          1.37834895 sec
D2          0.00344828 sec
D4          0.00172414 sec
D11         0.03000000 sec
D16         0.00020000 sec
D21         0.00344828 sec
D24         0.00086207 sec
IN0         0.00001505 sec
L0                   0
L31                  1
TD0                  2

======== CHANNEL f1 ========
NUC1                1H
P1               11.40 usec
P2               22.80 usec
P28               0.00 usec
PL1               1.00 dB
PL1W       13.76731014 W
SFO1       600.1317575 MHz

======== CHANNEL f2 ========
CPDPRG2  bi_p5m4sp_4sp.2
NUC2               13C
P3                9.80 usec
P14             500.00 usec
P24            2000.00 usec
P31            1730.00 usec
P63            1500.00 usec
PL0             120.00 dB
PL2               5.00 dB
PL12             20.74 dB
PL0W        0.00000000 W
PL2W       26.76886177 W
PL12W       0.71388775 W
SFO2       150.9186538 MHz
SP3              13.33 dB
SP7              13.33 dB
SP14             14.82 dB
SP18             18.73 dB
SP31             20.84 dB
SPNAM3   Crp60,0.5,20.1
SPNAM7     Crp60comp.4
SPNAM14  Crp32,1.5,20.2
SPNAM18   Crp60_xfilt.2
SPNAM31  Crp32,1.5,20.2
SPOAL3           0.500
SPOAL7           0.500
SPOAL14          0.500
SPOAL18          0.500
SPOAL31          0.500
SPOFFS3           0.00 Hz
SPOFFS7           0.00 Hz
SPOFFS14          0.00 Hz
SPOFFS18          0.00 Hz
SPOFFS31          0.00 Hz

====== GRADIENT CHANNEL =====
GPNAM1        SINE.100
GPNAM2        SINE.100
GPNAM3        SINE.100
GPNAM4        SINE.100
GPZ1             80.00 %
GPZ2             20.10 %
GPZ3             11.00 %
GPZ4             -5.00 %
P16            1000.00 usec
P19             600.00 usec
ND0                  2
TD                 128
SFO1          150.9187 MHz
FIDRES      259.391449 Hz
SW             220.000 ppm
FnMODE    Echo-Antiecho
SI                1024
SF         600.1300106 MHz
WDW              QSINE
SSB                  2
LB                0.00 Hz
GB                   0
PC                1.40
SI                1024
MC2       echo-antiecho
SF         150.9027771 MHz
WDW              QSINE
SSB                  2
LB                0.00 Hz
GB                   0
```

# JC–358–2
## HMBC.ucl CDCl3 {V:\Bruker\TOPSPIN} mjp 13

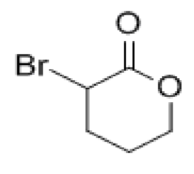

17

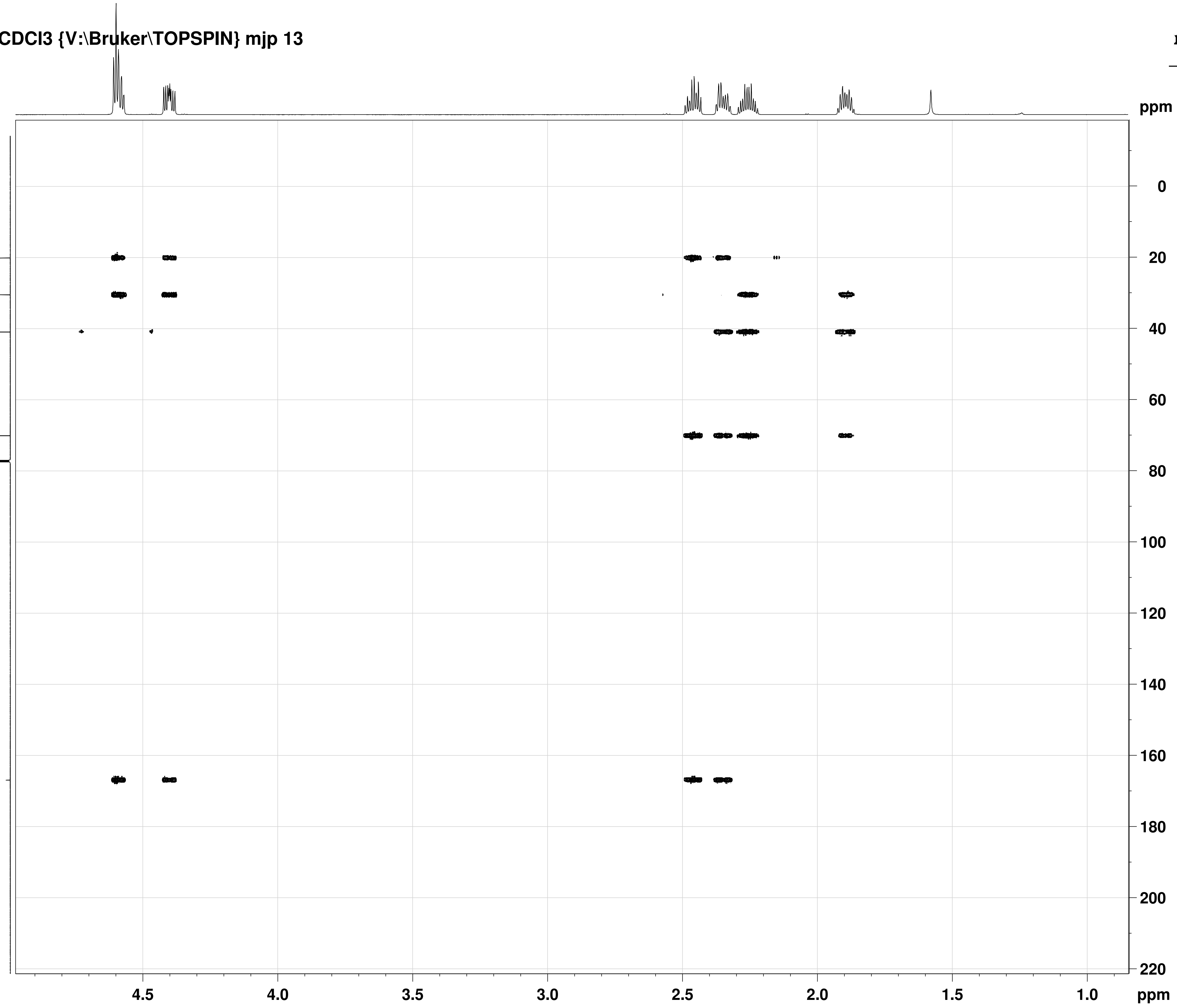


```
NAME              JC-358-2
EXPNO                   14
PROCNO                   1
Date_             20120323
Time                 22.43
INSTRUM              AV600
PROBHD    5 mm CPDCH 13C
PULPROG     hmbcetgpl3nd
TD                    4096
SOLVENT              CDCl3
NS                       2
DS                      16
SWH              2475.248 Hz
FIDRES           0.604308 Hz
AQ              0.8274420 sec
RG                    2050
DW                 202.000 usec
DE                    6.50 usec
TE                   298.0 K
CNST6          120.0000000
CNST7          160.0000000
CNST13          10.0000000
CNST30           0.5981147
D0              0.00000300 sec
D1              0.35040259 sec
D6              0.05000000 sec
D16             0.00020000 sec
IN0             0.00001380 sec

======== CHANNEL f1 ========
NUC1                    1H
P1                   11.40 usec
P2                   22.80 usec
PL1                   1.00 dB
PL1W           13.76731014 W
SFO1           600.1317575 MHz

======== CHANNEL f2 ========
NUC2                   13C
P3                    9.80 usec
P24                2000.00 usec
PL2                   5.00 dB
PL2W           26.76886177 W
SFO2           150.9178993 MHz
SP7                  13.33 dB
SPNAM7          Crp60comp.4
SPOAL7                0.500
SPOFFS7                0.00 Hz

====== GRADIENT CHANNEL =====
GPNAM1            SINE.100
GPNAM3            SINE.100
GPNAM4            SINE.100
GPNAM5            SINE.100
GPNAM6            SINE.100
GPZ1                 80.00 %
GPZ3                 14.00 %
GPZ4                 -8.00 %
GPZ5                 -4.00 %
GPZ6                 -2.00 %
P16                1000.00 usec
ND0                      2
TD                     256
SFO1              150.9179 MHz
FIDRES          141.485535 Hz
SW                 240.000 ppm
FnMODE        Echo-Antiecho
SI                    2048
SF             600.1300112 MHz
WDW                   SINE
SSB                      2
LB                    0.00 Hz
GB                       0
PC                    1.40
SI                    1024
MC2           echo-antiecho
SF             150.9027756 MHz
WDW                   SINE
SSB                      2
LB                    0.00 Hz
GB                       0
```

**JC–331–2**
**PROTON.ucl CDCl3 {V:\Bruker\TOPSPIN} mjp 56**

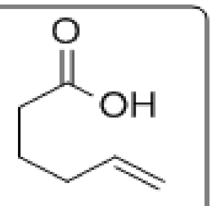

18a

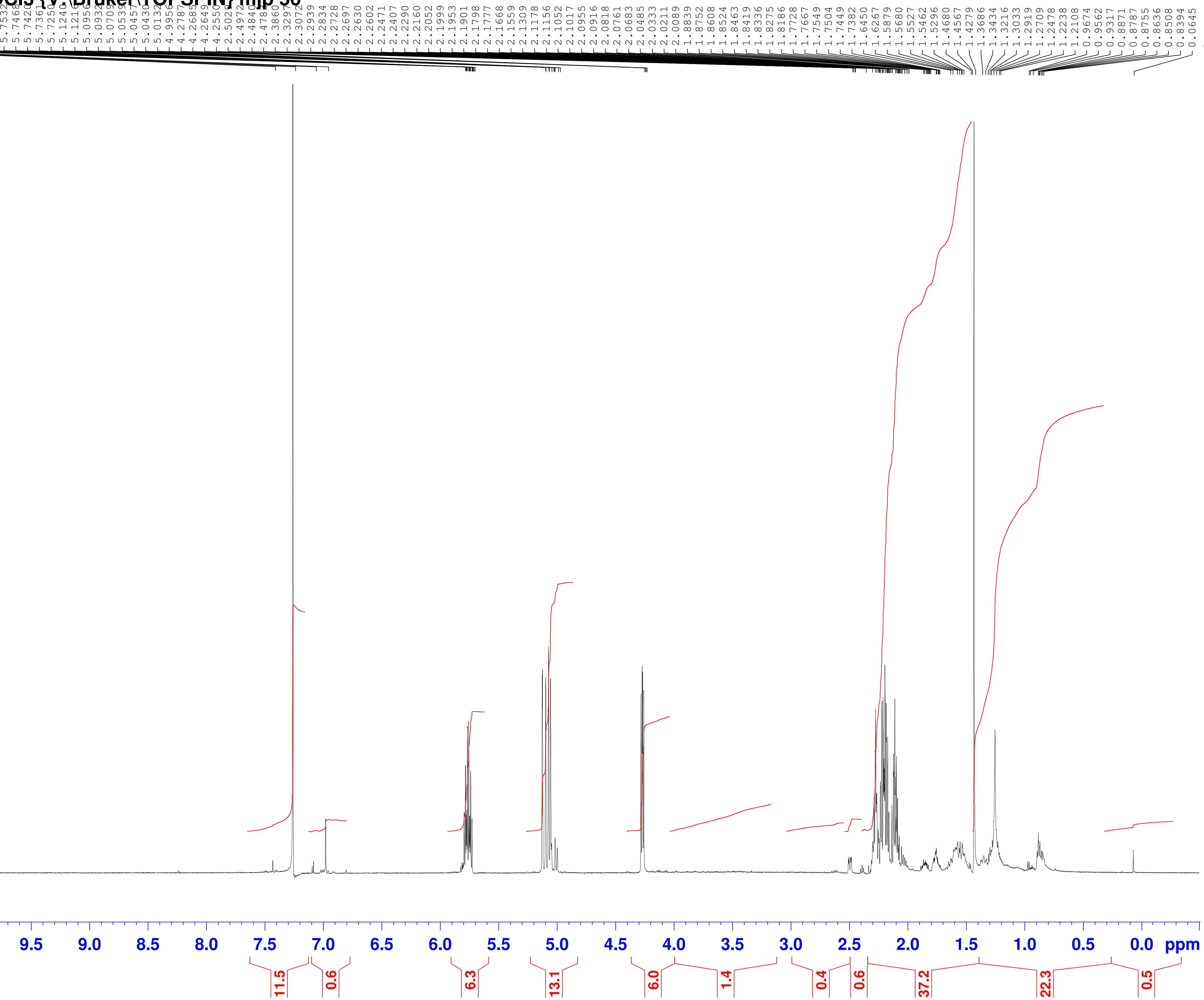


| | |
|---|---|
| NAME | JC-331-2 |
| EXPNO | 10 |
| PROCNO | 1 |
| Date_ | 20120222 |
| Time | 20.24 |
| INSTRUM | AV600 |
| PROBHD | 5 mm CPDCH 13C |
| PULPROG | zg30 |
| TD | 98682 |
| SOLVENT | CDCl3 |
| NS | 8 |
| DS | 0 |
| SWH | 12335.526 Hz |
| FIDRES | 0.125003 Hz |
| AQ | 3.9999604 sec |
| RG | 45.2 |
| DW | 40.533 use |
| DE | 10.48 use |
| TE | 298.0 K |
| D1 | 1.00000000 sec |
| TD0 | 1 |

======== CHANNEL f1 ========

| | |
|---|---|
| NUC1 | 1H |
| P1 | 11.40 use |
| PL1 | 1.00 dB |
| PL1W | 13.76731014 W |
| SFO1 | 600.1337061 MHz |
| SI | 32768 |
| SF | 600.1300116 MHz |
| WDW | EM |
| SSB | 0 |
| LB | 0.30 Hz |
| GB | 0 |
| PC | 1.40 |

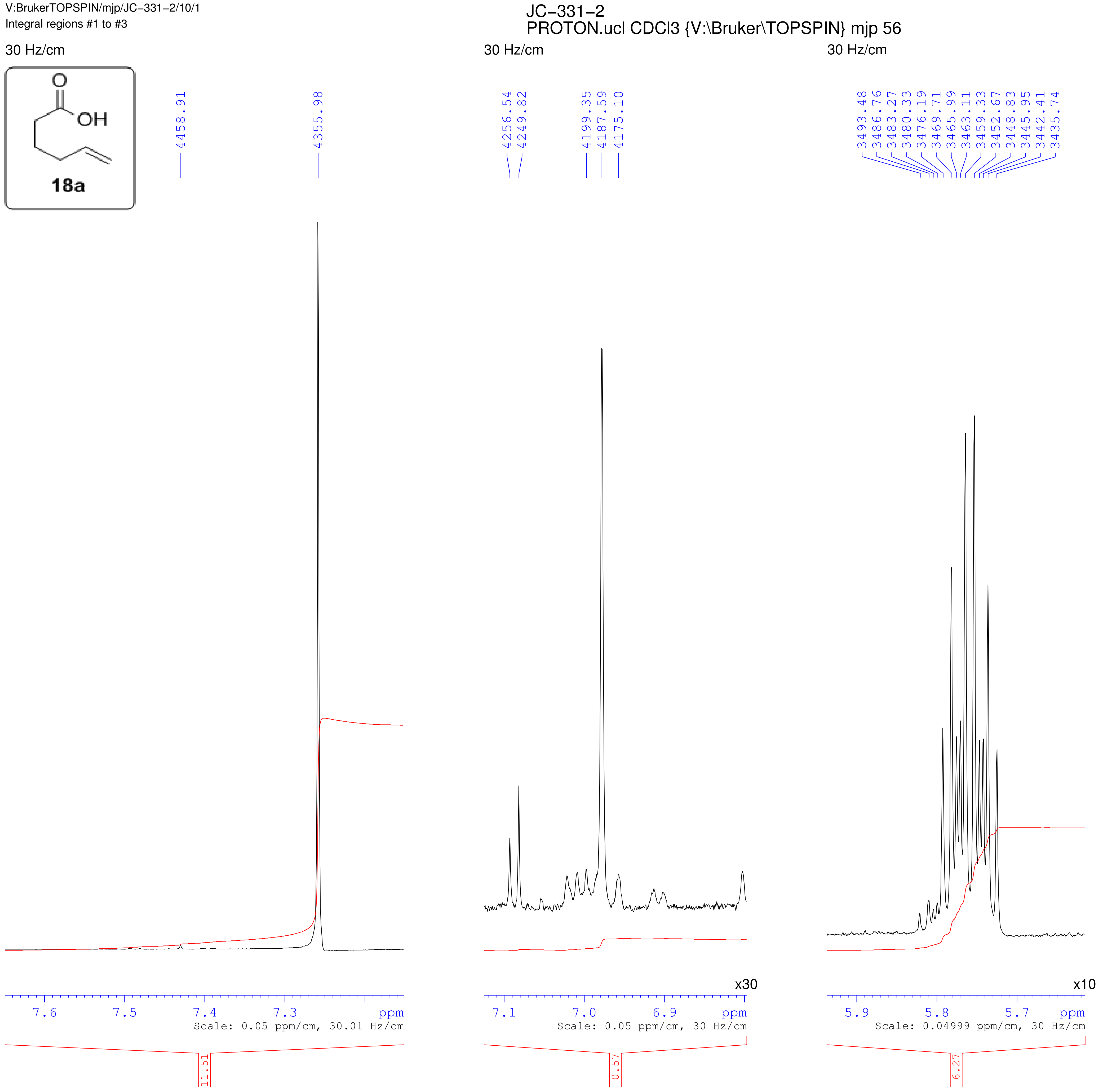

V:BrukerTOPSPIN/mjp/JC−331−2/10/1
Integral regions #1 to #3
JC−331−2
PROTON.ucl CDCl3 {V:\Bruker\TOPSPIN} mjp 56
30 Hz/cm
OH
O
18a
4458.91
4355.98
4256.54
4249.82
4199.35
4187.59
4175.10
3493.48
3486.76
3483.27
3480.33
3476.19
3469.71
3465.99
3463.11
3459.33
3452.67
3448.83
3445.95
3442.41
3435.74
x30
x10
7.6 7.5 7.4 7.3 ppm
Scale: 0.05 ppm/cm, 30.01 Hz/cm
7.1 7.0 6.9 ppm
Scale: 0.05 ppm/cm, 30 Hz/cm
5.9 5.8 5.7 ppm
Scale: 0.04999 ppm/cm, 30 Hz/cm
11.51
0.57
6.27

V:BrukerTOPSPIN/mjp/JC−331−2/10/1
Integral regions #4 to #5

# JC−331−2
PROTON.ucl CDCl3 {V:\Bruker\TOPSPIN} mjp 56

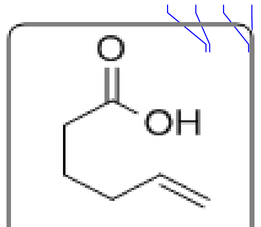

18a

30 Hz/cm

3075.07
3073.63
3058.02
3056.58
3043.20
3033.00
3028.08
3026.46
3008.57
2998.01

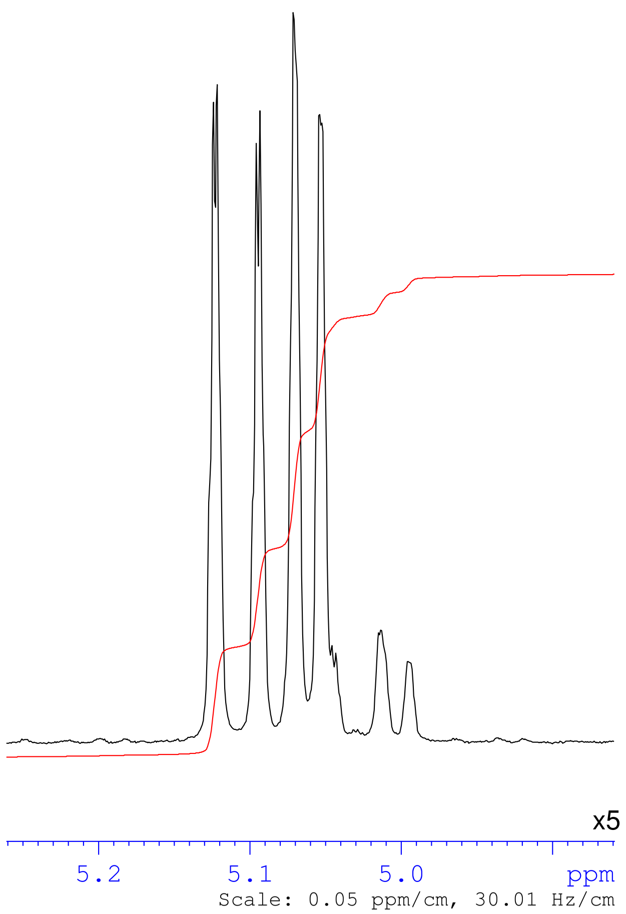


Scale: 0.05 ppm/cm, 30.01 Hz/cm

13.07

30 Hz/cm

2567.78
2561.65
2559.49
2553.55
2476.62

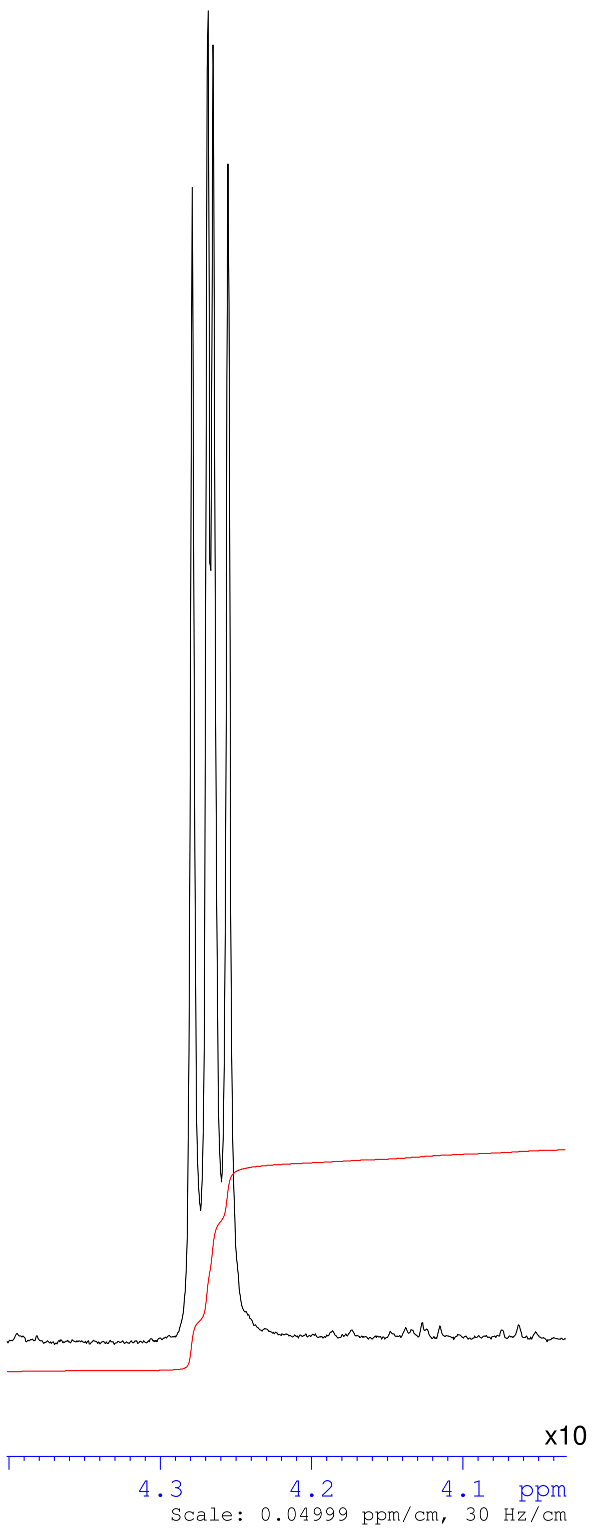


Scale: 0.04999 ppm/cm, 30 Hz/cm

6.02

**JC−331−2**
**C13CPD.ucl CDCl3 {V:\Bruker\TOPSPIN} mjp 56**

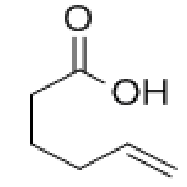

**18a**

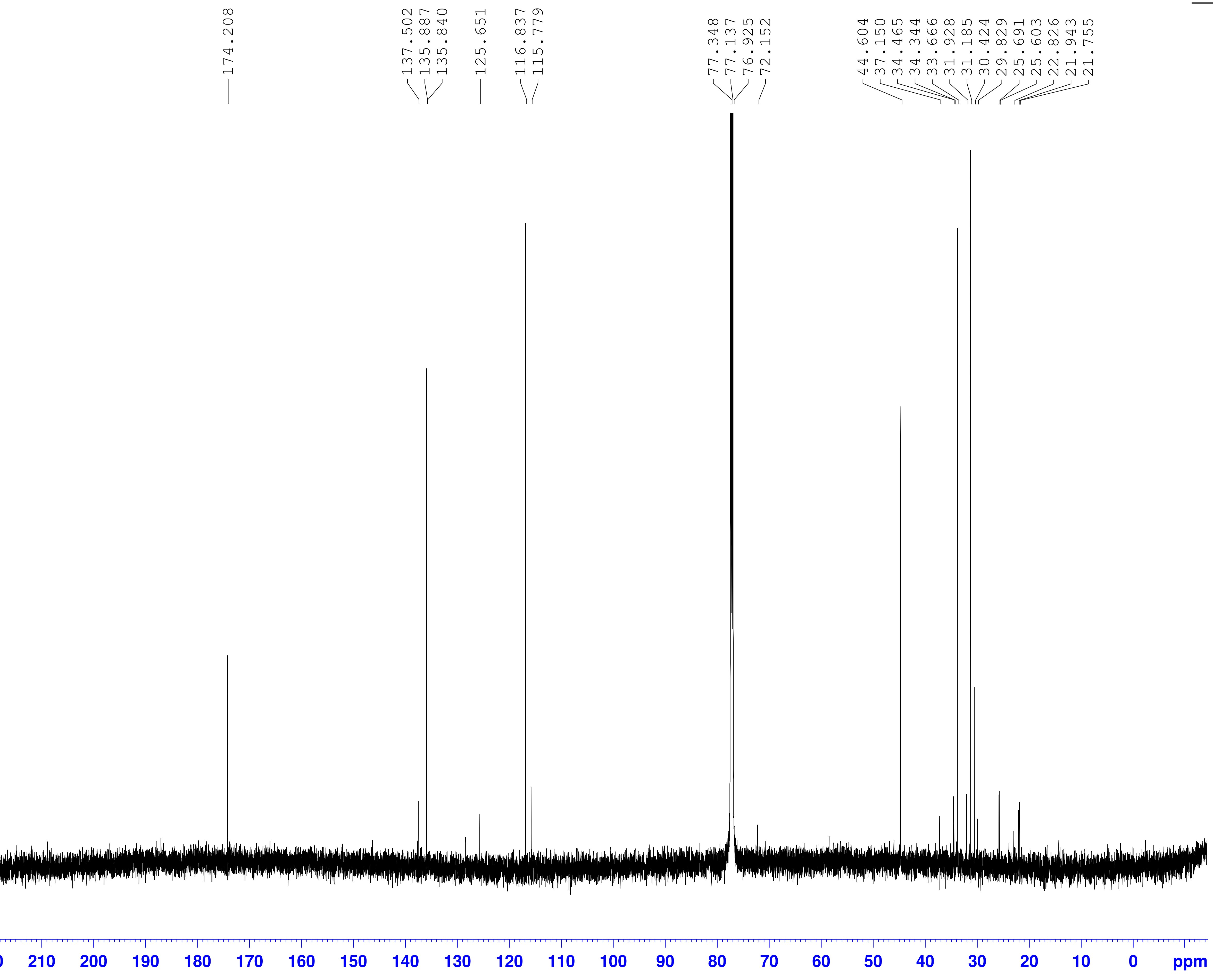


UCL

| | |
|---|---|
| NAME | JC-331-2 |
| EXPNO | 12 |
| PROCNO | 1 |
| Date_ | 20120222 |
| Time | 20.35 |
| INSTRUM | AV600 |
| PROBHD | 5 mm CPDCH 13C |
| PULPROG | zgpg30 |
| TD | 70308 |
| SOLVENT | CDCl3 |
| NS | 128 |
| DS | 0 |
| SWH | 39062.500 H |
| FIDRES | 0.555591 H |
| AQ | 0.8999924 s |
| RG | 1030 |
| DW | 12.800 u |
| DE | 21.12 u |
| TE | 298.0 K |
| D1 | 2.00000000 s |
| D11 | 0.03000000 s |
| TD0 | 1 |
| ======== CHANNEL f1 ===== | |
| NUC1 | 13C |
| P1 | 9.80 u |
| PL1 | 5.00 d |
| PL1W | 26.76886177 W |
| SFO1 | 150.9201628 M |
| ======== CHANNEL f2 ===== | |
| CPDPRG2 | waltz16 |
| NUC2 | 1H |
| PCPD2 | 70.00 u |
| PL2 | 1.00 d |
| PL12 | 17.23 d |
| PL13 | 20.00 d |
| PL2W | 13.76731014 W |
| PL12W | 0.32798135 W |
| PL13W | 0.17332016 W |
| SFO2 | 600.1324005 M |
| SI | 65536 |
| SF | 150.9027930 M |
| WDW | EM |
| SSB | 0 |
| LB | 1.00 H |
| GB | 0 |
| PC | 1.40 |

JC–331–2
COSY.ucl CDCl3 {V:\Bruker\TOPSPIN} mjp 56

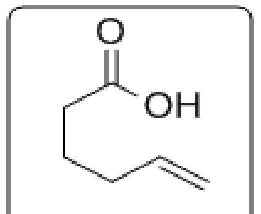

**18a**

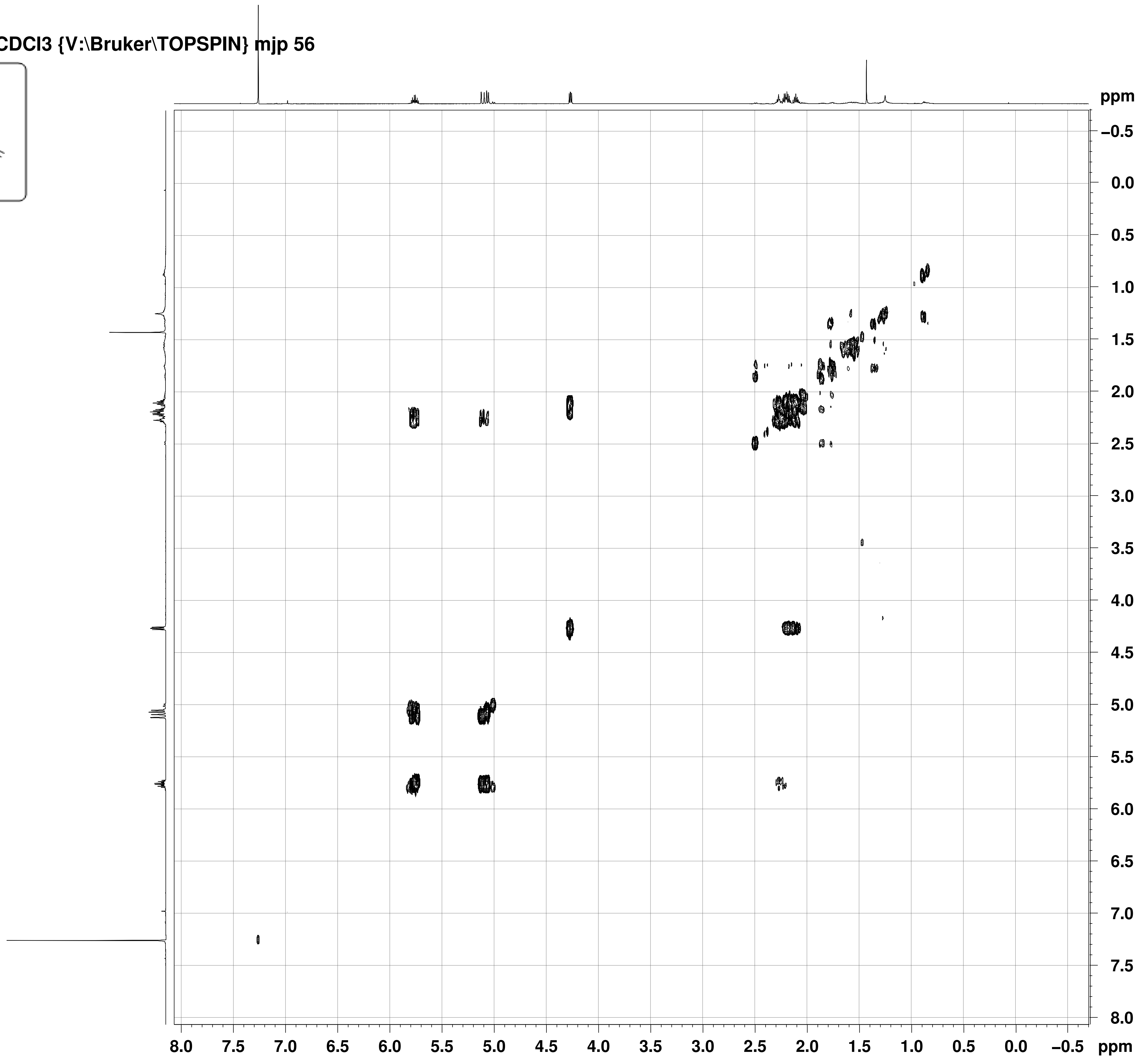


| | |
|---|---|
| NAME | JC-331-2 |
| EXPNO | 11 |
| PROCNO | 1 |
| Date_ | 20120222 |
| Time | 20.24 |
| INSTRUM | AV600 |
| PROBHD | 5 mm CPDCH 13C |
| PULPROG | cosygpmfqf |
| TD | 2048 |
| SOLVENT | CDCl3 |
| NS | 1 |
| DS | 8 |
| SWH | 5263.158 Hz |
| FIDRES | 2.569901 Hz |
| AQ | 0.1946100 sec |
| RG | 2050 |
| DW | 95.000 usec |
| DE | 6.50 usec |
| TE | 298.0 K |
| D0 | 0.00000300 sec |
| D1 | 1.70549703 sec |
| D13 | 0.00000400 sec |
| D16 | 0.00020000 sec |
| IN0 | 0.00019000 sec |
| ======== CHANNEL f1 ======== | |
| NUC1 | 1H |
| P1 | 11.40 usec |
| PL1 | 1.00 dB |
| PL1W | 13.76731014 W |
| SFO1 | 600.1322217 MHz |
| ====== GRADIENT CHANNEL ==== | |
| GPNAM1 | SINE.100 |
| GPNAM2 | SINE.100 |
| GPNAM3 | SINE.100 |
| GPZ1 | 16.00 % |
| GPZ2 | 12.00 % |
| GPZ3 | 40.00 % |
| P16 | 1000.00 usec |
| ND0 | 1 |
| TD | 128 |
| SFO1 | 600.1322 MHz |
| FIDRES | 41.118420 Hz |
| SW | 8.770 ppm |
| FnMODE | QF |
| SI | 1024 |
| SF | 600.1300092 MHz |
| WDW | QSINE |
| SSB | 0 |
| LB | 0.00 Hz |
| GB | 0 |
| PC | 1.40 |
| SI | 1024 |
| MC2 | QF |
| SF | 600.1300092 MHz |
| WDW | QSINE |
| SSB | 0 |
| LB | 0.00 Hz |
| GB | 0 |

JC–331–2
HSQC.ucl CDCl3 {V:\Bruker\TOPSPIN} mjp 56

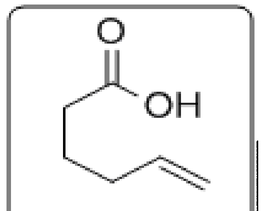


18a

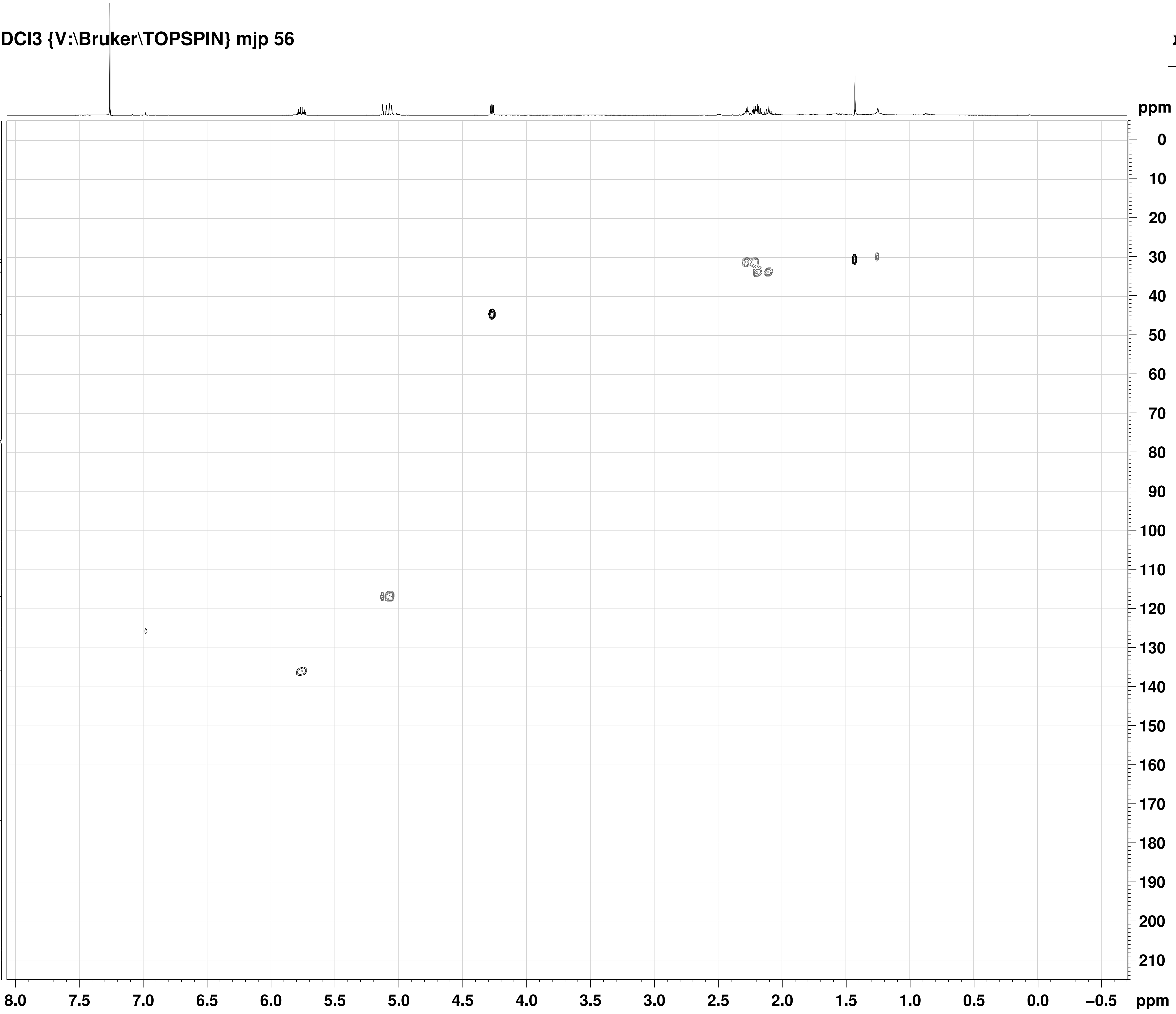


```
NAME          JC-331-2
EXPNO               13
PROCNO               1
Date_         20120222
Time             20.36
INSTRUM          AV600
PROBHD   5 mm CPDCH 13C
PULPROG  hsqcedetgpsisp2.4
TD                1024
SOLVENT          CDCl3
NS                   2
DS                  32
SWH           5263.158 Hz
FIDRES        5.139802 Hz
AQ           0.0973300 sec
RG                2050
DW              95.000 usec
DE                6.50 usec
TE               298.0 K
CNST2      145.0000000
CNST17      -0.5000000
D0          0.00000300 sec
D1          1.48791695 sec
D2          0.00344828 sec
D4          0.00172414 sec
D11         0.03000000 sec
D16         0.00020000 sec
D21         0.00344828 sec
D24         0.00086207 sec
IN0         0.00001505 sec
L0                   0
L31                  1
TD0                  2

======== CHANNEL f1 ========
NUC1                1H
P1               11.40 usec
P2               22.80 usec
P28               0.00 usec
PL1               1.00 dB
PL1W       13.76731014 W
SFO1       600.1322217 MHz

======== CHANNEL f2 ========
CPDPRG2  bi_p5m4sp_4sp.2
NUC2               13C
P3                9.80 usec
P14             500.00 usec
P24            2000.00 usec
P31            1730.00 usec
P63            1500.00 usec
PL0             120.00 dB
PL2               5.00 dB
PL12             20.74 dB
PL0W        0.00000000 W
PL2W       26.76886177 W
PL12W       0.71388775 W
SFO2       150.9186538 MHz
SP3              13.33 dB
SP7              13.33 dB
SP14             14.82 dB
SP18             18.73 dB
SP31             20.84 dB
SPNAM3   Crp60,0.5,20.1
SPNAM7     Crp60comp.4
SPNAM14  Crp32,1.5,20.2
SPNAM18   Crp60_xfilt.2
SPNAM31  Crp32,1.5,20.2
SPOAL3           0.500
SPOAL7           0.500
SPOAL14          0.500
SPOAL18          0.500
SPOAL31          0.500
SPOFFS3           0.00 Hz
SPOFFS7           0.00 Hz
SPOFFS14          0.00 Hz
SPOFFS18          0.00 Hz
SPOFFS31          0.00 Hz

====== GRADIENT CHANNEL =====
GPNAM1        SINE.100
GPNAM2        SINE.100
GPNAM3        SINE.100
GPNAM4        SINE.100
GPZ1             80.00 %
GPZ2             20.10 %
GPZ3             11.00 %
GPZ4             -5.00 %
P16            1000.00 usec
P19             600.00 usec
ND0                  2
TD                 128
SFO1          150.9187 MHz
FIDRES      259.391449 Hz
SW             220.000 ppm
FnMODE    Echo-Antiecho
SI                1024
SF         600.1300092 MHz
WDW              QSINE
SSB                  2
LB                0.00 Hz
GB                   0
PC                1.40
SI                1024
MC2       echo-antiecho
SF         150.9027771 MHz
WDW              QSINE
SSB                  2
LB                0.00 Hz
GB                   0
```

**JC–331–2**
**HMBC.ucl CDCl3 {V:\Bruker\TOPSPIN} mjp 56**

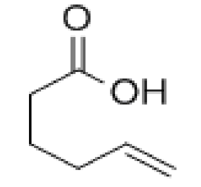

**18a**

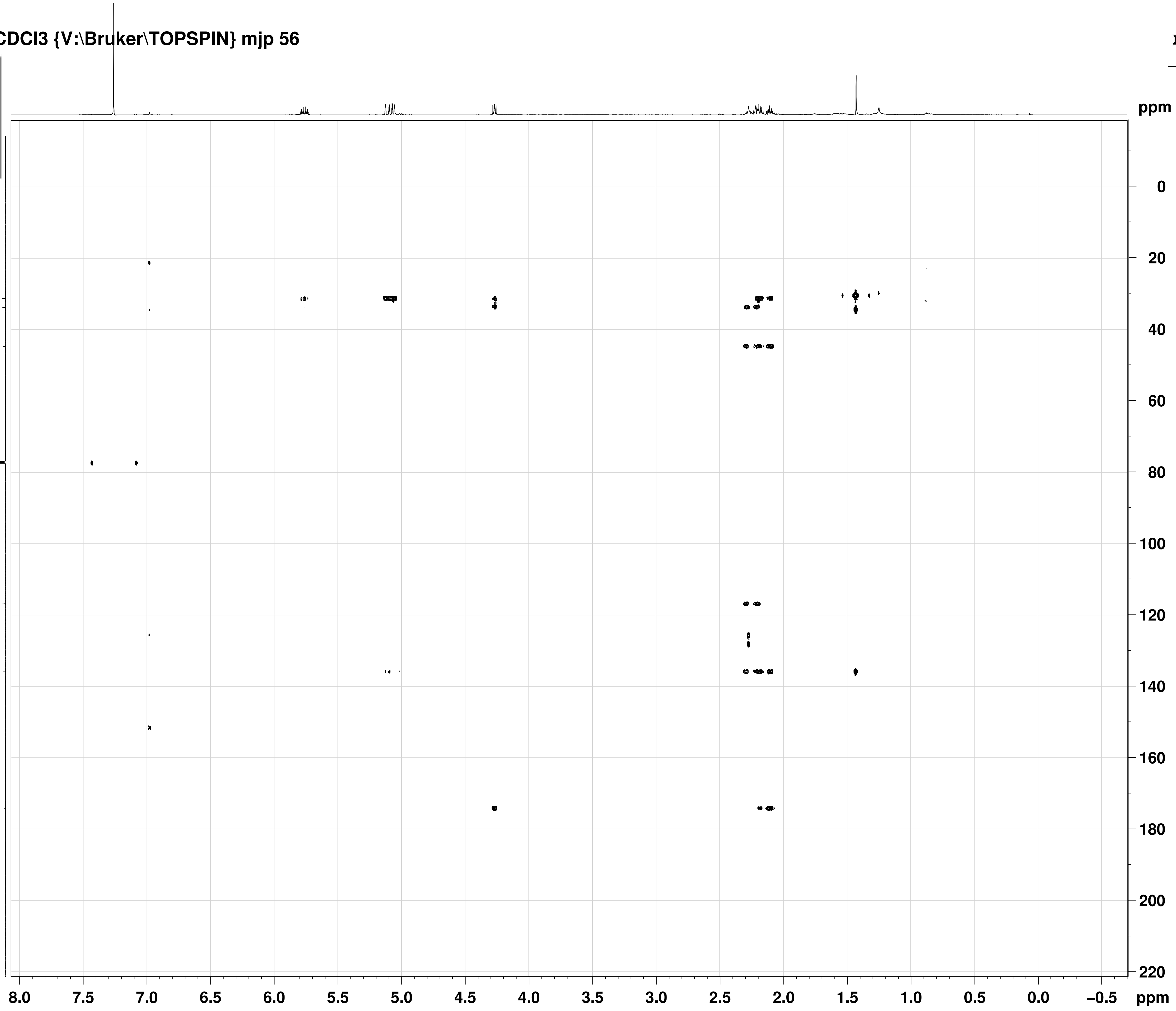


```
NAME            JC-331-2
EXPNO                 14
PROCNO                 1
Date_           20120222
Time               20.44
INSTRUM            AV600
PROBHD   5 mm CPDCH 13C
PULPROG    hmbcetgpl3nd
TD                  4096
SOLVENT            CDCl3
NS                     2
DS                    16
SWH             5263.158 Hz
FIDRES          1.284951 Hz
AQ             0.3891700 sec
RG                  2050
DW                95.000 usec
DE                  6.50 usec
TE                 298.0 K
CNST6        120.0000000
CNST7        160.0000000
CNST13        10.0000000
CNST30         0.5981150
D0            0.00000300 sec
D1            0.78867459 sec
D6            0.05000000 sec
D16           0.00020000 sec
IN0           0.00001380 sec

======== CHANNEL f1 ========
NUC1                  1H
P1                 11.40 usec
P2                 22.80 usec
PL1                 1.00 dB
PL1W         13.76731014 W
SFO1         600.1322217 MHz

======== CHANNEL f2 ========
NUC2                 13C
P3                  9.80 usec
P24              2000.00 usec
PL2                 5.00 dB
PL2W         26.76886177 W
SFO2         150.9178993 MHz
SP7                13.33 dB
SPNAM7       Crp60comp.4
SPOAL7             0.500
SPOFFS7             0.00 Hz

====== GRADIENT CHANNEL =====
GPNAM1          SINE.100
GPNAM3          SINE.100
GPNAM4          SINE.100
GPNAM5          SINE.100
GPNAM6          SINE.100
GPZ1               80.00 %
GPZ3               14.00 %
GPZ4               -8.00 %
GPZ5               -4.00 %
GPZ6               -2.00 %
P16              1000.00 usec
ND0                    2
TD                   256
SFO1            150.9179 MHz
FIDRES        141.485535 Hz
SW               240.000 ppm
FnMODE      Echo-Antiecho
SI                  2048
SF           600.1300105 MHz
WDW                 SINE
SSB                    2
LB                  0.00 Hz
GB                     0
PC                  1.40
SI                  1024
MC2         echo-antiecho
SF           150.9027756 MHz
WDW                 SINE
SSB                    2
LB                  0.00 Hz
GB                     0
```

JC–332–1
PROTON.ucl CDCl3 {V:\Bruker\TOPSPIN} mjp 6

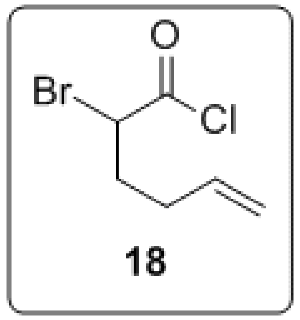


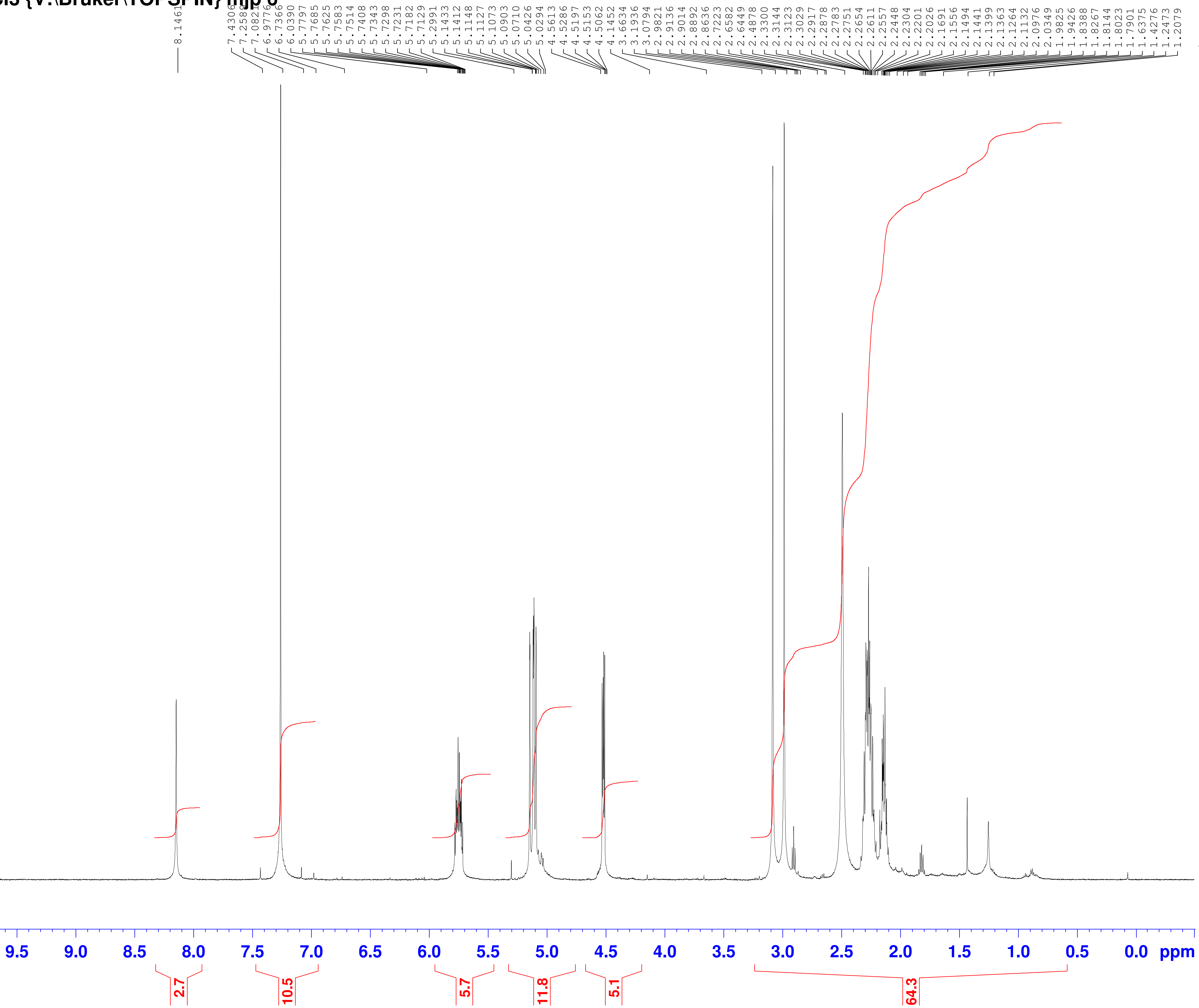


UCL

| | |
|---|---|
| NAME | JC-332-1 |
| EXPNO | 10 |
| PROCNO | 1 |
| Date_ | 20120228 |
| Time | 16.53 |
| INSTRUM | AV600 |
| PROBHD | 5 mm CPDCH 13C |
| PULPROG | zg30 |
| TD | 98682 |
| SOLVENT | CDCl3 |
| NS | 8 |
| DS | 0 |
| SWH | 12335.526 Hz |
| FIDRES | 0.125003 Hz |
| AQ | 3.9999604 sec |
| RG | 36 |
| DW | 40.533 use |
| DE | 10.48 use |
| TE | 298.0 K |
| D1 | 1.00000000 sec |
| TD0 | 1 |
| ======== CHANNEL f1 ======= | |
| NUC1 | 1H |
| P1 | 11.40 use |
| PL1 | 1.00 dB |
| PL1W | 13.76731014 W |
| SFO1 | 600.1337061 MHz |
| SI | 32768 |
| SF | 600.1300116 MHz |
| WDW | EM |
| SSB | 0 |
| LB | 0.30 Hz |
| GB | 0 |
| PC | 1.40 |

V:BrukerTOPSPIN/mjp/JC–332–1/10/1
Integral regions #1 to #3

JC–332–1
PROTON.ucl CDCl3 {V:\Bruker\TOPSPIN} mjp 6

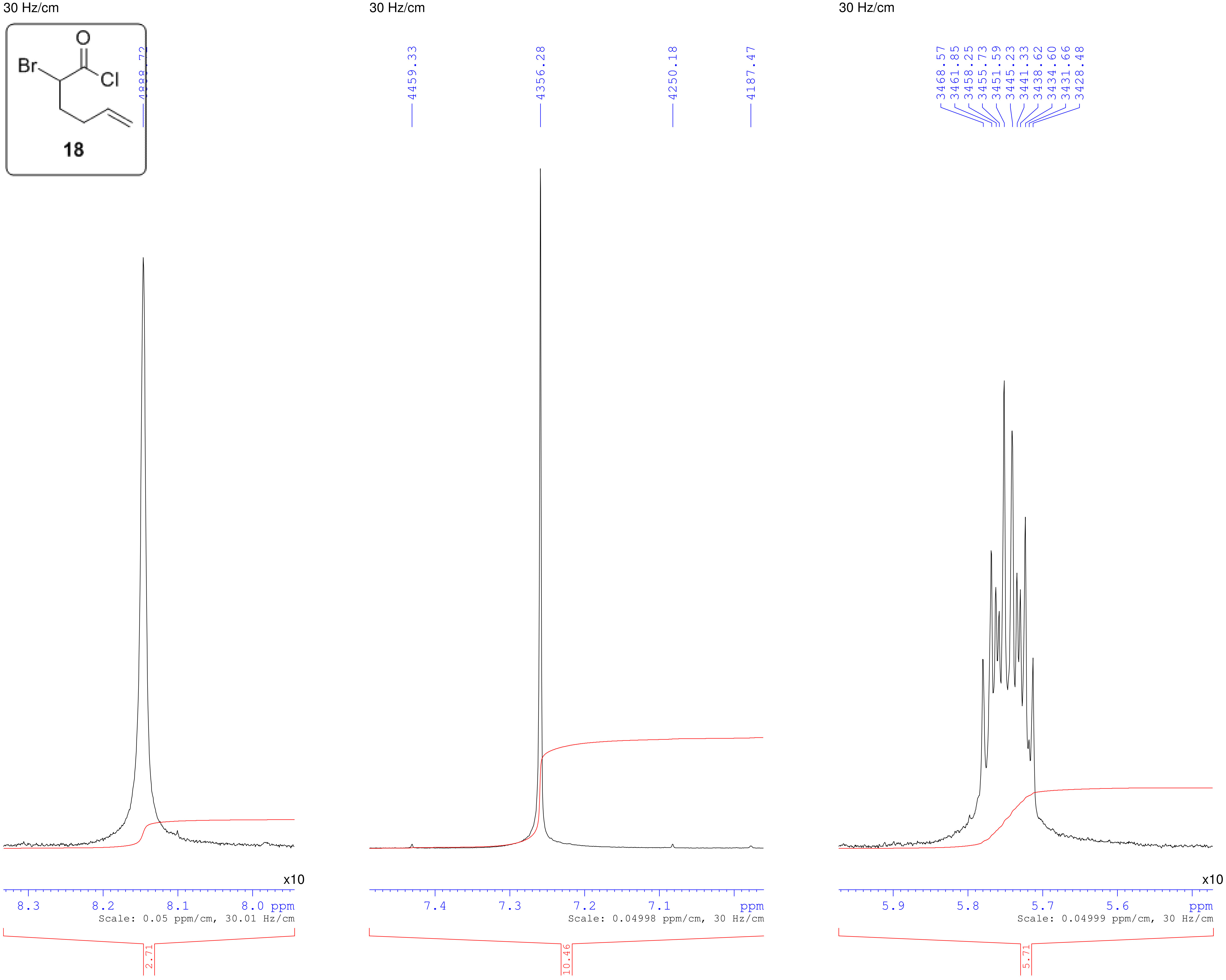

V:BrukerTOPSPIN/mjp/JC−332−1/10/1
Integral regions #4 to #5

30 Hz/cm

JC−332−1
PROTON.ucl CDCl3 {V:\Bruker\TOPSPIN} mjp 6

30 Hz/cm

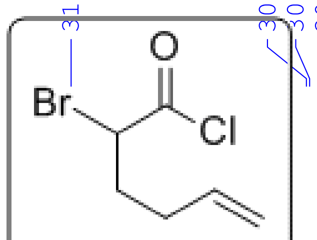


**18**

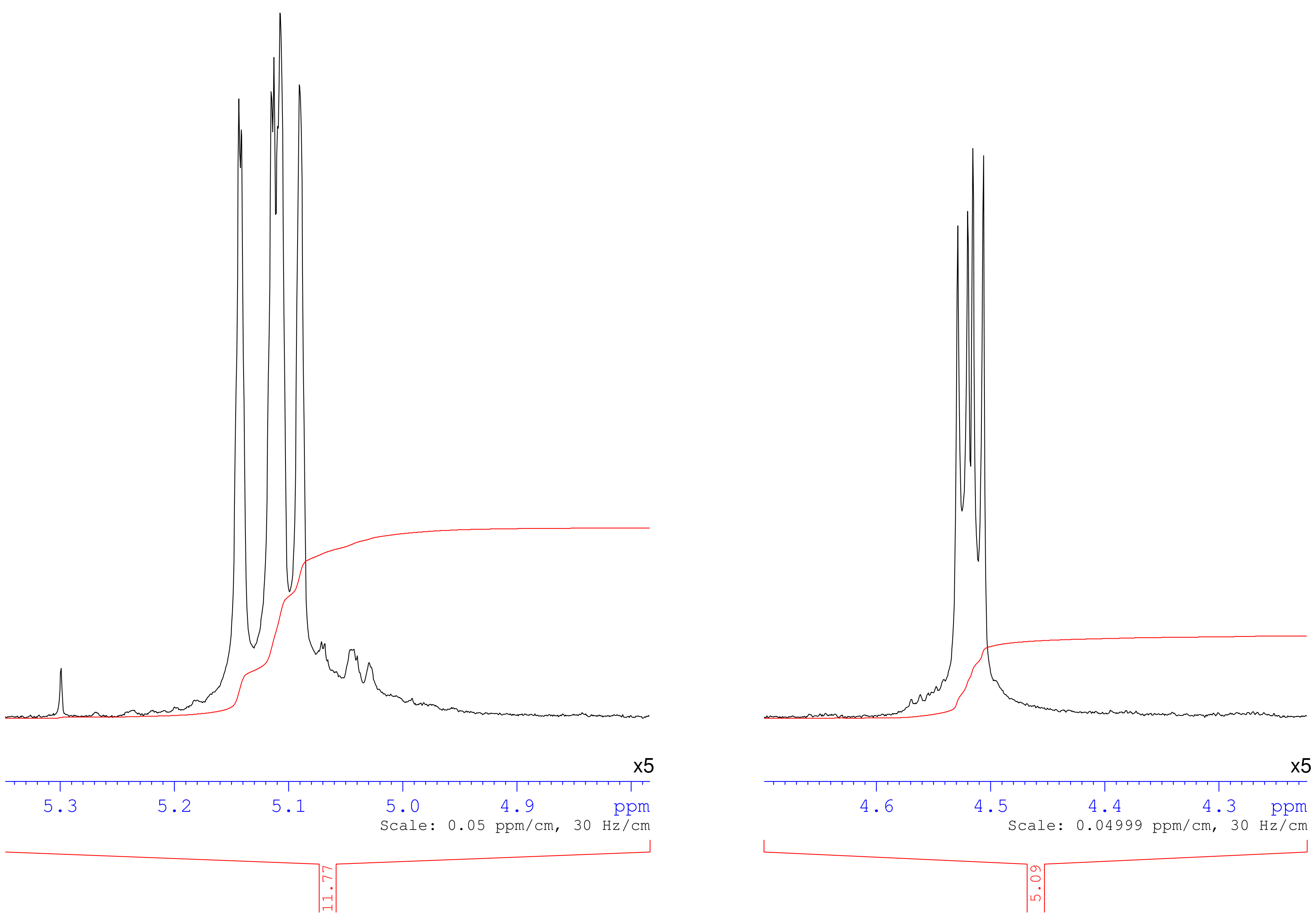

JC–332–1
C13CPD.ucl CDCl3 {V:\Bruker\TOPSPIN} mjp 6

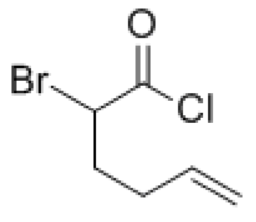

18

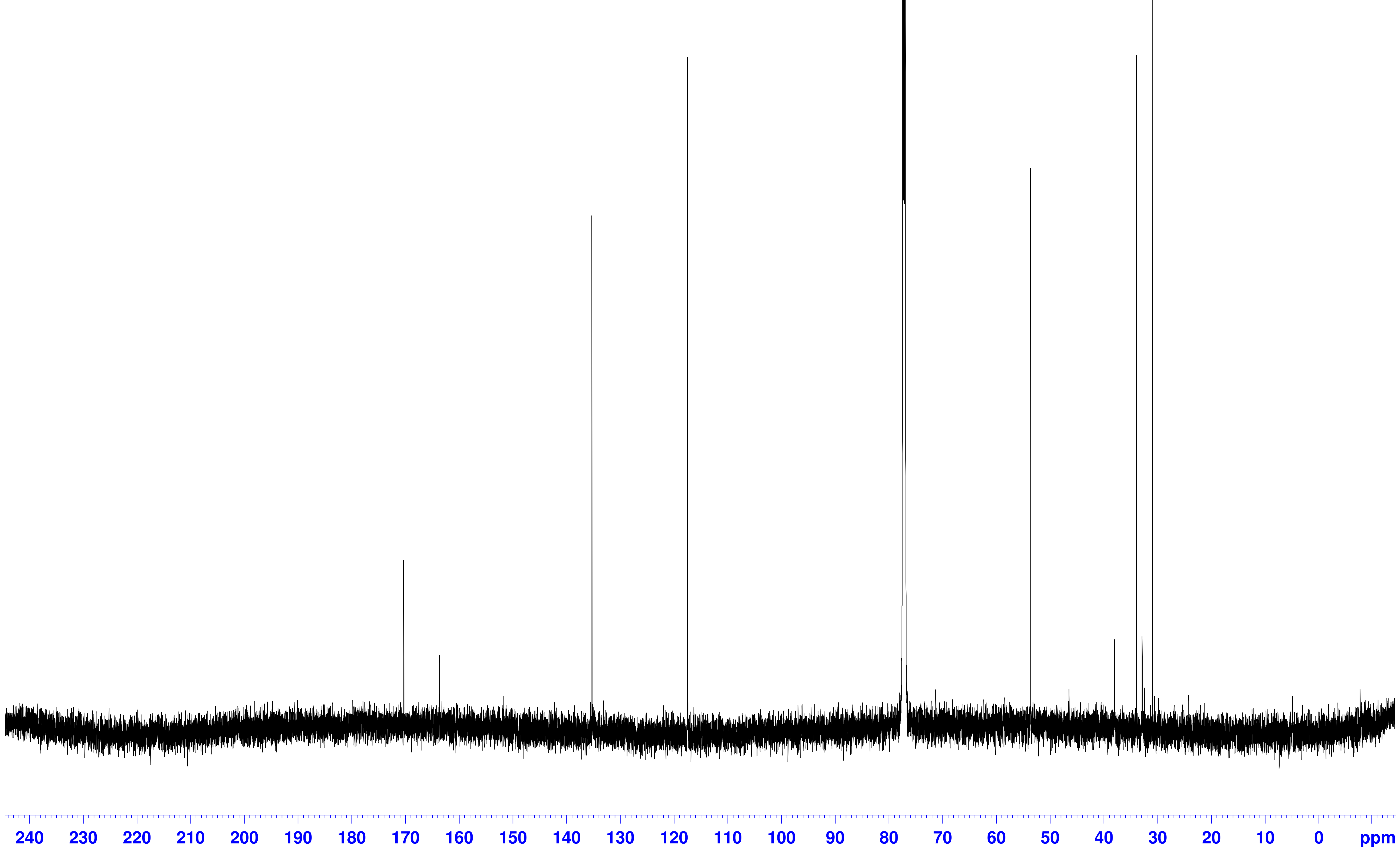


| | |
|---|---|
| NAME | JC-332-1 |
| EXPNO | 12 |
| PROCNO | 1 |
| Date_ | 20120228 |
| Time | 17.57 |
| INSTRUM | AV600 |
| PROBHD | 5 mm CPDCH 13C |
| PULPROG | zgpg30 |
| TD | 70308 |
| SOLVENT | CDCl3 |
| NS | 128 |
| DS | 0 |
| SWH | 39062.500 H |
| FIDRES | 0.555591 H |
| AQ | 0.8999924 s |
| RG | 1030 |
| DW | 12.800 u |
| DE | 21.12 u |
| TE | 298.0 K |
| D1 | 2.00000000 s |
| D11 | 0.03000000 s |
| TD0 | 1 |
| ======== CHANNEL f1 ===== | |
| NUC1 | 13C |
| P1 | 9.80 u |
| PL1 | 5.00 d |
| PL1W | 26.76886177 W |
| SFO1 | 150.9201628 M |
| ======== CHANNEL f2 ===== | |
| CPDPRG2 | waltz16 |
| NUC2 | 1H |
| PCPD2 | 70.00 u |
| PL2 | 1.00 d |
| PL12 | 17.23 d |
| PL13 | 20.00 d |
| PL2W | 13.76731014 W |
| PL12W | 0.32798135 W |
| PL13W | 0.17332016 W |
| SFO2 | 600.1324005 M |
| SI | 65536 |
| SF | 150.9027930 M |
| WDW | EM |
| SSB | 0 |
| LB | 1.00 H |
| GB | 0 |
| PC | 1.40 |

JC–332–1
COSY.ucl CDCl3 {V:\Bruker\TOPSPIN} mjp 6

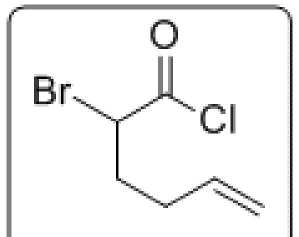

18

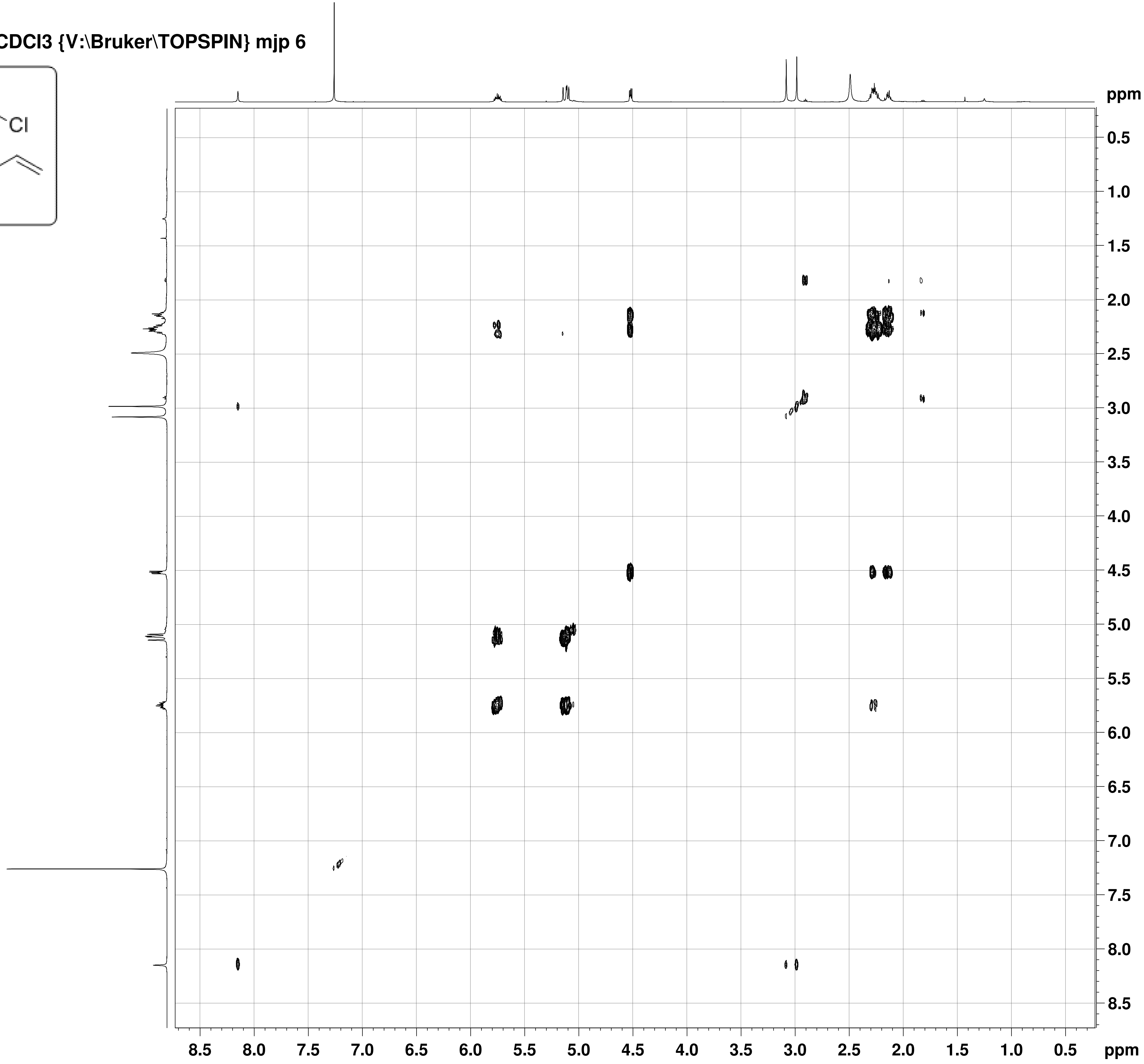


| | | |
|---|---|---|
| NAME | JC-332-1 | |
| EXPNO | 11 | |
| PROCNO | 1 | |
| Date_ | 20120228 | |
| Time | 17.46 | |
| INSTRUM | AV600 | |
| PROBHD | 5 mm CPDCH 13C | |
| PULPROG | cosygpmfqf | |
| TD | 2048 | |
| SOLVENT | CDCl3 | |
| NS | 1 | |
| DS | 8 | |
| SWH | 5102.041 | Hz |
| FIDRES | 2.491231 | Hz |
| AQ | 0.2007540 | sec |
| RG | 2050 | |
| DW | 98.000 | usec |
| DE | 6.50 | usec |
| TE | 298.0 | K |
| D0 | 0.00000300 | sec |
| D1 | 1.69935298 | sec |
| D13 | 0.00000400 | sec |
| D16 | 0.00020000 | sec |
| IN0 | 0.00019600 | sec |
| ======== CHANNEL f1 ======== | | |
| NUC1 | 1H | |
| P1 | 11.40 | usec |
| PL1 | 1.00 | dB |
| PL1W | 13.76731014 | W |
| SFO1 | 600.1326990 | MHz |
| ====== GRADIENT CHANNEL ==== | | |
| GPNAM1 | SINE.100 | |
| GPNAM2 | SINE.100 | |
| GPNAM3 | SINE.100 | |
| GPZ1 | 16.00 | % |
| GPZ2 | 12.00 | % |
| GPZ3 | 40.00 | % |
| P16 | 1000.00 | usec |
| ND0 | 1 | |
| TD | 128 | |
| SFO1 | 600.1327 | MHz |
| FIDRES | 39.859695 | Hz |
| SW | 8.502 | ppm |
| FnMODE | QF | |
| SI | 1024 | |
| SF | 600.1300093 | MHz |
| WDW | QSINE | |
| SSB | 0 | |
| LB | 0.00 | Hz |
| GB | 0 | |
| PC | 1.40 | |
| SI | 1024 | |
| MC2 | QF | |
| SF | 600.1300093 | MHz |
| WDW | QSINE | |
| SSB | 0 | |
| LB | 0.00 | Hz |
| GB | 0 | |

**JC–332–1**
**C13DEPT135.ucl CDCl3 {V:\Bruker\TOPSPIN} mjp 6**

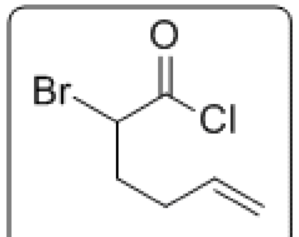


**18**

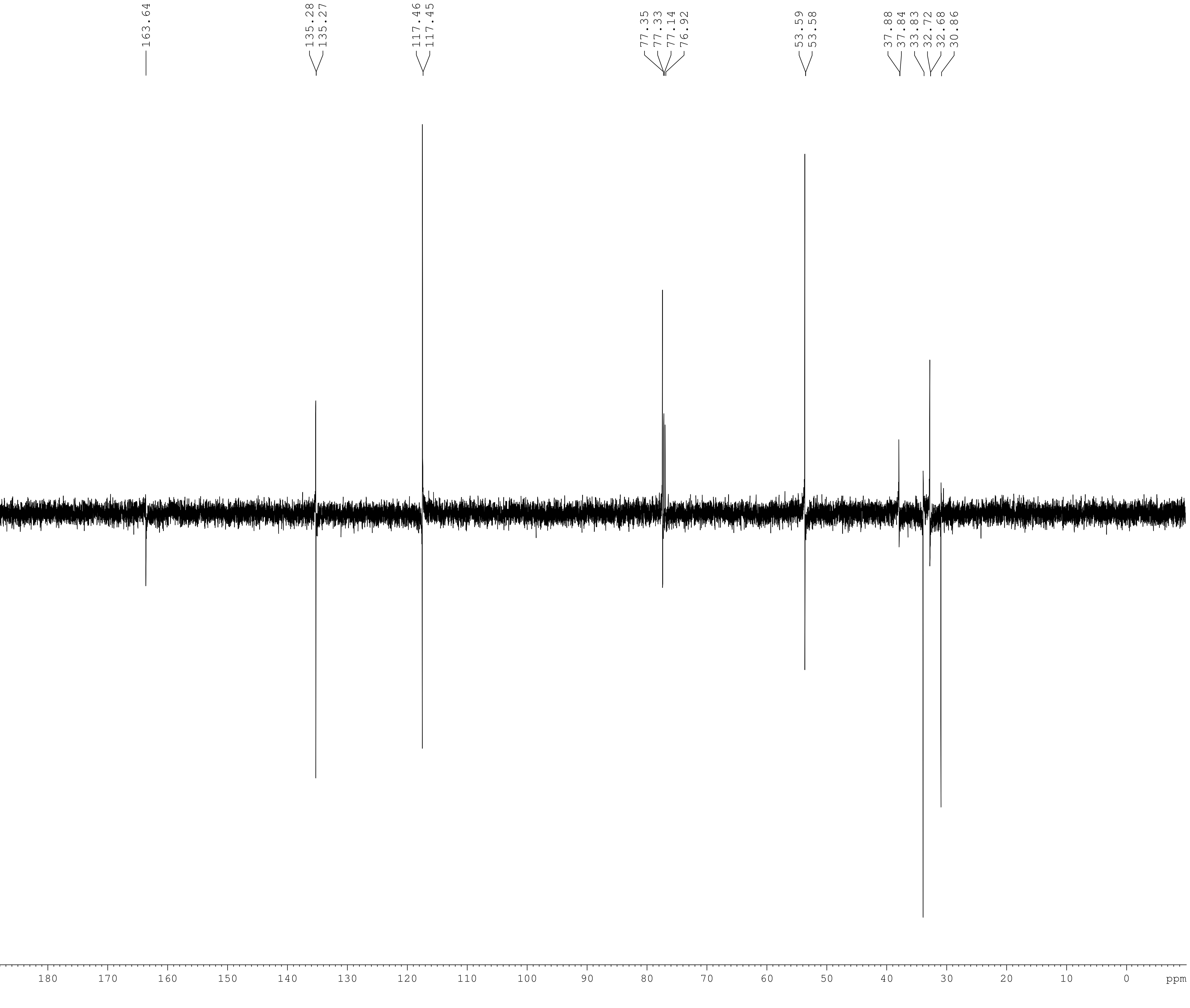


```
NAME          JC-332-1
EXPNO               15
PROCNO               1
Date_         20120228
Time             20.06
INSTRUM          AV600
PROBHD   5 mm CPDCH 13C
PULPROG        dept135
TD               70308
SOLVENT          CDCl3
NS                  64
DS                   4
SWH          39062.500 H
FIDRES        0.555591 H
AQ           0.8999924 s
RG                 256
DW              12.800 u
DE                6.50 u
TE               298.0 K
CNST2      145.0000000
D1          2.00000000 s
D2          0.00344828 s
D12         0.00002000 s
TD0                  1

======== CHANNEL f1 =====
NUC1               13C
P1                9.80 u
P2               19.60 u
PL1               5.00 d
PL1W       26.76886177 W
SFO1       150.9201628 M

======== CHANNEL f2 =====
CPDPRG2        waltz16
NUC2                1H
P3               10.80 u
P4               21.60 u
PCPD2            70.00 u
PL2               1.00 d
PL12             17.23 d
PL2W       13.76731014 W
PL12W       0.32798135 W
SFO2       600.1324005 M
SI               65536
SF         150.9027930 M
WDW                 EM
SSB                  0
LB                1.00 H
GB                   0
PC                1.40
```

JC–332–1
HSQC.ucl CDCl3 {V:\Bruker\TOPSPIN} mjp 6

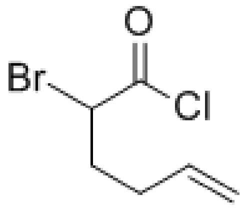


**18**

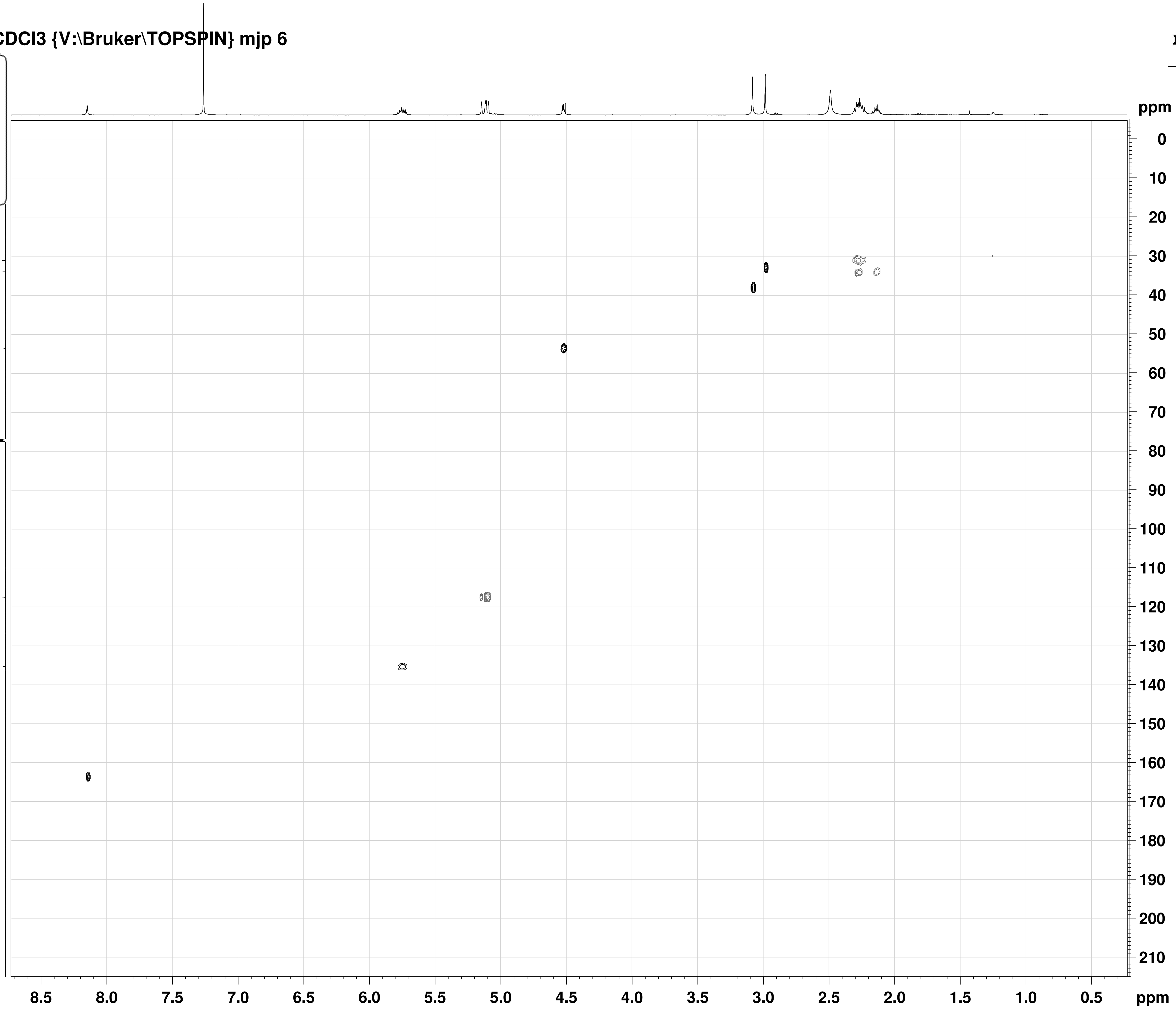


```
NAME              JC-332-1
EXPNO                   13
PROCNO                   1
Date_             20120228
Time                 19.44
INSTRUM              AV600
PROBHD   5 mm CPDCH 13C
PULPROG  hsqcedetgpsisp2.4
TD                    1024
SOLVENT              CDCl3
NS                       2
DS                      32
SWH               5102.041 Hz
FIDRES            4.982462 Hz
AQ               0.1004020 sec
RG                    2050
DW                  98.000 usec
DE                    6.50 usec
TE                   298.0 K
CNST2        145.0000000
CNST17        -0.5000000
D0            0.00000300 sec
D1            1.48484504 sec
D2            0.00344828 sec
D4            0.00172414 sec
D11           0.03000000 sec
D16           0.00020000 sec
D21           0.00344828 sec
D24           0.00086207 sec
IN0           0.00001505 sec
L0                       0
L31                      1
TD0                      2

======== CHANNEL f1 ========
NUC1                    1H
P1                   11.40 usec
P2                   22.80 usec
P28                   0.00 usec
PL1                   1.00 dB
PL1W          13.76731014 W
SFO1         600.1326990 MHz

======== CHANNEL f2 ========
CPDPRG2  bi_p5m4sp_4sp.2
NUC2                   13C
P3                    9.80 usec
P14                 500.00 usec
P24                2000.00 usec
P31                1730.00 usec
P63                1500.00 usec
PL0                 120.00 dB
PL2                   5.00 dB
PL12                 20.74 dB
PL0W           0.00000000 W
PL2W          26.76886177 W
PL12W          0.71388775 W
SFO2         150.9186538 MHz
SP3                  13.33 dB
SP7                  13.33 dB
SP14                 14.82 dB
SP18                 18.73 dB
SP31                 20.84 dB
SPNAM3    Crp60,0.5,20.1
SPNAM7        Crp60comp.4
SPNAM14   Crp32,1.5,20.2
SPNAM18    Crp60_xfilt.2
SPNAM31   Crp32,1.5,20.2
SPOAL3               0.500
SPOAL7               0.500
SPOAL14              0.500
SPOAL18              0.500
SPOAL31              0.500
SPOFFS3               0.00 Hz
SPOFFS7               0.00 Hz
SPOFFS14              0.00 Hz
SPOFFS18              0.00 Hz
SPOFFS31              0.00 Hz

====== GRADIENT CHANNEL =====
GPNAM1            SINE.100
GPNAM2            SINE.100
GPNAM3            SINE.100
GPNAM4            SINE.100
GPZ1                 80.00 %
GPZ2                 20.10 %
GPZ3                 11.00 %
GPZ4                 -5.00 %
P16                1000.00 usec
P19                 600.00 usec
ND0                      2
TD                     128
SFO1              150.9187 MHz
FIDRES          259.391449 Hz
SW                 220.000 ppm
FnMODE        Echo-Antiecho
SI                    1024
SF             600.1300093 MHz
WDW                  QSINE
SSB                      2
LB                    0.00 Hz
GB                       0
PC                    1.40
SI                    1024
MC2           echo-antiecho
SF             150.9027771 MHz
WDW                  QSINE
SSB                      2
LB                    0.00 Hz
GB                       0
```

**JC–332–1**
**HMBC.ucl CDCl3 {V:\Bruker\TOPSPIN} mjp 6**

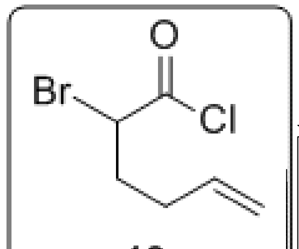

**18**

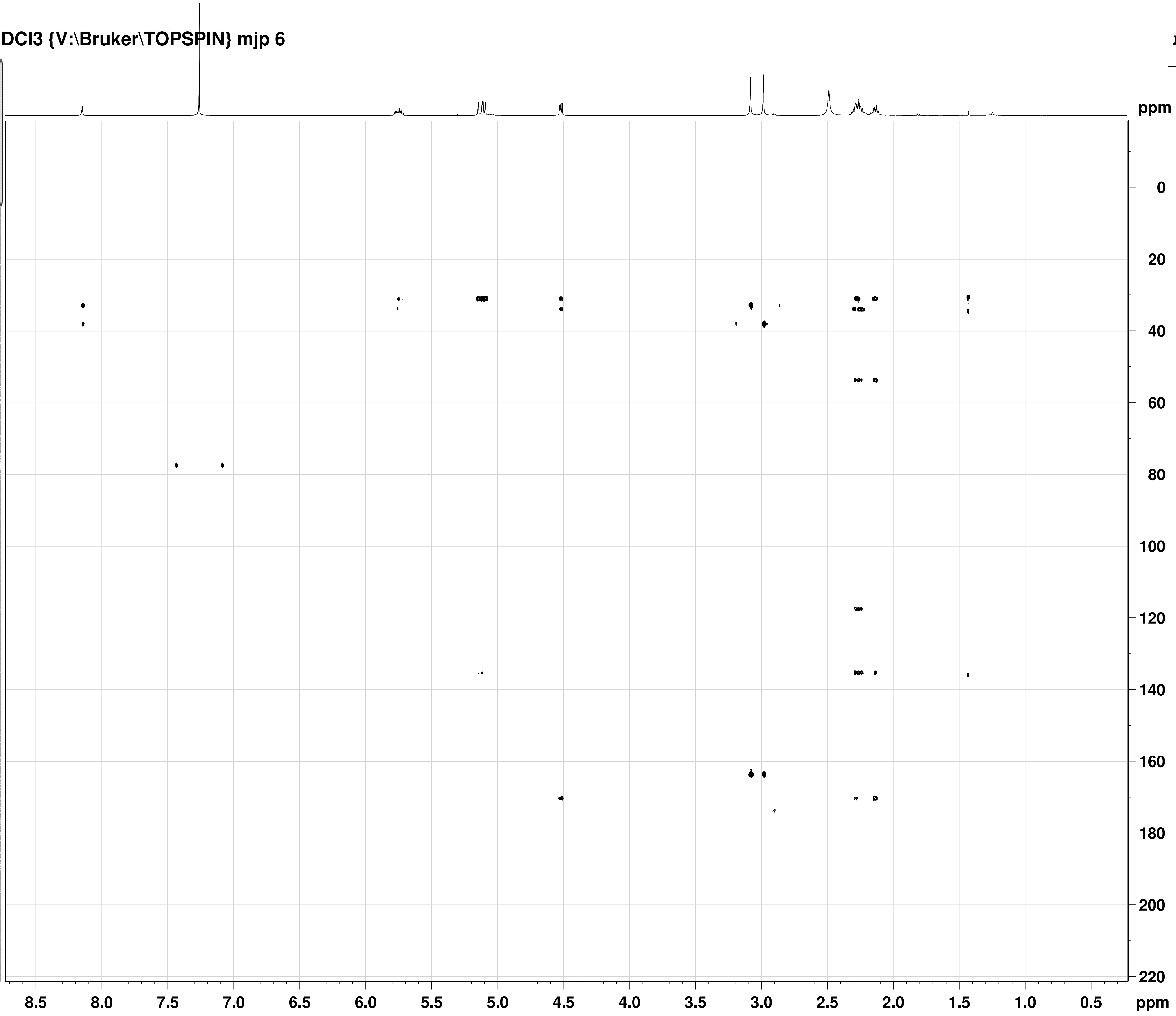


```
NAME          JC-332-1
EXPNO               14
PROCNO               1
Date_         20120228
Time             19.52
INSTRUM          AV600
PROBHD   5 mm CPDCH 13C
PULPROG    hmbcetgpl3nd
TD                4096
SOLVENT          CDCl3
NS                   2
DS                  16
SWH           5102.041 Hz
FIDRES        1.245615 Hz
AQ           0.4014580 sec
RG                2050
DW              98.000 usec
DE                6.50 usec
TE               298.0 K
CNST6      120.0000000
CNST7      160.0000000
CNST13      10.0000000
CNST30       0.5981152
D0          0.00000300 sec
D1          0.77638662 sec
D6          0.05000000 sec
D16         0.00020000 sec
IN0         0.00001380 sec

======== CHANNEL f1 ========
NUC1                1H
P1               11.40 usec
P2               22.80 usec
PL1               1.00 dB
PL1W       13.76731014 W
SFO1       600.1326990 MHz

======== CHANNEL f2 ========
NUC2               13C
P3                9.80 usec
P24            2000.00 usec
PL2               5.00 dB
PL2W       26.76886177 W
SFO2       150.9178993 MHz
SP7              13.33 dB
SPNAM7     Crp60comp.4
SPOAL7           0.500
SPOFFS7           0.00 Hz

====== GRADIENT CHANNEL =====
GPNAM1        SINE.100
GPNAM3        SINE.100
GPNAM4        SINE.100
GPNAM5        SINE.100
GPNAM6        SINE.100
GPZ1             80.00 %
GPZ3             14.00 %
GPZ4             -8.00 %
GPZ5             -4.00 %
GPZ6             -2.00 %
P16            1000.00 usec
ND0                  2
TD                 256
SFO1          150.9179 MHz
FIDRES      141.485535 Hz
SW             240.000 ppm
FnMODE    Echo-Antiecho
SI                2048
SF         600.1300105 MHz
WDW               SINE
SSB                  2
LB                0.00 Hz
GB                   0
PC                1.40
SI                1024
MC2        echo-antiecho
SF         150.9027756 MHz
WDW               SINE
SSB                  2
LB                0.00 Hz
GB                   0
```

**JC/365/1**

**PROTON.ucl CDCl3 {C:\Bruker\TOPSPIN} mjp 52**

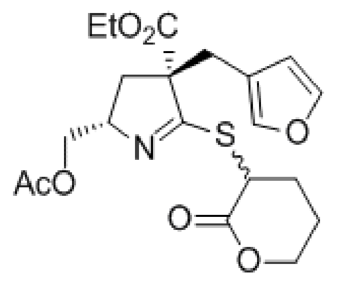

**19**

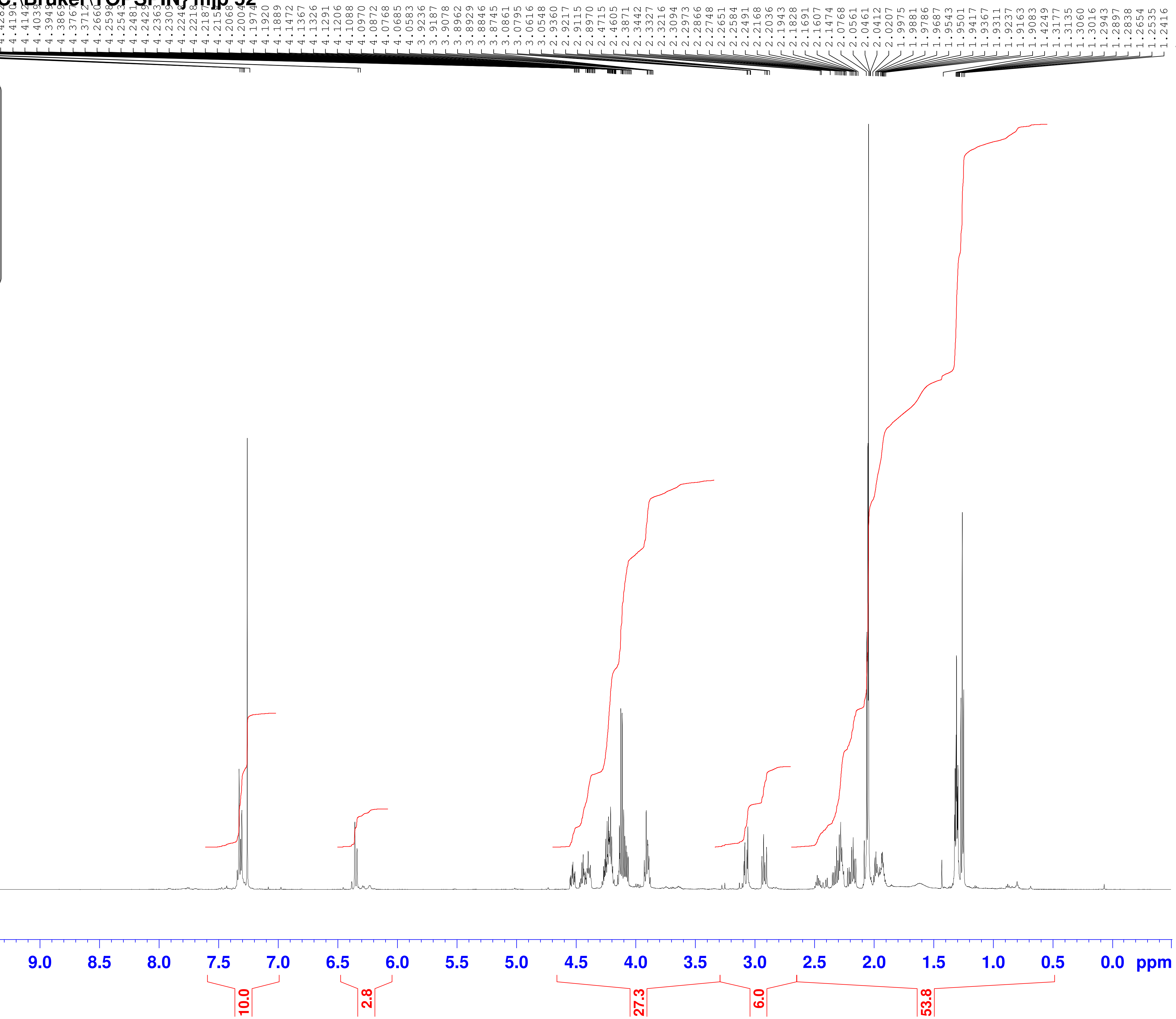


| | |
|---|---|
| NAME | Jul28-2010 |
| EXPNO | 100 |
| PROCNO | 1 |
| Date_ | 20100729 |
| Time | 1.37 |
| INSTRUM | AV600 |
| PROBHD | 5 mm CPDCH 13C |
| PULPROG | zg30 |
| TD | 98682 |
| SOLVENT | CDCl3 |
| NS | 8 |
| DS | 0 |
| SWH | 12335.526 Hz |
| FIDRES | 0.125003 Hz |
| AQ | 3.9999604 sec |
| RG | 57 |
| DW | 40.533 use |
| DE | 10.48 use |
| TE | 298.0 K |
| D1 | 1.00000000 sec |
| TD0 | 1 |
| ======== CHANNEL f1 ======== | |
| NUC1 | 1H |
| P1 | 11.40 use |
| PL1 | 1.00 dB |
| PL1W | 13.76731014 W |
| SFO1 | 600.1337061 MHz |
| SI | 32768 |
| SF | 600.1300116 MHz |
| WDW | EM |
| SSB | 0 |
| LB | 0.30 Hz |
| GB | 0 |
| PC | 1.40 |

**JC/365/1**
**C13CPD.ucl CDCl3 {C:\Bruker\TOPSPIN} mjp 52**

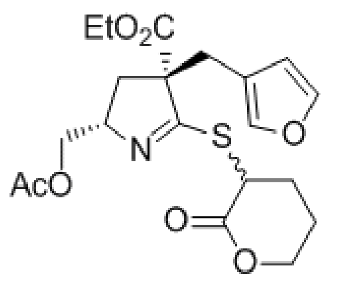


**19**

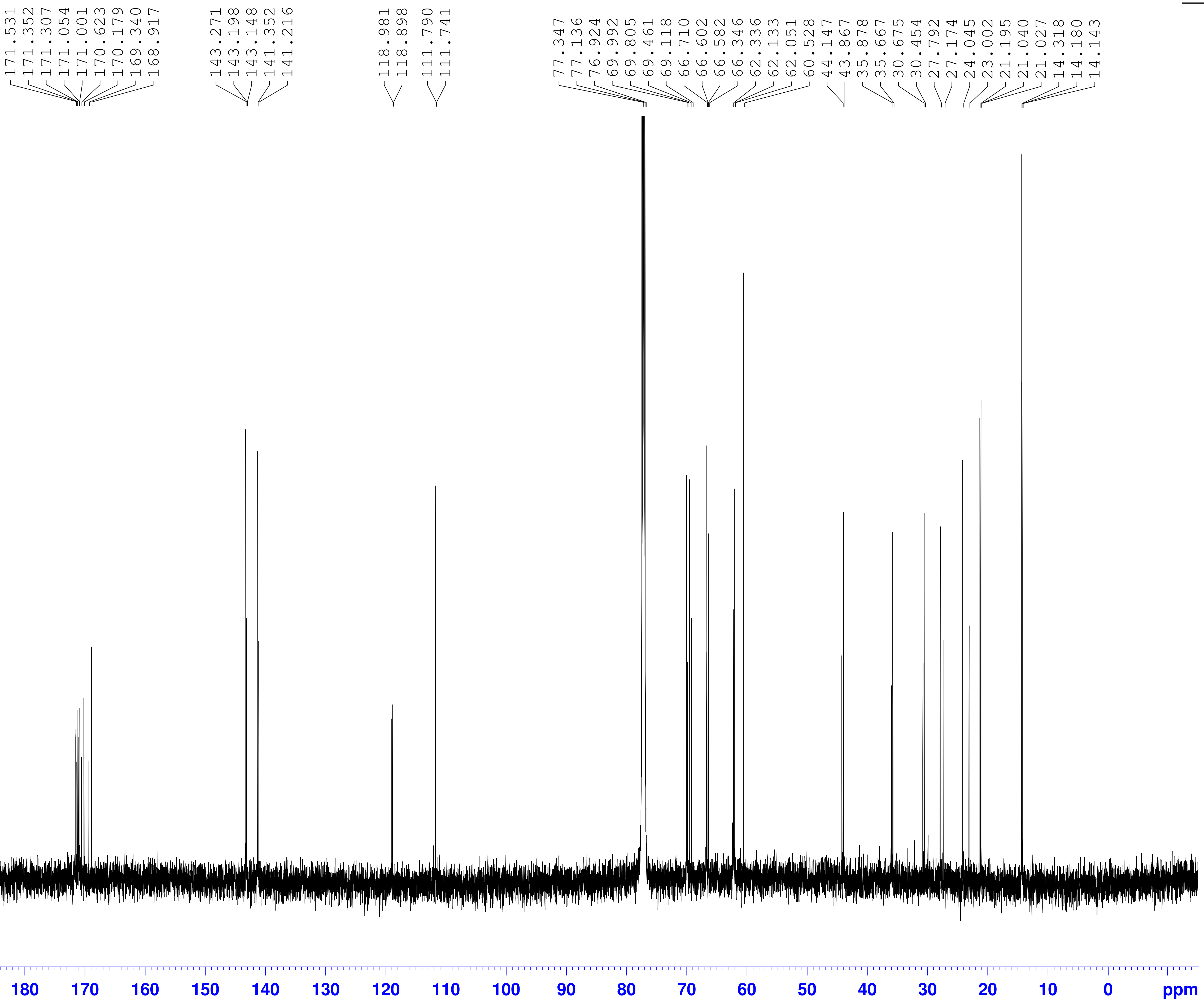


UCL

| | |
|---|---|
| NAME | Jul28-2010 |
| EXPNO | 101 |
| PROCNO | 1 |
| Date_ | 20100729 |
| Time | 1.43 |
| INSTRUM | AV600 |
| PROBHD | 5 mm CPDCH 13C |
| PULPROG | zgpg30 |
| TD | 65536 |
| SOLVENT | CDCl3 |
| NS | 128 |
| DS | 0 |
| SWH | 36057.691 H |
| FIDRES | 0.550197 H |
| AQ | 0.9088159 s |
| RG | 1030 |
| DW | 13.867 u |
| DE | 21.55 u |
| TE | 298.0 K |
| D1 | 2.00000000 s |
| D11 | 0.03000000 s |
| TD0 | 1 |

======== CHANNEL f1 =====

| | |
|---|---|
| NUC1 | 13C |
| P1 | 9.80 u |
| PL1 | 5.00 d |
| PL1W | 26.76886177 W |
| SFO1 | 150.9178981 M |

======== CHANNEL f2 =====

| | |
|---|---|
| CPDPRG2 | waltz16 |
| NUC2 | 1H |
| PCPD2 | 70.00 u |
| PL2 | 1.00 d |
| PL12 | 17.23 d |
| PL13 | 20.00 d |
| PL2W | 13.76731014 W |
| PL12W | 0.32798135 W |
| PL13W | 0.17332016 W |
| SFO2 | 600.1324005 M |
| SI | 32768 |
| SF | 150.9027930 M |
| WDW | EM |
| SSB | 0 |
| LB | 1.00 H |
| GB | 0 |
| PC | 1.40 |

JC/365/1
COSY.ucl CDCl3 {C:\Bruker\TOPSPIN} mjp 52

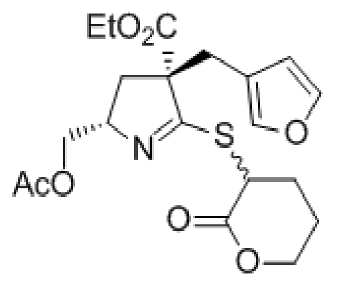


19

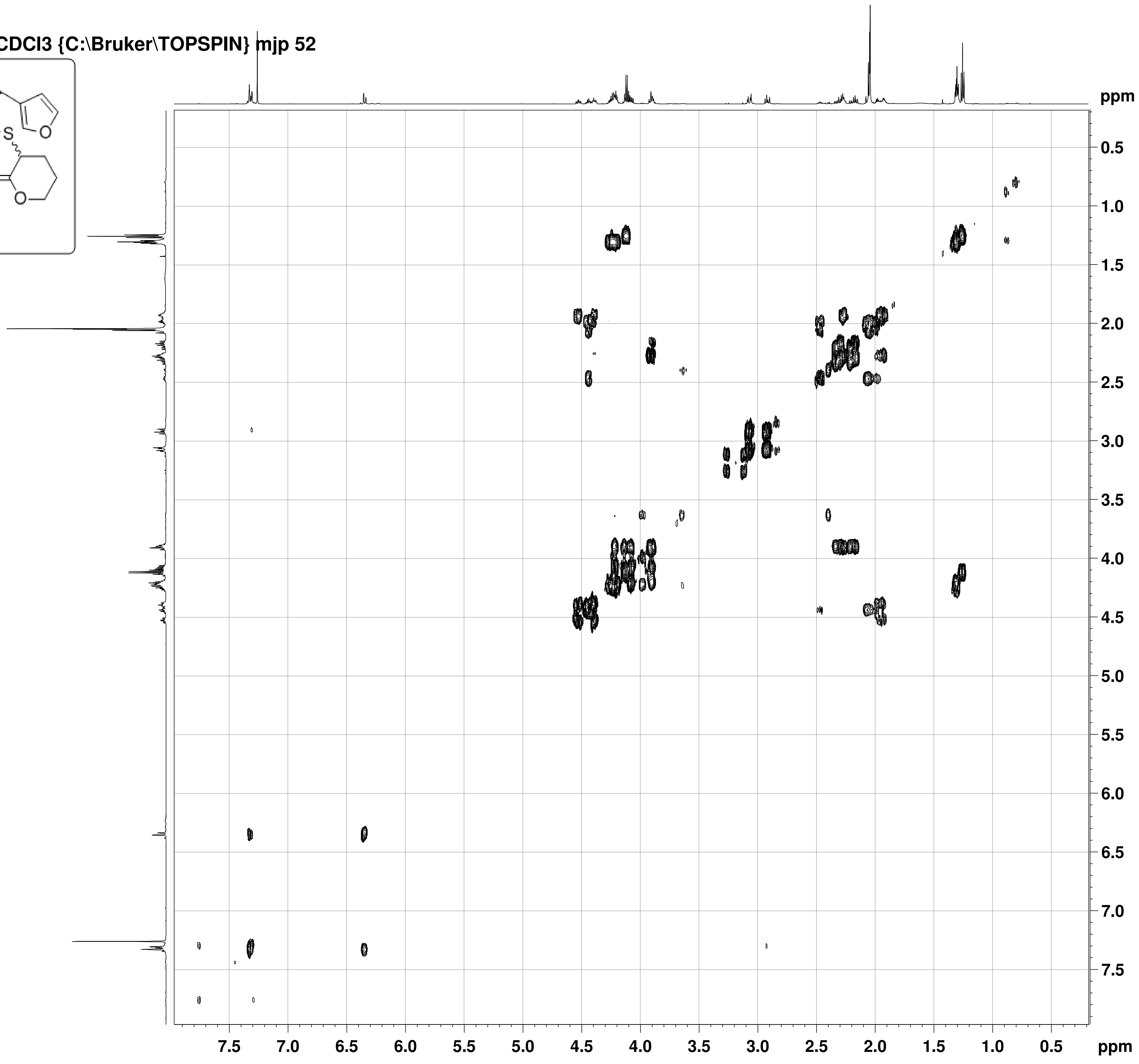


| | |
|---|---|
| NAME | Jul28-2010 |
| EXPNO | 103 |
| PROCNO | 1 |
| Date_ | 20100729 |
| Time | 1.52 |
| INSTRUM | AV600 |
| PROBHD | 5 mm CPDCH 13C |
| PULPROG | cosygpmfqf |
| TD | 2048 |
| SOLVENT | CDCl3 |
| NS | 1 |
| DS | 8 |
| SWH | 4672.897 Hz |
| FIDRES | 2.281688 Hz |
| AQ | 0.2191860 sec |
| RG | 2050 |
| DW | 107.000 usec |
| DE | 6.50 usec |
| TE | 298.0 K |
| D0 | 0.00000300 sec |
| D1 | 1.68092096 sec |
| D13 | 0.00000400 sec |
| D16 | 0.00020000 sec |
| IN0 | 0.00021400 sec |
| ======== CHANNEL f1 ======== | |
| NUC1 | 1H |
| P1 | 11.40 usec |
| PL1 | 1.00 dB |
| PL1W | 13.76731014 W |
| SFO1 | 600.1324569 MHz |
| ====== GRADIENT CHANNEL ==== | |
| GPNAM1 | SINE.100 |
| GPNAM2 | SINE.100 |
| GPNAM3 | SINE.100 |
| GPZ1 | 16.00 % |
| GPZ2 | 12.00 % |
| GPZ3 | 40.00 % |
| P16 | 1000.00 usec |
| ND0 | 1 |
| TD | 128 |
| SFO1 | 600.1325 MHz |
| FIDRES | 36.507008 Hz |
| SW | 7.786 ppm |
| FnMODE | QF |
| SI | 1024 |
| SF | 600.1300095 MHz |
| WDW | QSINE |
| SSB | 0 |
| LB | 0.00 Hz |
| GB | 0 |
| PC | 1.40 |
| SI | 1024 |
| MC2 | QF |
| SF | 600.1300095 MHz |
| WDW | QSINE |
| SSB | 0 |
| LB | 0.00 Hz |
| GB | 0 |

JC/365/1

HSQC.ucl CDCl3 {C:\Bruker\TOPSPIN} mjp 52

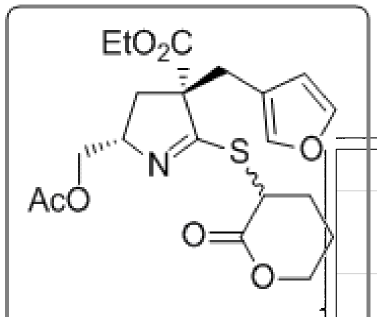


19

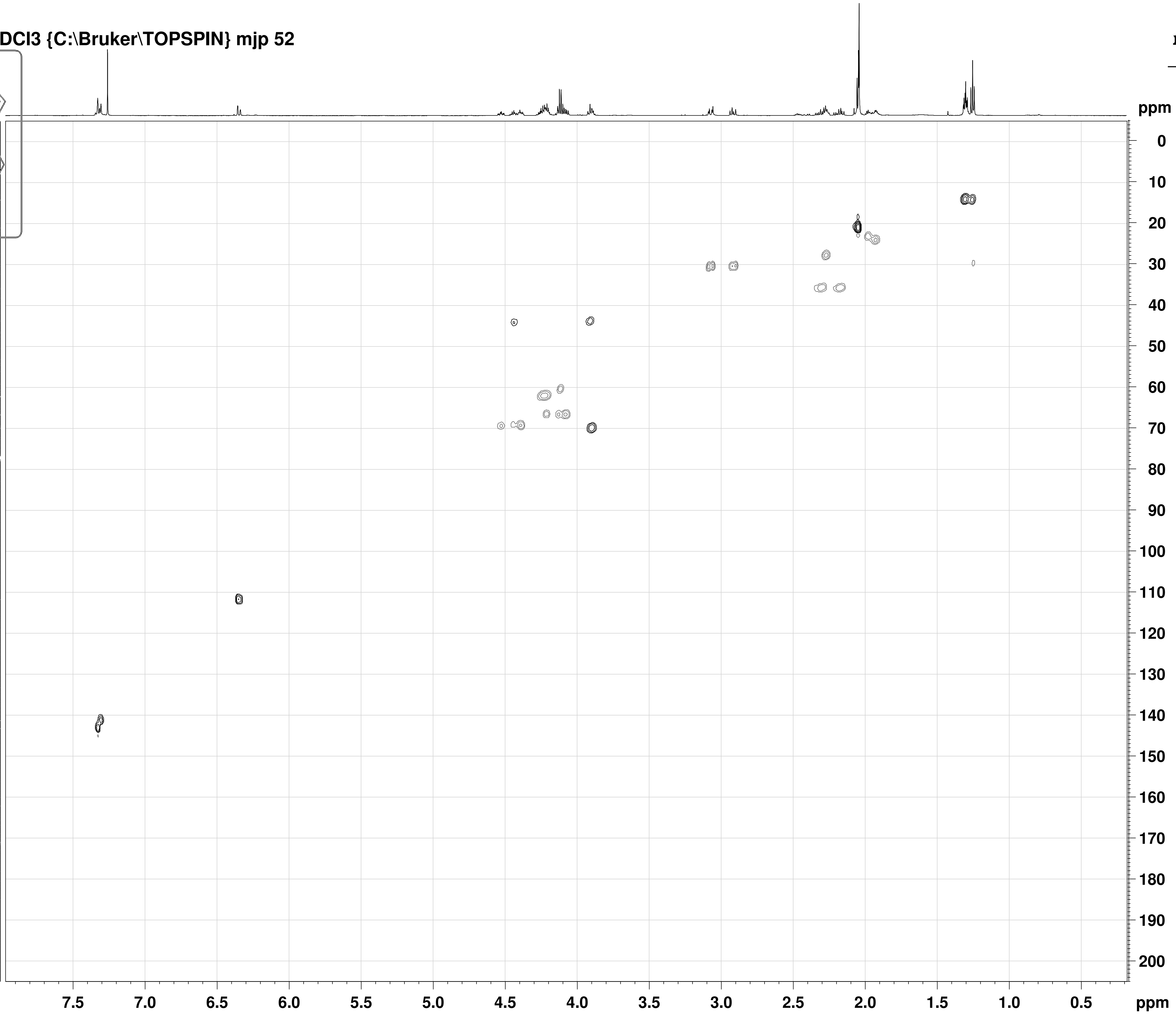


```
NAME            Jul28-2010
EXPNO                  102
PROCNO                   1
Date_             20100729
Time                  1.45
INSTRUM              AV600
PROBHD   5 mm CPDCH 13C
PULPROG  hsqcedetgpsisp2.4
TD                    1024
SOLVENT              CDCl3
NS                       2
DS                      32
SWH               4672.897 Hz
FIDRES            4.563376 Hz
AQ               0.1096180 sec
RG                    2050
DW                 107.000 usec
DE                    6.50 usec
TE                   298.0 K
CNST2          145.0000000
CNST17          -0.5000000
D0              0.00000300 sec
D1              1.47562897 sec
D2              0.00344828 sec
D4              0.00172414 sec
D11             0.03000000 sec
D16             0.00020000 sec
D21             0.00344828 sec
D24             0.00086207 sec
IN0             0.00001580 sec
L0                       0
L31                      1
TD0                      2

======== CHANNEL f1 ========
NUC1                    1H
P1                   11.40 usec
P2                   22.80 usec
P28                   0.00 usec
PL1                   1.00 dB
PL1W           13.76731014 W
SFO1         600.1324569 MHz

======== CHANNEL f2 ========
CPDPRG2   bi_p5m4sp_4sp.2
NUC2                   13C
P3                    9.80 usec
P14                 500.00 usec
P24                2000.00 usec
P31                1730.00 usec
P63                1500.00 usec
PL0                 120.00 dB
PL2                   5.00 dB
PL12                 20.74 dB
PL0W            0.00000000 W
PL2W           26.76886177 W
PL12W           0.71388775 W
SFO2         150.9178993 MHz
SP3                  13.33 dB
SP7                  13.33 dB
SP14                 14.82 dB
SP18                 18.73 dB
SP31                 20.84 dB
SPNAM3   Crp60,0.5,20.1
SPNAM7      Crp60comp.4
SPNAM14  Crp32,1.5,20.2
SPNAM18   Crp60_xfilt.2
SPNAM31  Crp32,1.5,20.2
SPOAL3               0.500
SPOAL7               0.500
SPOAL14              0.500
SPOAL18              0.500
SPOAL31              0.500
SPOFFS3               0.00 Hz
SPOFFS7               0.00 Hz
SPOFFS14              0.00 Hz
SPOFFS18              0.00 Hz
SPOFFS31              0.00 Hz

====== GRADIENT CHANNEL =====
GPNAM1           SINE.100
GPNAM2           SINE.100
GPNAM3           SINE.100
GPNAM4           SINE.100
GPZ1                 80.00 %
GPZ2                 20.10 %
GPZ3                 11.00 %
GPZ4                 -5.00 %
P16                1000.00 usec
P19                 600.00 usec
ND0                      2
TD                     128
SFO1          150.9179 MHz
FIDRES          247.599686 Hz
SW                 210.000 ppm
FnMODE        Echo-Antiecho
SI                    1024
SF           600.1300095 MHz
WDW                  QSINE
SSB                      2
LB                    0.00 Hz
GB                       0
PC                    1.40
SI                    1024
MC2            echo-antiecho
SF           150.9027781 MHz
WDW                  QSINE
SSB                      2
LB                    0.00 Hz
GB                       0
```

JC–366–2

PROTON.ucl CDCl3 {V:\Bruker\TOPSPIN} mjp 30

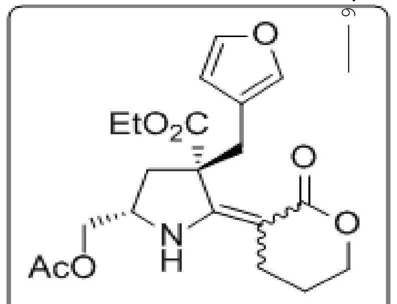


20

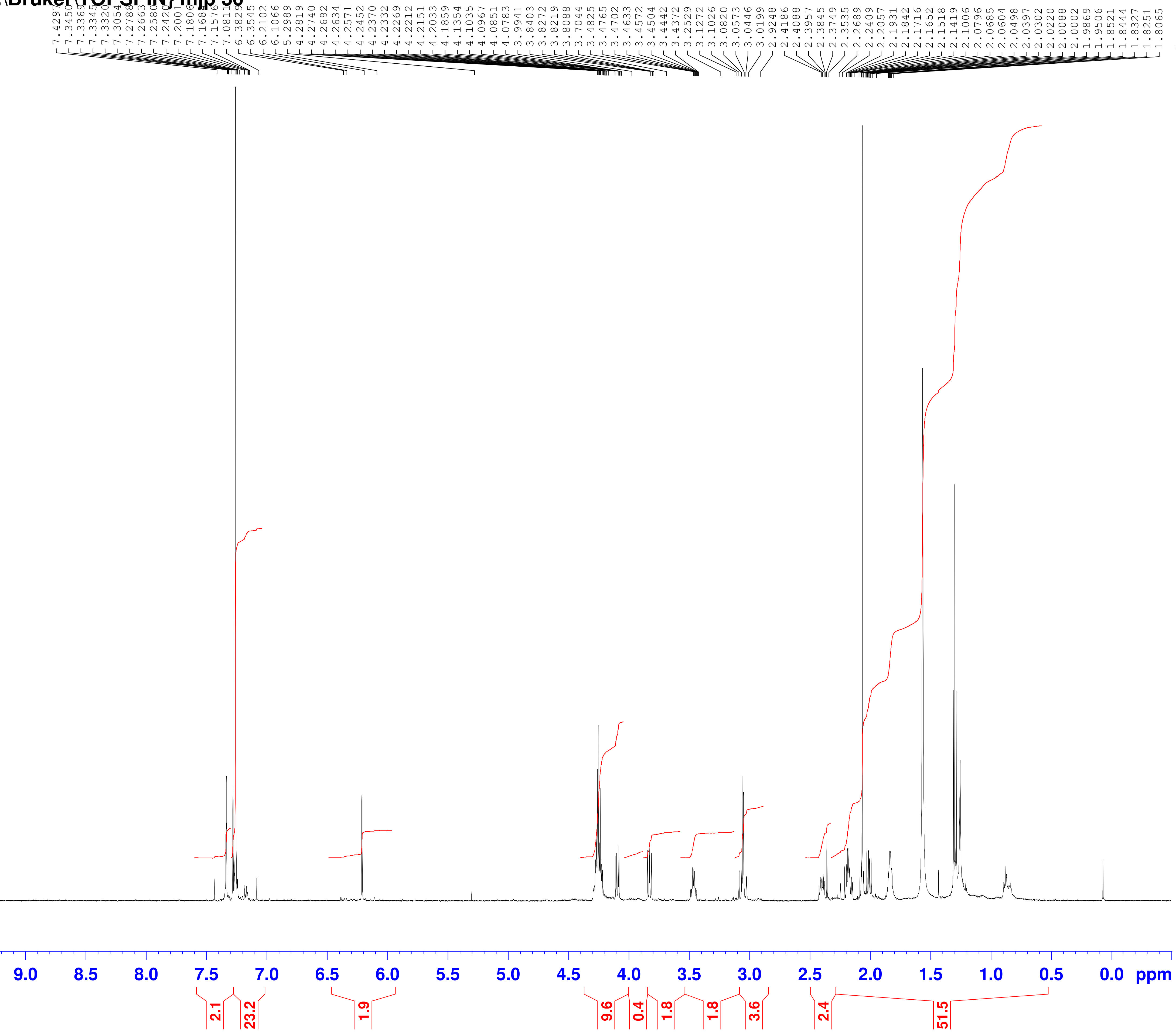


| | |
|---|---|
| NAME | JC-366-2 |
| EXPNO | 10 |
| PROCNO | 1 |
| Date_ | 20120327 |
| Time | 13.57 |
| INSTRUM | AV600 |
| PROBHD | 5 mm CPDCH 13C |
| PULPROG | zg30 |
| TD | 98682 |
| SOLVENT | CDCl3 |
| NS | 8 |
| DS | 0 |
| SWH | 12335.526 Hz |
| FIDRES | 0.125003 Hz |
| AQ | 3.9999604 sec |
| RG | 32 |
| DW | 40.533 use |
| DE | 10.48 use |
| TE | 298.0 K |
| D1 | 1.00000000 sec |
| TD0 | 1 |

======== CHANNEL f1 ========

| | |
|---|---|
| NUC1 | 1H |
| P1 | 11.40 use |
| PL1 | 1.00 dB |
| PL1W | 13.76731014 W |
| SFO1 | 600.1337061 MHz |
| SI | 32768 |
| SF | 600.1300116 MHz |
| WDW | EM |
| SSB | 0 |
| LB | 0.30 Hz |
| GB | 0 |
| PC | 1.40 |

V:BrukerTOPSPIN/mjp/JC–366–2/10/1
Integral regions #5 to #8

# JC–366–2
PROTON.ucl CDCl3 {V:\Bruker\TOPSPIN} mjp 30

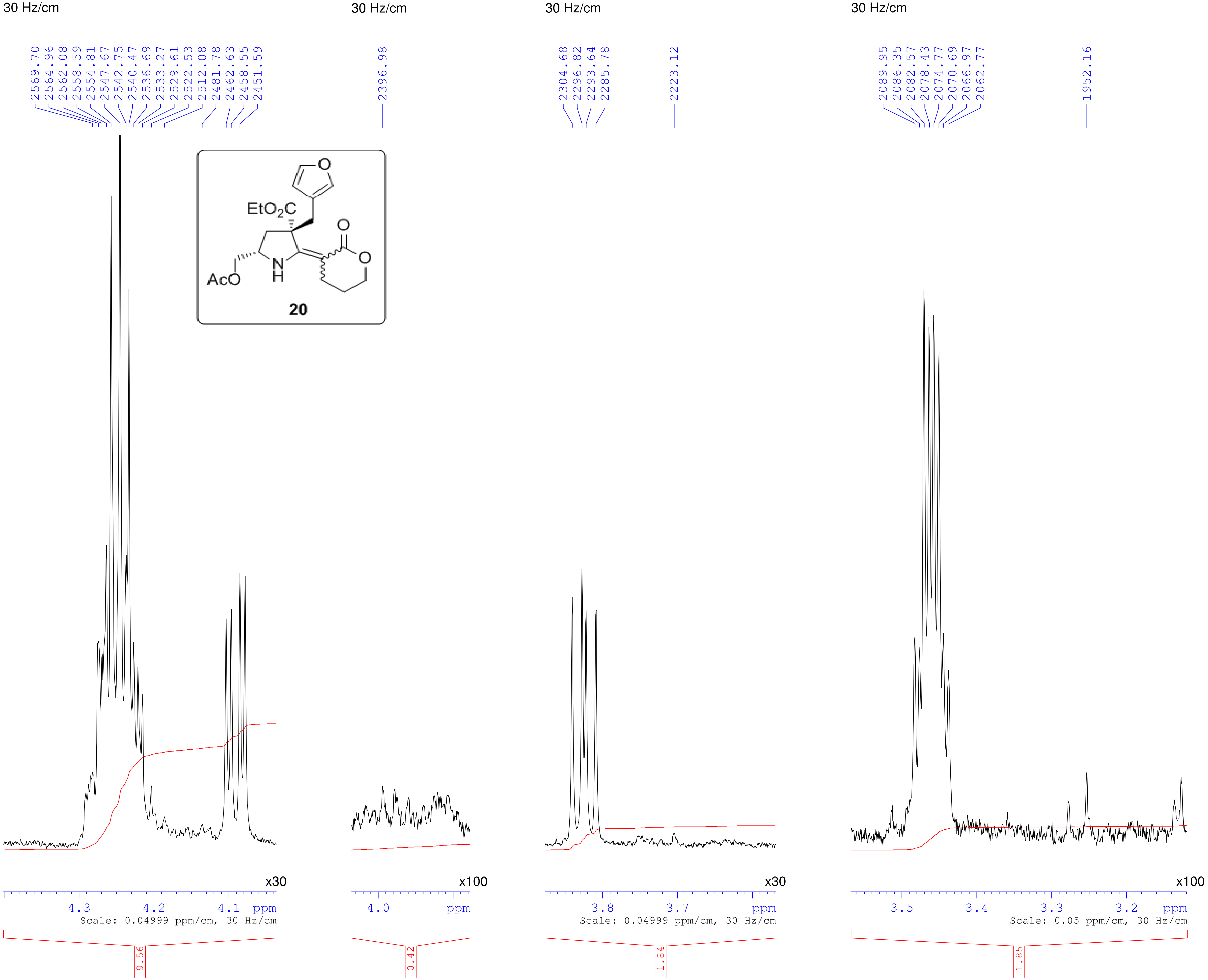

**JC−366−2**
**C13CPD.ucl CDCl3 {V:\Bruker\TOPSPIN} mjp 30**

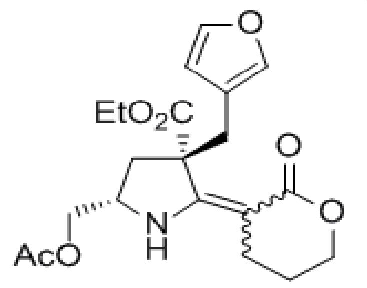


**20**

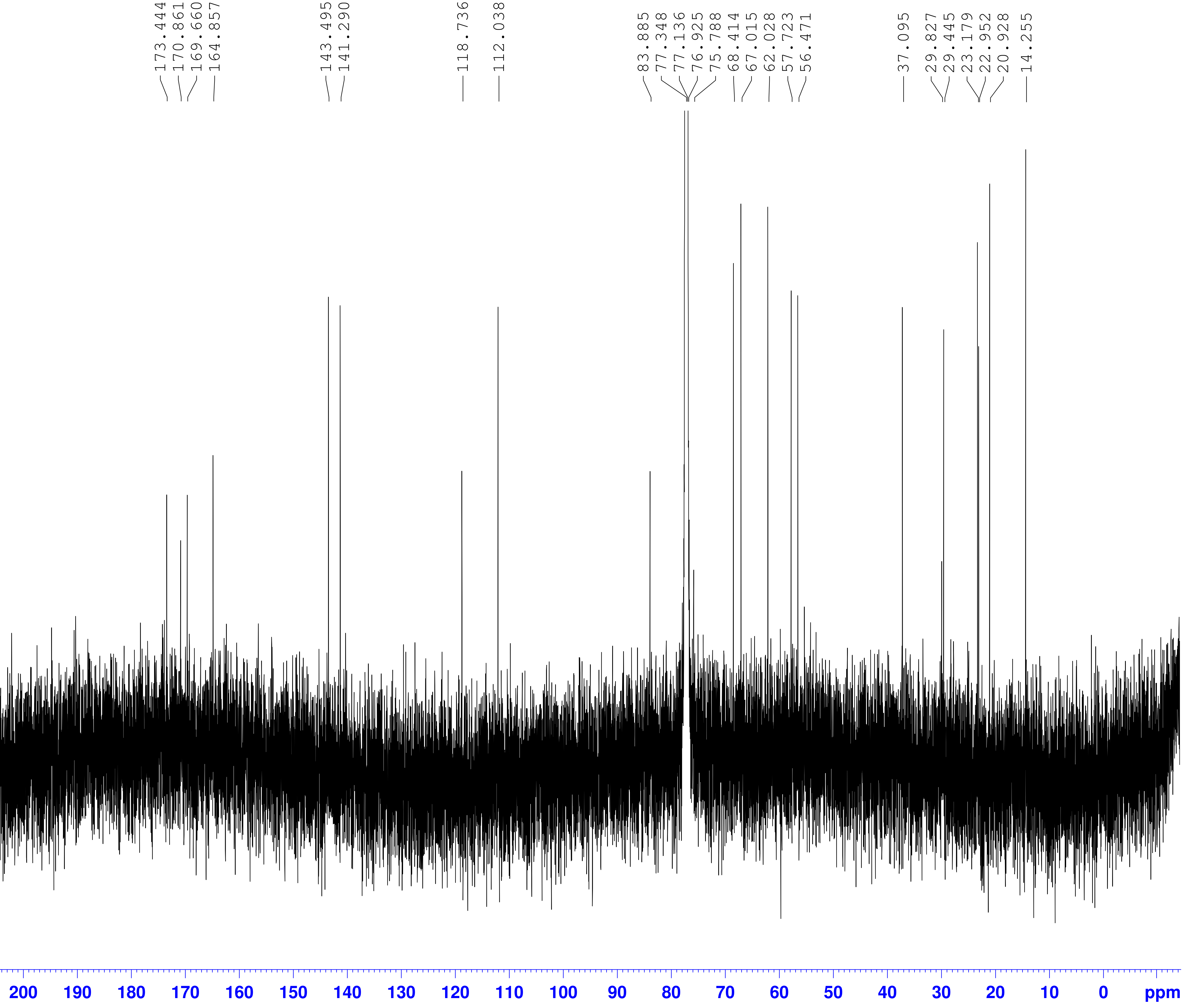


UCL

| | |
|---|---|
| NAME | JC-366-2 |
| EXPNO | 12 |
| PROCNO | 1 |
| Date_ | 20120327 |
| Time | 14.08 |
| INSTRUM | AV600 |
| PROBHD | 5 mm CPDCH 13C |
| PULPROG | zgpg30 |
| TD | 70308 |
| SOLVENT | CDCl3 |
| NS | 128 |
| DS | 0 |
| SWH | 39062.500 H |
| FIDRES | 0.555591 H |
| AQ | 0.8999924 s |
| RG | 1030 |
| DW | 12.800 u |
| DE | 21.12 u |
| TE | 298.0 K |
| D1 | 2.00000000 s |
| D11 | 0.03000000 s |
| TD0 | 1 |
| ======== CHANNEL f1 ===== | |
| NUC1 | 13C |
| P1 | 9.80 u |
| PL1 | 5.00 d |
| PL1W | 26.76886177 W |
| SFO1 | 150.9201628 M |
| ======== CHANNEL f2 ===== | |
| CPDPRG2 | waltz16 |
| NUC2 | 1H |
| PCPD2 | 70.00 u |
| PL2 | 1.00 d |
| PL12 | 17.23 d |
| PL13 | 20.00 d |
| PL2W | 13.76731014 W |
| PL12W | 0.32798135 W |
| PL13W | 0.17332016 W |
| SFO2 | 600.1324005 M |
| SI | 65536 |
| SF | 150.9027930 M |
| WDW | EM |
| SSB | 0 |
| LB | 1.00 H |
| GB | 0 |
| PC | 1.40 |

JC–366–2
COSY.ucl CDCl3 {V:\Bruker\TOPSPIN} mjp 30

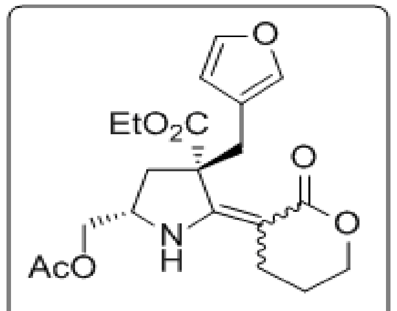

20

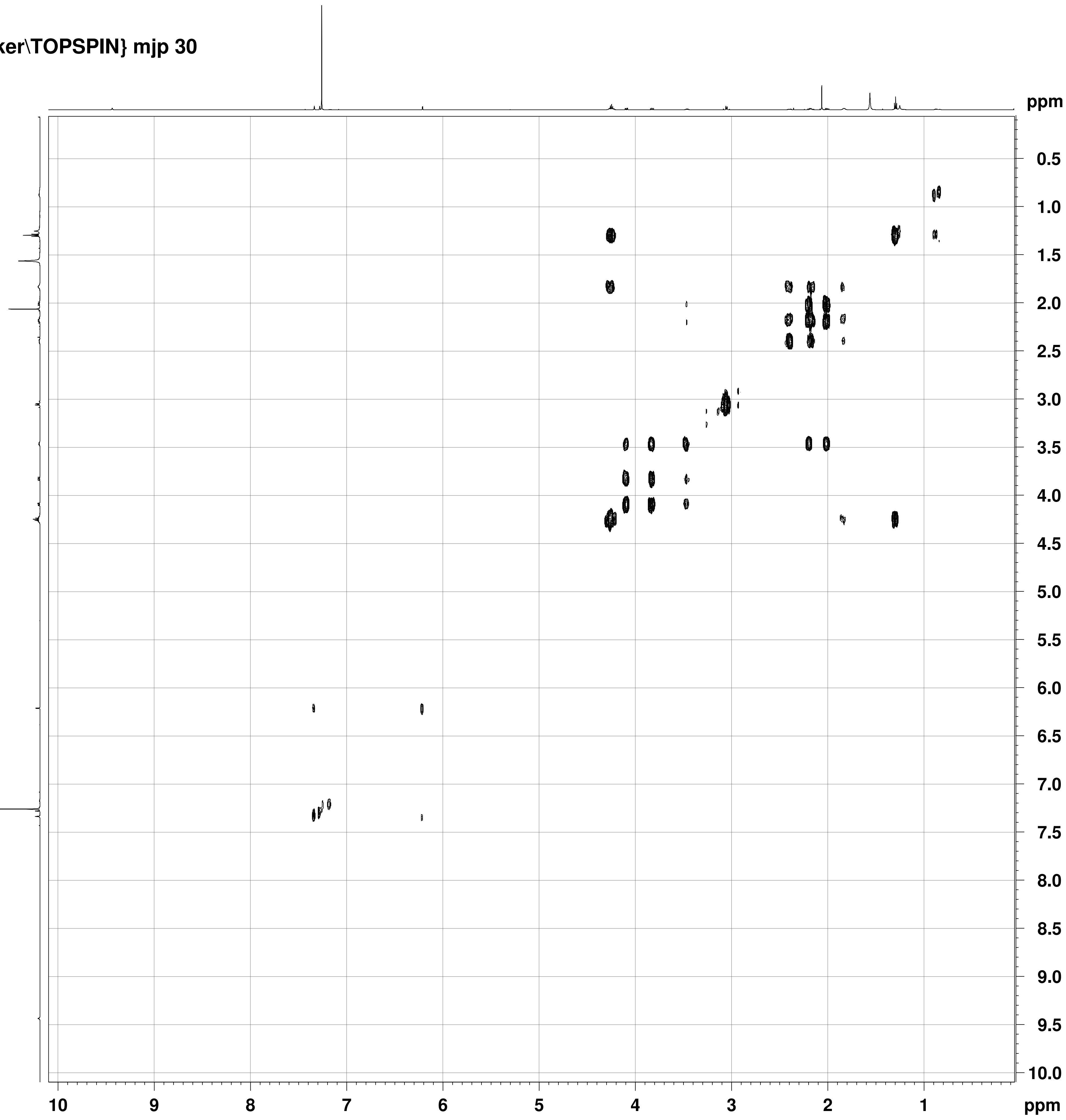


UCL

```
NAME              JC-366-2
EXPNO                   11
PROCNO                   1
Date_             20120327
Time                 13.58
INSTRUM              AV600
PROBHD   5 mm CPDCH 13C
PULPROG         cosygpmfqf
TD                    2048
SOLVENT              CDCl3
NS                       1
DS                       8
SWH           6024.096 Hz
FIDRES        2.941453 Hz
AQ           0.1700340 sec
RG                    2050
DW            83.000 usec
DE              6.50 usec
TE               298.0 K
D0          0.00000300 sec
D1          1.73007298 sec
D13         0.00000400 sec
D16         0.00020000 sec
IN0         0.00016600 sec

======== CHANNEL f1 ========
NUC1                    1H
P1              11.40 usec
PL1                1.00 dB
PL1W         13.76731014 W
SFO1        600.1330591 MHz

====== GRADIENT CHANNEL ====
GPNAM1            SINE.100
GPNAM2            SINE.100
GPNAM3            SINE.100
GPZ1               16.00 %
GPZ2               12.00 %
GPZ3               40.00 %
P16            1000.00 usec
ND0                      1
TD                     128
SFO1          600.1331 MHz
FIDRES       47.063251 Hz
SW              10.038 ppm
FnMODE                  QF
SI                    1024
SF         600.1300088 MHz
WDW                  QSINE
SSB                      0
LB                 0.00 Hz
GB                       0
PC                    1.40
SI                    1024
MC2                     QF
SF         600.1300088 MHz
WDW                  QSINE
SSB                      0
LB                 0.00 Hz
GB                       0
```

**JC–366–2**
**C13DEPT135.ucl CDCl3 {V:\Bruker\TOPSPIN} mjp 30**

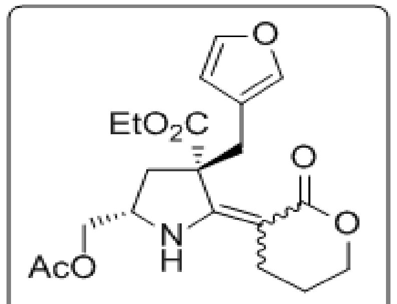


20

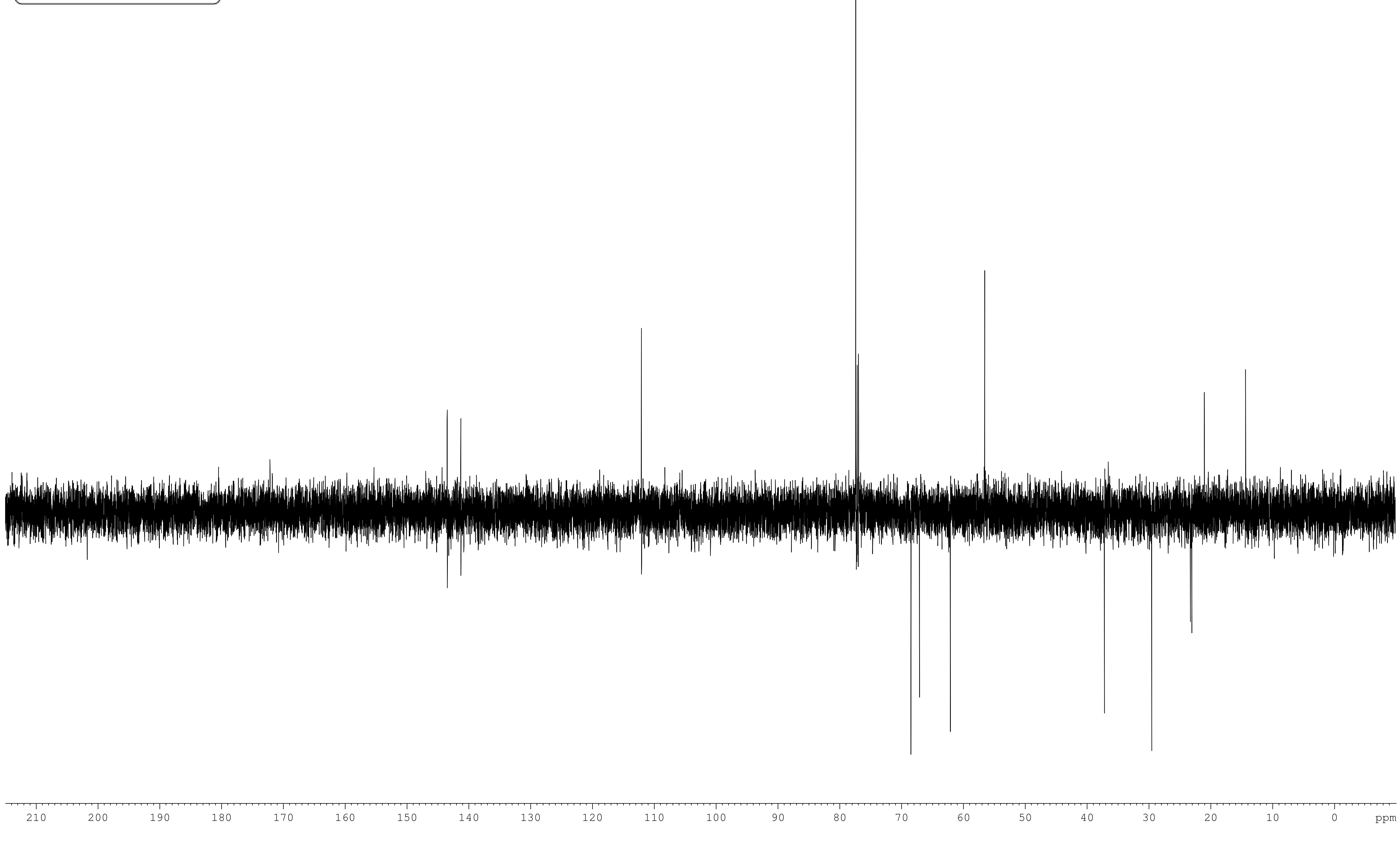


| | |
|---|---|
| NAME | JC-366-2 |
| EXPNO | 15 |
| PROCNO | 1 |
| Date_ | 20120327 |
| Time | 14.31 |
| INSTRUM | AV600 |
| PROBHD | 5 mm CPDCH 13C |
| PULPROG | dept135 |
| TD | 70308 |
| SOLVENT | CDCl3 |
| NS | 64 |
| DS | 4 |
| SWH | 39062.500 Hz |
| FIDRES | 0.555591 Hz |
| AQ | 0.8999924 sec |
| RG | 256 |
| DW | 12.800 usec |
| DE | 6.50 usec |
| TE | 298.0 K |
| CNST2 | 145.0000000 |
| D1 | 2.00000000 sec |
| D2 | 0.00344828 sec |
| D12 | 0.00002000 sec |
| TD0 | 1 |
| ======== CHANNEL f1 ======== | |
| NUC1 | 13C |
| P1 | 9.80 usec |
| P2 | 19.60 usec |
| PL1 | 5.00 dB |
| PL1W | 26.76886177 W |
| SFO1 | 150.9201628 MHz |
| ======== CHANNEL f2 ======== | |
| CPDPRG2 | waltz16 |
| NUC2 | 1H |
| P3 | 10.80 usec |
| P4 | 21.60 usec |
| PCPD2 | 70.00 usec |
| PL2 | 1.00 dB |
| PL12 | 17.23 dB |
| PL2W | 13.76731014 W |
| PL12W | 0.32798135 W |
| SFO2 | 600.1324005 MHz |
| SI | 65536 |
| SF | 150.9027930 MHz |
| WDW | EM |
| SSB | 0 |
| LB | 1.00 Hz |
| GB | 0 |
| PC | 1.40 |

**JC–366–2**
**HSQC.ucl CDCl3 {V:\Bruker\TOPSPIN} mjp 30**

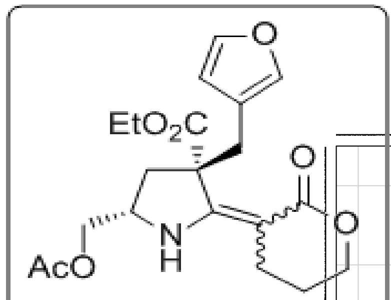

**20**

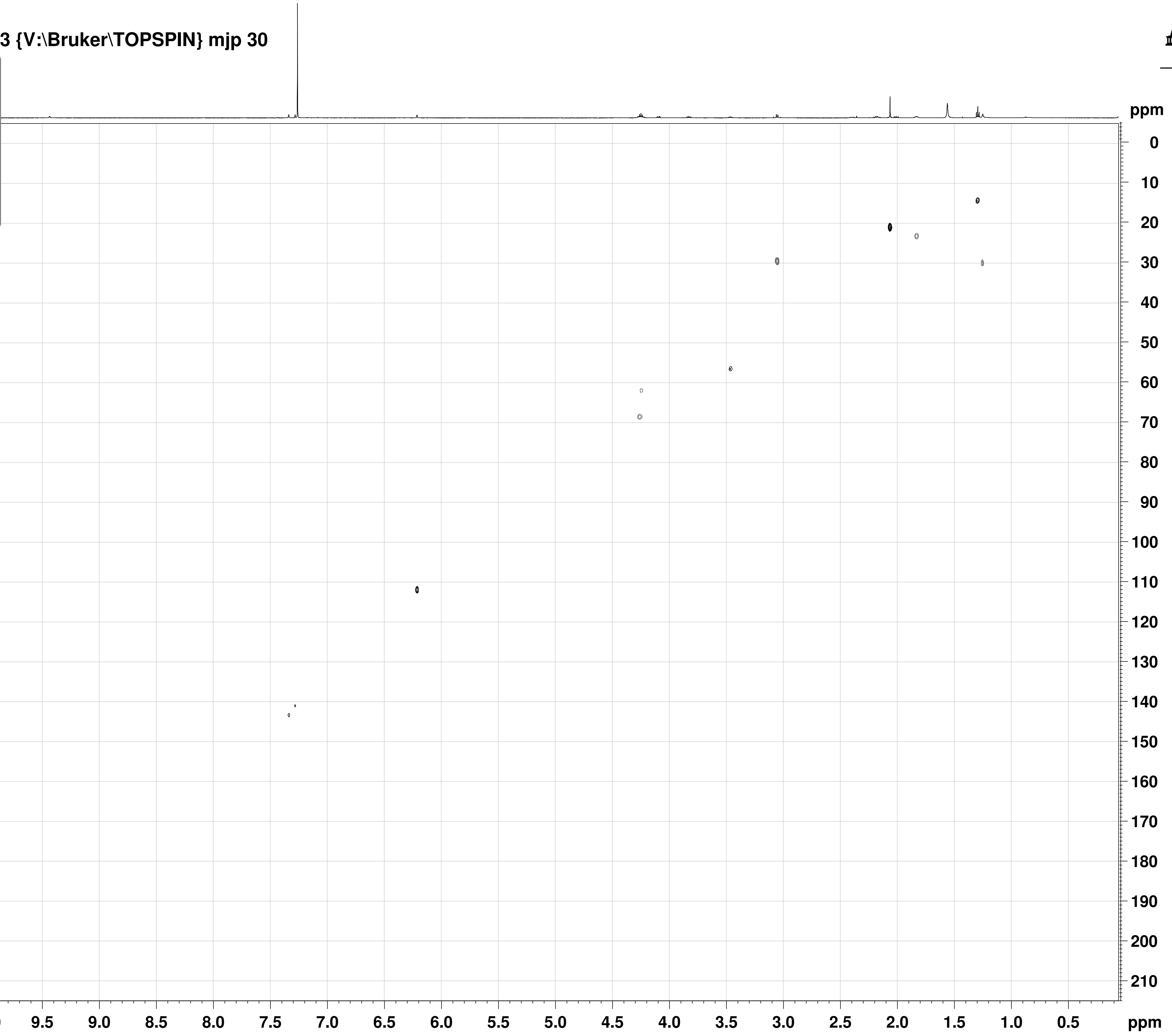


| | | |
|---|---|---|
| NAME | JC-366-2 | |
| EXPNO | 13 | |
| PROCNO | 1 | |
| Date_ | 20120327 | |
| Time | 14.10 | |
| INSTRUM | AV600 | |
| PROBHD | 5 mm CPDCH 13C | |
| PULPROG | hsqcedetgpsisp2.4 | |
| TD | 1024 | |
| SOLVENT | CDCl3 | |
| NS | 2 | |
| DS | 32 | |
| SWH | 6024.096 | Hz |
| FIDRES | 5.882906 | Hz |
| AQ | 0.0850420 | sec |
| RG | 2050 | |
| DW | 83.000 | usec |
| DE | 6.50 | usec |
| TE | 298.0 | K |
| CNST2 | 145.0000000 | |
| CNST17 | -0.5000000 | |
| D0 | 0.00000300 | sec |
| D1 | 1.50020504 | sec |
| D2 | 0.00344828 | sec |
| D4 | 0.00172414 | sec |
| D11 | 0.03000000 | sec |
| D16 | 0.00020000 | sec |
| D21 | 0.00344828 | sec |
| D24 | 0.00086207 | sec |
| IN0 | 0.00001505 | sec |
| L0 | 0 | |
| L31 | 1 | |
| TD0 | 2 | |
| ======== CHANNEL f1 ======== | | |
| NUC1 | 1H | |
| P1 | 11.40 | usec |
| P2 | 22.80 | usec |
| P28 | 0.00 | usec |
| PL1 | 1.00 | dB |
| PL1W | 13.76731014 | W |
| SFO1 | 600.1330591 | MHz |
| ======== CHANNEL f2 ======== | | |
| CPDPRG2 | bi_p5m4sp_4sp.2 | |
| NUC2 | 13C | |
| P3 | 9.80 | usec |
| P14 | 500.00 | usec |
| P24 | 2000.00 | usec |
| P31 | 1730.00 | usec |
| P63 | 1500.00 | usec |
| PL0 | 120.00 | dB |
| PL2 | 5.00 | dB |
| PL12 | 20.74 | dB |
| PL0W | 0.00000000 | W |
| PL2W | 26.76886177 | W |
| PL12W | 0.71388775 | W |
| SFO2 | 150.9186538 | MHz |
| SP3 | 13.33 | dB |
| SP7 | 13.33 | dB |
| SP14 | 14.82 | dB |
| SP18 | 18.73 | dB |
| SP31 | 20.84 | dB |
| SPNAM3 | Crp60,0.5,20.1 | |
| SPNAM7 | Crp60comp.4 | |
| SPNAM14 | Crp32,1.5,20.2 | |
| SPNAM18 | Crp60_xfilt.2 | |
| SPNAM31 | Crp32,1.5,20.2 | |
| SPOAL3 | 0.500 | |
| SPOAL7 | 0.500 | |
| SPOAL14 | 0.500 | |
| SPOAL18 | 0.500 | |
| SPOAL31 | 0.500 | |
| SPOFFS3 | 0.00 | Hz |
| SPOFFS7 | 0.00 | Hz |
| SPOFFS14 | 0.00 | Hz |
| SPOFFS18 | 0.00 | Hz |
| SPOFFS31 | 0.00 | Hz |
| ====== GRADIENT CHANNEL ===== | | |
| GPNAM1 | SINE.100 | |
| GPNAM2 | SINE.100 | |
| GPNAM3 | SINE.100 | |
| GPNAM4 | SINE.100 | |
| GPZ1 | 80.00 | % |
| GPZ2 | 20.10 | % |
| GPZ3 | 11.00 | % |
| GPZ4 | -5.00 | % |
| P16 | 1000.00 | usec |
| P19 | 600.00 | usec |
| ND0 | 2 | |
| TD | 128 | |
| SFO1 | 150.9187 | MHz |
| FIDRES | 259.391449 | Hz |
| SW | 220.000 | ppm |
| FnMODE | Echo-Antiecho | |
| SI | 1024 | |
| SF | 600.1300088 | MHz |
| WDW | QSINE | |
| SSB | 2 | |
| LB | 0.00 | Hz |
| GB | 0 | |
| PC | 1.40 | |
| SI | 1024 | |
| MC2 | echo-antiecho | |
| SF | 150.9027771 | MHz |
| WDW | QSINE | |
| SSB | 2 | |
| LB | 0.00 | Hz |
| GB | 0 | |

JC–366–2
HMBC.ucl CDCl3 {V:\Bruker\TOPSPIN} mjp 30

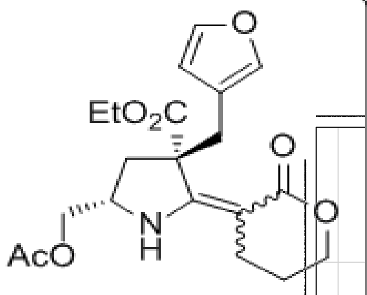


20

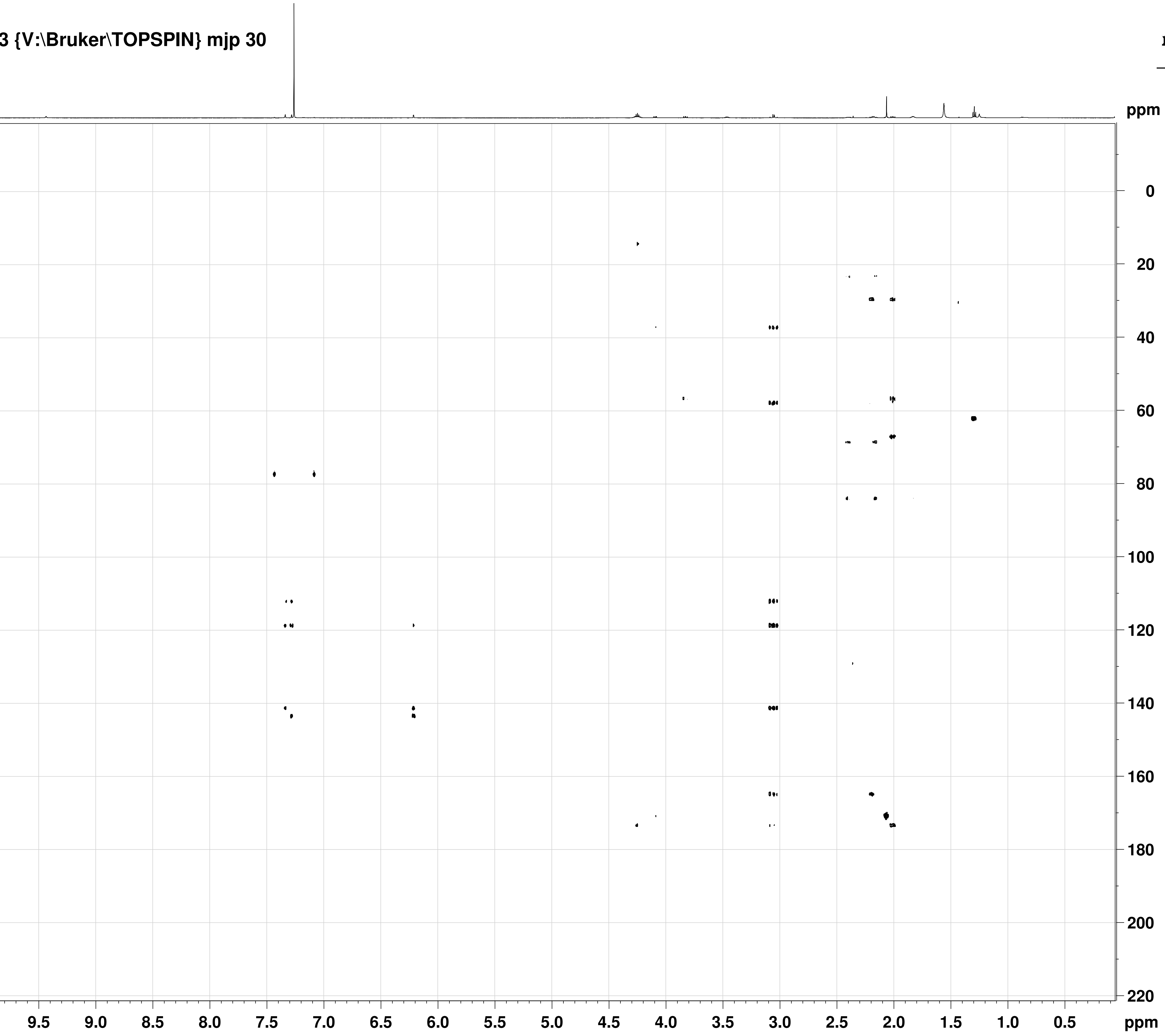


UCL

NAME JC-366-2
EXPNO 14
PROCNO 1
Date_ 20120327
Time 14.17
INSTRUM AV600
PROBHD 5 mm CPDCH 13C
PULPROG hmbcetgpl3nd
TD 4096
SOLVENT CDCl3
NS 2
DS 16
SWH 6024.096 Hz
FIDRES 1.470727 Hz
AQ 0.3400180 sec
RG 2050
DW 83.000 usec
DE 6.50 usec
TE 298.0 K
CNST6 120.0000000
CNST7 160.0000000
CNST13 10.0000000
CNST30 0.5981154
D0 0.00000300 sec
D1 0.83782661 sec
D6 0.05000000 sec
D16 0.00020000 sec
IN0 0.00001380 sec

======== CHANNEL f1 ========
NUC1 1H
P1 11.40 usec
P2 22.80 usec
PL1 1.00 dB
PL1W 13.76731014 W
SFO1 600.1330591 MHz

======== CHANNEL f2 ========
NUC2 13C
P3 9.80 usec
P24 2000.00 usec
PL2 5.00 dB
PL2W 26.76886177 W
SFO2 150.9178993 MHz
SP7 13.33 dB
SPNAM7 Crp60comp.4
SPOAL7 0.500
SPOFFS7 0.00 Hz

====== GRADIENT CHANNEL =====
GPNAM1 SINE.100
GPNAM3 SINE.100
GPNAM4 SINE.100
GPNAM5 SINE.100
GPNAM6 SINE.100
GPZ1 80.00 %
GPZ3 14.00 %
GPZ4 -8.00 %
GPZ5 -4.00 %
GPZ6 -2.00 %
P16 1000.00 usec
ND0 2
TD 256
SFO1 150.9179 MHz
FIDRES 141.485535 Hz
SW 240.000 ppm
FnMODE Echo-Antiecho
SI 2048
SF 600.1300103 MHz
WDW SINE
SSB 2
LB 0.00 Hz
GB 0
PC 1.40
SI 1024
MC2 echo-antiecho
SF 150.9027756 MHz
WDW SINE
SSB 2
LB 0.00 Hz
GB 0

# JC–333–4

## PROTON.ucl CDCl3 {V:\Bruker\TOPSPIN} mjp 58

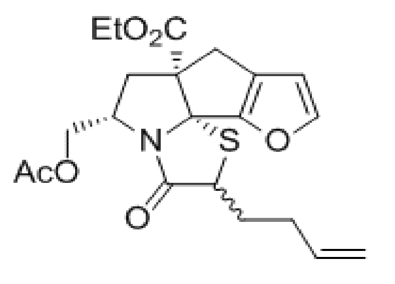


**21**

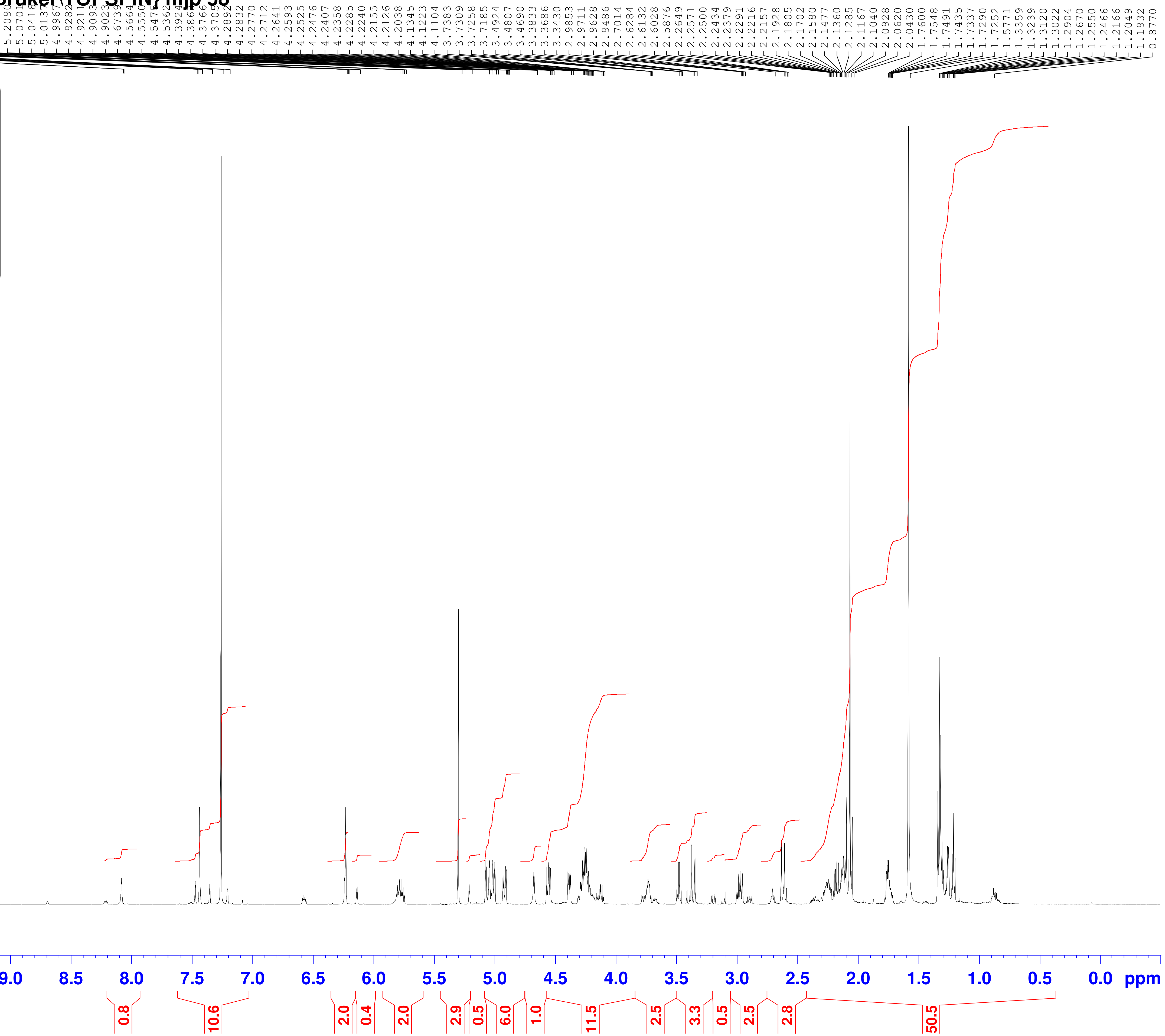


UCL

```
NAME              JC-333-4
EXPNO                   10
PROCNO                   1
Date_             20120515
Time                  2.23
INSTRUM              AV600
PROBHD   5 mm CPDCH 13C
PULPROG               zg30
TD                   98682
SOLVENT              CDCl3
NS                      32
DS                       0
SWH             12335.526 Hz
FIDRES           0.125003 Hz
AQ              3.9999604 sec
RG                    40.3
DW                  40.533 use
DE                   10.48 use
TE                   298.0 K
D1             1.00000000 sec
TD0                      1

======== CHANNEL f1 =======
NUC1                    1H
P1                   11.40 use
PL1                   1.00 dB
PL1W           13.76731014 W
SFO1           600.1337061 MHz
SI                   32768
SF             600.1300116 MHz
WDW                     EM
SSB                      0
LB                    0.30 Hz
GB                       0
PC                    1.40
```

V:BrukerTOPSPIN/mjp/JC−333−4/10/1
Integral regions #5 to #9

JC−333−4
PROTON.ucl CDCl3 {V:\Bruker\TOPSPIN} mjp 58

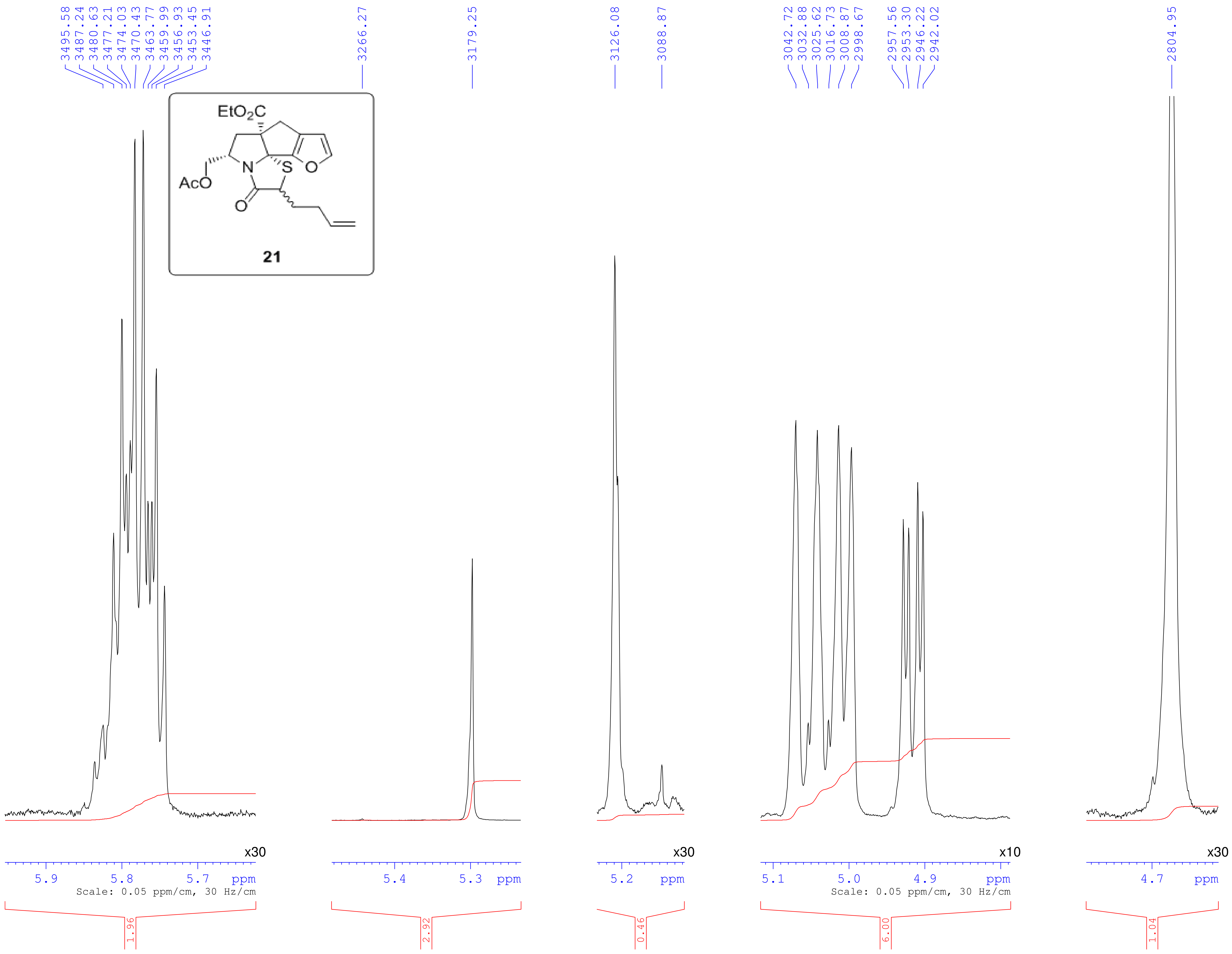

JC–333–4
C13CPD.ucl CDCl3 {V:\Bruker\TOPSPIN} mjp 58

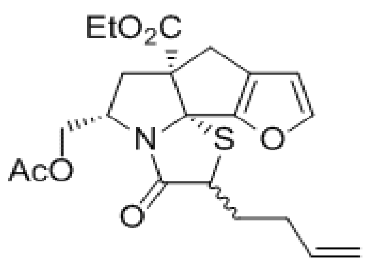


21

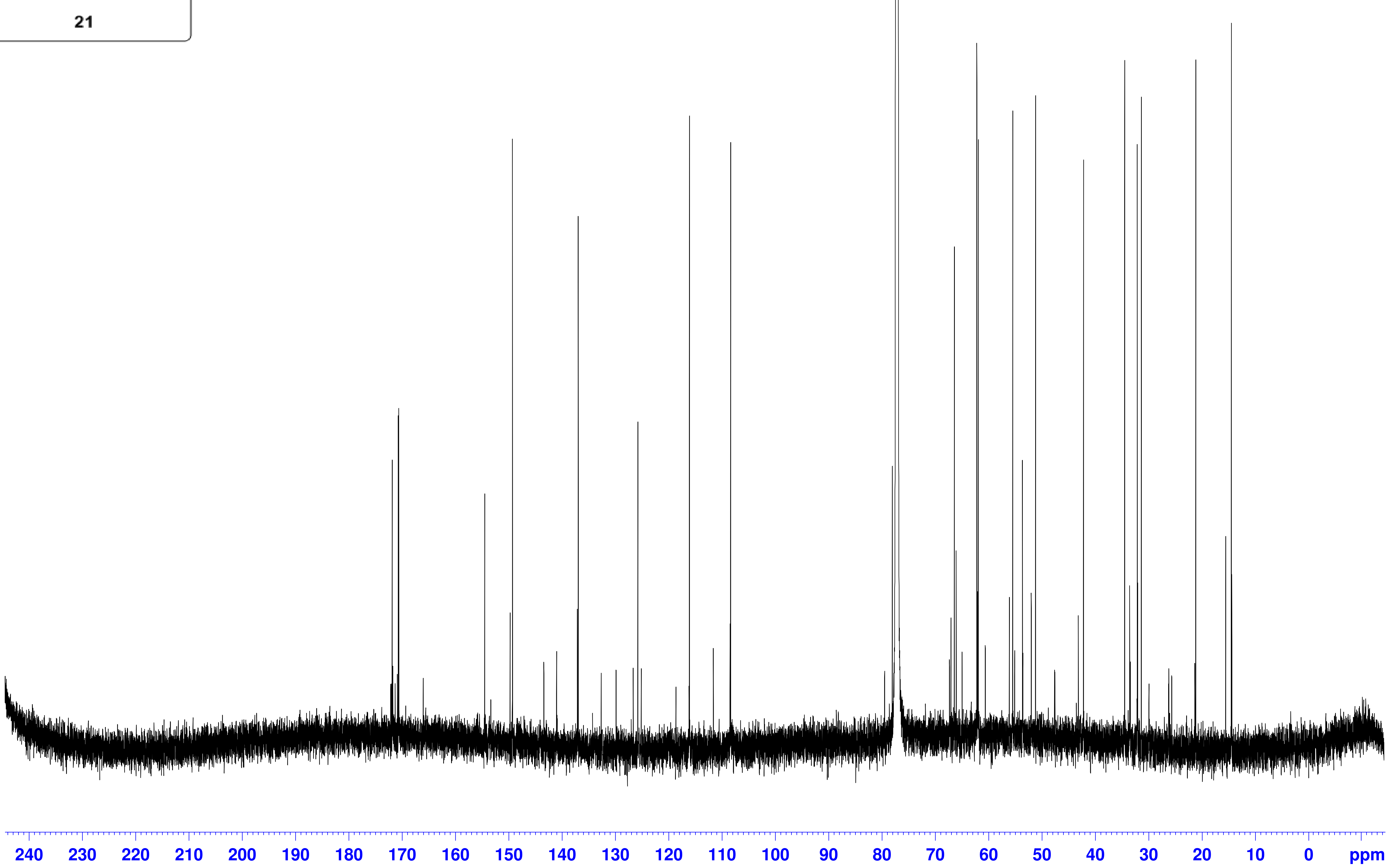


UCL

| | | |
|---|---|---|
| NAME | JC-333-4 | |
| EXPNO | 12 | |
| PROCNO | 1 | |
| Date_ | 20120515 | |
| Time | 3.06 | |
| INSTRUM | AV600 | |
| PROBHD | 5 mm CPDCH 13C | |
| PULPROG | zgpg30 | |
| TD | 70308 | |
| SOLVENT | CDCl3 | |
| NS | 512 | |
| DS | 0 | |
| SWH | 39062.500 | H |
| FIDRES | 0.555591 | H |
| AQ | 0.8999924 | s |
| RG | 1030 | |
| DW | 12.800 | u |
| DE | 21.12 | u |
| TE | 298.0 | K |
| D1 | 2.00000000 | s |
| D11 | 0.03000000 | s |
| TD0 | 1 | |
| ======== CHANNEL f1 ===== | | |
| NUC1 | 13C | |
| P1 | 9.80 | u |
| PL1 | 5.00 | d |
| PL1W | 26.76886177 | W |
| SFO1 | 150.9201628 | M |
| ======== CHANNEL f2 ===== | | |
| CPDPRG2 | waltz16 | |
| NUC2 | 1H | |
| PCPD2 | 70.00 | u |
| PL2 | 1.00 | d |
| PL12 | 17.23 | d |
| PL13 | 20.00 | d |
| PL2W | 13.76731014 | W |
| PL12W | 0.32798135 | W |
| PL13W | 0.17332016 | W |
| SFO2 | 600.1324005 | M |
| SI | 65536 | |
| SF | 150.9027930 | M |
| WDW | EM | |
| SSB | 0 | |
| LB | 1.00 | H |
| GB | 0 | |
| PC | 1.40 | |

JC–333–4
COSY.ucl CDCl3 {V:\Bruker\TOPSPIN} mjp 58

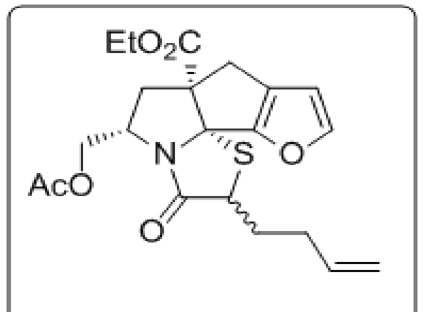


21

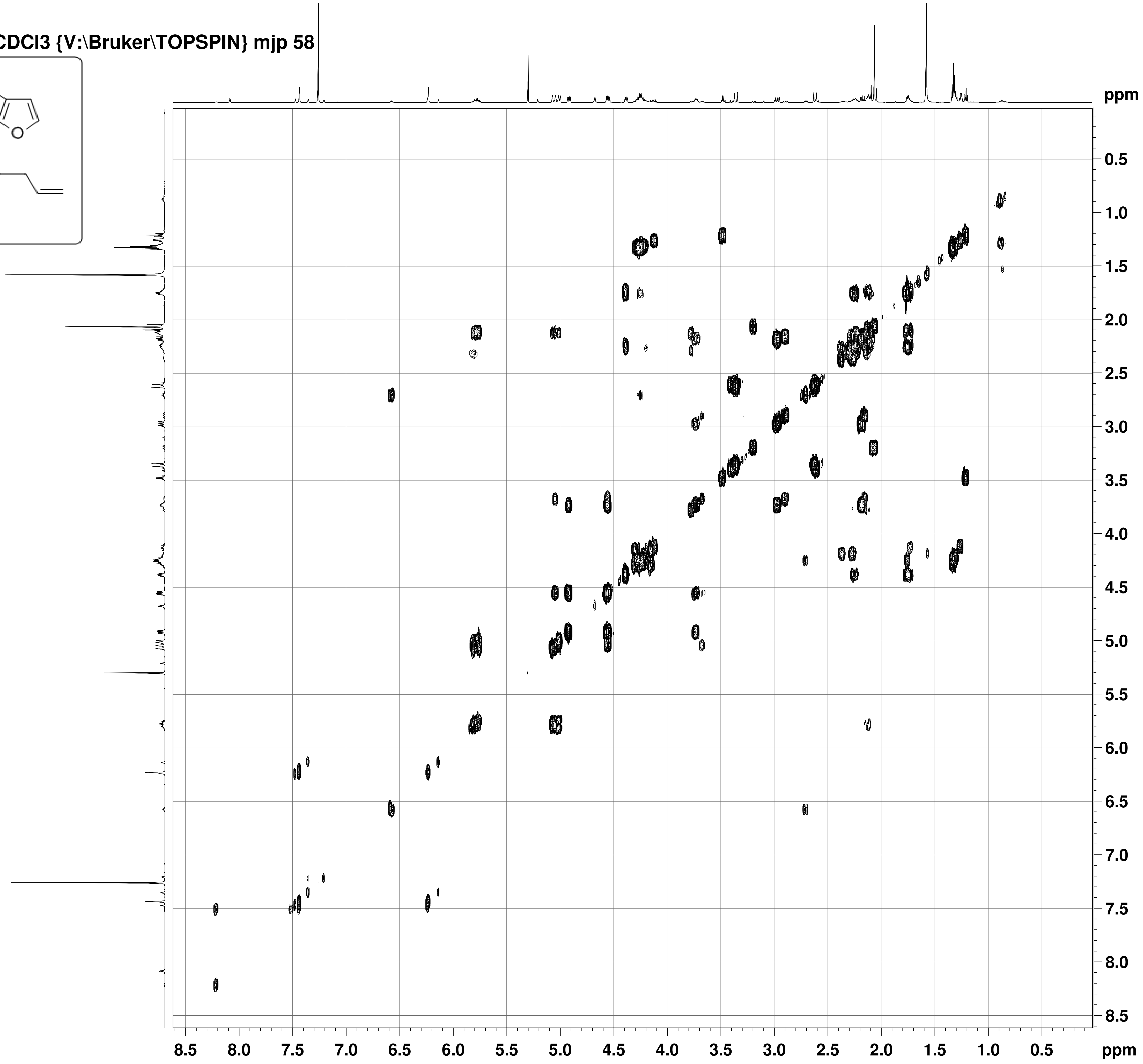


| | |
|---|---|
| NAME | JC-333-4 |
| EXPNO | 11 |
| PROCNO | 1 |
| Date_ | 20120515 |
| Time | 2.24 |
| INSTRUM | AV600 |
| PROBHD | 5 mm CPDCH 13C |
| PULPROG | cosygpmfqf |
| TD | 2048 |
| SOLVENT | CDCl3 |
| NS | 4 |
| DS | 8 |
| SWH | 5154.639 Hz |
| FIDRES | 2.516914 Hz |
| AQ | 0.1987060 sec |
| RG | 2050 |
| DW | 97.000 usec |
| DE | 6.50 usec |
| TE | 298.0 K |
| D0 | 0.00000300 sec |
| D1 | 1.70140100 sec |
| D13 | 0.00000400 sec |
| D16 | 0.00020000 sec |
| IN0 | 0.00019400 sec |
| ======== CHANNEL f1 ======== | |
| NUC1 | 1H |
| P1 | 11.40 usec |
| PL1 | 1.00 dB |
| PL1W | 13.76731014 W |
| SFO1 | 600.1326051 MHz |
| ====== GRADIENT CHANNEL ==== | |
| GPNAM1 | SINE.100 |
| GPNAM2 | SINE.100 |
| GPNAM3 | SINE.100 |
| GPZ1 | 16.00 % |
| GPZ2 | 12.00 % |
| GPZ3 | 40.00 % |
| P16 | 1000.00 usec |
| ND0 | 1 |
| TD | 128 |
| SFO1 | 600.1326 MHz |
| FIDRES | 40.270618 Hz |
| SW | 8.589 ppm |
| FnMODE | QF |
| SI | 1024 |
| SF | 600.1300093 MHz |
| WDW | QSINE |
| SSB | 0 |
| LB | 0.00 Hz |
| GB | 0 |
| PC | 1.40 |
| SI | 1024 |
| MC2 | QF |
| SF | 600.1300093 MHz |
| WDW | QSINE |
| SSB | 0 |
| LB | 0.00 Hz |
| GB | 0 |

# JC–333–4
## C13DEPT135.ucl CDCl3 {V:\Bruker\TOPSPIN} mjp 58

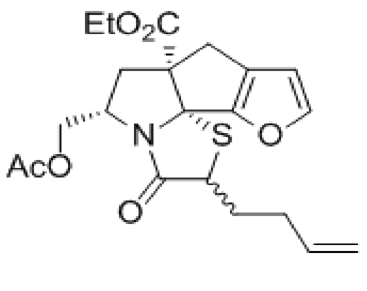

**21**

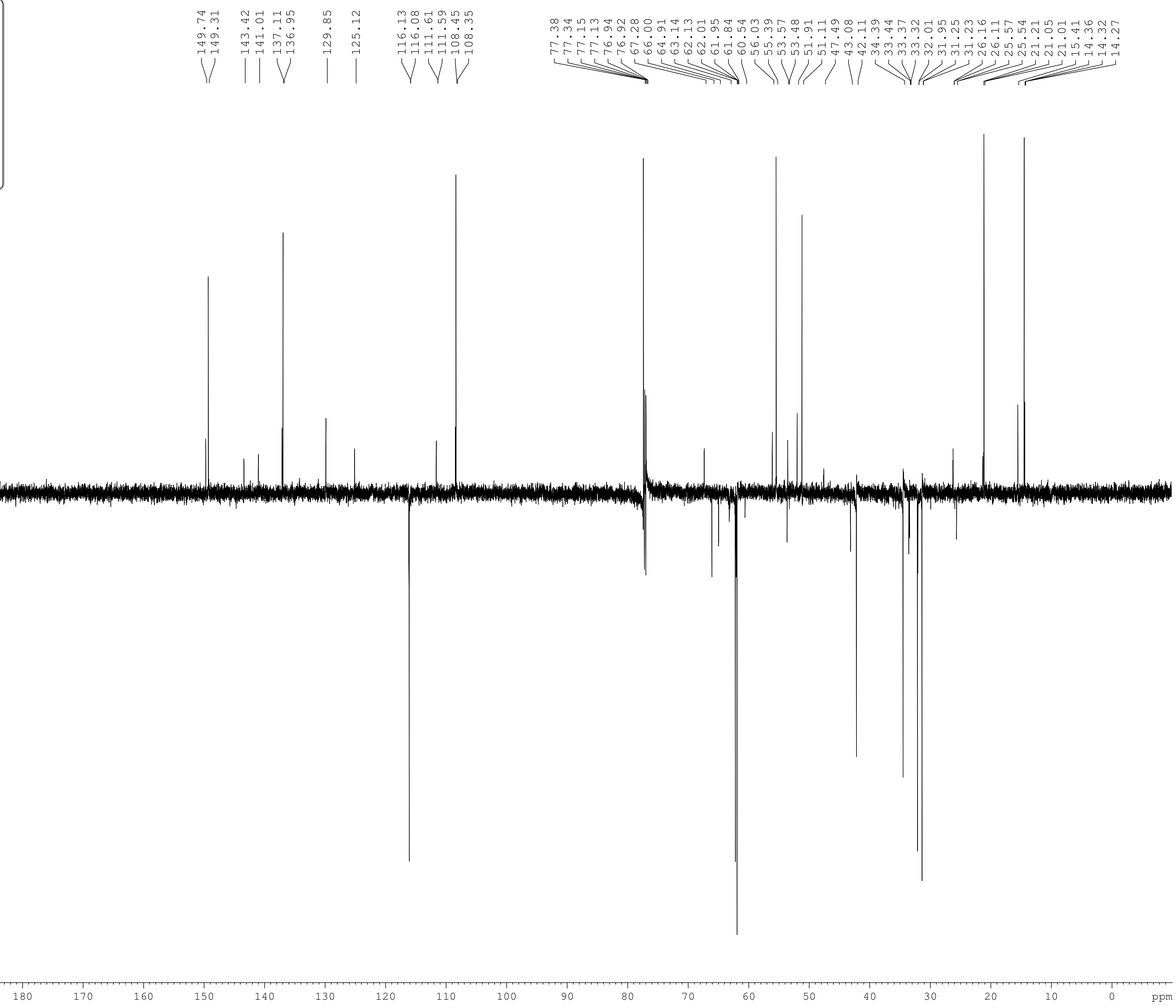


| | |
|---|---|
| NAME | JC-333-4 |
| EXPNO | 15 |
| PROCNO | 1 |
| Date_ | 20120515 |
| Time | 4.32 |
| INSTRUM | AV600 |
| PROBHD | 5 mm CPDCH 13C |
| PULPROG | dept135 |
| TD | 70308 |
| SOLVENT | CDCl3 |
| NS | 256 |
| DS | 4 |
| SWH | 39062.500 Hz |
| FIDRES | 0.555591 Hz |
| AQ | 0.8999924 sec |
| RG | 256 |
| DW | 12.800 usec |
| DE | 6.50 usec |
| TE | 298.0 K |
| CNST2 | 145.0000000 |
| D1 | 2.00000000 sec |
| D2 | 0.00344828 sec |
| D12 | 0.00002000 sec |
| TD0 | 1 |

======== CHANNEL f1 ========

| | |
|---|---|
| NUC1 | 13C |
| P1 | 9.80 usec |
| P2 | 19.60 usec |
| PL1 | 5.00 dB |
| PL1W | 26.76886177 W |
| SFO1 | 150.9201628 MHz |

======== CHANNEL f2 ========

| | |
|---|---|
| CPDPRG2 | waltz16 |
| NUC2 | 1H |
| P3 | 10.80 usec |
| P4 | 21.60 usec |
| PCPD2 | 70.00 usec |
| PL2 | 1.00 dB |
| PL12 | 17.23 dB |
| PL2W | 13.76731014 W |
| PL12W | 0.32798135 W |
| SFO2 | 600.1324005 MHz |
| SI | 65536 |
| SF | 150.9027930 MHz |
| WDW | EM |
| SSB | 0 |
| LB | 1.00 Hz |
| GB | 0 |
| PC | 1.40 |

JC–333–4
HSQC.ucl CDCl3 {V:\Bruker\TOPSPIN} mjp 58

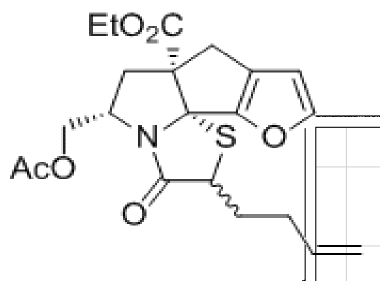


21

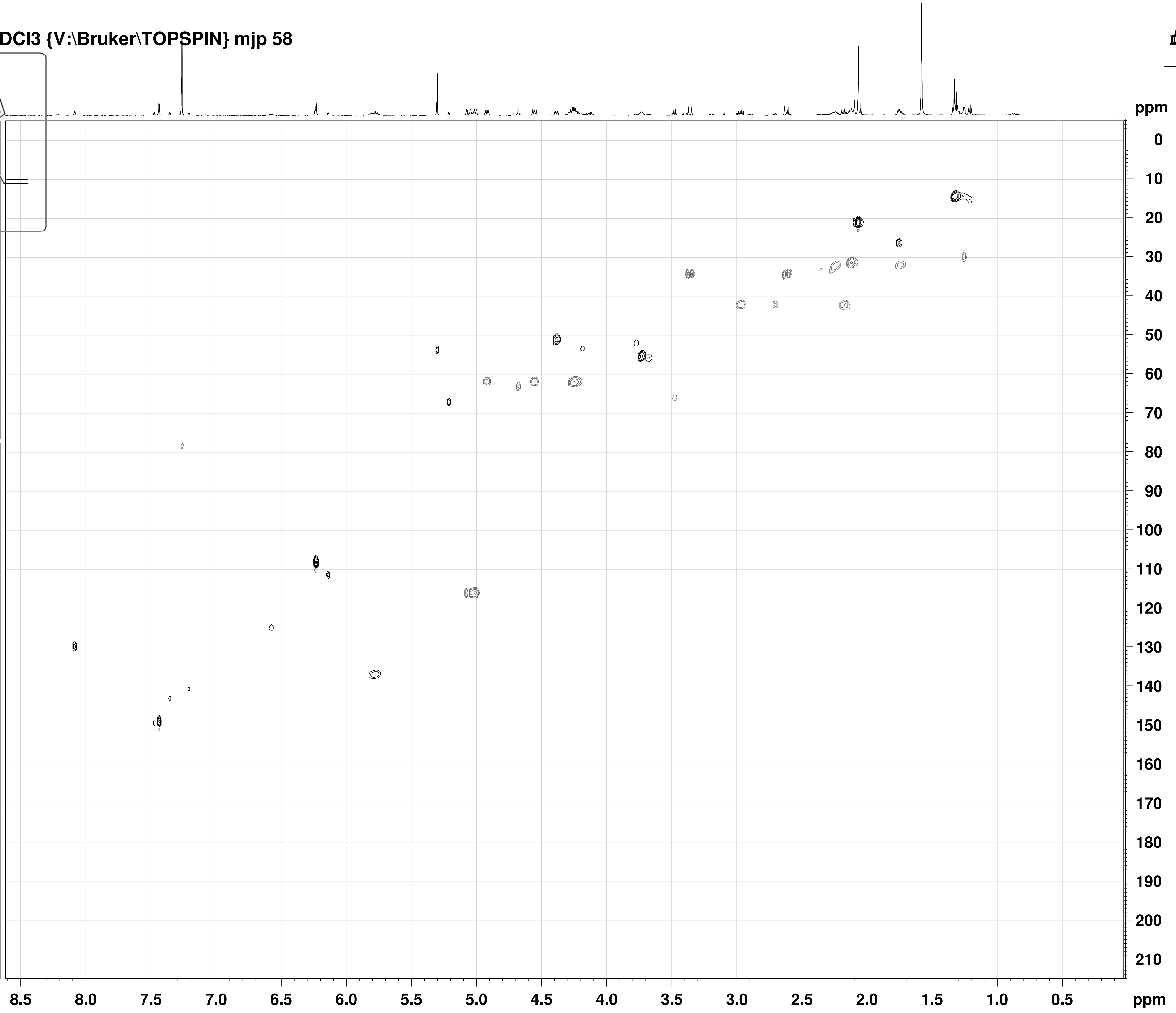


UCL

```
NAME          JC-333-4
EXPNO               13
PROCNO               1
Date_         20120515
Time              3.08
INSTRUM          AV600
PROBHD   5 mm CPDCH 13C
PULPROG  hsqcedetgpsisp2.4
TD                1024
SOLVENT          CDCl3
NS                   8
DS                  32
SWH           5154.639 Hz
FIDRES        5.033827 Hz
AQ           0.0993780 sec
RG                2050
DW              97.000 usec
DE                6.50 usec
TE               298.0 K
CNST2      145.0000000
CNST17      -0.5000000
D0          0.00000300 sec
D1          1.48586905 sec
D2          0.00344828 sec
D4          0.00172414 sec
D11         0.03000000 sec
D16         0.00020000 sec
D21         0.00344828 sec
D24         0.00086207 sec
IN0         0.00001505 sec
L0                   0
L31                  1
TD0                  2

======== CHANNEL f1 ========
NUC1                1H
P1               11.40 usec
P2               22.80 usec
P28               0.00 usec
PL1               1.00 dB
PL1W       13.76731014 W
SFO1      600.1326051 MHz

======== CHANNEL f2 ========
CPDPRG2  bi_p5m4sp_4sp.2
NUC2               13C
P3                9.80 usec
P14             500.00 usec
P24            2000.00 usec
P31            1730.00 usec
P63            1500.00 usec
PL0             120.00 dB
PL2               5.00 dB
PL12             20.74 dB
PL0W        0.00000000 W
PL2W       26.76886177 W
PL12W       0.71388775 W
SFO2      150.9186538 MHz
SP3              13.33 dB
SP7              13.33 dB
SP14             14.82 dB
SP18             18.73 dB
SP31             20.84 dB
SPNAM3   Crp60,0.5,20.1
SPNAM7     Crp60comp.4
SPNAM14  Crp32,1.5,20.2
SPNAM18   Crp60_xfilt.2
SPNAM31  Crp32,1.5,20.2
SPOAL3           0.500
SPOAL7           0.500
SPOAL14          0.500
SPOAL18          0.500
SPOAL31          0.500
SPOFFS3           0.00 Hz
SPOFFS7           0.00 Hz
SPOFFS14          0.00 Hz
SPOFFS18          0.00 Hz
SPOFFS31          0.00 Hz

====== GRADIENT CHANNEL =====
GPNAM1        SINE.100
GPNAM2        SINE.100
GPNAM3        SINE.100
GPNAM4        SINE.100
GPZ1             80.00 %
GPZ2             20.10 %
GPZ3             11.00 %
GPZ4             -5.00 %
P16            1000.00 usec
P19             600.00 usec
ND0                  2
TD                 128
SFO1          150.9187 MHz
FIDRES      259.391449 Hz
SW             220.000 ppm
FnMODE    Echo-Antiecho
SI                1024
SF         600.1300093 MHz
WDW              QSINE
SSB                  2
LB                0.00 Hz
GB                   0
PC                1.40
SI                1024
MC2        echo-antiecho
SF         150.9027771 MHz
WDW              QSINE
SSB                  2
LB                0.00 Hz
GB                   0
```

**JC–333–4**
**HMBC.ucl CDCl3 {V:\Bruker\TOPSPIN} mjp 58**

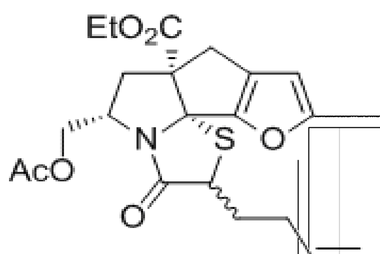


21

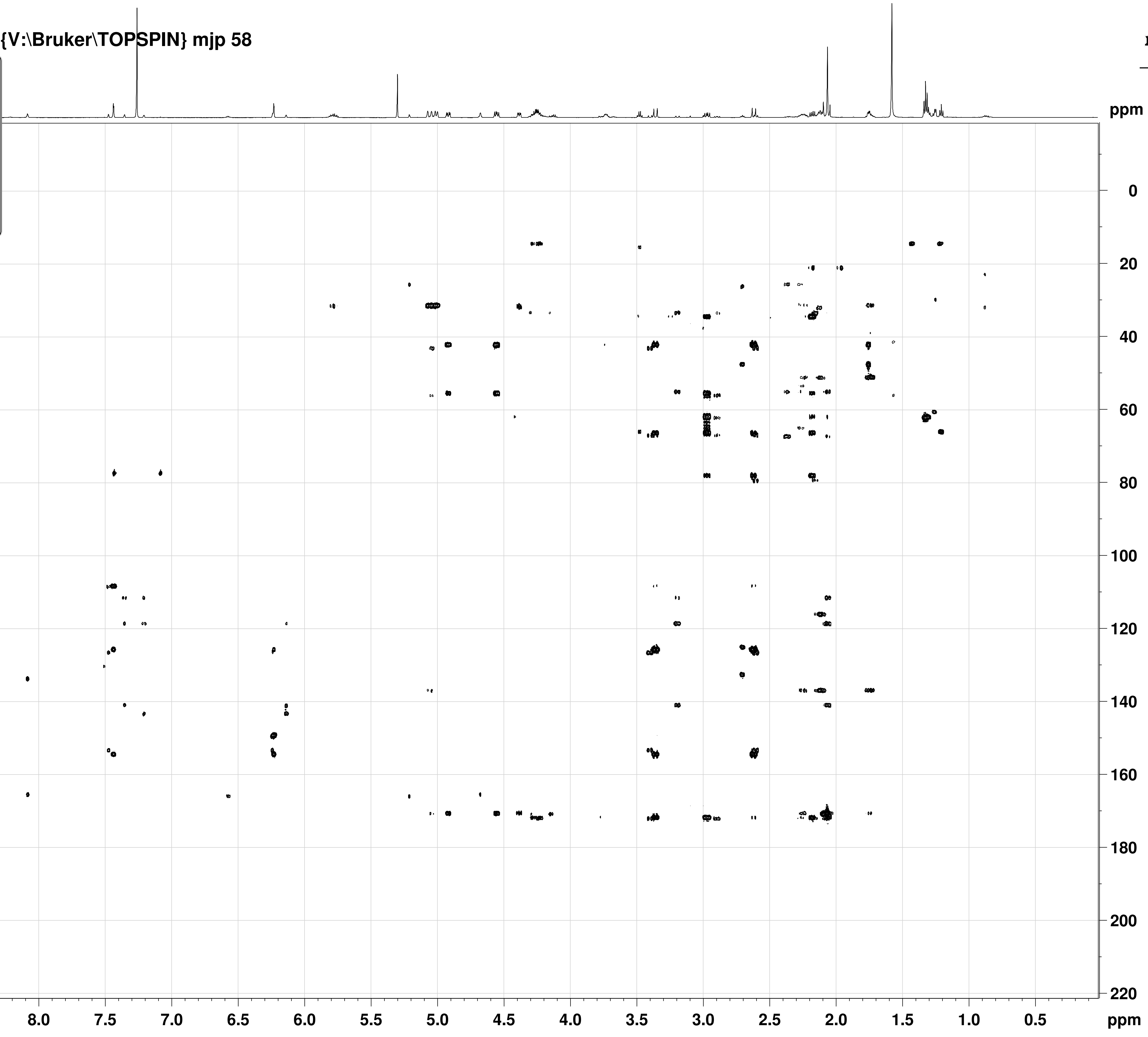


UCL

```
NAME              JC-333-4
EXPNO                   14
PROCNO                   1
Date_             20120515
Time                  3.36
INSTRUM              AV600
PROBHD   5 mm CPDCH 13C
PULPROG     hmbcetgpl3nd
TD                    4096
SOLVENT              CDCl3
NS                       8
DS                      16
SWH             5154.639 Hz
FIDRES          1.258457 Hz
AQ             0.3973620 sec
RG                    2050
DW                  97.000 usec
DE                    6.50 usec
TE                   298.0 K
CNST6          120.0000000
CNST7          160.0000000
CNST13          10.0000000
CNST30           0.5981152
D0            0.00000300 sec
D1            0.78048259 sec
D6            0.05000000 sec
D16           0.00020000 sec
IN0           0.00001380 sec

======== CHANNEL f1 ========
NUC1                    1H
P1                   11.40 usec
P2                   22.80 usec
PL1                   1.00 dB
PL1W           13.76731014 W
SFO1           600.1326051 MHz

======== CHANNEL f2 ========
NUC2                   13C
P3                    9.80 usec
P24                2000.00 usec
PL2                   5.00 dB
PL2W           26.76886177 W
SFO2           150.9178993 MHz
SP7                  13.33 dB
SPNAM7         Crp60comp.4
SPOAL7               0.500
SPOFFS7               0.00 Hz

====== GRADIENT CHANNEL =====
GPNAM1           SINE.100
GPNAM3           SINE.100
GPNAM4           SINE.100
GPNAM5           SINE.100
GPNAM6           SINE.100
GPZ1                80.00 %
GPZ3                14.00 %
GPZ4                -8.00 %
GPZ5                -4.00 %
GPZ6                -2.00 %
P16               1000.00 usec
ND0                     2
TD                    256
SFO1             150.9179 MHz
FIDRES         141.485535 Hz
SW                240.000 ppm
FnMODE      Echo-Antiecho
SI                   2048
SF            600.1300105 MHz
WDW                  SINE
SSB                     2
LB                   0.00 Hz
GB                      0
PC                   1.40
SI                   1024
MC2          echo-antiecho
SF            150.9027756 MHz
WDW                  SINE
SSB                     2
LB                   0.00 Hz
GB                      0
```

**JC–333–2**
**PROTON.ucl CDCl3 {V:\Bruker\TOPSPIN} mjp 25**

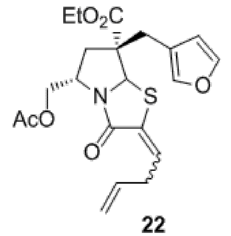


**22**

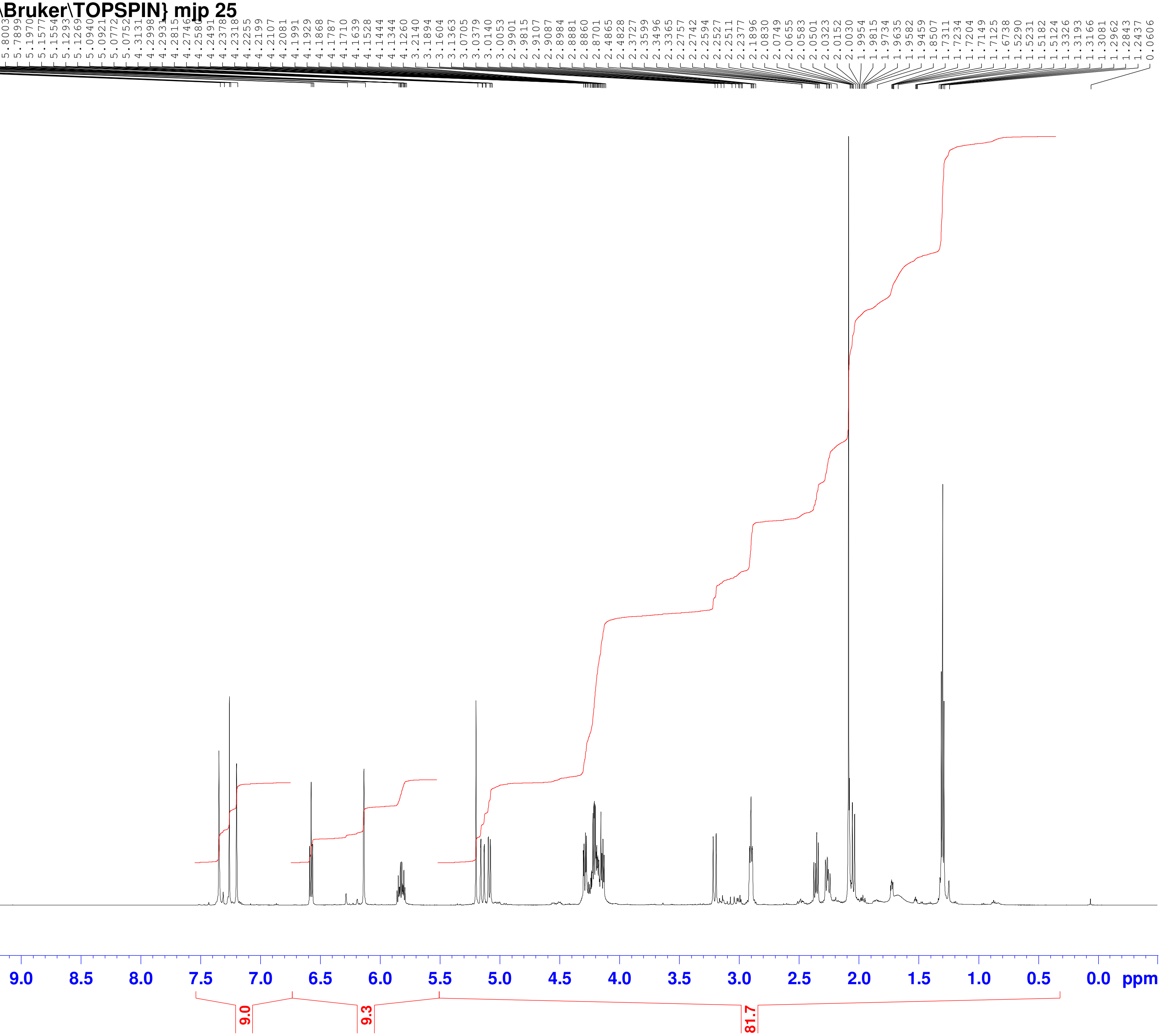


| | |
|---|---|
| NAME | JC-333-2 r |
| EXPNO | 10 |
| PROCNO | 1 |
| Date_ | 20120229 |
| Time | 17.37 |
| INSTRUM | AV600 |
| PROBHD | 5 mm CPDCH 13C |
| PULPROG | zg30 |
| TD | 98682 |
| SOLVENT | CDCl3 |
| NS | 8 |
| DS | 0 |
| SWH | 12335.526 Hz |
| FIDRES | 0.125003 Hz |
| AQ | 3.9999604 sec |
| RG | 28.5 |
| DW | 40.533 use |
| DE | 10.48 use |
| TE | 298.0 K |
| D1 | 1.00000000 sec |
| TD0 | 1 |

======== CHANNEL f1 ========

| | |
|---|---|
| NUC1 | 1H |
| P1 | 11.40 use |
| PL1 | 1.00 dB |
| PL1W | 13.76731014 W |
| SFO1 | 600.1337061 MHz |
| SI | 32768 |
| SF | 600.1300116 MHz |
| WDW | EM |
| SSB | 0 |
| LB | 0.30 Hz |
| GB | 0 |
| PC | 1.40 |

V:BrukerTOPSPIN/mjp/JC−333−2 r/10/1
Integral regions #2 to #2

30 Hz/cm

JC−333−2
PROTON.ucl CDCl3 {V:\Bruker\TOPSPIN} mjp 25

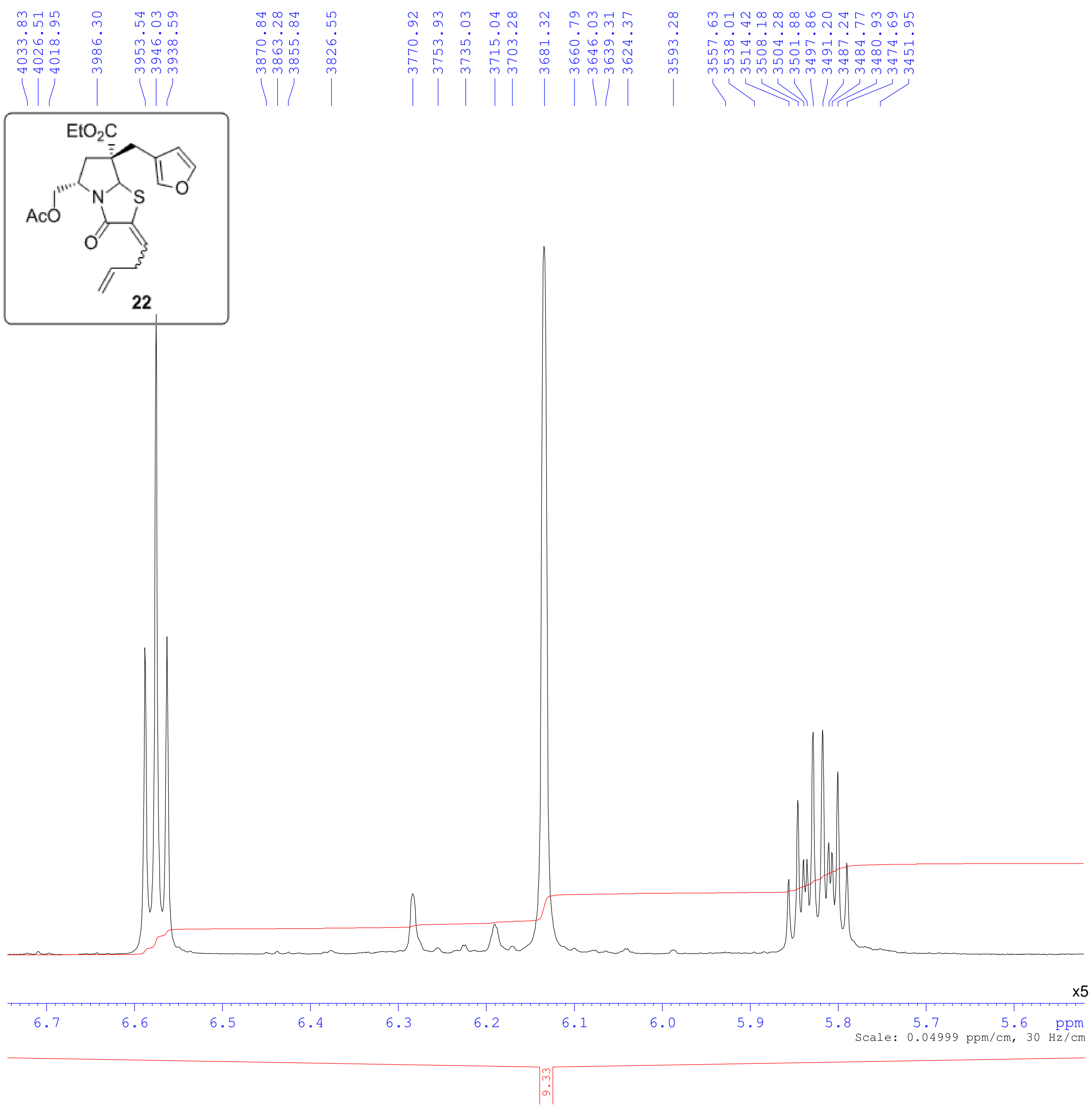

JC-333-2
C13CPD.ucl CDCl3 {V:\Bruker\TOPSPIN} mjp 25

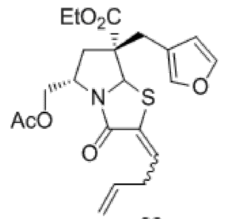


22

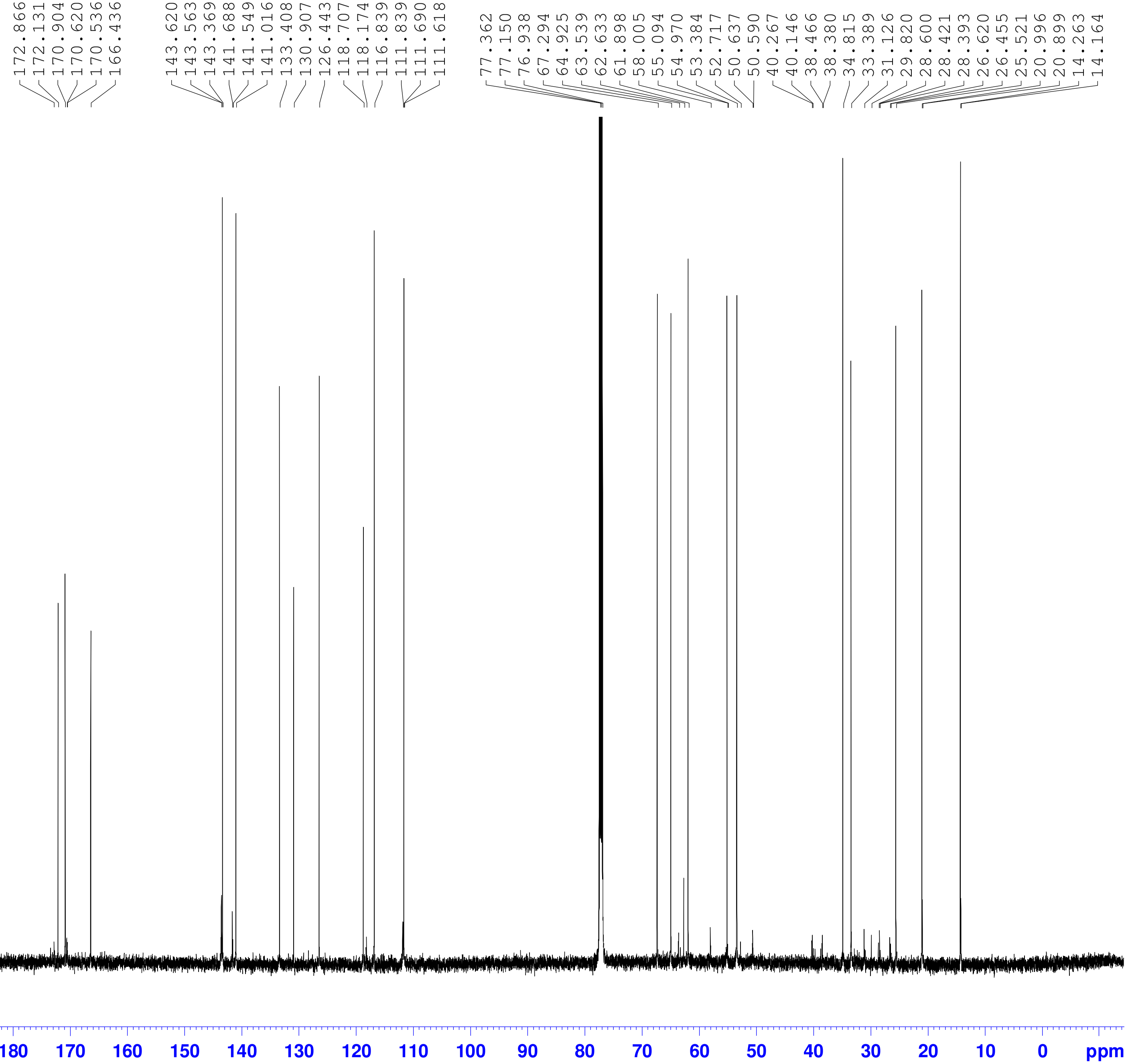


UCL



| | |
|---|---|
| NAME | JC-333-2 r |
| EXPNO | 12 |
| PROCNO | 1 |
| Date_ | 20120229 |
| Time | 17.49 |
| INSTRUM | AV600 |
| PROBHD | 5 mm CPDCH 13C |
| PULPROG | zgpg30 |
| TD | 70308 |
| SOLVENT | CDCl3 |
| NS | 128 |
| DS | 0 |
| SWH | 39062.500 H |
| FIDRES | 0.555591 H |
| AQ | 0.8999924 s |
| RG | 1030 |
| DW | 12.800 u |
| DE | 21.12 u |
| TE | 298.0 K |
| D1 | 2.00000000 s |
| D11 | 0.03000000 s |
| TD0 | 1 |
| ======== CHANNEL f1 ===== | |
| NUC1 | 13C |
| P1 | 9.80 u |
| PL1 | 5.00 d |
| PL1W | 26.76886177 W |
| SFO1 | 150.9201628 M |
| ======== CHANNEL f2 ===== | |
| CPDPRG2 | waltz16 |
| NUC2 | 1H |
| PCPD2 | 70.00 u |
| PL2 | 1.00 d |
| PL12 | 17.23 d |
| PL13 | 20.00 d |
| PL2W | 13.76731014 W |
| PL12W | 0.32798135 W |
| PL13W | 0.17332016 W |
| SFO2 | 600.1324005 M |
| SI | 65536 |
| SF | 150.9027930 M |
| WDW | EM |
| SSB | 0 |
| LB | 1.00 H |
| GB | 0 |
| PC | 1.40 |

JC-333-2 r.011.001.2rr.esp

COSY

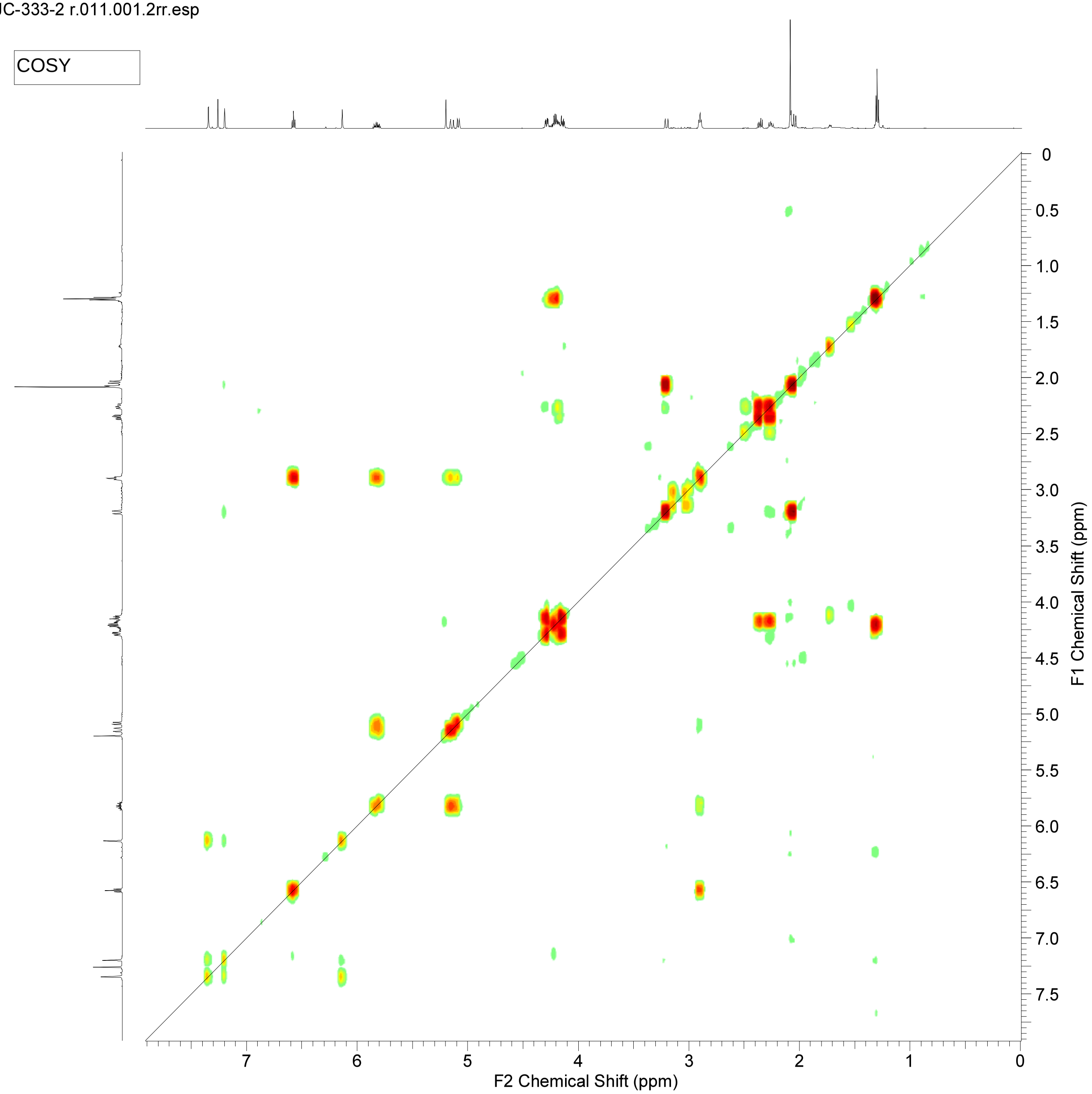


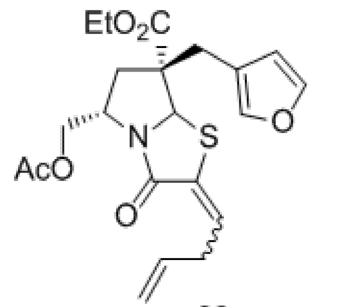


22

JC-333-2 r.011.001.2rr.esp

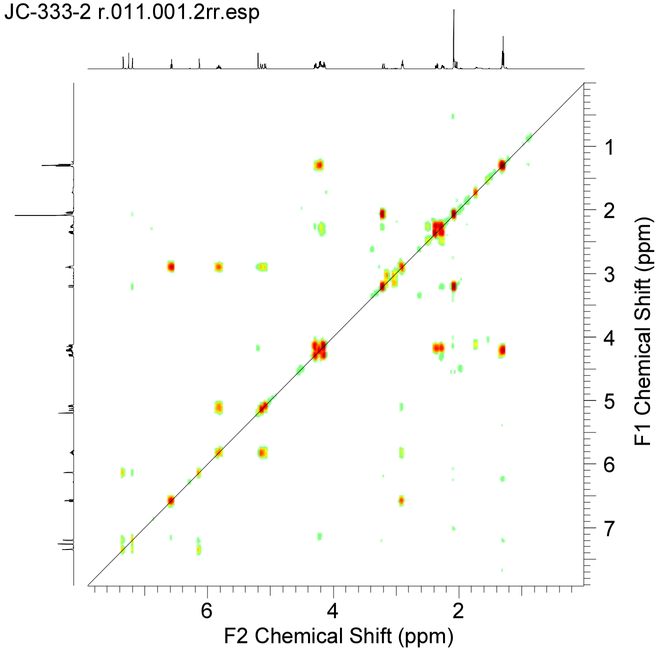

**JC−333−2**
**C13DEPT135.ucl CDCl3 {V:\Bruker\TOPSPIN} mjp 25**

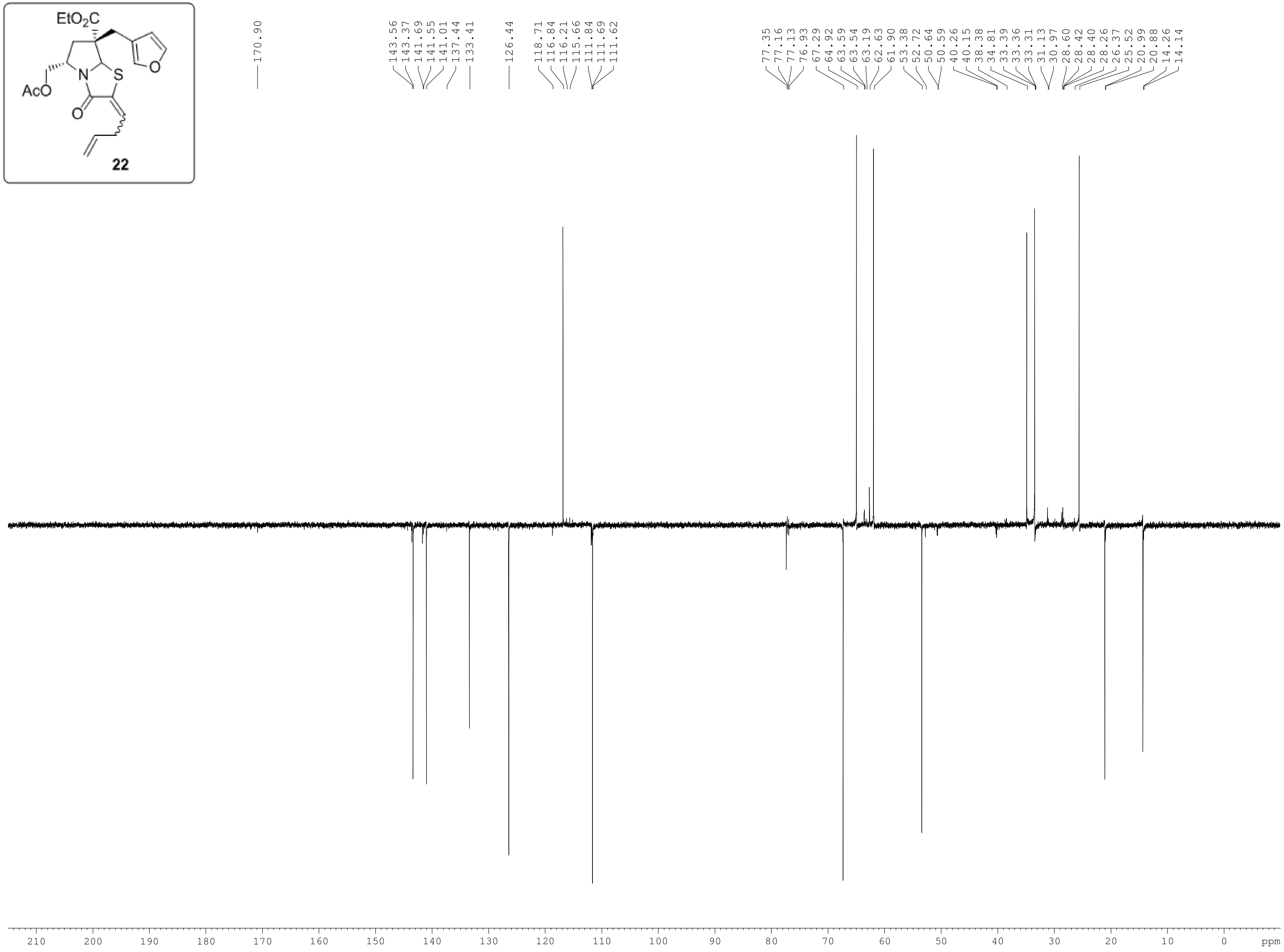


| | |
|---|---|
| NAME | JC-333-2 r |
| EXPNO | 15 |
| PROCNO | 1 |
| Date_ | 20120301 |
| Time | 0.09 |
| INSTRUM | AV600 |
| PROBHD | 5 mm CPDCH 13C |
| PULPROG | dept135 |
| TD | 70308 |
| SOLVENT | CDCl3 |
| NS | 64 |
| DS | 4 |
| SWH | 39062.500 Hz |
| FIDRES | 0.555591 Hz |
| AQ | 0.8999924 sec |
| RG | 256 |
| DW | 12.800 usec |
| DE | 6.50 usec |
| TE | 298.0 K |
| CNST2 | 145.0000000 |
| D1 | 2.00000000 sec |
| D2 | 0.00344828 sec |
| D12 | 0.00002000 sec |
| TD0 | 1 |
| ======== CHANNEL f1 ===== | |
| NUC1 | 13C |
| P1 | 9.80 usec |
| P2 | 19.60 usec |
| PL1 | 5.00 dB |
| PL1W | 26.76886177 W |
| SFO1 | 150.9201628 MHz |
| ======== CHANNEL f2 ===== | |
| CPDPRG2 | waltz16 |
| NUC2 | 1H |
| P3 | 10.80 usec |
| P4 | 21.60 usec |
| PCPD2 | 70.00 usec |
| PL2 | 1.00 dB |
| PL12 | 17.23 dB |
| PL2W | 13.76731014 W |
| PL12W | 0.32798135 W |
| SFO2 | 600.1324005 MHz |
| SI | 65536 |
| SF | 150.9027930 MHz |
| WDW | EM |
| SSB | 0 |
| LB | 1.00 Hz |
| GB | 0 |
| PC | 1.40 |

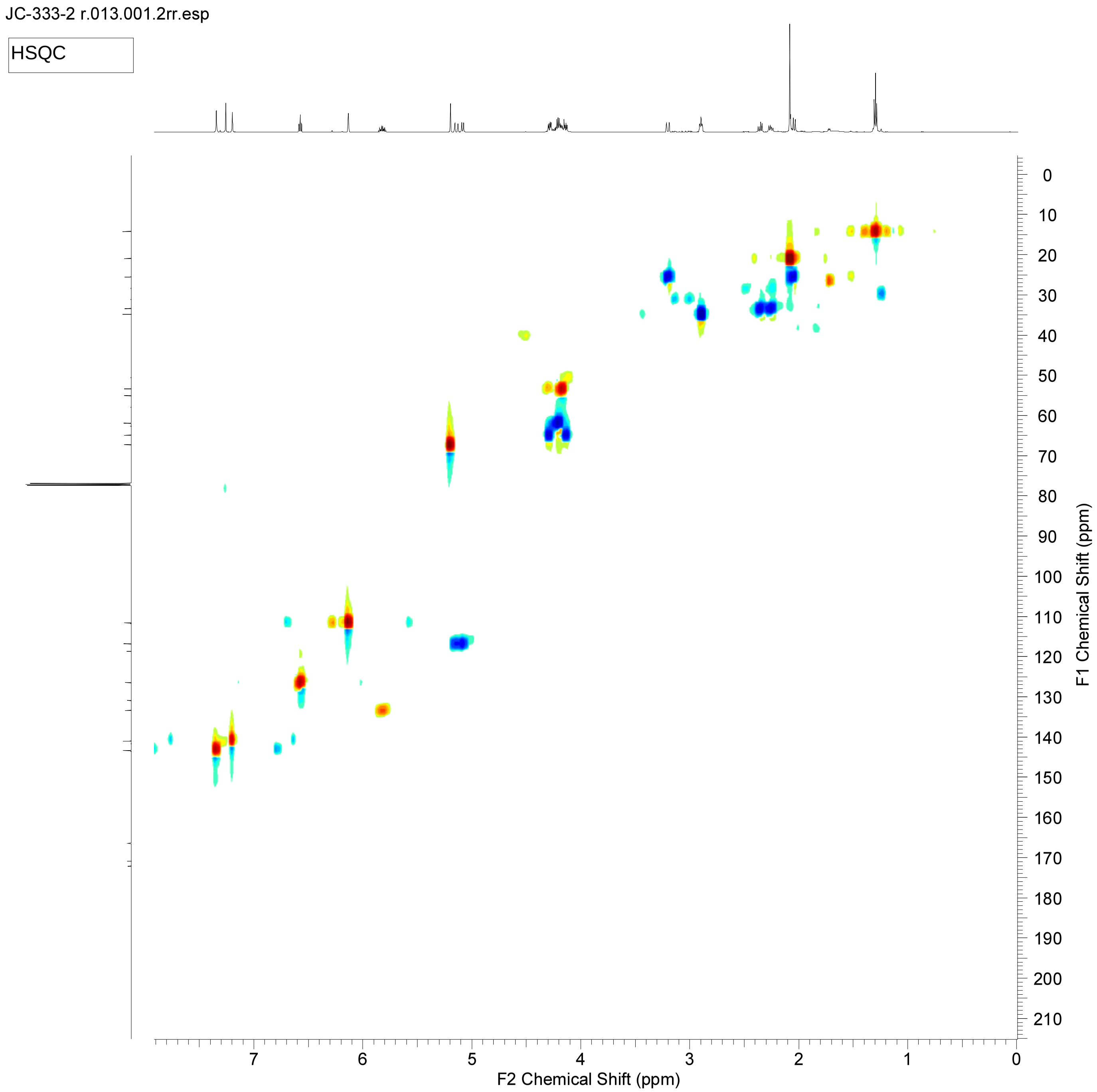
JC-333-2 r.013.001.2rr.esp
HSQC
F2 Chemical Shift (ppm)
F1 Chemical Shift (ppm)

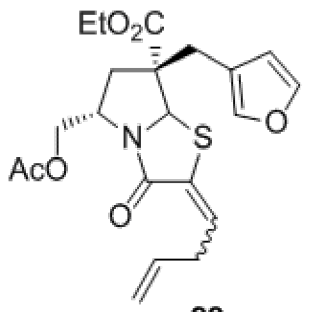
EtO2C
AcO
N
S
O
O

22

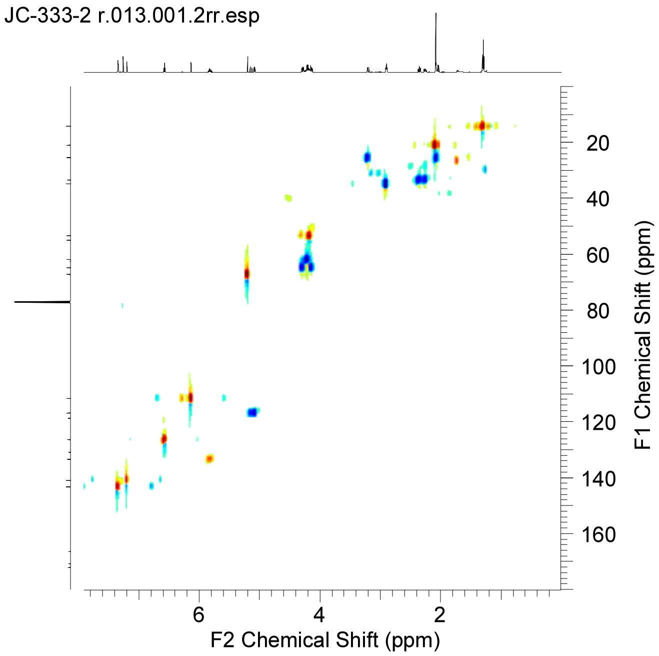
JC-333-2 r.013.001.2rr.esp
F2 Chemical Shift (ppm)
F1 Chemical Shift (ppm)

JC-333-2 r.014.001.2rr.esp

HMBC

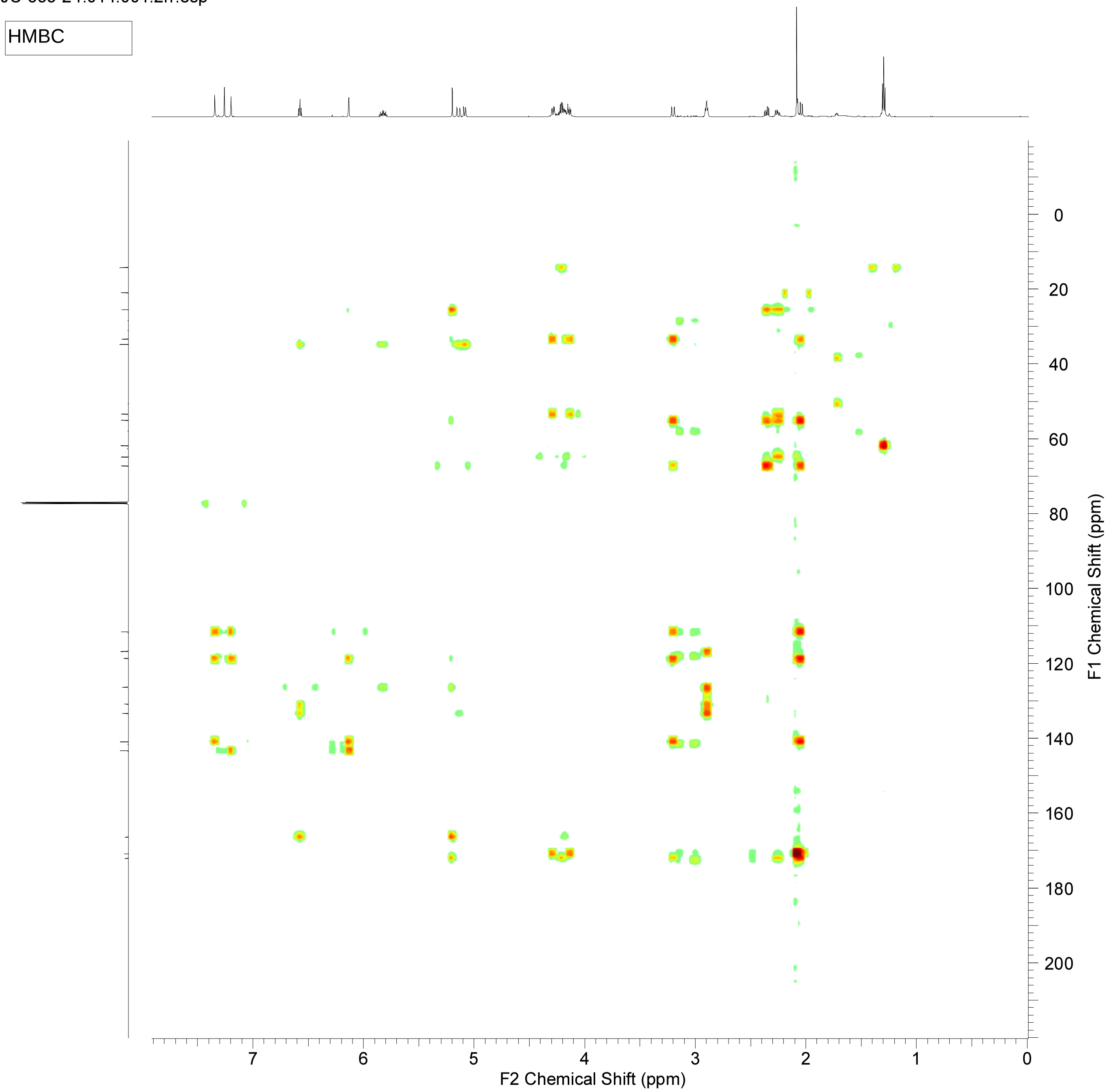


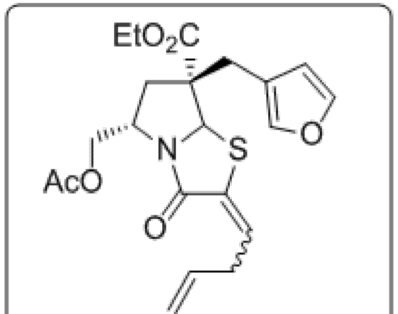


22

JC-333-2 r.014.001.2rr.esp

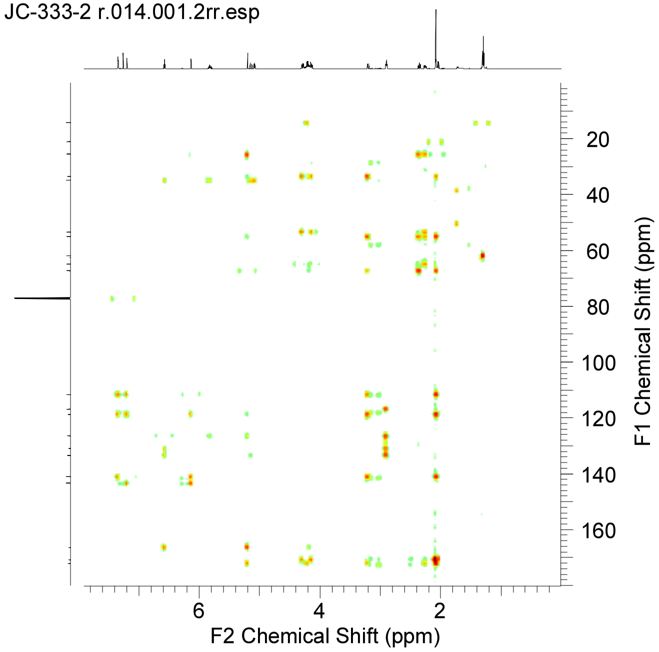

**JC–578–2**
**PROTON.ucl CDCl3 {V:\Bruker\TOPSPIN} mjp 7**

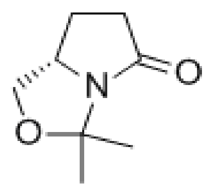

**27**

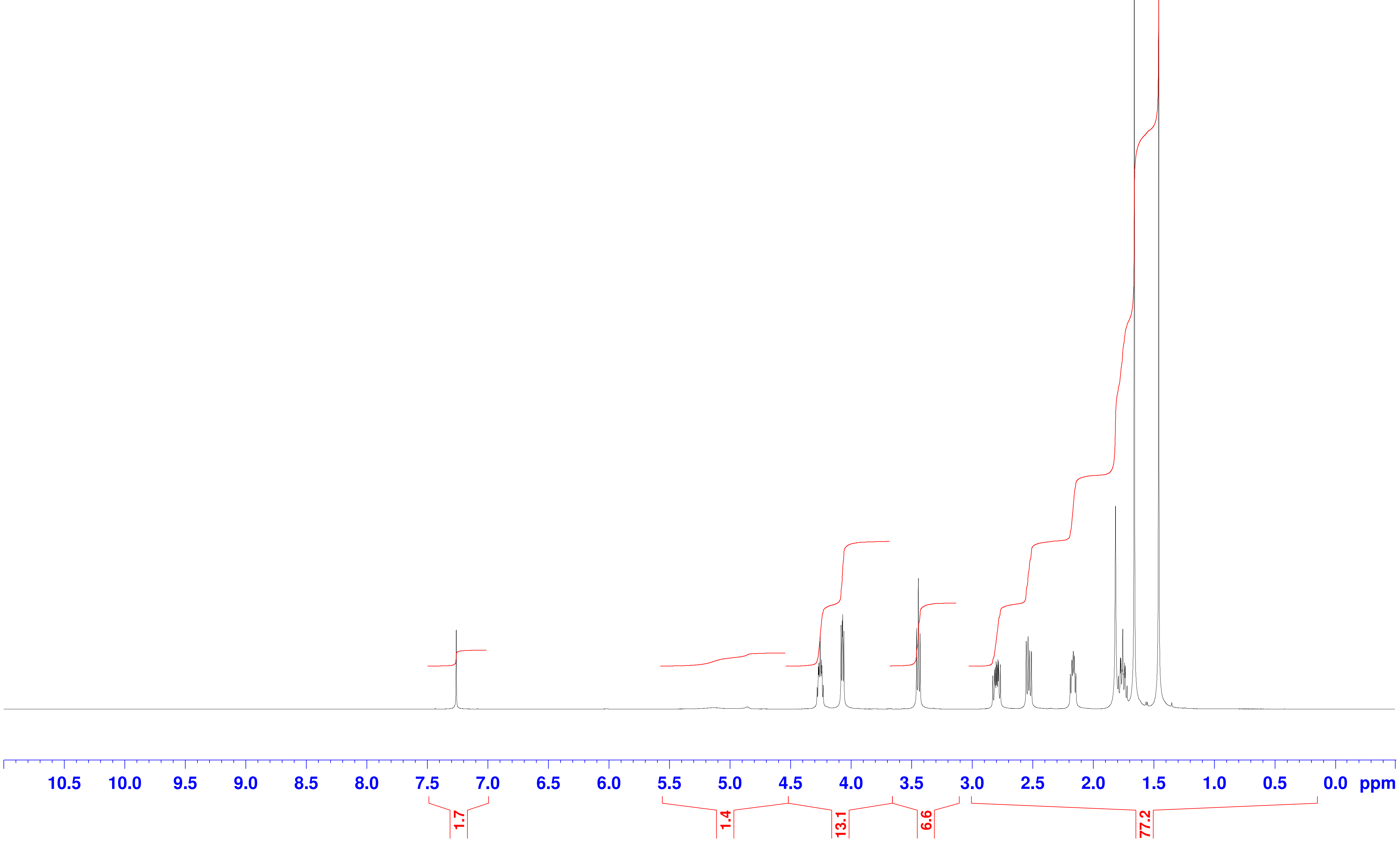


| | |
|---|---|
| NAME | JC-578-2 |
| EXPNO | 10 |
| PROCNO | 1 |
| Date_ | 20110817 |
| Time | 10.50 |
| INSTRUM | AV600 |
| PROBHD | 5 mm CPDCH 13C |
| PULPROG | zg30 |
| TD | 98682 |
| SOLVENT | CDCl3 |
| NS | 8 |
| DS | 0 |
| SWH | 12335.526 Hz |
| FIDRES | 0.125003 Hz |
| AQ | 3.9999604 sec |
| RG | 28.5 |
| DW | 40.533 use |
| DE | 10.48 use |
| TE | 298.0 K |
| D1 | 1.00000000 sec |
| TD0 | 1 |

======== CHANNEL f1 =======

| | |
|---|---|
| NUC1 | 1H |
| P1 | 11.40 use |
| PL1 | 1.00 dB |
| PL1W | 13.76731014 W |
| SFO1 | 600.1337061 MHz |
| SI | 32768 |
| SF | 600.1300116 MHz |
| WDW | EM |
| SSB | 0 |
| LB | 0.30 Hz |
| GB | 0 |
| PC | 1.40 |

JC–578–2
C13CPD.ucl CDCl3 {V:\Bruker\TOPSPIN} mjp 7

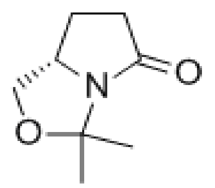

27

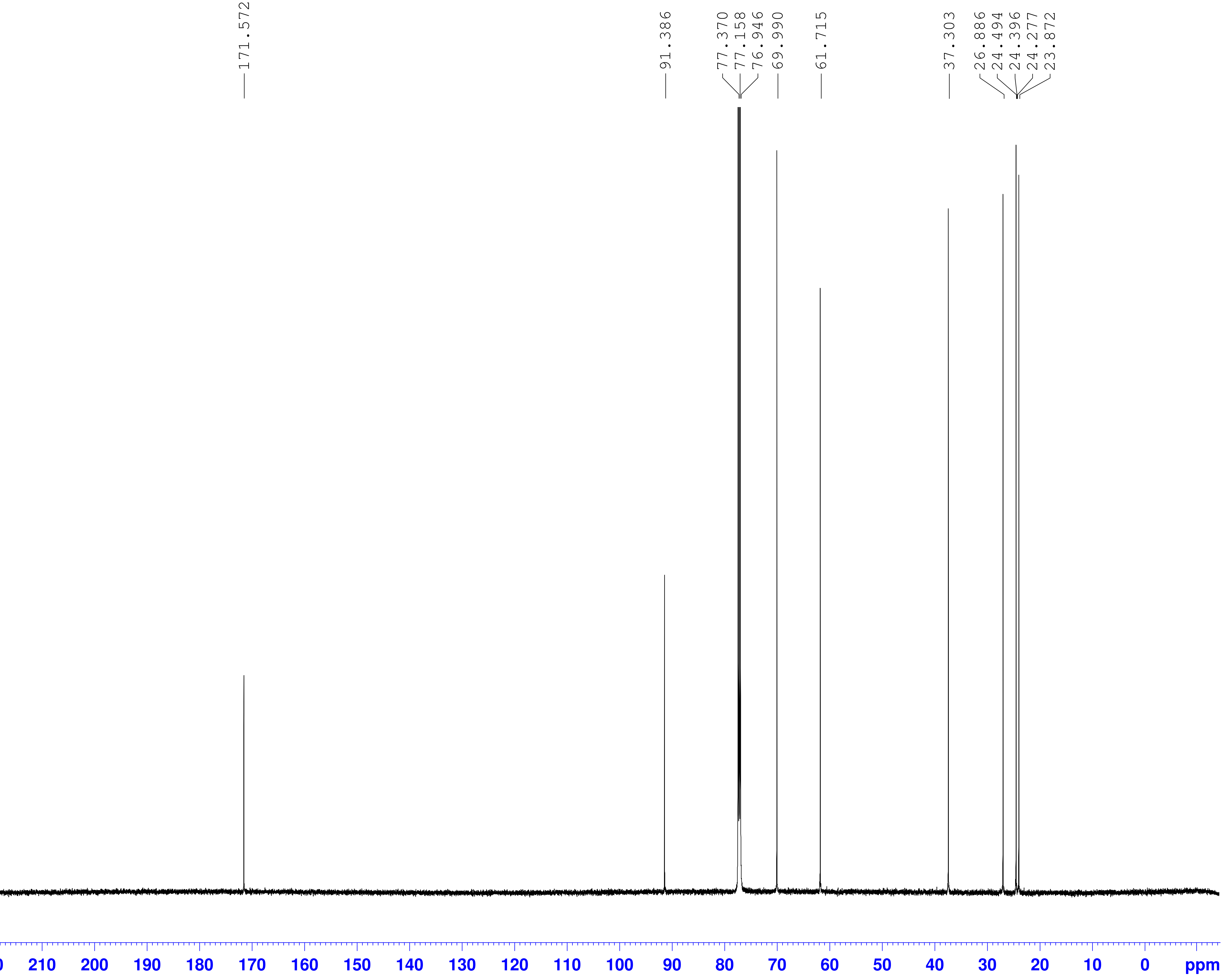


| | |
|---|---|
| NAME | JC-578-2 |
| EXPNO | 12 |
| PROCNO | 1 |
| Date_ | 20110817 |
| Time | 11.02 |
| INSTRUM | AV600 |
| PROBHD | 5 mm CPDCH 13C |
| PULPROG | zgpg30 |
| TD | 70308 |
| SOLVENT | CDCl3 |
| NS | 128 |
| DS | 0 |
| SWH | 39062.500 H |
| FIDRES | 0.555591 H |
| AQ | 0.8999924 s |
| RG | 1030 |
| DW | 12.800 u |
| DE | 21.12 u |
| TE | 298.0 K |
| D1 | 2.00000000 s |
| D11 | 0.03000000 s |
| TD0 | 1 |
| ======== CHANNEL f1 ===== | |
| NUC1 | 13C |
| P1 | 9.80 u |
| PL1 | 5.00 d |
| PL1W | 26.76886177 W |
| SFO1 | 150.9201628 M |
| ======== CHANNEL f2 ===== | |
| CPDPRG2 | waltz16 |
| NUC2 | 1H |
| PCPD2 | 70.00 u |
| PL2 | 1.00 d |
| PL12 | 17.23 d |
| PL13 | 20.00 d |
| PL2W | 13.76731014 W |
| PL12W | 0.32798135 W |
| PL13W | 0.17332016 W |
| SFO2 | 600.1324005 M |
| SI | 65536 |
| SF | 150.9027930 M |
| WDW | EM |
| SSB | 0 |
| LB | 1.00 H |
| GB | 0 |
| PC | 1.40 |

JC–578–2
COSY.ucl CDCl3 {V:\Bruker\TOPSPIN} mjp 7

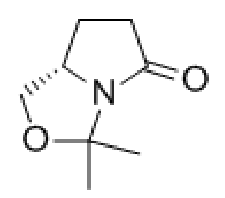

27

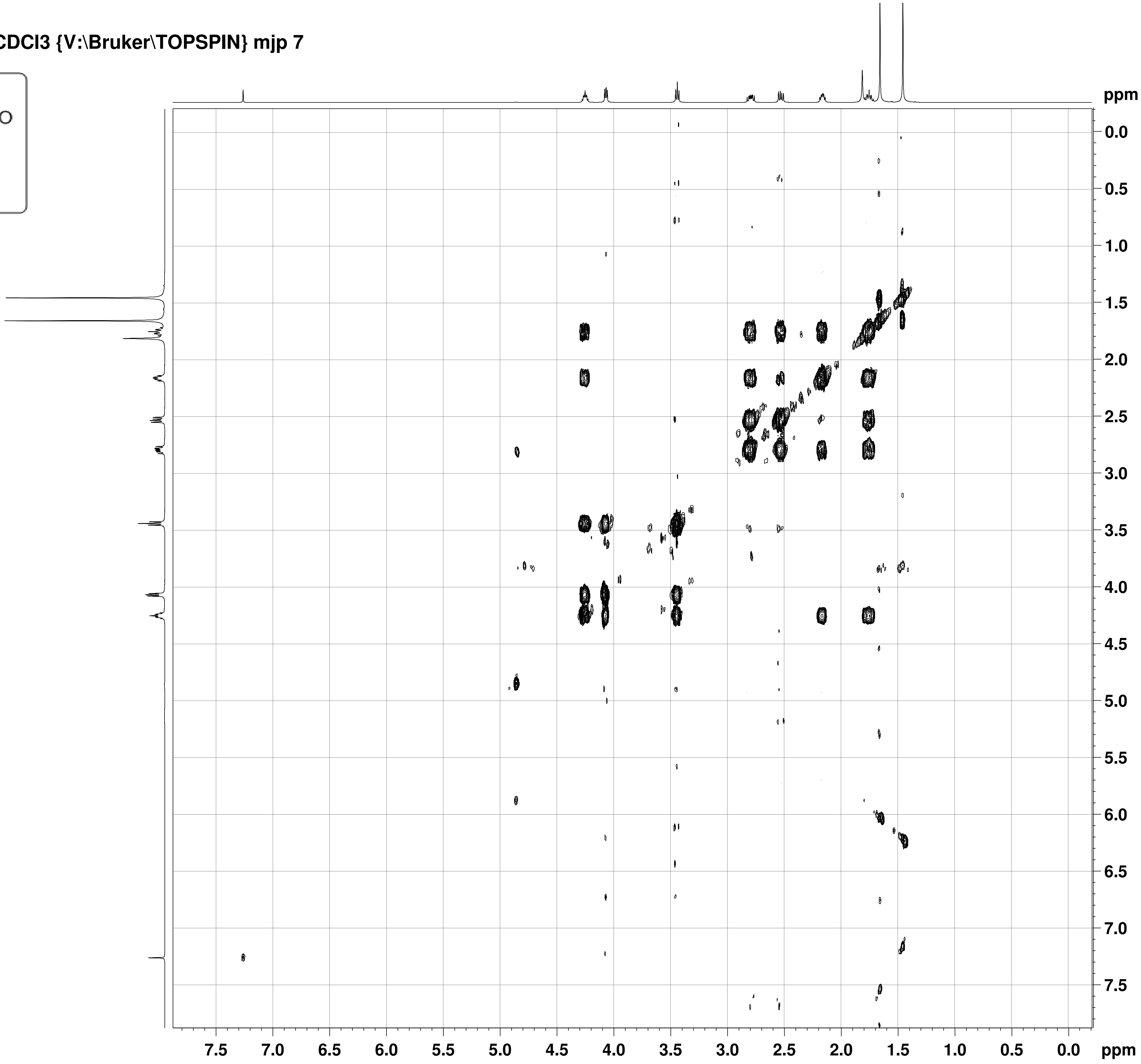


| | |
|---|---|
| NAME | JC-578-2 |
| EXPNO | 11 |
| PROCNO | 1 |
| Date_ | 20110817 |
| Time | 10.51 |
| INSTRUM | AV600 |
| PROBHD | 5 mm CPDCH 13C |
| PULPROG | cosygpmfqf |
| TD | 2048 |
| SOLVENT | CDCl3 |
| NS | 1 |
| DS | 8 |
| SWH | 4854.369 Hz |
| FIDRES | 2.370297 Hz |
| AQ | 0.2109940 sec |
| RG | 2050 |
| DW | 103.000 usec |
| DE | 6.50 usec |
| TE | 298.0 K |
| D0 | 0.00000300 sec |
| D1 | 1.68911302 sec |
| D13 | 0.00000400 sec |
| D16 | 0.00020000 sec |
| IN0 | 0.00020600 sec |

======== CHANNEL f1 ========

| | |
|---|---|
| NUC1 | 1H |
| P1 | 11.40 usec |
| PL1 | 1.00 dB |
| PL1W | 13.76731014 W |
| SFO1 | 600.1323128 MHz |

====== GRADIENT CHANNEL ====

| | |
|---|---|
| GPNAM1 | SINE.100 |
| GPNAM2 | SINE.100 |
| GPNAM3 | SINE.100 |
| GPZ1 | 16.00 % |
| GPZ2 | 12.00 % |
| GPZ3 | 40.00 % |
| P16 | 1000.00 usec |
| ND0 | 1 |
| TD | 128 |
| SFO1 | 600.1323 MHz |
| FIDRES | 37.924759 Hz |
| SW | 8.089 ppm |
| FnMODE | QF |
| SI | 1024 |
| SF | 600.1300094 MHz |
| WDW | QSINE |
| SSB | 0 |
| LB | 0.00 Hz |
| GB | 0 |
| PC | 1.40 |
| SI | 1024 |
| MC2 | QF |
| SF | 600.1300094 MHz |
| WDW | QSINE |
| SSB | 0 |
| LB | 0.00 Hz |
| GB | 0 |

JC–578–2
C13DEPT135.ucl CDCl3 {V:\Bruker\TOPSPIN} mjp 7

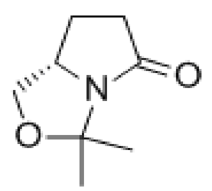

27

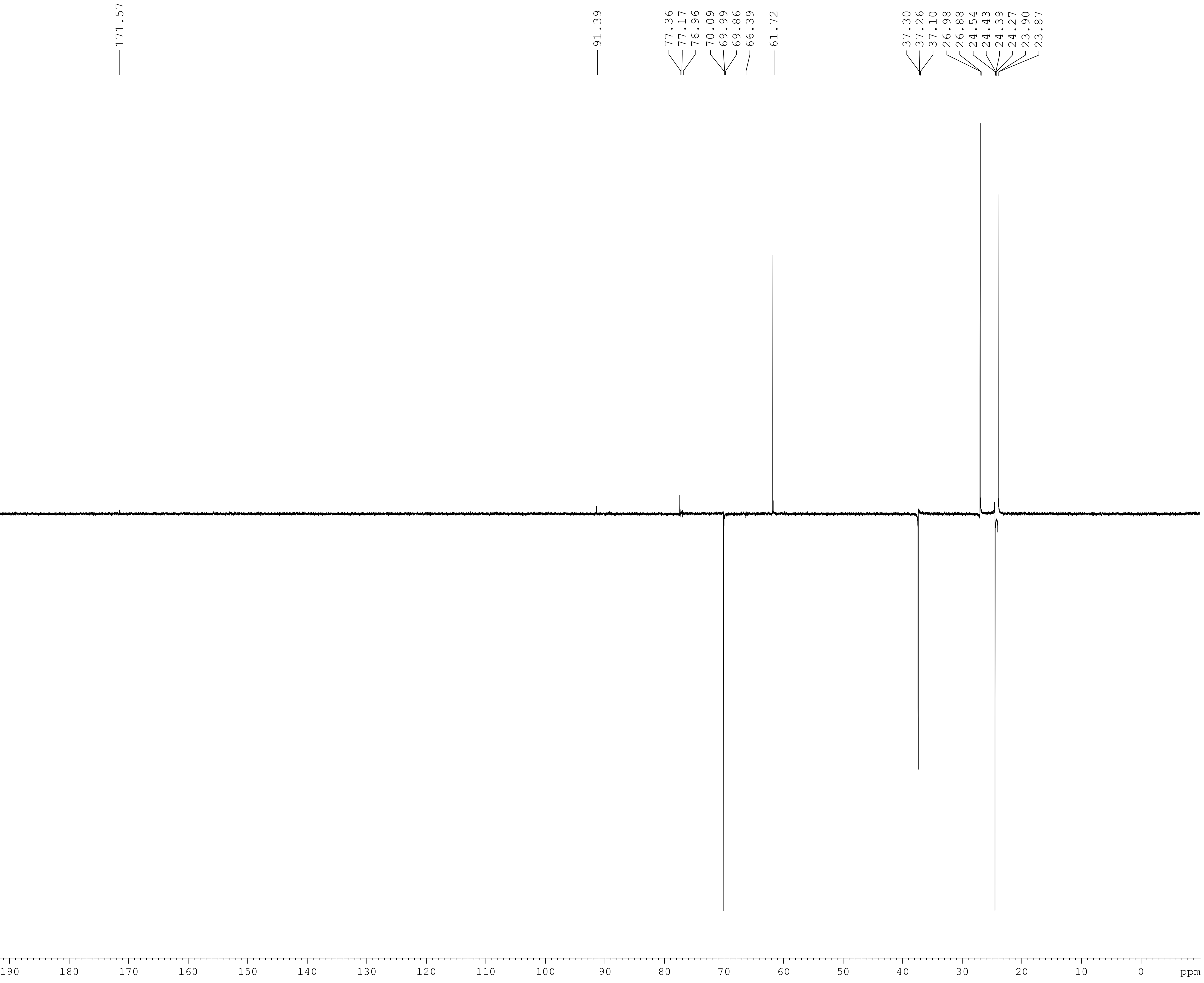


UCL

| | |
|---|---|
| NAME | JC-578-2 |
| EXPNO | 15 |
| PROCNO | 1 |
| Date_ | 20110817 |
| Time | 11.25 |
| INSTRUM | AV600 |
| PROBHD | 5 mm CPDCH 13C |
| PULPROG | dept135 |
| TD | 70308 |
| SOLVENT | CDCl3 |
| NS | 64 |
| DS | 4 |
| SWH | 39062.500 H |
| FIDRES | 0.555591 H |
| AQ | 0.8999924 s |
| RG | 256 |
| DW | 12.800 u |
| DE | 6.50 u |
| TE | 298.0 K |
| CNST2 | 145.0000000 |
| D1 | 2.00000000 s |
| D2 | 0.00344828 s |
| D12 | 0.00002000 s |
| TD0 | 1 |
| ======== CHANNEL f1 ===== | |
| NUC1 | 13C |
| P1 | 9.80 u |
| P2 | 19.60 u |
| PL1 | 5.00 d |
| PL1W | 26.76886177 W |
| SFO1 | 150.9201628 M |
| ======== CHANNEL f2 ===== | |
| CPDPRG2 | waltz16 |
| NUC2 | 1H |
| P3 | 10.80 u |
| P4 | 21.60 u |
| PCPD2 | 70.00 u |
| PL2 | 1.00 d |
| PL12 | 17.23 d |
| PL2W | 13.76731014 W |
| PL12W | 0.32798135 W |
| SFO2 | 600.1324005 M |
| SI | 65536 |
| SF | 150.9027930 M |
| WDW | EM |
| SSB | 0 |
| LB | 1.00 H |
| GB | 0 |
| PC | 1.40 |

**JC–578–2**
**HSQC.ucl CDCl3 {V:\Bruker\TOPSPIN} mjp 7**

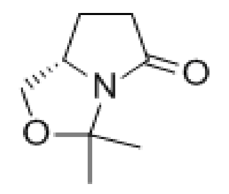

27

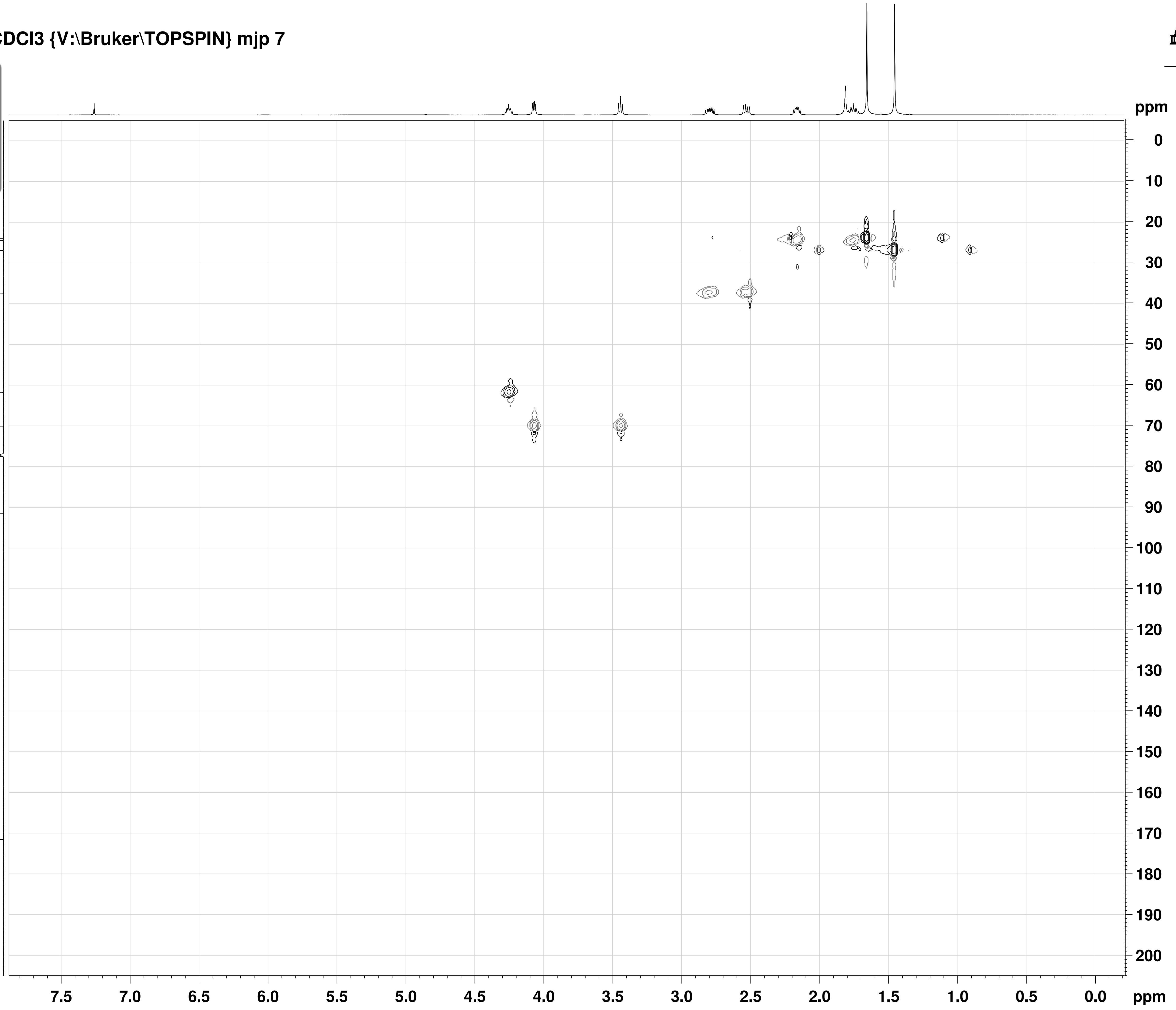

| | |
|---|---|
| NAME | JC-578-2 |
| EXPNO | 13 |
| PROCNO | 1 |
| Date_ | 20110817 |
| Time | 11.03 |
| INSTRUM | AV600 |
| PROBHD | 5 mm CPDCH 13C |
| PULPROG | hsqcedetgpsisp2.4 |
| TD | 1024 |
| SOLVENT | CDCl3 |
| NS | 2 |
| DS | 32 |
| SWH | 4854.369 Hz |
| FIDRES | 4.740595 Hz |
| AQ | 0.1055220 sec |
| RG | 2050 |
| DW | 103.000 usec |
| DE | 6.50 usec |
| TE | 298.0 K |
| CNST2 | 145.0000000 |
| CNST17 | -0.5000000 |
| D0 | 0.00000300 sec |
| D1 | 1.47972500 sec |
| D2 | 0.00344828 sec |
| D4 | 0.00172414 sec |
| D11 | 0.03000000 sec |
| D16 | 0.00020000 sec |
| D21 | 0.00344828 sec |
| D24 | 0.00086207 sec |
| IN0 | 0.00001580 sec |
| L0 | 0 |
| L31 | 1 |
| TD0 | 2 |
| ======== CHANNEL f1 ======== | |
| NUC1 | 1H |
| P1 | 11.40 usec |
| P2 | 22.80 usec |
| P28 | 0.00 usec |
| PL1 | 1.00 dB |
| PL1W | 13.76731014 W |
| SFO1 | 600.1323128 MHz |
| ======== CHANNEL f2 ======== | |
| CPDPRG2 | bi_p5m4sp_4sp.2 |
| NUC2 | 13C |
| P3 | 9.80 usec |
| P14 | 500.00 usec |
| P24 | 2000.00 usec |
| P31 | 1730.00 usec |
| P63 | 1500.00 usec |
| PL0 | 120.00 dB |
| PL2 | 5.00 dB |
| PL12 | 20.74 dB |
| PL0W | 0.00000000 W |
| PL2W | 26.76886177 W |
| PL12W | 0.71388775 W |
| SFO2 | 150.9178993 MHz |
| SP3 | 13.33 dB |
| SP7 | 13.33 dB |
| SP14 | 14.82 dB |
| SP18 | 18.73 dB |
| SP31 | 20.84 dB |
| SPNAM3 | Crp60,0.5,20.1 |
| SPNAM7 | Crp60comp.4 |
| SPNAM14 | Crp32,1.5,20.2 |
| SPNAM18 | Crp60_xfilt.2 |
| SPNAM31 | Crp32,1.5,20.2 |
| SPOAL3 | 0.500 |
| SPOAL7 | 0.500 |
| SPOAL14 | 0.500 |
| SPOAL18 | 0.500 |
| SPOAL31 | 0.500 |
| SPOFFS3 | 0.00 Hz |
| SPOFFS7 | 0.00 Hz |
| SPOFFS14 | 0.00 Hz |
| SPOFFS18 | 0.00 Hz |
| SPOFFS31 | 0.00 Hz |
| ====== GRADIENT CHANNEL ===== | |
| GPNAM1 | SINE.100 |
| GPNAM2 | SINE.100 |
| GPNAM3 | SINE.100 |
| GPNAM4 | SINE.100 |
| GPZ1 | 80.00 % |
| GPZ2 | 20.10 % |
| GPZ3 | 11.00 % |
| GPZ4 | -5.00 % |
| P16 | 1000.00 usec |
| P19 | 600.00 usec |
| ND0 | 2 |
| TD | 128 |
| SFO1 | 150.9179 MHz |
| FIDRES | 247.599686 Hz |
| SW | 210.000 ppm |
| FnMODE | Echo-Antiecho |
| SI | 1024 |
| SF | 600.1300094 MHz |
| WDW | QSINE |
| SSB | 2 |
| LB | 0.00 Hz |
| GB | 0 |
| PC | 1.40 |
| SI | 1024 |
| MC2 | echo-antiecho |
| SF | 150.9027778 MHz |
| WDW | QSINE |
| SSB | 2 |
| LB | 0.00 Hz |
| GB | 0 |

JC–578–2
HMBC.ucl CDCl3 {V:\Bruker\TOPSPIN} mjp 7

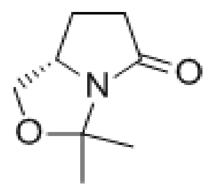


27

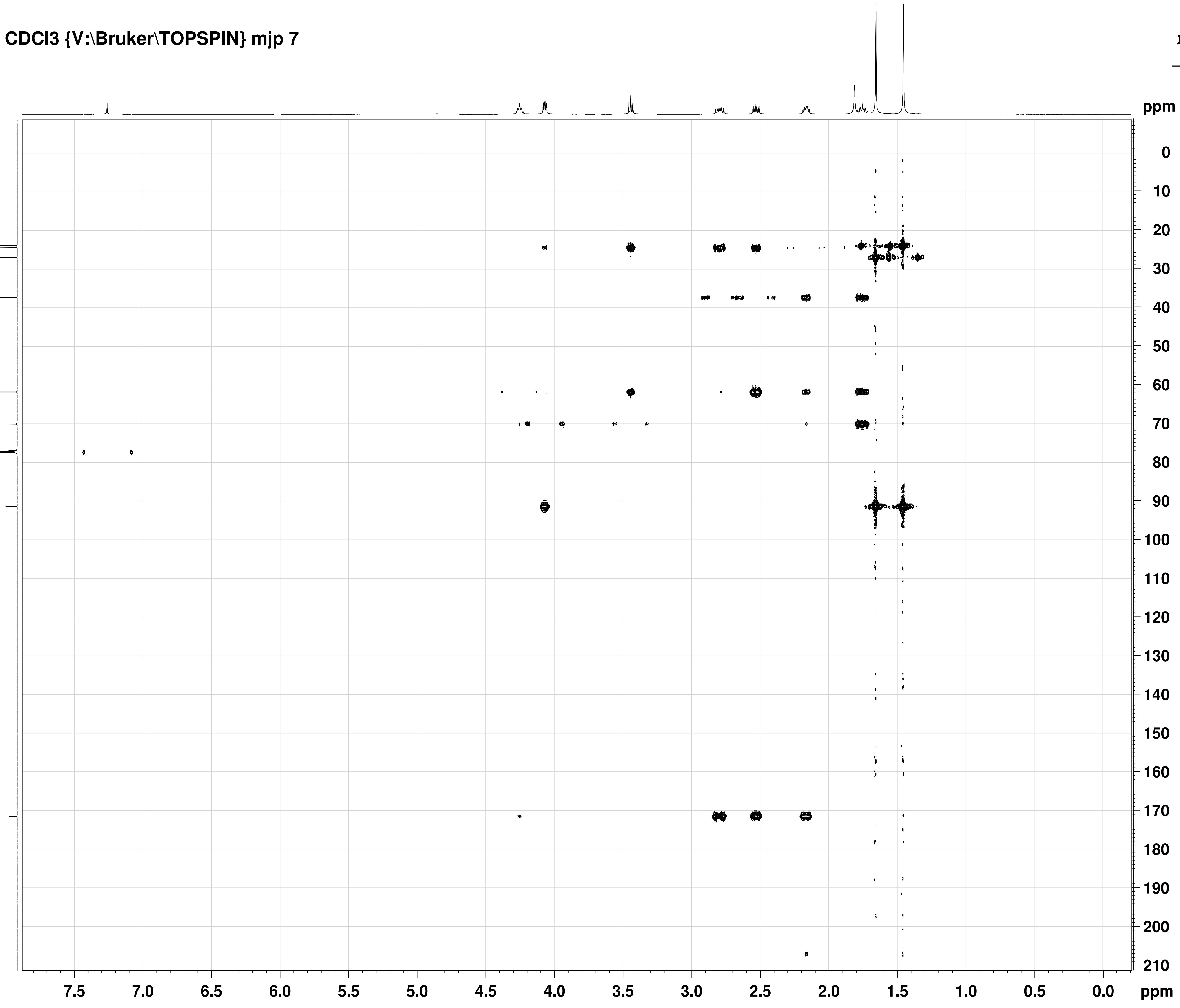


| | | |
|---|---|---|
| NAME | JC-578-2 | |
| EXPNO | 14 | |
| PROCNO | 1 | |
| Date_ | 20110817 | |
| Time | 11.11 | |
| INSTRUM | AV600 | |
| PROBHD | 5 mm CPDCH 13C | |
| PULPROG | hmbcetgpl3nd | |
| TD | 4096 | |
| SOLVENT | CDCl3 | |
| NS | 2 | |
| DS | 16 | |
| SWH | 4854.369 | Hz |
| FIDRES | 1.185149 | Hz |
| AQ | 0.4219380 | sec |
| RG | 2050 | |
| DW | 103.000 | usec |
| DE | 6.50 | usec |
| TE | 298.0 | K |
| CNST6 | 120.0000000 | |
| CNST7 | 160.0000000 | |
| CNST13 | 10.0000000 | |
| CNST30 | 0.5981150 | |
| D0 | 0.00000300 | sec |
| D1 | 0.75590658 | sec |
| D6 | 0.05000000 | sec |
| D16 | 0.00020000 | sec |
| IN0 | 0.00001505 | sec |
| ======== CHANNEL f1 ======== | | |
| NUC1 | 1H | |
| P1 | 11.40 | usec |
| P2 | 22.80 | usec |
| PL1 | 1.00 | dB |
| PL1W | 13.76731014 | W |
| SFO1 | 600.1323128 | MHz |
| ======== CHANNEL f2 ======== | | |
| NUC2 | 13C | |
| P3 | 9.80 | usec |
| P24 | 2000.00 | usec |
| PL2 | 5.00 | dB |
| PL2W | 26.76886177 | W |
| SFO2 | 150.9178993 | MHz |
| SP7 | 13.33 | dB |
| SPNAM7 | Crp60comp.4 | |
| SPOAL7 | 0.500 | |
| SPOFFS7 | 0.00 | Hz |
| ====== GRADIENT CHANNEL ===== | | |
| GPNAM1 | SINE.100 | |
| GPNAM3 | SINE.100 | |
| GPNAM4 | SINE.100 | |
| GPNAM5 | SINE.100 | |
| GPNAM6 | SINE.100 | |
| GPZ1 | 80.00 | % |
| GPZ3 | 14.00 | % |
| GPZ4 | -8.00 | % |
| GPZ5 | -4.00 | % |
| GPZ6 | -2.00 | % |
| P16 | 1000.00 | usec |
| ND0 | 2 | |
| TD | 256 | |
| SFO1 | 150.9179 | MHz |
| FIDRES | 129.695068 | Hz |
| SW | 220.000 | ppm |
| FnMODE | Echo-Antiecho | |
| SI | 2048 | |
| SF | 600.1300106 | MHz |
| WDW | SINE | |
| SSB | 2 | |
| LB | 0.00 | Hz |
| GB | 0 | |
| PC | 1.40 | |
| SI | 1024 | |
| MC2 | echo-antiecho | |
| SF | 150.9027771 | MHz |
| WDW | SINE | |
| SSB | 2 | |
| LB | 0.00 | Hz |
| GB | 0 | |

JC–579–3
PROTON.ucl CDCl3 {V:\Bruker\TOPSPIN} mjp 40

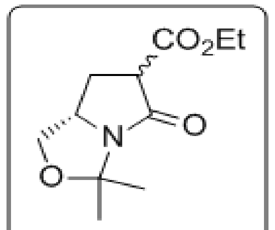


28

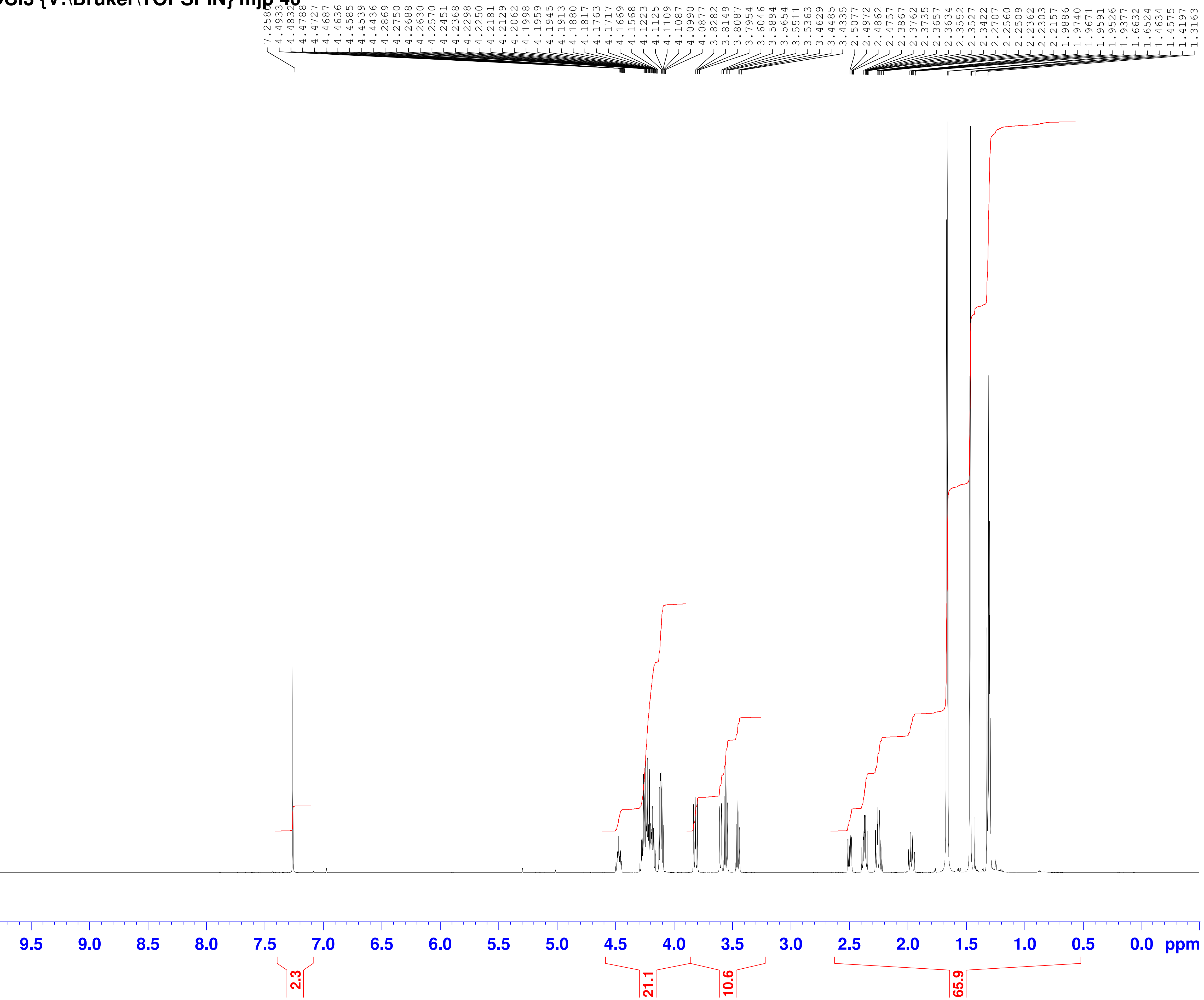


UCL

| | | |
|---|---|---|
| NAME | JC-579-3 | |
| EXPNO | 10 | |
| PROCNO | 1 | |
| Date_ | 20110822 | |
| Time | 10.49 | |
| INSTRUM | AV600 | |
| PROBHD | 5 mm CPDCH 13C | |
| PULPROG | zg30 | |
| TD | 98682 | |
| SOLVENT | CDCl3 | |
| NS | 8 | |
| DS | 0 | |
| SWH | 12335.526 | Hz |
| FIDRES | 0.125003 | Hz |
| AQ | 3.9999604 | sec |
| RG | 28.5 | |
| DW | 40.533 | use |
| DE | 10.48 | use |
| TE | 298.0 | K |
| D1 | 1.00000000 | sec |
| TD0 | 1 | |

======== CHANNEL f1 ========

| | | |
|---|---|---|
| NUC1 | 1H | |
| P1 | 11.40 | use |
| PL1 | 1.00 | dB |
| PL1W | 13.76731014 | W |
| SFO1 | 600.1337061 | MHz |
| SI | 32768 | |
| SF | 600.1300116 | MHz |
| WDW | EM | |
| SSB | 0 | |
| LB | 0.30 | Hz |
| GB | 0 | |
| PC | 1.40 | |

**JC–579–3**
**C13CPD.ucl CDCl3 {V:\Bruker\TOPSPIN} mjp 40**

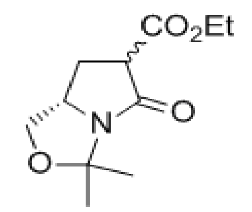


**28**

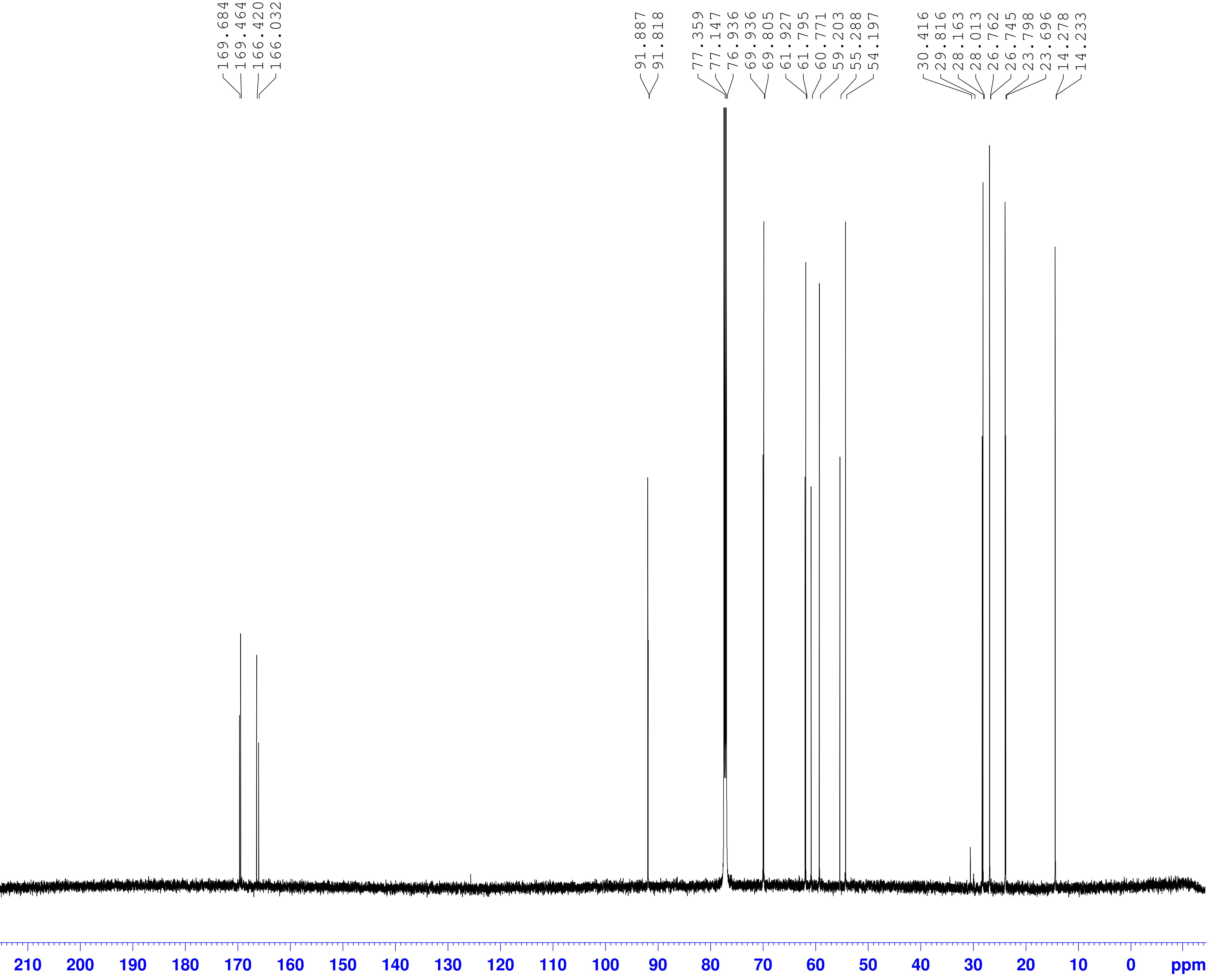


UCL

```
NAME              JC-579-3
EXPNO                   12
PROCNO                   1
Date_             20110822
Time                 11.01
INSTRUM              AV600
PROBHD   5 mm CPDCH 13C
PULPROG            zgpg30
TD                   70308
SOLVENT              CDCl3
NS                     128
DS                       0
SWH            39062.500 H
FIDRES          0.555591 H
AQ             0.8999924 s
RG                    1030
DW                12.800 u
DE                 21.12 u
TE                 298.0 K
D1            2.00000000 s
D11           0.03000000 s
TD0                      1

======== CHANNEL f1 =====
NUC1                   13C
P1                  9.80 u
PL1                 5.00 d
PL1W         26.76886177 W
SFO1         150.9201628 M

======== CHANNEL f2 =====
CPDPRG2            waltz16
NUC2                    1H
PCPD2              70.00 u
PL2                 1.00 d
PL12               17.23 d
PL13               20.00 d
PL2W         13.76731014 W
PL12W         0.32798135 W
PL13W         0.17332016 W
SFO2         600.1324005 M
SI                   65536
SF           150.9027930 M
WDW                     EM
SSB                      0
LB                  1.00 H
GB                       0
PC                    1.40
```

JC–579–3
COSY.ucl CDCl3 {V:\Bruker\TOPSPIN} mjp 40

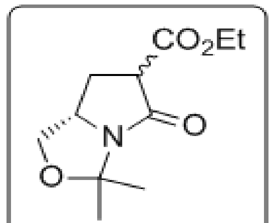


28

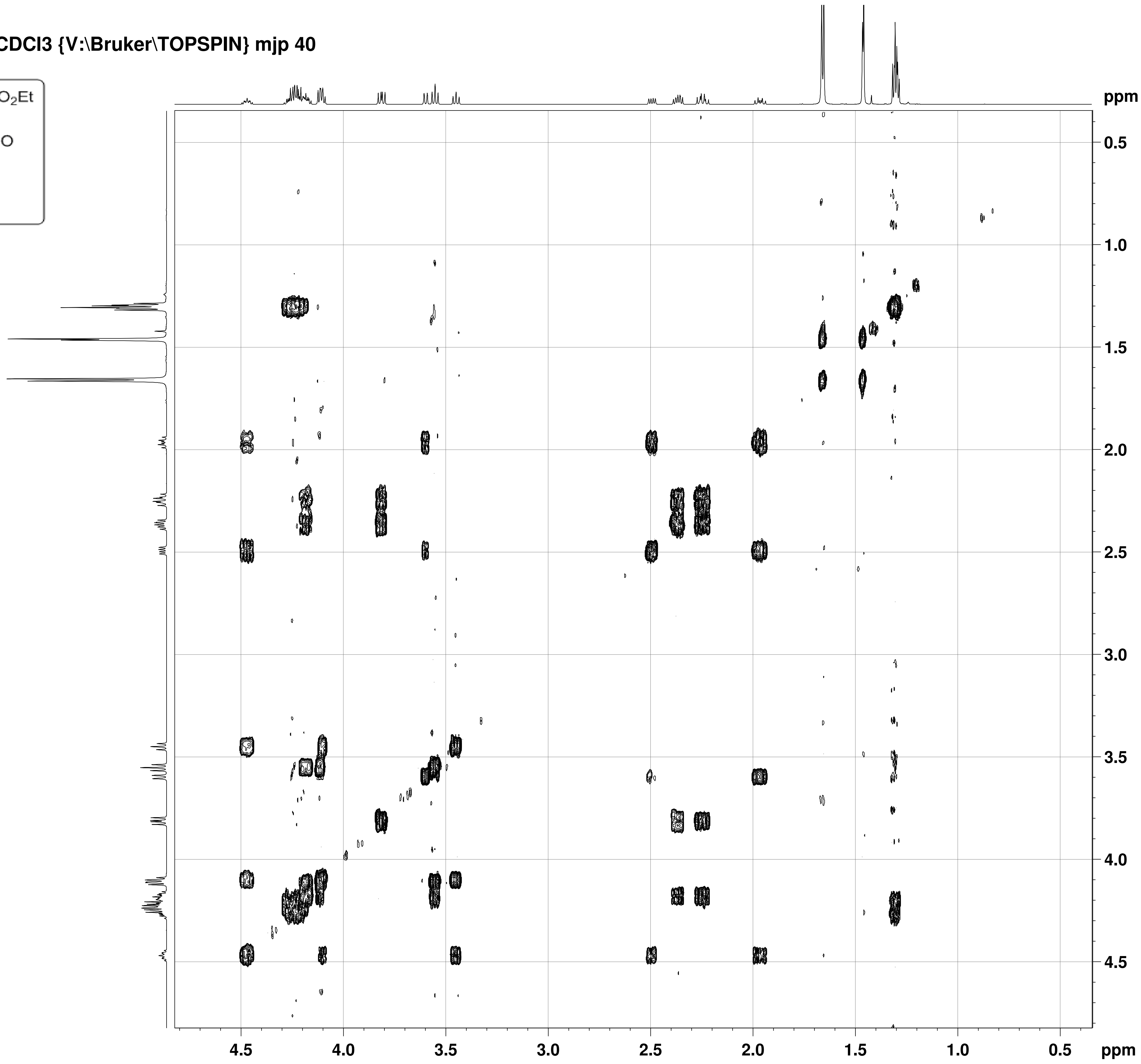


| | |
|---|---|
| NAME | JC-579-3 |
| EXPNO | 11 |
| PROCNO | 1 |
| Date_ | 20110822 |
| Time | 10.50 |
| INSTRUM | AV600 |
| PROBHD | 5 mm CPDCH 13C |
| PULPROG | cosygpmfqf |
| TD | 2048 |
| SOLVENT | CDCl3 |
| NS | 1 |
| DS | 8 |
| SWH | 2688.172 Hz |
| FIDRES | 1.312584 Hz |
| AQ | 0.3809780 sec |
| RG | 2050 |
| DW | 186.000 usec |
| DE | 6.50 usec |
| TE | 298.0 K |
| D0 | 0.00000300 sec |
| D1 | 1.51912904 sec |
| D13 | 0.00000400 sec |
| D16 | 0.00020000 sec |
| IN0 | 0.00037200 sec |
| ======== CHANNEL f1 ======== | |
| NUC1 | 1H |
| P1 | 11.40 usec |
| PL1 | 1.00 dB |
| PL1W | 13.76731014 W |
| SFO1 | 600.1315621 MHz |
| ====== GRADIENT CHANNEL ==== | |
| GPNAM1 | SINE.100 |
| GPNAM2 | SINE.100 |
| GPNAM3 | SINE.100 |
| GPZ1 | 16.00 % |
| GPZ2 | 12.00 % |
| GPZ3 | 40.00 % |
| P16 | 1000.00 usec |
| ND0 | 1 |
| TD | 128 |
| SFO1 | 600.1316 MHz |
| FIDRES | 21.001345 Hz |
| SW | 4.479 ppm |
| FnMODE | QF |
| SI | 1024 |
| SF | 600.1300105 MHz |
| WDW | QSINE |
| SSB | 0 |
| LB | 0.00 Hz |
| GB | 0 |
| PC | 1.40 |
| SI | 1024 |
| MC2 | QF |
| SF | 600.1300105 MHz |
| WDW | QSINE |
| SSB | 0 |
| LB | 0.00 Hz |
| GB | 0 |

**JC–579–3**
**C13DEPT135.ucl CDCl3 {V:\Bruker\TOPSPIN} mjp 40**

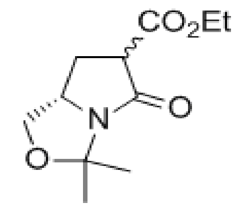


**28**

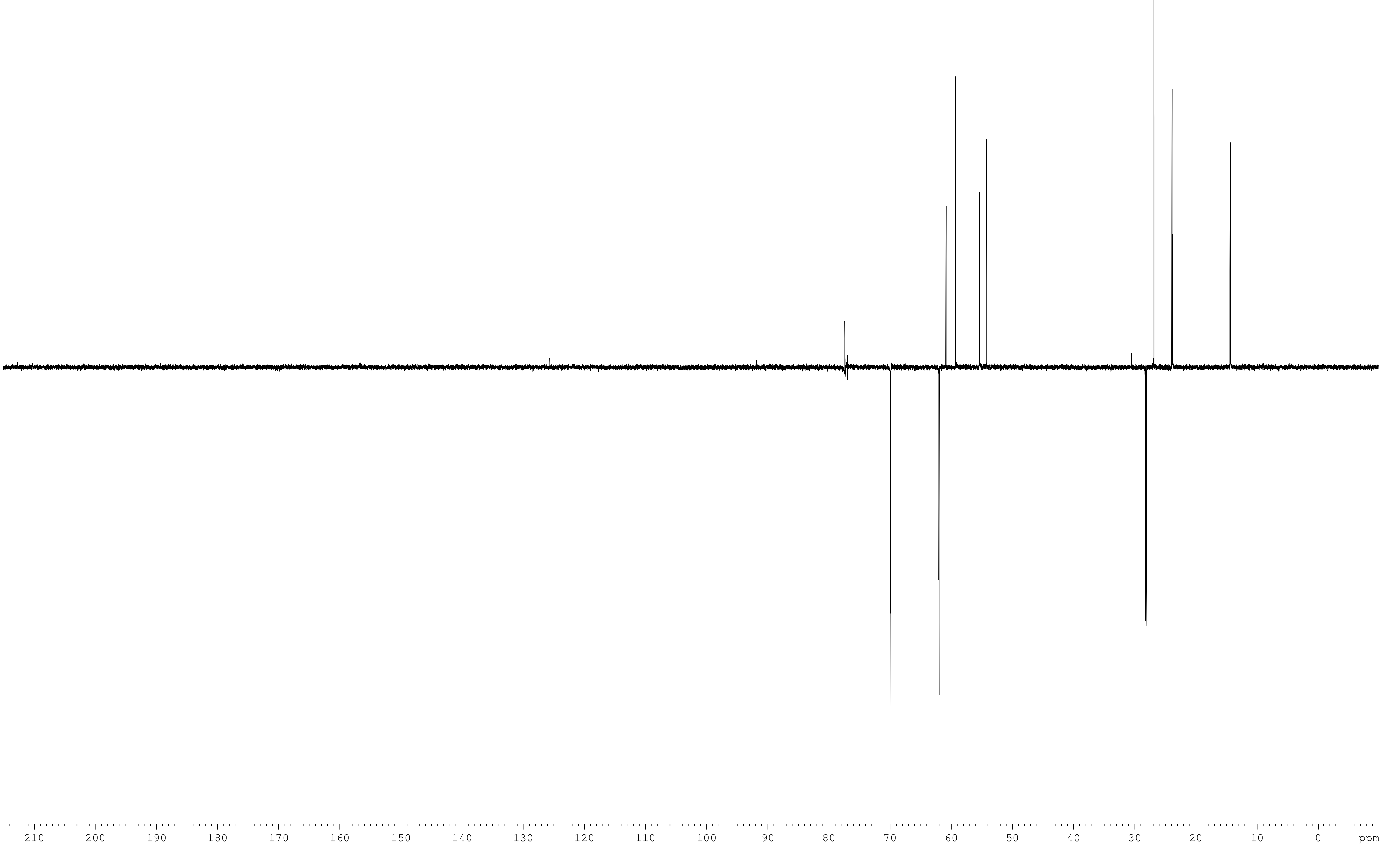


```
NAME          JC-579-3
EXPNO               15
PROCNO               1
Date_         20110822
Time             11.24
INSTRUM          AV600
PROBHD   5 mm CPDCH 13C
PULPROG        dept135
TD               70308
SOLVENT          CDCl3
NS                  64
DS                   4
SWH          39062.500 Hz
FIDRES        0.555591 Hz
AQ           0.8999924 sec
RG                 256
DW              12.800 usec
DE                6.50 usec
TE               298.0 K
CNST2      145.0000000
D1          2.00000000 sec
D2          0.00344828 sec
D12         0.00002000 sec
TD0                  1

======== CHANNEL f1 =====
NUC1               13C
P1                9.80 usec
P2               19.60 usec
PL1               5.00 dB
PL1W       26.76886177 W
SFO1       150.9201628 MHz

======== CHANNEL f2 =====
CPDPRG2        waltz16
NUC2                1H
P3               10.80 usec
P4               21.60 usec
PCPD2            70.00 usec
PL2               1.00 dB
PL12             17.23 dB
PL2W       13.76731014 W
PL12W       0.32798135 W
SFO2       600.1324005 MHz
SI               65536
SF         150.9027930 MHz
WDW                 EM
SSB                  0
LB                1.00 Hz
GB                   0
PC                1.40
```

JC–579–3
HSQC.ucl CDCl3 {V:\Bruker\TOPSPIN} mjp 40

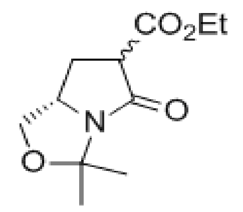


28

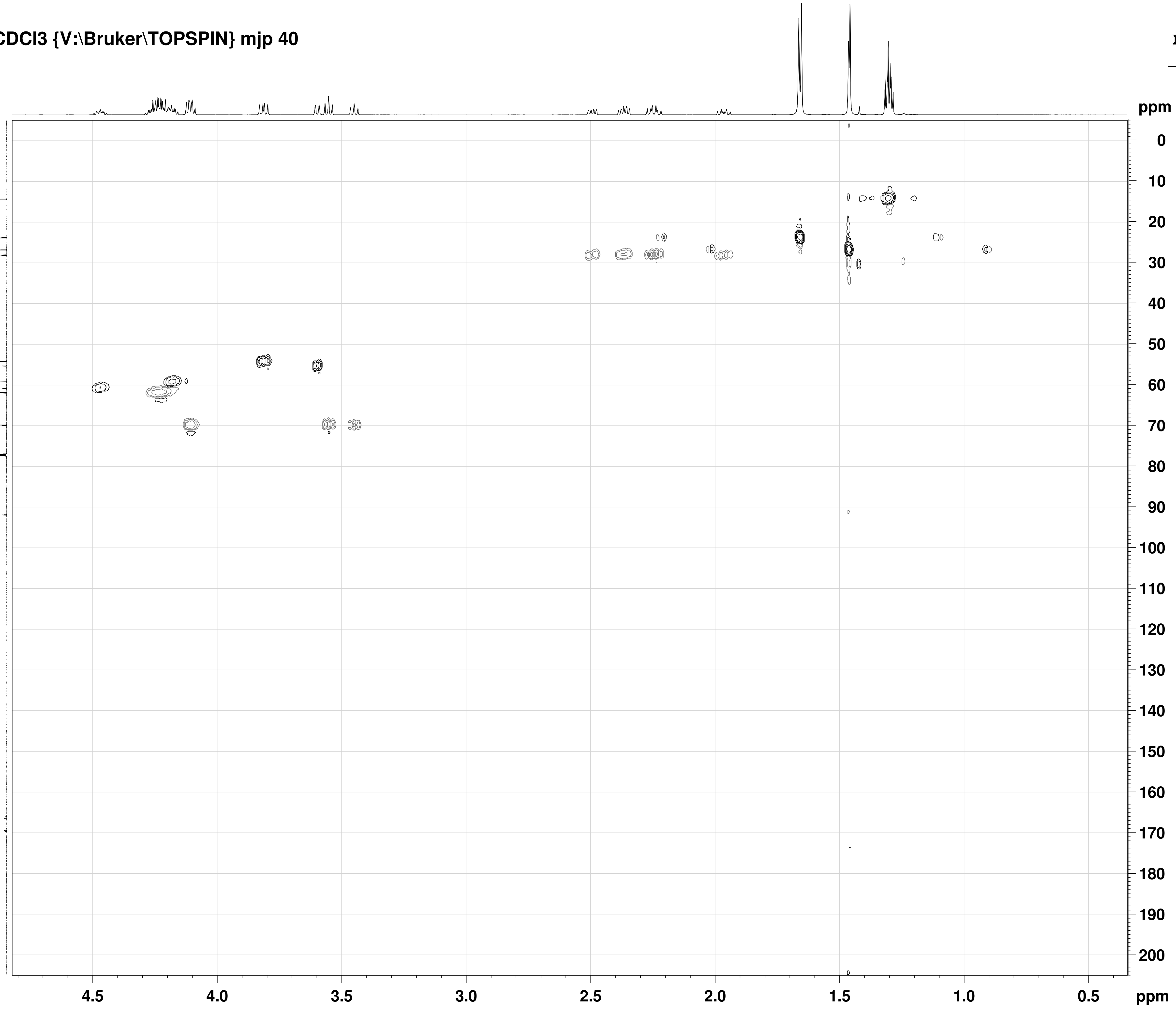


```
NAME        JC-579-3
EXPNO             13
PROCNO             1
Date_       20110822
Time           11.02
INSTRUM        AV600
PROBHD   5 mm CPDCH 13C
PULPROG  hsqcedetgpsisp2.4
TD              1024
SOLVENT         CDCl3
NS                 2
DS                32
SWH         2688.172 Hz
FIDRES      2.625168 Hz
AQ         0.1905140 sec
RG              2050
DW           186.000 usec
DE              6.50 usec
TE             298.0 K
CNST2    145.0000000
CNST17    -0.5000000
D0        0.00000300 sec
D1        1.39473295 sec
D2        0.00344828 sec
D4        0.00172414 sec
D11       0.03000000 sec
D16       0.00020000 sec
D21       0.00344828 sec
D24       0.00086207 sec
IN0       0.00001580 sec
L0                 0
L31                1
TD0                2

======== CHANNEL f1 ========
NUC1              1H
P1             11.40 usec
P2             22.80 usec
P28             0.00 usec
PL1             1.00 dB
PL1W     13.76731014 W
SFO1     600.1315621 MHz

======== CHANNEL f2 ========
CPDPRG2  bi_p5m4sp_4sp.2
NUC2             13C
P3              9.80 usec
P14           500.00 usec
P24          2000.00 usec
P31          1730.00 usec
P63          1500.00 usec
PL0           120.00 dB
PL2             5.00 dB
PL12           20.74 dB
PL0W      0.00000000 W
PL2W     26.76886177 W
PL12W     0.71388775 W
SFO2     150.9178993 MHz
SP3            13.33 dB
SP7            13.33 dB
SP14           14.82 dB
SP18           18.73 dB
SP31           20.84 dB
SPNAM3   Crp60,0.5,20.1
SPNAM7     Crp60comp.4
SPNAM14  Crp32,1.5,20.2
SPNAM18   Crp60_xfilt.2
SPNAM31  Crp32,1.5,20.2
SPOAL3         0.500
SPOAL7         0.500
SPOAL14        0.500
SPOAL18        0.500
SPOAL31        0.500
SPOFFS3         0.00 Hz
SPOFFS7         0.00 Hz
SPOFFS14        0.00 Hz
SPOFFS18        0.00 Hz
SPOFFS31        0.00 Hz

====== GRADIENT CHANNEL =====
GPNAM1      SINE.100
GPNAM2      SINE.100
GPNAM3      SINE.100
GPNAM4      SINE.100
GPZ1           80.00 %
GPZ2           20.10 %
GPZ3           11.00 %
GPZ4           -5.00 %
P16          1000.00 usec
P19           600.00 usec
ND0                2
TD               128
SFO1        150.9179 MHz
FIDRES    247.599686 Hz
SW           210.000 ppm
FnMODE   Echo-Antiecho
SI              1024
SF       600.1300105 MHz
WDW            QSINE
SSB                2
LB              0.00 Hz
GB                 0
PC              1.40
SI              1024
MC2     echo-antiecho
SF       150.9027778 MHz
WDW            QSINE
SSB                2
LB              0.00 Hz
GB                 0
```

**JC–579–3**
**HMBC.ucl CDCl3 {V:\Bruker\TOPSPIN} mjp 40**

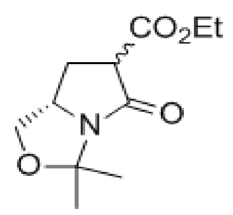


**28**

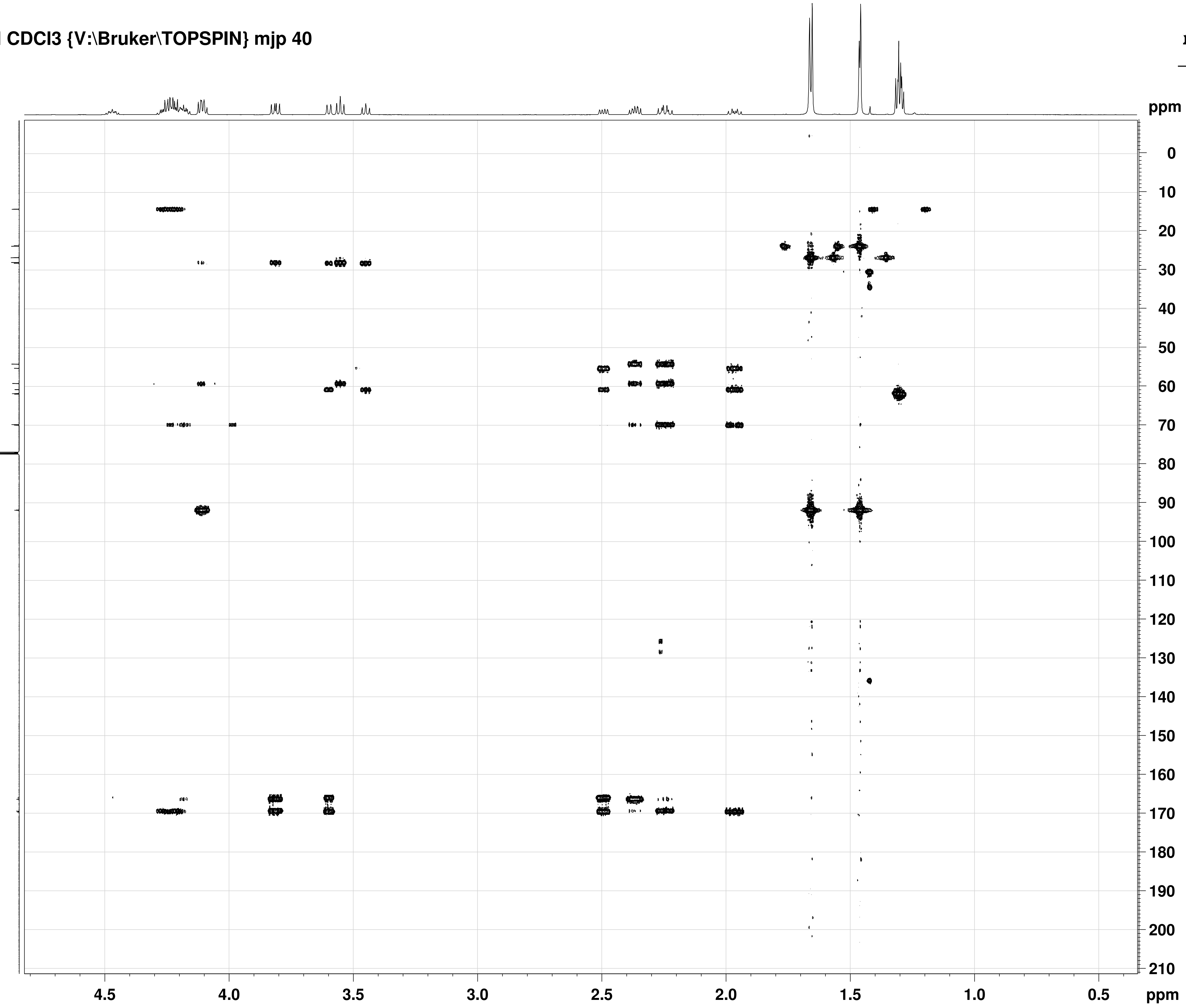


| | |
|---|---|
| NAME | JC-579-3 |
| EXPNO | 14 |
| PROCNO | 1 |
| Date_ | 20110822 |
| Time | 11.10 |
| INSTRUM | AV600 |
| PROBHD | 5 mm CPDCH 13C |
| PULPROG | hmbcetgpl3nd |
| TD | 4096 |
| SOLVENT | CDCl3 |
| NS | 2 |
| DS | 16 |
| SWH | 2688.172 Hz |
| FIDRES | 0.656292 Hz |
| AQ | 0.7619060 sec |
| RG | 2050 |
| DW | 186.000 usec |
| DE | 6.50 usec |
| TE | 298.0 K |
| CNST6 | 120.0000000 |
| CNST7 | 160.0000000 |
| CNST13 | 10.0000000 |
| CNST30 | 0.5981146 |
| D0 | 0.00000300 sec |
| D1 | 0.41593859 sec |
| D6 | 0.05000000 sec |
| D16 | 0.00020000 sec |
| IN0 | 0.00001505 sec |
| ======== CHANNEL f1 ======== | |
| NUC1 | 1H |
| P1 | 11.40 usec |
| P2 | 22.80 usec |
| PL1 | 1.00 dB |
| PL1W | 13.76731014 W |
| SFO1 | 600.1315621 MHz |
| ======== CHANNEL f2 ======== | |
| NUC2 | 13C |
| P3 | 9.80 usec |
| P24 | 2000.00 usec |
| PL2 | 5.00 dB |
| PL2W | 26.76886177 W |
| SFO2 | 150.9178993 MHz |
| SP7 | 13.33 dB |
| SPNAM7 | Crp60comp.4 |
| SPOAL7 | 0.500 |
| SPOFFS7 | 0.00 Hz |
| ====== GRADIENT CHANNEL ===== | |
| GPNAM1 | SINE.100 |
| GPNAM3 | SINE.100 |
| GPNAM4 | SINE.100 |
| GPNAM5 | SINE.100 |
| GPNAM6 | SINE.100 |
| GPZ1 | 80.00 % |
| GPZ3 | 14.00 % |
| GPZ4 | -8.00 % |
| GPZ5 | -4.00 % |
| GPZ6 | -2.00 % |
| P16 | 1000.00 usec |
| ND0 | 2 |
| TD | 256 |
| SFO1 | 150.9179 MHz |
| FIDRES | 129.695068 Hz |
| SW | 220.000 ppm |
| FnMODE | Echo-Antiecho |
| SI | 2048 |
| SF | 600.1300111 MHz |
| WDW | SINE |
| SSB | 2 |
| LB | 0.00 Hz |
| GB | 0 |
| PC | 1.40 |
| SI | 1024 |
| MC2 | echo-antiecho |
| SF | 150.9027771 MHz |
| WDW | SINE |
| SSB | 2 |
| LB | 0.00 Hz |
| GB | 0 |

**JC–595–2**
**PROTON.ucl CDCl3 {V:\Bruker\TOPSPIN} mjp 39**

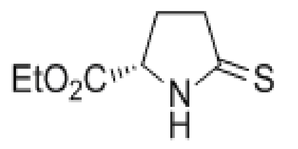


**30a**

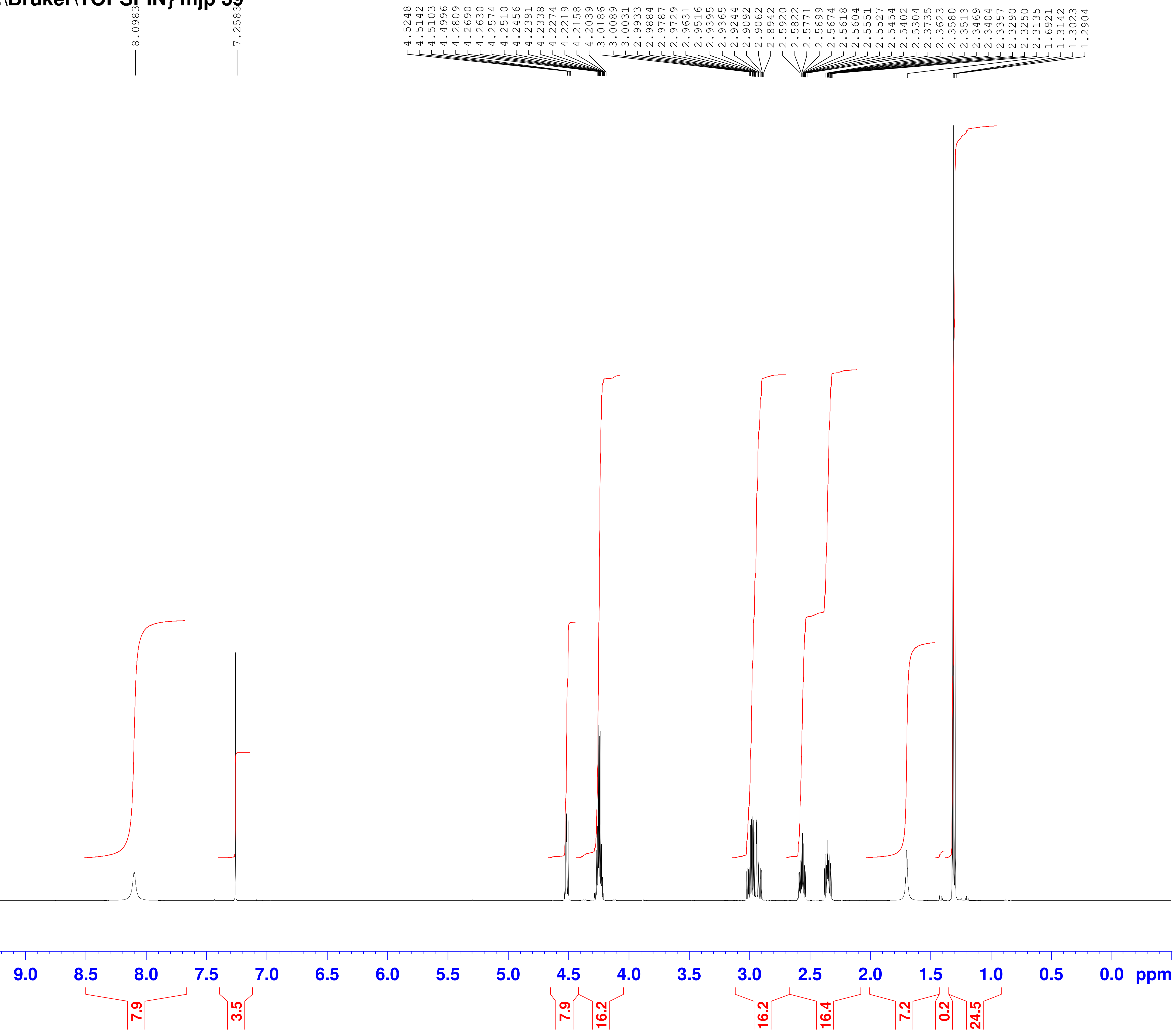


| | |
|---|---|
| NAME | JC-595-2 |
| EXPNO | 10 |
| PROCNO | 1 |
| Date_ | 20110902 |
| Time | 14.15 |
| INSTRUM | AV600 |
| PROBHD | 5 mm CPDCH 13C |
| PULPROG | zg30 |
| TD | 98682 |
| SOLVENT | CDCl3 |
| NS | 8 |
| DS | 0 |
| SWH | 12335.526 Hz |
| FIDRES | 0.125003 Hz |
| AQ | 3.9999604 sec |
| RG | 40.3 |
| DW | 40.533 use |
| DE | 10.48 use |
| TE | 298.0 K |
| D1 | 1.00000000 sec |
| TD0 | 1 |

======== CHANNEL f1 ========

| | |
|---|---|
| NUC1 | 1H |
| P1 | 11.40 use |
| PL1 | 1.00 dB |
| PL1W | 13.76731014 W |
| SFO1 | 600.1337061 MHz |
| SI | 32768 |
| SF | 600.1300116 MHz |
| WDW | EM |
| SSB | 0 |
| LB | 0.30 Hz |
| GB | 0 |
| PC | 1.40 |

**JC–595–2**
**C13CPD.ucl CDCl3 {V:\Bruker\TOPSPIN} mjp 39**

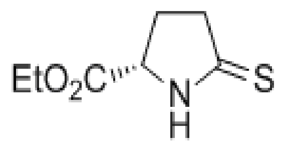

**30a**

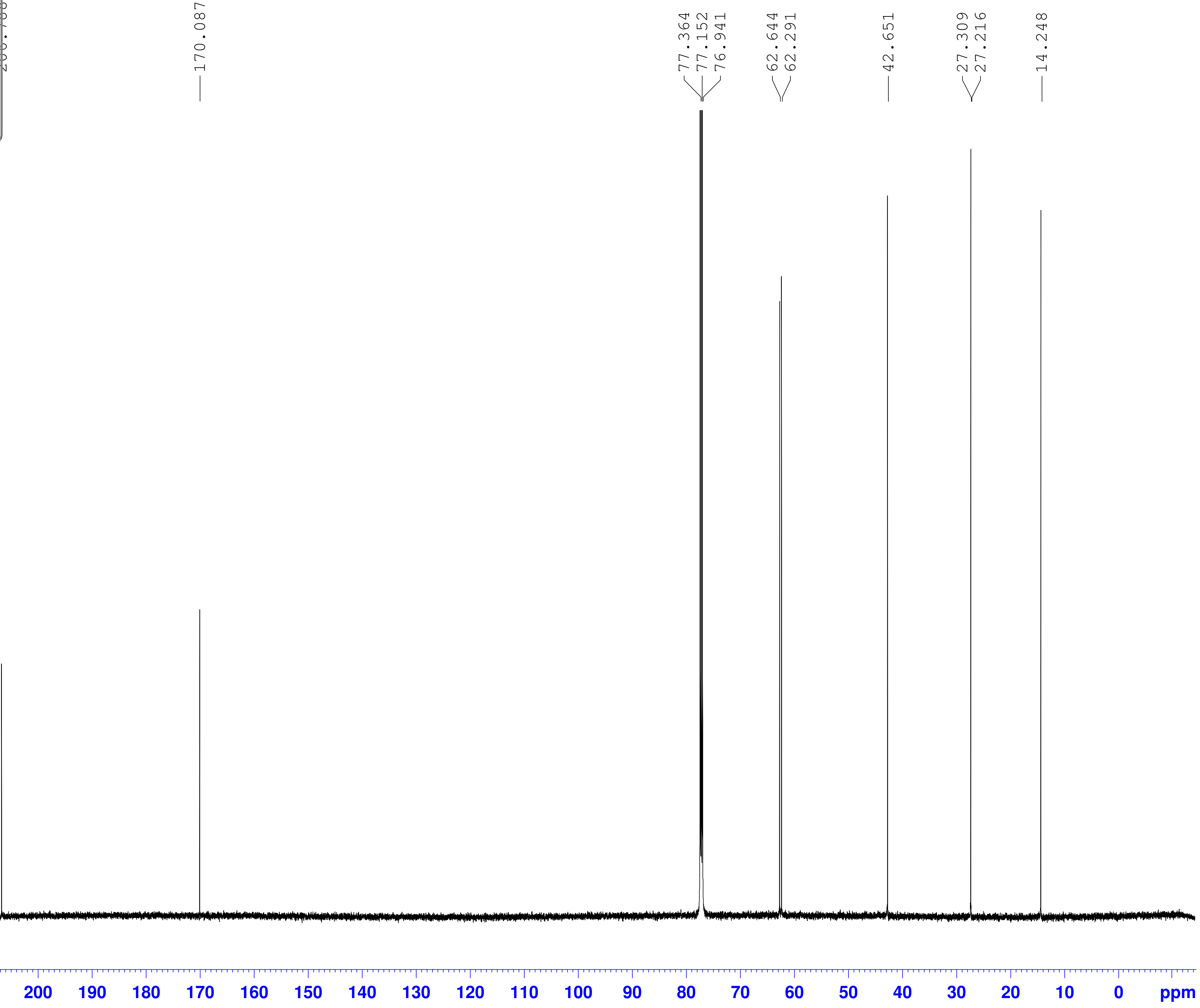


```
NAME            JC-595-2
EXPNO                 12
PROCNO                 1
Date_           20110902
Time               18.22
INSTRUM            AV600
PROBHD   5 mm CPDCH 13C
PULPROG          zgpg30
TD                 70308
SOLVENT            CDCl3
NS                   128
DS                     0
SWH           39062.500 H
FIDRES         0.555591 H
AQ            0.8999924 s
RG                  1030
DW               12.800 u
DE                21.12 u
TE                298.0 K
D1           2.00000000 s
D11          0.03000000 s
TD0                    1

======== CHANNEL f1 =====
NUC1                 13C
P1                  9.80 u
PL1                 5.00 d
PL1W         26.76886177 W
SFO1         150.9201628 M

======== CHANNEL f2 =====
CPDPRG2          waltz16
NUC2                  1H
PCPD2              70.00 u
PL2                 1.00 d
PL12               17.23 d
PL13               20.00 d
PL2W         13.76731014 W
PL12W         0.32798135 W
PL13W         0.17332016 W
SFO2         600.1324005 M
SI                 65536
SF           150.9027930 M
WDW                   EM
SSB                    0
LB                  1.00 H
GB                     0
PC                  1.40
```

JC–595–2
COSY.ucl CDCl3 {V:\Bruker\TOPSPIN} mjp 39

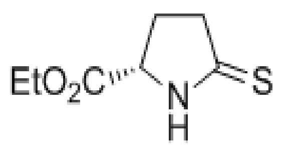

30a

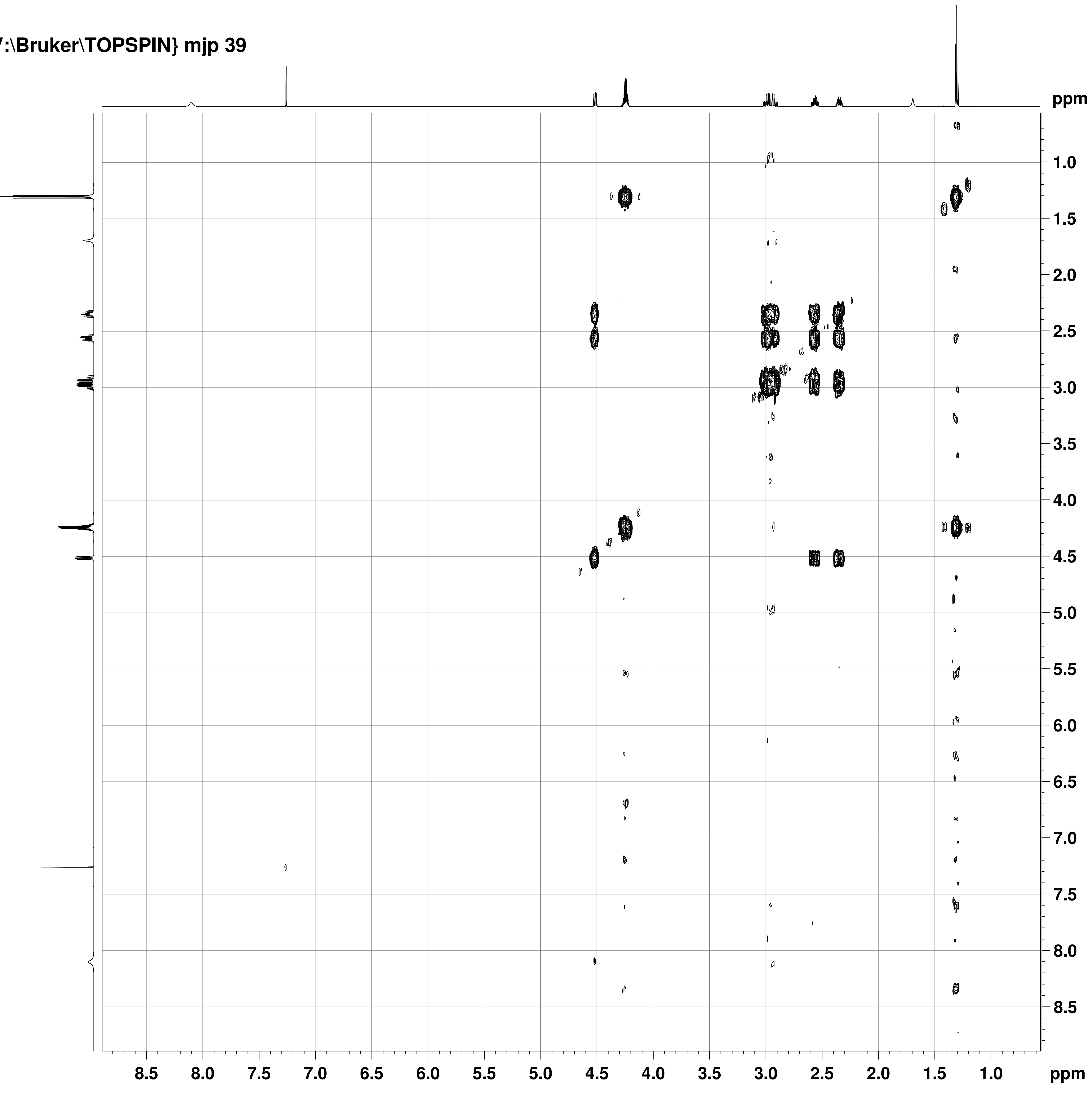


| | |
|---|---|
| NAME | JC-595-2 |
| EXPNO | 11 |
| PROCNO | 1 |
| Date_ | 20110902 |
| Time | 14.16 |
| INSTRUM | AV600 |
| PROBHD | 5 mm CPDCH 13C |
| PULPROG | cosygpmfqf |
| TD | 2048 |
| SOLVENT | CDCl3 |
| NS | 1 |
| DS | 8 |
| SWH | 5000.000 Hz |
| FIDRES | 2.441406 Hz |
| AQ | 0.2048500 sec |
| RG | 2050 |
| DW | 100.000 usec |
| DE | 6.50 usec |
| TE | 298.0 K |
| D0 | 0.00000300 sec |
| D1 | 1.69525695 sec |
| D13 | 0.00000400 sec |
| D16 | 0.00020000 sec |
| IN0 | 0.00020000 sec |

======== CHANNEL f1 ========

| | |
|---|---|
| NUC1 | 1H |
| P1 | 11.40 usec |
| PL1 | 1.00 dB |
| PL1W | 13.76731014 W |
| SFO1 | 600.1328483 MHz |

====== GRADIENT CHANNEL =====

| | |
|---|---|
| GPNAM1 | SINE.100 |
| GPNAM2 | SINE.100 |
| GPNAM3 | SINE.100 |
| GPZ1 | 16.00 % |
| GPZ2 | 12.00 % |
| GPZ3 | 40.00 % |
| P16 | 1000.00 usec |
| ND0 | 1 |
| TD | 128 |
| SFO1 | 600.1328 MHz |
| FIDRES | 39.062500 Hz |
| SW | 8.331 ppm |
| FnMODE | QF |
| SI | 1024 |
| SF | 600.1300093 MHz |
| WDW | QSINE |
| SSB | 0 |
| LB | 0.00 Hz |
| GB | 0 |
| PC | 1.40 |
| SI | 1024 |
| MC2 | QF |
| SF | 600.1300093 MHz |
| WDW | QSINE |
| SSB | 0 |
| LB | 0.00 Hz |
| GB | 0 |

JC–595–2
HSQC.ucl CDCl3 {V:\Bruker\TOPSPIN} mjp 39

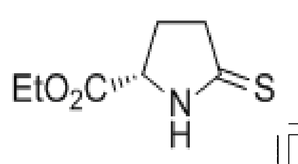

30a

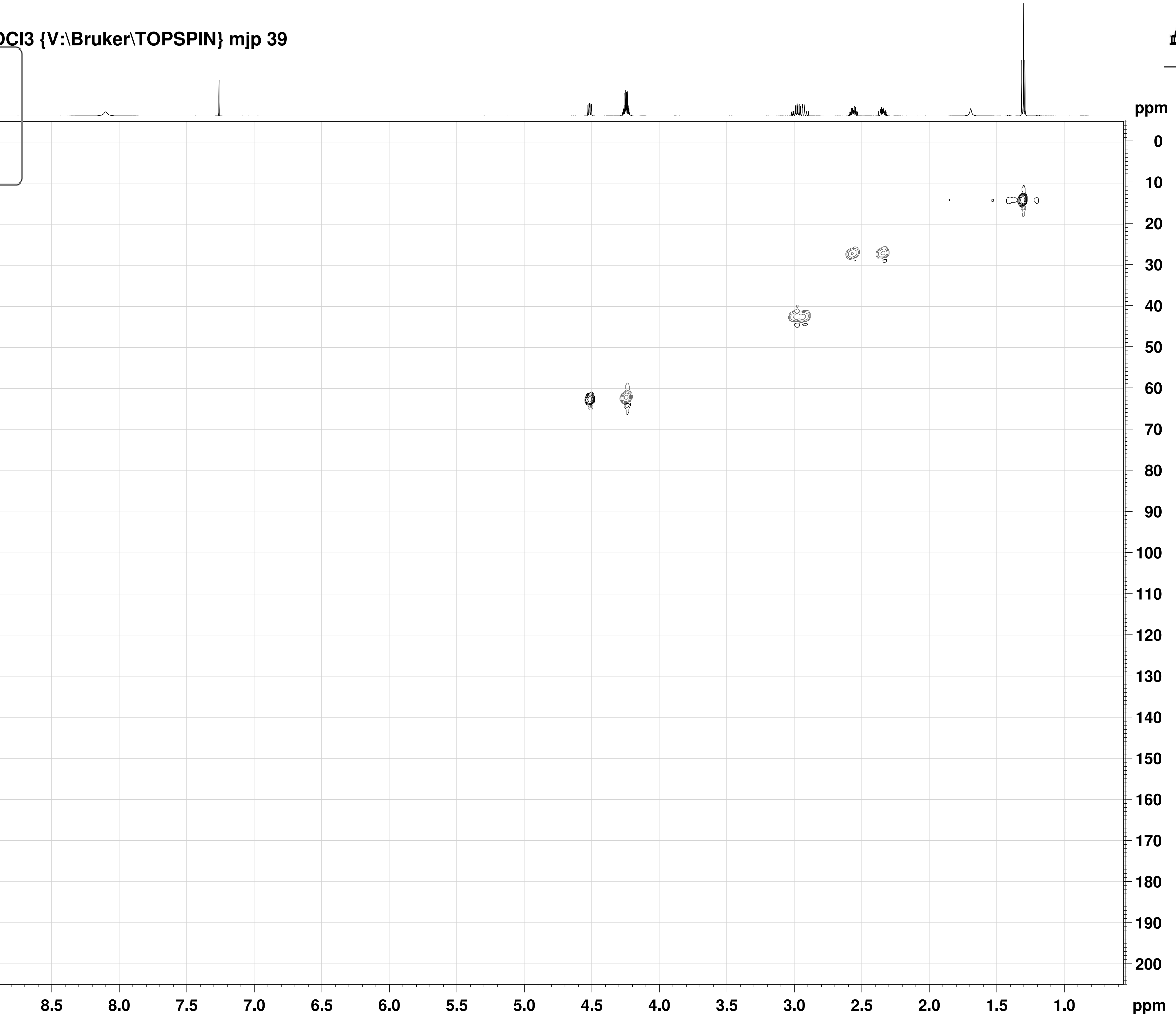


| | | |
|---|---|---|
| NAME | JC-595-2 | |
| EXPNO | 13 | |
| PROCNO | 1 | |
| Date_ | 20110902 | |
| Time | 18.23 | |
| INSTRUM | AV600 | |
| PROBHD | 5 mm CPDCH 13C | |
| PULPROG | hsqcedetgpsisp2.4 | |
| TD | 1024 | |
| SOLVENT | CDCl3 | |
| NS | 2 | |
| DS | 32 | |
| SWH | 5000.000 | Hz |
| FIDRES | 4.882813 | Hz |
| AQ | 0.1024500 | sec |
| RG | 2050 | |
| DW | 100.000 | usec |
| DE | 6.50 | usec |
| TE | 298.0 | K |
| CNST2 | 145.0000000 | |
| CNST17 | -0.5000000 | |
| D0 | 0.00000300 | sec |
| D1 | 1.48279703 | sec |
| D2 | 0.00344828 | sec |
| D4 | 0.00172414 | sec |
| D11 | 0.03000000 | sec |
| D16 | 0.00020000 | sec |
| D21 | 0.00344828 | sec |
| D24 | 0.00086207 | sec |
| IN0 | 0.00001580 | sec |
| L0 | 0 | |
| L31 | 1 | |
| TD0 | 2 | |
| ======== CHANNEL f1 ======== | | |
| NUC1 | 1H | |
| P1 | 11.40 | usec |
| P2 | 22.80 | usec |
| P28 | 0.00 | usec |
| PL1 | 1.00 | dB |
| PL1W | 13.76731014 | W |
| SFO1 | 600.1328483 | MHz |
| ======== CHANNEL f2 ======== | | |
| CPDPRG2 | bi_p5m4sp_4sp.2 | |
| NUC2 | 13C | |
| P3 | 9.80 | usec |
| P14 | 500.00 | usec |
| P24 | 2000.00 | usec |
| P31 | 1730.00 | usec |
| P63 | 1500.00 | usec |
| PL0 | 120.00 | dB |
| PL2 | 5.00 | dB |
| PL12 | 20.74 | dB |
| PL0W | 0.00000000 | W |
| PL2W | 26.76886177 | W |
| PL12W | 0.71388775 | W |
| SFO2 | 150.9178993 | MHz |
| SP3 | 13.33 | dB |
| SP7 | 13.33 | dB |
| SP14 | 14.82 | dB |
| SP18 | 18.73 | dB |
| SP31 | 20.84 | dB |
| SPNAM3 | Crp60,0.5,20.1 | |
| SPNAM7 | Crp60comp.4 | |
| SPNAM14 | Crp32,1.5,20.2 | |
| SPNAM18 | Crp60_xfilt.2 | |
| SPNAM31 | Crp32,1.5,20.2 | |
| SPOAL3 | 0.500 | |
| SPOAL7 | 0.500 | |
| SPOAL14 | 0.500 | |
| SPOAL18 | 0.500 | |
| SPOAL31 | 0.500 | |
| SPOFFS3 | 0.00 | Hz |
| SPOFFS7 | 0.00 | Hz |
| SPOFFS14 | 0.00 | Hz |
| SPOFFS18 | 0.00 | Hz |
| SPOFFS31 | 0.00 | Hz |
| ====== GRADIENT CHANNEL ===== | | |
| GPNAM1 | SINE.100 | |
| GPNAM2 | SINE.100 | |
| GPNAM3 | SINE.100 | |
| GPNAM4 | SINE.100 | |
| GPZ1 | 80.00 | % |
| GPZ2 | 20.10 | % |
| GPZ3 | 11.00 | % |
| GPZ4 | -5.00 | % |
| P16 | 1000.00 | usec |
| P19 | 600.00 | usec |
| ND0 | 2 | |
| TD | 128 | |
| SFO1 | 150.9179 | MHz |
| FIDRES | 247.599686 | Hz |
| SW | 210.000 | ppm |
| FnMODE | Echo-Antiecho | |
| SI | 1024 | |
| SF | 600.1300093 | MHz |
| WDW | QSINE | |
| SSB | 2 | |
| LB | 0.00 | Hz |
| GB | 0 | |
| PC | 1.40 | |
| SI | 1024 | |
| MC2 | echo-antiecho | |
| SF | 150.9027778 | MHz |
| WDW | QSINE | |
| SSB | 2 | |
| LB | 0.00 | Hz |
| GB | 0 | |

**JC–595–2**
**HMBC.ucl CDCl3 {V:\Bruker\TOPSPIN} mjp 39**

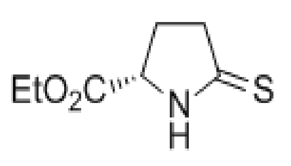


**30a**

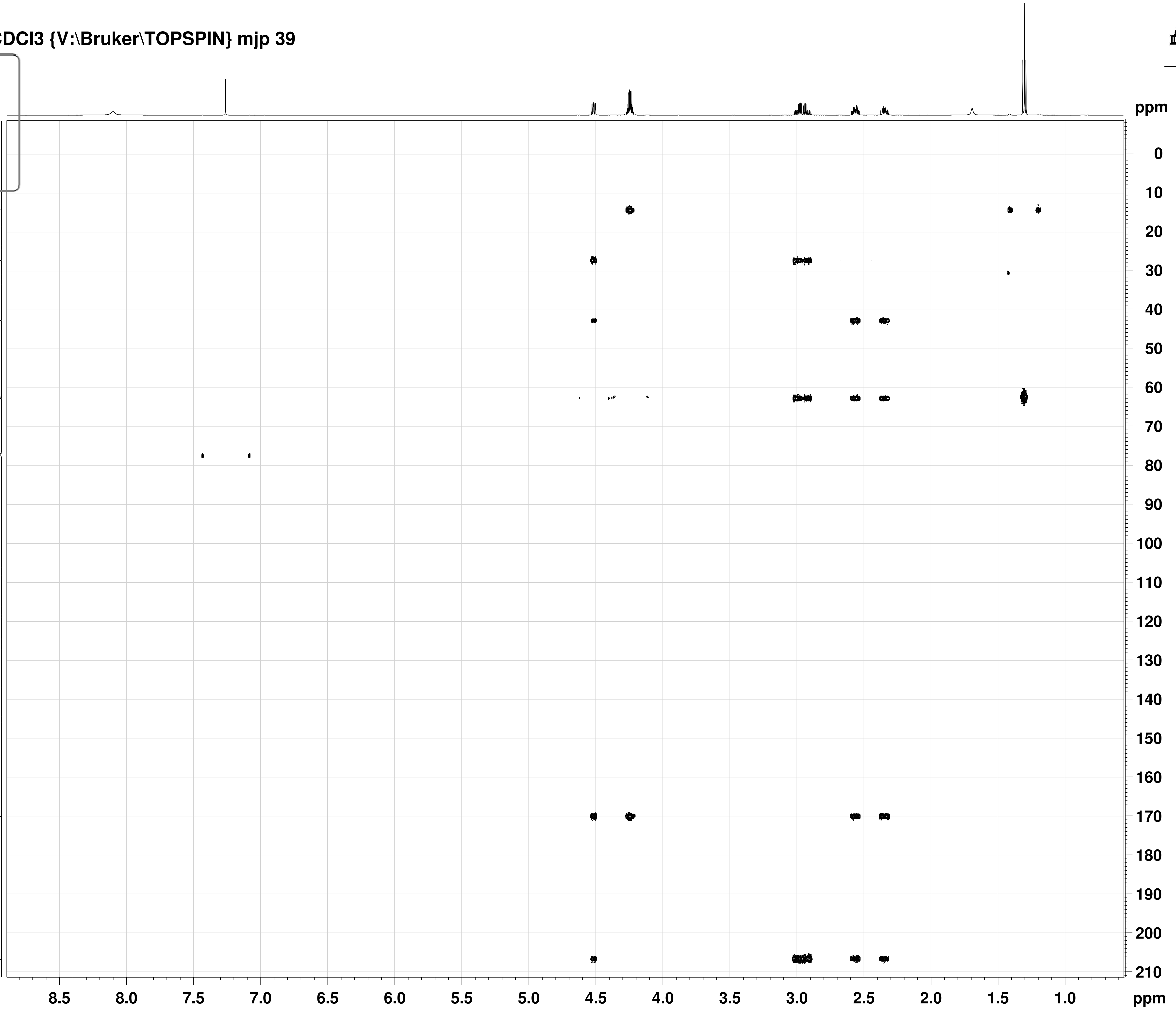


| | |
|---|---|
| NAME | JC-595-2 |
| EXPNO | 14 |
| PROCNO | 1 |
| Date_ | 20110902 |
| Time | 18.31 |
| INSTRUM | AV600 |
| PROBHD | 5 mm CPDCH 13C |
| PULPROG | hmbcetgpl3nd |
| TD | 4096 |
| SOLVENT | CDCl3 |
| NS | 2 |
| DS | 16 |
| SWH | 5000.000 Hz |
| FIDRES | 1.220703 Hz |
| AQ | 0.4096500 sec |
| RG | 2050 |
| DW | 100.000 usec |
| DE | 6.50 usec |
| TE | 298.0 K |
| CNST6 | 120.0000000 |
| CNST7 | 160.0000000 |
| CNST13 | 10.0000000 |
| CNST30 | 0.5981153 |
| D0 | 0.00000300 sec |
| D1 | 0.76819462 sec |
| D6 | 0.05000000 sec |
| D16 | 0.00020000 sec |
| IN0 | 0.00001505 sec |
| ======== CHANNEL f1 ======== | |
| NUC1 | 1H |
| P1 | 11.40 usec |
| P2 | 22.80 usec |
| PL1 | 1.00 dB |
| PL1W | 13.76731014 W |
| SFO1 | 600.1328483 MHz |
| ======== CHANNEL f2 ======== | |
| NUC2 | 13C |
| P3 | 9.80 usec |
| P24 | 2000.00 usec |
| PL2 | 5.00 dB |
| PL2W | 26.76886177 W |
| SFO2 | 150.9178993 MHz |
| SP7 | 13.33 dB |
| SPNAM7 | Crp60comp.4 |
| SPOAL7 | 0.500 |
| SPOFFS7 | 0.00 Hz |
| ====== GRADIENT CHANNEL ===== | |
| GPNAM1 | SINE.100 |
| GPNAM3 | SINE.100 |
| GPNAM4 | SINE.100 |
| GPNAM5 | SINE.100 |
| GPNAM6 | SINE.100 |
| GPZ1 | 80.00 % |
| GPZ3 | 14.00 % |
| GPZ4 | -8.00 % |
| GPZ5 | -4.00 % |
| GPZ6 | -2.00 % |
| P16 | 1000.00 usec |
| ND0 | 2 |
| TD | 256 |
| SFO1 | 150.9179 MHz |
| FIDRES | 129.695068 Hz |
| SW | 220.000 ppm |
| FnMODE | Echo-Antiecho |
| SI | 2048 |
| SF | 600.1300106 MHz |
| WDW | SINE |
| SSB | 2 |
| LB | 0.00 Hz |
| GB | 0 |
| PC | 1.40 |
| SI | 1024 |
| MC2 | echo-antiecho |
| SF | 150.9027771 MHz |
| WDW | SINE |
| SSB | 2 |
| LB | 0.00 Hz |
| GB | 0 |

**JC–597–2**
**PROTON.ucl DMSO {V:\Bruker\TOPSPIN} mjp 34**

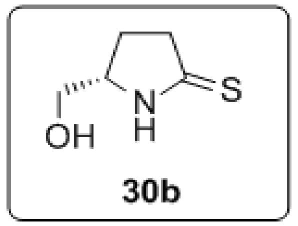


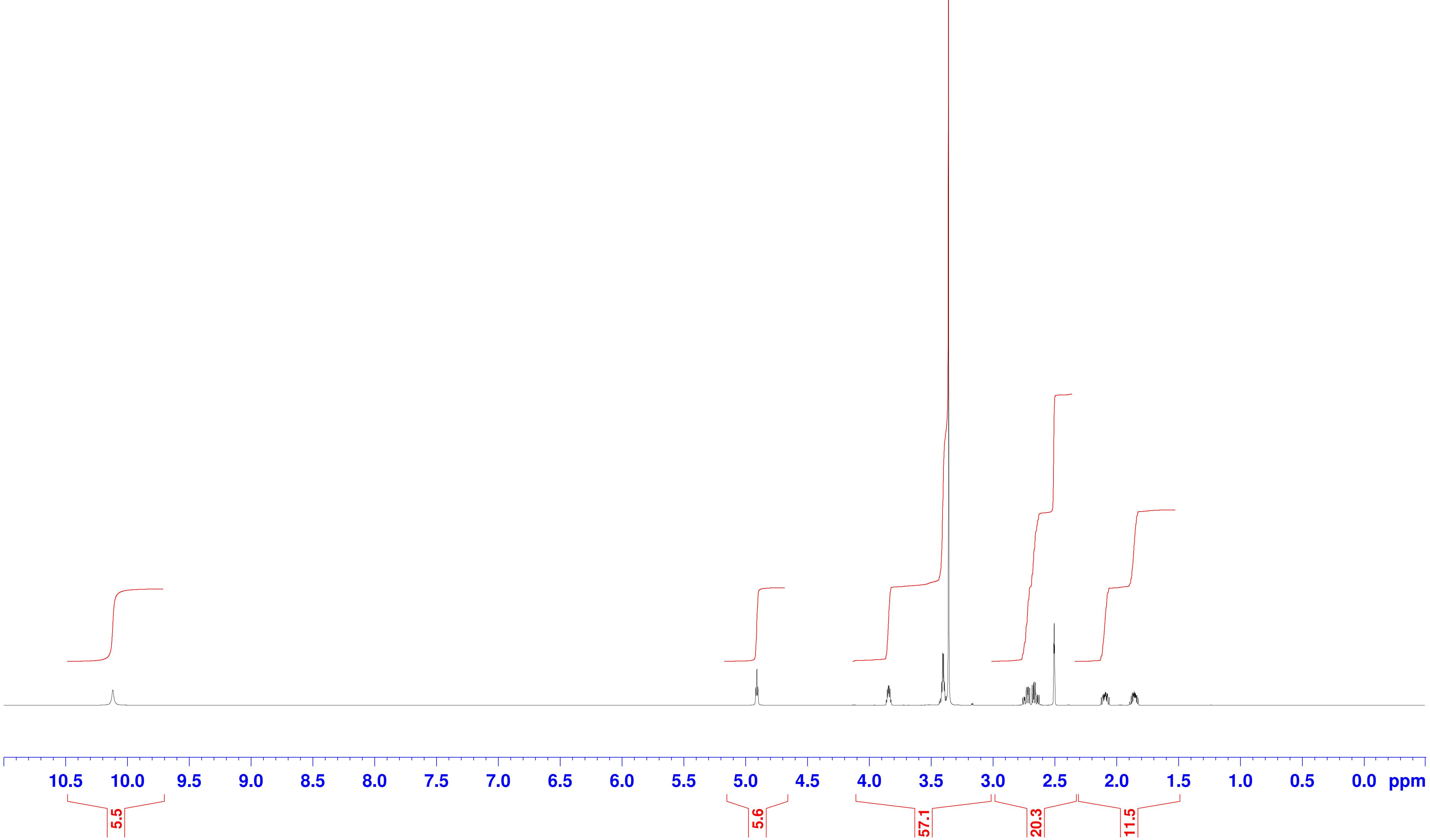


| | |
|---|---|
| NAME | JC-597-2 |
| EXPNO | 10 |
| PROCNO | 1 |
| Date_ | 20110905 |
| Time | 17.19 |
| INSTRUM | AV600 |
| PROBHD | 5 mm CPDCH 13C |
| PULPROG | zg30 |
| TD | 98682 |
| SOLVENT | DMSO |
| NS | 16 |
| DS | 0 |
| SWH | 12335.526 Hz |
| FIDRES | 0.125003 Hz |
| AQ | 3.9999604 sec |
| RG | 32 |
| DW | 40.533 use |
| DE | 10.48 use |
| TE | 298.0 K |
| D1 | 1.00000000 sec |
| TD0 | 1 |

======== CHANNEL f1 =======

| | |
|---|---|
| NUC1 | 1H |
| P1 | 11.40 use |
| PL1 | 1.00 dB |
| PL1W | 13.76731014 W |
| SFO1 | 600.1337061 MHz |
| SI | 32768 |
| SF | 600.1300041 MHz |
| WDW | EM |
| SSB | 0 |
| LB | 0.30 Hz |
| GB | 0 |
| PC | 1.40 |

JC−597−2
C13CPD.ucl DMSO {V:\Bruker\TOPSPIN} mjp 34

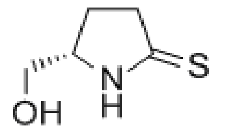

30b

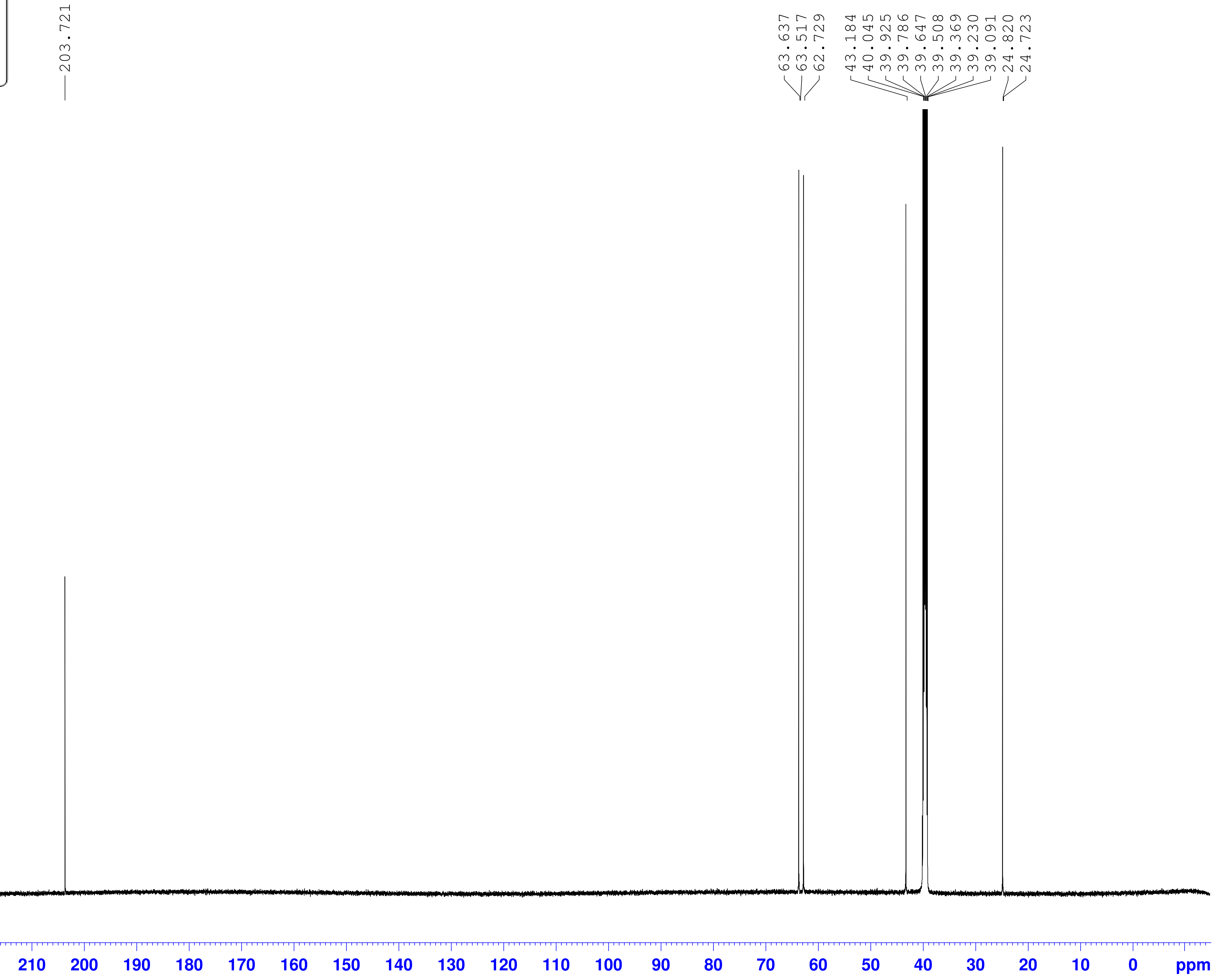


| | |
|---|---|
| NAME | JC-597-2 |
| EXPNO | 12 |
| PROCNO | 1 |
| Date_ | 20110906 |
| Time | 3.38 |
| INSTRUM | AV600 |
| PROBHD | 5 mm CPDCH 13C |
| PULPROG | zgpg30 |
| TD | 70308 |
| SOLVENT | DMSO |
| NS | 512 |
| DS | 0 |
| SWH | 39062.500 H |
| FIDRES | 0.555591 H |
| AQ | 0.8999924 s |
| RG | 1030 |
| DW | 12.800 u |
| DE | 21.12 u |
| TE | 298.0 K |
| D1 | 2.00000000 s |
| D11 | 0.03000000 s |
| TD0 | 1 |
| ======== CHANNEL f1 ===== | |
| NUC1 | 13C |
| P1 | 9.80 u |
| PL1 | 5.00 d |
| PL1W | 26.76886177 W |
| SFO1 | 150.9201628 M |
| ======== CHANNEL f2 ===== | |
| CPDPRG2 | waltz16 |
| NUC2 | 1H |
| PCPD2 | 70.00 u |
| PL2 | 1.00 d |
| PL12 | 17.23 d |
| PL13 | 20.00 d |
| PL2W | 13.76731014 W |
| PL12W | 0.32798135 W |
| PL13W | 0.17332016 W |
| SFO2 | 600.1324005 M |
| SI | 65536 |
| SF | 150.9028745 M |
| WDW | EM |
| SSB | 0 |
| LB | 1.00 H |
| GB | 0 |
| PC | 1.40 |

# JC–597–2
# COSY.ucl DMSO {V:\Bruker\TOPSPIN} mjp 34

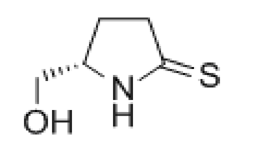

30b

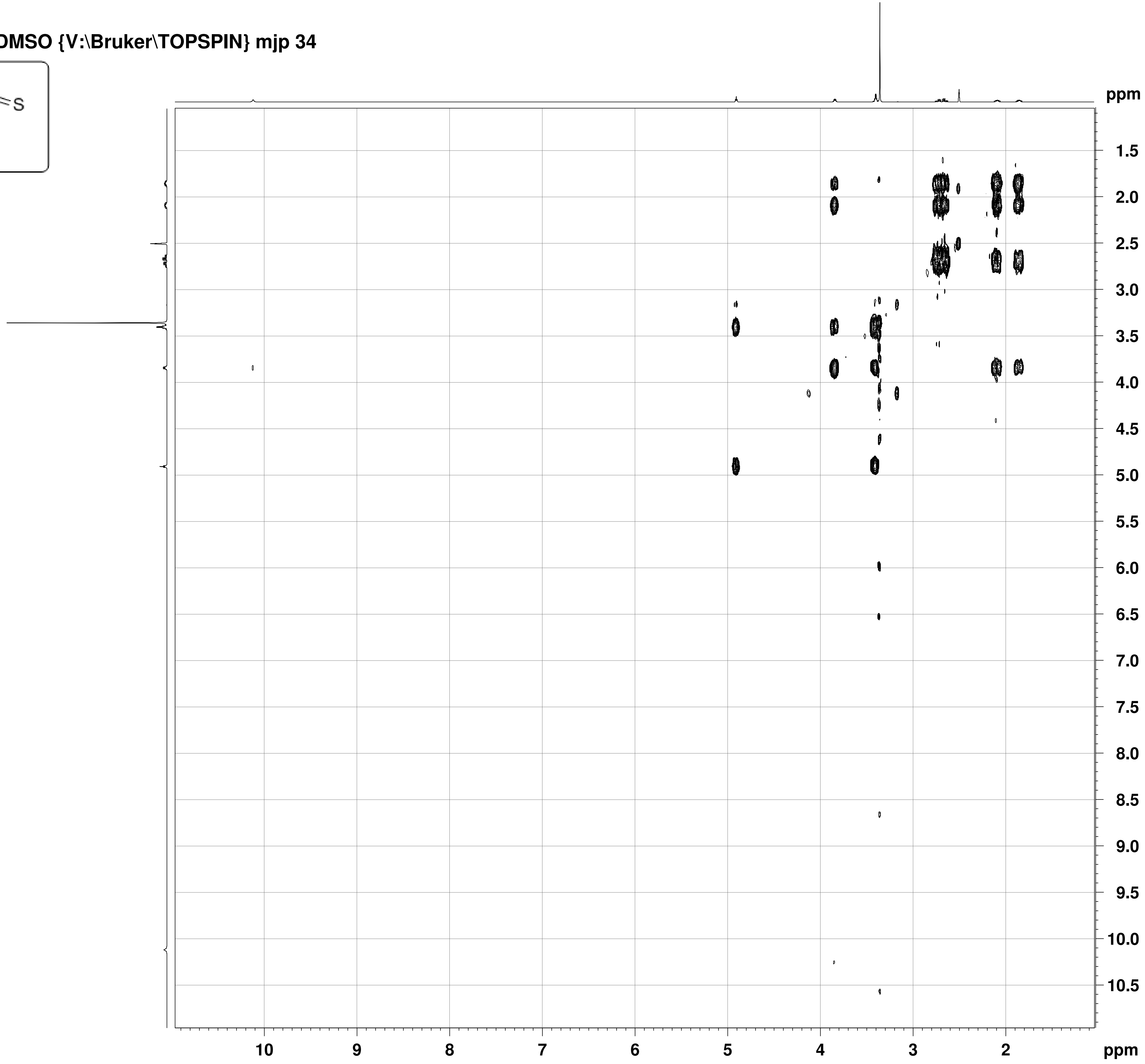


```
NAME              JC-597-2
EXPNO                   11
PROCNO                   1
Date_             20110905
Time                 17.19
INSTRUM              AV600
PROBHD   5 mm CPDCH 13C
PULPROG         cosygpmfqf
TD                    2048
SOLVENT               DMSO
NS                       1
DS                       8
SWH               5952.381 Hz
FIDRES            2.906436 Hz
AQ               0.1720820 sec
RG                    2050
DW                  84.000 usec
DE                    6.50 usec
TE                   298.0 K
D0              0.00000300 sec
D1              1.72802496 sec
D13             0.00000400 sec
D16             0.00020000 sec
IN0             0.00016800 sec

======== CHANNEL f1 ========
NUC1                    1H
P1                   11.40 usec
PL1                   1.00 dB
PL1W           13.76731014 W
SFO1           600.1336061 MHz

====== GRADIENT CHANNEL ====
GPNAM1            SINE.100
GPNAM2            SINE.100
GPNAM3            SINE.100
GPZ1                 16.00 %
GPZ2                 12.00 %
GPZ3                 40.00 %
P16                1000.00 usec
ND0                      1
TD                     128
SFO1              600.1336 MHz
FIDRES           46.502975 Hz
SW                   9.918 ppm
FnMODE                  QF
SI                    1024
SF             600.1300014 MHz
WDW                  QSINE
SSB                      0
LB                    0.00 Hz
GB                       0
PC                    1.40
SI                    1024
MC2                     QF
SF             600.1300014 MHz
WDW                  QSINE
SSB                      0
LB                    0.00 Hz
GB                       0
```

**JC–597–2**
**HSQC.ucl DMSO {V:\Bruker\TOPSPIN} mjp 34**

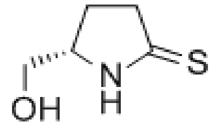

**30b**

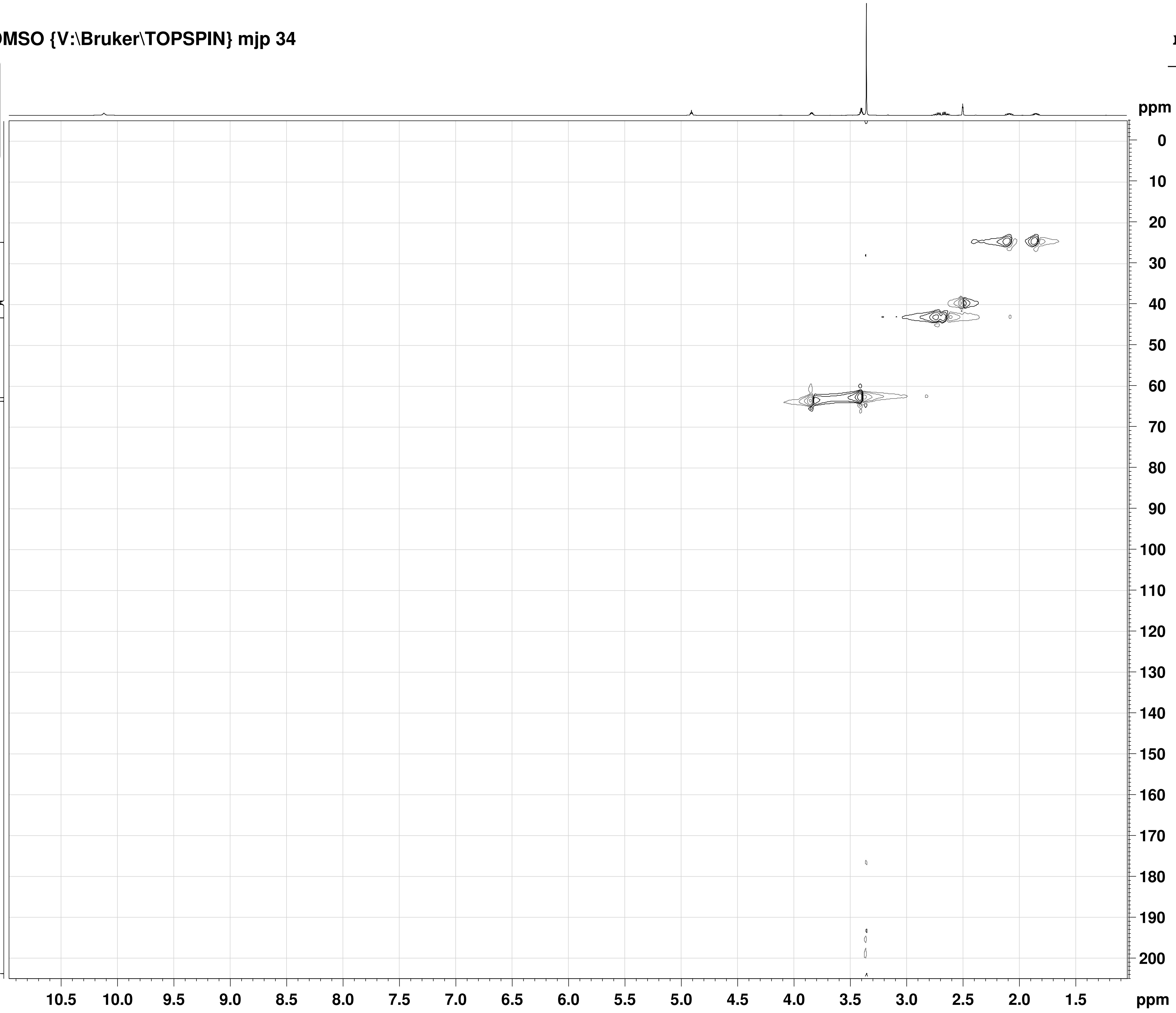


| | |
|---|---|
| NAME | JC-597-2 |
| EXPNO | 13 |
| PROCNO | 1 |
| Date_ | 20110906 |
| Time | 3.40 |
| INSTRUM | AV600 |
| PROBHD | 5 mm CPDCH 13C |
| PULPROG | hsqcedetgpsisp2.4 |
| TD | 1024 |
| SOLVENT | DMSO |
| NS | 2 |
| DS | 32 |
| SWH | 5952.381 Hz |
| FIDRES | 5.812872 Hz |
| AQ | 0.0860660 sec |
| RG | 2050 |
| DW | 84.000 usec |
| DE | 6.50 usec |
| TE | 298.0 K |
| CNST2 | 145.0000000 |
| CNST17 | -0.5000000 |
| D0 | 0.00000300 sec |
| D1 | 1.49918103 sec |
| D2 | 0.00344828 sec |
| D4 | 0.00172414 sec |
| D11 | 0.03000000 sec |
| D16 | 0.00020000 sec |
| D21 | 0.00344828 sec |
| D24 | 0.00086207 sec |
| IN0 | 0.00001580 sec |
| L0 | 0 |
| L31 | 1 |
| TD0 | 2 |
| ======== CHANNEL f1 ======== | |
| NUC1 | 1H |
| P1 | 11.40 usec |
| P2 | 22.80 usec |
| P28 | 0.00 usec |
| PL1 | 1.00 dB |
| PL1W | 13.76731014 W |
| SFO1 | 600.1336061 MHz |
| ======== CHANNEL f2 ======== | |
| CPDPRG2 | bi_p5m4sp_4sp.2 |
| NUC2 | 13C |
| P3 | 9.80 usec |
| P14 | 500.00 usec |
| P24 | 2000.00 usec |
| P31 | 1730.00 usec |
| P63 | 1500.00 usec |
| PL0 | 120.00 dB |
| PL2 | 5.00 dB |
| PL12 | 20.74 dB |
| PL0W | 0.00000000 W |
| PL2W | 26.76886177 W |
| PL12W | 0.71388775 W |
| SFO2 | 150.9178993 MHz |
| SP3 | 13.33 dB |
| SP7 | 13.33 dB |
| SP14 | 14.82 dB |
| SP18 | 18.73 dB |
| SP31 | 20.84 dB |
| SPNAM3 | Crp60,0.5,20.1 |
| SPNAM7 | Crp60comp.4 |
| SPNAM14 | Crp32,1.5,20.2 |
| SPNAM18 | Crp60_xfilt.2 |
| SPNAM31 | Crp32,1.5,20.2 |
| SPOAL3 | 0.500 |
| SPOAL7 | 0.500 |
| SPOAL14 | 0.500 |
| SPOAL18 | 0.500 |
| SPOAL31 | 0.500 |
| SPOFFS3 | 0.00 Hz |
| SPOFFS7 | 0.00 Hz |
| SPOFFS14 | 0.00 Hz |
| SPOFFS18 | 0.00 Hz |
| SPOFFS31 | 0.00 Hz |
| ====== GRADIENT CHANNEL ===== | |
| GPNAM1 | SINE.100 |
| GPNAM2 | SINE.100 |
| GPNAM3 | SINE.100 |
| GPNAM4 | SINE.100 |
| GPZ1 | 80.00 % |
| GPZ2 | 20.10 % |
| GPZ3 | 11.00 % |
| GPZ4 | -5.00 % |
| P16 | 1000.00 usec |
| P19 | 600.00 usec |
| ND0 | 2 |
| TD | 128 |
| SFO1 | 150.9179 MHz |
| FIDRES | 247.599686 Hz |
| SW | 210.000 ppm |
| FnMODE | Echo-Antiecho |
| SI | 1024 |
| SF | 600.1300014 MHz |
| WDW | QSINE |
| SSB | 2 |
| LB | 0.00 Hz |
| GB | 0 |
| PC | 1.40 |
| SI | 1024 |
| MC2 | echo-antiecho |
| SF | 150.9028593 MHz |
| WDW | QSINE |
| SSB | 2 |
| LB | 0.00 Hz |
| GB | 0 |

JC–597–2
HMBC.ucl DMSO {V:\Bruker\TOPSPIN} mjp 34

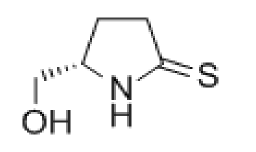

30b

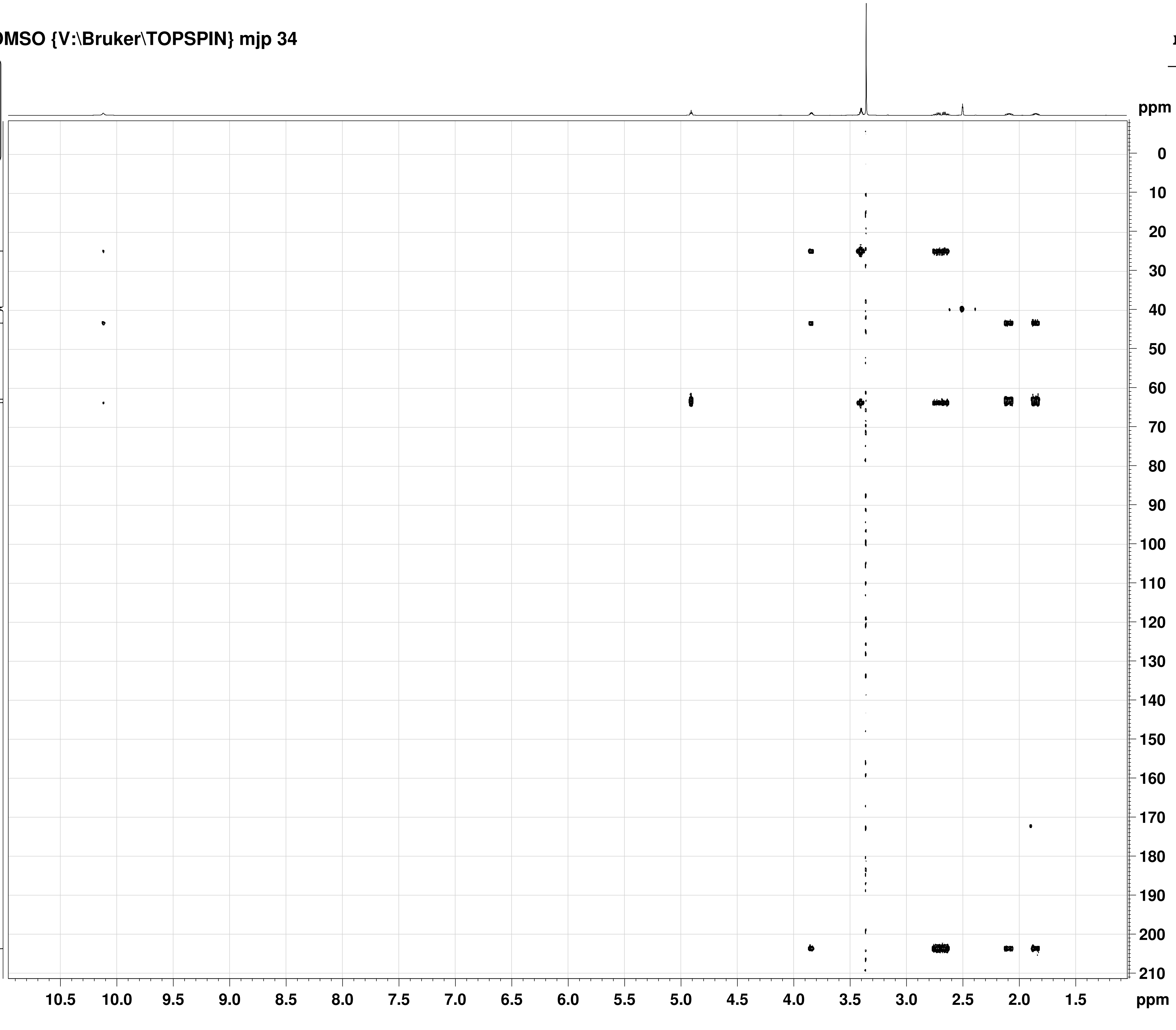


| | |
|---|---|
| NAME | JC-597-2 |
| EXPNO | 14 |
| PROCNO | 1 |
| Date_ | 20110906 |
| Time | 3.47 |
| INSTRUM | AV600 |
| PROBHD | 5 mm CPDCH 13C |
| PULPROG | hmbcetgpl3nd |
| TD | 4096 |
| SOLVENT | DMSO |
| NS | 2 |
| DS | 16 |
| SWH | 5952.381 Hz |
| FIDRES | 1.453218 Hz |
| AQ | 0.3441140 sec |
| RG | 2050 |
| DW | 84.000 usec |
| DE | 6.50 usec |
| TE | 298.0 K |
| CNST6 | 120.0000000 |
| CNST7 | 160.0000000 |
| CNST13 | 10.0000000 |
| CNST30 | 0.5981157 |
| D0 | 0.00000300 sec |
| D1 | 0.83373058 sec |
| D6 | 0.05000000 sec |
| D16 | 0.00020000 sec |
| IN0 | 0.00001505 sec |
| ======== CHANNEL f1 ======== | |
| NUC1 | 1H |
| P1 | 11.40 usec |
| P2 | 22.80 usec |
| PL1 | 1.00 dB |
| PL1W | 13.76731014 W |
| SFO1 | 600.1336061 MHz |
| ======== CHANNEL f2 ======== | |
| NUC2 | 13C |
| P3 | 9.80 usec |
| P24 | 2000.00 usec |
| PL2 | 5.00 dB |
| PL2W | 26.76886177 W |
| SFO2 | 150.9178993 MHz |
| SP7 | 13.33 dB |
| SPNAM7 | Crp60comp.4 |
| SPOAL7 | 0.500 |
| SPOFFS7 | 0.00 Hz |
| ====== GRADIENT CHANNEL ===== | |
| GPNAM1 | SINE.100 |
| GPNAM3 | SINE.100 |
| GPNAM4 | SINE.100 |
| GPNAM5 | SINE.100 |
| GPNAM6 | SINE.100 |
| GPZ1 | 80.00 % |
| GPZ3 | 14.00 % |
| GPZ4 | -8.00 % |
| GPZ5 | -4.00 % |
| GPZ6 | -2.00 % |
| P16 | 1000.00 usec |
| ND0 | 2 |
| TD | 256 |
| SFO1 | 150.9179 MHz |
| FIDRES | 129.695068 Hz |
| SW | 220.000 ppm |
| FnMODE | Echo-Antiecho |
| SI | 2048 |
| SF | 600.1300028 MHz |
| WDW | SINE |
| SSB | 2 |
| LB | 0.00 Hz |
| GB | 0 |
| PC | 1.40 |
| SI | 1024 |
| MC2 | echo-antiecho |
| SF | 150.9028586 MHz |
| WDW | SINE |
| SSB | 2 |
| LB | 0.00 Hz |
| GB | 0 |

**JC–598–2**
**PROTON.ucl CDCl3 {V:\Bruker\TOPSPIN} mjp 42**

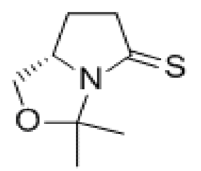

**29**

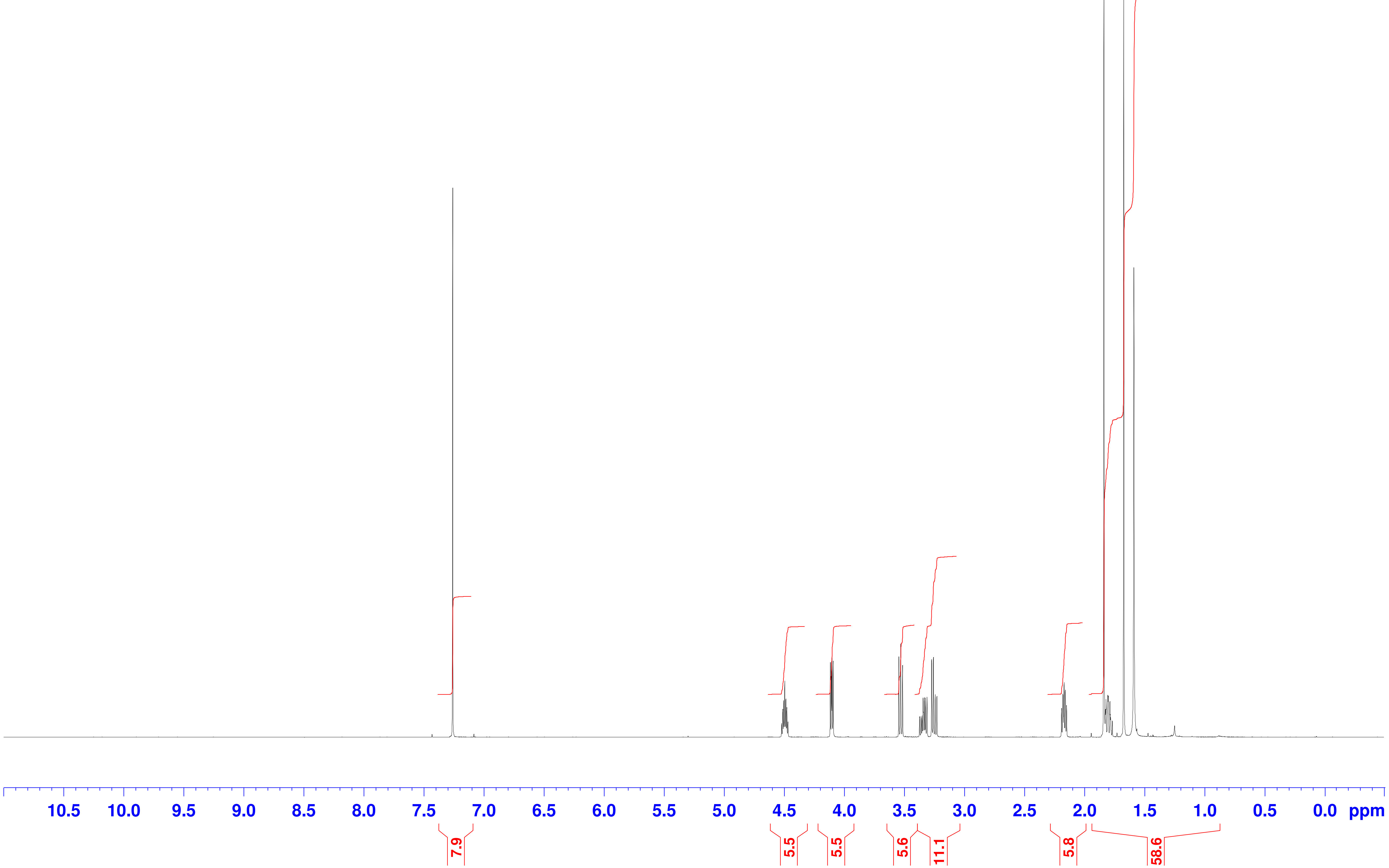


UCL

| | |
|---|---|
| NAME | JC-598-2 |
| EXPNO | 10 |
| PROCNO | 1 |
| Date_ | 20110906 |
| Time | 17.44 |
| INSTRUM | AV600 |
| PROBHD | 5 mm CPDCH 13C |
| PULPROG | zg30 |
| TD | 98682 |
| SOLVENT | CDCl3 |
| NS | 8 |
| DS | 0 |
| SWH | 12335.526 Hz |
| FIDRES | 0.125003 Hz |
| AQ | 3.9999604 sec |
| RG | 40.3 |
| DW | 40.533 use |
| DE | 10.48 use |
| TE | 298.0 K |
| D1 | 1.00000000 sec |
| TD0 | 1 |

======== CHANNEL f1 =======

| | |
|---|---|
| NUC1 | 1H |
| P1 | 11.40 use |
| PL1 | 1.00 dB |
| PL1W | 13.76731014 W |
| SFO1 | 600.1337061 MHz |
| SI | 32768 |
| SF | 600.1300116 MHz |
| WDW | EM |
| SSB | 0 |
| LB | 0.30 Hz |
| GB | 0 |
| PC | 1.40 |

JC−598−2
C13CPD.ucl CDCl3 {V:\Bruker\TOPSPIN} mjp 42

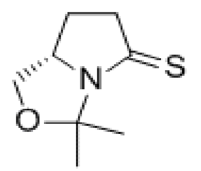

29

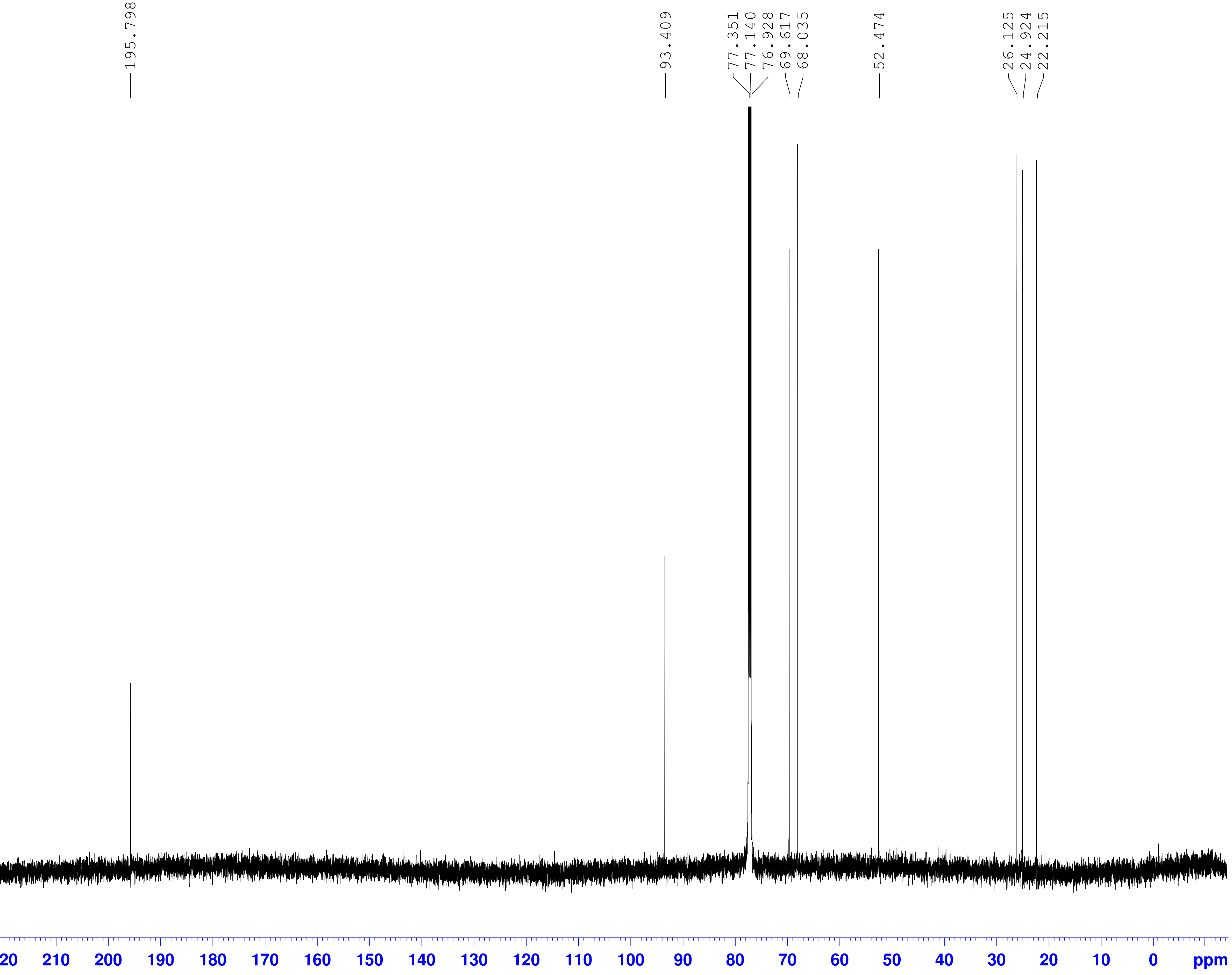


| | |
|---|---|
| NAME | JC-598-2 |
| EXPNO | 12 |
| PROCNO | 1 |
| Date_ | 20110906 |
| Time | 18.02 |
| INSTRUM | AV600 |
| PROBHD | 5 mm CPDCH 13C |
| PULPROG | zgpg30 |
| TD | 70308 |
| SOLVENT | CDCl3 |
| NS | 128 |
| DS | 0 |
| SWH | 39062.500 H |
| FIDRES | 0.555591 H |
| AQ | 0.8999924 s |
| RG | 1030 |
| DW | 12.800 u |
| DE | 21.12 u |
| TE | 298.0 K |
| D1 | 2.00000000 s |
| D11 | 0.03000000 s |
| TD0 | 1 |

======== CHANNEL f1 =====

| | |
|---|---|
| NUC1 | 13C |
| P1 | 9.80 u |
| PL1 | 5.00 d |
| PL1W | 26.76886177 W |
| SFO1 | 150.9201628 M |

======== CHANNEL f2 =====

| | |
|---|---|
| CPDPRG2 | waltz16 |
| NUC2 | 1H |
| PCPD2 | 70.00 u |
| PL2 | 1.00 d |
| PL12 | 17.23 d |
| PL13 | 20.00 d |
| PL2W | 13.76731014 W |
| PL12W | 0.32798135 W |
| PL13W | 0.17332016 W |
| SFO2 | 600.1324005 M |
| SI | 65536 |
| SF | 150.9027930 M |
| WDW | EM |
| SSB | 0 |
| LB | 1.00 H |
| GB | 0 |
| PC | 1.40 |

JC–598–2
COSY.ucl CDCl3 {V:\Bruker\TOPSPIN} mjp 42

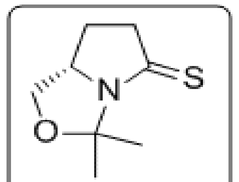


29

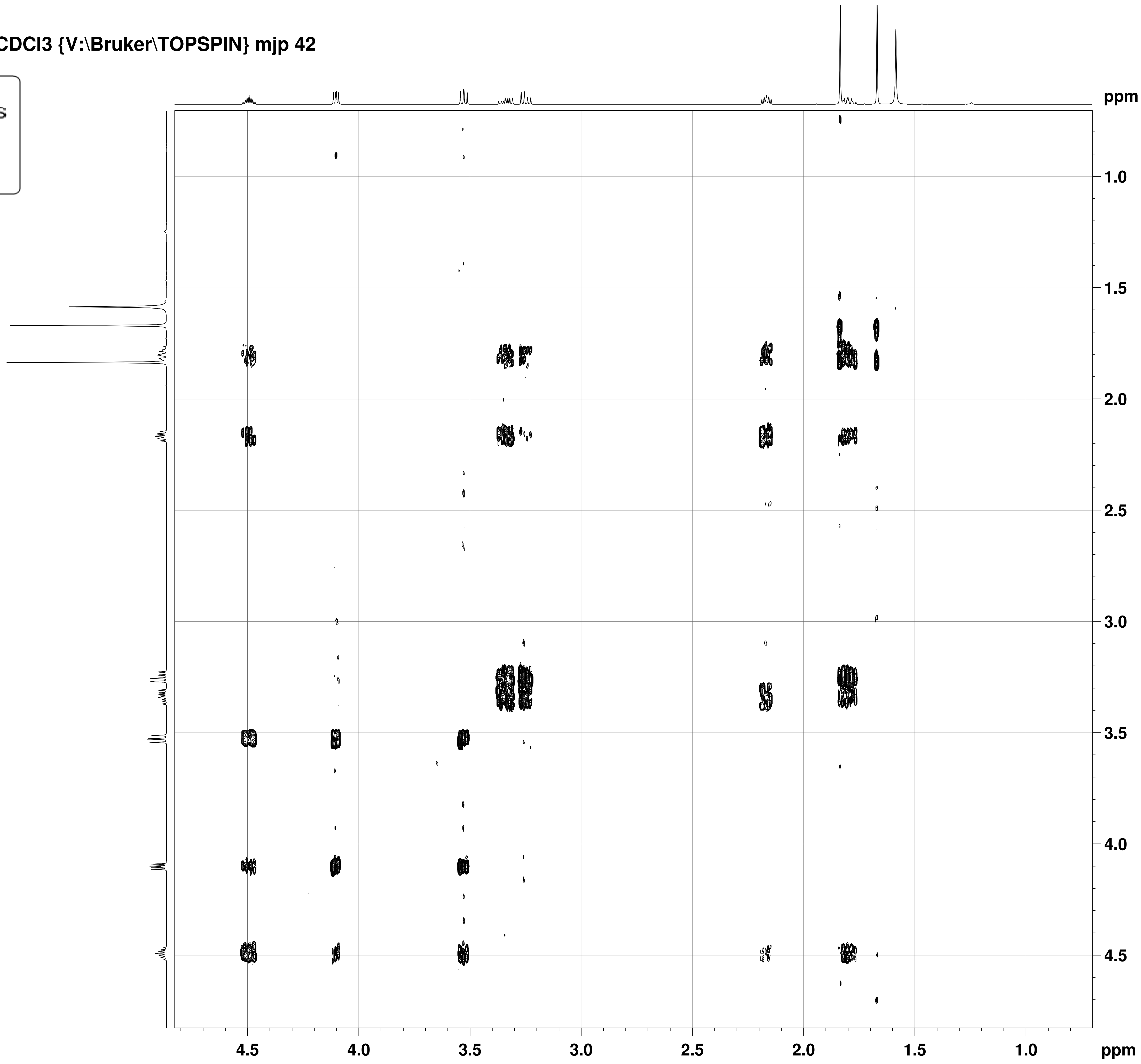


| | |
|---|---|
| NAME | JC-598-2 |
| EXPNO | 11 |
| PROCNO | 1 |
| Date_ | 20110906 |
| Time | 17.45 |
| INSTRUM | AV600 |
| PROBHD | 5 mm CPDCH 13C |
| PULPROG | cosygpmfqf |
| TD | 2048 |
| SOLVENT | CDCl3 |
| NS | 1 |
| DS | 8 |
| SWH | 2475.248 Hz |
| FIDRES | 1.208617 Hz |
| AQ | 0.4137460 sec |
| RG | 2050 |
| DW | 202.000 usec |
| DE | 6.50 usec |
| TE | 298.0 K |
| D0 | 0.00000300 sec |
| D1 | 1.48636103 sec |
| D13 | 0.00000400 sec |
| D16 | 0.00020000 sec |
| IN0 | 0.00040400 sec |
| ======== CHANNEL f1 ======== | |
| NUC1 | 1H |
| P1 | 11.40 usec |
| PL1 | 1.00 dB |
| PL1W | 13.76731014 W |
| SFO1 | 600.1316709 MHz |
| ====== GRADIENT CHANNEL ==== | |
| GPNAM1 | SINE.100 |
| GPNAM2 | SINE.100 |
| GPNAM3 | SINE.100 |
| GPZ1 | 16.00 % |
| GPZ2 | 12.00 % |
| GPZ3 | 40.00 % |
| P16 | 1000.00 usec |
| ND0 | 1 |
| TD | 128 |
| SFO1 | 600.1317 MHz |
| FIDRES | 19.337872 Hz |
| SW | 4.125 ppm |
| FnMODE | QF |
| SI | 1024 |
| SF | 600.1300106 MHz |
| WDW | QSINE |
| SSB | 0 |
| LB | 0.00 Hz |
| GB | 0 |
| PC | 1.40 |
| SI | 1024 |
| MC2 | QF |
| SF | 600.1300106 MHz |
| WDW | QSINE |
| SSB | 0 |
| LB | 0.00 Hz |
| GB | 0 |

**JC–598–2**
**HSQC.ucl CDCl3 {V:\Bruker\TOPSPIN} mjp 42**

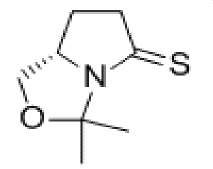

**29**

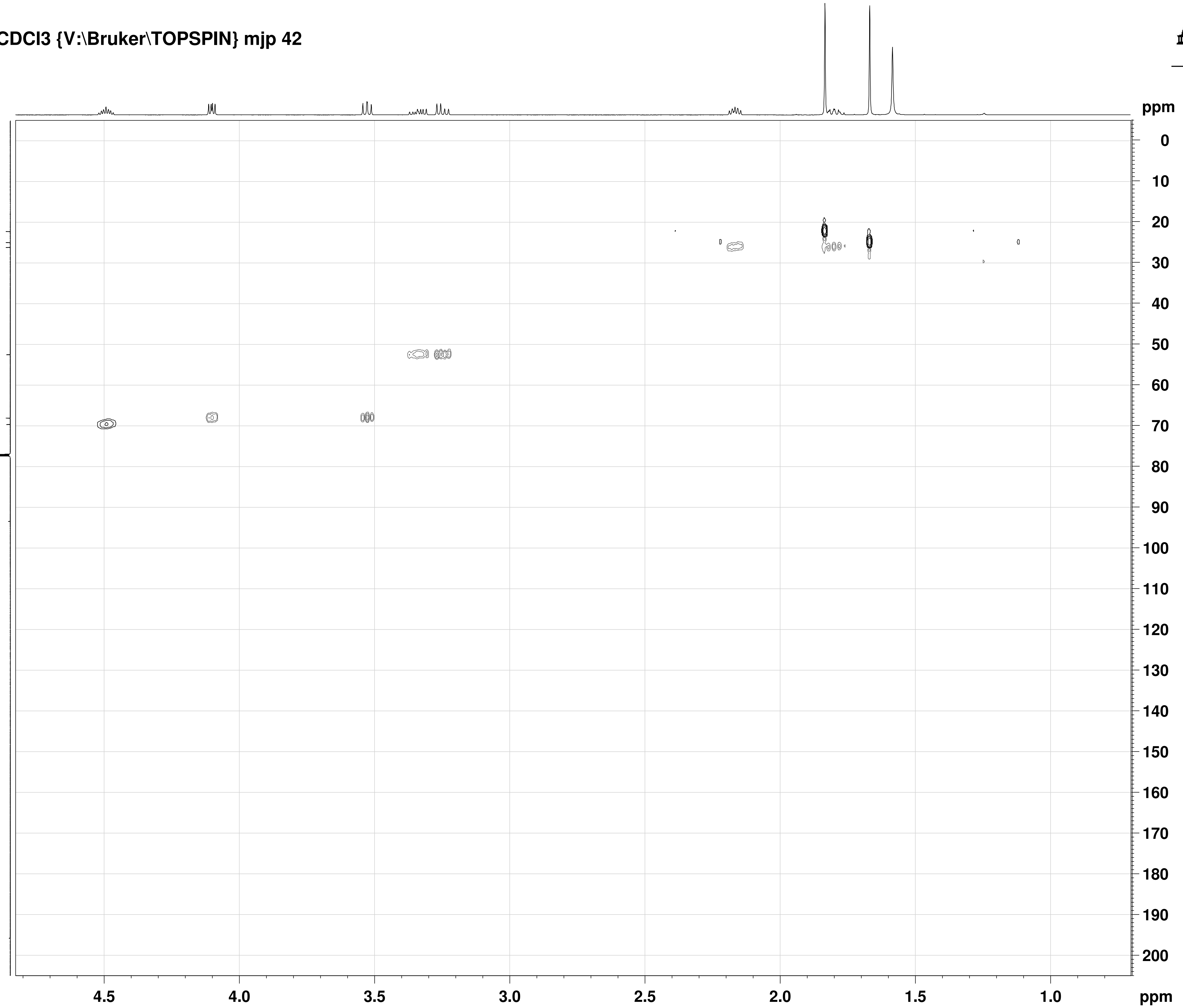


```
NAME            JC-598-2
EXPNO                 13
PROCNO                 1
Date_           20110906
Time               18.04
INSTRUM            AV600
PROBHD   5 mm CPDCH 13C
PULPROG  hsqcedetgpsisp2.4
TD                  1024
SOLVENT            CDCl3
NS                     2
DS                    32
SWH             2475.248 Hz
FIDRES          2.417234 Hz
AQ             0.2068980 sec
RG                  2050
DW               202.000 usec
DE                  6.50 usec
TE                 298.0 K
CNST2        145.0000000
CNST17        -0.5000000
D0            0.00000300 sec
D1            1.37834895 sec
D2            0.00344828 sec
D4            0.00172414 sec
D11           0.03000000 sec
D16           0.00020000 sec
D21           0.00344828 sec
D24           0.00086207 sec
IN0           0.00001580 sec
L0                     0
L31                    1
TD0                    2

======== CHANNEL f1 ========
NUC1                  1H
P1                 11.40 usec
P2                 22.80 usec
P28                 0.00 usec
PL1                 1.00 dB
PL1W         13.76731014 W
SFO1         600.1316709 MHz

======== CHANNEL f2 ========
CPDPRG2  bi_p5m4sp_4sp.2
NUC2                 13C
P3                  9.80 usec
P14               500.00 usec
P24              2000.00 usec
P31              1730.00 usec
P63              1500.00 usec
PL0               120.00 dB
PL2                 5.00 dB
PL12               20.74 dB
PL0W          0.00000000 W
PL2W         26.76886177 W
PL12W         0.71388775 W
SFO2         150.9178993 MHz
SP3                13.33 dB
SP7                13.33 dB
SP14               14.82 dB
SP18               18.73 dB
SP31               20.84 dB
SPNAM3    Crp60,0.5,20.1
SPNAM7      Crp60comp.4
SPNAM14   Crp32,1.5,20.2
SPNAM18    Crp60_xfilt.2
SPNAM31   Crp32,1.5,20.2
SPOAL3             0.500
SPOAL7             0.500
SPOAL14            0.500
SPOAL18            0.500
SPOAL31            0.500
SPOFFS3             0.00 Hz
SPOFFS7             0.00 Hz
SPOFFS14            0.00 Hz
SPOFFS18            0.00 Hz
SPOFFS31            0.00 Hz

====== GRADIENT CHANNEL =====
GPNAM1          SINE.100
GPNAM2          SINE.100
GPNAM3          SINE.100
GPNAM4          SINE.100
GPZ1               80.00 %
GPZ2               20.10 %
GPZ3               11.00 %
GPZ4               -5.00 %
P16              1000.00 usec
P19               600.00 usec
ND0                    2
TD                   128
SFO1            150.9179 MHz
FIDRES        247.599686 Hz
SW               210.000 ppm
FnMODE      Echo-Antiecho
SI                  1024
SF           600.1300106 MHz
WDW                QSINE
SSB                    2
LB                  0.00 Hz
GB                     0
PC                  1.40
SI                  1024
MC2         echo-antiecho
SF           150.9027778 MHz
WDW                QSINE
SSB                    2
LB                  0.00 Hz
GB                     0
```

# JC–598–2
## HMBC.ucl CDCl3 {V:\Bruker\TOPSPIN} mjp 42

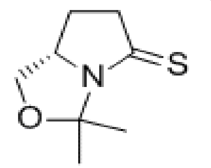

29

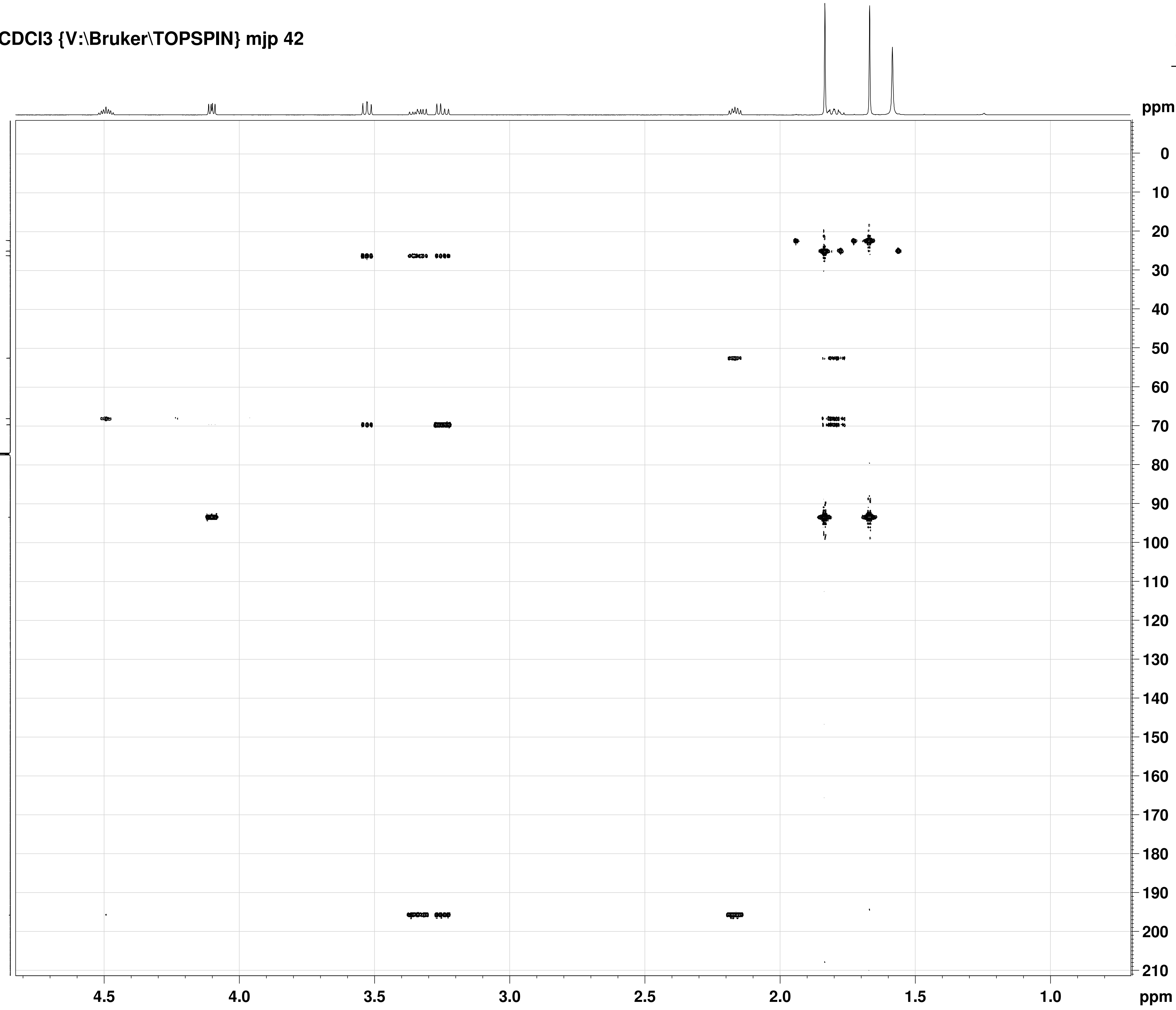


```
NAME              JC-598-2
EXPNO                   14
PROCNO                   1
Date_             20110906
Time                 18.12
INSTRUM              AV600
PROBHD   5 mm CPDCH 13C
PULPROG      hmbcetgpl3nd
TD                    4096
SOLVENT              CDCl3
NS                       2
DS                      16
SWH               2475.248 Hz
FIDRES            0.604308 Hz
AQ               0.8274420 sec
RG                    2050
DW                 202.000 usec
DE                    6.50 usec
TE                   298.0 K
CNST6          120.0000000
CNST7          160.0000000
CNST13          10.0000000
CNST30           0.5981147
D0              0.00000300 sec
D1              0.35040259 sec
D6              0.05000000 sec
D16             0.00020000 sec
IN0             0.00001505 sec

======== CHANNEL f1 ========
NUC1                    1H
P1                   11.40 usec
P2                   22.80 usec
PL1                   1.00 dB
PL1W           13.76731014 W
SFO1           600.1316709 MHz

======== CHANNEL f2 ========
NUC2                   13C
P3                    9.80 usec
P24                2000.00 usec
PL2                   5.00 dB
PL2W           26.76886177 W
SFO2           150.9178993 MHz
SP7                  13.33 dB
SPNAM7         Crp60comp.4
SPOAL7               0.500
SPOFFS7               0.00 Hz

====== GRADIENT CHANNEL =====
GPNAM1           SINE.100
GPNAM3           SINE.100
GPNAM4           SINE.100
GPNAM5           SINE.100
GPNAM6           SINE.100
GPZ1                80.00 %
GPZ3                14.00 %
GPZ4                -8.00 %
GPZ5                -4.00 %
GPZ6                -2.00 %
P16               1000.00 usec
ND0                     2
TD                    256
SFO1             150.9179 MHz
FIDRES         129.695068 Hz
SW                220.000 ppm
FnMODE       Echo-Antiecho
SI                   2048
SF            600.1300112 MHz
WDW                  SINE
SSB                     2
LB                   0.00 Hz
GB                      0
PC                   1.40
SI                   1024
MC2          echo-antiecho
SF            150.9027771 MHz
WDW                  SINE
SSB                     2
LB                   0.00 Hz
GB                      0
```

JC–642–3
PROTON.ucl CDCl3 {V:\Bruker\TOPSPIN} mjp 49

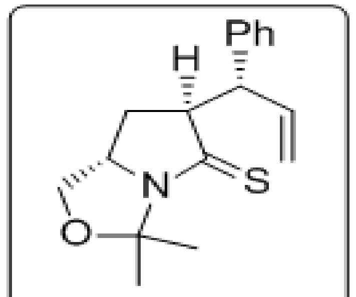


**36**

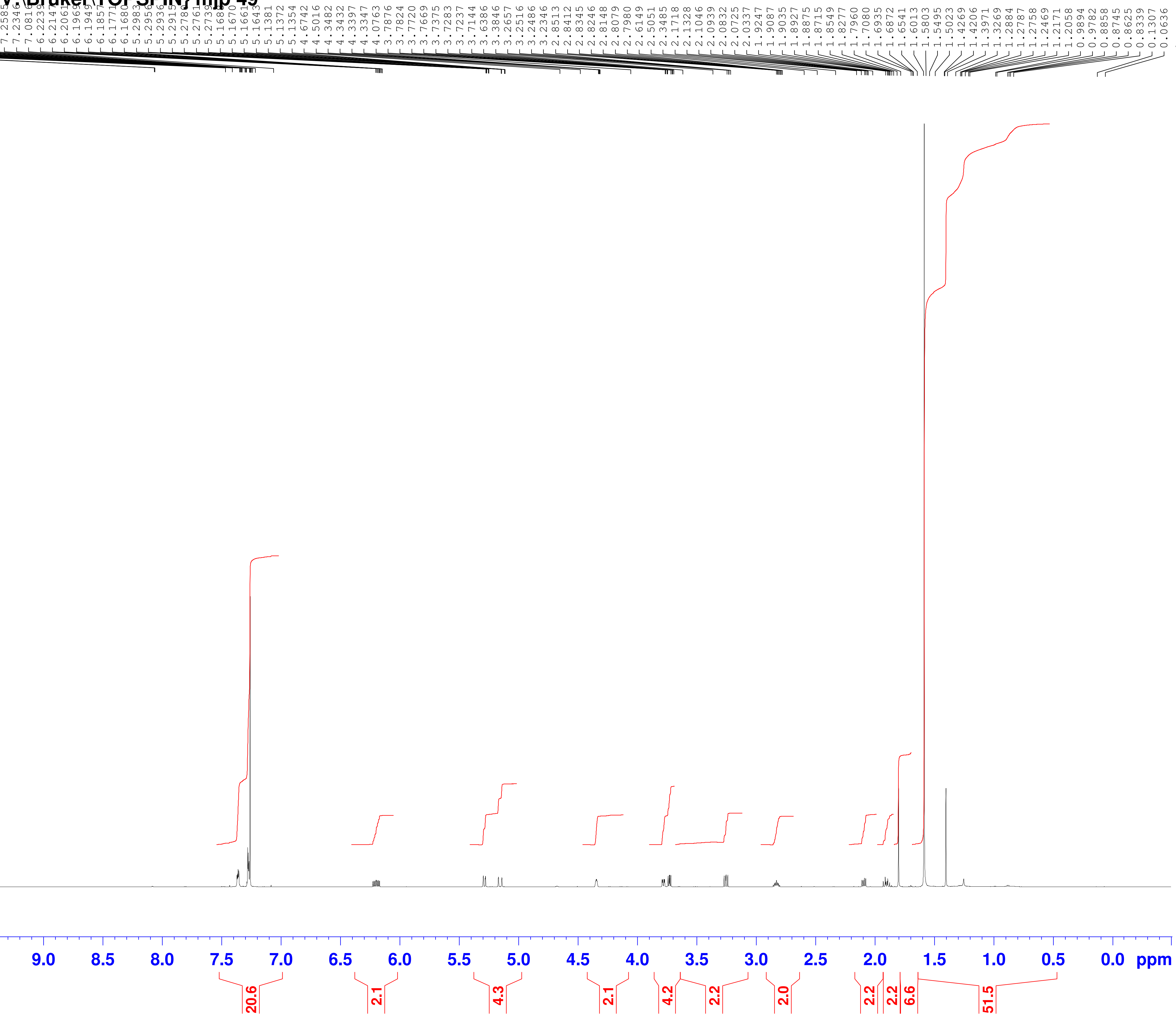


UCL

| | |
|---|---|
| NAME | JC-642-3 |
| EXPNO | 10 |
| PROCNO | 1 |
| Date_ | 20120103 |
| Time | 18.34 |
| INSTRUM | AV600 |
| PROBHD | 5 mm CPDCH 13C |
| PULPROG | zg30 |
| TD | 98682 |
| SOLVENT | CDCl3 |
| NS | 32 |
| DS | 0 |
| SWH | 12335.526 Hz |
| FIDRES | 0.125003 Hz |
| AQ | 3.9999604 sec |
| RG | 36 |
| DW | 40.533 use |
| DE | 10.48 use |
| TE | 298.0 K |
| D1 | 1.00000000 sec |
| TD0 | 1 |

======== CHANNEL f1 ========

| | |
|---|---|
| NUC1 | 1H |
| P1 | 11.40 use |
| PL1 | 1.00 dB |
| PL1W | 13.76731014 W |
| SFO1 | 600.1337061 MHz |
| SI | 32768 |
| SF | 600.1300116 MHz |
| WDW | EM |
| SSB | 0 |
| LB | 0.30 Hz |
| GB | 0 |
| PC | 1.40 |

JC–642–3
C13CPD.ucl CDCl3 {V:\Bruker\TOPSPIN} mjp 49

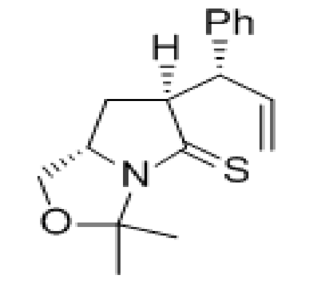


36

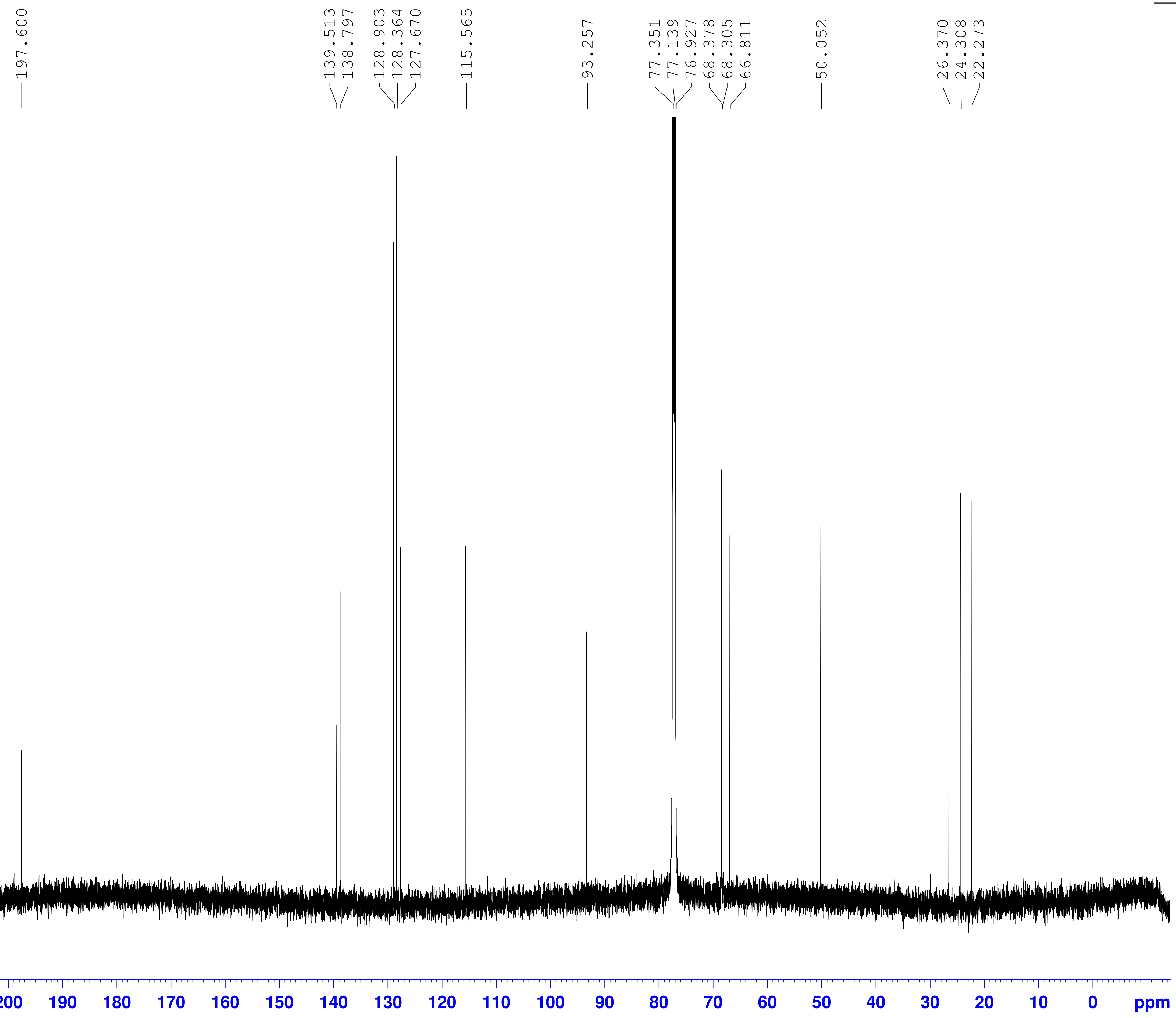


| | |
|---|---|
| NAME | JC-642-3 |
| EXPNO | 12 |
| PROCNO | 1 |
| Date_ | 20120103 |
| Time | 19.04 |
| INSTRUM | AV600 |
| PROBHD | 5 mm CPDCH 13C |
| PULPROG | zgpg30 |
| TD | 70308 |
| SOLVENT | CDCl3 |
| NS | 512 |
| DS | 0 |
| SWH | 39062.500 H |
| FIDRES | 0.555591 H |
| AQ | 0.8999924 s |
| RG | 1030 |
| DW | 12.800 u |
| DE | 21.12 u |
| TE | 298.0 K |
| D1 | 2.00000000 s |
| D11 | 0.03000000 s |
| TD0 | 1 |

======== CHANNEL f1 =====

| | |
|---|---|
| NUC1 | 13C |
| P1 | 9.80 u |
| PL1 | 5.00 d |
| PL1W | 26.76886177 W |
| SFO1 | 150.9201628 M |

======== CHANNEL f2 =====

| | |
|---|---|
| CPDPRG2 | waltz16 |
| NUC2 | 1H |
| PCPD2 | 70.00 u |
| PL2 | 1.00 d |
| PL12 | 17.23 d |
| PL13 | 20.00 d |
| PL2W | 13.76731014 W |
| PL12W | 0.32798135 W |
| PL13W | 0.17332016 W |
| SFO2 | 600.1324005 M |
| SI | 65536 |
| SF | 150.9027930 M |
| WDW | EM |
| SSB | 0 |
| LB | 1.00 H |
| GB | 0 |
| PC | 1.40 |

JC–642–3
COSY.ucl CDCl3 {V:\Bruker\TOPSPIN} mjp 49

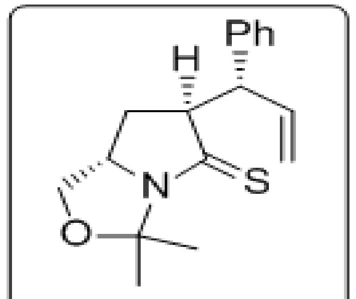

**36**

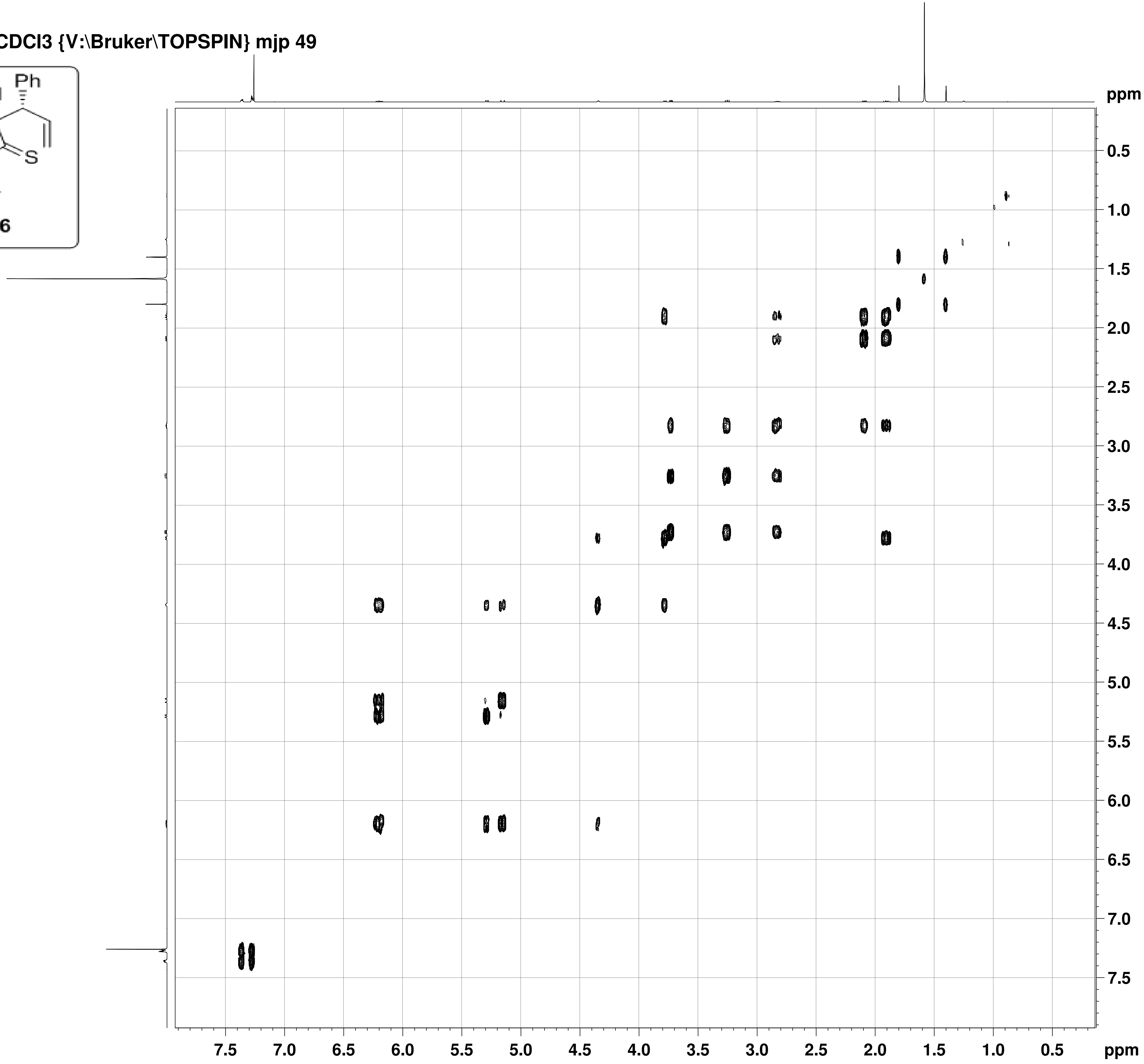


UCL

```
NAME             JC-642-3
EXPNO                  11
PROCNO                  1
Date_            20120103
Time                18.34
INSTRUM             AV600
PROBHD   5 mm CPDCH 13C
PULPROG        cosygpmfqf
TD                   2048
SOLVENT             CDCl3
NS                      1
DS                      8
SWH              4672.897 Hz
FIDRES           2.281688 Hz
AQ              0.2191860 sec
RG                   2050
DW                107.000 usec
DE                   6.50 usec
TE                  298.0 K
D0             0.00000300 sec
D1             1.68092096 sec
D13            0.00000400 sec
D16            0.00020000 sec
IN0            0.00021400 sec

======== CHANNEL f1 ========
NUC1                   1H
P1                  11.40 usec
PL1                  1.00 dB
PL1W          13.76731014 W
SFO1          600.1324312 MHz

====== GRADIENT CHANNEL ====
GPNAM1           SINE.100
GPNAM2           SINE.100
GPNAM3           SINE.100
GPZ1                16.00 %
GPZ2                12.00 %
GPZ3                40.00 %
P16               1000.00 usec
ND0                     1
TD                    128
SFO1             600.1324 MHz
FIDRES          36.507008 Hz
SW                  7.786 ppm
FnMODE                 QF
SI                   1024
SF            600.1300095 MHz
WDW                 QSINE
SSB                     0
LB                   0.00 Hz
GB                      0
PC                   1.40
SI                   1024
MC2                    QF
SF            600.1300095 MHz
WDW                 QSINE
SSB                     0
LB                   0.00 Hz
GB                      0
```

JC–642–3
NOESY.ucl CDCl3 {V:\Bruker\TOPSPIN} mjp 49

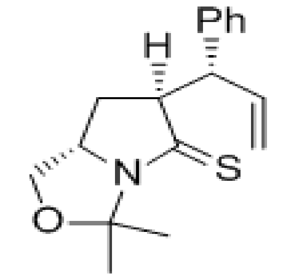


36

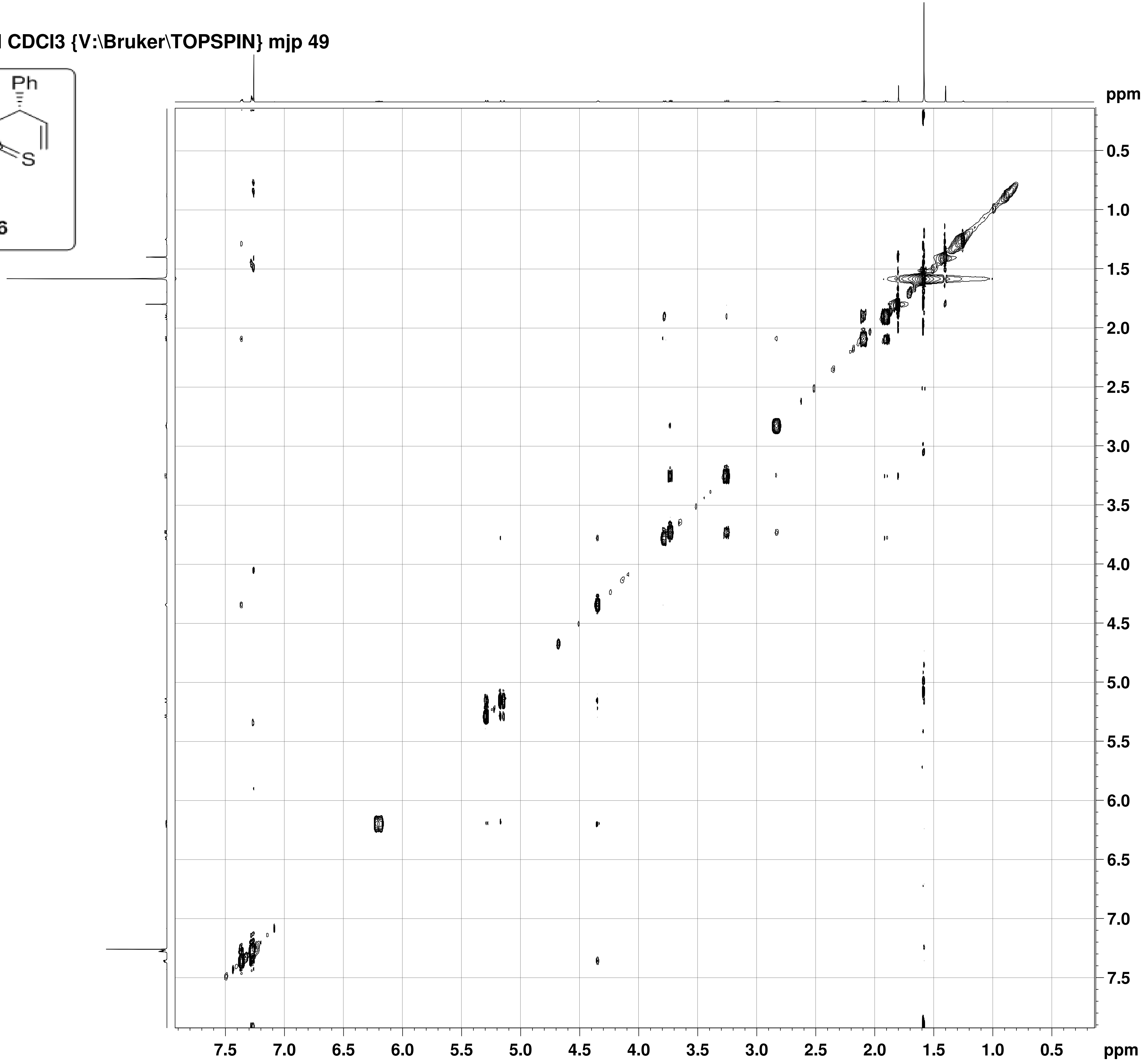


| | |
|---|---|
| NAME | JC-642-3 |
| EXPNO | 15 |
| PROCNO | 1 |
| Date_ | 20120103 |
| Time | 19.25 |
| INSTRUM | AV600 |
| PROBHD | 5 mm CPDCH 13C |
| PULPROG | noesygpph |
| TD | 2048 |
| SOLVENT | CDCl3 |
| NS | 2 |
| DS | 16 |
| SWH | 4672.897 Hz |
| FIDRES | 2.281688 Hz |
| AQ | 0.2191860 sec |
| RG | 50.8 |
| DW | 107.000 usec |
| DE | 6.50 usec |
| TE | 298.0 K |
| D0 | 0.00009325 sec |
| D1 | 1.97009897 sec |
| D8 | 0.60000002 sec |
| D16 | 0.00020000 sec |
| IN0 | 0.00021400 sec |
| ======== CHANNEL f1 ======== | |
| NUC1 | 1H |
| P1 | 10.80 usec |
| P2 | 21.60 usec |
| PL1 | 1.00 dB |
| PL1W | 13.76731014 W |
| SFO1 | 600.1324312 MHz |
| ====== GRADIENT CHANNEL ==== | |
| GPNAM1 | SINE.100 |
| GPNAM2 | SINE.100 |
| GPZ1 | 40.00 % |
| GPZ2 | -40.00 % |
| P16 | 1000.00 usec |
| ND0 | 1 |
| TD | 256 |
| SFO1 | 600.1324 MHz |
| FIDRES | 18.253504 Hz |
| SW | 7.786 ppm |
| FnMODE | States-TPPI |
| SI | 1024 |
| SF | 600.1300095 MHz |
| WDW | QSINE |
| SSB | 2 |
| LB | 0.00 Hz |
| GB | 0 |
| PC | 1.00 |
| SI | 1024 |
| MC2 | States-TPPI |
| SF | 600.1300095 MHz |
| WDW | QSINE |
| SSB | 2 |
| LB | 0.00 Hz |
| GB | 0 |

JC–642–3
HSQC.ucl CDCl3 {V:\Bruker\TOPSPIN} mjp 49

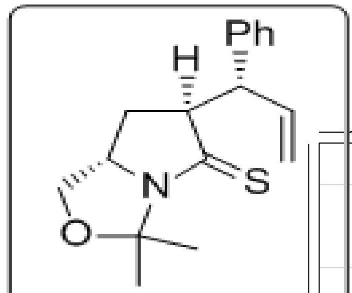


**36**

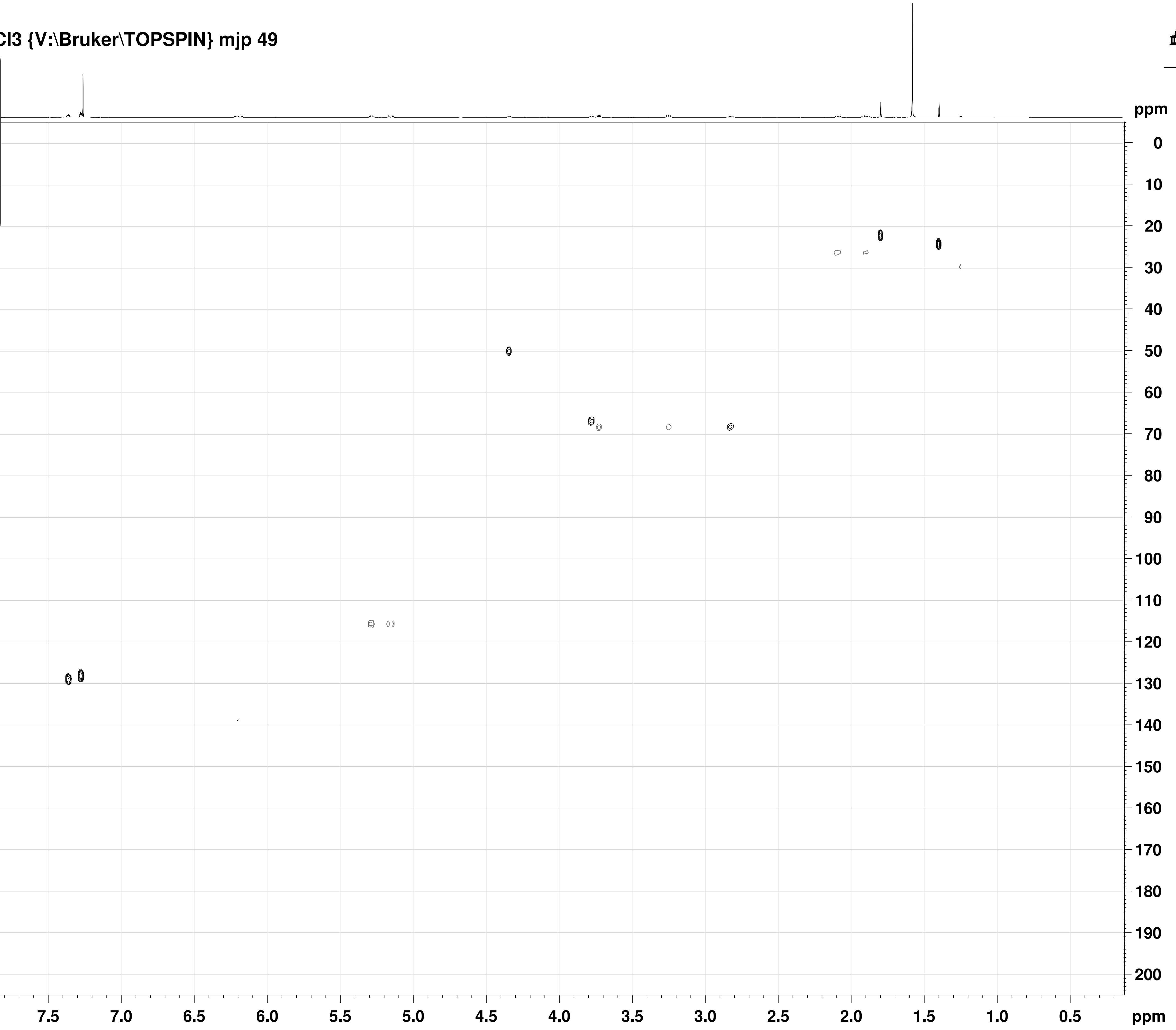


UCL

```
NAME            JC-642-3
EXPNO                 13
PROCNO                 1
Date_           20120103
Time               19.05
INSTRUM            AV600
PROBHD   5 mm CPDCH 13C
PULPROG  hsqcedetgpsisp2.4
TD                  1024
SOLVENT            CDCl3
NS                     2
DS                    32
SWH             4672.897 Hz
FIDRES          4.563376 Hz
AQ             0.1096180 sec
RG                  2050
DW               107.000 usec
DE                  6.50 usec
TE                 298.0 K
CNST2        145.0000000
CNST17        -0.5000000
D0            0.00000300 sec
D1            1.47562897 sec
D2            0.00344828 sec
D4            0.00172414 sec
D11           0.03000000 sec
D16           0.00020000 sec
D21           0.00344828 sec
D24           0.00086207 sec
IN0           0.00001580 sec
L0                     0
L31                    1
TD0                    2

======== CHANNEL f1 ========
NUC1                  1H
P1                 11.40 usec
P2                 22.80 usec
P28                 0.00 usec
PL1                 1.00 dB
PL1W         13.76731014 W
SFO1         600.1324312 MHz

======== CHANNEL f2 ========
CPDPRG2  bi_p5m4sp_4sp.2
NUC2                 13C
P3                  9.80 usec
P14               500.00 usec
P24              2000.00 usec
P31              1730.00 usec
P63              1500.00 usec
PL0               120.00 dB
PL2                 5.00 dB
PL12               20.74 dB
PL0W          0.00000000 W
PL2W         26.76886177 W
PL12W         0.71388775 W
SFO2         150.9178993 MHz
SP3                13.33 dB
SP7                13.33 dB
SP14               14.82 dB
SP18               18.73 dB
SP31               20.84 dB
SPNAM3    Crp60,0.5,20.1
SPNAM7       Crp60comp.4
SPNAM14   Crp32,1.5,20.2
SPNAM18    Crp60_xfilt.2
SPNAM31   Crp32,1.5,20.2
SPOAL3             0.500
SPOAL7             0.500
SPOAL14            0.500
SPOAL18            0.500
SPOAL31            0.500
SPOFFS3             0.00 Hz
SPOFFS7             0.00 Hz
SPOFFS14            0.00 Hz
SPOFFS18            0.00 Hz
SPOFFS31            0.00 Hz

====== GRADIENT CHANNEL =====
GPNAM1          SINE.100
GPNAM2          SINE.100
GPNAM3          SINE.100
GPNAM4          SINE.100
GPZ1               80.00 %
GPZ2               20.10 %
GPZ3               11.00 %
GPZ4               -5.00 %
P16              1000.00 usec
P19               600.00 usec
ND0                    2
TD                   128
SFO1            150.9179 MHz
FIDRES        247.599686 Hz
SW               210.000 ppm
FnMODE      Echo-Antiecho
SI                  1024
SF           600.1300095 MHz
WDW                QSINE
SSB                    2
LB                  0.00 Hz
GB                     0
PC                  1.40
SI                  1024
MC2         echo-antiecho
SF           150.9027778 MHz
WDW                QSINE
SSB                    2
LB                  0.00 Hz
GB                     0
```

JC–642–3
HMBC.ucl CDCl3 {V:\Bruker\TOPSPIN} mjp 49

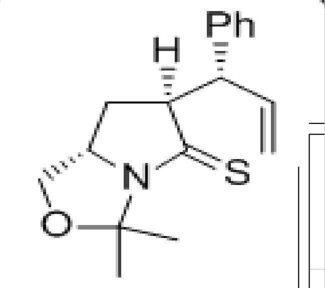


36

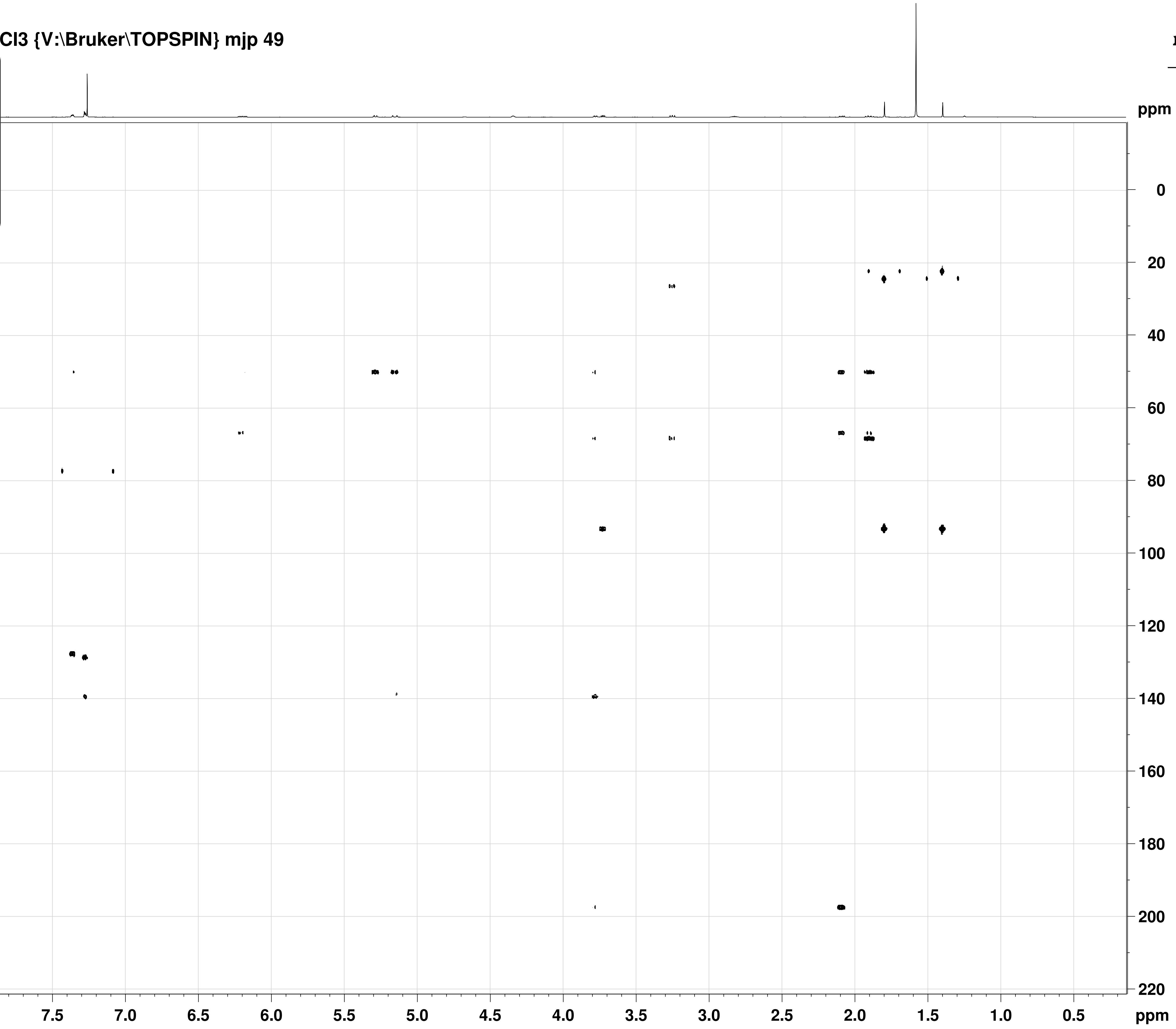


UCL

```
NAME             JC-642-3
EXPNO                  14
PROCNO                  1
Date_            20120103
Time                19.13
INSTRUM             AV600
PROBHD   5 mm CPDCH 13C
PULPROG    hmbcetgpl3nd
TD                   4096
SOLVENT             CDCl3
NS                      2
DS                     16
SWH              4672.897 Hz
FIDRES           1.140844 Hz
AQ              0.4383220 sec
RG                   2050
DW                107.000 usec
DE                   6.50 usec
TE                  298.0 K
CNST6         120.0000000
CNST7         160.0000000
CNST13         10.0000000
CNST30          0.5981151
D0             0.00000300 sec
D1             0.73952258 sec
D6             0.05000000 sec
D16            0.00020000 sec
IN0            0.00001380 sec

======== CHANNEL f1 ========
NUC1                   1H
P1                  11.40 usec
P2                  22.80 usec
PL1                  1.00 dB
PL1W          13.76731014 W
SFO1          600.1324312 MHz

======== CHANNEL f2 ========
NUC2                  13C
P3                   9.80 usec
P24               2000.00 usec
PL2                  5.00 dB
PL2W          26.76886177 W
SFO2          150.9178993 MHz
SP7                 13.33 dB
SPNAM7        Crp60comp.4
SPOAL7              0.500
SPOFFS7              0.00 Hz

====== GRADIENT CHANNEL =====
GPNAM1           SINE.100
GPNAM3           SINE.100
GPNAM4           SINE.100
GPNAM5           SINE.100
GPNAM6           SINE.100
GPZ1                80.00 %
GPZ3                14.00 %
GPZ4                -8.00 %
GPZ5                -4.00 %
GPZ6                -2.00 %
P16               1000.00 usec
ND0                     2
TD                    256
SFO1             150.9179 MHz
FIDRES         141.485535 Hz
SW                240.000 ppm
FnMODE       Echo-Antiecho
SI                   2048
SF            600.1300106 MHz
WDW                  SINE
SSB                     2
LB                   0.00 Hz
GB                      0
PC                   1.40
SI                   1024
MC2          echo-antiecho
SF            150.9027756 MHz
WDW                  SINE
SSB                     2
LB                   0.00 Hz
GB                      0
```

**JC–814–3**
**PROTON.ucl CDCl3 {V:\Bruker\TOPSPIN} mjp 21**

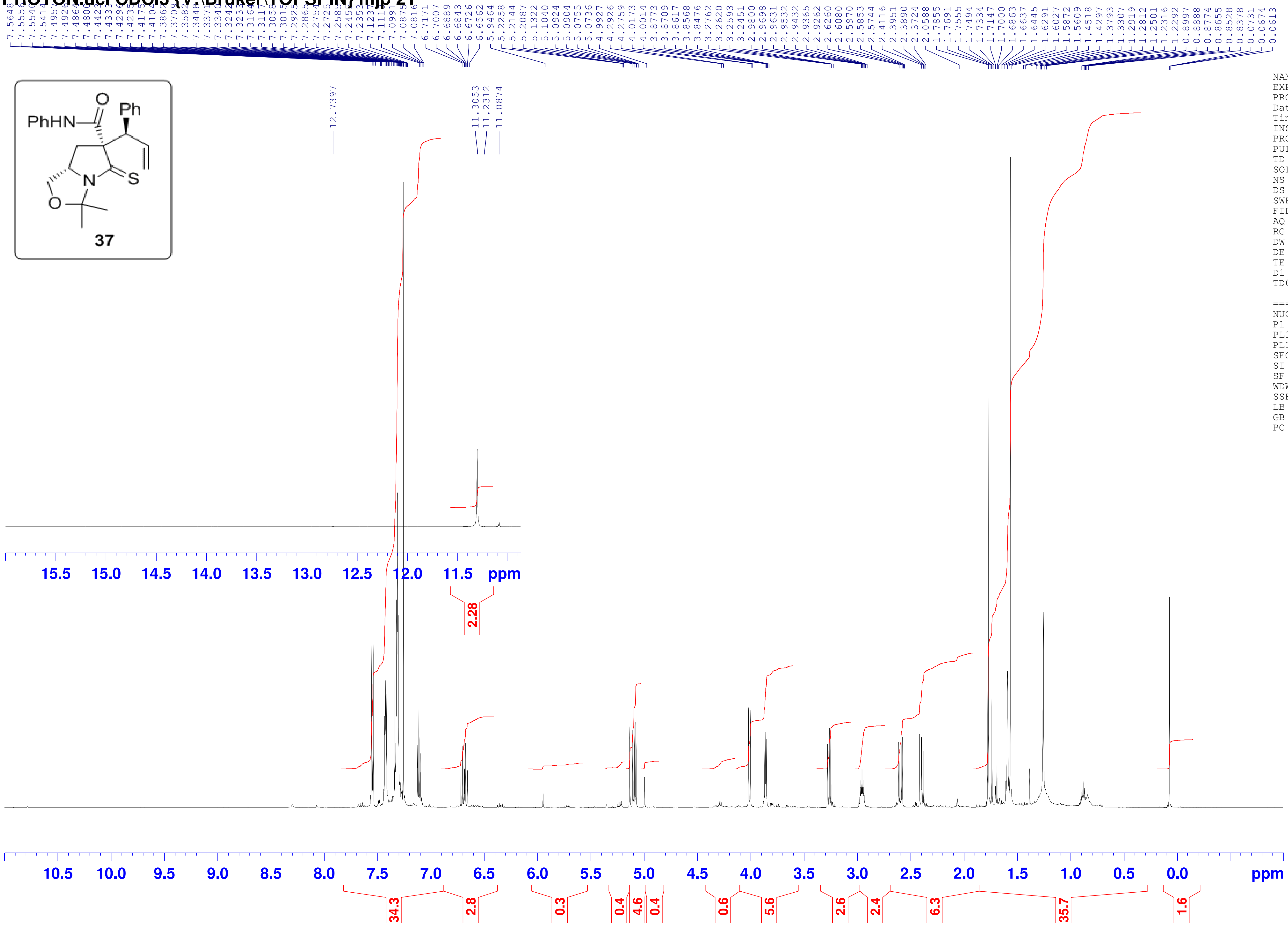


| | |
|---|---|
| NAME | JC-814-3 |
| EXPNO | 10 |
| PROCNO | 1 |
| Date_ | 20120616 |
| Time | 16.07 |
| INSTRUM | AV600 |
| PROBHD | 5 mm CPDCH 13C |
| PULPROG | zg30 |
| TD | 98682 |
| SOLVENT | CDCl3 |
| NS | 8 |
| DS | 0 |
| SWH | 12335.526 Hz |
| FIDRES | 0.125003 Hz |
| AQ | 3.9999604 sec |
| RG | 28.5 |
| DW | 40.533 usec |
| DE | 10.48 usec |
| TE | 298.0 K |
| D1 | 1.00000000 sec |
| TD0 | 1 |
| ======== CHANNEL f1 ===== | |
| NUC1 | 1H |
| P1 | 11.40 usec |
| PL1 | 1.00 dB |
| PL1W | 13.76731014 W |
| SFO1 | 600.1337061 MHz |
| SI | 32768 |
| SF | 600.1300116 MHz |
| WDW | EM |
| SSB | 0 |
| LB | 0.30 Hz |
| GB | 0 |
| PC | 1.40 |

V:BrukerTOPSPIN/mjp/JC–814–3/10/1
Integral regions #3 to #6

JC–814–3
PROTON.ucl CDCl3 {V:\Bruker\TOPSPIN} mjp 21

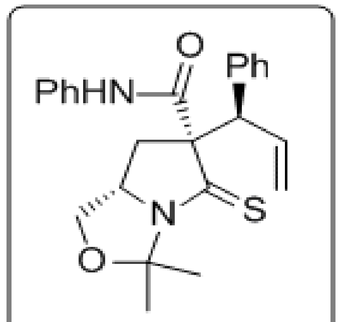


37

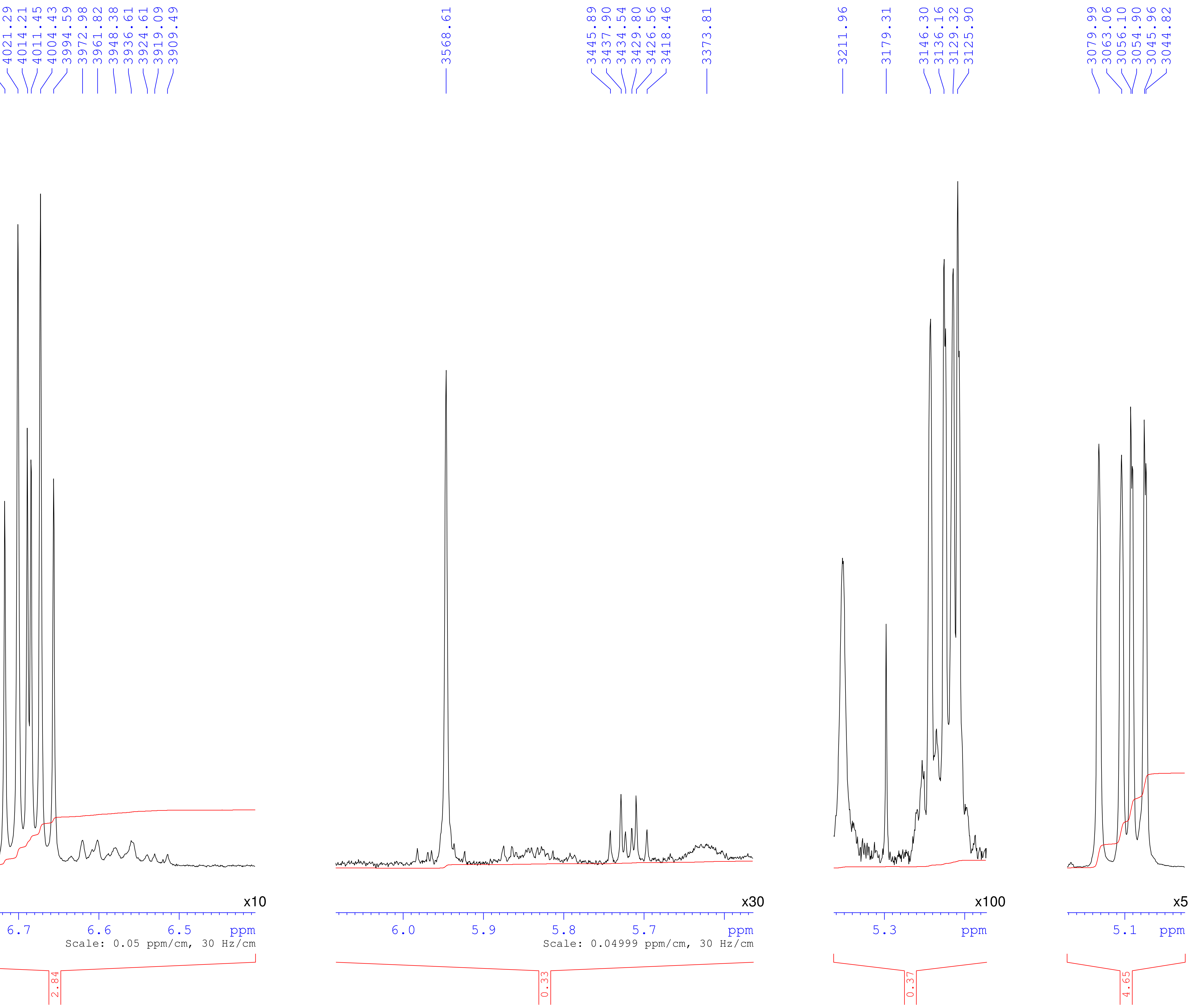

V:BrukerTOPSPIN/mjp/JC–814–3/10/1
Integral regions #7 to #10

# JC–814–3
PROTON.ucl CDCl3 {V:\Bruker\TOPSPIN} mjp 21

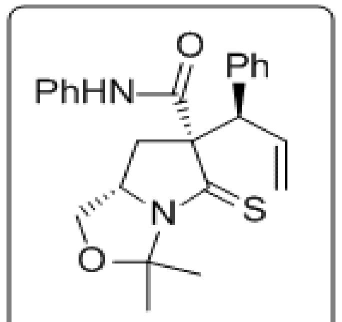


**37**

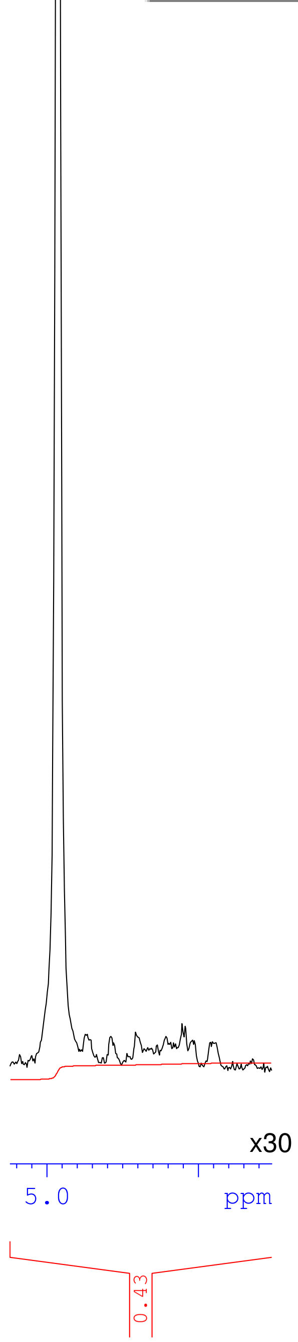


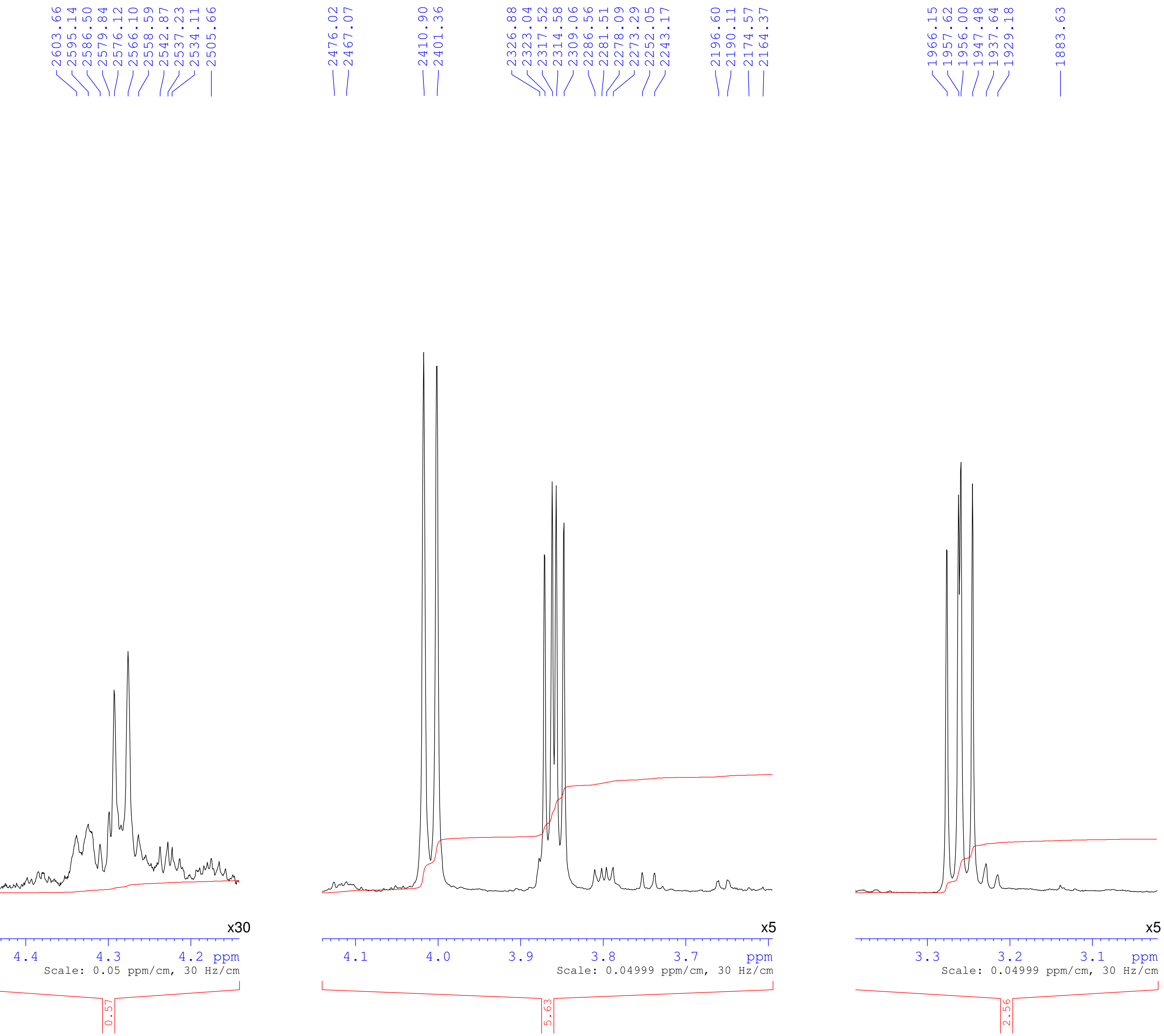

V:BrukerTOPSPIN/mjp/JC–814–3/10/1
Integral regions #11 to #12

JC–814–3
PROTON.ucl CDCl3 {V:\Bruker\TOPSPIN} mjp 21

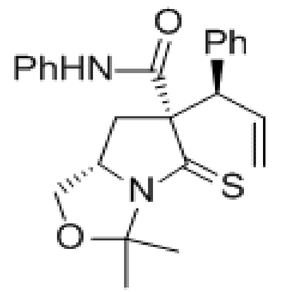


37

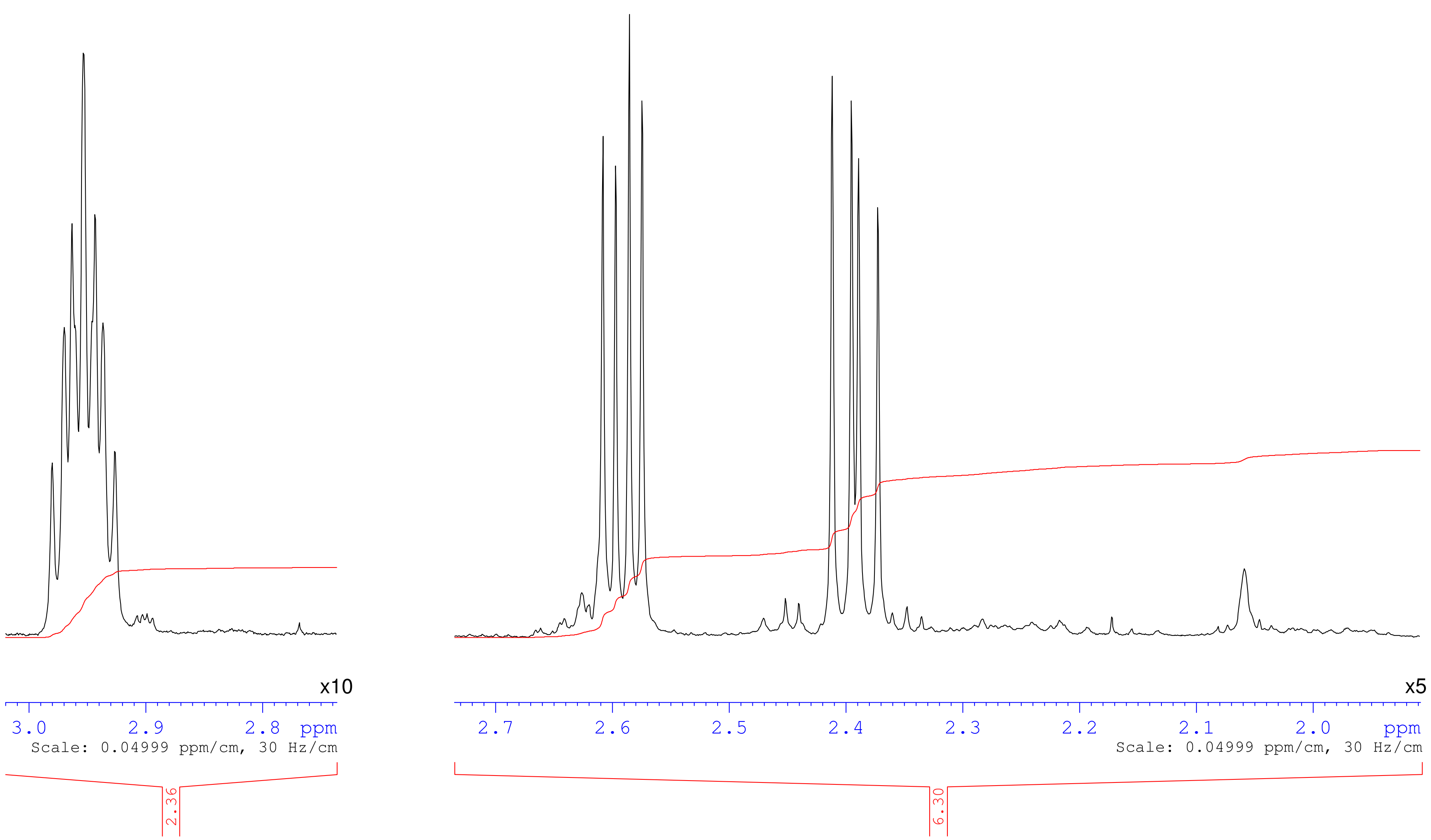

JC–814–3
C13CPD.ucl CDCl3 {V:\Bruker\TOPSPIN} mjp 21

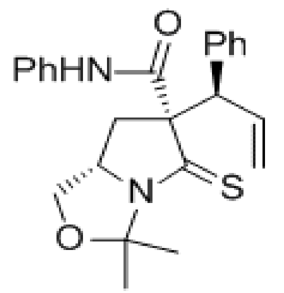


**37**

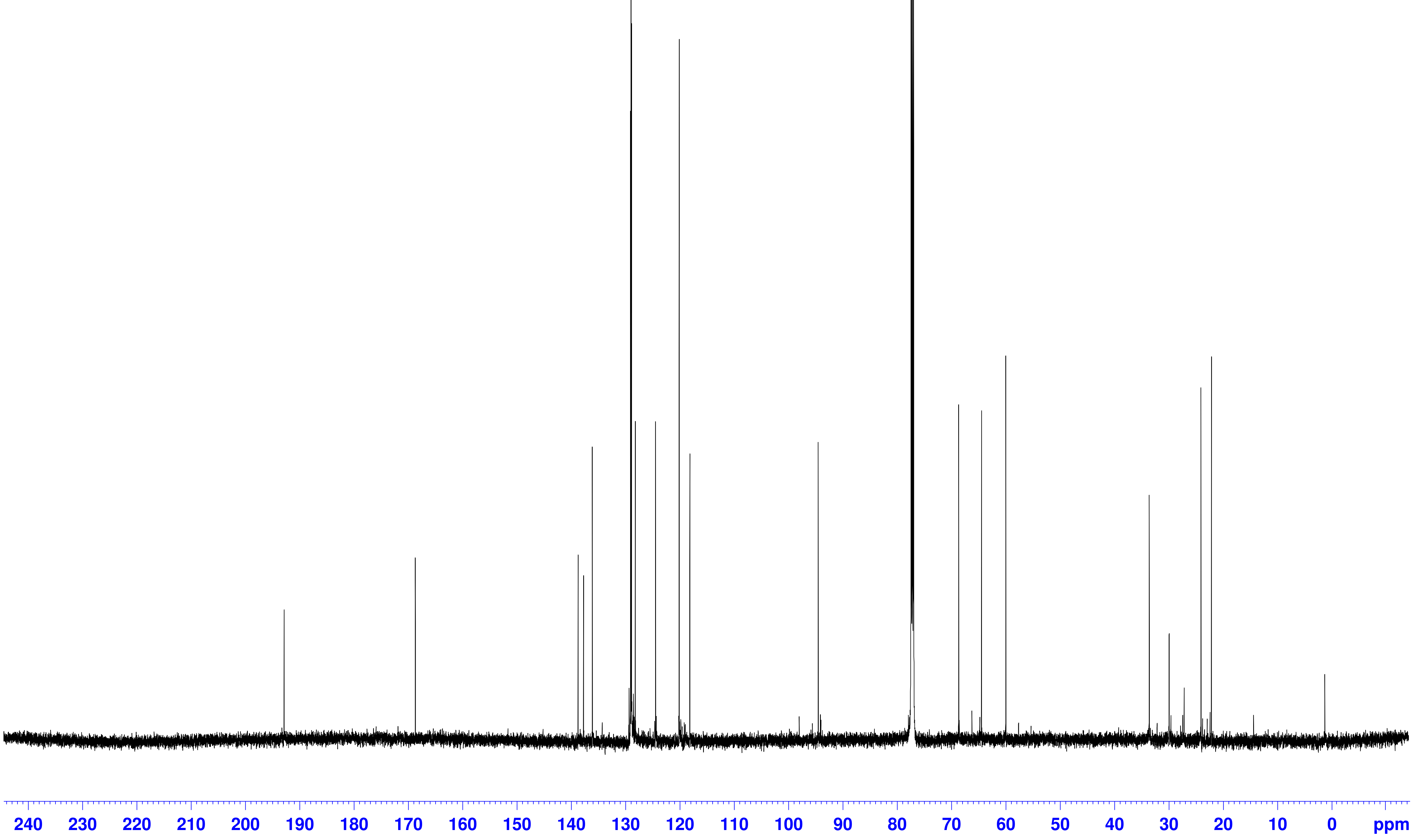


UCL

| | |
|---|---|
| NAME | JC-814-3 |
| EXPNO | 12 |
| PROCNO | 1 |
| Date_ | 20120616 |
| Time | 16.18 |
| INSTRUM | AV600 |
| PROBHD | 5 mm CPDCH 13C |
| PULPROG | zgpg30 |
| TD | 70308 |
| SOLVENT | CDCl3 |
| NS | 128 |
| DS | 0 |
| SWH | 39062.500 H |
| FIDRES | 0.555591 H |
| AQ | 0.8999924 s |
| RG | 1030 |
| DW | 12.800 u |
| DE | 21.12 u |
| TE | 298.0 K |
| D1 | 2.00000000 s |
| D11 | 0.03000000 s |
| TD0 | 1 |
| ======== CHANNEL f1 ===== | |
| NUC1 | 13C |
| P1 | 9.80 u |
| PL1 | 5.00 d |
| PL1W | 26.76886177 W |
| SFO1 | 150.9201628 M |
| ======== CHANNEL f2 ===== | |
| CPDPRG2 | waltz16 |
| NUC2 | 1H |
| PCPD2 | 70.00 u |
| PL2 | 1.00 d |
| PL12 | 17.23 d |
| PL13 | 20.00 d |
| PL2W | 13.76731014 W |
| PL12W | 0.32798135 W |
| PL13W | 0.17332016 W |
| SFO2 | 600.1324005 M |
| SI | 65536 |
| SF | 150.9027930 M |
| WDW | EM |
| SSB | 0 |
| LB | 1.00 H |
| GB | 0 |
| PC | 1.40 |

JC–814–3
COSY.ucl CDCl3 {V:\Bruker\TOPSPIN} mjp 21

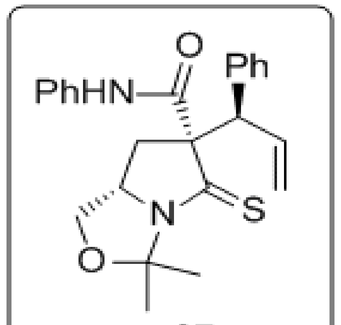


37

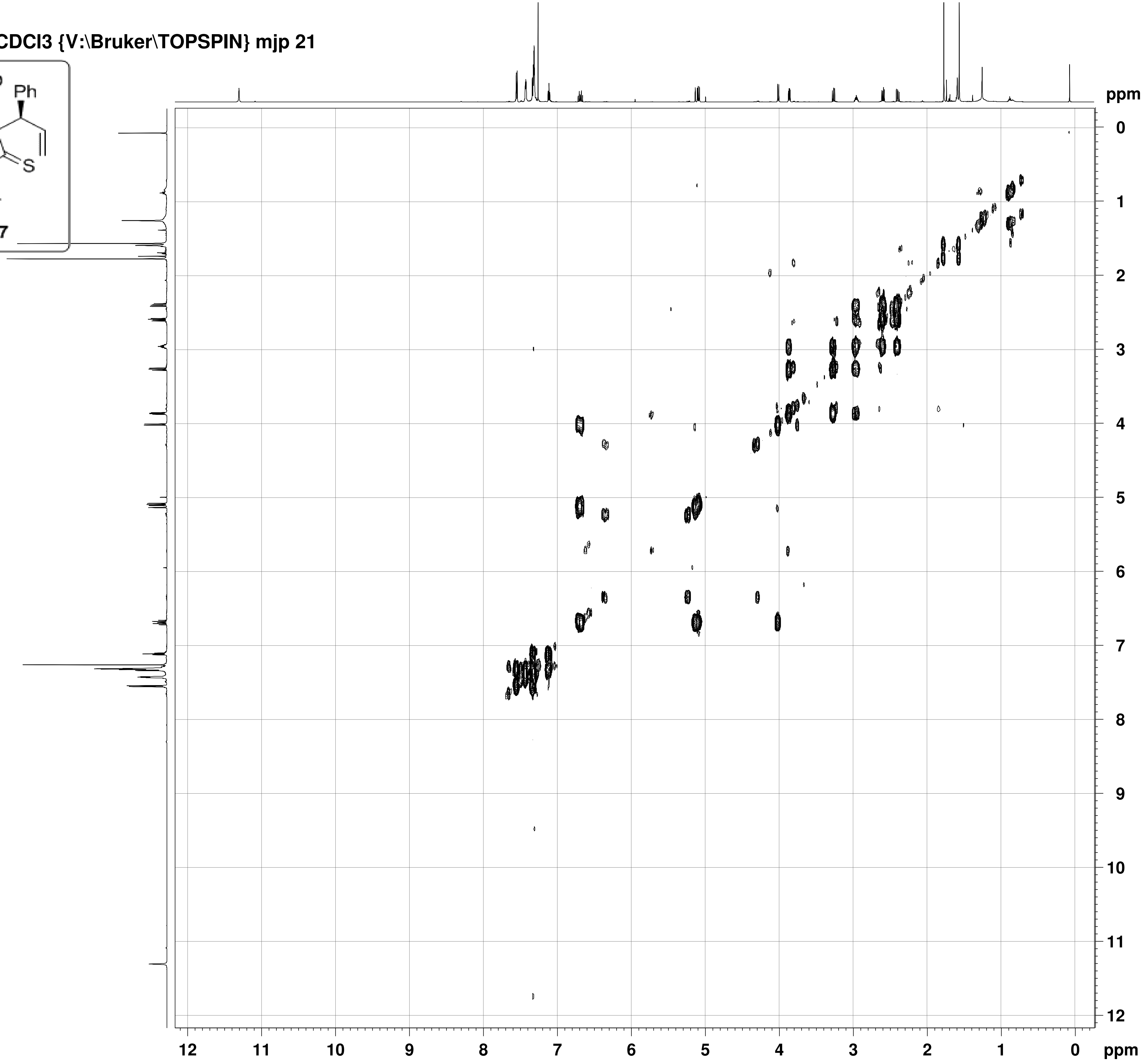


| | |
|---|---|
| NAME | JC-814-3 |
| EXPNO | 11 |
| PROCNO | 1 |
| Date_ | 20120616 |
| Time | 16.07 |
| INSTRUM | AV600 |
| PROBHD | 5 mm CPDCH 13C |
| PULPROG | cosygpmfqf |
| TD | 2048 |
| SOLVENT | CDCl3 |
| NS | 1 |
| DS | 8 |
| SWH | 7462.687 Hz |
| FIDRES | 3.643890 Hz |
| AQ | 0.1372660 sec |
| RG | 2050 |
| DW | 67.000 usec |
| DE | 6.50 usec |
| TE | 298.0 K |
| D0 | 0.00000300 sec |
| D1 | 1.76284099 sec |
| D13 | 0.00000400 sec |
| D16 | 0.00020000 sec |
| IN0 | 0.00013400 sec |
| ======== CHANNEL f1 ======== | |
| NUC1 | 1H |
| P1 | 11.40 usec |
| PL1 | 1.00 dB |
| PL1W | 13.76731014 W |
| SFO1 | 600.1335837 MHz |
| ====== GRADIENT CHANNEL ==== | |
| GPNAM1 | SINE.100 |
| GPNAM2 | SINE.100 |
| GPNAM3 | SINE.100 |
| GPZ1 | 16.00 % |
| GPZ2 | 12.00 % |
| GPZ3 | 40.00 % |
| P16 | 1000.00 usec |
| ND0 | 1 |
| TD | 128 |
| SFO1 | 600.1336 MHz |
| FIDRES | 58.302238 Hz |
| SW | 12.435 ppm |
| FnMODE | QF |
| SI | 1024 |
| SF | 600.1300081 MHz |
| WDW | QSINE |
| SSB | 0 |
| LB | 0.00 Hz |
| GB | 0 |
| PC | 1.40 |
| SI | 1024 |
| MC2 | QF |
| SF | 600.1300081 MHz |
| WDW | QSINE |
| SSB | 0 |
| LB | 0.00 Hz |
| GB | 0 |

**JC–814–3**
**C13DEPT135.ucl CDCl3 {V:\Bruker\TOPSPIN} mjp 21**

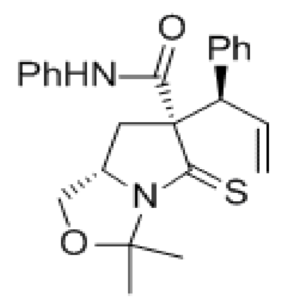


**37**

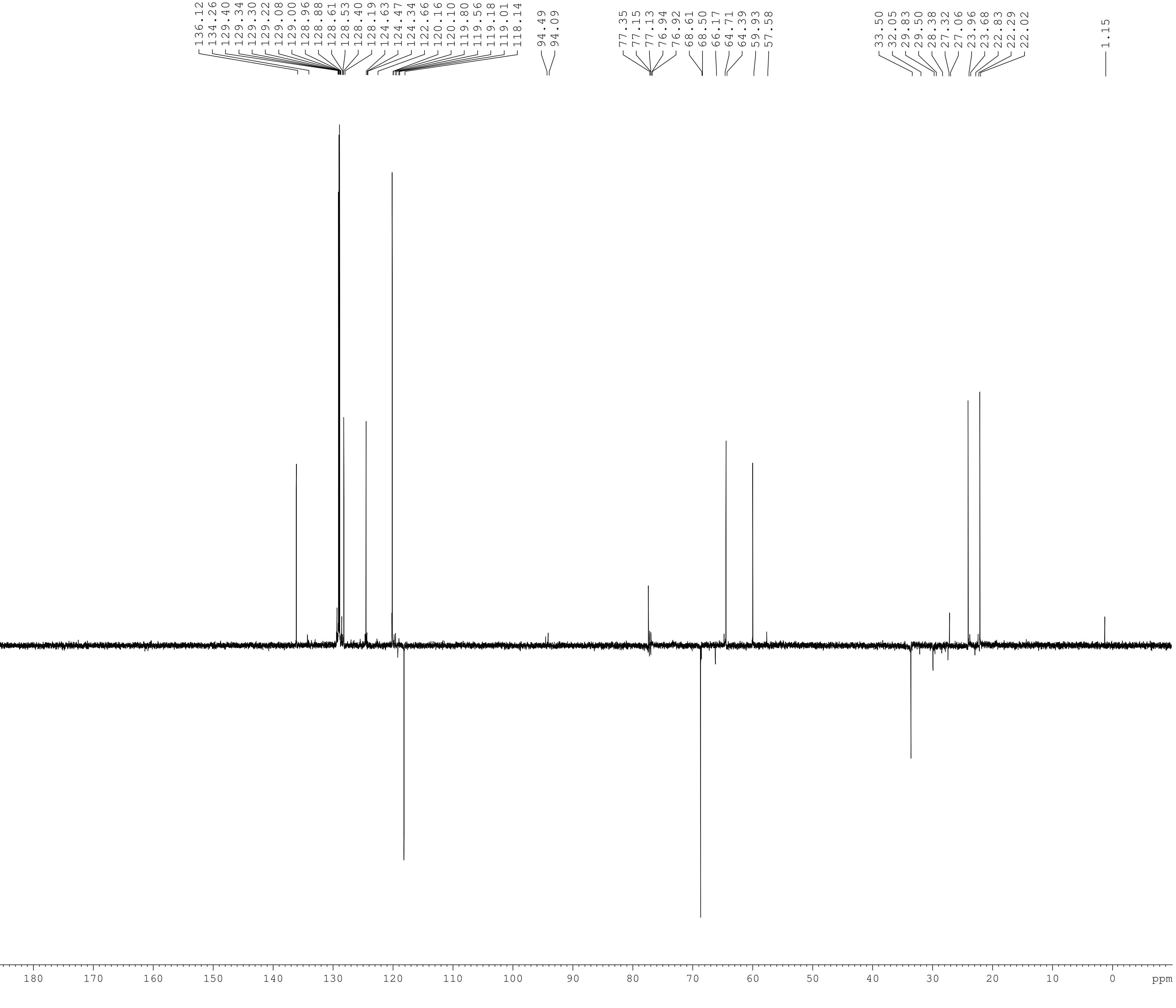


UCL

| | |
|---|---|
| NAME | JC-814-3 |
| EXPNO | 15 |
| PROCNO | 1 |
| Date_ | 20120616 |
| Time | 16.41 |
| INSTRUM | AV600 |
| PROBHD | 5 mm CPDCH 13C |
| PULPROG | dept135 |
| TD | 70308 |
| SOLVENT | CDCl3 |
| NS | 64 |
| DS | 4 |
| SWH | 39062.500 H |
| FIDRES | 0.555591 H |
| AQ | 0.8999924 s |
| RG | 256 |
| DW | 12.800 u |
| DE | 6.50 u |
| TE | 298.0 K |
| CNST2 | 145.0000000 |
| D1 | 2.00000000 s |
| D2 | 0.00344828 s |
| D12 | 0.00002000 s |
| TD0 | 1 |
| ======== CHANNEL f1 ===== | |
| NUC1 | 13C |
| P1 | 9.80 u |
| P2 | 19.60 u |
| PL1 | 5.00 d |
| PL1W | 26.76886177 W |
| SFO1 | 150.9201628 M |
| ======== CHANNEL f2 ===== | |
| CPDPRG2 | waltz16 |
| NUC2 | 1H |
| P3 | 10.80 u |
| P4 | 21.60 u |
| PCPD2 | 70.00 u |
| PL2 | 1.00 d |
| PL12 | 17.23 d |
| PL2W | 13.76731014 W |
| PL12W | 0.32798135 W |
| SFO2 | 600.1324005 M |
| SI | 65536 |
| SF | 150.9027930 M |
| WDW | EM |
| SSB | 0 |
| LB | 1.00 H |
| GB | 0 |
| PC | 1.40 |

**JC–814–3**
**HSQC.ucl CDCl3 {V:\Bruker\TOPSPIN} mjp 21**

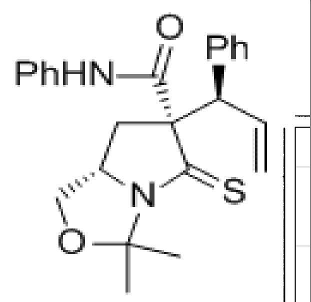


**37**

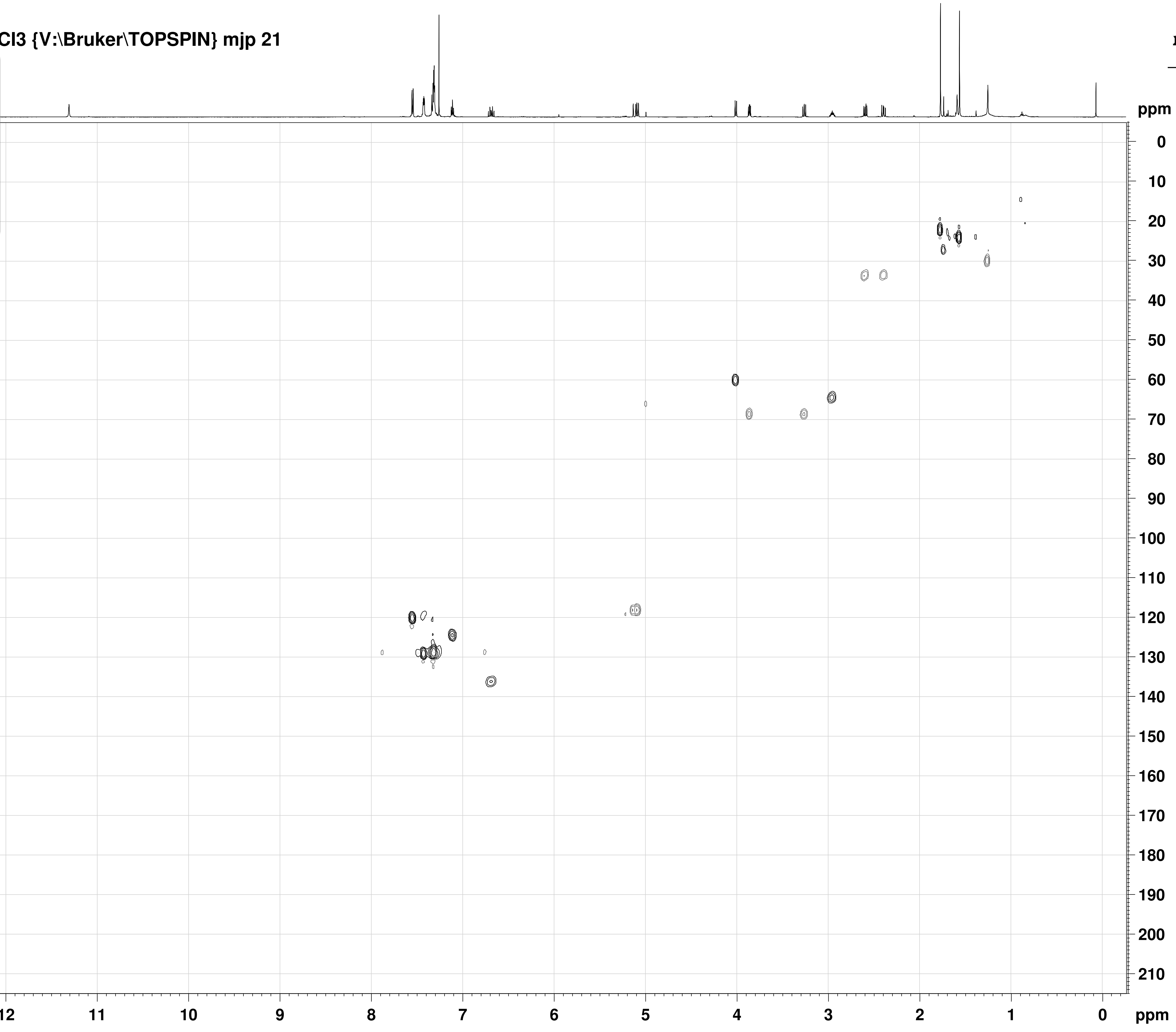


```
NAME             JC-814-3
EXPNO                  13
PROCNO                  1
Date_            20120616
Time                16.19
INSTRUM             AV600
PROBHD   5 mm CPDCH 13C
PULPROG  hsqcedetgpsisp2.4
TD                   1024
SOLVENT             CDCl3
NS                      2
DS                     32
SWH              7462.687 Hz
FIDRES           7.287780 Hz
AQ              0.0686580 sec
RG                   2050
DW                 67.000 usec
DE                   6.50 usec
TE                  298.0 K
CNST2         145.0000000
CNST17         -0.5000000
D0             0.00000300 sec
D1             1.51658905 sec
D2             0.00344828 sec
D4             0.00172414 sec
D11            0.03000000 sec
D16            0.00020000 sec
D21            0.00344828 sec
D24            0.00086207 sec
IN0            0.00001505 sec
L0                      0
L31                     1
TD0                     2

======== CHANNEL f1 ========
NUC1                   1H
P1                  11.40 usec
P2                  22.80 usec
P28                  0.00 usec
PL1                  1.00 dB
PL1W          13.76731014 W
SFO1          600.1335837 MHz

======== CHANNEL f2 ========
CPDPRG2  bi_p5m4sp_4sp.2
NUC2                  13C
P3                   9.80 usec
P14                500.00 usec
P24               2000.00 usec
P31               1730.00 usec
P63               1500.00 usec
PL0                120.00 dB
PL2                  5.00 dB
PL12                20.74 dB
PL0W           0.00000000 W
PL2W          26.76886177 W
PL12W          0.71388775 W
SFO2          150.9186538 MHz
SP3                 13.33 dB
SP7                 13.33 dB
SP14                14.82 dB
SP18                18.73 dB
SP31                20.84 dB
SPNAM3    Crp60,0.5,20.1
SPNAM7       Crp60comp.4
SPNAM14   Crp32,1.5,20.2
SPNAM18    Crp60_xfilt.2
SPNAM31   Crp32,1.5,20.2
SPOAL3              0.500
SPOAL7              0.500
SPOAL14             0.500
SPOAL18             0.500
SPOAL31             0.500
SPOFFS3              0.00 Hz
SPOFFS7              0.00 Hz
SPOFFS14             0.00 Hz
SPOFFS18             0.00 Hz
SPOFFS31             0.00 Hz

====== GRADIENT CHANNEL =====
GPNAM1           SINE.100
GPNAM2           SINE.100
GPNAM3           SINE.100
GPNAM4           SINE.100
GPZ1                80.00 %
GPZ2                20.10 %
GPZ3                11.00 %
GPZ4                -5.00 %
P16               1000.00 usec
P19                600.00 usec
ND0                     2
TD                    128
SFO1             150.9187 MHz
FIDRES         259.391449 Hz
SW                220.000 ppm
FnMODE       Echo-Antiecho
SI                   1024
SF            600.1300081 MHz
WDW                 QSINE
SSB                     2
LB                   0.00 Hz
GB                      0
PC                   1.40
SI                   1024
MC2           echo-antiecho
SF            150.9027771 MHz
WDW                 QSINE
SSB                     2
LB                   0.00 Hz
GB                      0
```

**JC–814–3**
**HMBC.ucl CDCl3 {V:\Bruker\TOPSPIN} mjp 21**

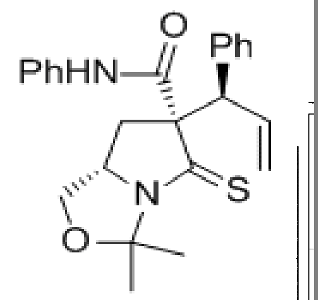


**37**

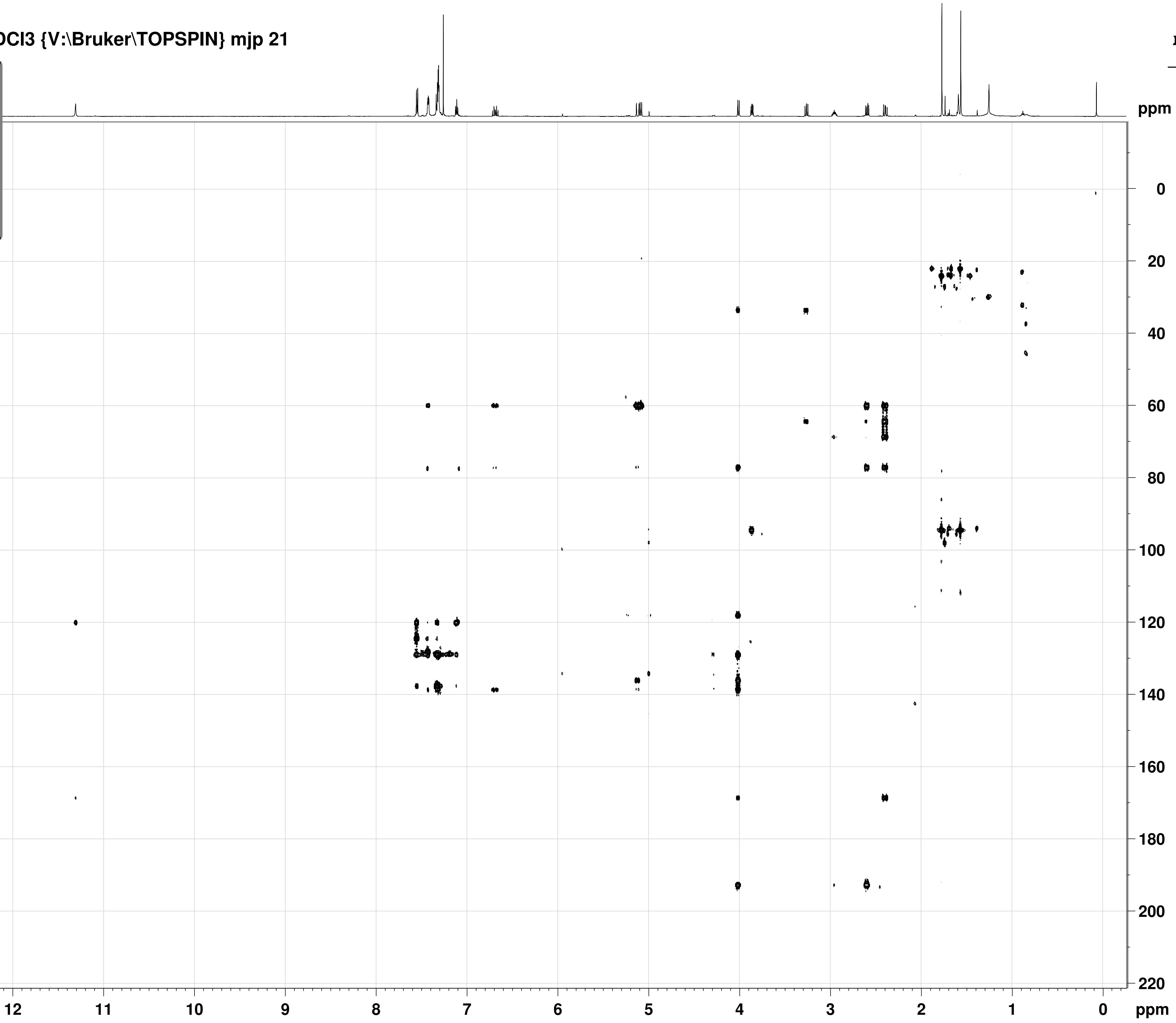


```
NAME              JC-814-3
EXPNO                   14
PROCNO                   1
Date_             20120616
Time                 16.27
INSTRUM              AV600
PROBHD   5 mm CPDCH 13C
PULPROG     hmbcetgpl3nd
TD                    4096
SOLVENT              CDCl3
NS                       2
DS                      16
SWH              7462.687 Hz
FIDRES           1.821945 Hz
AQ              0.2744820 sec
RG                    2050
DW                  67.000 usec
DE                    6.50 usec
TE                   298.0 K
CNST6          120.0000000
CNST7          160.0000000
CNST13          10.0000000
CNST30           0.5981157
D0              0.00000300 sec
D1              0.90336257 sec
D6              0.05000000 sec
D16             0.00020000 sec
IN0             0.00001380 sec

======== CHANNEL f1 ========
NUC1                    1H
P1                   11.40 usec
P2                   22.80 usec
PL1                   1.00 dB
PL1W           13.76731014 W
SFO1           600.1335837 MHz

======== CHANNEL f2 ========
NUC2                   13C
P3                    9.80 usec
P24                2000.00 usec
PL2                   5.00 dB
PL2W           26.76886177 W
SFO2           150.9178993 MHz
SP7                  13.33 dB
SPNAM7         Crp60comp.4
SPOAL7               0.500
SPOFFS7               0.00 Hz

====== GRADIENT CHANNEL =====
GPNAM1            SINE.100
GPNAM3            SINE.100
GPNAM4            SINE.100
GPNAM5            SINE.100
GPNAM6            SINE.100
GPZ1                 80.00 %
GPZ3                 14.00 %
GPZ4                 -8.00 %
GPZ5                 -4.00 %
GPZ6                 -2.00 %
P16                1000.00 usec
ND0                      2
TD                     256
SFO1              150.9179 MHz
FIDRES          141.485535 Hz
SW                 240.000 ppm
FnMODE        Echo-Antiecho
SI                    2048
SF             600.1300100 MHz
WDW                   SINE
SSB                      2
LB                    0.00 Hz
GB                       0
PC                    1.40
SI                    1024
MC2           echo-antiecho
SF             150.9027756 MHz
WDW                   SINE
SSB                      2
LB                    0.00 Hz
GB                       0
```
